# Integrating transcriptomic network reconstruction and eQTL analyses reveals mechanistic connections between genomic architecture and *Brassica rapa* development

**DOI:** 10.1371/journal.pgen.1008367

**Published:** 2019-09-12

**Authors:** Robert L. Baker, Wen Fung Leong, Marcus T. Brock, Matthew J. Rubin, R. J. Cody Markelz, Stephen Welch, Julin N. Maloof, Cynthia Weinig

**Affiliations:** 1 Department of Biology, Miami University, Oxford, Ohio, United States of America; 2 Department of Agronomy, Kansas State University, Manhattan, Kansas, United States of America; 3 Department of Botany, University of Wyoming, Laramie, Wyoming, United States of America; 4 Department of Plant Biology, University of California Davis, Davis, California, United States of America; Eidgenossische Technische Hochschule Zurich, SWITZERLAND

## Abstract

Plant developmental dynamics can be heritable, genetically correlated with fitness and yield, and undergo selection. Therefore, characterizing the mechanistic connections between the genetic architecture governing plant development and the resulting ontogenetic dynamics of plants in field settings is critically important for agricultural production and evolutionary ecology. We use hierarchical Bayesian Function-Valued Trait (FVT) models to estimate *Brassica rapa* growth curves throughout ontogeny, across two treatments, and in two growing seasons. We find genetic variation for plasticity of growth rates and final sizes, but not the inflection point (transition from accelerating to decelerating growth) of growth curves. There are trade-offs between growth rate and duration, indicating that selection for maximum yields at early harvest dates may come at the expense of late harvest yields and vice versa. We generate eigengene modules and determine which are co-expressed with FVT traits using a Weighted Gene Co-expression Analysis. Independently, we seed a Mutual Rank co-expression network model with FVT traits to identify specific genes and gene networks related to FVT. GO-analyses of eigengene modules indicate roles for actin/cytoskeletal genes, herbivore resistance/wounding responses, and cell division, while MR networks demonstrate a close association between metabolic regulation and plant growth. We determine that combining FVT Quantitative Trait Loci (QTL) and MR genes/WGCNA eigengene expression profiles better characterizes phenotypic variation than any single data type (i.e. QTL, gene, or eigengene alone). Our network analysis allows us to employ a targeted eQTL analysis, which we use to identify regulatory hotspots for FVT. We examine *cis* vs. *trans* eQTL that mechanistically link FVT QTL with structural trait variation. Colocalization of FVT, gene, and eigengene eQTL provide strong evidence for candidate genes influencing plant height. The study is the first to explore eQTL for FVT, and specifically do so in agroecologically relevant field settings.

## Author summary

We estimate the developmental dynamics of plant growth using mathematical functions to fit continuous functions to discrete plant height data collected throughout growth, and we use the parameters defining these mathematical functions as data. We identify genomic regions controlling plant growth and filter a novel transcriptomic data set using network reconstruction models to identify the genes and eigengenes associated with plant height. We combine these genomic and transcriptomic data to predict variation in plant height, and we use quantitative genetics to mechanistically connect plant genetics, transcriptomics, and development. Our approach demonstrates two powerful methods for the type of data reduction (FVT modeling and gene expression network reconstruction for targeted eQTL analyses) and data integration that will be necessary for driving forward the field of genetics in the post-genomic era. To the best of our knowledge, we are the first to apply these techniques to continuous models of plant development, and the first to do so in agroecologically relevant field settings.

## Introduction

Plant developmental dynamics are correlated with fitness and yield [1,2]. Therefore, characterizing the mechanistic connections between the genetic architecture governing plant development and the resulting ontogenetic dynamics of plants in field settings is critically important to improving agricultural production and understanding evolutionary fitness. Forward genetic approaches such as quantitative trait mapping are an attractive method of characterizing genetic architecture because they do not require *a priori* information such as candidate loci and can be used to describe additive effects as well as pleiotropic and epistatic loci [3–5]. Transcriptomic co-expression analyses and expression QTL (eQTL) have also been used to identify the underlying genetic architecture responsible for phenotypic variation [e.g. 6]. Recently, combining information from genomic association studies and transcriptomic expression analyses has been used to pinpoint candidate genes [7–10]. However, co-expression network analyses can also provide insight into the mechanistic connections between QTL genotypes and phenotypes. Here, we ask whether QTL, co-expression analyses, or a combination thereof best predict phenotypic variation. In combination with a targeted eQTL analyses in agroecologically relevant field settings, we characterize the mechanistic connections between the genomic architecture, transcriptomic expression networks, and phenotypic variation throughout plant development.

Development rarely occurs in discrete steps, yet developmental data are typically collected at multiple distinct but inter-dependent time points. Function-Valued Trait (FVT) modeling is one method of estimating the underlying continuous nature of development and avoiding complicated repeated measures statistics, which often compromise statistical power in downstream analyses [11,12]. One approach to FVT modeling involves fitting mathematical functions to discrete data to estimate continuous curves that represent the change of a trait or character as a function, typically of time [13–15]. Although there are multiple approaches to modeling continuous growth, one particular advantage of FVT modeling is that parameters describing developmental growth curves can be extracted from the FVT models and used as biologically interpretable and inter-relatable traits such as the relationship between growth rates, durations, inflection points denoting cessation of growth, and final sizes. This ‘parameters as data’ approach enables a broad array of analyses at both genetic and phenotypic levels [2,16]. In the current study, we employ a Bayesian hierarchical approach to FVT modeling that utilizes global information from the entire dataset as well as each genotype to estimate replicate-level parameters describing growth curves that underlie the developmental dynamics of plant height.

One inherent but seldom addressed complication in studying developmental genetics is that development of a given trait rarely occurs independently of organism-level attributes. For instance, in plants the pool of available carbon can severely limit and alter organismal-level development including aspects of determinate structures such as leaves [17,18] and indeterminant growth such as plant height [19]. Further, including physiological parameters such as carbon assimilation in plant breeding models is predicted to accelerate and improve yield gains [20]. One solution is using a hierarchical Bayesian approach to FVT modeling that incorporates genotype-specific values for organism-level physiological conditions such as carbon availability (estimated, for instance, using maximum photosynthetic capacity, A_max_) to statistically factor out variation caused by resource availability. Accounting for carbon availability in FVT parameter estimation can increase estimates of heritability and improve QTL mapping results [21,22].

QTL mapping provides a well-tested method of uncovering the genetic architecture of complex Function-Valued Traits (FVT). FVT variation may arise from structural or regulatory genes that differ among sampled genotypes. Examining gene expression can therefore provide insight into the mechanistic connections between genomic architecture and developmental dynamics of phenotypes [23–26]. We use Mutual Rank (MR) and Weighted Gene Co-expression Network Analyses (WGCNA) to identify expression networks associated with FVT trait variation. These networks are then used to focus our analysis to specific sub-sets of biologically relevant expression traits for eQTL mapping [27,28]. Interestingly, the genomic architecture of eQTL appears to depart from that of other phenotypic QTL such as FVT QTL in two important respects: first, gene expression traits tend to have only one or a few eQTL whereas morphological phenotypic traits are often highly polygenic [29]. Second, eQTL from multiple expression traits in diverse taxa from yeast to *Brassica* can be highly colocalized into eQTL “hotspots”. These hotspots may indicate a regulatory gene or switch that has a disproportionate impact on downstream gene expression [30–32]. In contrast, QTL for morphological traits may colocalize, but typically they do not do so to the same extent [31,33]. Whether general eQTL trends hold for targeted expression traits in agroecologically relevant field settings remains unknown. Further, to the best of our knowledge eQTL mapping has not been used to examine the mechanistic basis of developmental morphology captured via function-valued trait modeling.

Here, we estimate continuous developmental growth curves of plant height, a trait that when selected upon can lead to more effective increases in yield than directly selecting on yield itself [34], in a set of *Brassica rapa* Recombinant Inbred Lines (RILs) while mathematically factoring out the effects of carbon availability. We examine the patterns of genetic correlations among parameters describing change in height over time such as growth duration and final plant size, and we ask whether these developmental parameters correlate with yields. Using QTL mapping, we outline the genetic architecture of plant height development. Next, we use MR and WGCNA to identify genes and gene network modules whose expression patterns correlate with FVT parameters. We compare the predictive capacity of QTL and co-expression approaches in two ways: first, we test the relative effectiveness of QTL *vs*. MR genes *vs*. WGCNA module eigengenes (and combinations thereof) in explaining genetic variation of developmental traits. Second, we test whether QTL for FVT traits are enriched for genes identified via co-expression approaches. To explore the mechanistic basis of FVT QTL, we perform eQTL mapping on our MR genes and WGCNA module eigengenes. For eQTL and FVT QTL that colocalize, we explore the relative proportion *cis-* vs. *trans*-eQTL and their effect sizes. We ask whether eQTL colocalize to regulatory hotspots and if so how these compare to FVT QTL. Our eQTL analysis offers an additional line of inference for candidate gene identification as well as a potential mechanistic explanation for the regulation of yield-related FVT QTL.

## Results

### Function-Valued Traits (FVTs)

**Fig 1:**
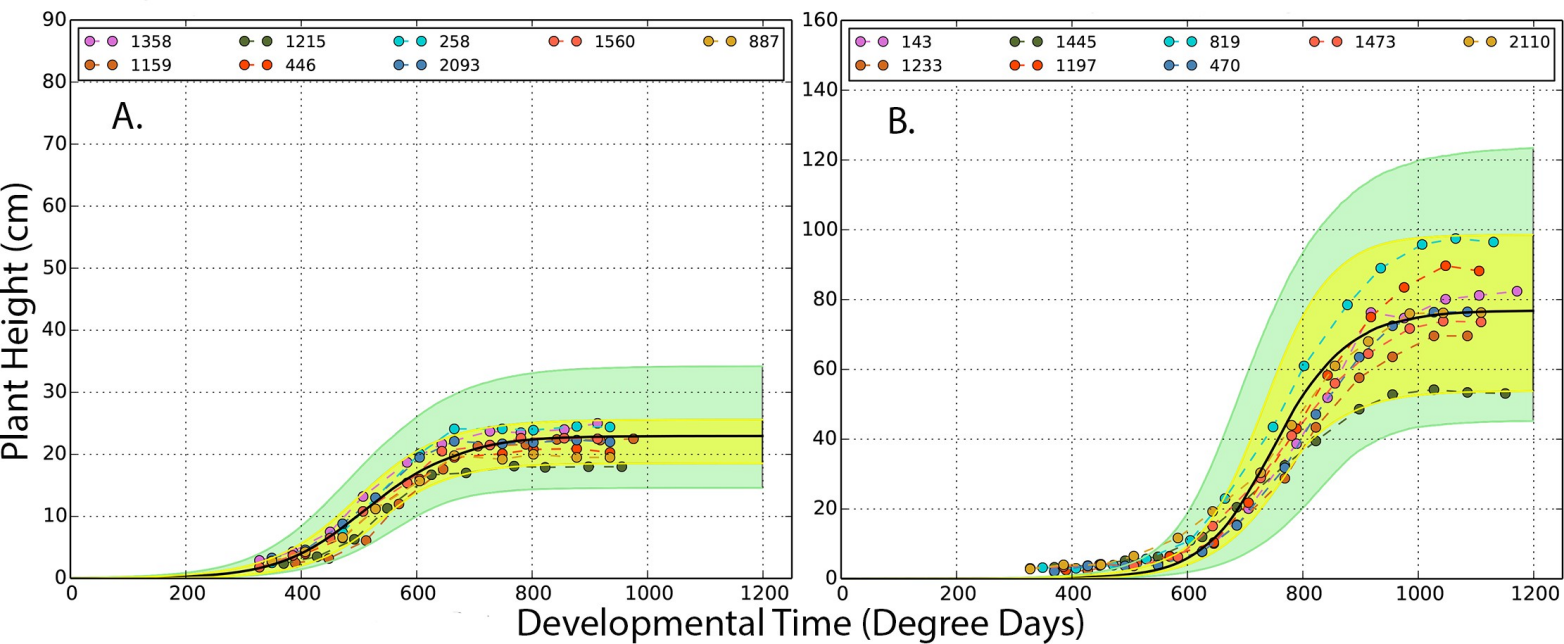
Representative genotypes (A, IMB211; B, R500) of Bayesian FVT trait estimation approaches for uncrowded plants from the 2012 season. Within each panel, dots represent observed data. Colors indicate replicates within each genotype and indicate that each replicate was measured multiple times throughout the growing season. The black line is the Bayesian estimate of logistic growth curve that best represents each genotype. The yellow envelope is a 95% credible envelope for the observed data; the green envelope is a 95% credible envelope for where new data is predicted to occur for a specific genotype and environment combination.

*Brassica rapa* Recombinant Inbred Lines from the IMB11xR500 cross were grown in the field in 2011 and 2012. In each year, there were two treatments: crowded and uncrowded. Multiple replicates of the full RIL set were planted out for each combination of year and treatment. FVT modeling was conducted based on replicate-level data, the data were sufficient to support all aspects of the growth curves modeled, and the models fit the data well (Fig 1 for example model fits). Plots for all FVT models can be found in S1 Fig and a conceptual overview of the analyses performed is presented in S2 Fig.

### Phenotypic plasticity and heritability

To assess the effects of the environment on plastic growth responses, we analyzed raw replicate level data. Although there were main effects of Block (nested within treatment) and genotype (RIL ID) for all traits, there were no significant main effects of crowding (treatment; Table 1). However, there was genetic variation for a plastic response to crowding for all traits except iD (inflection time, in Degree Days; treatment-by-genotype interaction; Table 1).

**Table 1:** Phenotypic plasticity and heritabilities of FVT parameters. Block is nested within the Treatment effect. Treatment corresponds to the crowded and uncrowded treatments in 2012 and Genotype indicates RIL id. Significant effects are emphasized by bold text.

		Random effects–Chi Square value (degrees of freedom)				Heritabilities (%)	
Trait	Model t-value (df)	Block (Treat)	Treatment	Genotype	Treatment × Genotype	UN 2012	CR 2012
**r**	16.62 (1.08) *	80.2 (2) ***	7.28e-12 (1) NS	136 (1) ***	211 (1) ***	74.5	76.0
**d**	43.32 (1.57) **	58.5 (2) ***	3.64e-12 (1) NS	294 (1) ***	4.88 (1) *	79.5	79.3
**iD**	37.16 (1.65) **	98.2 (2) ***	1.42e-10 (1) NS	369 (1) ***	0.34 (1) NS	86.8	83.7
**Hmax**	8.70 (1.83) *	116 (2) ***	0.0 (1) NS	226.4 (1) ***	42.3 (1) ***	81.2	68.1

Signif. codes: p < 0.001 ‘***’; p < 0.01 ‘**’; p < 0.05 ‘*’; p < 0.1 ‘.’; p > 0.1 ‘NS’

### Genetic correlations

**Fig 2:**
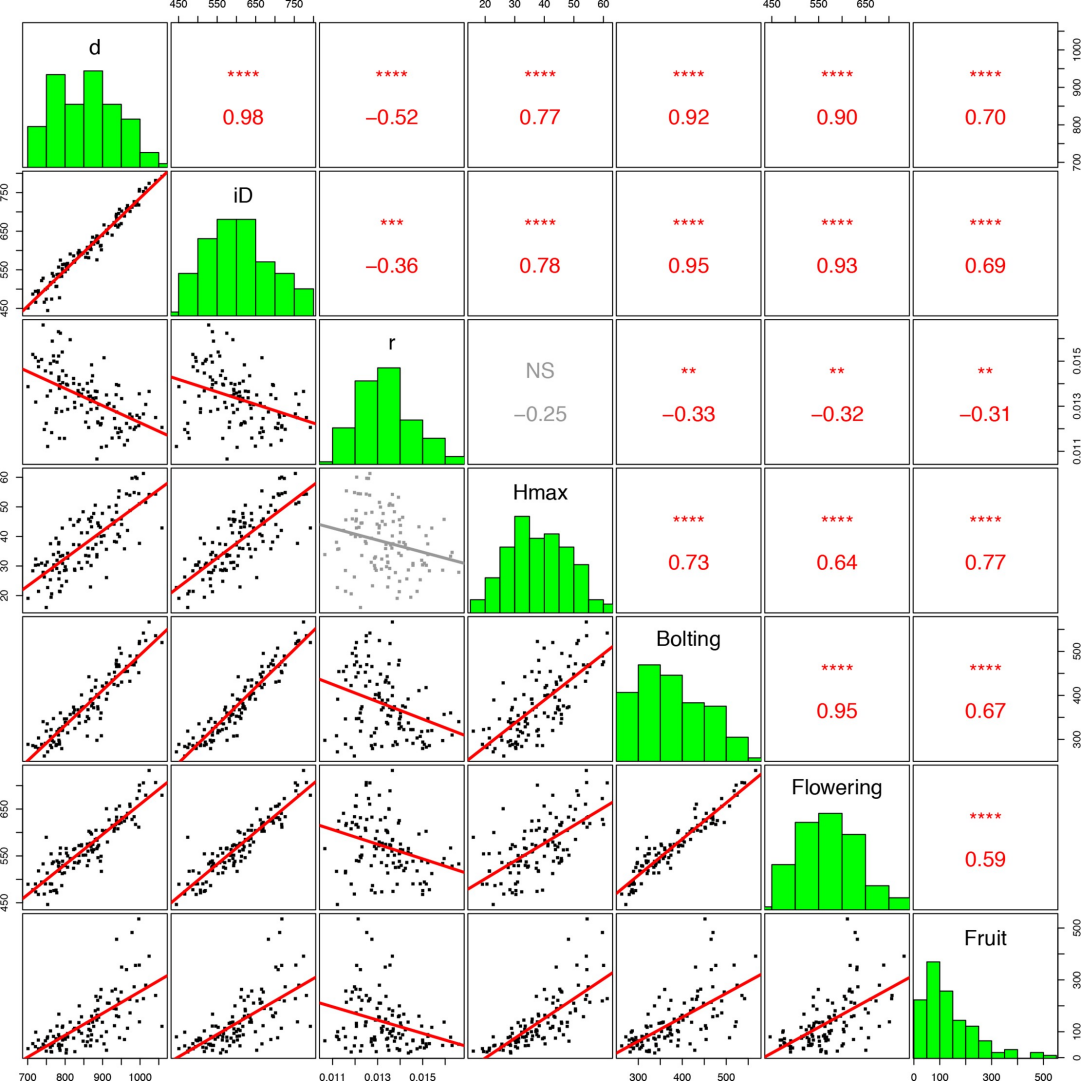
Genetic correlations among UN 2012 FVT height, phenology, and fitness traits. Each point is a genotypic mean (BLUP). Bonferroni corrections for multiple tests (n = 7) have been applied. Non-significant correlations are in gray. All time is expressed in Degree Days. * p<0.05, ** p<0.01, *** p<0.001, **** p<0.0001, NS p ≥ 0.05.

To explore the genetic relationships among the height FVT parameters and previously published estimates of plant phenology and fitness, we conducted a correlation analysis on BLUPs of each trait. In general, the pattern of genetic correlations within years and treatments was similar. Uncrowded (UN) r from 2012 was correlated with all traits except Hmax (Fig 2). In contrast, Crowded (CR) r in 2012 was negatively correlated with other all other 2012 CR FVT traits, with all CR phenology traits (except the bolting-to-flowering interval) and CR fitness traits (S3 Fig). UNr in 2012 was negatively correlated with UNd and iD but not Hmax. UNr 2012 was also negatively correlated with phenology and fitness. These patterns of genetic correlations are largely consistent across both years and treatments (S3 Fig); a representative subset of correlations from 2012 is presented in Fig 2.

### QTL mapping

**Fig 3:**
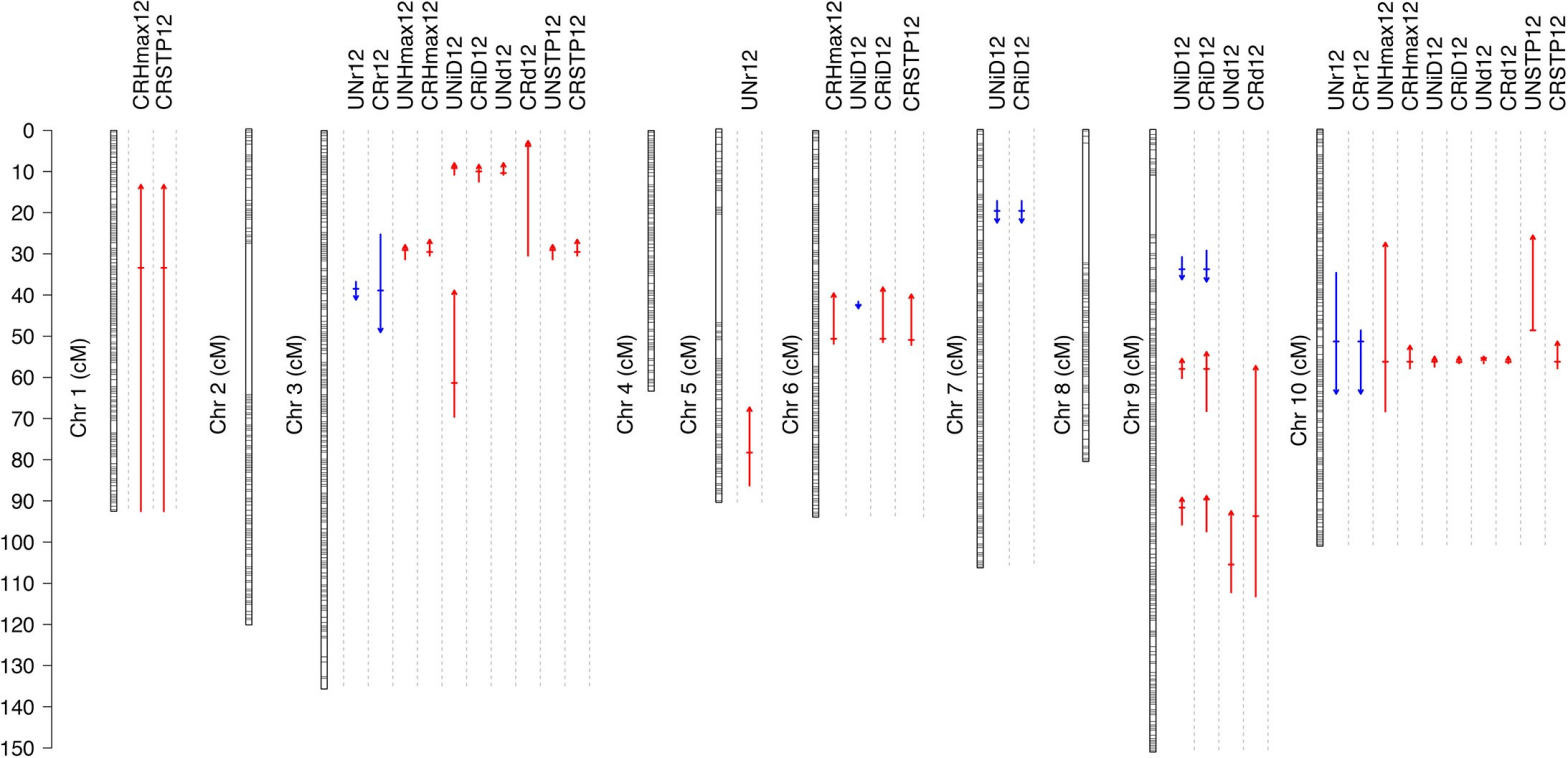
A map of all QTL identified in 2012. Horizontal lines on chromosomes indicate the position of RNAseq markers used to genetic map construction. Each QTL is indicated with a vertical arrow under the trait name. Horizontal hatches indicate QTL position, the arrow length indicates 1.5 LOD support limits. Arrow heads and color (up, red = positive; down, blue = negative) indicate QTL direction relative to the R500 parent. Exact locations, markers, and LOD scores for all QTL can be found in S1 (Table).

To further explore the genetic architecture of the height FVT parameters, we conducted QTL mapping analyses of the height FVT traits. In total we mapped 32 individual QTL from 2012 (2011 FVT QTL are presented in S1 Table); however, an alternative interpretation is that we mapped as few as 9 highly pleiotropic QTL. QTL were observed throughout the genome, except on chromosomes 2, 4, and 8. Most QTL localized to chromosome 3, 9 and 10. Across all traits, each QTL explained 29% of trait variation on average. The minimum explained variance was 9.5% and the maximum was 73% of variance (Fig 3 & S1 Table).

### Genes under FVT QTL

To determine positional candidates within mapped FVT QTL, we compared our FVT QTL to the *B*. *rapa* genome and identified genes under the QTL. We restricted our search to QTL with LOD > 9 (Table 2). All 9 of these QTL were on either chromosome 3 or 10. Because several of the QTL co-localized (had overlapping 1.5 LOD confidence intervals), we often found the same genes under multiple QTL. After removing duplicate entries, we found 490 unique genes underlying the 9 QTL investigated (S2 Table).

**Table 2:** Fishers exact tests for enrichment of FVT QTL for MR-identified genes.

		QTL		
		Yes	No	p-value
**MR10**	Yes	0	2	1.0
	No	5,816	37,645	(NS)
**MR20**	Yes	16	0	6.91e-13
	No	6,800	37,647	***
**MR30**	Yes	25	4	4.98e-09
	No	5,791	37,643	***
**MR50**	Yes	46	10	9.93e-21
	No	5,770	37,637	***

p> 0.05, NS; p<0.0001, ****

### RNAseq

We used RNA sequencing (RNAseq) to understand the transcriptomic mechanisms underlying FVT QTL and as an alternative approach for examining the genetic architecture of our FVT traits without *a priori* knowledge. 21,147 genes of 28,668 genes with detectable expression in UN treatment were differentially expressed among RILs (FDR < 0.01). The 10,000 genes with the most variable expression among RILs were used for downstream network analysis.

### Mutual Rank (MR) network analysis

**Fig 4:**
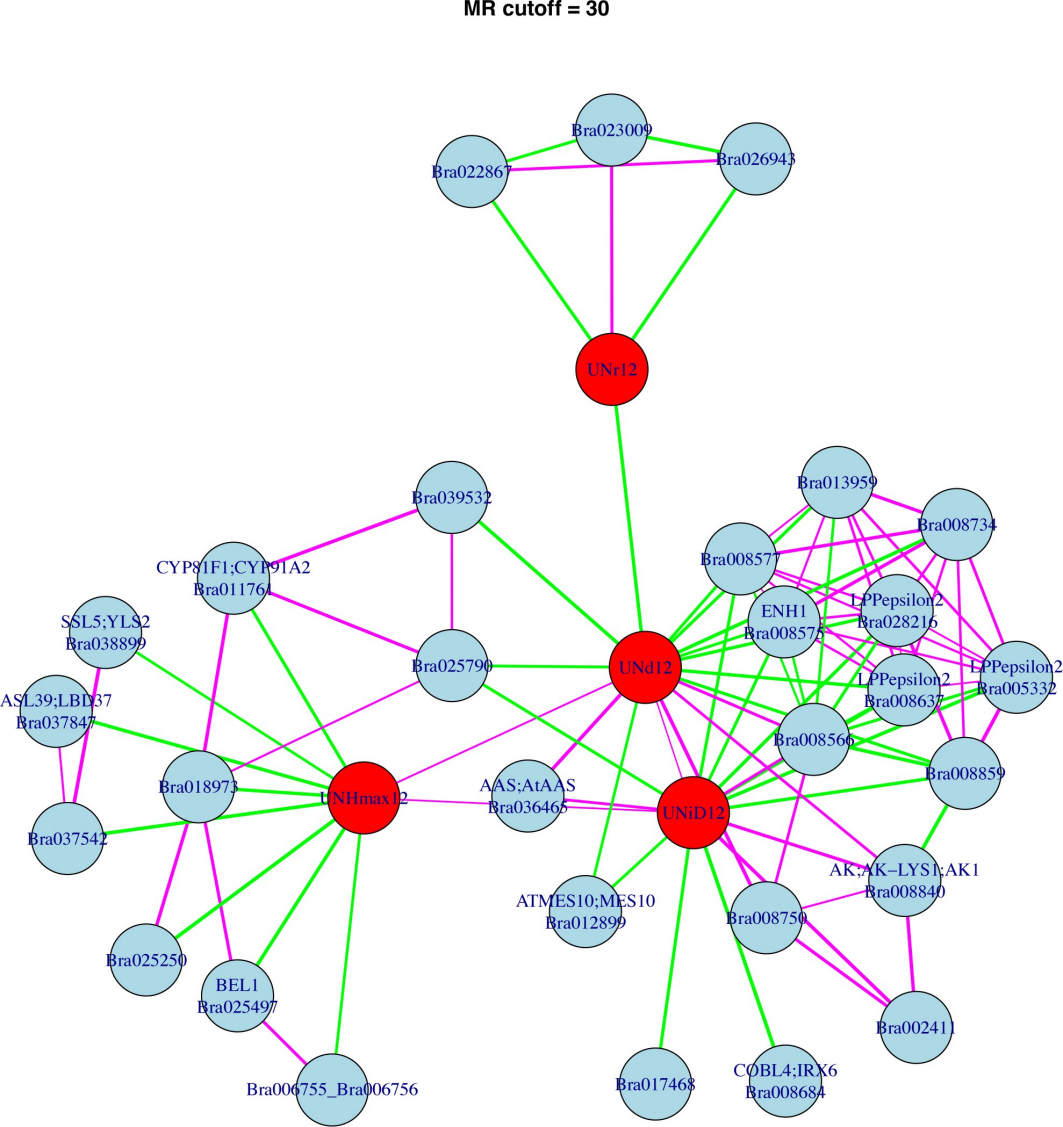
A scale-free diagram of the Mutual Rank network nucleated around FVT traits from 2012 with a cutoff of 30. Network nodes consist of either FVT traits or co-expressed genes. FVT traits are shown in red circles and genes are indicated in blue circles. Network edges indicate significant correlations. Purple lines indicate positive correlation values while green lines indicate negative correlation values and line thickness corresponds to strength of the correlation. UN, uncrowded; r, growth rate; d, duration of growth; iD, time in degree days when the growth curve reached its inflection point; Hmax, estimated maximum height based on FVT modeling. Additional network cutoffs, 2011, and 2012 crowded networks are in S4 Fig; gene names and annotations are in S3 Table.

MR Network Analysis is an alternative method that is independent of QTL analysis for identifying genes that may contribute to phenotypic variation. Genes identified as members of MR networks therefore likely contribute to and predict phenotypic variation. To find gene co-expression networks relevant to the FVT model parameters, we built MR networks nucleated on each FVT model parameter and performed permutation analyses to determine the statistical significance of our networks. Ninety-five or more of 100 permutations had zero connections between FVT parameters and gene expression. Therefore, our MR networks are enriched for *bona fide* connections at a variety of MR threshold cutoffs (The MR30 network is shown in Fig 4; larger networks become difficult to visualize and are presented in S4 Fig). Complete gene membership for all MR-thresholds annotated with the best hit obtained by blastn against the predicted *A*. *thaliana* proteome are presented in supplemental materials S3 Table.

We used Fisher’s exact test to determine whether FVT QTL were enriched for MR-identified genes. We found no evidence for enrichment for MR10 networks (p = 1.0) but significant evidence for enrichment for MR20, MR30, and MR50 networks (p<5E-09; Table 2). In theory, MR10 networks should contain only those genes whose expression values are most highly correlated with FVT phenotypes. The non-significant results for MR10 may be caused by low power due to the single gene identified.

To visualize the relationship between FVTs and genes in the MR networks, we made scatter plots of each FVT against each directly connected MR gene (S5 Fig). For a minority of these genes (21 of 71) the samples group into two clusters on the scatter plot, corresponding to RILs with low/high expression of the MR gene. This pattern suggests that expression of the MR gene and the FVT value are either controlled by the same gene or by two closely linked genes. However, the majority (50 of 71) of the FVT / MR gene pairs show a relatively continuous linear relationship. This pattern is unlikely to arise simply by linkage and suggests that expression of these MR genes is indeed biologically related to FVT values.

### Single Nucleotide Polymorphism (SNP) identification in for MR genes

To identify candidate SNPs that could alter protein function of MR gene products, we compared the sequence data from the RIL population for all MR50 genes and identified segregating SNPs that are predicted to cause an amino acid changes (see methods). We identified a total of 53 SNP variants across 24 MR50 genes (S4 Table). Most of these were mis-sense mutations predicted to have moderate effects on gene function (including three mis-sense mutations in Br025497, a *BEL1* homolog). However, one frame-shift mutation predicted to have a high impact on gene function was identified in unannotated gene Bra08635.

### Weighted Gene Co-expression Network Analysis (WGCNA)

**Fig 5:**
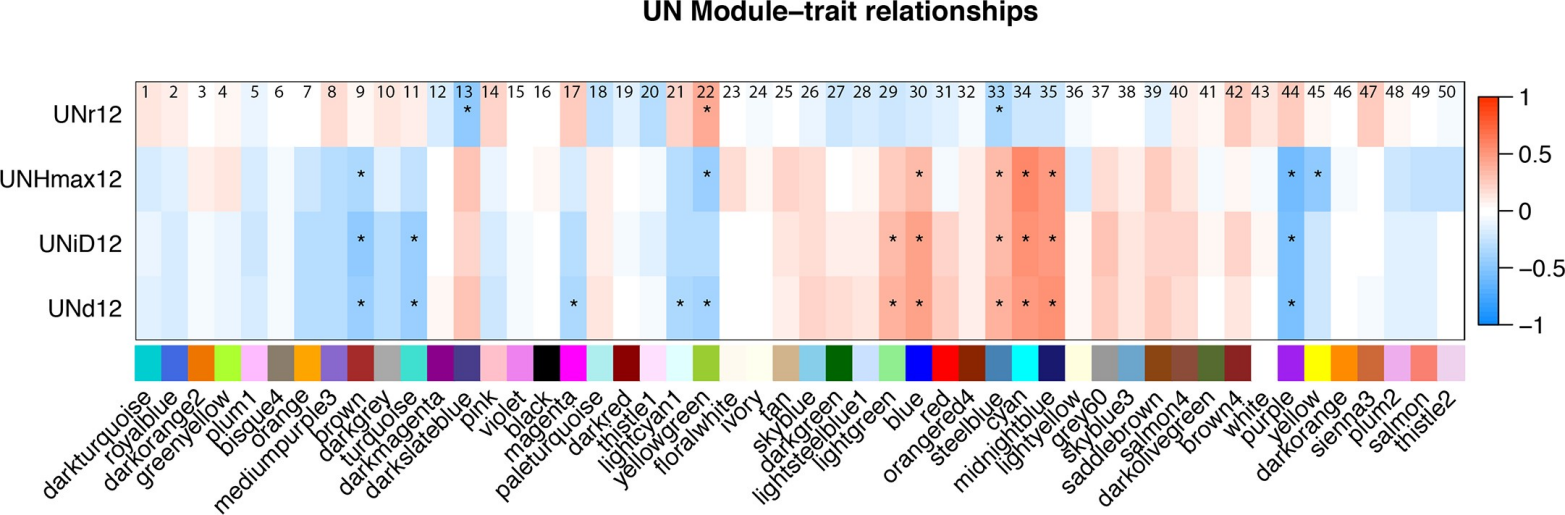
Correlations among WGCNA identified eigengenes and UN 2012 FVT traits. Significant correlations are denoted with an asterisk. r, growth rate; d, duration of growth; iD, time in degree days when the growth curve reached its inflection point; Hmax, estimated maximum height based on FVT modeling. Modules are named using a numerical system (above) and color scheme (below).

In a second, statistically independent approach to identifying gene expression networks related to estimates of FVT trait parameters, we used a Weighted Gene Co-expression Network Analysis (WGCNA) to identify 50 gene co-expression network modules consisting of a median of 90 genes each. We calculated eigengene values for gene expression within each module. Modules of interest were identified as those with a significant correlation between the eigengene expression values and FVT model parameters across the RILs (Fig 5). Gene Ontology (GO) enrichment analysis was performed to examine the potential function of correlated modules (S5 Table); below we discuss correlations with module eigengenes that had at least one GO term enriched. There are positive correlations between 2012 BLUPs for maximum height (Hmax), growth duration (d), and the time that the growth curve reached its inflection point (iD) and the “cyan (34)” module (related to protein translation), the “midnight blue (35)” module (related to wounding/herbivore defense responses as well as some abiotic stress responses), and the “blue (30)” module (enriched for genes related to cell division and development). This suggests that plants that have a longer duration of growth and reach a higher maximum height produce more protein, undergo more rounds of cell division, and have increased defense signaling. These three parameters also showed negative correlations with the “brown (9)” module (enriched for actin cytoskeleton and protein dephosphorylation terms). Hmax is negatively correlated with “yellow (45)” (enriched for terms related to photosynthesis). This correlation could be caused by a difference in cellular maturation rates: plants with more rapid cellular differentiation would be expected to show an upregulation of chloroplast genes and reduced growth due to earlier differentiation and consequently relative lack of cell elongation.

### Comparisons of QTL and network modeling for phenotypic prediction

To test the effectiveness of QTL, MR genes, and WGCNA in explaining the variation in FVT trait estimates, we compared a series of additive linear models based on QTL, MR genes, or WGCNA eigengenes both singly and in combination. For UNr (in 2012), models containing only QTL outperformed models containing either MR30 identified gene expression or WGCNA-identified eigengene expression (Table 3). For two-data type models, models with only QTL outperformed those containing multiple data types. For Hmax, MR gene expression outperformed both QTL and WGCNA-identified eigengene expression as well as combinations of two data types. For both traits, the full model (with all three data types for r, but which reduced to WGCNA and MR gene expression values for Hmax) were the best models for explaining phenotypic variation (r: F_(5,110)_ = 25.31, p<0.0001; Hmax: F_(9,106)_ = 33.16, p<0.0001). Similarly, the best two-data type models were a significantly better fit to the data than the best single-data type models (*r*: F_(5,114)_ = 40.182, p<0.0001; Hmax: F(4,113) = 80.398, p<0.0001). For all comparisons, the significantly better model according to ANOVA also had lower AIC scores (Table 3). Taken together, these results indicate that although each approach has significant predictive capacity, combining multiple approaches improves estimation of trait variation.

**Table 3:** Comparison of additive linear models using genetic and transcriptomic data to explain 2012 uncrowded phenotypic data.

Trait	Best single-data type model	AIC	Next best AIC (next best model)	Formula^§^	Best model F-value (DF), significance and adjusted R^2^
r	QTL	-1305.97	-1256.43 (WGCNA)	y ~ rQTL2 + rQTL2 +r QTL3	F(3, 113) = 30.9 *** R^2^ = 0.4361
Hmax	MR	735.5348	783.6546 (WGCNA)	y ~ Bra03899 + Bra011761 + Bra006755_Bra006756 + Bra036465 + Bra008859 + Bra037542	F(6,109) = 45.48 *** R^2^ = 0.6989
**Best 2-data type model**					
r	QTL + WGCNA (reduces to just QTL)	-1305.97	-1297.869 (MR+WGCNA)	y ~ rQTL1 + rQTL2 + rQTL3	F(3,113) = 30.9 *** R^2^ = 0.4361
Hmax	MR + WGCNA (reduces to just MR)	734.2895	752.3889 (QTL+MR; reduces to just MR*)	y ~ Bra011761 + Bra006755_Bra006756 + Bra13959 + Bra08840 + Bra008859 + Bra037542 _ Bra002411	F(7,108) = 40.16 *** R^2^ = 0.7045
**Best overall model**					
r	Full model (QTL+ MR+ WGCNA)	-1308.602	-1305.97 (QTL + WGCNA)	y ~ rQTL2 + yellowgreen(22) + Bra006755_Bra06756 + Bra025790 + Bra028216	F(5, 110) = 25.31 *** R^2^ = 0.5138
Hmax	Full model (reduces to MR + WGCNA)	731.63	-734.2895 (MR + WGCMA; reduces to just MR*)	y ~ yellow(45) + Bra011761 + Bra006755_Bra006756 + Bra008575 + Bra008577 + Bra008840 + Bra008859 + Bra037542 + Bra002411	F(9,106) = 33.16 *** R^2^ = 0.7157

*** p < 0.0001

* This model reduced to include just MR gene expression values but is different from the best Hmax single-data type model that also includes just MR gene expression values.

^§^ *r*QTL 1–3 have markers at A03x 6417941, A05x23393567, and A10x11427369, respectively

### eQTL analyses and colocalization of eQTL with FVT QTL

**Fig 6:**
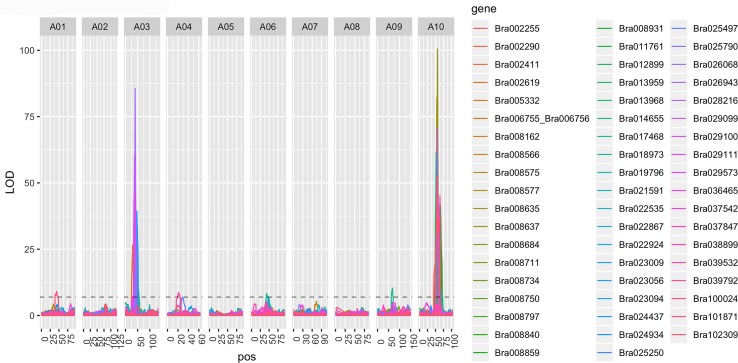
Expression trait QTL (eQTL) identified using Composite Interval Mapping (CIM) for MR50-identified genes where MR networks were nucleated around UN FVT traits. Note the eQTL hotspots on chromosomes 3 and 10.

Because including MR and WGCNA results both improved upon linear models for FVT traits that contained just QTL (Table 3) and because all models that included MR and WGCNA gene/eigengene expression values were significant and predicted FVT trait variation, we used eQTL analyses to assess the mechanistic relationship between MR gene/WGCNA eigengene expression and FVT QTL. For the 56 MR50-identified genes, 40 genes had a total of 41 significant eQTL, 22 of which were *cis*-eQTL (S6 Table). The 41 eQTL were distributed on chromosomes 1, 3, 4, 6, 9, and 10. In congruence with FVT QTL mapping results, there were eQTL with particularly high LOD scores on chromosomes 3 and 10 (LOD >75; Fig 6). There was significant overlap among 2012 FVT QTL confidence intervals and MR eQTL confidence intervals based on permutation tests (n = 1000, p = 0.003). One explanation for co-localization of FVT QTL and MR eQTL is pleiotropy; i.e. the same genetic change is causing changes in MR gene expression and in the FVT trait. An alternative interpretation is that causal loci are in linkage disequilibrium. These two interpretations are not mutually exclusive; it is likely that pleiotropy explains the association for some traits and linkage explains the association for others.

Of the 40 MR50 genes that had eQTL, a total of 37 genes had a total of 38 eQTL that overlapped with FVT QTL. Eighteen of the 37 MR50 genes with eQTL that colocalized with FVT QTL had *cis*-eQTL (Table 4). The co-occurrence of these loci as MR-identified *cis*-eQTL and FVT QTL identifies a list of strong candidate genes for regulating the FVT traits. One MR gene (Bra 012899) had multiple eQTL that overlapped with FVT QTL; both of these were on chromosomes 3 and 10 and both were *trans*-eQTL.

**Table 4:** MR-identified genes with *cis*-eQTL that co-localize with UN 2012 FVT QTL. Note that because multiple FVT QTL overlap, a single MR *cis*-eQTL may colocalize with FVT QTL for multiple traits. Genes with missense mutations (S4 Table) are indicated in bold.

MR gene	MR network	Chromo-some	FVT trait	eQTL LOD	AGI	*A*. *thaliana* symbol
**Bra022867**	20	3	iD	36.97	AT2G31810	NA
Bra023009	20	3	iD	25.05	AT2G43590	NA
**Bra023056**	50	3	iD	37.78	AT2G36230	*APG10;HISN3*
**Bra023094**	50	3	iD	39.40	AT2G37050	NA
**Bra029099**	50	3	R	59.98	AT5G53050	NA
Bra029100	50	3	R	28.52	AT5G53045	NA
**Bra008566**	20	10	r, Hmax	31.22	AT5G17270	NA
Bra008575	20	10	r, Hmax	67.28	AT5G17170	ENH1
Bra008577	20	10	r, Hmax	82.10	AT5G17150	NA
**Bra008635**	50	10	iD, r, d, Hmax	47.76	AT5G16210	NA
Bra008637	20	10	iD, r, d, Hmax	100.525	AT5G66450	*LPPepsilon2*
Bra008684	30	10	iD, r, Hmax	17.86	AT5G15630	*COBL4;IRX6*
**Bra008711**	50	10	r, Hmax	24.58	AT5G15250	*ATFTSH6;FTSH6*
Bra008734	30	10	r, Hmax	41.79	AT5G14860	NA
Bra008750	30	10	r, Hmax	15.37	AT5G14600	NA
**Bra008840**	20	10	r, Hmax	22.52	AT5G13280	*AK;AK-LYS1;AK1*
Bra008859	20	10	r, Hmax	41.59	AT5G13070	NA
Bra008931	50	10	Hmax	8.51	AT5G11880	NA

Next we performed eQTL analyses (S6 Fig) for the 11 WGCNA-identified eigengene modules that were significantly correlated with UN 2012 FVT (see Fig 5). Six of these 11 eigengenes had eQTL: Chromosome 3 harbored strong eigengene eQTL for “darkslateblue (13)”, “steelblue (33)”, and “yellowgreen (22)” (all with no go enrichment; nge). Chromosome 6 had eQTL for “midnightblue (35)” (herbivore/wounding). Chromosome 10 had eQTL in two locations, one for “brown (9)” (actin cytoskeleton) and “lightgreen (29)” (nge), the other for “midnightblue (35)” (herbivore/wounding). Four eigengenes had eQTL that colocalized with FVT QTL, indicating a potential causative connection between eigengenes and FVT for r, iD, and Hmax (Table 5). However, each eigengene had only one eQTL that colocalized with an FVT QTL.

**Table 5:** Eigengene eQTL and FVT QTL colocalization.

Trait (eigengene)	Chromo-some	FVT trait	LOD range
Brown (9)	10	r, Hmax	6.05–7.22
Darkslateblue (13)	3	r, iD	47.82–47.82
Midnightblue (35)	10	r, Hmax,	8.82–9.67
Yellowgreen (22)	3	Hmax	48.42–48.55

The second chromosome 10 location (“midnightblue (35)”) overlaps with the FVT QTL9 and the eigengene has significant correlations with d and iD FVTs indicating a possible causative connection. We then performed permutation tests and determined that FVT QTL were enriched for WGCNA eQTL (n = 1000, p = 0.002).

## Discussion

Plant height is often correlated with fitness and yield. Height is a complex and dynamic trait that changes over the course of development, and variation in plant height is necessarily generated through variation in developmental dynamics. However, similar heights can be achieved through multiple different growth curves. Quantifying the underlying genetic architecture and mechanistic basis of growth dynamics may result in improved estimations of final plant height, fitness, and yield. Here, we use Bayesian hierarchical modeling to estimate Function-Valued Trait (FVT) parameters describing continuous plant growth and explore their correlations with phenology and fitness. We test whether mapped QTL, genes identified through Mutual Rank (MR) co-expression, eigengenes identified through Weighted Gene Network Co-expression Analyses (WGCNA), or combinations of these information types best explain variation in agroecologically relevant FVT traits in the field. Further, we employ eQTL analyses to explore the regulatory mechanisms that connect FVT QTL with phenotypic variation.

Although development typically occurs in a continuous fashion, most studies quantifying development necessarily collect data at discrete timepoints. We take a “parameters as data” approach to FVT modeling to estimate the continuous nature of plant development [2,16]. Much as floral development or leaf development has well defined core molecular genetic pathways that govern organ formation, elaboration, or elongation [reviewed in 35], there is likely a core genetic architecture that contributes to plant height. However, exogenous and endogenous factors can influence the outputs of these developmental programs. For instance, crowding may trigger a shade avoidance response and lead to rapid increases in height [e.g. 36]. Similarly, plant carbon status can affect the developmental morphology and final size of organs such as leaves [17,18,21]. We took two approaches to examining the core developmental genetics of plant height. First, we grew plants across multiple growing seasons and in crowded and uncrowded conditions. Second, we included a genotype-specific co-factor in our FVT models that accounts for variation in photosynthetic rates (approximated through A_max_), thereby statistically factoring out variation due to carbon availability and allowing us to more directly interrogate the developmental genetic architecture and molecular mechanisms contributing to plant height [21,22]. In our study, all FVT traits had relatively high broad sense heritabilities (>70%), and all had significant main effects of genotype. Although there were no significant main effects of treatment (i.e. treatment means did not differ), all FVT trait estimates (except iD) exhibited genetic variation for assimilation-independent phenotypic plasticity via a genotype by environment (G×E) interaction, likely because of rank-order differences across treatments at the genotypic level (Table 1).

Morphological phenotypes, such as components of yield and height, can be highly integrated throughout development [reviewed in 37]. Final height is often used as a proxy for yield or fitness, yet plant growth dynamics throughout ontogeny may also be correlated with aspects of yield such as fruit and seed set [38,39]. In our experimental set of *Brassica rapa* Recombinant Inbred Lines (RILs), plant developmental dynamics including duration of growth (d), the inflection point in the growth curve that represents the change from exponentially accelerating to decelerating growth (iD), and estimates of final plant height (Hmax) were all significantly and positively genetically correlated (Fig 2). Interestingly, growth rates (r) were negatively correlated with d and iD, but were not correlated with Hmax, indicating that while there is a trade-off between growth rates and durations, duration of growth may be more important for final plant height than growth rate. All of our estimates of plant growth and final size were significantly genetically correlated with phenology and yield traits, demonstrating that the developmental dynamics of growth can be related to crop yields and plant fitness through mechanisms that are at least partially independent of final size. Because final size is positively correlated with yields while growth rates are negatively correlated with yields, selection for maximum yields at early harvest dates may come at the expense of late harvest yields and vice versa.

To examine the genetic architecture underlying the FVT estimates of growth rates, durations, and final sizes, we mapped QTL for FVT parameters. Of particular note, when QTL for r colocalized with d, the QTL were of opposite sign, confirming our negative genetic correlations between growth rates and durations, and indicating potentially pleiotropic loci contributing to both traits. On average, FVT QTL explained 24% of trait variation and the number of genes under each QTL ranged into the hundreds. To narrow down the list of candidate genes and understand the mechanistic regulation of FVT via QTL, we took two additional transcriptomic co-expression approaches to exploring regulation of FVT traits: First, we seeded a Mutual Rank (MR) co-expression network with FVT traits and asked which gene expression values correlated with variation in FVT traits. Second, we constructed 50 eigengenes based on a Weighted Gene Co-expression Network Analysis (WGCNA) and asked which eigengenes were correlated with individual FVT trait. We found that FVT QTL were significantly enriched for MR genes, indicating that these two approaches were identifying some common drivers of FVT traits. To compare the effectiveness of all three approaches, we asked whether QTL, MR genes, or eigengenes best explained variance in FVT traits. Although QTL outperformed both co-expression network modeling approaches for r, combining data from multiple approaches yielded improvements in our models, indicating that even though QTL, MR genes, and eigengenes often physically co-localize within the genome, they are not interchangeable with one another (Table 3).

To understand the potential function of genes related to growth WGCNA and MR networks, we examined gene annotations of homologous *Arabidopsis thaliana* genes. Although about half of the eigengenes that correlated with FVT BLUPs had no gene ontology enrichment, three eigengenes with eQTL on chromosome 9 were enriched for actin/cytoskeleton, herbivore/wounding and cell division. The MR30 genes include a homolog of the homeodomain gene *BEL1* [40]; *BEL1* homologs have been implicated in regulation of the shoot apical meristem [41] and thus could be related to plant growth. The *BEL1* homolog had three missense mutations predicted to have moderate impact on genet function. An additional gene was identified with homology to the COBRA family gene *COBL4/IRX6* (negatively correlated with iD), involved in secondary cell wall biosynthesis. The MR30 network also contains a number of genes involved in metabolic homeostasis. Four of these genes are localized to the plastid and negatively correlated with d and iD, including three orthologs of the *plastidic lipid phosphate phosphatase epsilon 2* gene (*LPPε2*), which is potentially involved in synthesis of diacylglycerol, a precursor to essential photosynthetic membrane components [42]. Another plastid-localized MR30 network gene is *ENHANCER OF SOS3-1 (ENH1)*; *ENH1* functions to mitigate the effects of reactive oxygen species [43]. Thus, plants with longer growing periods appear to put less resources into photosynthesis. The MR30 network also includes a homolog of the *A*. *thaliana LATERAL ORGAN BOUNDARY DOMAIN37 (LBD37)* gene, which is an important regulator of nitrogen response in both *A*. *thaliana* and *Oryza sativa* [44,45]. *LDB37* is negatively correlated with Hmax and had two moderate missense mutations. Two genes involved in amino acid synthesis or homeostasis are present in the MR30 network and show positive correlations with d and iD: first, a homolog of *ASPARTATE KINASE1 (AK1)*, which is required for regulation of aspartate, lysine, and methionine was recovered [46]. The *B*. *rapa AK1* homolog had a single moderate mis-sense mutation. Second, *AROMATIC ALDEHYDE SYNTHASE (AAS)*, which converts phenylalanine into phenylacetaldehyde [47] was also present. Overall the MR30 network results point to a close connection between metabolic regulation and growth.

Transcriptomic data allowed us to further explore the regulatory control of the FVT using eQTL mapping of WGCNA eigengenes and MR genes. eQTL mapping treats gene expression levels as quantitative traits. When combined with QTL studies of morphological phenotypes, the ultimate goal of eQTL mapping is to identify the molecular genetic changes in gene expression that lead to structural phenotypic variation, thus providing mechanistic explanations for the associations between genotype and phenotype [48]. In humans, such studies demonstrate that eQTL can be used in a cell-type specific fashion to annotate GWAS associations [49]. Based on the 56 MR50 genes in our study, we identified 41 significant eQTL, 40 of which colocalized with FVT QTL. Six of the 11 WGCNA eigengenes that correlated with FVT also had eQTL, and four of these eQTL colocalized with FVT QTL. These data demonstrate that the relationship between genomic loci (FVT QTL) and phenotypic variation in FVT traits is likely mediated by gene expression, specifically the expression of the genes and eigengenes we identified via MR and WGCNA.

Our eQTL results qualitatively departed from common morphological trait QTL analyses in two ways. First, MR-identified gene expression traits mapped to all chromosomes except chromosome 2, but two locations had multiple eQTL with very high LOD scores (>75): the top of chromosome 3 and the middle of chromosome 10. Virtually all genes had eQTL that mapped to one of these two locations, a common result potentially indicating an eQTL ‘hotspot’ [50]. A previous study of the effects of soil phosphorous using the same *B*. *rapa* RILs also identified eQTL hotspots [30], but on different chromosomes. The colocalization of eQTL hotspots and FVT QTL may indicate novel regions involved in pleiotropic co-regulation of several downstream genes in the regulatory network contributing to change in plant height [29].

Although the presence of eQTL hotspots indicates pleiotropic gene regulation, our eQTL analyses also qualitatively departed from the FVT QTL analysis in that most of the gene expression traits we mapped were not polygenic. Of the 56 MR gene expression traits mapped, only 1 had multiple eQTL that colocalized with FVT QTL. eQTL studies commonly find a relative paucity of polygenic regulation compared to structural QTL studies, and our results support the general consensus that expression traits and structural phenotypes have distinctly different genetic architectures [but see 32 for a counter-example]. However, most eQTL are of relatively large effect, meaning that many small effect eQTL could remain undetected and contribute to polygenic regulation of gene expression traits [29], and these eQTL may or may not occur in regulatory hotspots.

To further understand the regulation of expression traits and FVT QTL, we divided MR eQTL into two classes: putative *cis-* and *trans*-eQTL where *cis*-eQTL likely correspond to *cis*-regulatory elements influencing gene expression [51]. In contrast, *trans*-eQTL do not contain the gene whose expression pattern is mapped and likely correspond to *trans*-acting factors such as transcription factors that influence the MR gene expression [52]. In our study, 53% of all eQTL identified were *cis*-eQTL. Of the 40 MR genes with eQTL that colocalized with FVT QTL, 18 were *cis* (45%) and the remaining 22 were in *trans*, which is a much higher than the proportion of *cis-*eQTL than identified in an intraspecific maize cross [53]. Because our *B*. *rapa* RILs are also generated from an intraspecific cross, theoretical and experimental work suggesting that *trans* gene regulation should be more prevalent than *cis* regulation at the intraspecific level [54,55, but see 56 for an exception]. Our targeted eQTL mapping conducted in an agroecologically relevant field setting deviates from these expectations, indicating that our network construction may act as an strong filter for biologically relevant candidate genes with *cis*-eQTL.

**Fig 7:**
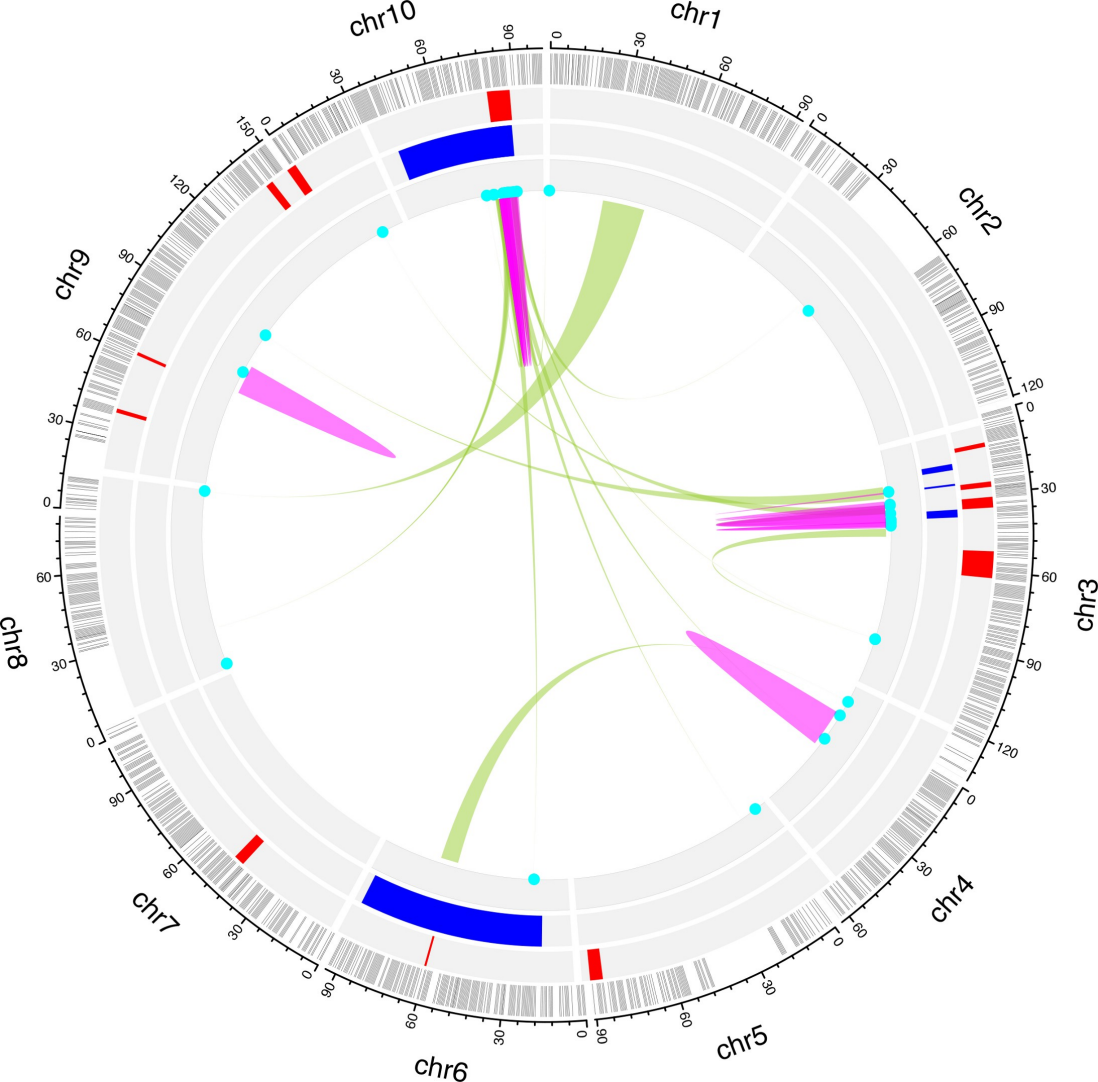
Integrating data from multiple biological levels and analyses reveals the mechanistic regulatory connections between genomic architecture and *Brassica rapa* developmental phenotypes. Function-Valued Trait QTL (2012 uncrowded data, Fig 3), Weighted Gene Co-expression Network Analysis (WGCNA) identified eigengene eQTL (S4 Table), and genes identified via Mutual Rank (MR 30) co-expression (S3 Table) occur at regulatory hotspots on chromosomes 10 and 3, indicating that these MR genes are candidate master regulators that integrate information to generate developmental trait variation. MR gene *cis*-eQTL (pink links) on chr10 and 3 lend further credence to this relationship. MR genes with *trans*-eQTL (green links) that map to these hotspots are putative upstream genes feeding in to the FVT regulatory network (Fig 6). From exterior to center: chromosomes in black, linkage map in grey, FVT QTL in red, eigengene eQTL in blue, MR genes in cyan, MR *trans*-eQTL in light green and MR *cis*-eQTL in pink.

Our study demonstrates the importance of examining not just final plant height, but the developmental dynamics that contribute to height growth curves in agroecologically relevant field settings. We fit function-valued trait models to our data and, while statistically factoring out aspects of physiology such as carbon assimilation rates, demonstrate that parameters describing continuous developmental growth curves are correlated with plant fitness and yield. There is genetic variation for plasticity of growth rates and final sizes, but not the inflection point (transition from accelerating to decelerating growth) of growth curves. Changes in the sign of bivariate correlations indicate a trade-off between yields at given final size vs. yields at early developmental times. We map FVT QTL to multiple chromosomes and utilize a guided eQTL mapping approach to investigate the regulatory mechanisms connecting genotype to FVT phenotype. Specifically, we use WGCNA to identify eigengenes for actin/cytoskeleton and cell division processes whose expression values that correlate with FVT traits. FVT trait seeded MR co-expression networks had an overall association with metabolic regulation and growth processes. We demonstrate that combining multiple approaches yields the best explanation of phenotypic variance. We identify more *cis*-eQTL than expected, and these eQTL are highly colocalized at regulatory hotspots, likely including transcription factors that influence downstream gene regulation. Because our *cis*- and trans-eQTL hotspots colocalize with FVT QTL, these expression traits are likely components of the molecular regulatory mechanisms mediating the generation of FVT phenotypic variation from genomic variation (Fig 7).

## Materials and methods

### Species description

*Brassica rapa* (Brasssicaceae) is an herbaceous crop species first domesticated in Eurasia. This study was conducted on Recombinant Inbred Lines (RILs) derived from crossing R500, a yellow sarson oil seed variety, with IMB211, which is a rapid cycling line derived from the Wisconsin Fast Plant line (WFP). All RILs are expected to be >99% homozygous [57–60]. In comparison with IMB211, R500 flowers later, attains a larger size and greater biomass, and allocates more resources to seed production. This experiment includes 120 RILs as well as R500 and representative IMB211 genotypes.

### Experimental design and data collection

In 2011, and 2012, the IMB211 × R500 RILs were germinated in the University of Wyoming greenhouse in fertilized field soil, and transplanted into the field at two planting densities, as previously described [1]. Briefly, crowded (CR) plants consisted of 5 plants of the same genotype per 4” peat pot with the central plant designated as a focal individual. The uncrowded (UN) treatment consisted of a single plant per pot. When the cotyledons were expanded, plants were transplanted to the field into randomly located blocks that consisted of either UN or CR plants. Each block contained a full RIL set (and representatives of the RIL parental genotypes), and RIL locations were randomized within blocks with 25cm between each focal plant. For phenotypic data collection 6 UN blocks were transplanted into the field in 2011 and in 2012 8 CR and 8 UN blocks were transplanted. In 2011, an additional 5 UN blocks were transplanted into the field for RNAseq. Plants were watered daily to field capacity and treated with pesticides as needed following Baker *et al*. [1]. Each year, we collected data on the timing of germination, bolting, and flowering by surveying plants 5–7×/week. We recorded temperature data every 5s in the greenhouse and field using a series of Onset Hobo data loggers (Bourne, MA, USA) and a Campbell Scientific (Logan, UT, USA) CR23X data logger equipped with a Vaisala (Helsinki, Finland) HMP-50 sensor. Temperature data were used to produce hourly and daily means, as well as hourly and daily minimums and maximums, for Degree Day (DD) calculations, which used a *B*. *rapa*-specific base value of 0.96°C [61].

### Morphological data

Plant height was recorded for all plants starting at leaf emergence. In 2011, height was measured 6 times during the growing season, and these measurements captured final heights. In 2012, height was measured 2–3 times per week until senescence. Perhaps because of the increased frequency of data collection for 2012 FVT trait estimates, our RNAseq data corresponds more closely to 2012 plant-level phenotypic data compared to 2011, and we focus our analyses on 2012 plant-level phenotypic data. We present 2012 results for all FVT data; full results of FVT traits and FVT QTL including 2011 data can be found in supplemental materials. Flowering phenology and performance were estimated based on 2012 fruit and seed numbers, as described in Baker *et al*. [1].

### Function-Valued Trait (FVT) modeling and data analysis

Height data were visually inspected for erroneous data points on a replicate level following Baker et al [1]. FVT modeling for trait estimation used Bayesian approaches that fit logistic growth curves to longitudinal height data [Eq 1; adapted from 21]. Height for each individual replicate plant is represented by a minimum of 5 and maximum of 13 sequential measurements. Briefly, we utilized a three-level hierarchical Bayesian model that retains the measurement data structure to account for information across all plants and genetic lines within the population, including replicate plants within each line.

$$\frac{d}{dt}H\;=\;rH\left(\frac{H_{Hmax}-H}{H_{Hmax}}\right)$$ 1

Replicate-level parameters were extracted from the fitted logistic growth curves and treated as trait data [13,14,21,62,63]. These parameters include the growth rate (r, cm/DD), and an estimate of the maximum height based on the asymptote of the logistic growth curve (Hmax, in cm). Additional parameters were algebraically extracted from the growth curve and include the duration of growth (d, in DD) and the *i*nflection point of the growth curve in Degree Days (iD, in DD). The parameter d was defined as the time in DD when 95% of the final size (Hmax) was achieved. The parameter iD reflects the transition from exponentially accelerating to decelerating growth rates.

The hierarchical Bayesian model was implemented using PyMC, a Bayesian Statistical Modeling Python module. The model parameters were estimated via MCMC using the Metropolis-Hastings algorithm [64,65]. The MCMC estimations were performed using a single chain to sample 500,000 iterations, which includes the first discarded 440,000 burn-in iterations; the remaining 60,000 iterations were retained. By thinning to 1 iteration in 20, the retained iterations were reduced to 3,000 samples for every FVT parameter from which the posterior distributions were tabulated. All parameters’ trace and auto-correlation plots were examined to ensure that the MCMC chain had adequate mixing and had reached convergence. All observed data for each genotype were plotted with two 95% credible interval envelopes. The inner, yellow envelope represents the credible intervals for the model based on the observed data, and the green envelope (Fig 1., S1 Fig) is the 95% credible interval where future observations from the same environment are expected [22,66].

### Phenotypic plasticity

To detect environmental factors that might affect the correspondence between genotype and phenotype, we analyzed replicate level phenotypic datasets from 2012. We tested for the main effects of genotype and treatment and all possible interactions using the lme4 and pbkrtest packages in the R statistical environment [67–69]. In these tests, all effects were considered random and block was nested within the treatment effect. Significant main effects of environment (treatment) were considered evidence of phenotypic plasticity, and interactions of treatment × genotype was considered evidence for genetic variation in phenotypic plasticity.

### Best Linear Unbiased Predictions (BLUPs)

BLUPs were calculated independently for UN and CR treatments in R using the lmer function in the lme4 package while controlling for block effects [69,70]. Broad sense heritability (H^2^) was calculated as the genotypic variance divided by the sum of genotypic, block, and residual variances.

### Genetic correlations

We assessed the genetic correlations among height FVT and previously published phenology and fitness traits [1] across both environments and years using Pearson’s correlations of trait BLUPs. Bonferroni corrections for multiple testing were applied to all genetic correlations.

### QTL mapping

QTL analyses were performed in R/qtl [71] based on a map with 1451 SNPs having an average distance of 0.7 cM between informative markers [58]. The scanone function was used to perform interval mapping (1cM resolution with estimated genotyping errors of 0.001 using Haley Knott regression) to identify additive QTL [72]. All significance thresholds (0.95) were obtained using 10,000 scanone permutations [71,72]. Significant QTL identified via scanone were used to seed a search of QTL model space using an iterative process (fitqtl, refineqtl, and addqtl functions using 1000 imputations at 1cM resolution with estimated genotyping errors of 0.001) to identify additional QTL while taking into account the effects of QTL identified by scanone and addqtl. After each iteration, non-significant QTL were dropped and significant QTL were added to the model. QTL and their 1.5LOD confidence intervals are displayed using MapChart2.0 [73]. Percent variance explained (PVE) is calculated as PVE = 100 × (1–10^(-2 LOD/ n)). We compared QTL peaks to the *B*. *rapa* genome [Version 1.5;, 74] to identify positional candidate genes underlying each QTL. A similar approach was used for mapping eigengene QTL (see below). However, the R/qtl implementation of composite interval mapping [72] was used.

### RNAseq

We used the RNA sequencing data previously reported in Markelz et al [58]. Briefly, in 2011 five UN blocks of plants designated for destructive sampling were transplanted into the field and allowed to establish for three weeks. Apical meristem tissue, consisting of the upper 1cm of the bolting inflorescence, was collected from three individual replicate plants per RIL and immediately flash frozen on liquid nitrogen as described in Markelz et al [58]. RNA library preparation and sequencing were performed as previously described [58,75]. Reads were mapped to the *B*. *rapa* CDS reference described in [76] using BWA [77], with an average of 6.52 Million mapped reads per replicate. Read counts were imported to R [67] and filtered to retain genes where more than 2 counts per million were observed in at least 44 RILs. Libraries were normalized using the trimmed mean of M-values (TMM) method [78] and a variance stabilizing transformation was done using voom [79].

### Genetic network reconstruction

To reconstruct gene co-expression networks, the fitted gene expression values for each RIL from the limma-voom fit (expression ~ RIL) were used and filtered to keep the top 10,000 genes most variable between RILs.

For each sample type, two network reconstruction methods were used. First, mutual correlation rank (MR) networks [80] were constructed. Pairwise MRs were calculated between each of the 10,000 genes and also between each gene and the BLUP parameter estimates from the 2011 and 2012 FVT models. A series of increasingly large growth-related networks were defined using genes directly connected to the FVT parameters with MR thresholds of ≤ 10, 20, 30, and 50. Multiple different phenotypes were used to jointly seed each network, therefore networks may contain more nodes (and more genes) than the thresholds suggest. However, because some gene expression levels are uniquely correlated with specific phenotypes while others may be correlated with multiple phenotypes, the number of nodes is less than the product of the threshold value and number of phenotypes used to seed the network. Permutation analysis was used to test the network size expected by random chance at each threshold; 95 or more of 100 permutation networks had zero edges connecting FVT BLUPs and gene expression, showing that our MR networks are recovering statistically significant connections. We used the blastn algorithm [81] with the discontiguous megablast option and an E-value cutoff of 0.001 to compare *B*. *rapa* genes to *A*.*thaliana* genes (TAIR10 annotation; ftp://ftp.arabidopsis.org/home/tair/Sequences/blast_datasets/TAIR10_blastsets/TAIR10_cds_20101214_updated).

Second, we constructed networks using a Weighted Gene Correlation Network Analysis [WGCNA; 82,83]. For these networks a soft threshold power of 3 was used, corresponding to the lowest power that had a correlation coefficient > 0.9 with a scale-free network topology. We used the “signed hybrid” network, which only connects genes with positive correlation coefficients. This network consisted of 50 modules with a median of 91 genes per module. The eigengene expression value of each module was determined using WGCNA functions. The Pearson correlation between each module’s eigengene expression value and each FVT BLUP was calculated to identify modules potentially related to FVTs. Modules were considered significantly associated with a FVT BLUP if the multiple-testing corrected p-value (method = “holm” in R function p.adjust) for the correlation test was less than 0.05. Gene Ontology (GO) category enrichment was performed on each significant module; we only examined the Biological Process (BP) and Cellular Compartment (CC) categories. Categories were considered significantly enriched if the false discovery rate adjusted p-value was < 0.05.

### Single Nucleotide Polymorphism (SNP) identification in MR genes

To identify SNPs in Mutual Rank genes that could alter protein function, we compared the RNA sequence data from the RIL population for all MR50 genes to find segregating SNPs predicted to alter the amino acid sequence of the gene product. To do this, we used samtools v1. [84] to subset 434 BAM files from the individual RIL RNAseq replicates to retain reads overlapping the coding sequence of each MR gene. We then used GNU parallel [85] to FreeBayes v1.1.0-46-g8d2b3a0 [86] to identify SNPs segregating in the population. Each SNP was annotated with SnpEff v4.3t [87] and the resulting vcf file was imported into R [88] for filtering. SNPs that had a minimum depth of 400 (~ 1 read per bam file), that were predicted to be segregating in the population, and that were predicted to cause a change in the amino acid sequence of the gene product were retained; the minimum quality score (QUAL) of the retained SNPs was 330. Each resulting variant was manually evaluated in IGV [89] and strong candidates were retained.

### Comparing approaches for genetic architecture

We compared the effectiveness of QTL, MR, and WGCNA approaches for predicting phenotypic variation in r and Hmax through a series of multivariate linear regression models (lm function in R). We extracted the effect size and direction for each QTL using the effectplot function in r/qtl [72]. In all cases, the trait BLUPs were the dependent variable, and all allele-specific effect sizes, gene expression, and eigengene expression values were independent variables. For each trait we generated three types of additive models: 1) models with one type of independent variable (genotypic information based on alleles harbored at each QTL including allele-specific effect sizes and direction or genotype specific gene expression values for MR genes or genotype specific eigengene expression values), 2) models with two types of independent variables (QTL and MR gene expression, QTL and eigengene expression, or MR gene expression and eigengene expression), and 3) full models with all three data types as independent variables. For each trait we included only significant QTL, genes from the MR30 network, and eigengenes that were significantly correlated with the trait of interest. Each model was subjected to a backwards model reduction routine where non-significant terms were iteratively removed until all terms in the model had significant effects on the dependent variable (p<0.10). We used AIC scores to compare final models.

### Relationships between co-expression and FVT QTL

We performed Fisher’s exact test to determine whether the FVT QTL regions were enriched for genes and/or eigengenes identified via MR and WGCNA network analyses. Enrichment of FVT QTL for MR-identified genes was interpreted as evidence that the MR-identified genes are candidate causal genes for the FVT trait of interest.

### eQTL analyses

To explore the regulatory mechanisms of MR-identified genes and WGCNA-identified eigengenes, as well as their potential connection to FVT QTL, we performed eQTL analyses. Our network analyses effectively allowed us to reduce the number of expression traits mapped from 10,000 to less than 75. Therefore, we used Composite Interval Mapping [90], which is usually considered too computationally intensive for eQTL studies. CIM typically has narrower confidence intervals and should result in fewer spurious overlaps among potentially correlated expression traits. We used permutation testing [91] to establish a genome and experiment wide significance threshold for each gene or eigengene. For each of 1,000 permutations we recorded the highest LOD score observed for eQTL regulating MR genes or eigengenes; the 95^th^ percentile of these LOD scores was then used as the p < 0.05% significance threshold for declaring an eQTL significant.

### Overlap between eQTL and FVT QTL

The bayesint function in r/qtl was used to define 99% confidence intervals for each eQTL. For some eQTL with very high LOD scores the resulting confidence interval was a single base pair (clearly unrealistic given the limitations imposed by the number of recombination events in a mapping population). For such eQTL we used a window of +/- 2.5cM around the identified base pair as the eQTL interval. The resulting intervals were then examined for overlap with FVT QTL intervals.

### *cis* and *trans*-eQTL

We defined *cis*-eQTL as eQTL that include the physical gene generating the mRNA transcript and *trans*-eQTL as any eQTL that does not include the physical location of the gene. For MR-identified genes, *cis*-eQTL are interpreted as evidence of variation in *cis* regulatory elements such as promoters whereas *trans*-eQTL are interpreted as evidence for *trans*-acting regulatory proteins such as transcription factors, other signaling proteins, or small RNAs that modulate gene expression. Because eigengenes represent the composite expression of a median of 90 genes, one cannot assign *cis-* vs. *trans*-eQTL identity for these traits (although the majority of their action is expected to be in trans). MR gene or eigengene eQTL that colocalize with FVT QTL may explain the underlying basis for the FVT QTL, and such colocalizing eQTL represent candidate causal genes for the FVT eQTL locus. An alternative explanation is that eQTL that co-localize with FVT QTL are in linkage disequilibrium with the FVT QTL candidate gene. eQTL that do not co-localize with FVT QTL may still be affecting plant development, but at a level not directly detectable in the FVT QTL mapping.

## Supporting information

S1 Fig Figures. FVT model fits.
(PDF)

S2 Fig Conceptual overview of analyses. (PNG)

S3 Fig Genetic correlations among traits. (PDF)

S4 Fig Mutual Rank (MR) networks. (PDF)

S5 Fig Relationships between FVT and MR gene expression. (PDF)

S6 Fig eQTL for WGCNA eigengenes. (DOCX)

S1 Table QTLs for FVT traits. (CSV)

S2 Table Genes under FVT QTLs. (CSV)

S3 Table Names and annotations of MR genes. (CSV)

S4 Table Predicted amino-acid changing SNPs in MR genes. (CSV)

S5 Table GO analysis for WGCNA eigengene modules. (CSV)

S6 Table MR eQTL results. (CSV)

S7 Table FVT BLUPs. (CSV)
