# Correction: Integrating transcriptomic network reconstruction and eQTL analyses reveals mechanistic connections between genomic architecture and *Brassica rapa* development

**DOI:** 10.1371/journal.pgen.1009131

**Published:** 2020-10-08

**Authors:** Robert L. Baker, Wen Fung Leong, Marcus T. Brock, Matthew J. Rubin, R. J. Cody Markelz, Stephen Welch, Julin N. Maloof, Cynthia Weinig

In the Data Availability Statement, the following information is missing: Code for building networks from *B*. *rapa* RIL RNAseq data can be found at https://github.com/MaloofLab/Baker-2019-PLosG-Brapa-neworks.
