# Supplementary material for: Integrating transcriptomic network reconstruction and eQTL analyses reveals mechanistic connections between genomic architecture and Brassica rapa development
Source: PLoS Genet. 2019 Sep 12;15(9):e1008367. doi: 10.1371/journal.pgen.1008367 (PMC6759183; doi:10.1371/journal.pgen.1008367)
Supplement: S1 Fig — FVT model fits. (PDF) [file pgen.1008367.s001.pdf]

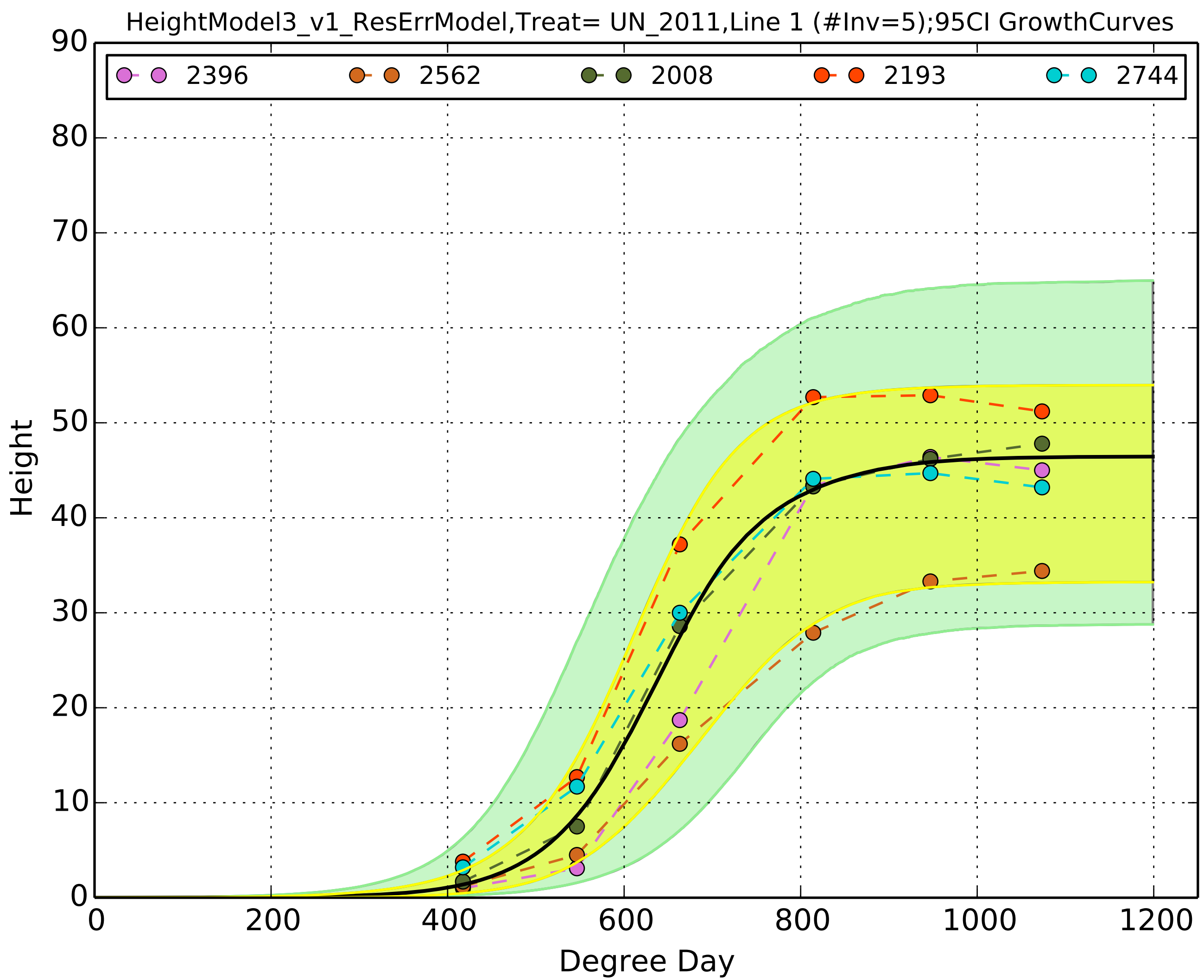

HeightModel3_v1_ResErrModel,Treat= UN_2011,Line 1 (#Inv=5);95CI GrowthCurves
2396
2562
2008
2193
2744
Height
Degree Day
0
10
20
30
40
50
60
70
80
90
0
200
400
600
800
1000
1200

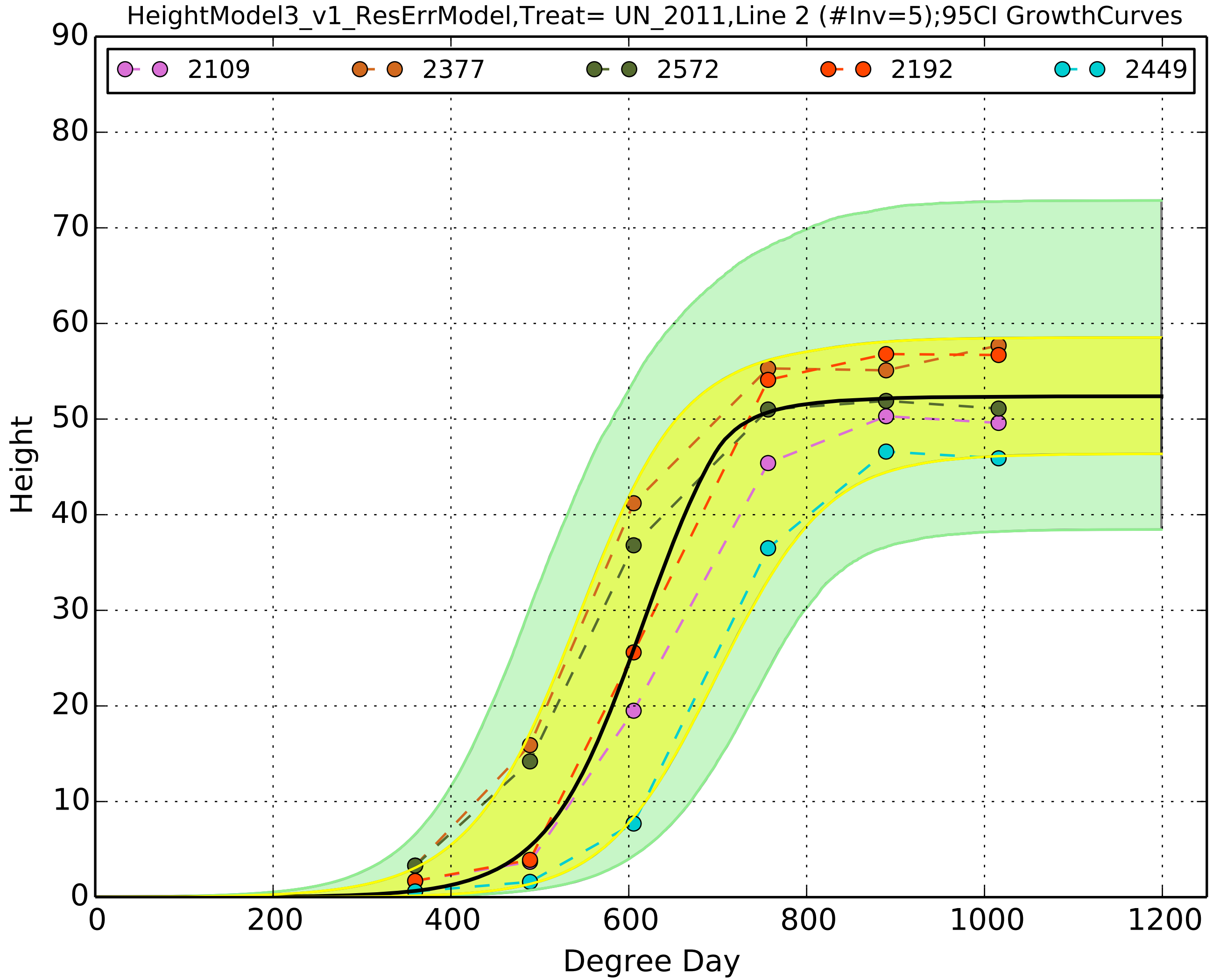
HeightModel3_v1_ResErrModel,Treat= UN_2011,Line 2 (#Inv=5);95CI GrowthCurves
2109
2377
2572
2192
2449
Height
Degree Day
0
10
20
30
40
50
60
70
80
90
0
200
400
600
800
1000
1200

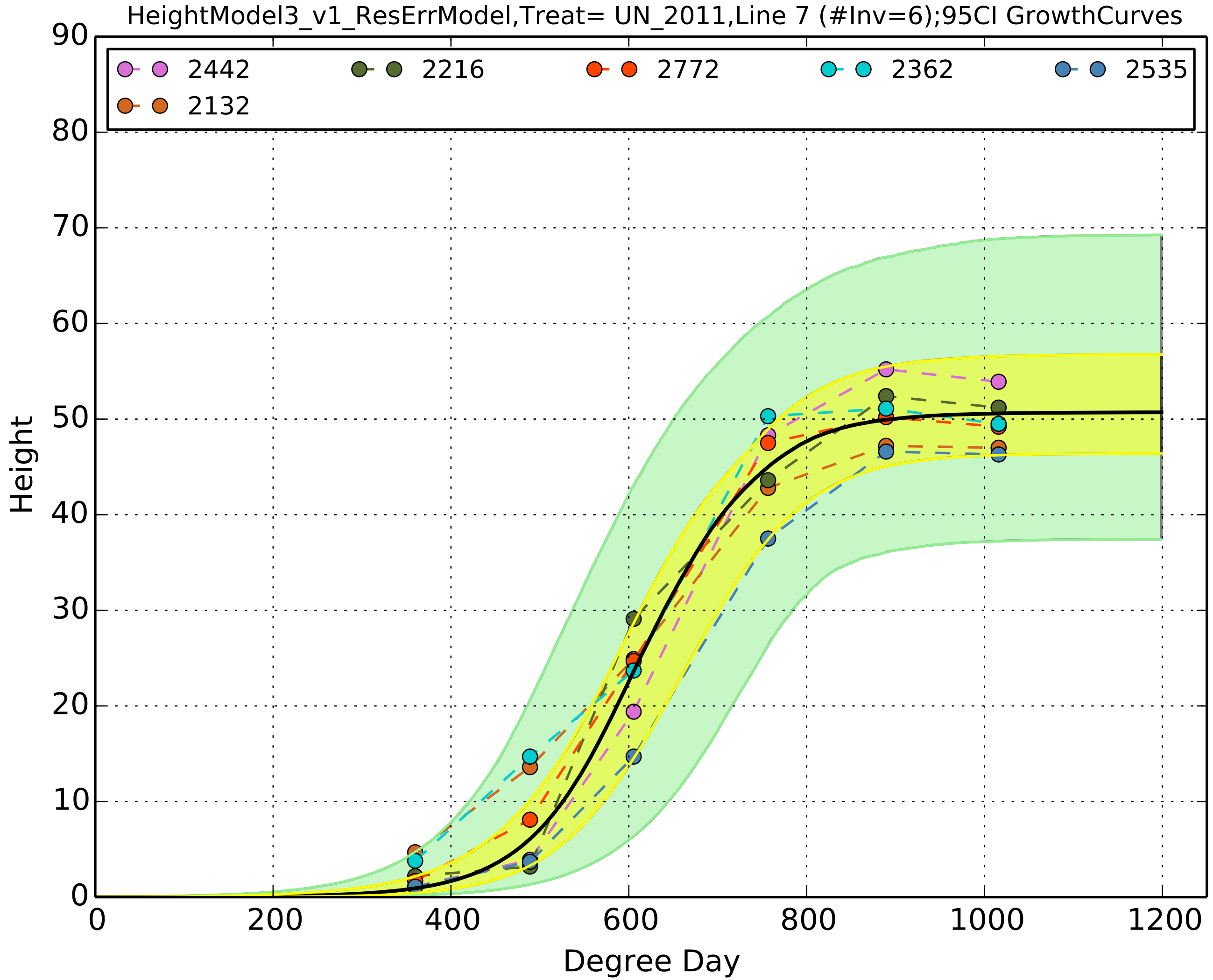
HeightModel3_v1_ResErrModel,Treat= UN_2011,Line 7 (#Inv=6);95CI GrowthCurves
2442
2216
2772
2362
2535
2132
Height
Degree Day
0
10
20
30
40
50
60
70
80
90
0
200
400
600
800
1000
1200

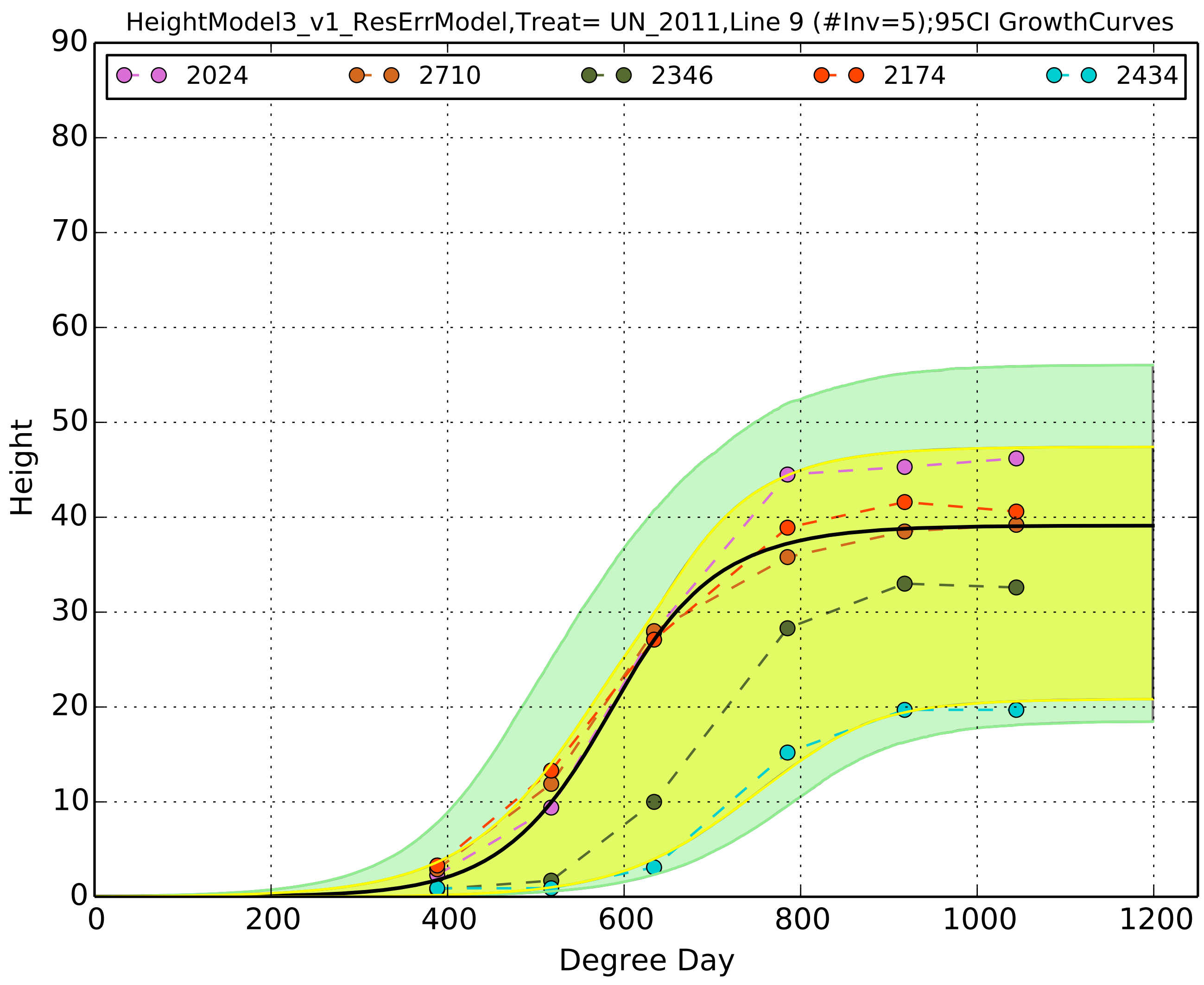

HeightModel3_v1_ResErrModel,Treat= UN_2011,Line 9 (#Inv=5);95CI GrowthCurves
2024
2710
2346
2174
2434
Height
Degree Day
0
10
20
30
40
50
60
70
80
90
0
200
400
600
800
1000
1200

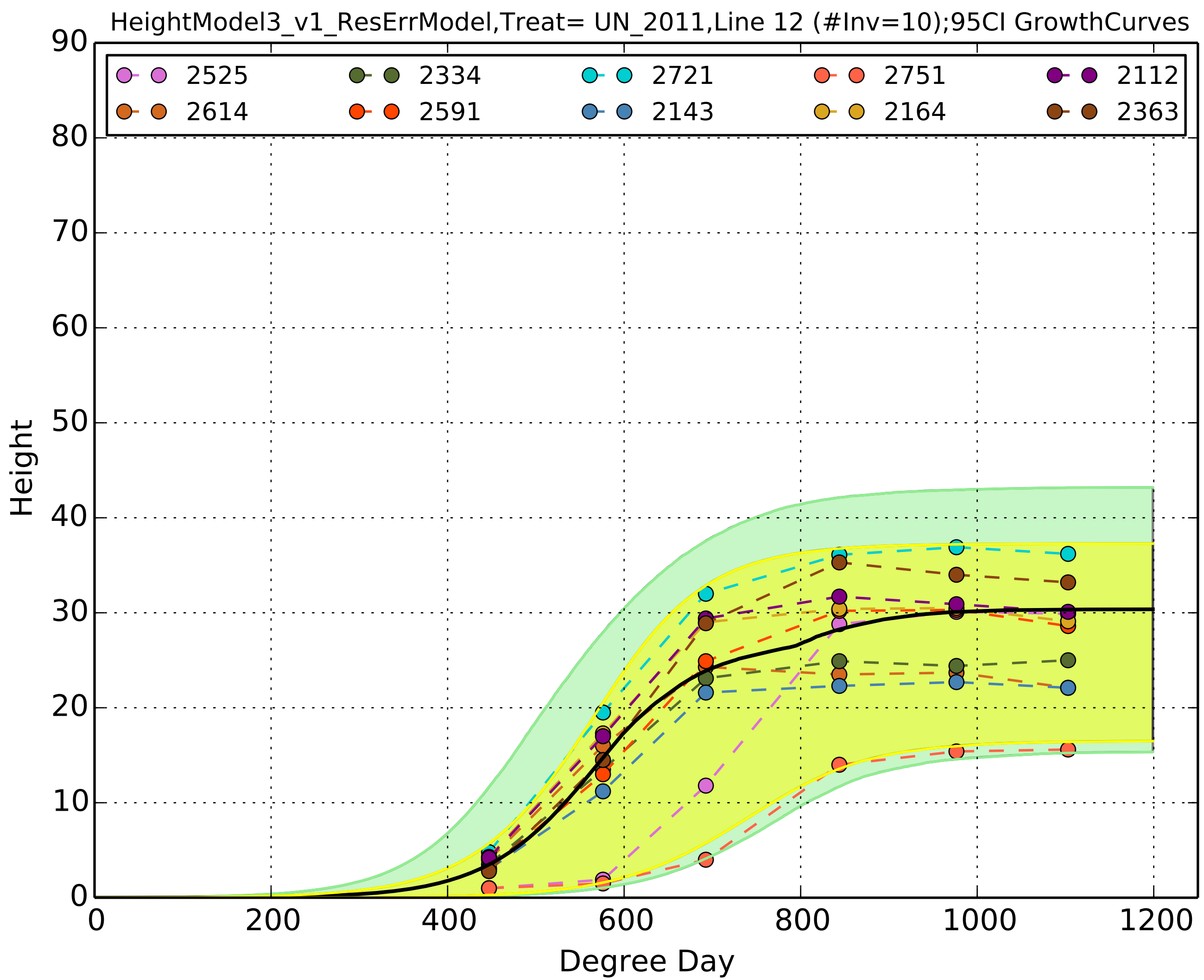

HeightModel3_v1_ResErrModel,Treat= UN_2011,Line 12 (#Inv=10);95CI GrowthCurves
2525
2334
2721
2751
2112
2614
2591
2143
2164
2363
Height
Degree Day
0
10
20
30
40
50
60
70
80
90
0
200
400
600
800
1000
1200

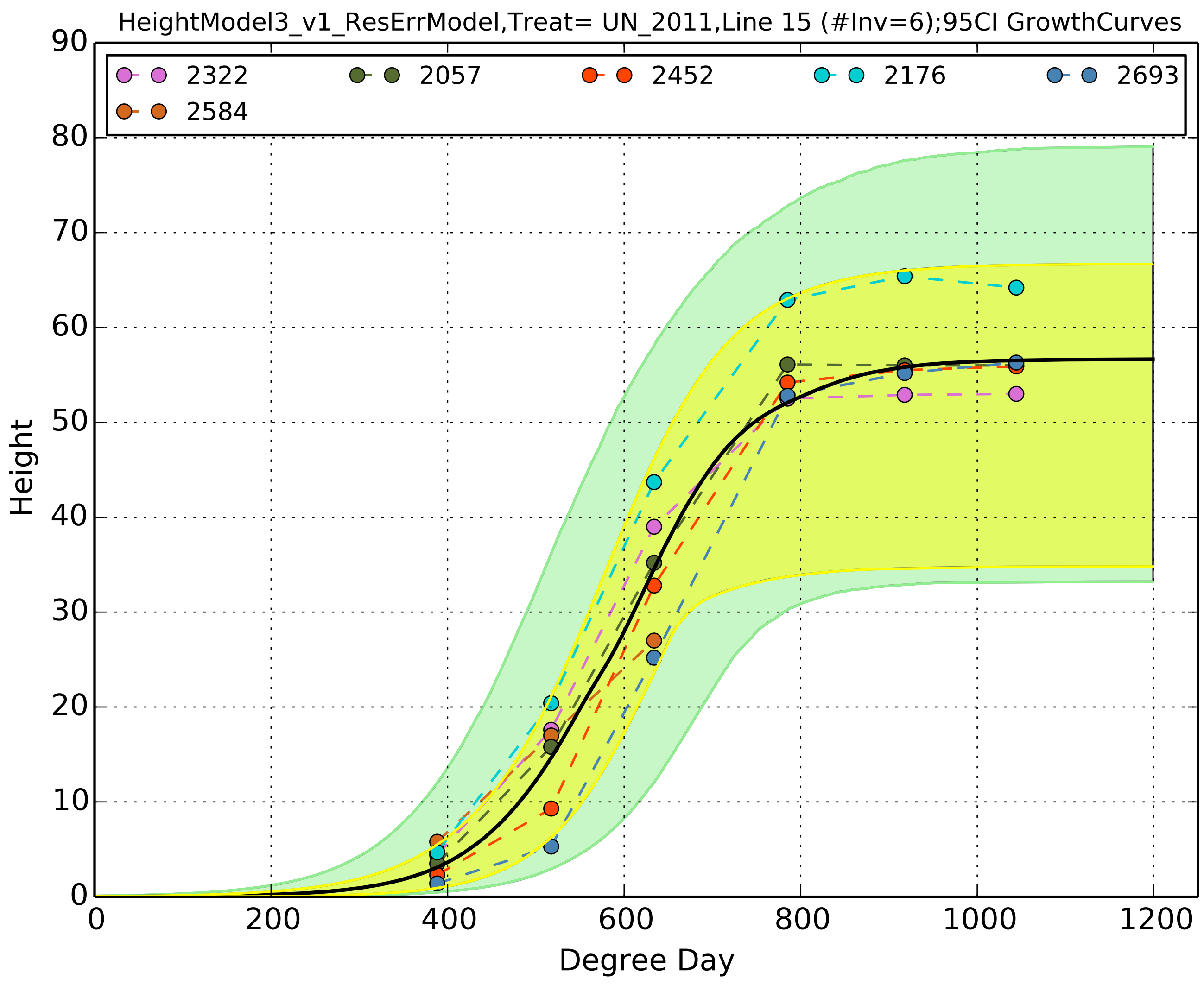
HeightModel3_v1_ResErrModel,Treat= UN_2011,Line 15 (#Inv=6);95CI GrowthCurves
2322
2057
2452
2176
2693
2584
Height
Degree Day
0
10
20
30
40
50
60
70
80
90
0
200
400
600
800
1000
1200

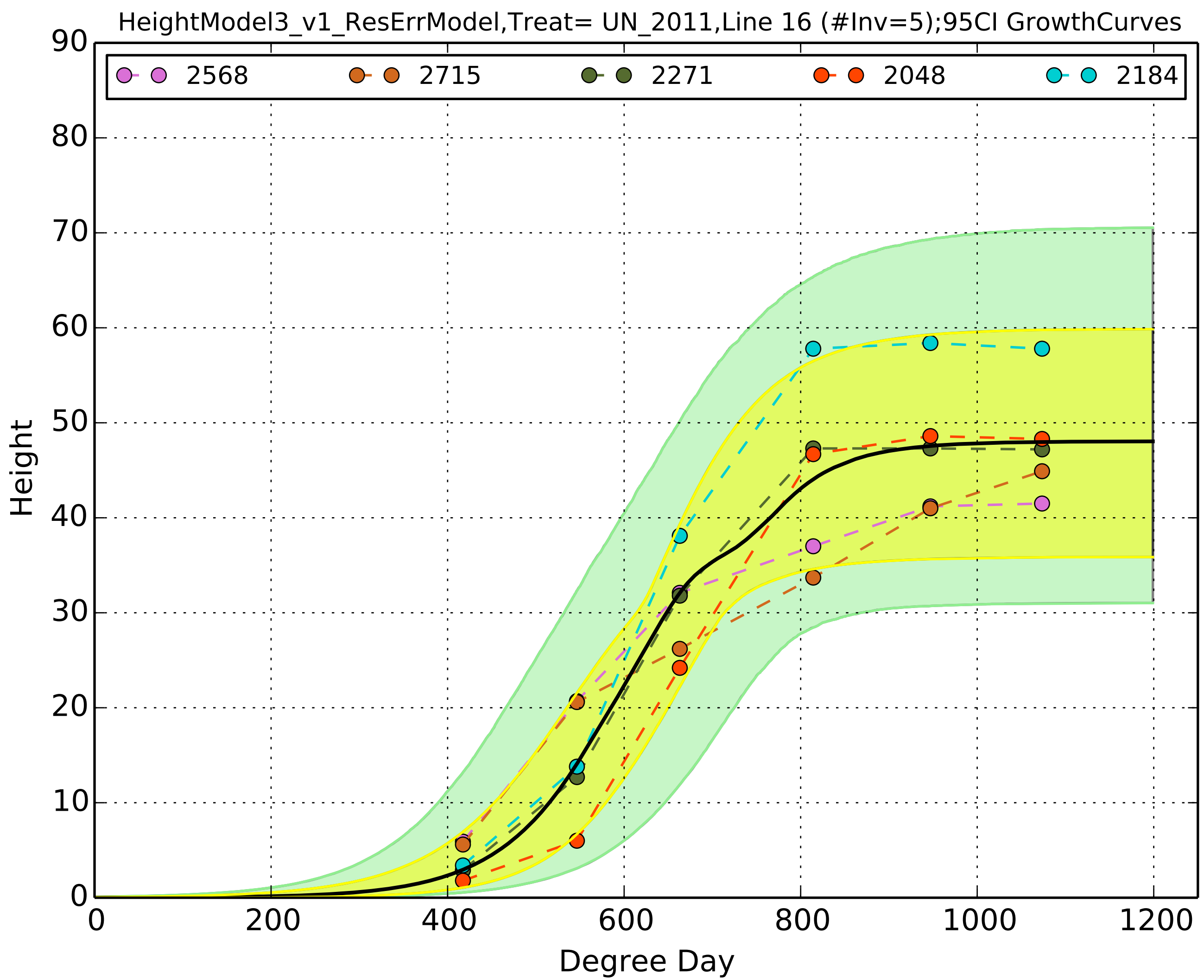

HeightModel3_v1_ResErrModel,Treat= UN_2011,Line 16 (#Inv=5);95CI GrowthCurves
2568
2715
2271
2048
2184
Height
Degree Day
0
10
20
30
40
50
60
70
80
90
0
200
400
600
800
1000
1200

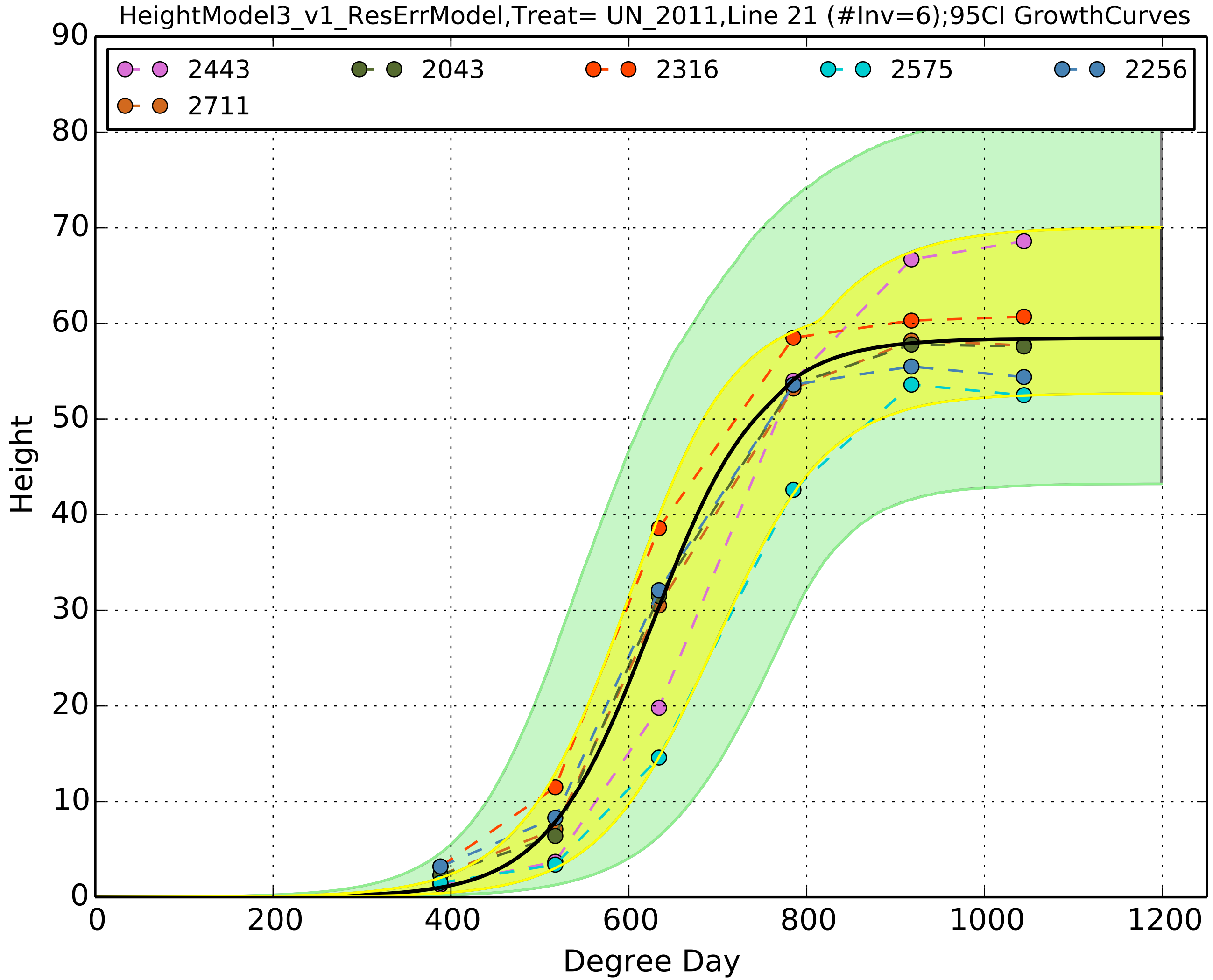

HeightModel3_v1_ResErrModel,Treat= UN_2011,Line 21 (#Inv=6);95CI GrowthCurves
2443
2043
2316
2575
2256
2711
Height
Degree Day
0
10
20
30
40
50
60
70
80
90
0
200
400
600
800
1000
1200

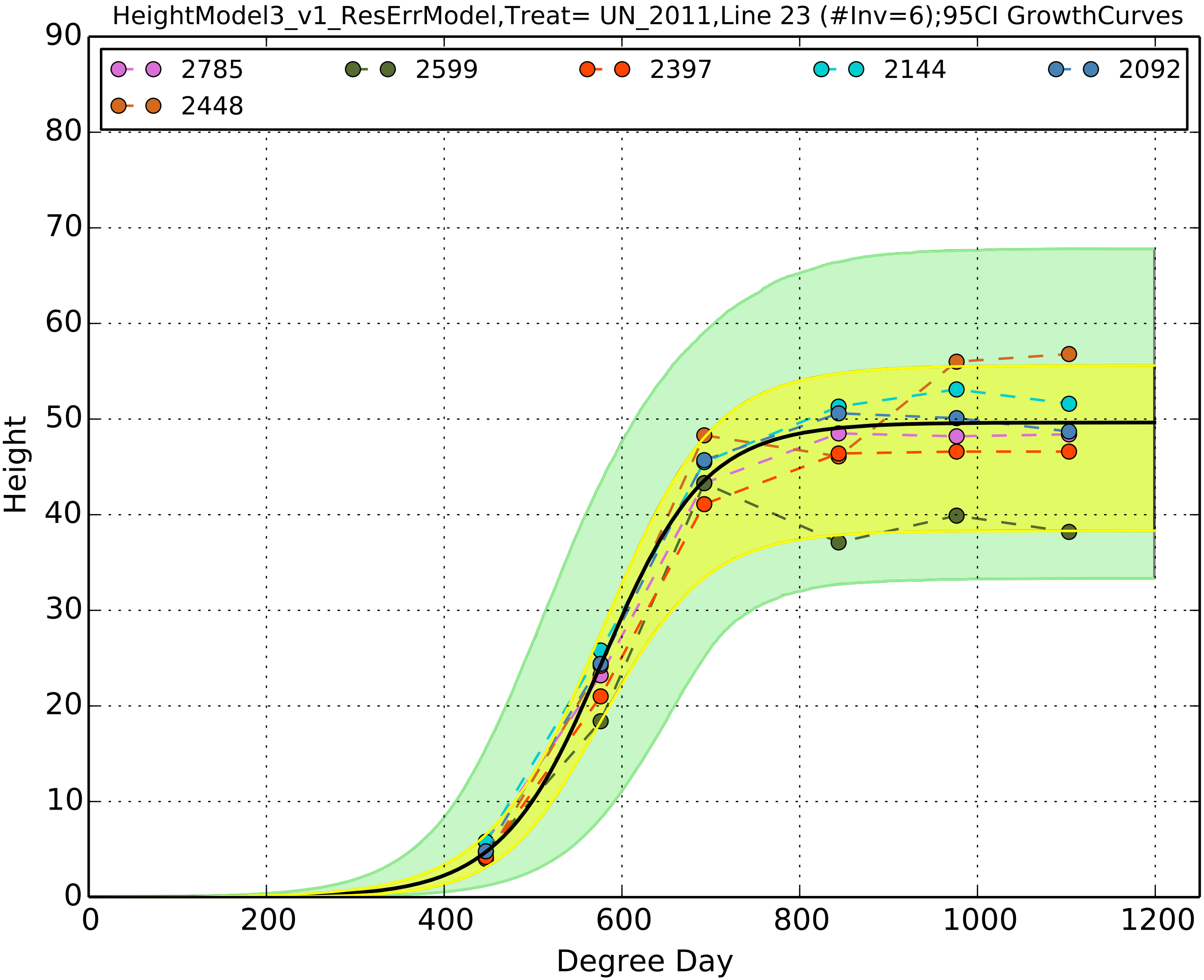

HeightModel3_v1_ResErrModel,Treat= UN_2011,Line 23 (#Inv=6);95CI GrowthCurves
2785
2599
2397
2144
2092
2448
Height
Degree Day
0
10
20
30
40
50
60
70
80
90
0
200
400
600
800
1000
1200

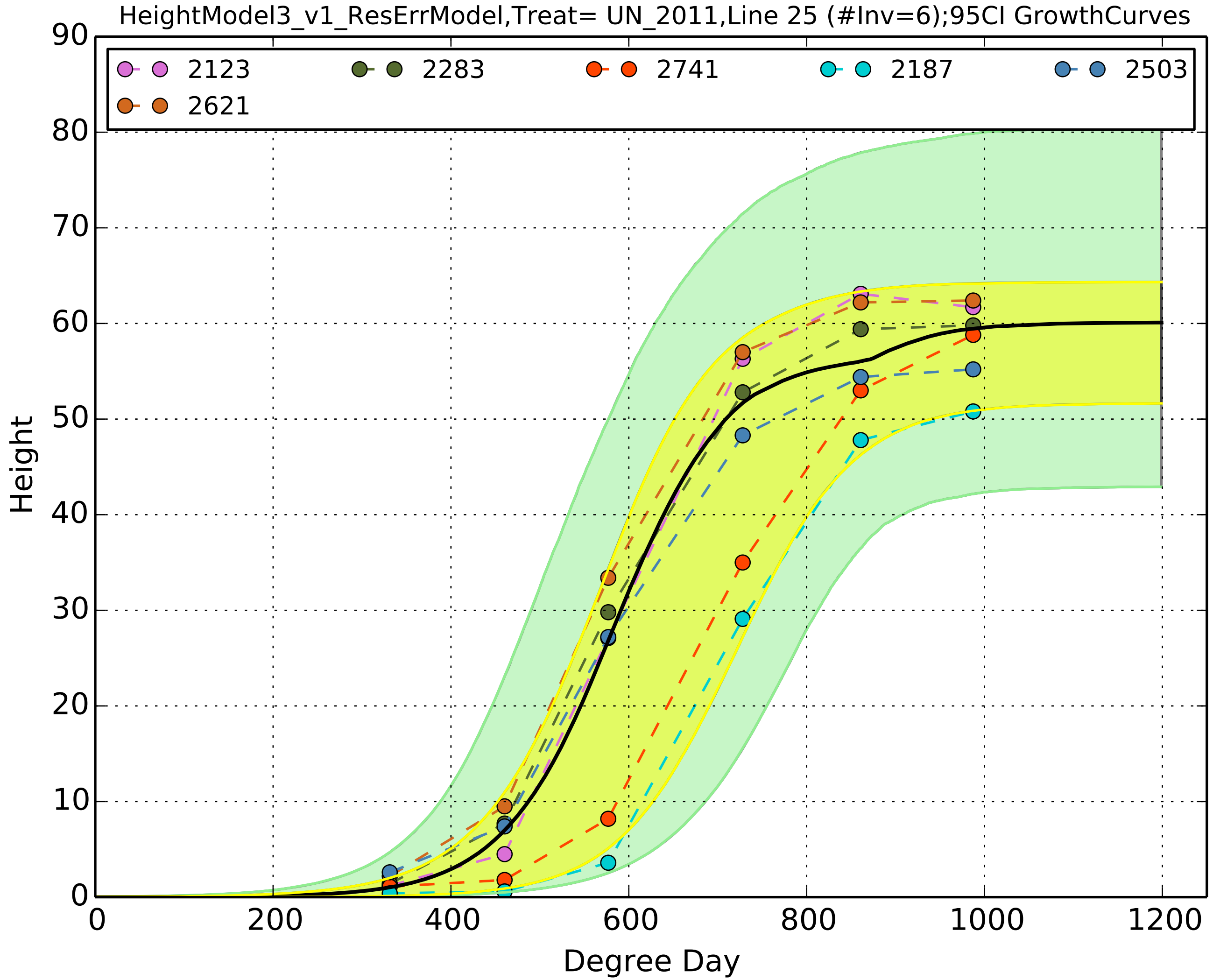

HeightModel3_v1_ResErrModel,Treat= UN_2011,Line 25 (#Inv=6);95CI GrowthCurves
2123
2283
2741
2187
2503
2621
Height
Degree Day
0
10
20
30
40
50
60
70
80
90
0
200
400
600
800
1000
1200

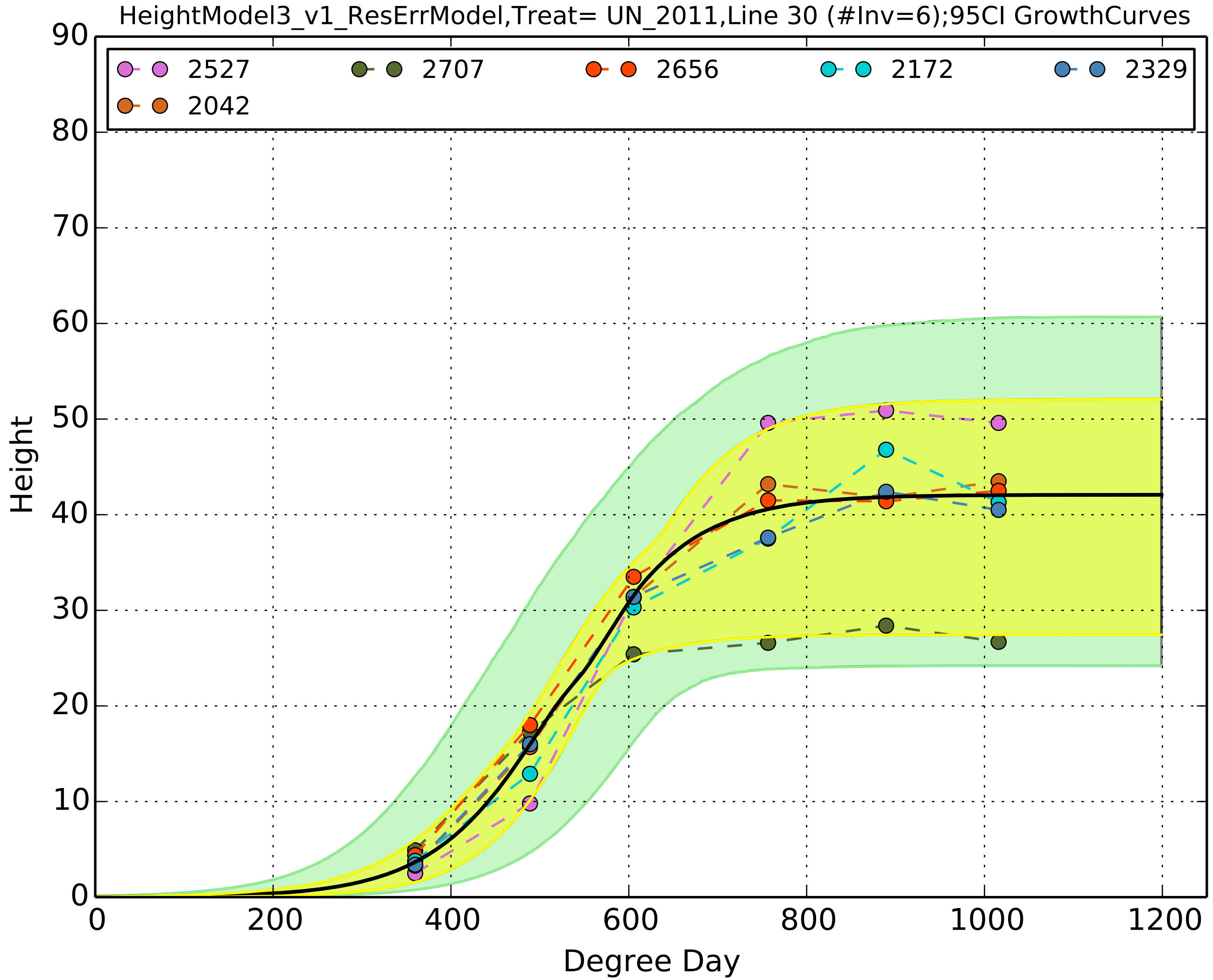

HeightModel3_v1_ResErrModel,Treat= UN_2011,Line 30 (#Inv=6);95CI GrowthCurves
2527
2707
2656
2172
2329
2042
Height
Degree Day
0
10
20
30
40
50
60
70
80
90
0
200
400
600
800
1000
1200

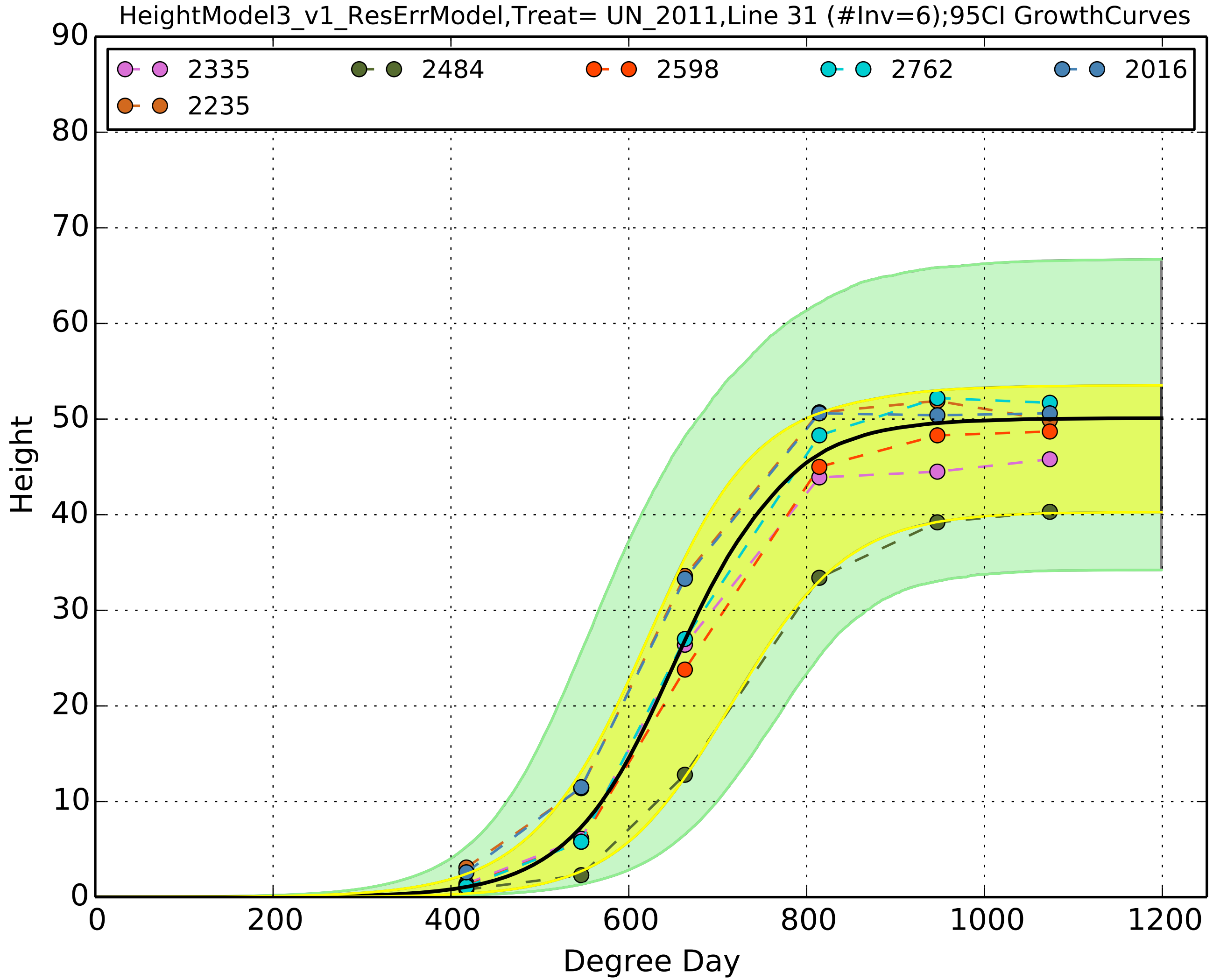

HeightModel3_v1_ResErrModel,Treat= UN_2011,Line 31 (#Inv=6);95CI GrowthCurves
2335
2484
2598
2762
2016
2235
Height
Degree Day
0
10
20
30
40
50
60
70
80
90
0
200
400
600
800
1000
1200

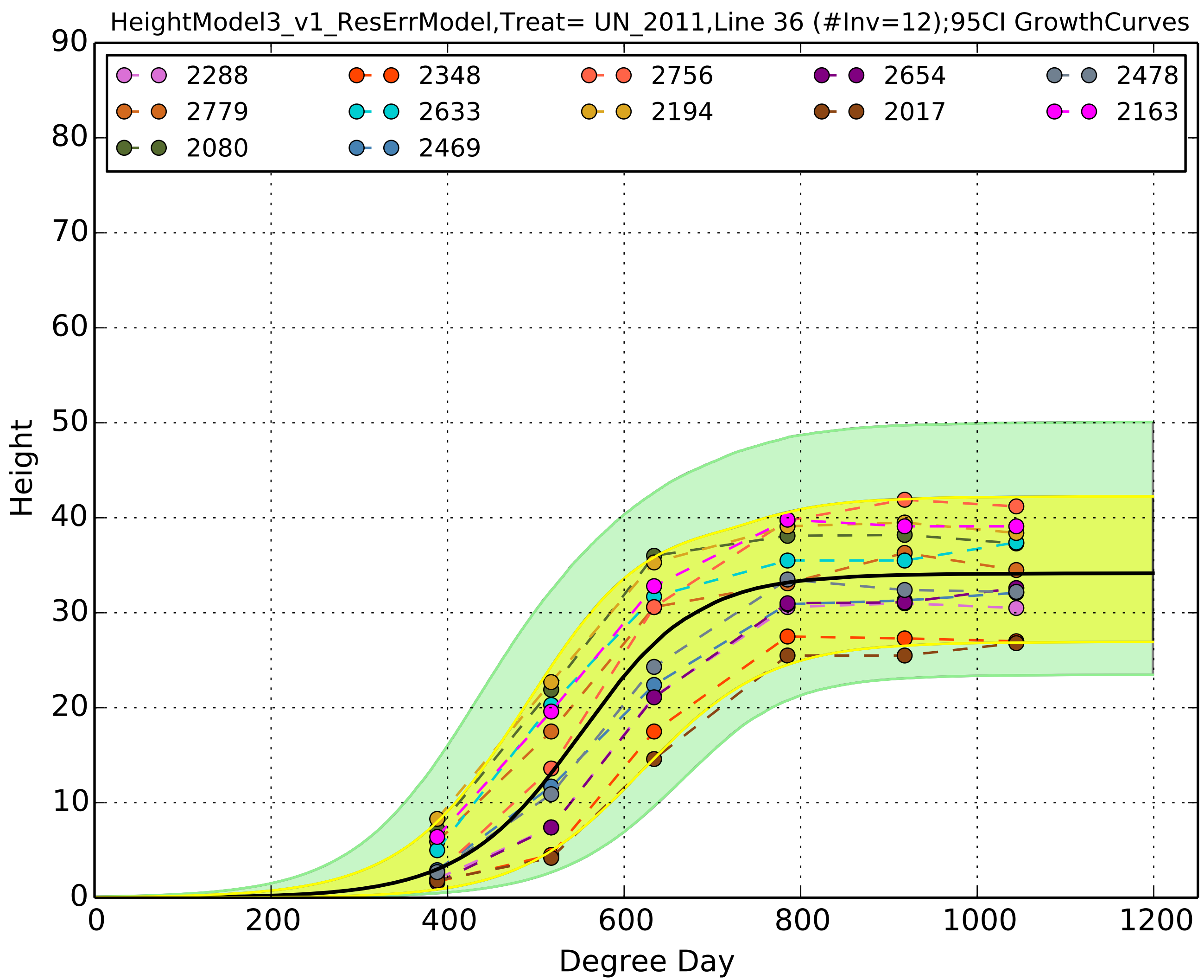

HeightModel3_v1_ResErrModel,Treat= UN_2011,Line 36 (#Inv=12);95CI GrowthCurves
2288
2348
2756
2654
2478
2779
2633
2194
2017
2163
2080
2469
Height
Degree Day
0
10
20
30
40
50
60
70
80
90
0
200
400
600
800
1000
1200

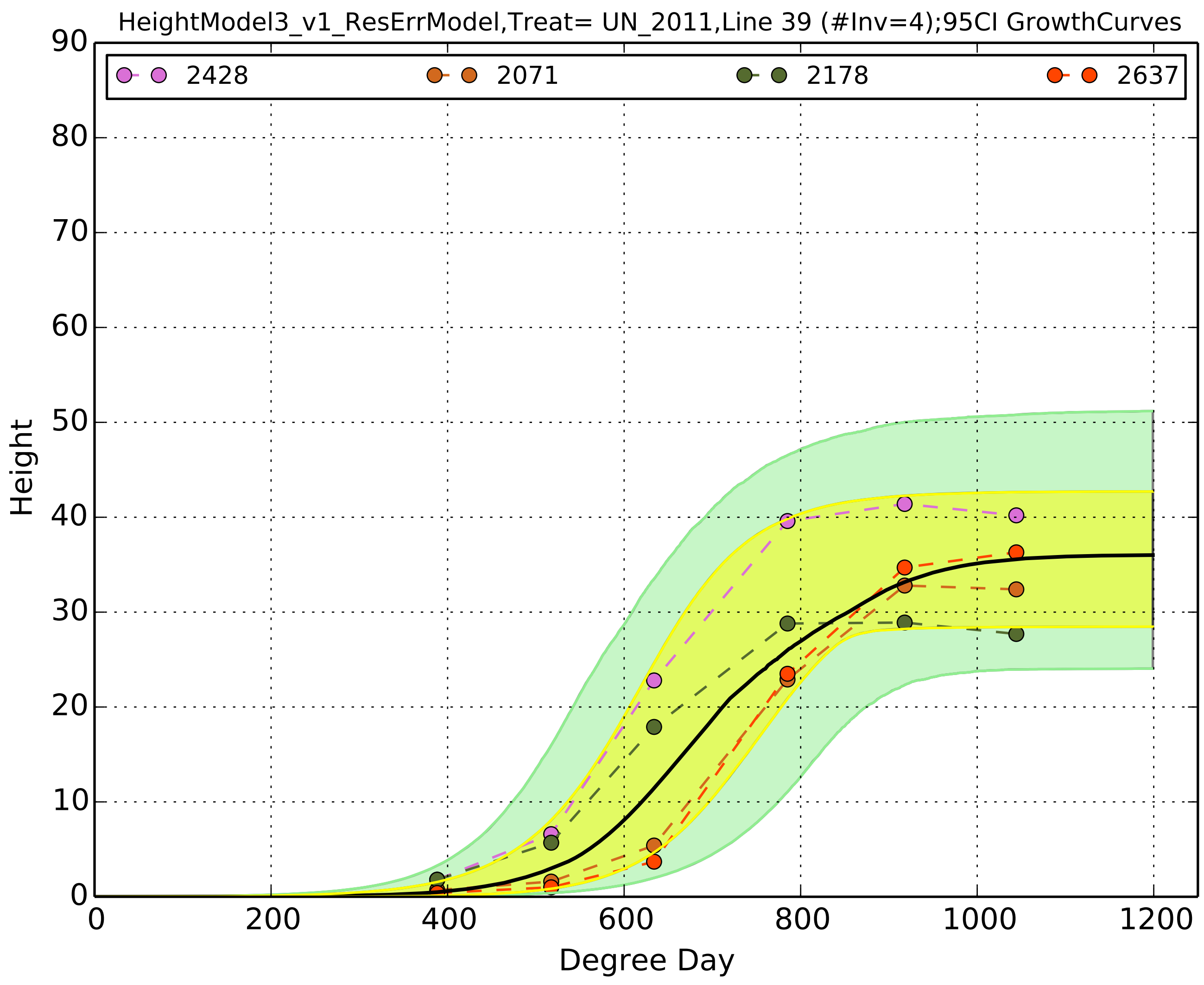

HeightModel3_v1_ResErrModel,Treat= UN_2011,Line 39 (#Inv=4);95CI GrowthCurves
2428
2071
2178
2637
Height
Degree Day
0
10
20
30
40
50
60
70
80
90
0
200
400
600
800
1000
1200

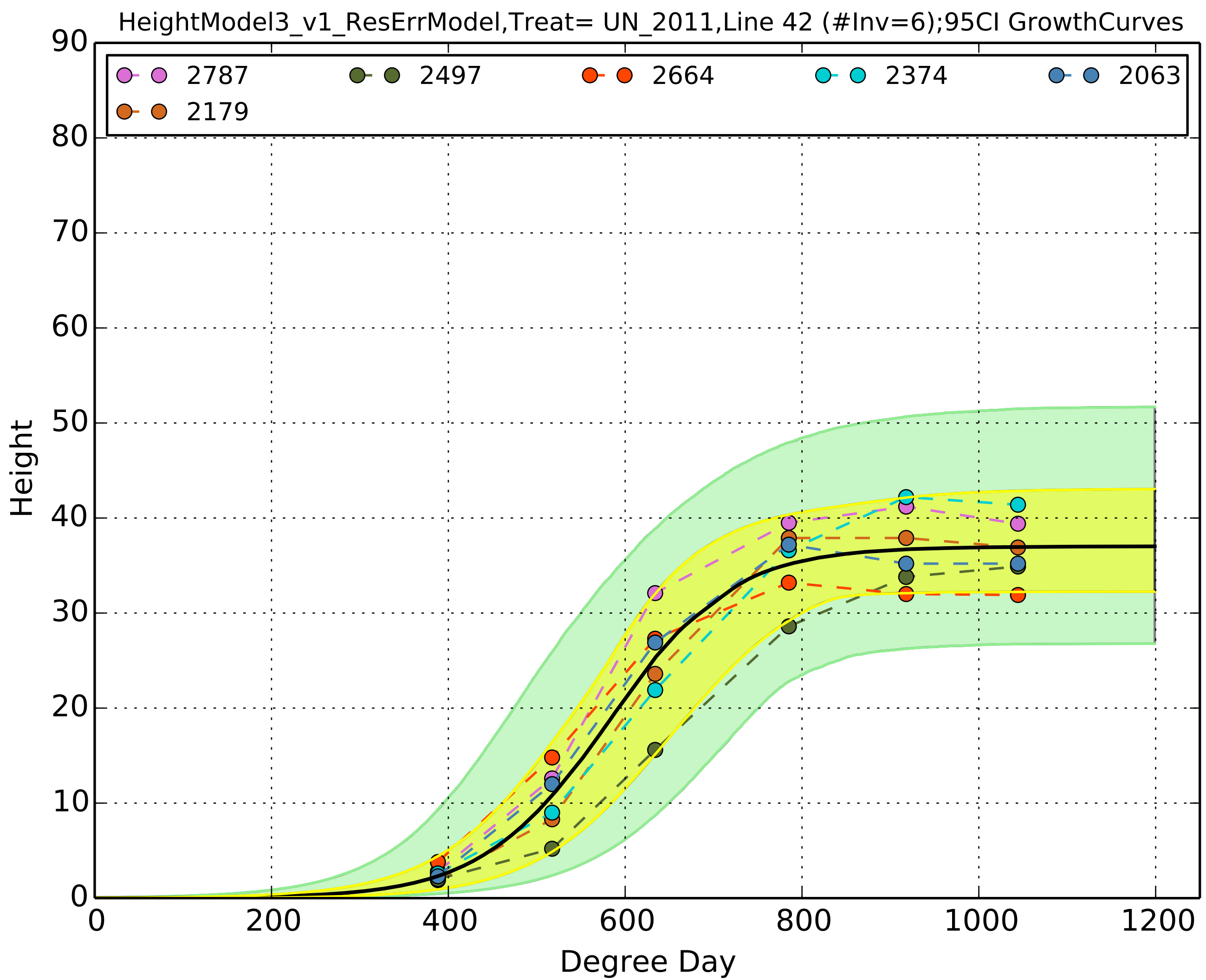
HeightModel3_v1_ResErrModel,Treat= UN_2011,Line 42 (#Inv=6);95CI GrowthCurves
2787
2497
2664
2374
2063
2179
Height
Degree Day
0
10
20
30
40
50
60
70
80
90
0
200
400
600
800
1000
1200

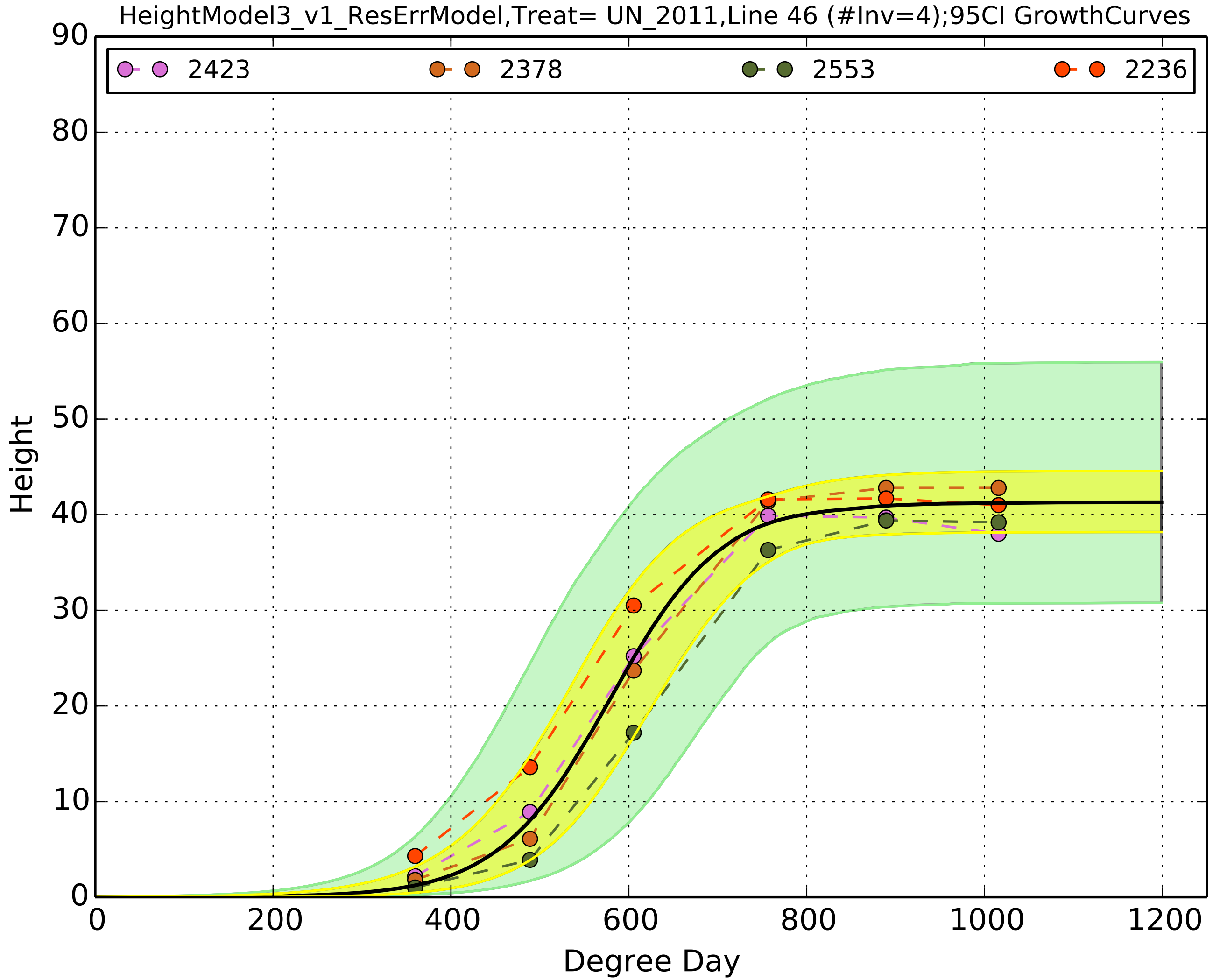

HeightModel3_v1_ResErrModel,Treat= UN_2011,Line 46 (#Inv=4);95CI GrowthCurves
2423
2378
2553
2236
Height
Degree Day
0
10
20
30
40
50
60
70
80
90
0
200
400
600
800
1000
1200

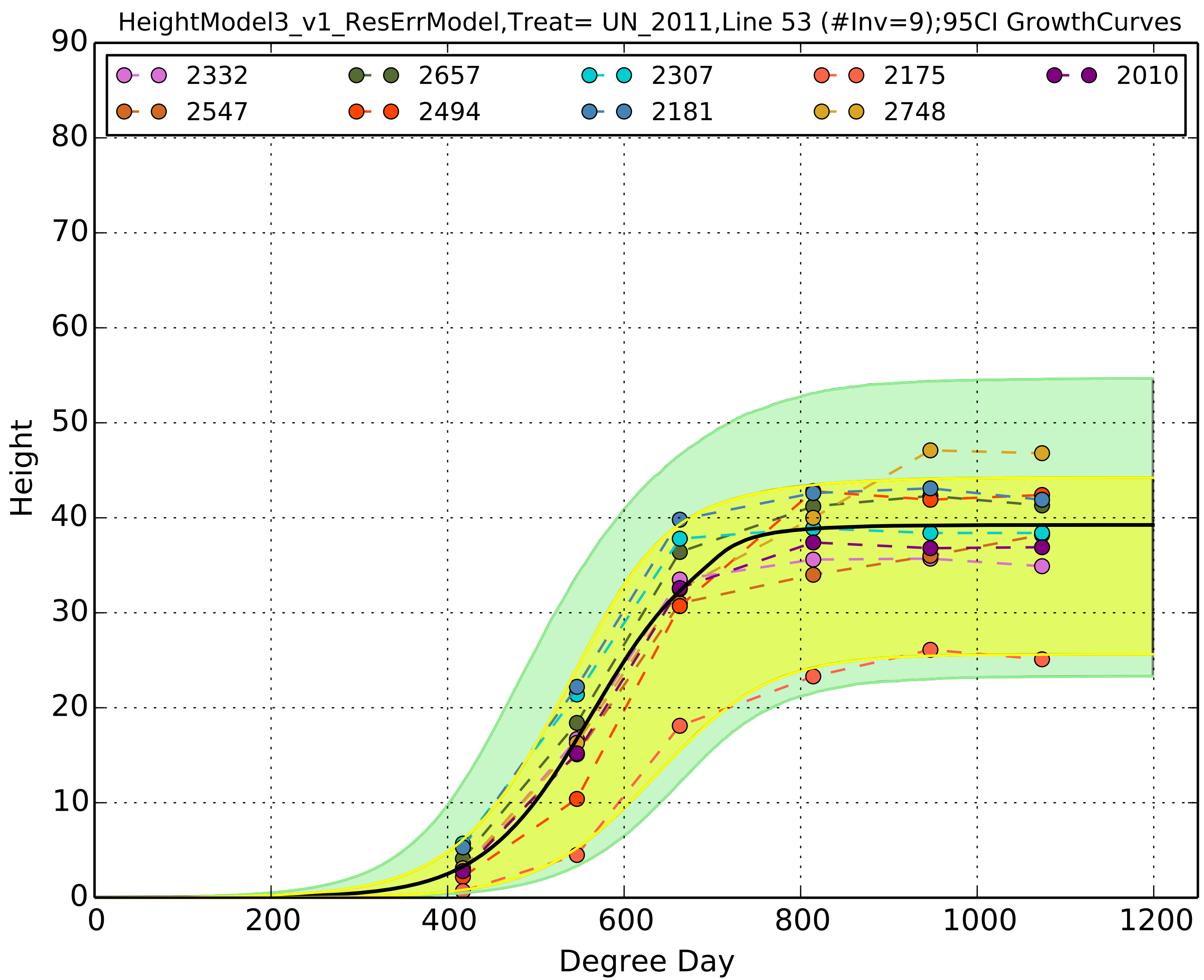
HeightModel3_v1_ResErrModel,Treat= UN_2011,Line 53 (#Inv=9);95CI GrowthCurves
2332
2657
2307
2175
2010
2547
2494
2181
2748
Height
Degree Day
0
10
20
30
40
50
60
70
80
90
0
200
400
600
800
1000
1200

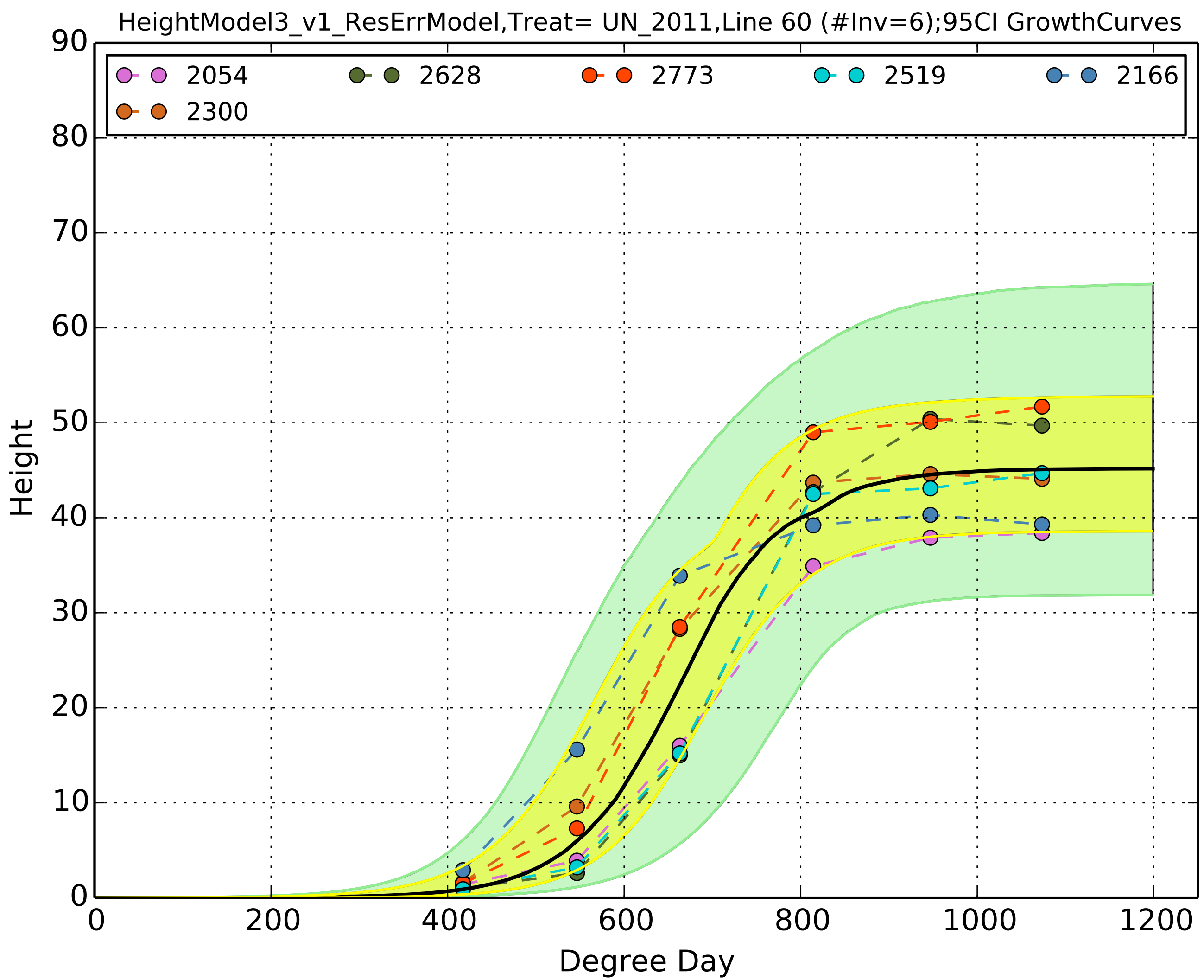

HeightModel3_v1_ResErrModel,Treat= UN_2011,Line 60 (#Inv=6);95CI GrowthCurves
2054
2628
2773
2519
2166
2300
Height
Degree Day
0
10
20
30
40
50
60
70
80
90
0
200
400
600
800
1000
1200

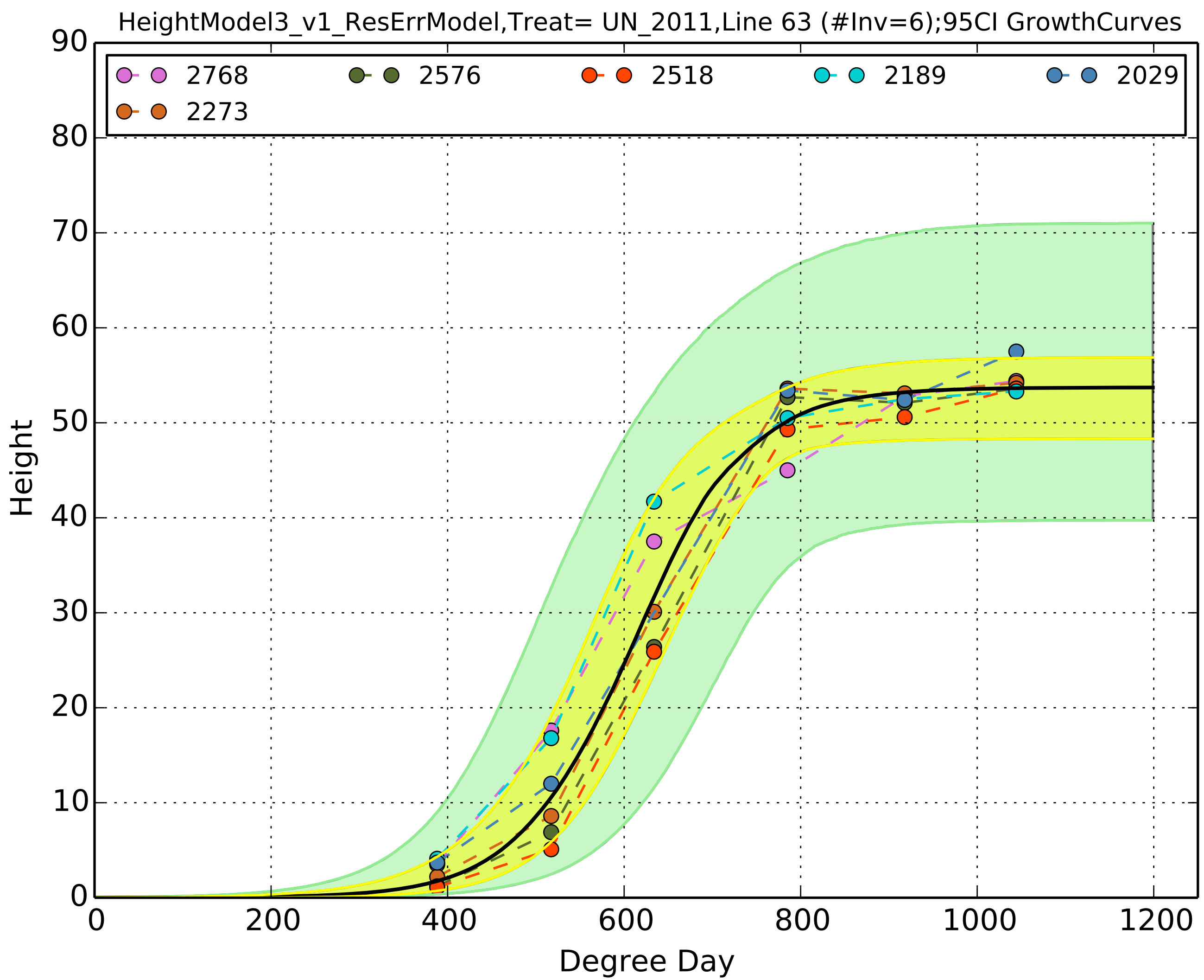

HeightModel3_v1_ResErrModel,Treat= UN_2011,Line 63 (#Inv=6);95CI GrowthCurves
2768
2576
2518
2189
2029
2273
Height
Degree Day
0
10
20
30
40
50
60
70
80
90
0
200
400
600
800
1000
1200

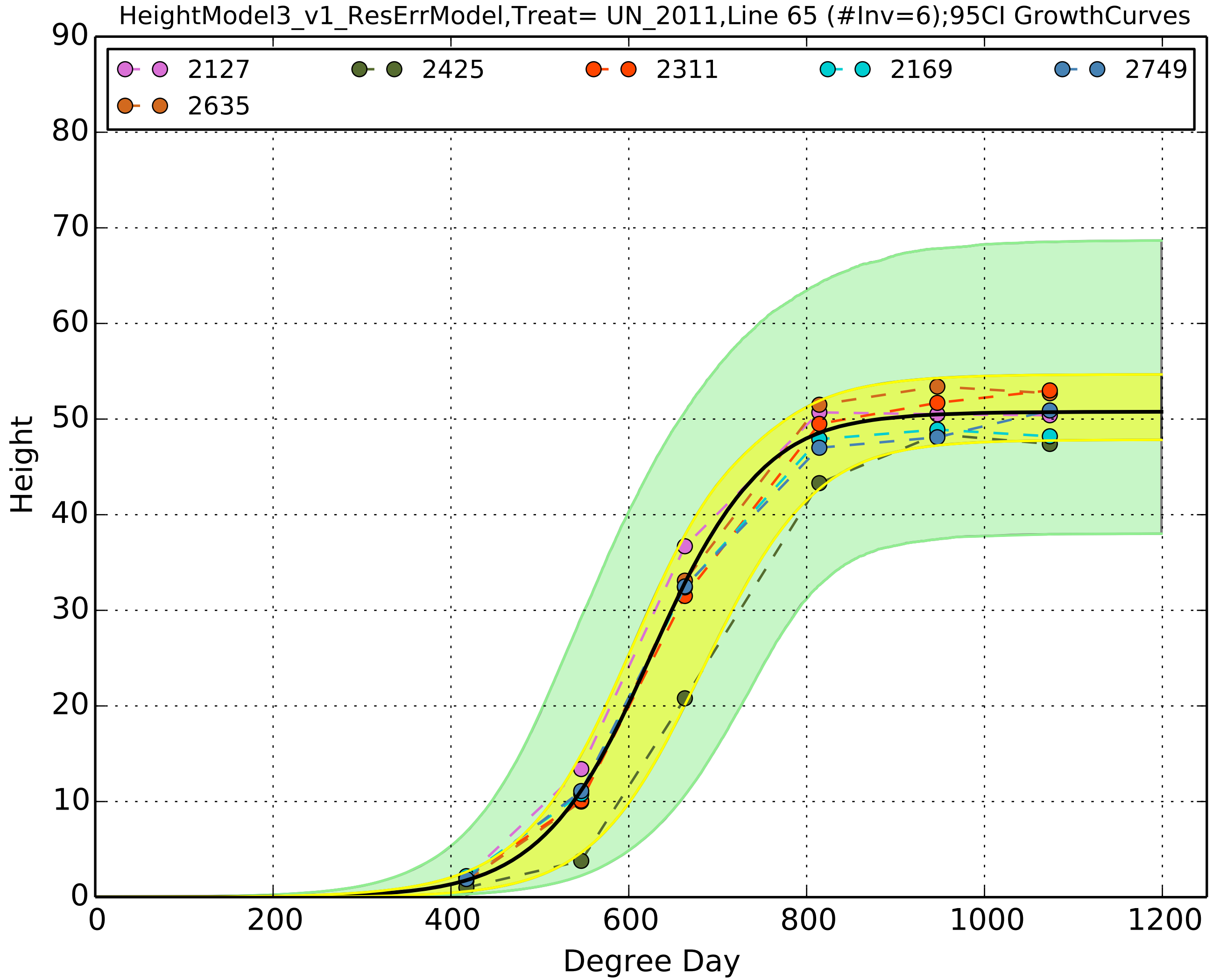

HeightModel3_v1_ResErrModel,Treat= UN_2011,Line 65 (#Inv=6);95CI GrowthCurves
2127
2425
2311
2169
2749
2635
Height
Degree Day
0
10
20
30
40
50
60
70
80
90
0
200
400
600
800
1000
1200

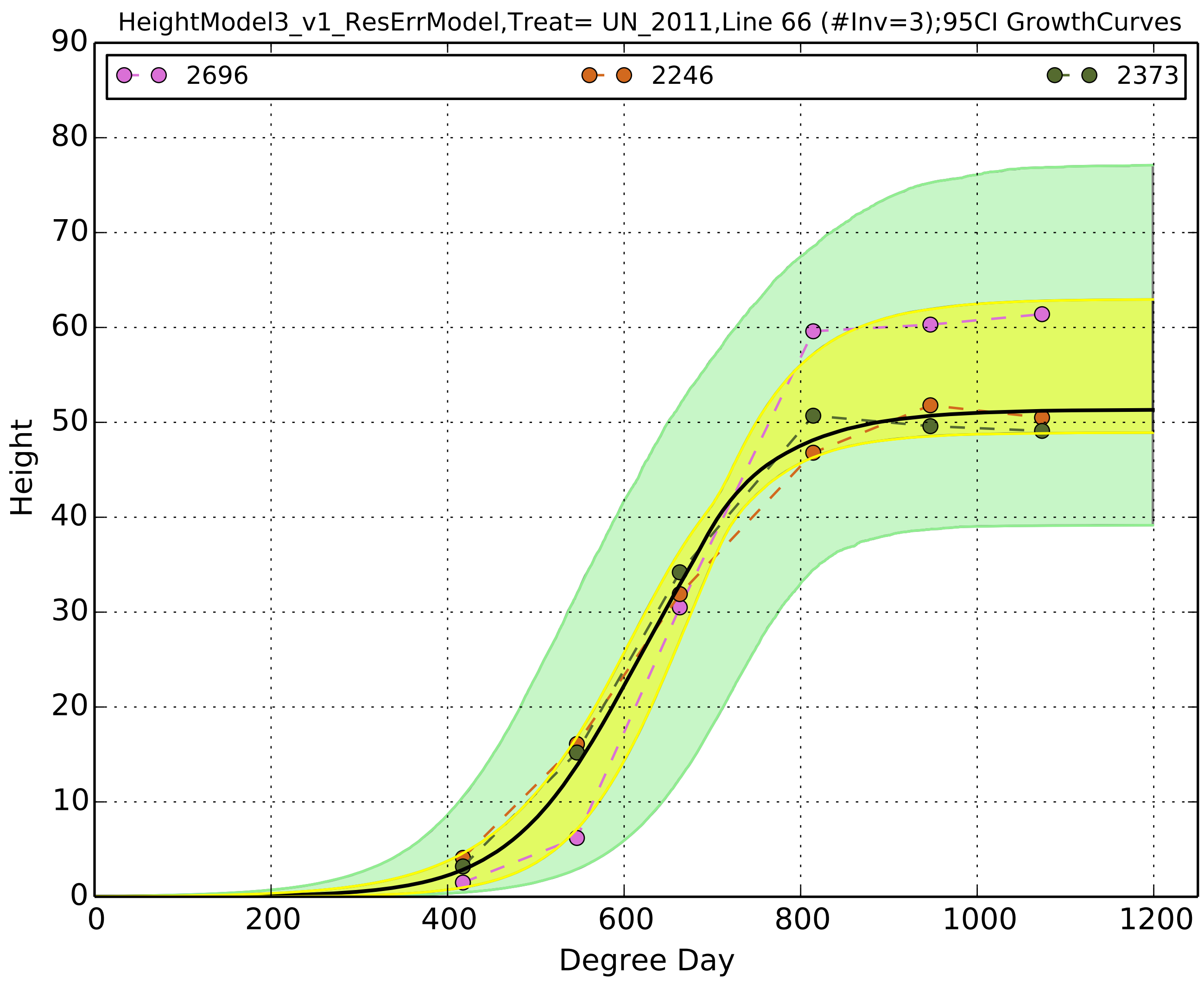

HeightModel3_v1_ResErrModel,Treat= UN_2011,Line 66 (#Inv=3);95CI GrowthCurves
2696
2246
2373
Height
Degree Day
0
10
20
30
40
50
60
70
80
90
0
200
400
600
800
1000
1200

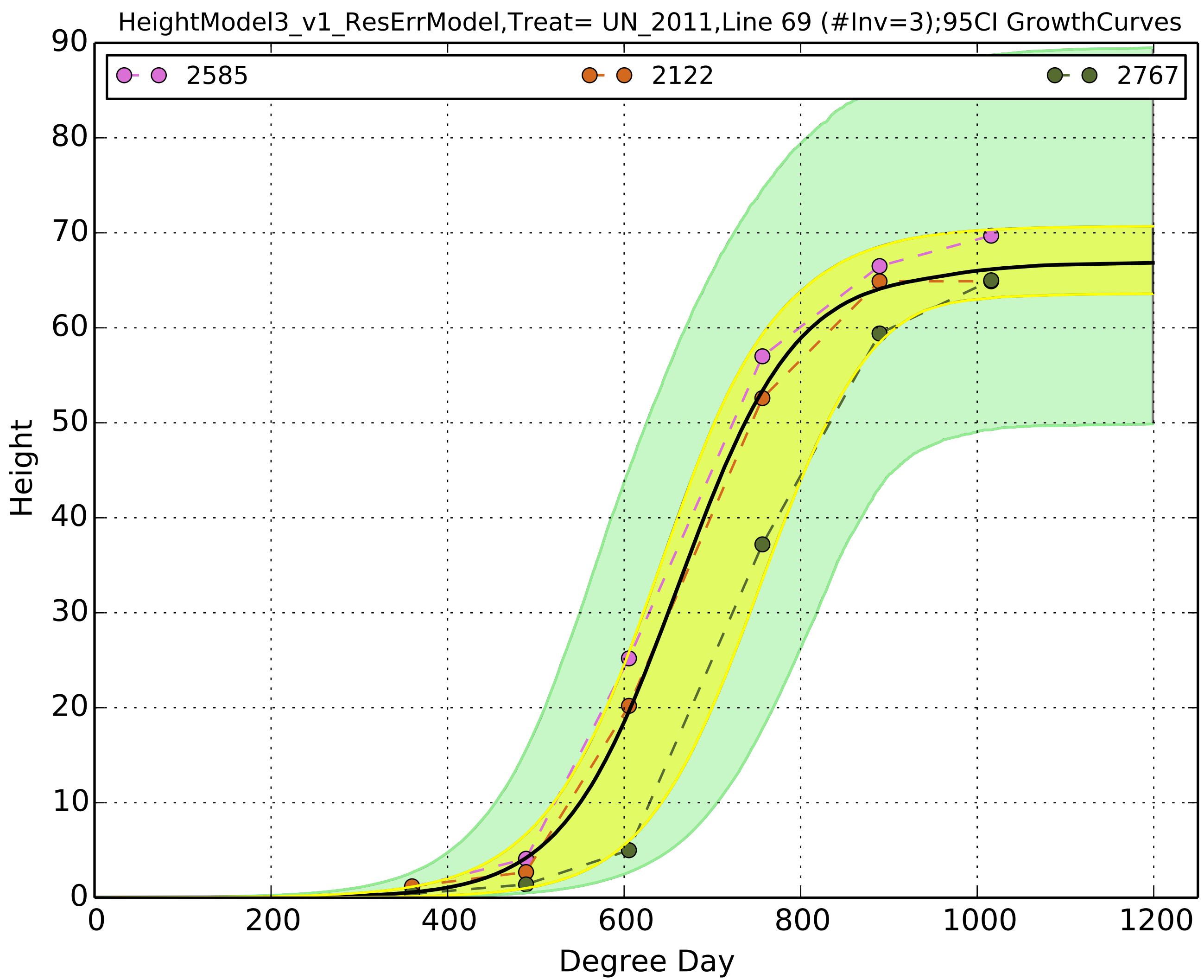

HeightModel3_v1_ResErrModel,Treat= UN_2011,Line 69 (#Inv=3);95CI GrowthCurves
2585
2122
2767
Height
Degree Day
0
10
20
30
40
50
60
70
80
90
0
200
400
600
800
1000
1200

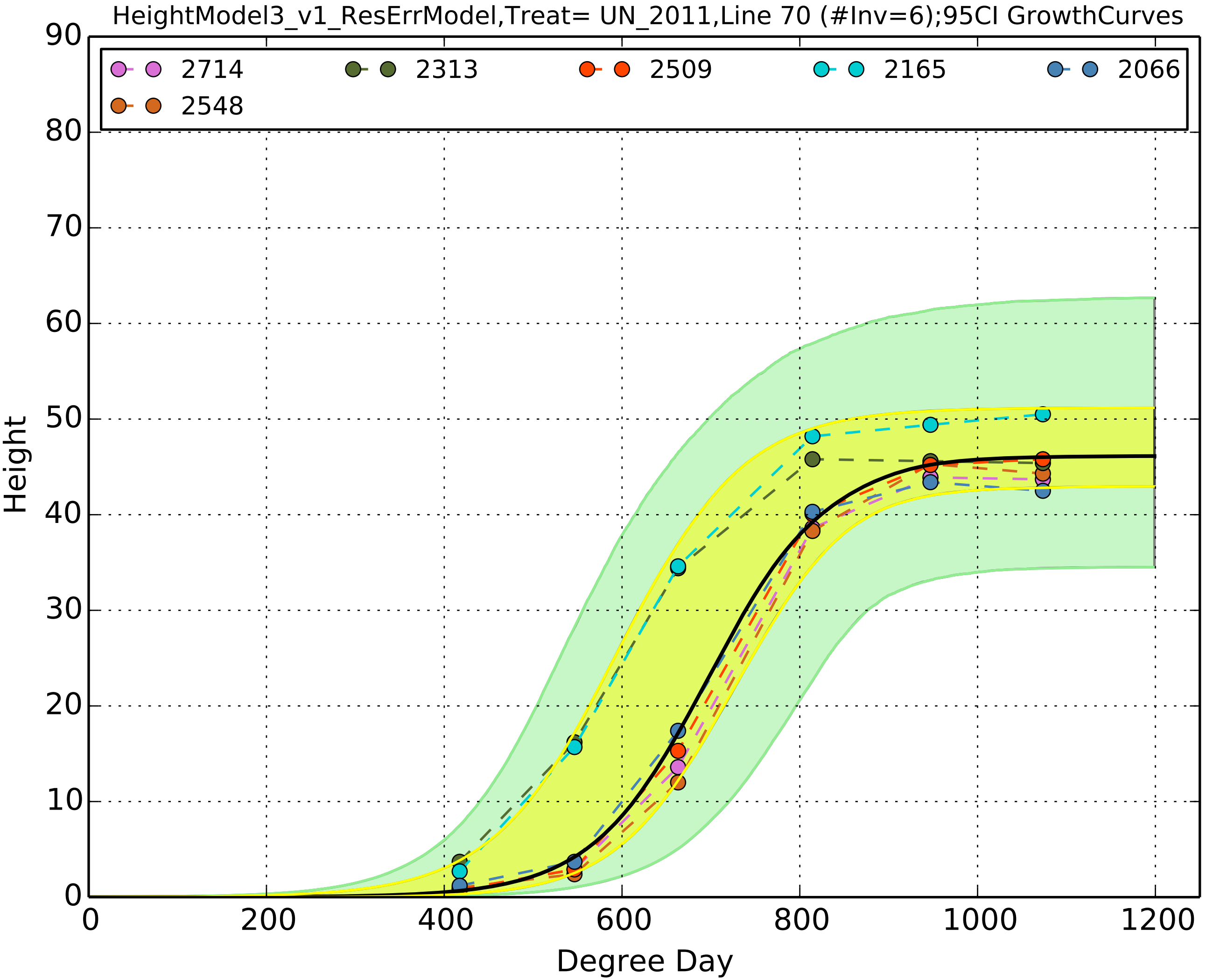
HeightModel3_v1_ResErrModel,Treat= UN_2011,Line 70 (#Inv=6);95CI GrowthCurves
2714
2313
2509
2165
2066
2548
Height
Degree Day
0
10
20
30
40
50
60
70
80
90
0
200
400
600
800
1000
1200

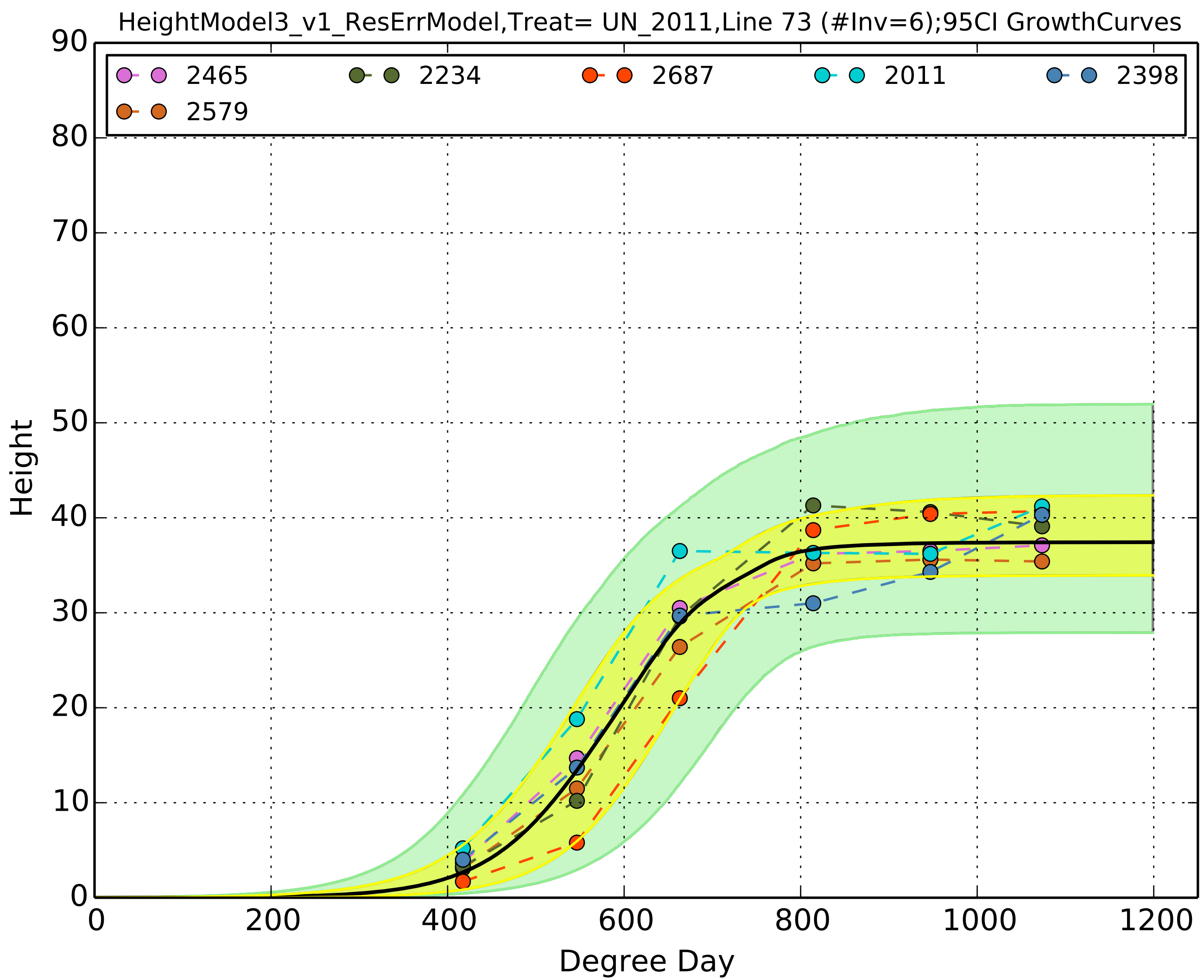

HeightModel3_v1_ResErrModel,Treat= UN_2011,Line 73 (#Inv=6);95CI GrowthCurves
2465
2234
2687
2011
2398
2579
Height
Degree Day
0
10
20
30
40
50
60
70
80
90
0
200
400
600
800
1000
1200

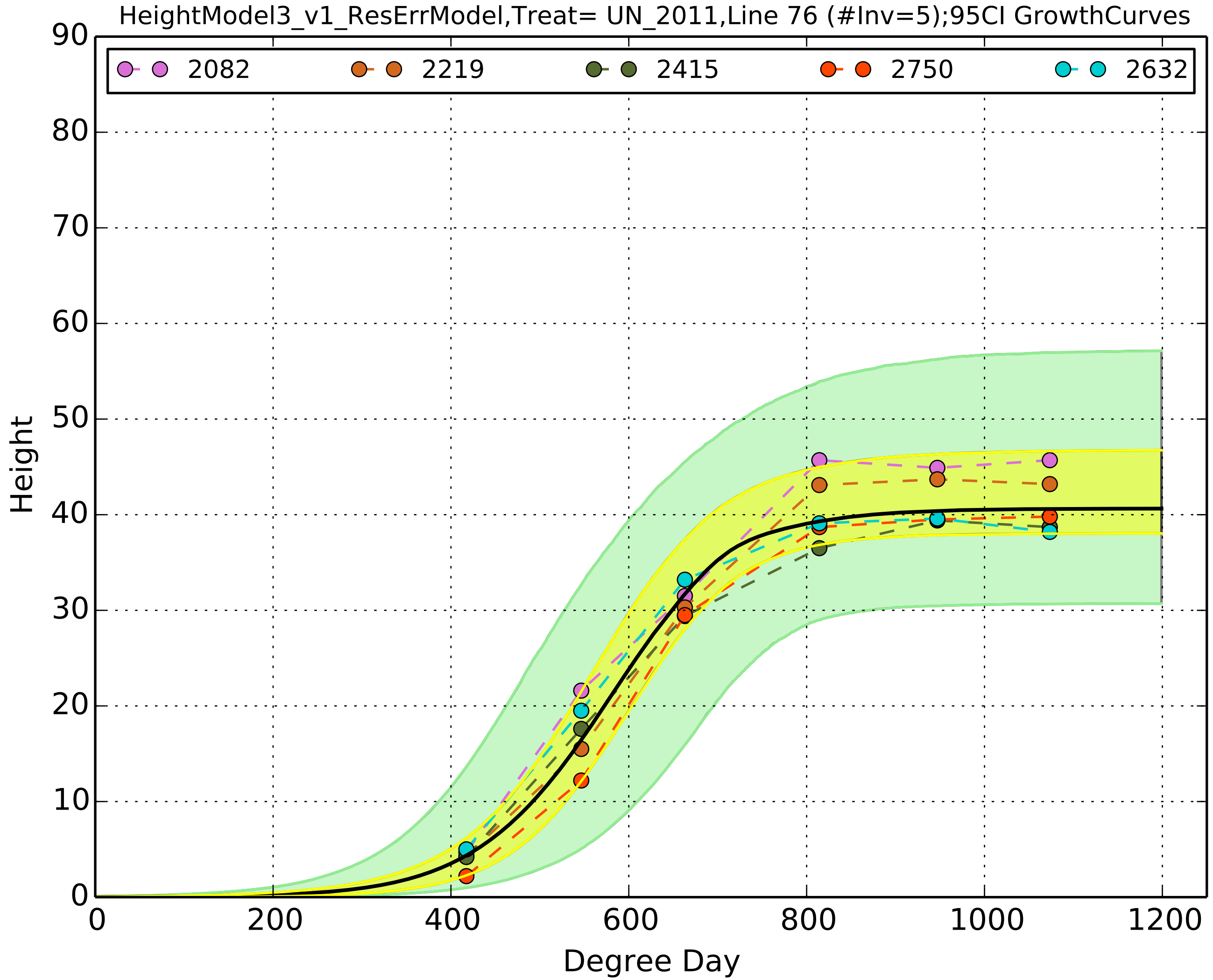
HeightModel3_v1_ResErrModel,Treat= UN_2011,Line 76 (#Inv=5);95CI GrowthCurves
2082
2219
2415
2750
2632
Height
Degree Day
0
10
20
30
40
50
60
70
80
90
0
200
400
600
800
1000
1200

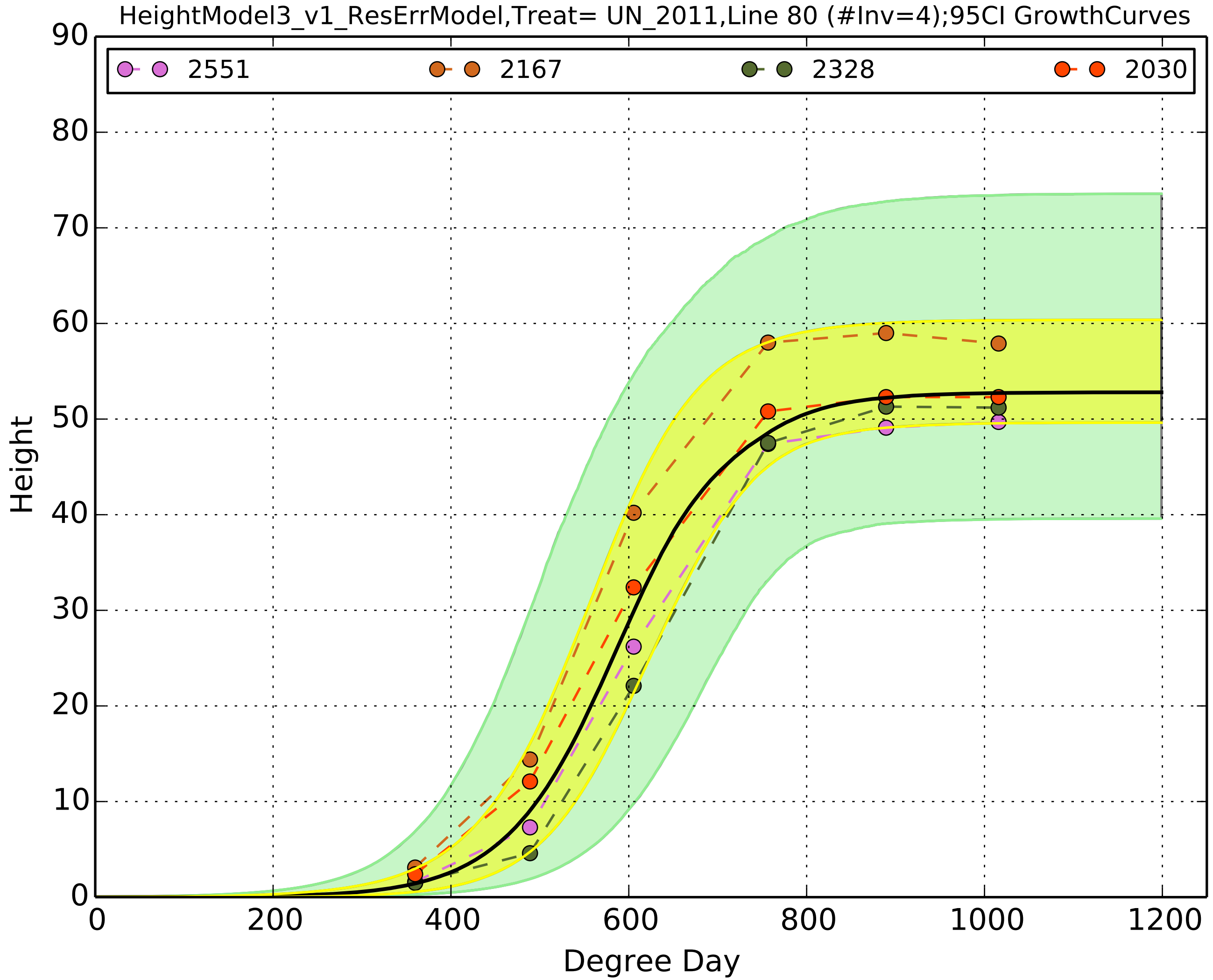

HeightModel3_v1_ResErrModel,Treat= UN_2011,Line 80 (#Inv=4);95CI GrowthCurves
2551
2167
2328
2030
Height
Degree Day
0
10
20
30
40
50
60
70
80
90
0
200
400
600
800
1000
1200

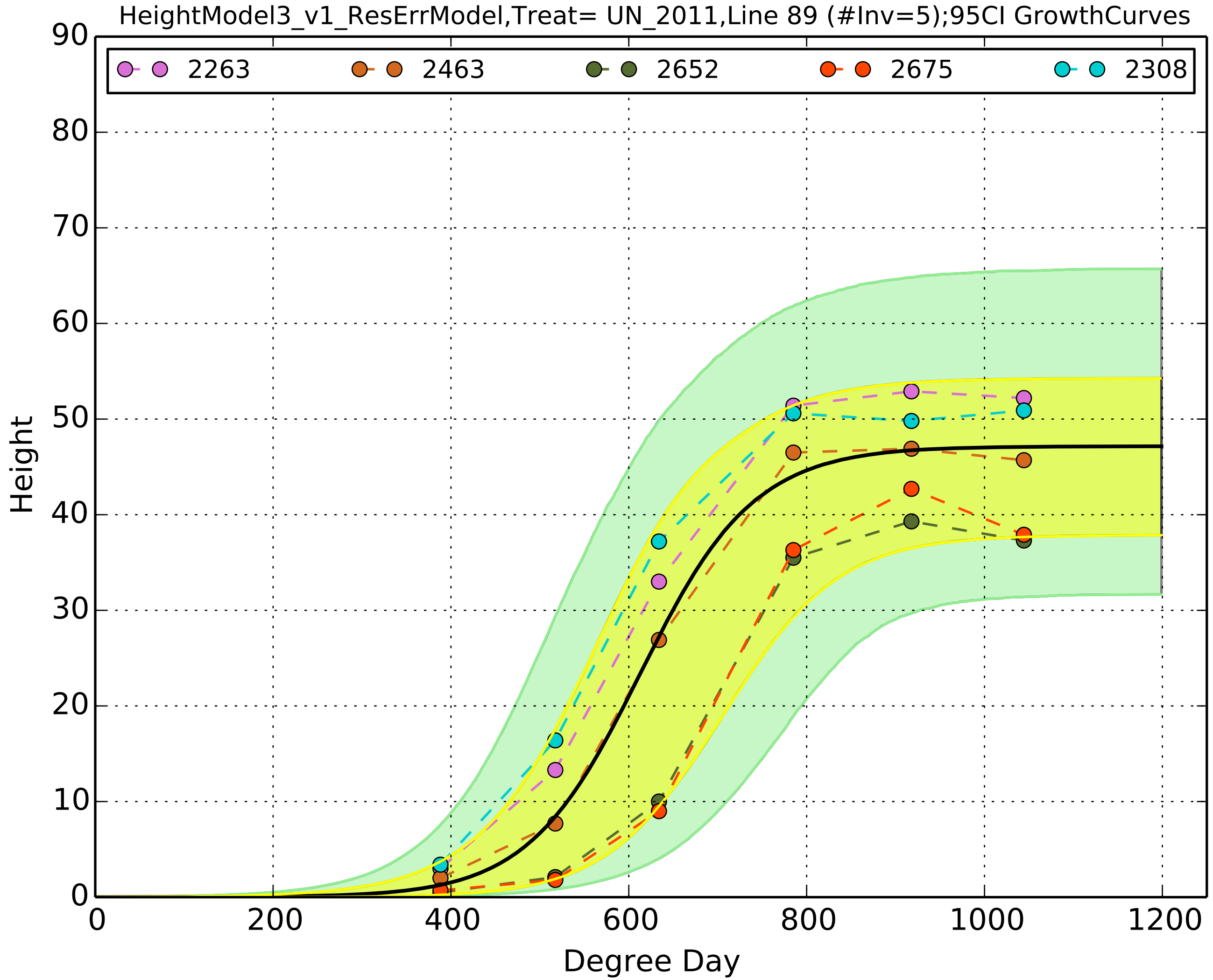

HeightModel3_v1_ResErrModel,Treat= UN_2011,Line 89 (#Inv=5);95CI GrowthCurves
2263
2463
2652
2675
2308
Height
Degree Day
0
10
20
30
40
50
60
70
80
90
0
200
400
600
800
1000
1200

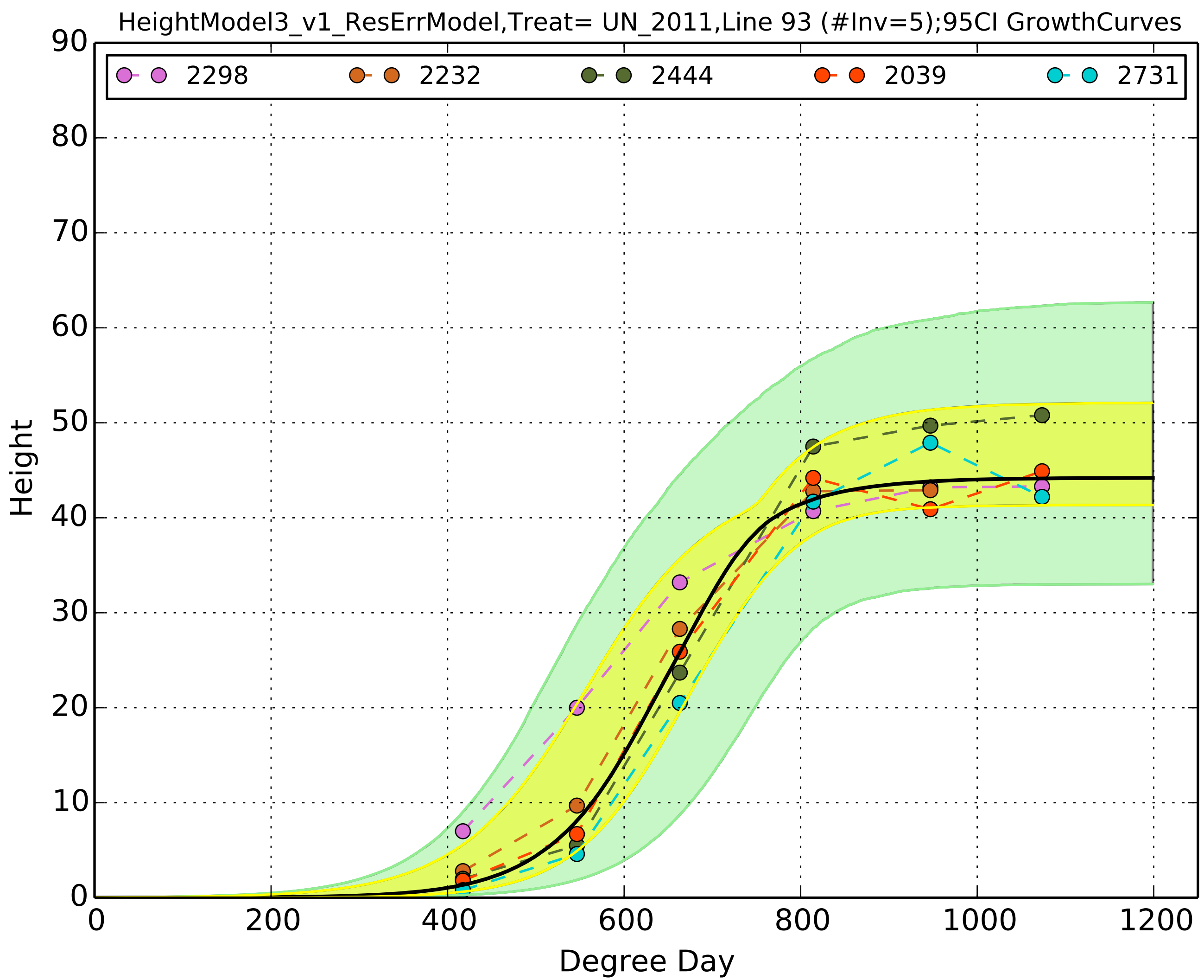
HeightModel3_v1_ResErrModel,Treat= UN_2011,Line 93 (#Inv=5);95CI GrowthCurves
2298
2232
2444
2039
2731
Height
Degree Day
0
10
20
30
40
50
60
70
80
90
0
200
400
600
800
1000
1200

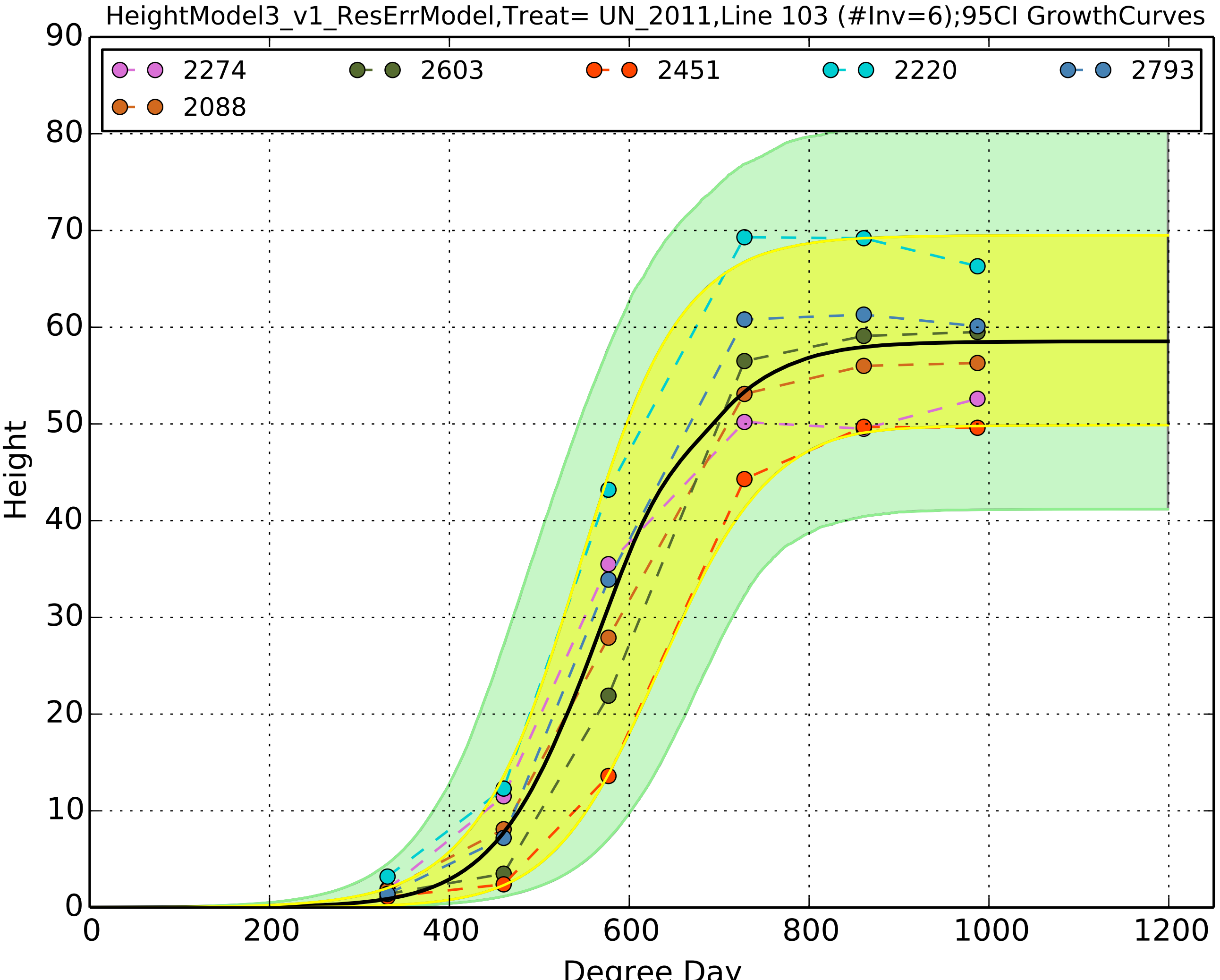
HeightModel3_v1_ResErrModel,Treat= UN_2011,Line 103 (#Inv=6);95CI GrowthCurves
2274
2603
2451
2220
2793
2088
Height
Degree Day
0
10
20
30
40
50
60
70
80
90
0
200
400
600
800
1000
1200

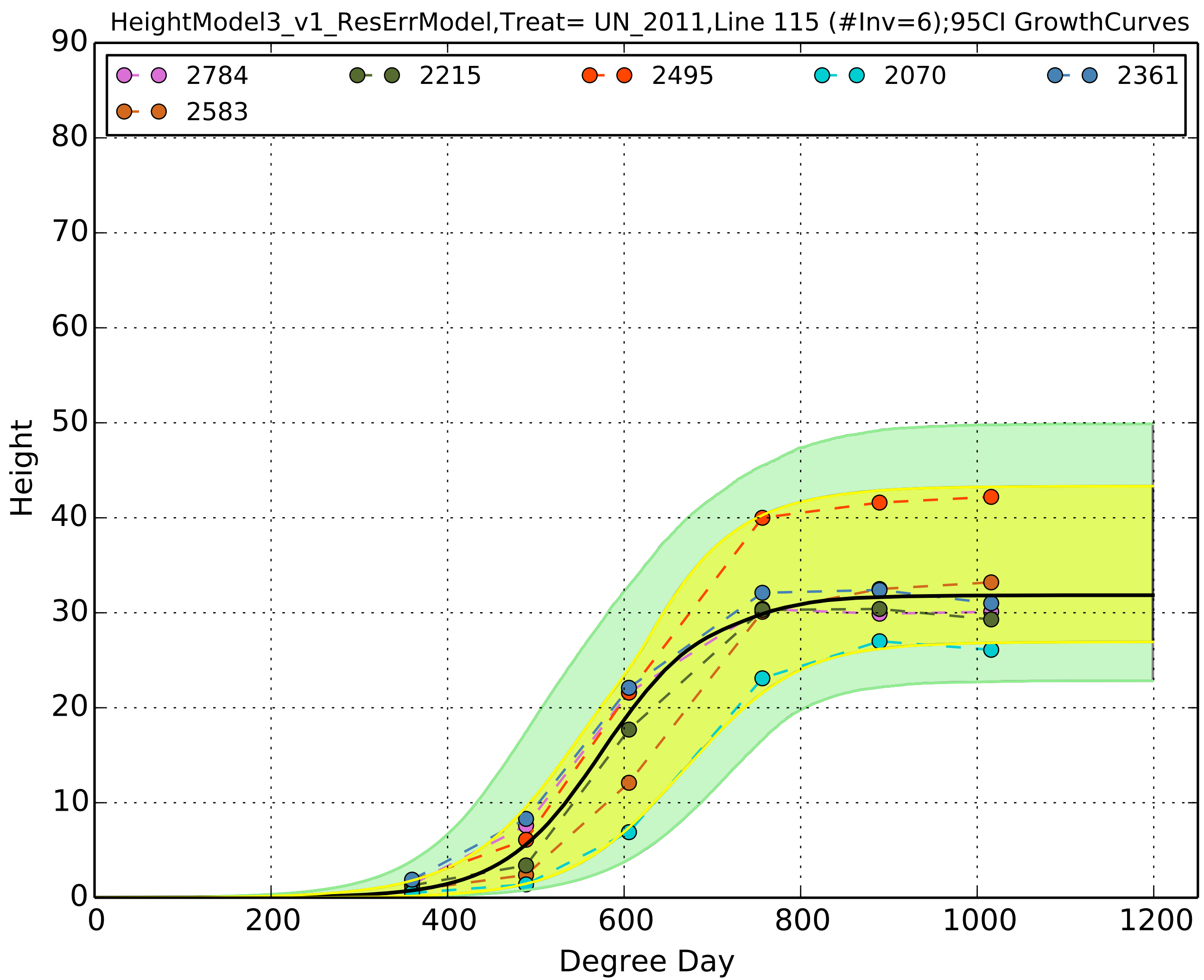
HeightModel3_v1_ResErrModel,Treat= UN_2011,Line 115 (#Inv=6);95CI GrowthCurves
2784
2215
2495
2070
2361
2583
Height
Degree Day
0
10
20
30
40
50
60
70
80
90
0
200
400
600
800
1000
1200

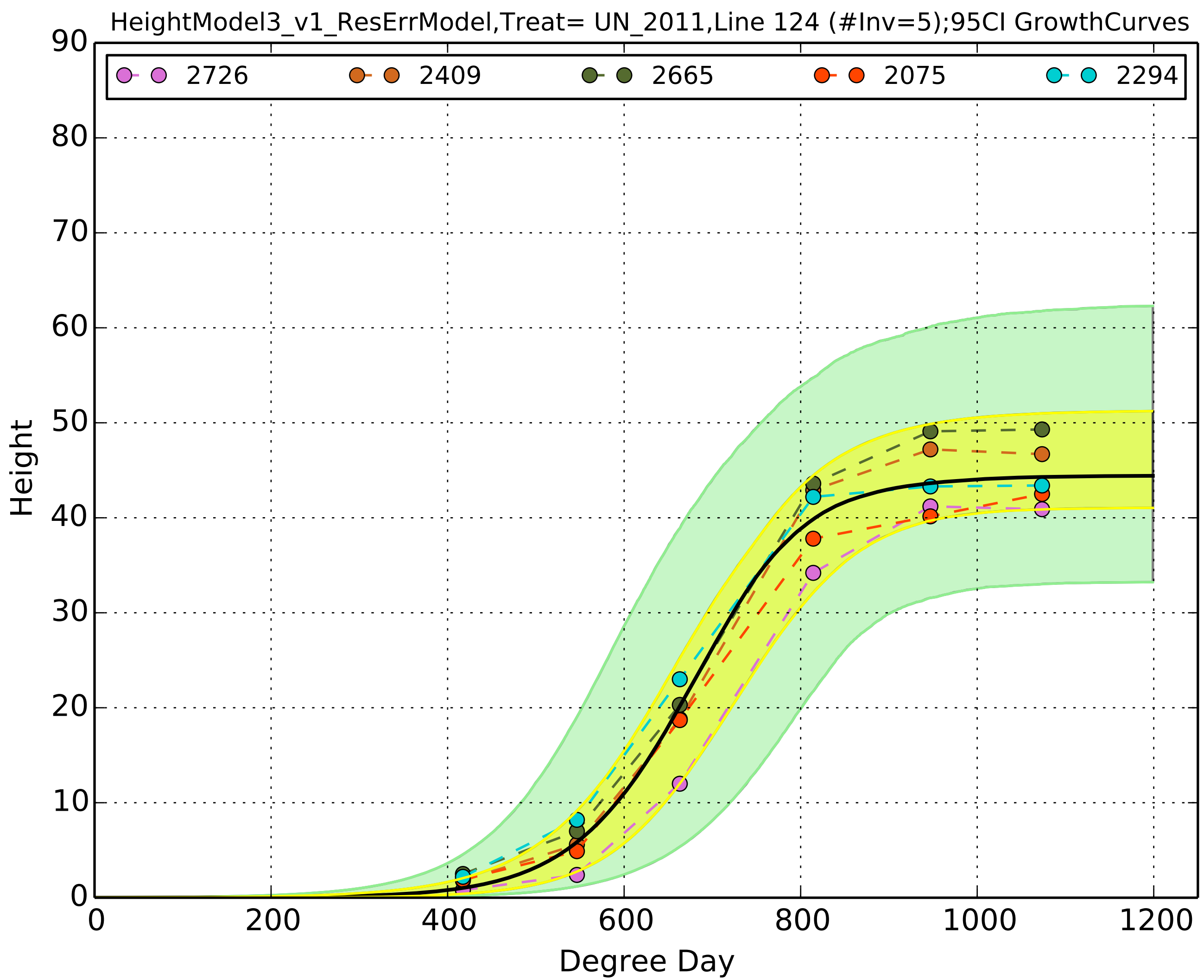

HeightModel3_v1_ResErrModel,Treat= UN_2011,Line 124 (#Inv=5);95CI GrowthCurves
2726
2409
2665
2075
2294
Height
Degree Day
0
10
20
30
40
50
60
70
80
90
0
200
400
600
800
1000
1200

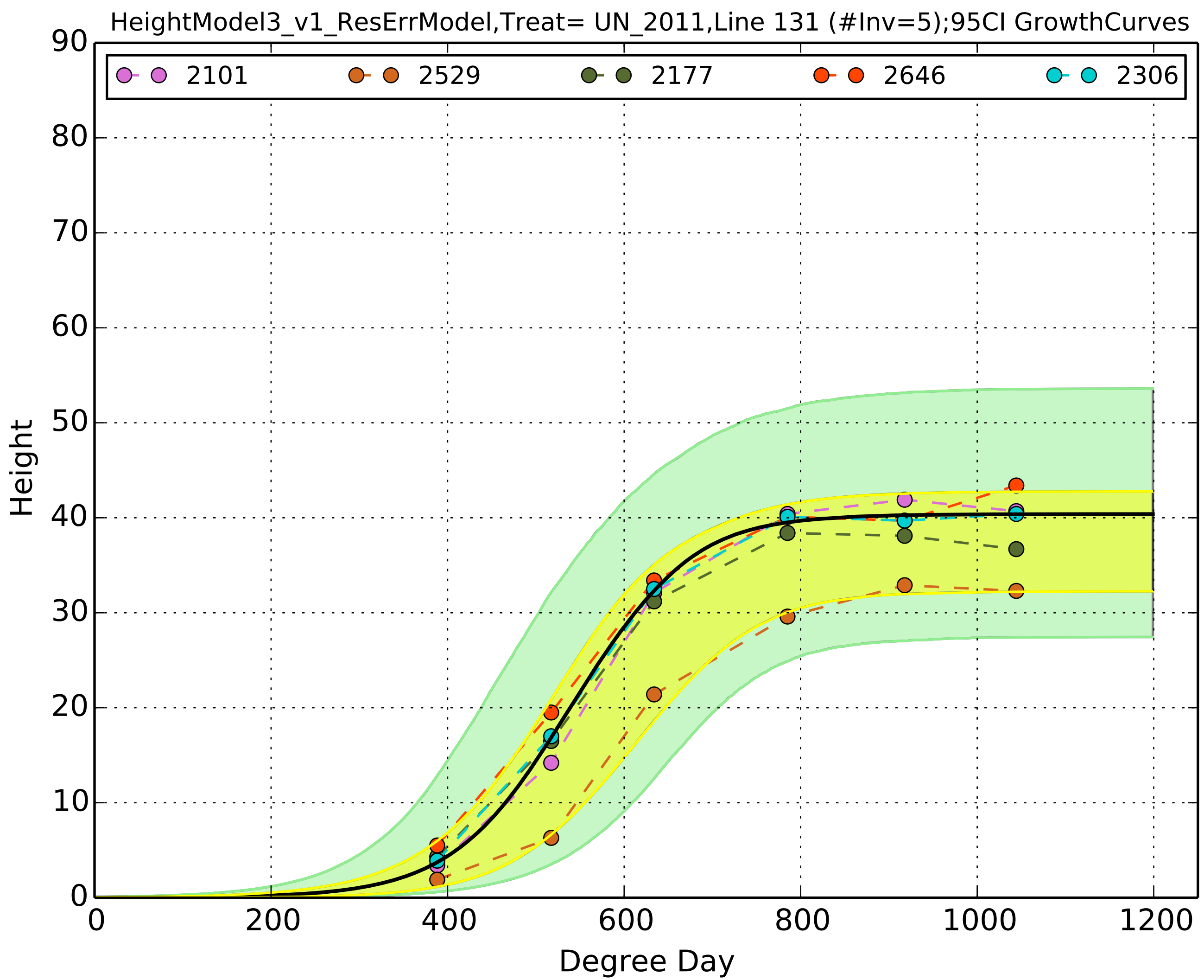
HeightModel3_v1_ResErrModel,Treat= UN_2011,Line 131 (#Inv=5);95CI GrowthCurves
2101
2529
2177
2646
2306
Height
Degree Day
0
10
20
30
40
50
60
70
80
90
0
200
400
600
800
1000
1200

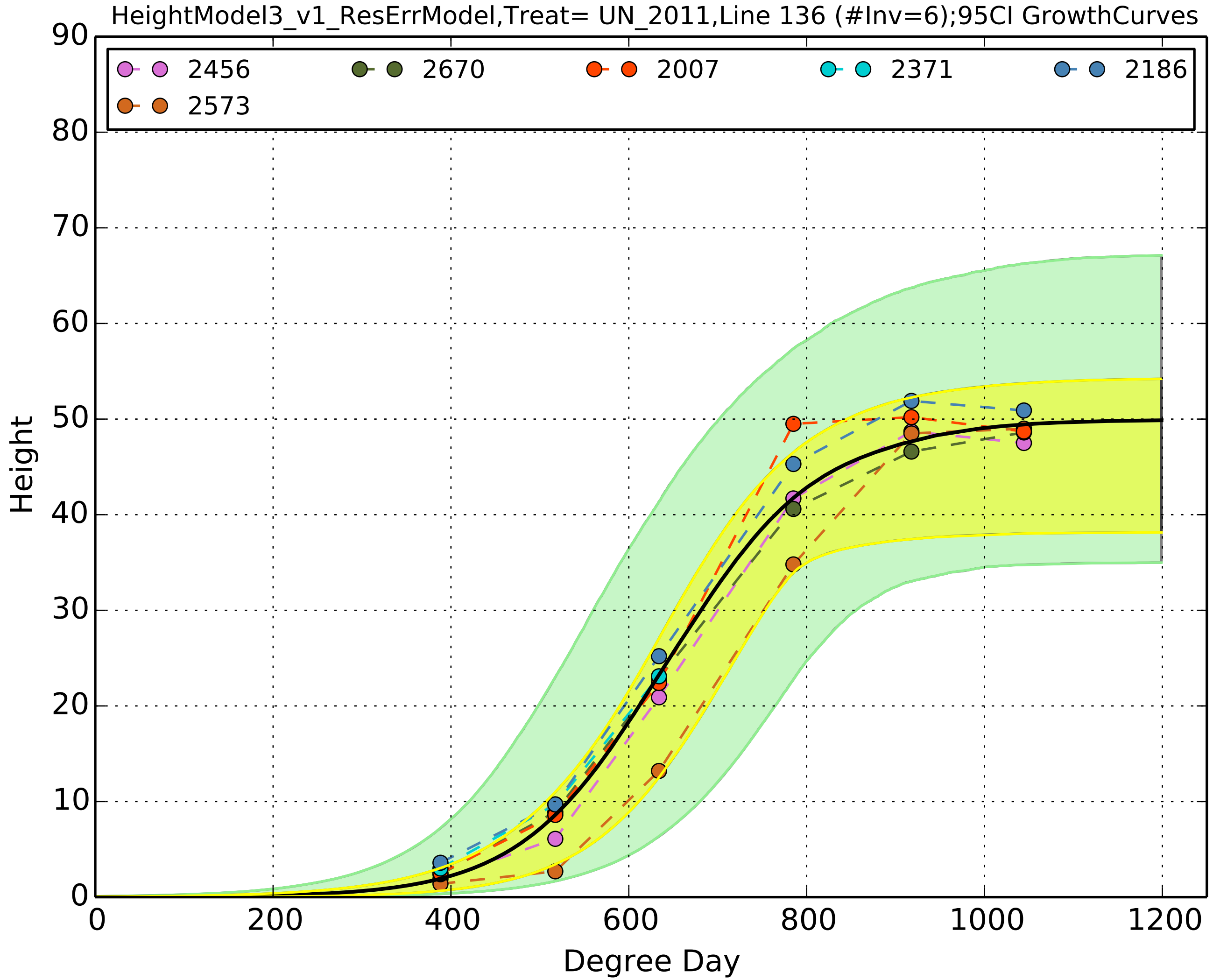

HeightModel3_v1_ResErrModel,Treat= UN_2011,Line 136 (#Inv=6);95CI GrowthCurves
2456
2670
2007
2371
2186
2573
Height
Degree Day
0
10
20
30
40
50
60
70
80
90
0
200
400
600
800
1000
1200

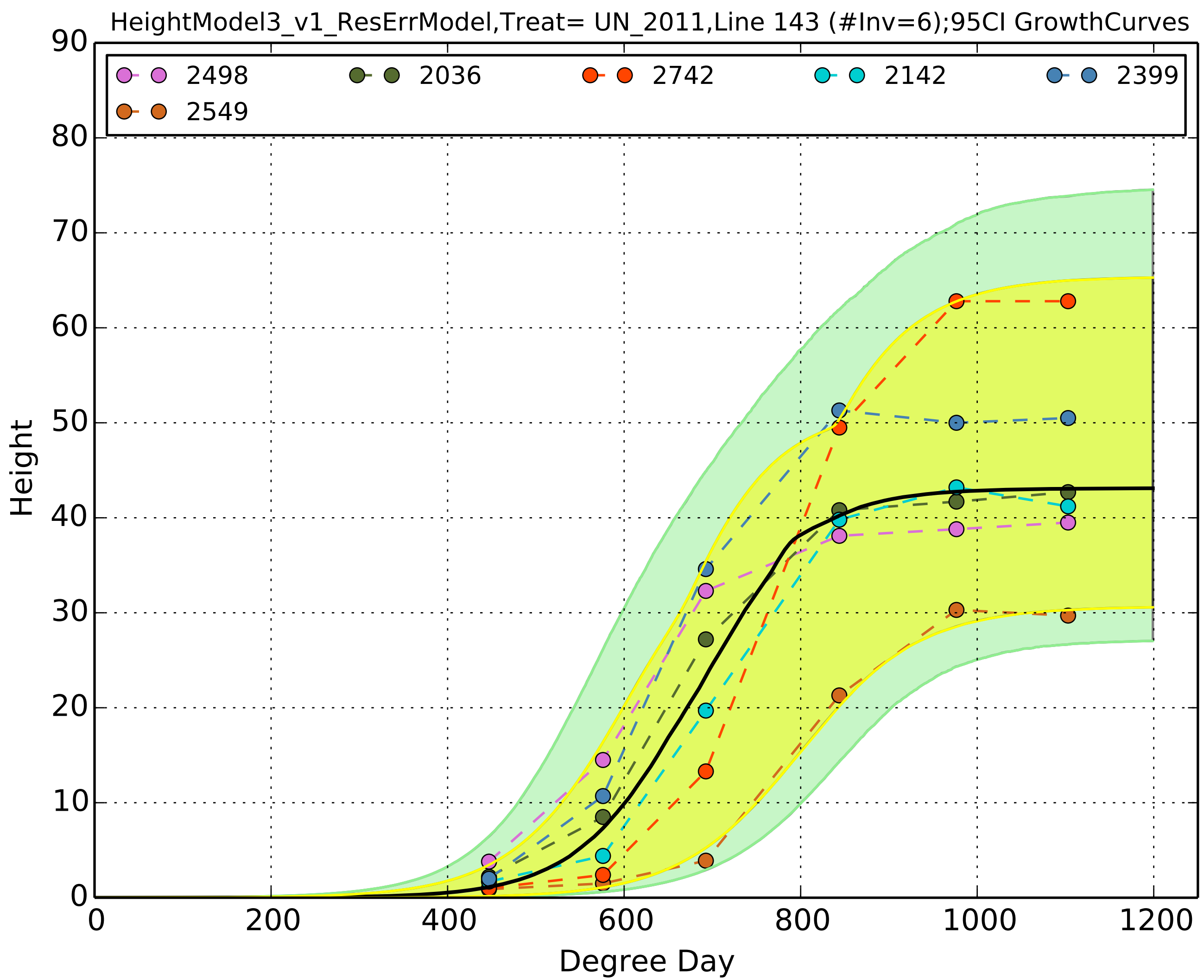
HeightModel3_v1_ResErrModel,Treat= UN_2011,Line 143 (#Inv=6);95CI GrowthCurves
2498
2036
2742
2142
2399
2549
Height
Degree Day
0
10
20
30
40
50
60
70
80
90
0
200
400
600
800
1000
1200

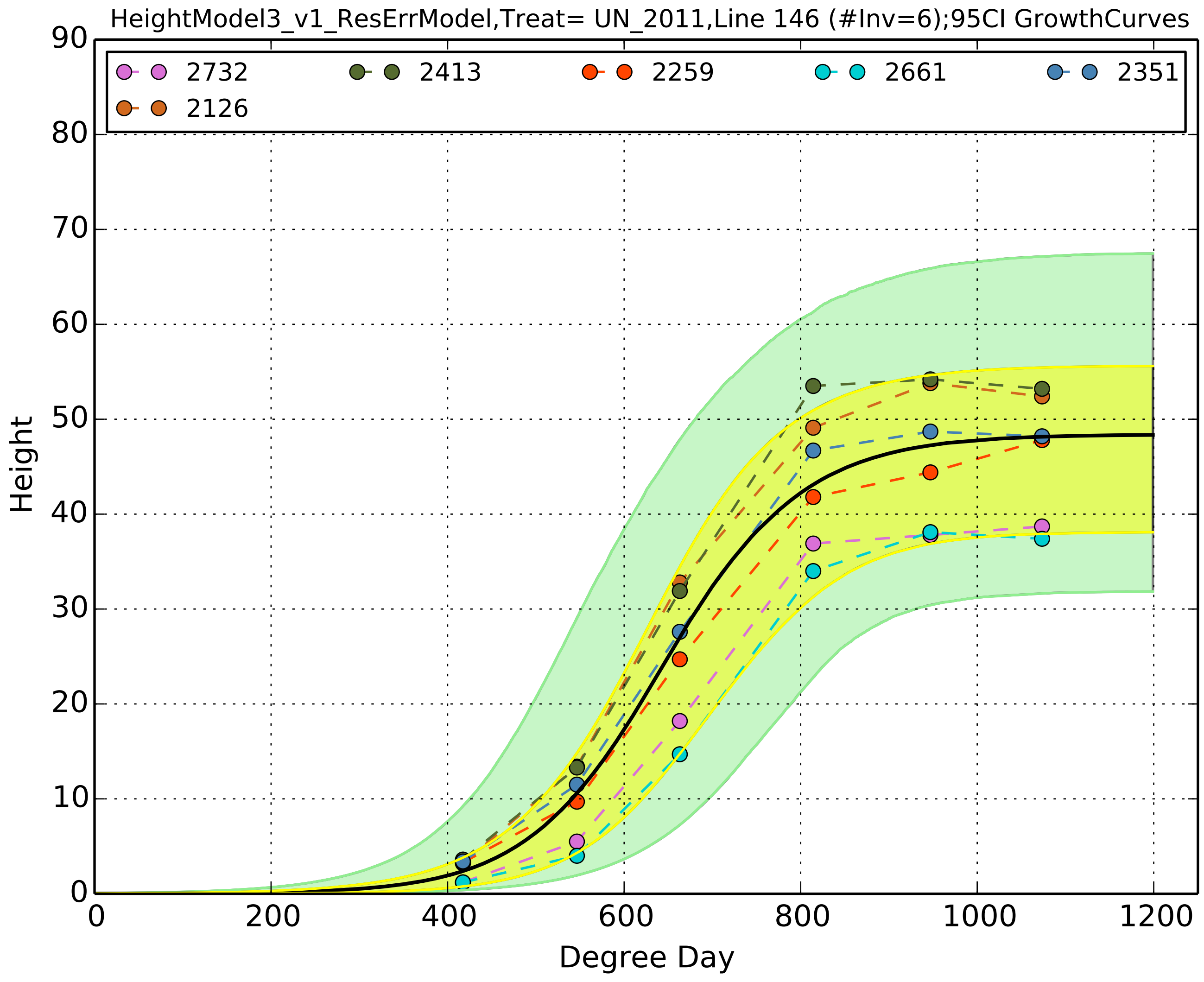
HeightModel3_v1_ResErrModel,Treat= UN_2011,Line 146 (#Inv=6);95CI GrowthCurves
2732
2413
2259
2661
2351
2126
Height
Degree Day
0
10
20
30
40
50
60
70
80
90
0
200
400
600
800
1000
1200

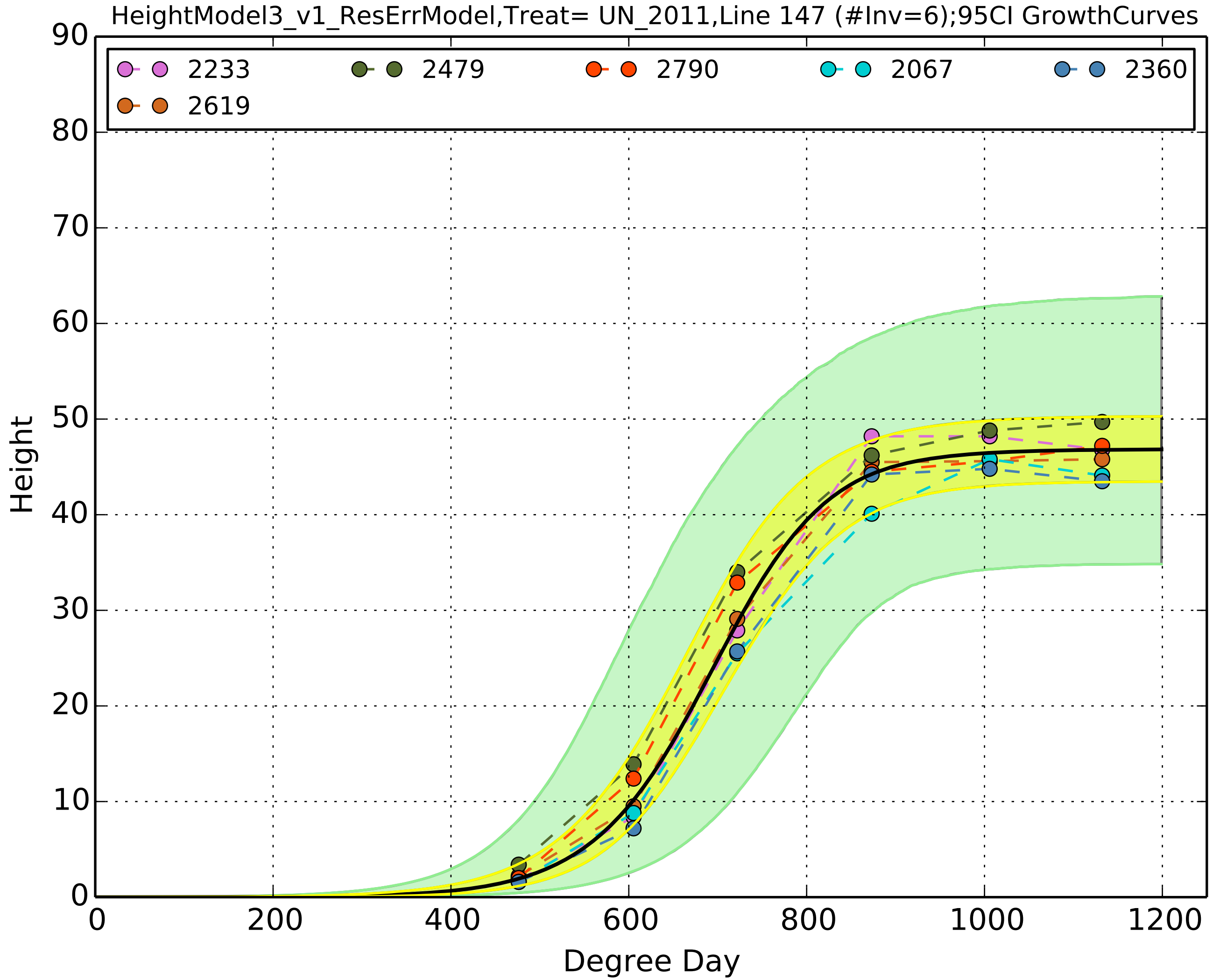

HeightModel3_v1_ResErrModel,Treat= UN_2011,Line 147 (#Inv=6);95CI GrowthCurves
2233
2479
2790
2067
2360
2619
Height
Degree Day
0
10
20
30
40
50
60
70
80
90
0
200
400
600
800
1000
1200

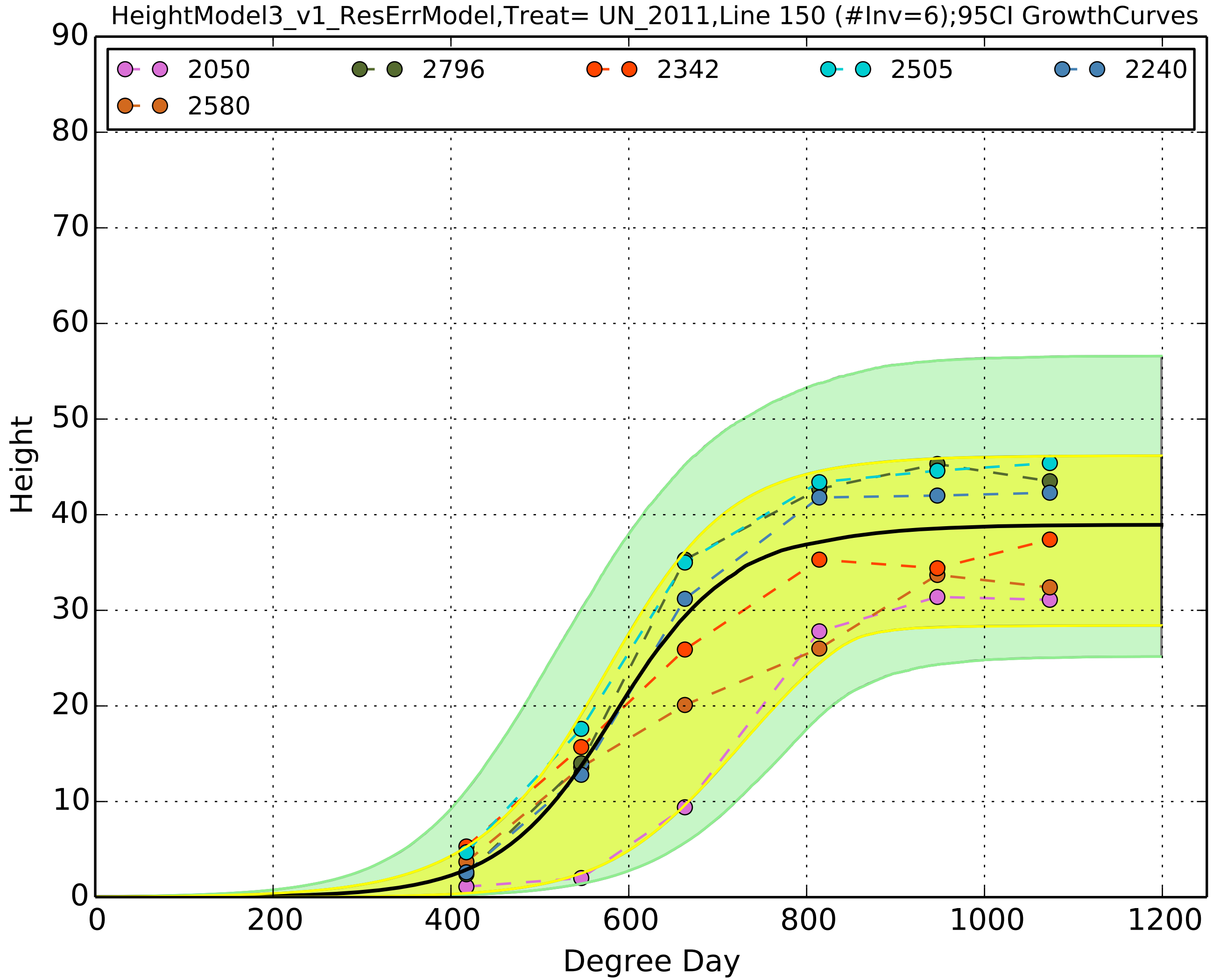

HeightModel3_v1_ResErrModel,Treat= UN_2011,Line 150 (#Inv=6);95CI GrowthCurves
2050
2796
2342
2505
2240
2580
Height
Degree Day
0
10
20
30
40
50
60
70
80
90
0
200
400
600
800
1000
1200

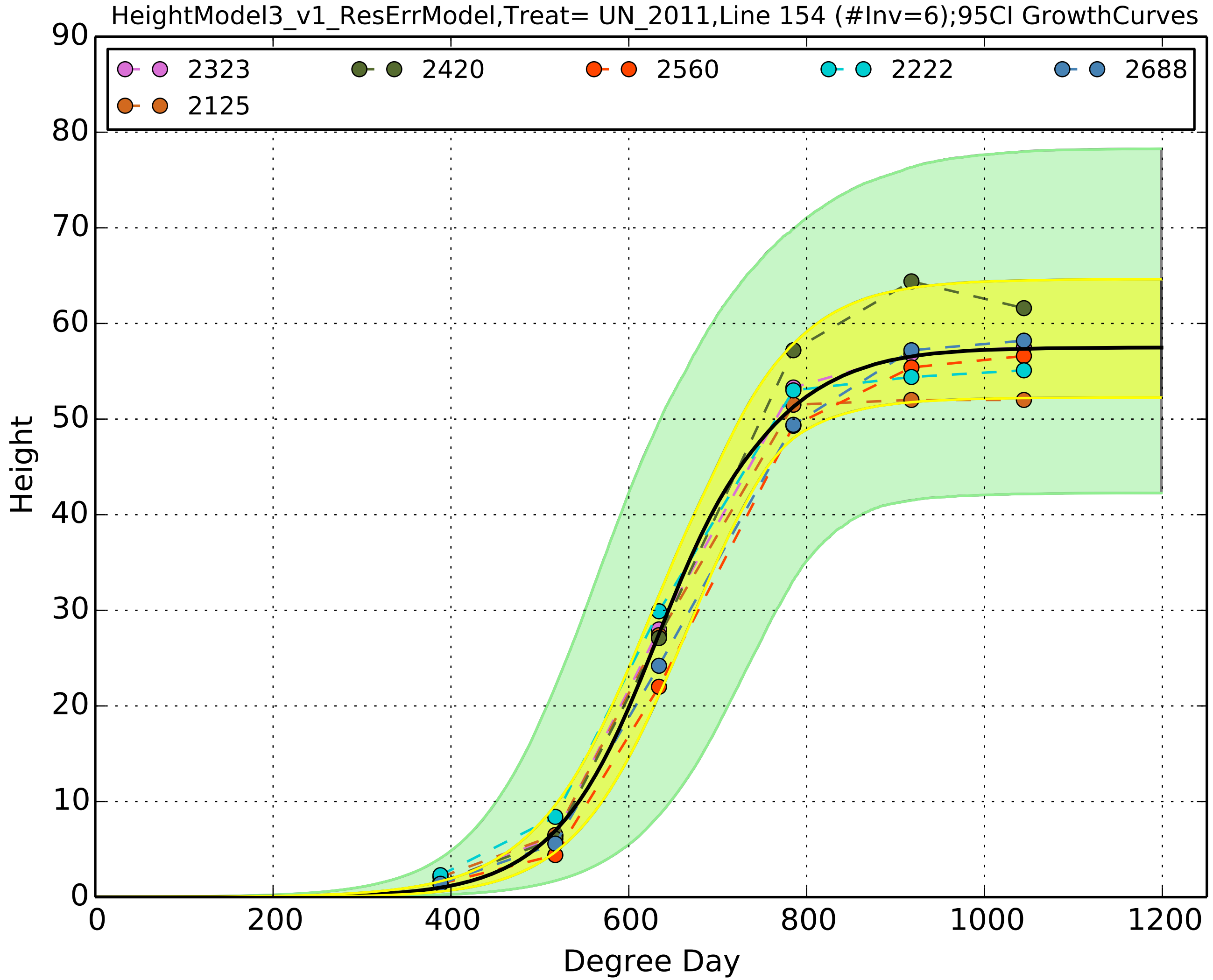

HeightModel3_v1_ResErrModel,Treat= UN_2011,Line 154 (#Inv=6);95CI GrowthCurves
2323
2420
2560
2222
2688
2125
Height
Degree Day
0
10
20
30
40
50
60
70
80
90
0
200
400
600
800
1000
1200

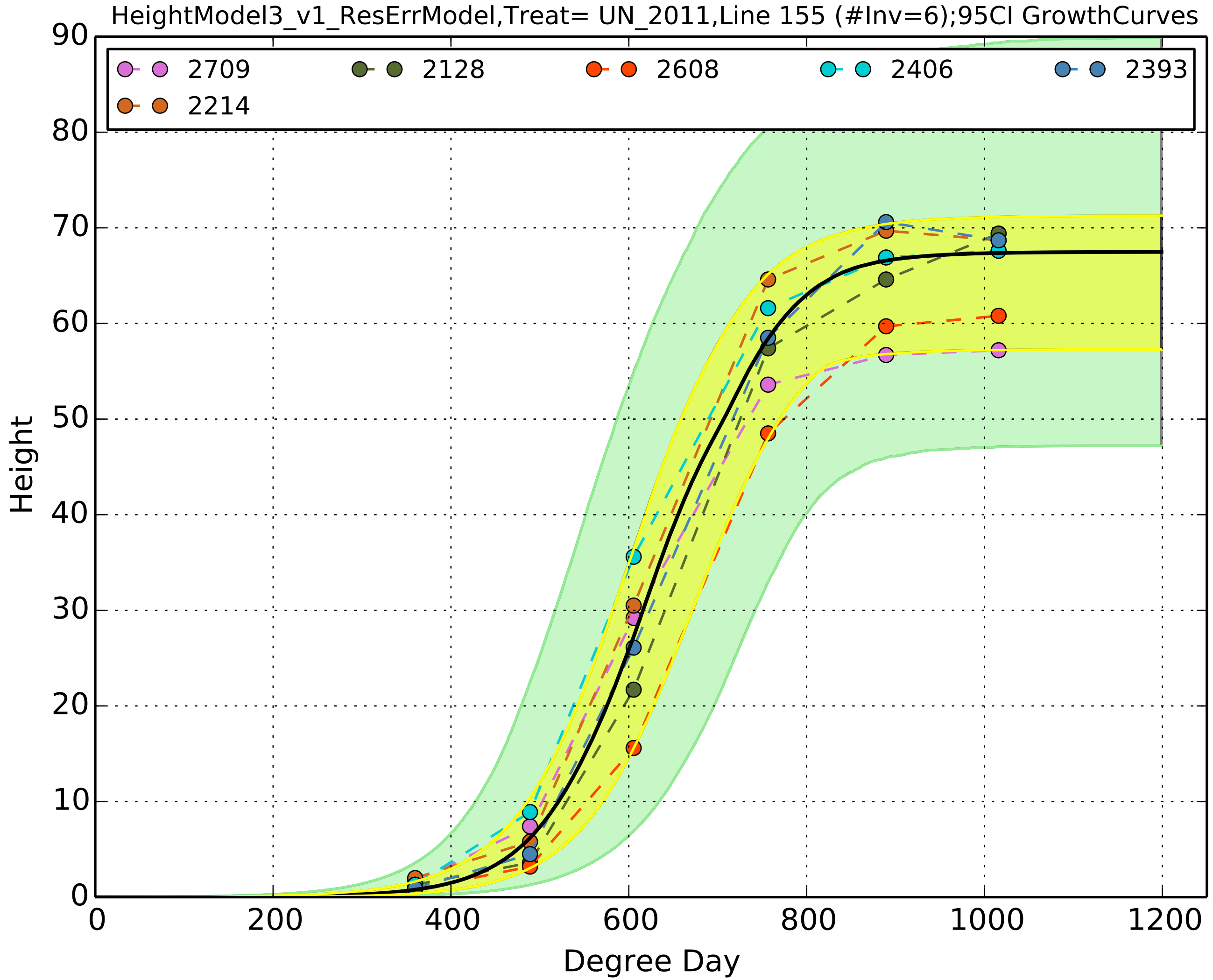
HeightModel3_v1_ResErrModel,Treat= UN_2011,Line 155 (#Inv=6);95CI GrowthCurves
2709
2128
2608
2406
2393
2214
Height
Degree Day
0
10
20
30
40
50
60
70
80
90
0
200
400
600
800
1000
1200

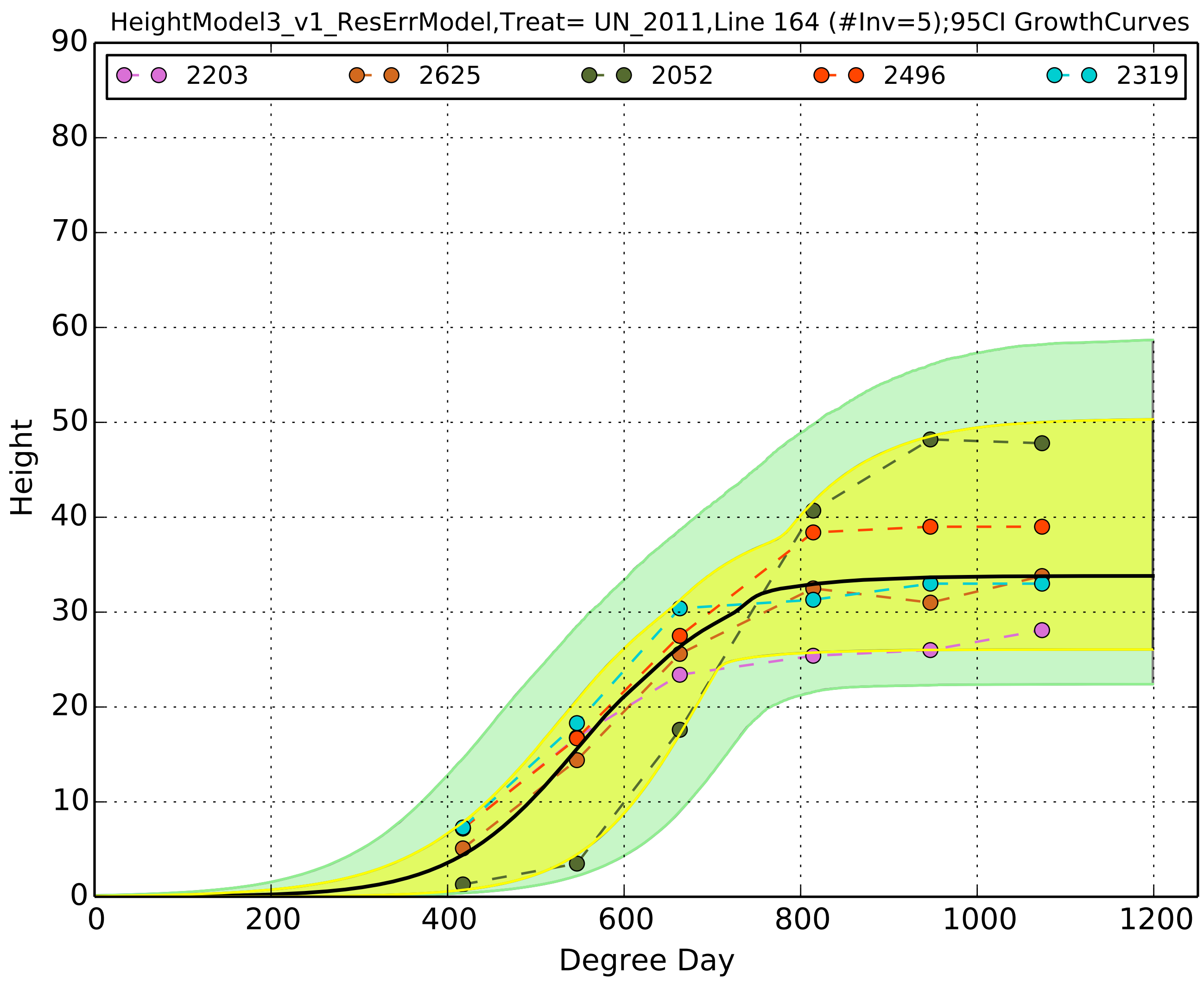
HeightModel3_v1_ResErrModel,Treat= UN_2011,Line 164 (#Inv=5);95CI GrowthCurves
2203
2625
2052
2496
2319
Height
Degree Day
0
10
20
30
40
50
60
70
80
90
0
200
400
600
800
1000
1200

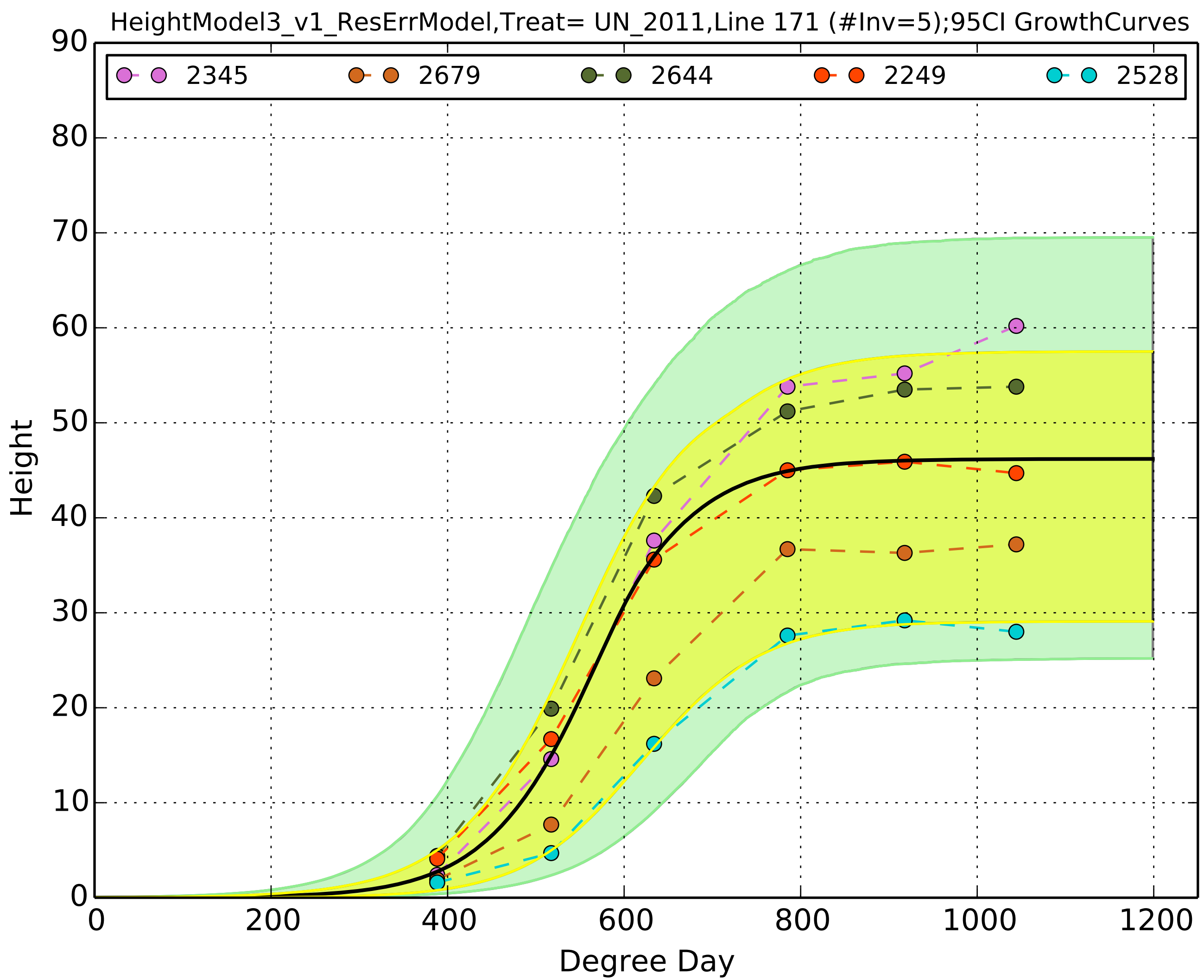

HeightModel3_v1_ResErrModel,Treat= UN_2011,Line 171 (#Inv=5);95CI GrowthCurves
2345
2679
2644
2249
2528
Height
Degree Day
0
10
20
30
40
50
60
70
80
90
0
200
400
600
800
1000
1200

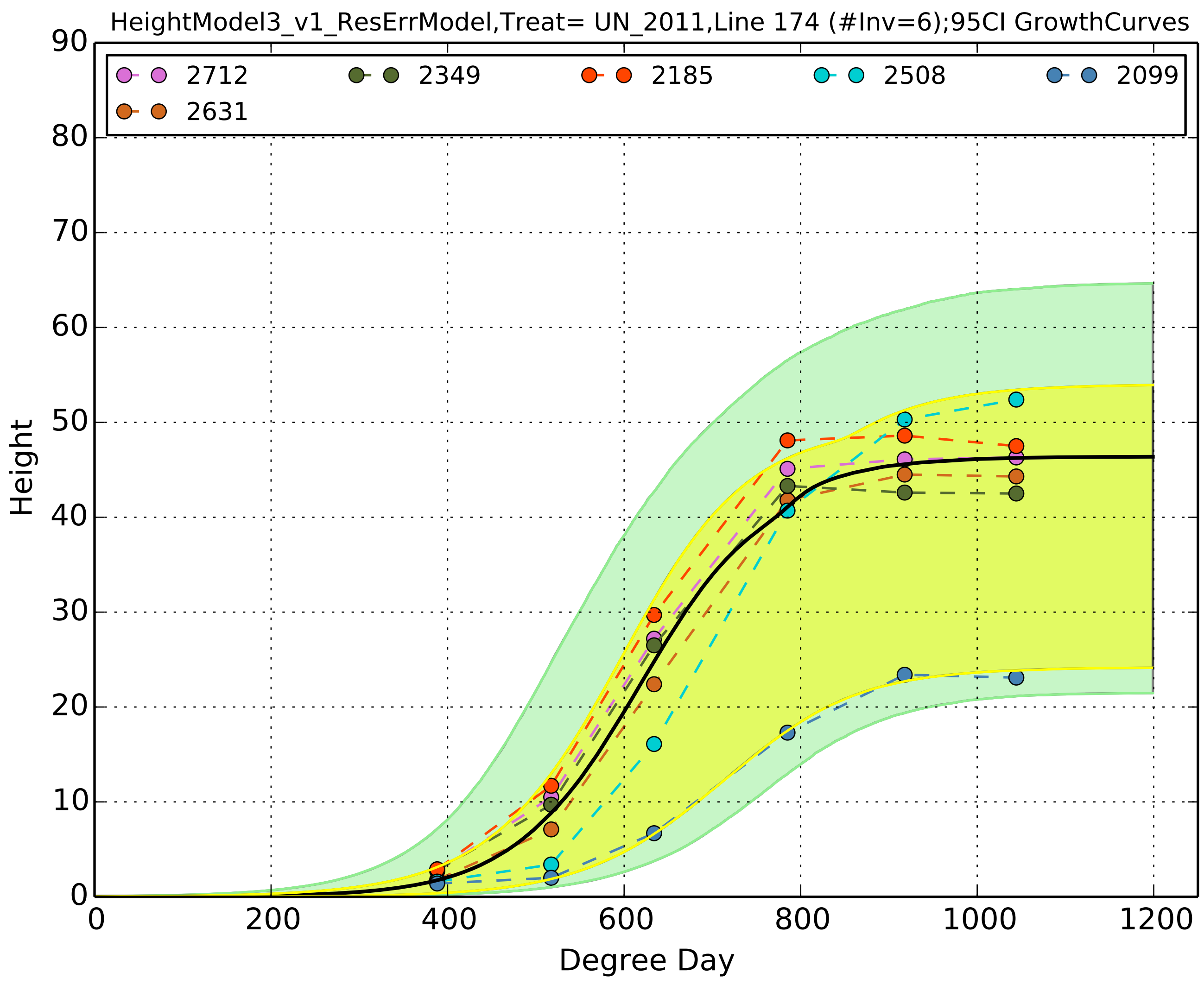

HeightModel3_v1_ResErrModel,Treat= UN_2011,Line 174 (#Inv=6);95CI GrowthCurves
2712
2349
2185
2508
2099
2631
Height
Degree Day
0
10
20
30
40
50
60
70
80
90
0
200
400
600
800
1000
1200

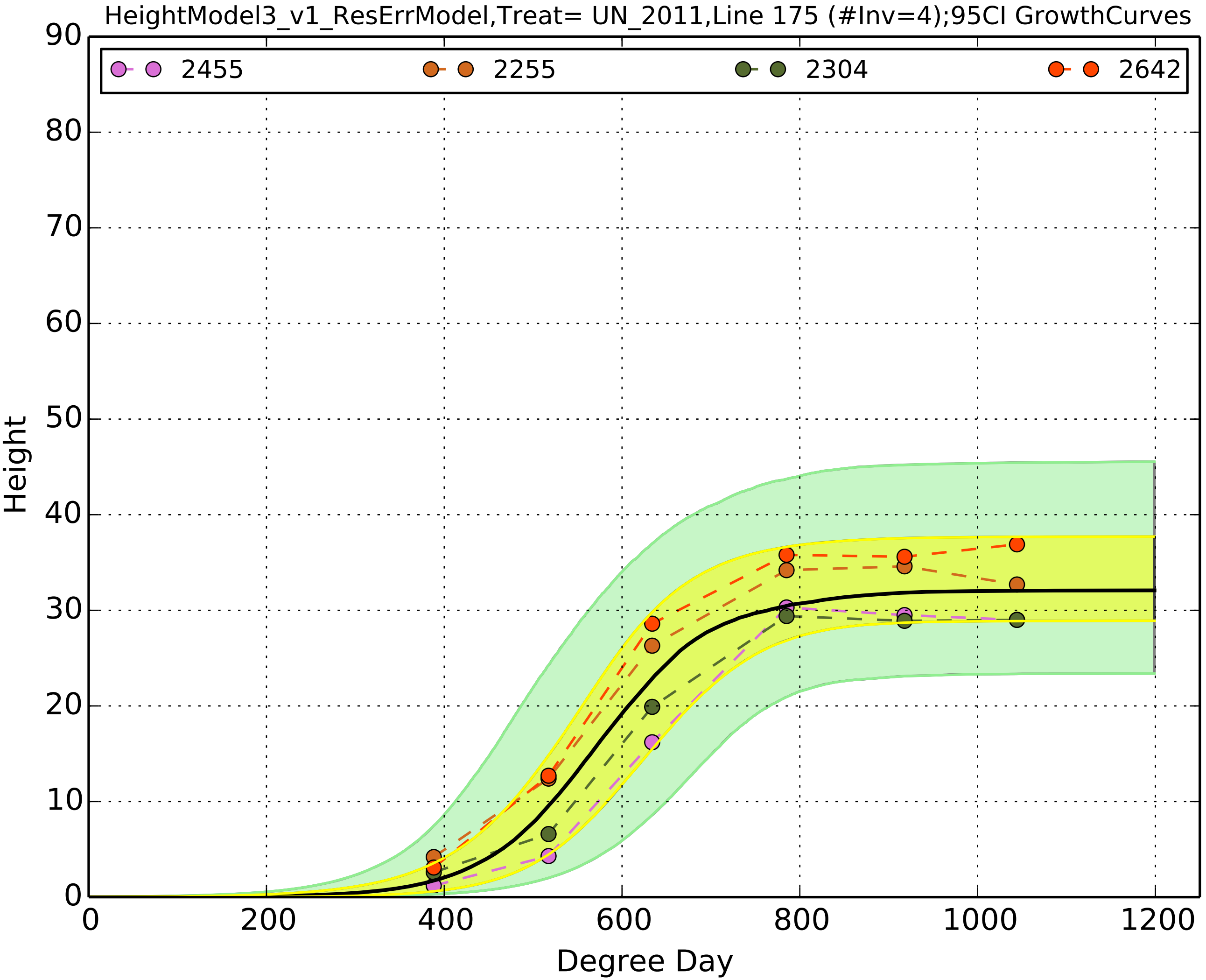
HeightModel3_v1_ResErrModel,Treat= UN_2011,Line 175 (#Inv=4);95CI GrowthCurves
2455
2255
2304
2642
Height
Degree Day
0
10
20
30
40
50
60
70
80
90
0
200
400
600
800
1000
1200

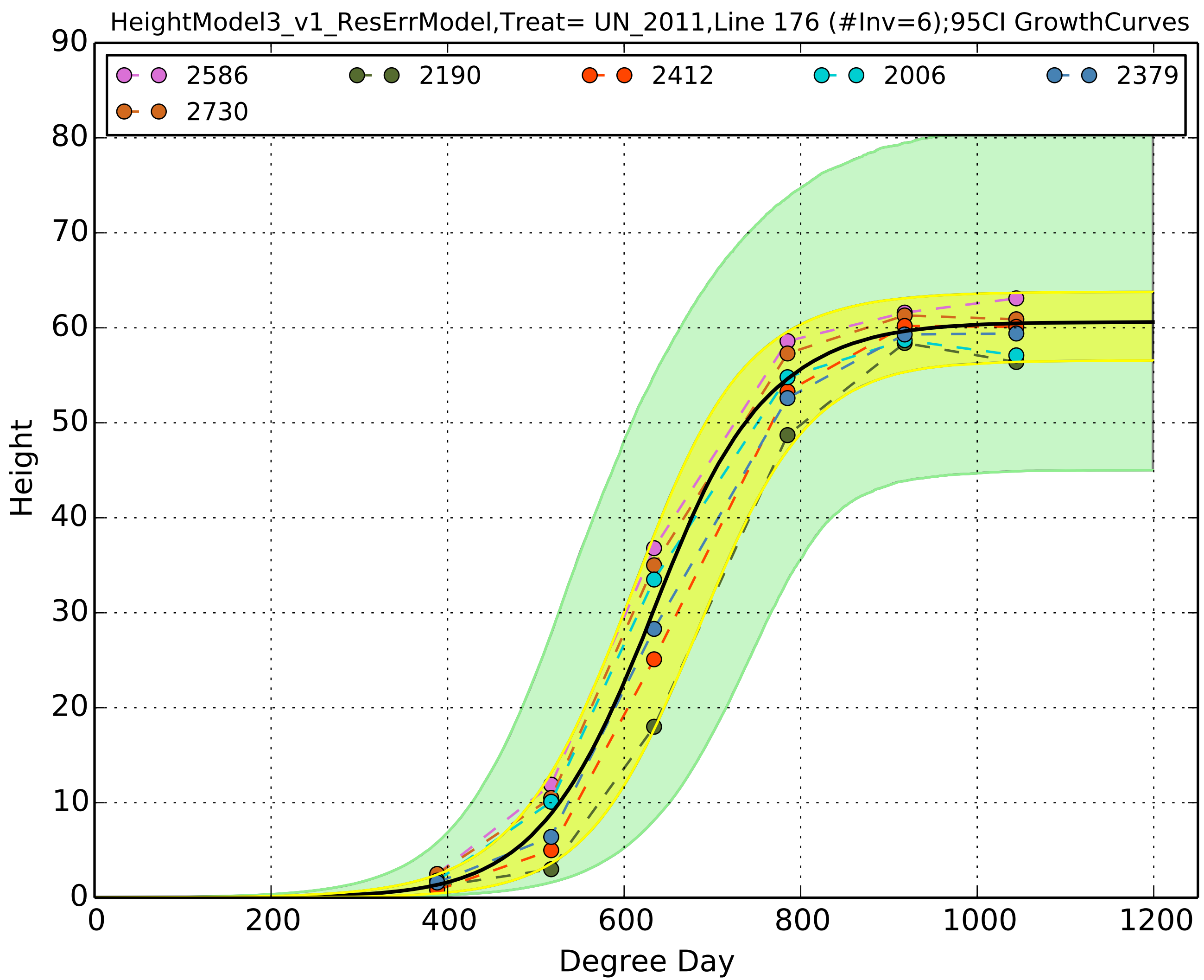

HeightModel3_v1_ResErrModel,Treat= UN_2011,Line 176 (#Inv=6);95CI GrowthCurves
2586
2190
2412
2006
2379
2730
Height
Degree Day
0
10
20
30
40
50
60
70
80
90
0
200
400
600
800
1000
1200

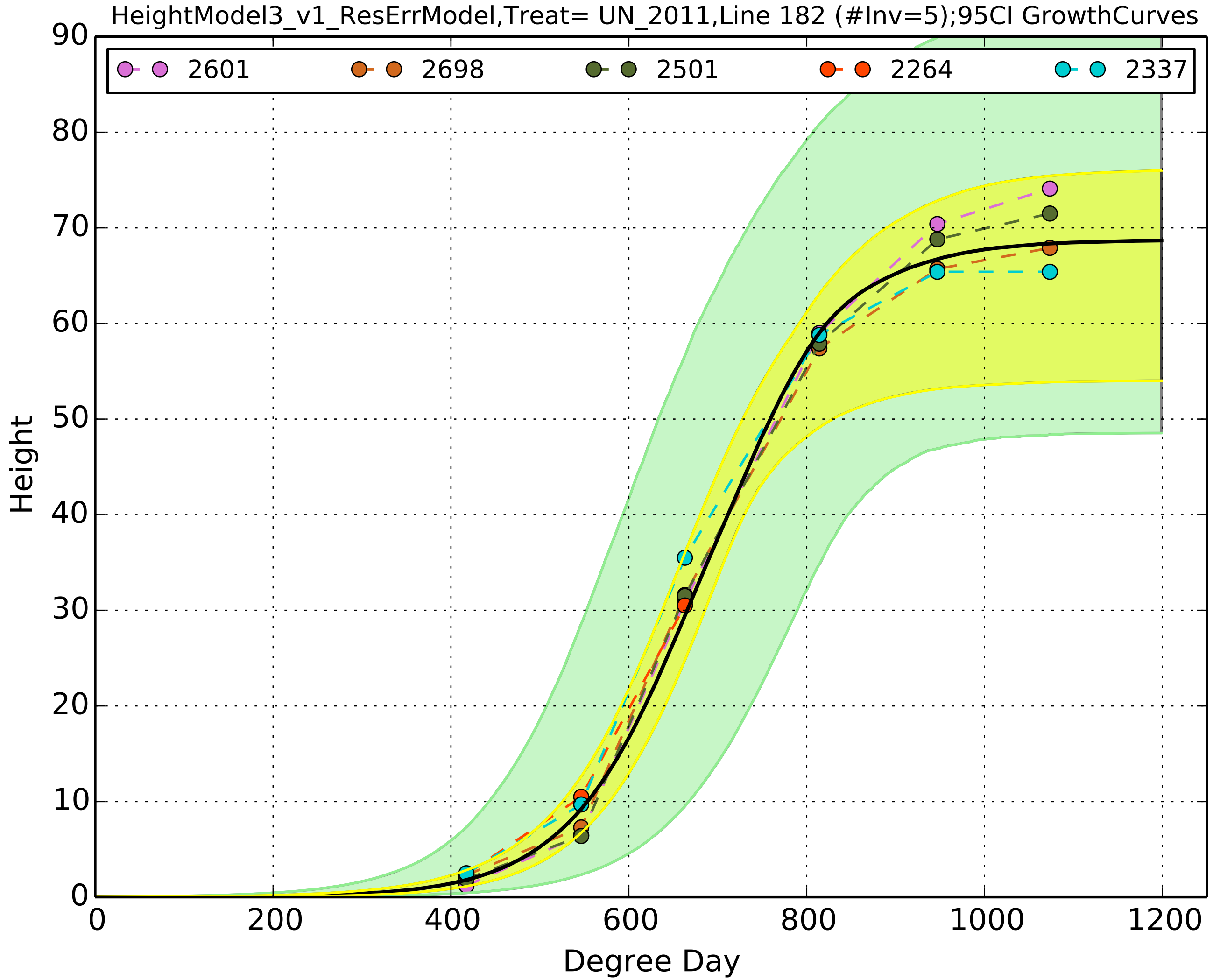
HeightModel3_v1_ResErrModel,Treat= UN_2011,Line 182 (#Inv=5);95CI GrowthCurves
2601
2698
2501
2264
2337
Height
Degree Day
0
10
20
30
40
50
60
70
80
90
0
200
400
600
800
1000
1200

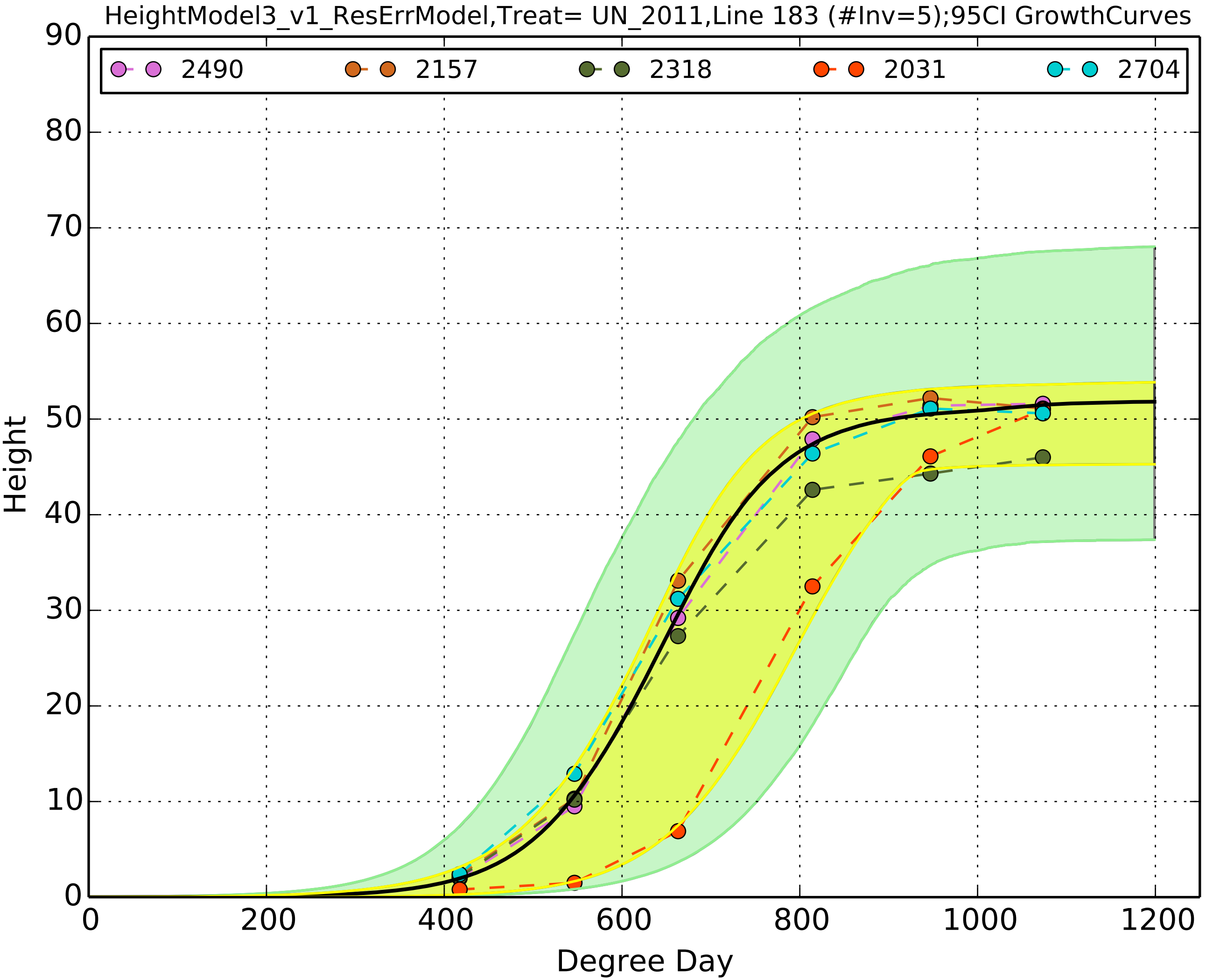
HeightModel3_v1_ResErrModel,Treat= UN_2011,Line 183 (#Inv=5);95CI GrowthCurves
2490
2157
2318
2031
2704
Height
Degree Day
0
10
20
30
40
50
60
70
80
90
0
200
400
600
800
1000
1200

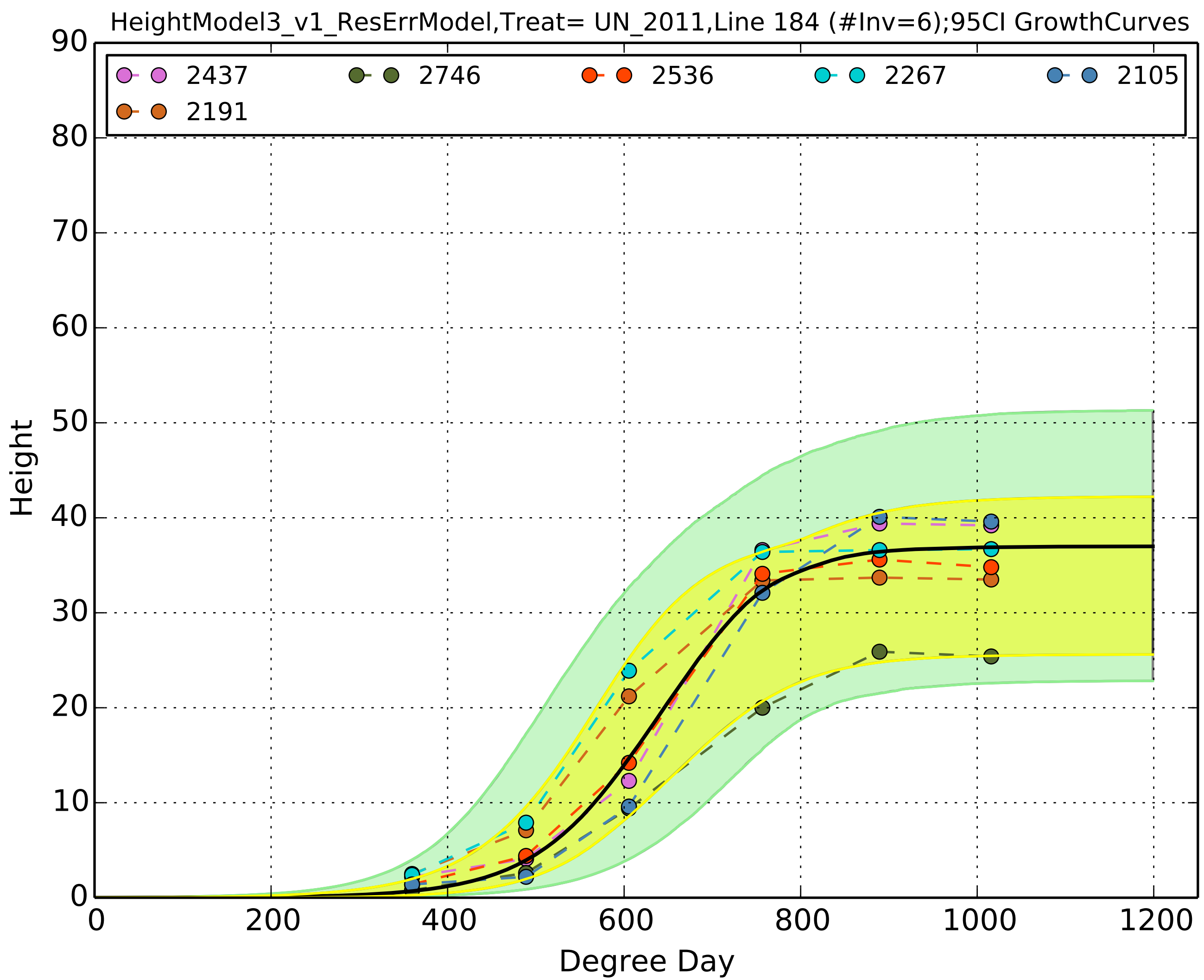
HeightModel3_v1_ResErrModel,Treat= UN_2011,Line 184 (#Inv=6);95CI GrowthCurves
2437
2746
2536
2267
2105
2191
Height
Degree Day
0
10
20
30
40
50
60
70
80
90
0
200
400
600
800
1000
1200

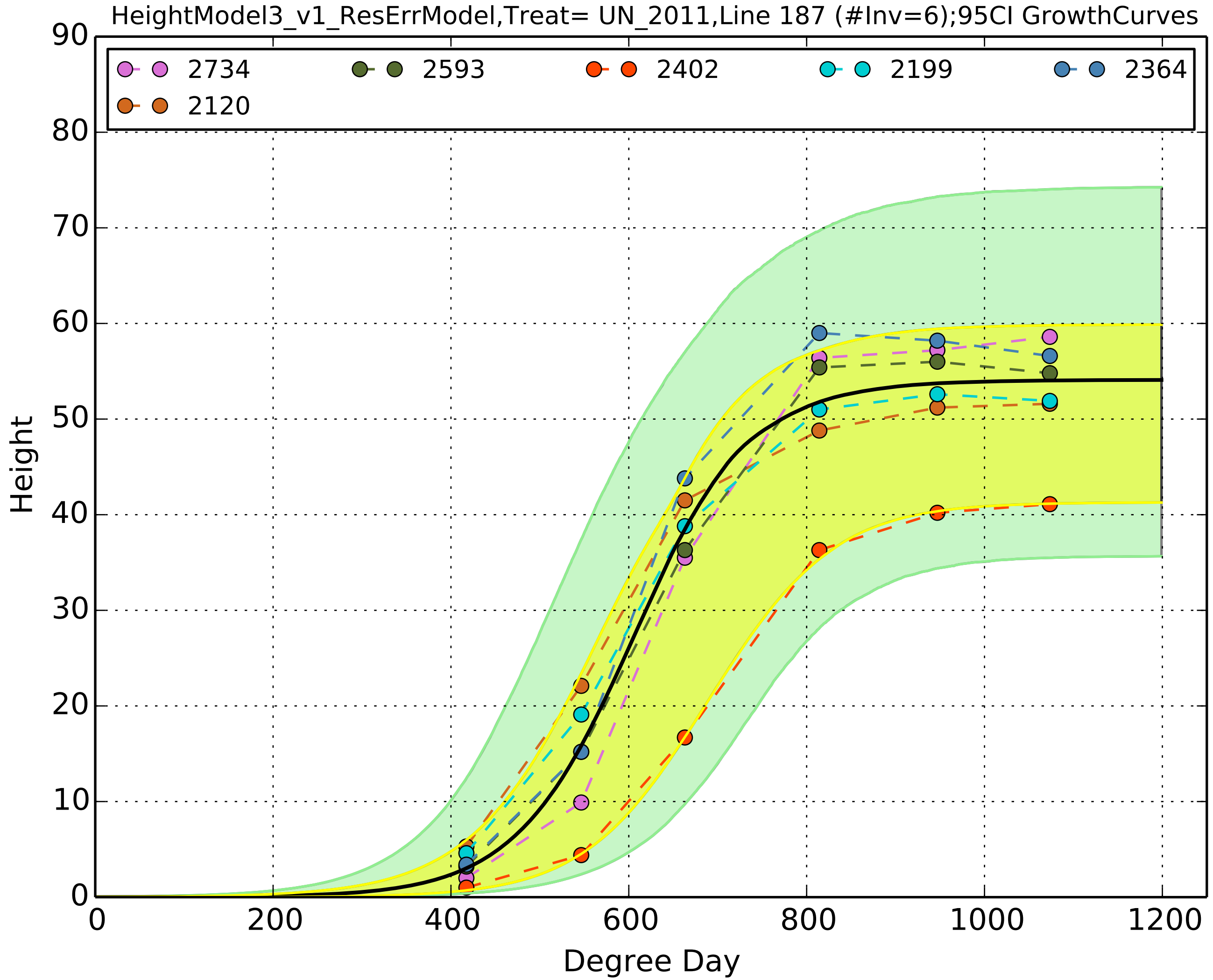

HeightModel3_v1_ResErrModel,Treat= UN_2011,Line 187 (#Inv=6);95CI GrowthCurves
2734
2593
2402
2199
2364
2120
Height
Degree Day
0
10
20
30
40
50
60
70
80
90
0
200
400
600
800
1000
1200

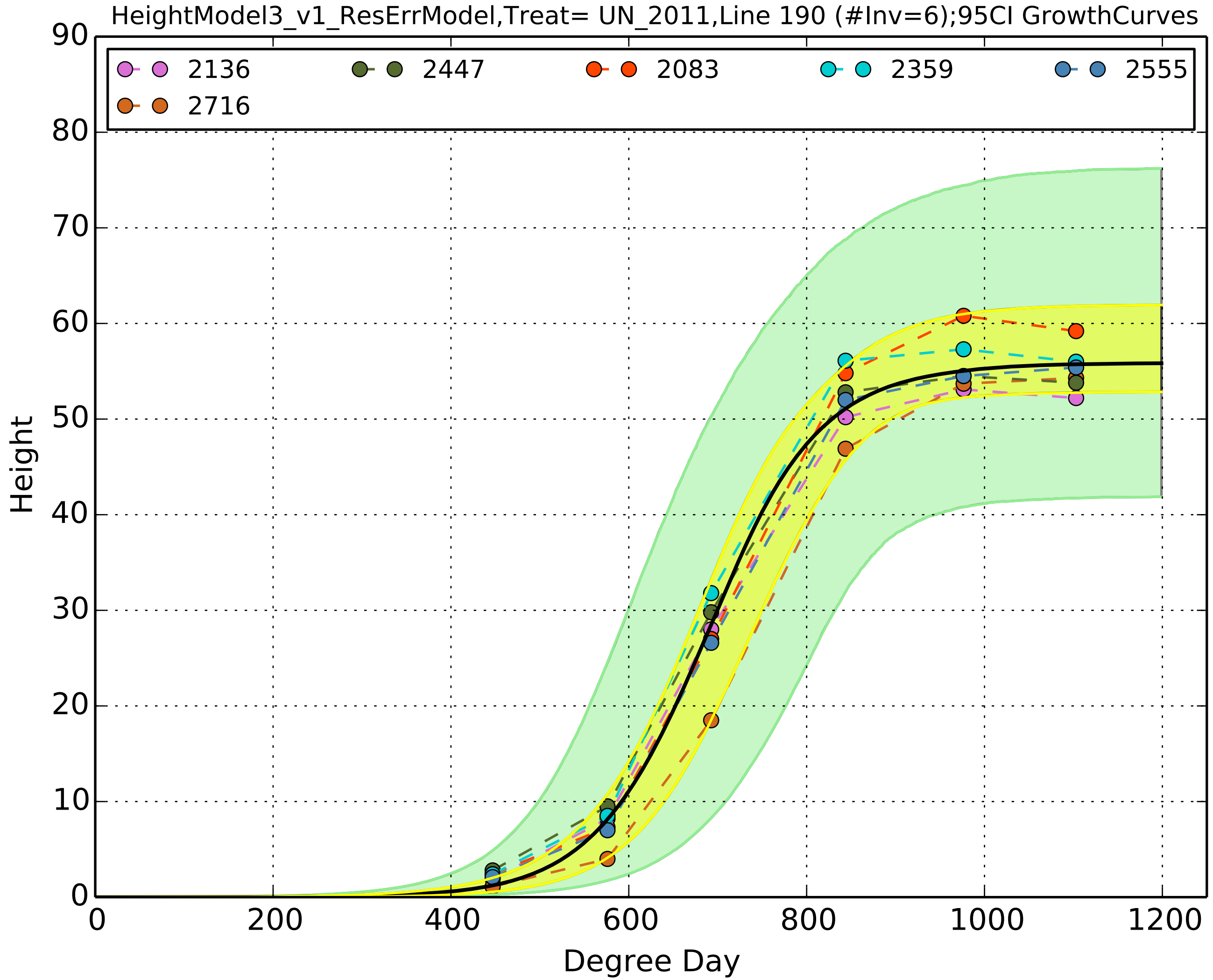

HeightModel3_v1_ResErrModel,Treat= UN_2011,Line 190 (#Inv=6);95CI GrowthCurves
2136
2447
2083
2359
2555
2716
Height
Degree Day
0
10
20
30
40
50
60
70
80
90
0
200
400
600
800
1000
1200

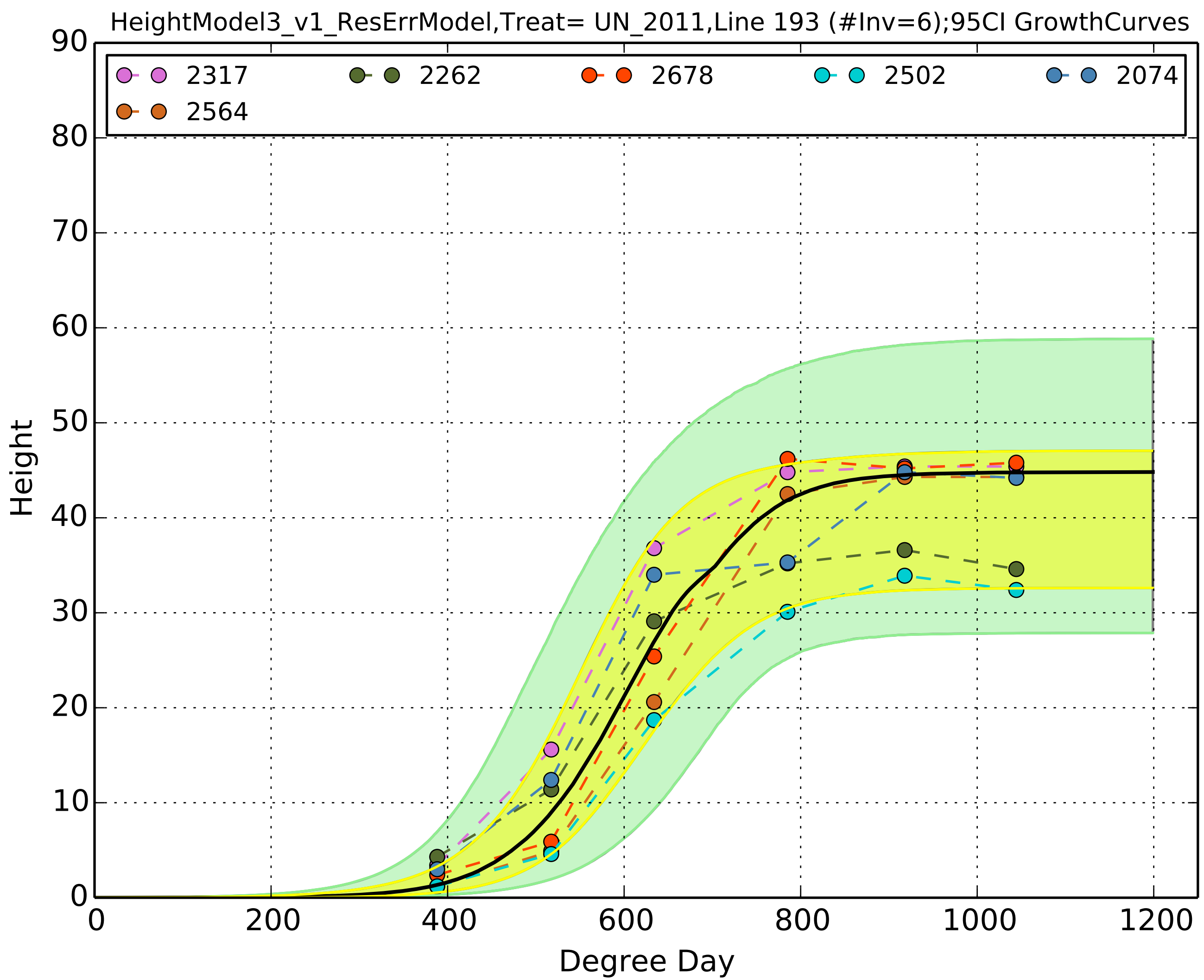

HeightModel3_v1_ResErrModel,Treat= UN_2011,Line 193 (#Inv=6);95CI GrowthCurves
2317
2262
2678
2502
2074
2564
Height
Degree Day
0
10
20
30
40
50
60
70
80
90
0
200
400
600
800
1000
1200

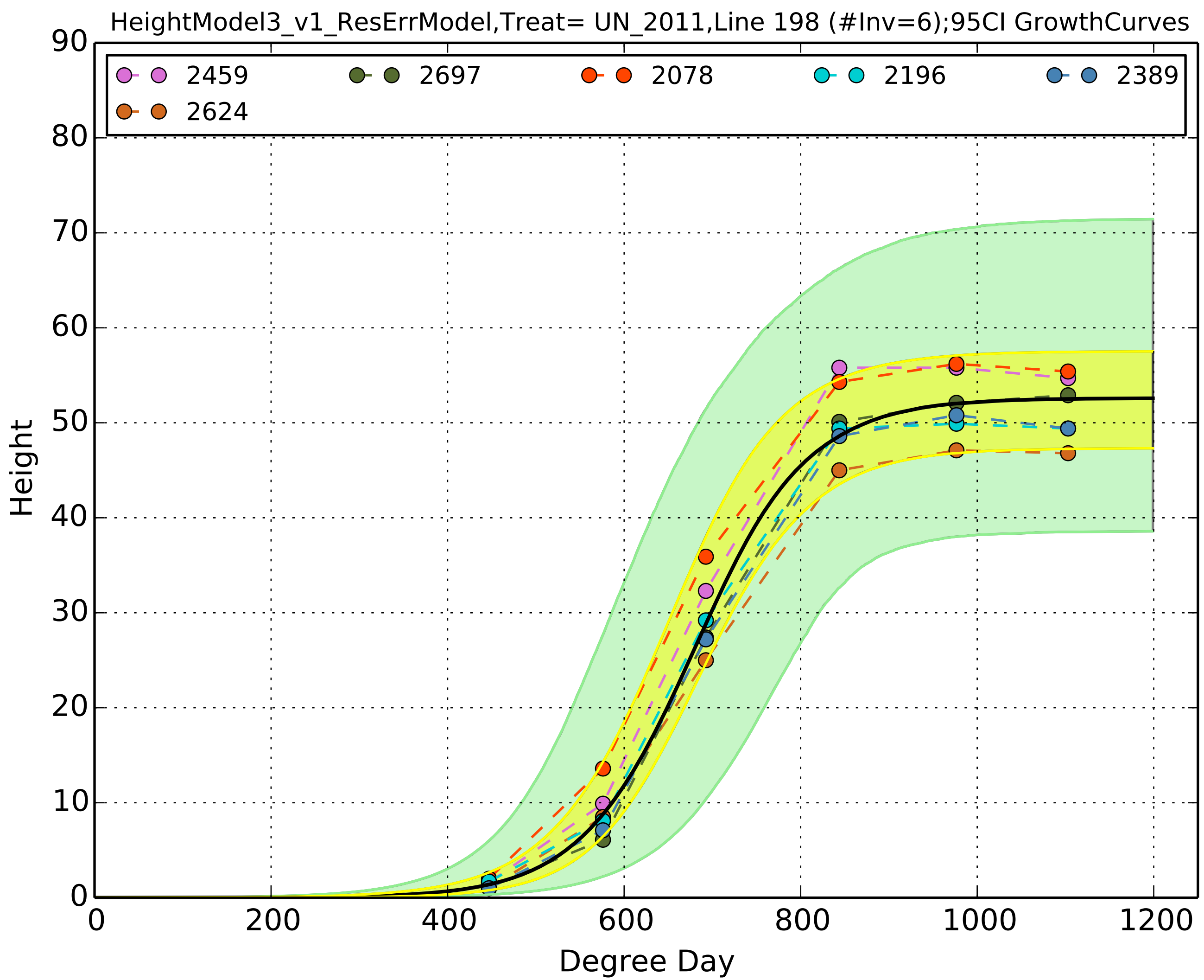

HeightModel3_v1_ResErrModel,Treat= UN_2011,Line 198 (#Inv=6);95CI GrowthCurves
2459
2697
2078
2196
2389
2624
Height
Degree Day
0
10
20
30
40
50
60
70
80
90
0
200
400
600
800
1000
1200

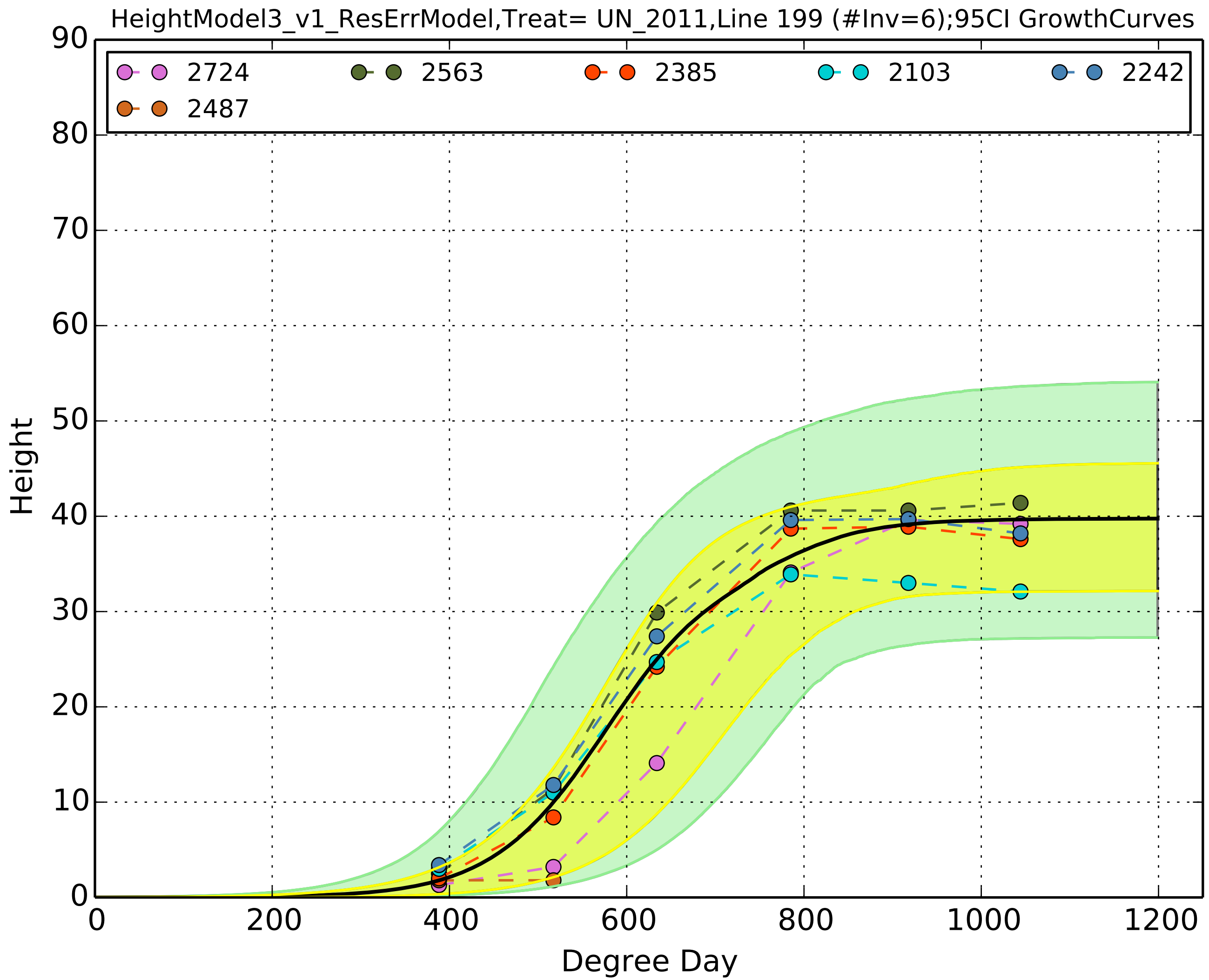

HeightModel3_v1_ResErrModel,Treat= UN_2011,Line 199 (#Inv=6);95CI GrowthCurves
2724
2563
2385
2103
2242
2487
Height
Degree Day
0
10
20
30
40
50
60
70
80
90
0
200
400
600
800
1000
1200

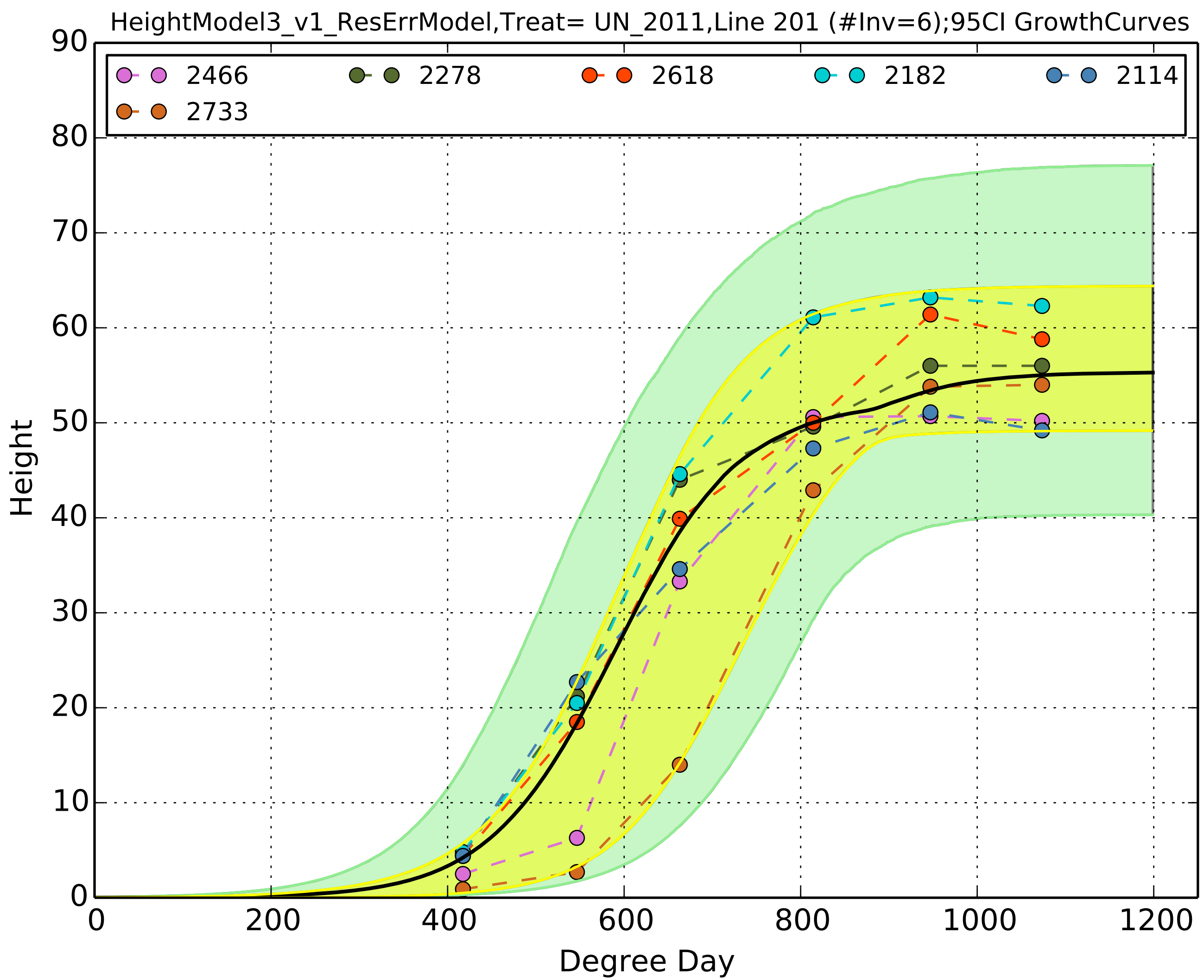

HeightModel3_v1_ResErrModel,Treat= UN_2011,Line 201 (#Inv=6);95CI GrowthCurves
2466
2278
2618
2182
2114
2733
Height
Degree Day
0
10
20
30
40
50
60
70
80
90
0
200
400
600
800
1000
1200

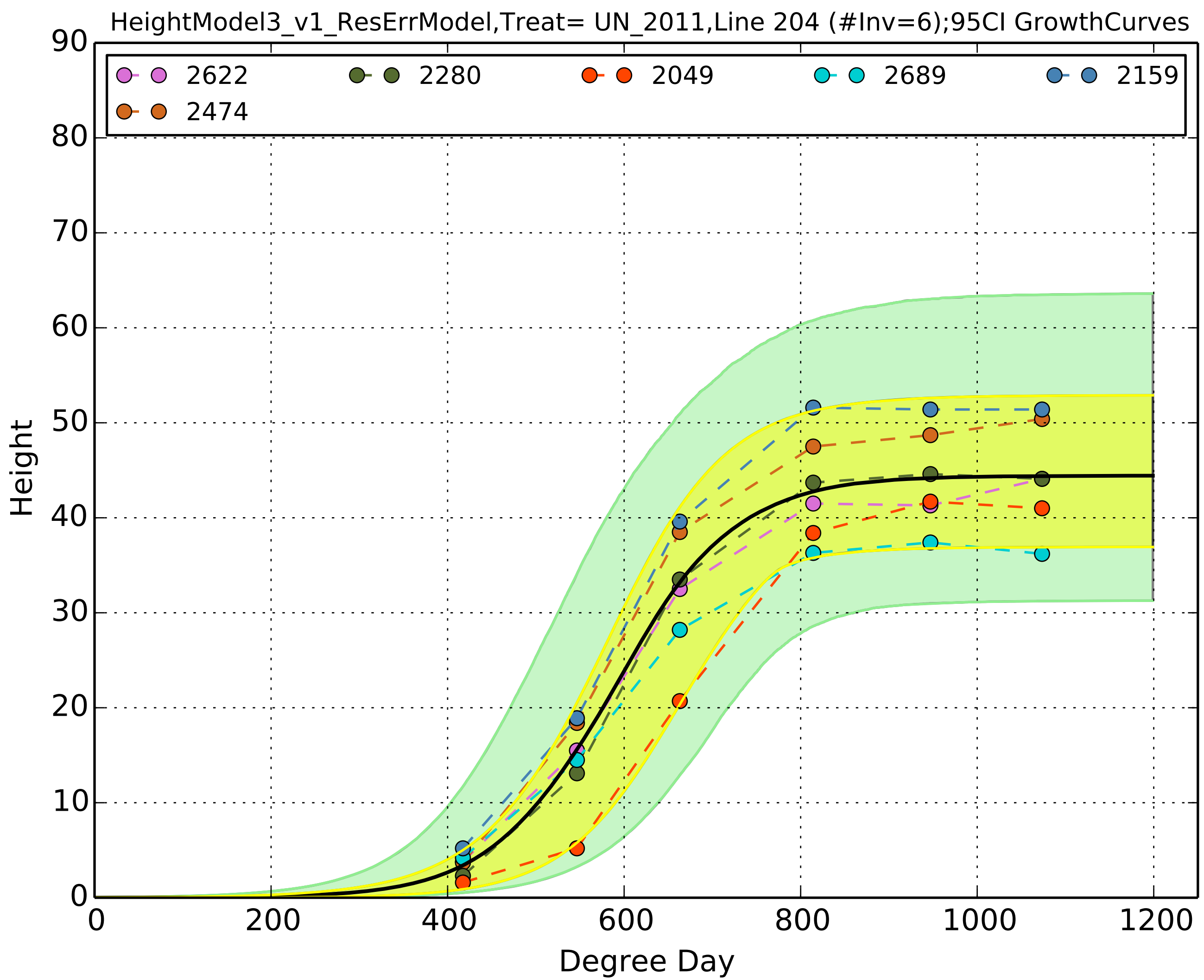

HeightModel3_v1_ResErrModel,Treat= UN_2011,Line 204 (#Inv=6);95CI GrowthCurves
2622
2280
2049
2689
2159
2474
Height
Degree Day
0
10
20
30
40
50
60
70
80
90
0
200
400
600
800
1000
1200

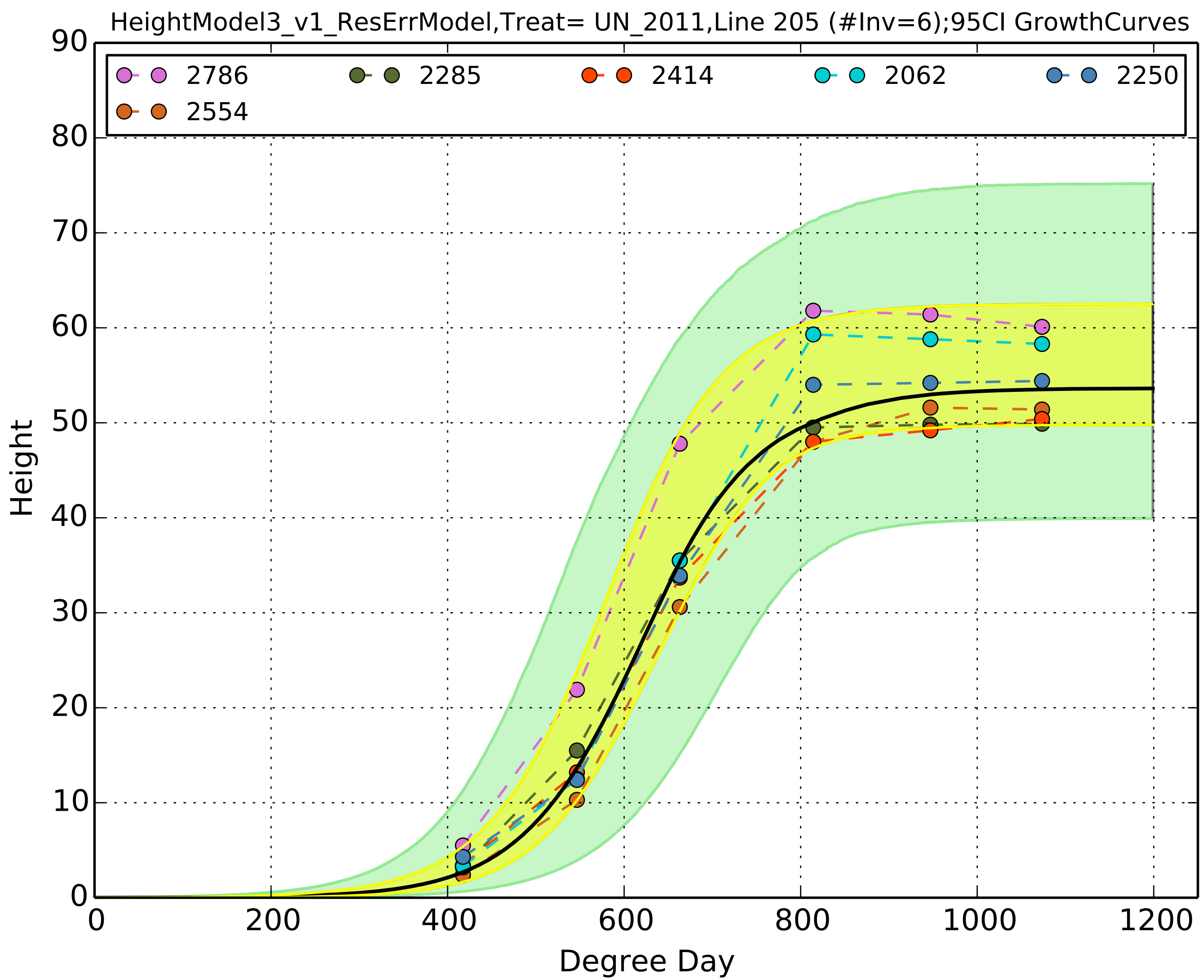
HeightModel3_v1_ResErrModel,Treat= UN_2011,Line 205 (#Inv=6);95CI GrowthCurves
2786
2285
2414
2062
2250
2554
Height
Degree Day
0
10
20
30
40
50
60
70
80
90
0
200
400
600
800
1000
1200

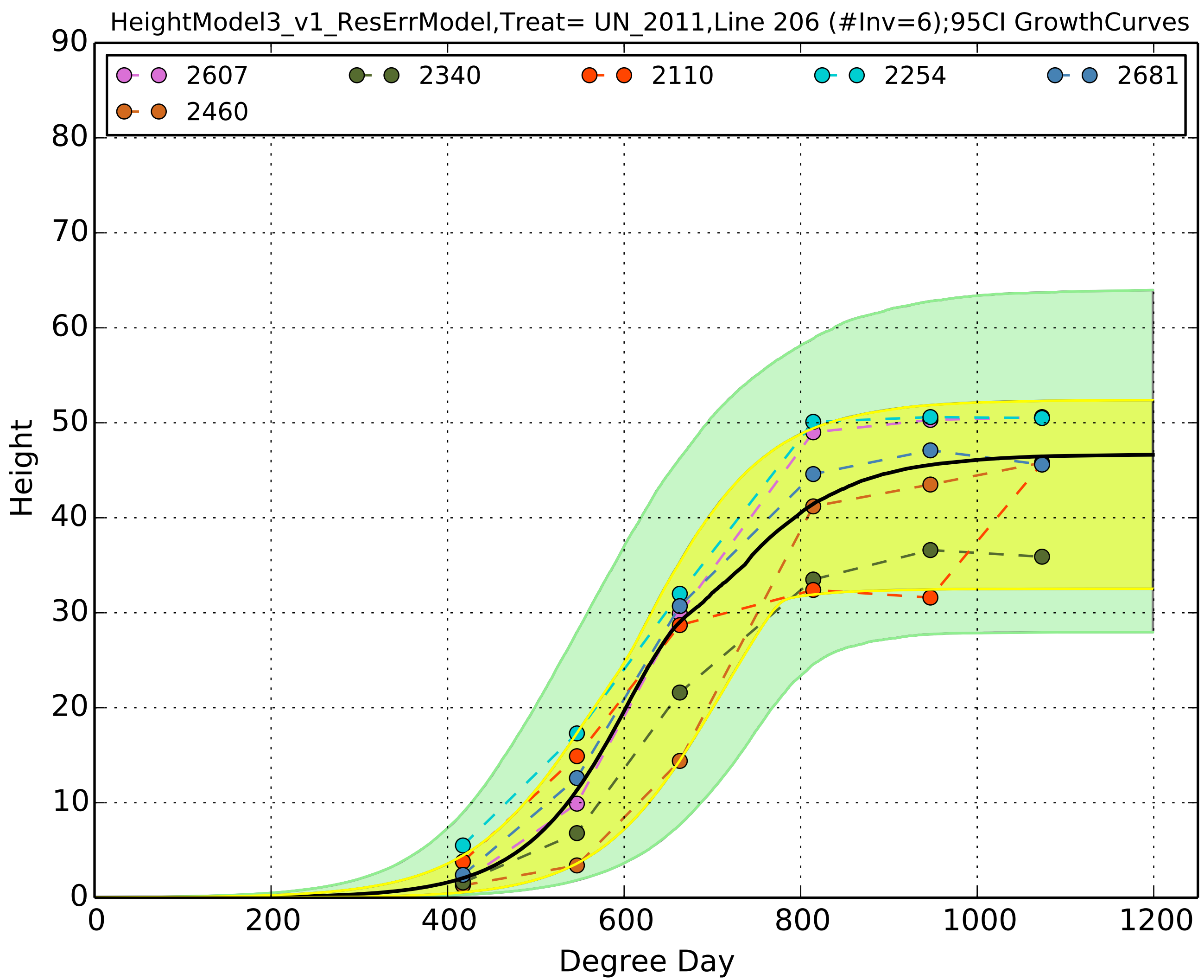

HeightModel3_v1_ResErrModel,Treat= UN_2011,Line 206 (#Inv=6);95CI GrowthCurves
2607
2340
2110
2254
2681
2460
Height
Degree Day
0
10
20
30
40
50
60
70
80
90
0
200
400
600
800
1000
1200

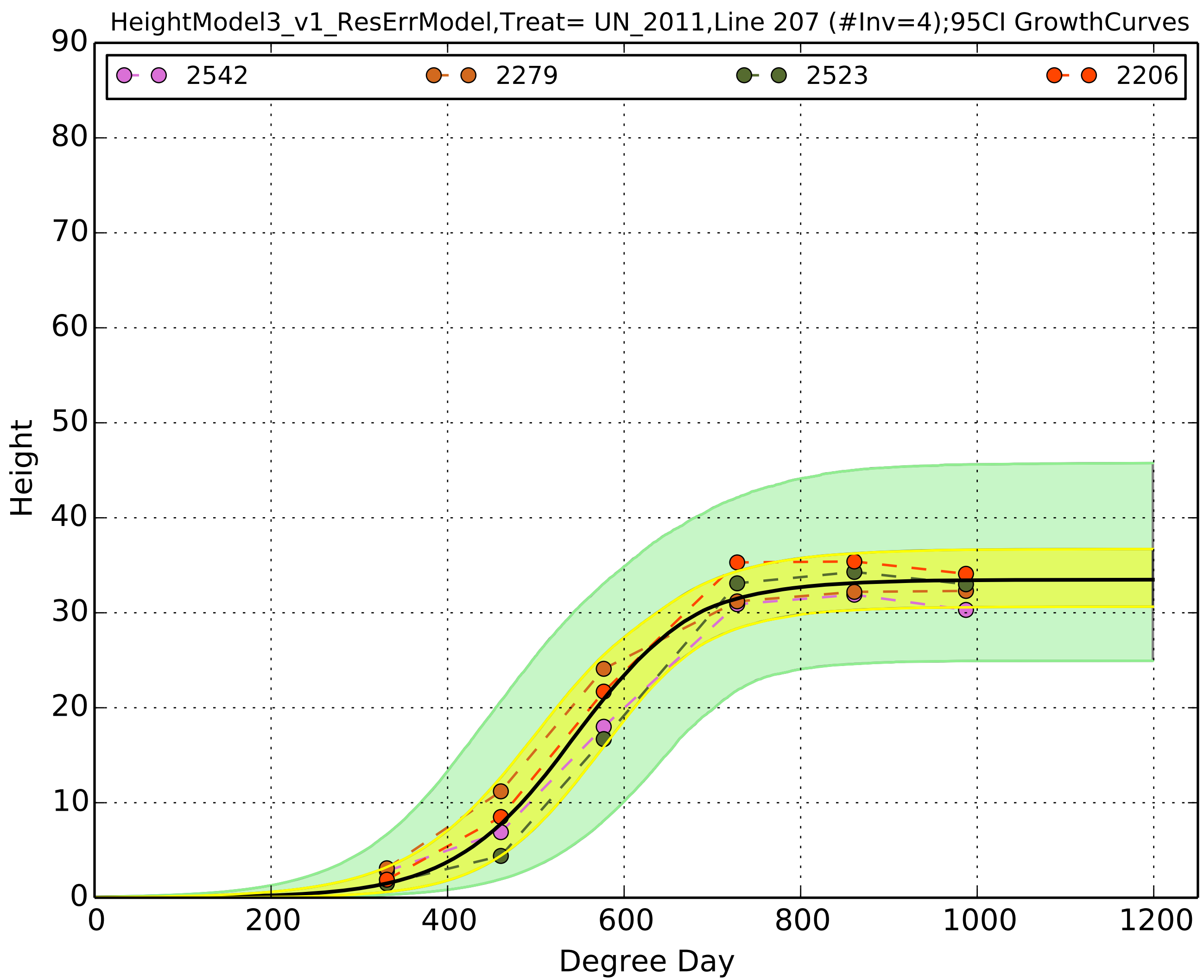

HeightModel3_v1_ResErrModel,Treat= UN_2011,Line 207 (#Inv=4);95CI GrowthCurves
2542
2279
2523
2206
Height
Degree Day
0
10
20
30
40
50
60
70
80
90
0
200
400
600
800
1000
1200

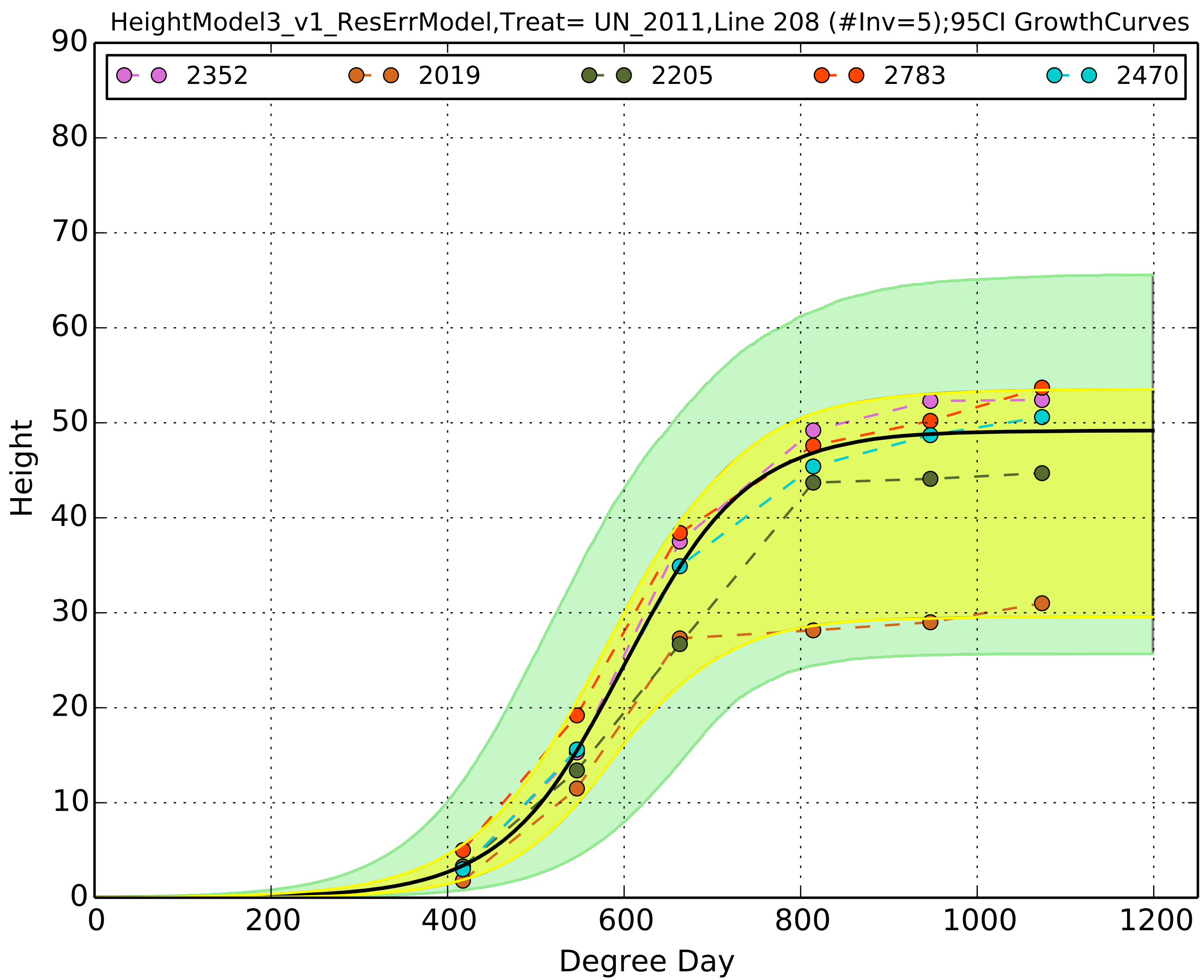
HeightModel3_v1_ResErrModel,Treat= UN_2011,Line 208 (#Inv=5);95CI GrowthCurves
2352
2019
2205
2783
2470
Height
Degree Day
0
10
20
30
40
50
60
70
80
90
0
200
400
600
800
1000
1200

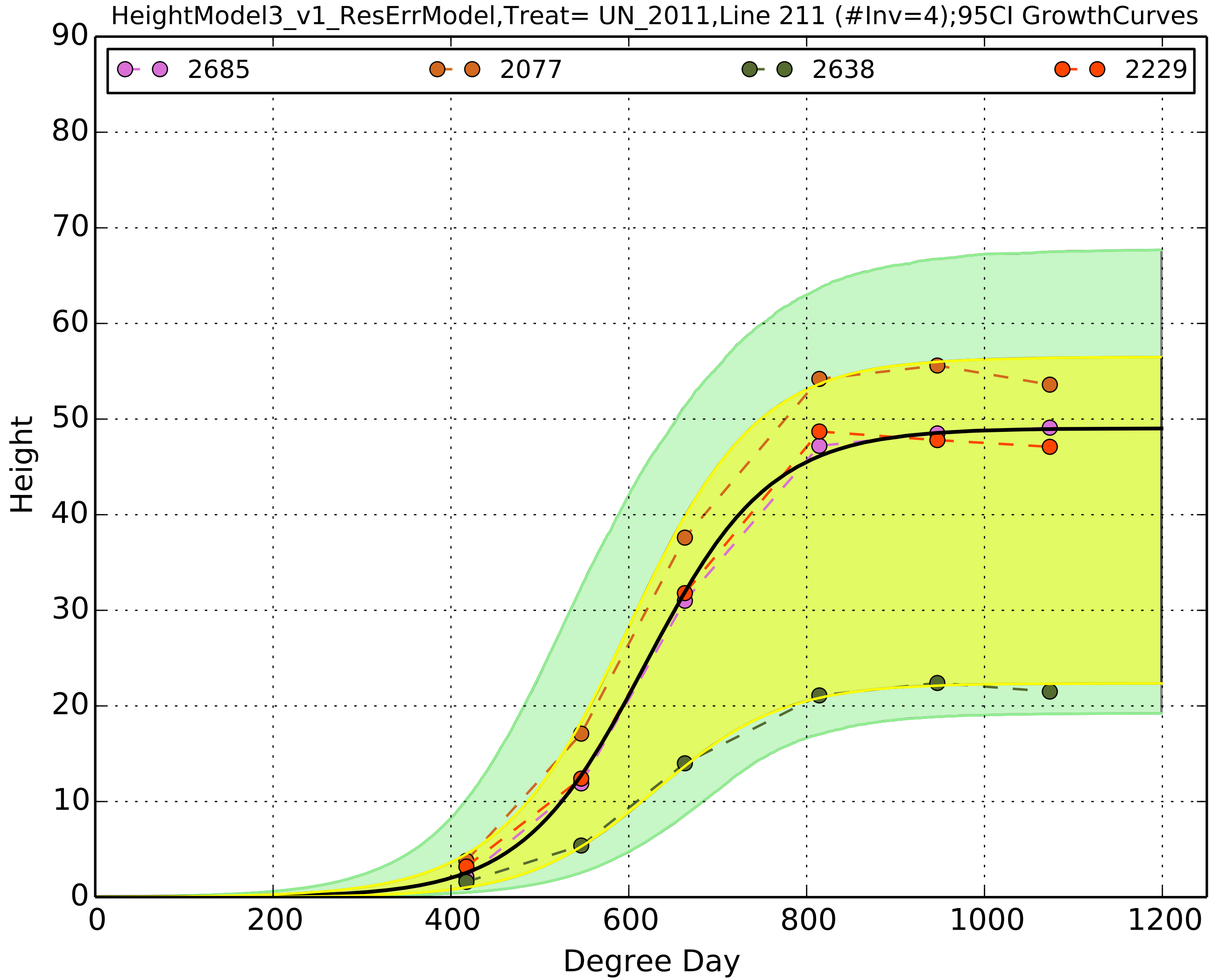

HeightModel3_v1_ResErrModel,Treat= UN_2011,Line 211 (#Inv=4);95CI GrowthCurves
2685
2077
2638
2229
Height
Degree Day
0
10
20
30
40
50
60
70
80
90
0
200
400
600
800
1000
1200

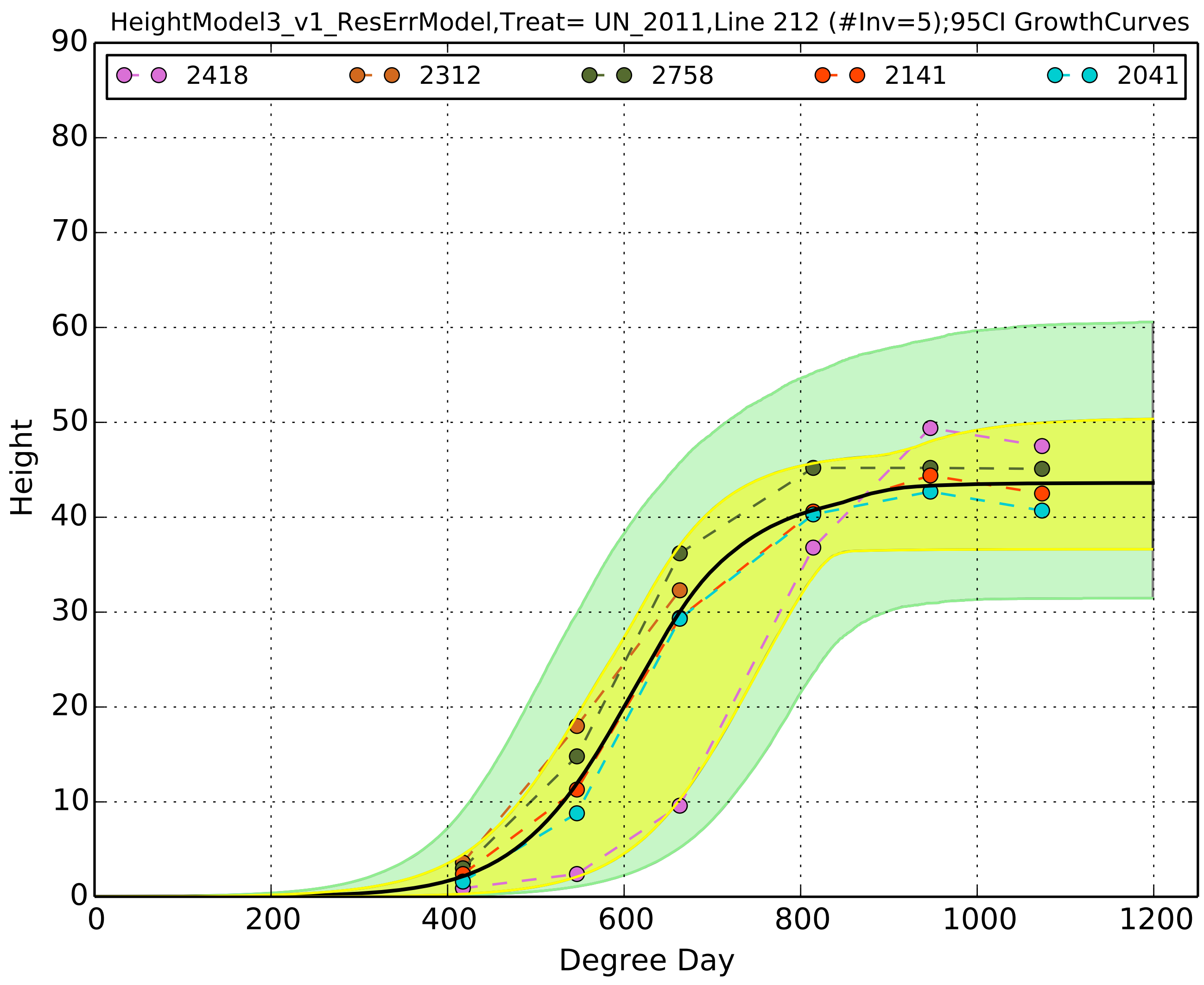

HeightModel3_v1_ResErrModel,Treat= UN_2011,Line 212 (#Inv=5);95CI GrowthCurves
2418
2312
2758
2141
2041
Height
Degree Day
0
10
20
30
40
50
60
70
80
90
0
200
400
600
800
1000
1200

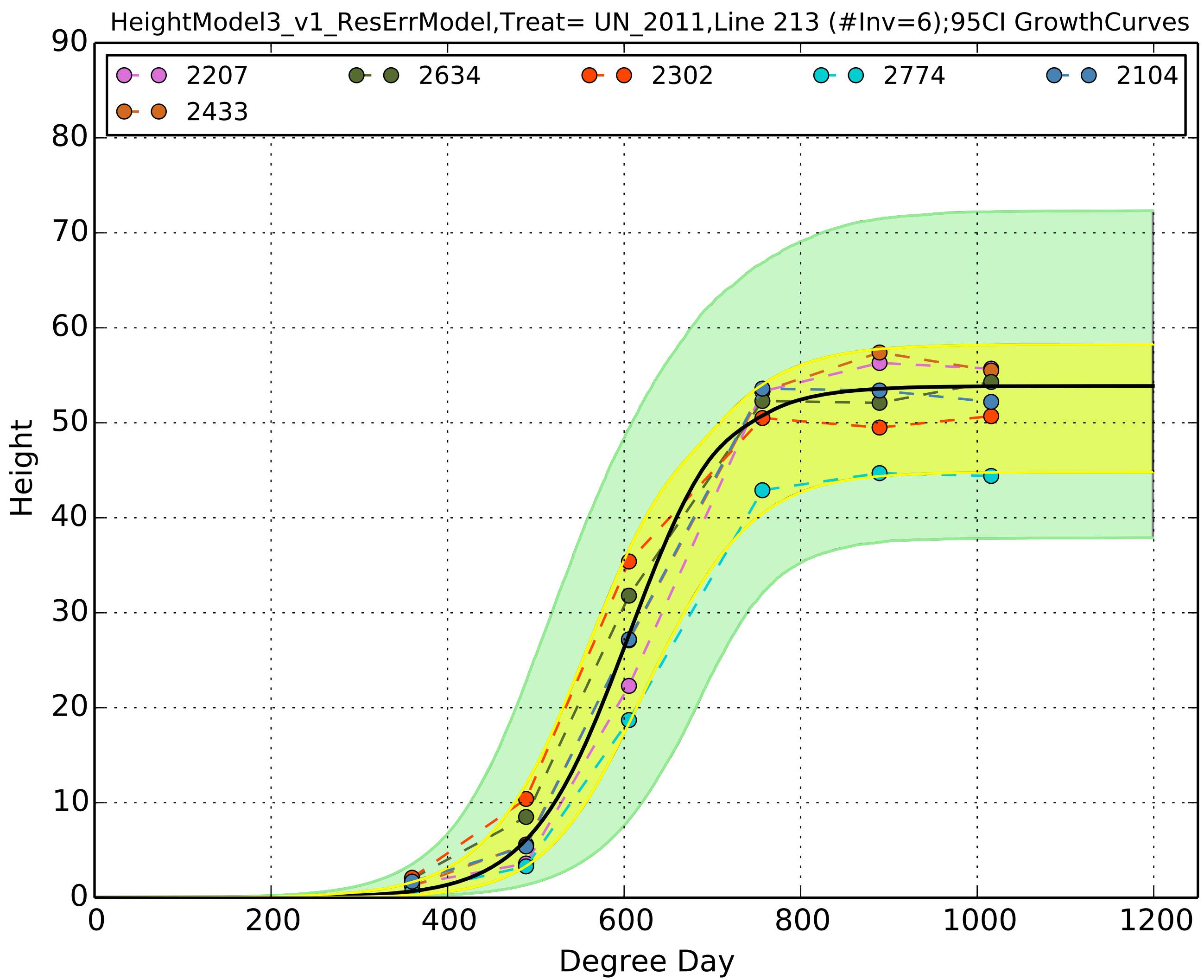

HeightModel3_v1_ResErrModel,Treat= UN_2011,Line 213 (#Inv=6);95CI GrowthCurves
2207
2634
2302
2774
2104
2433
Height
Degree Day
0
10
20
30
40
50
60
70
80
90
0
200
400
600
800
1000
1200

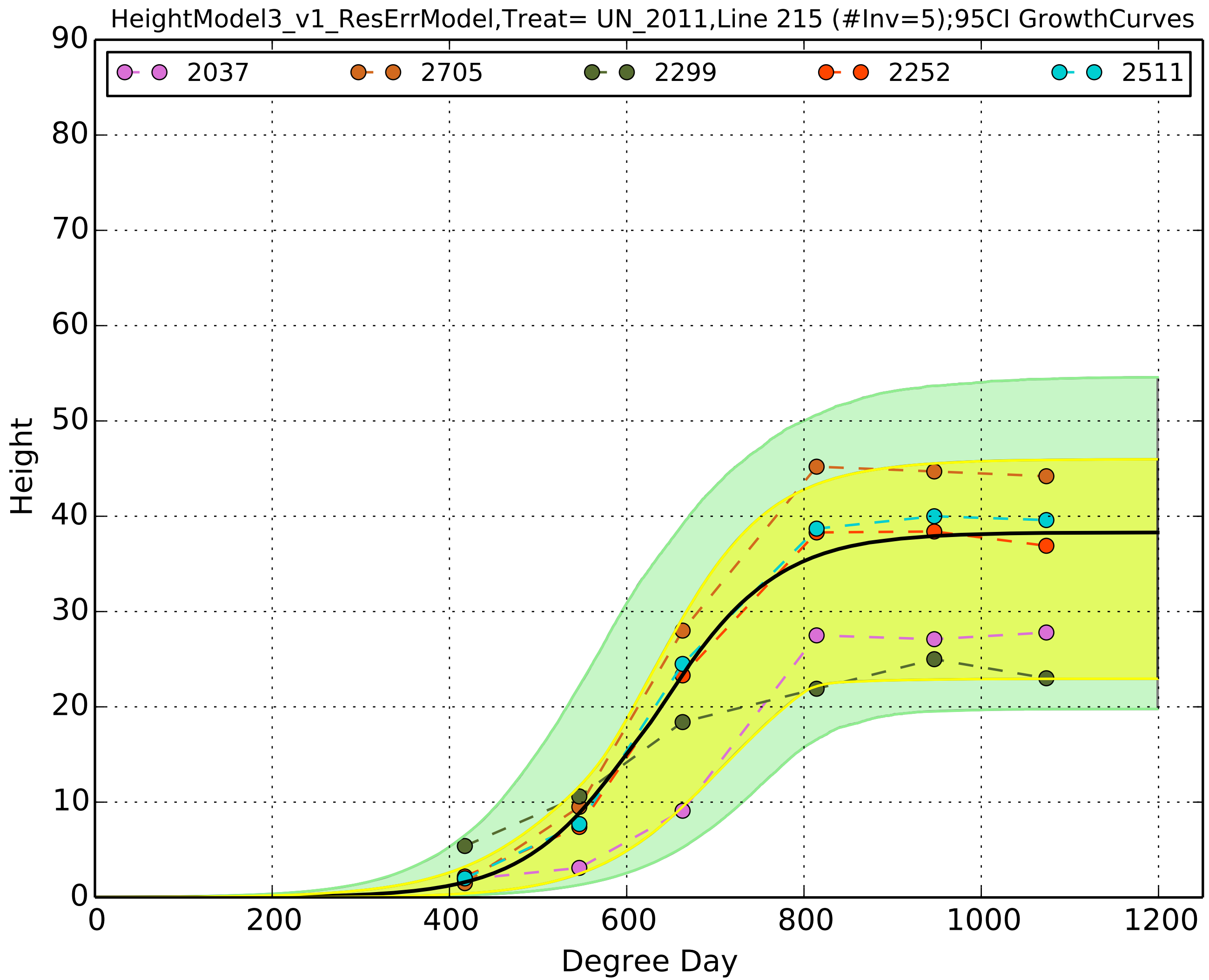

HeightModel3_v1_ResErrModel,Treat= UN_2011,Line 215 (#Inv=5);95CI GrowthCurves
2037
2705
2299
2252
2511
Height
Degree Day
0
10
20
30
40
50
60
70
80
90
0
200
400
600
800
1000
1200

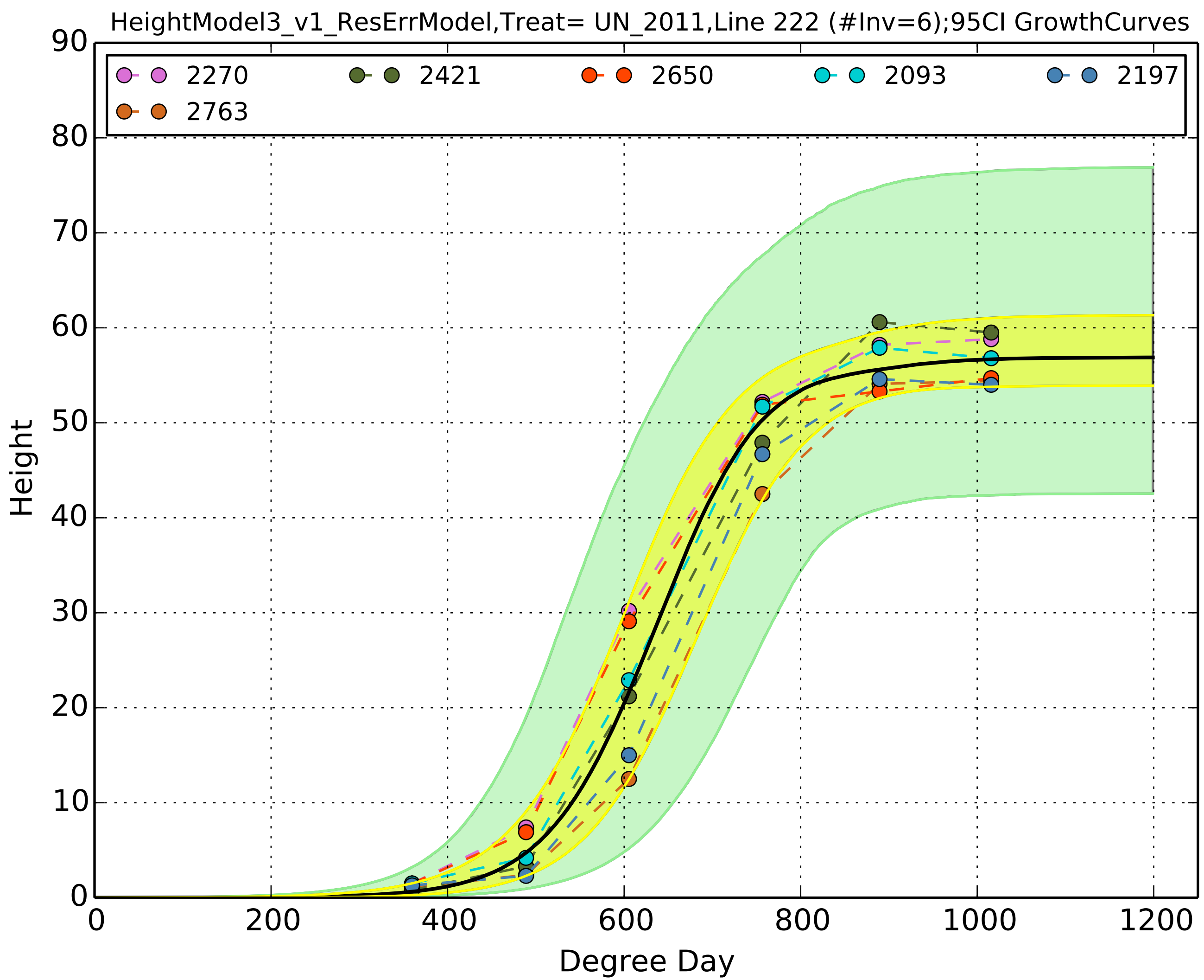

HeightModel3_v1_ResErrModel,Treat= UN_2011,Line 222 (#Inv=6);95CI GrowthCurves
2270
2421
2650
2093
2197
2763
Height
Degree Day
0
10
20
30
40
50
60
70
80
90
0
200
400
600
800
1000
1200

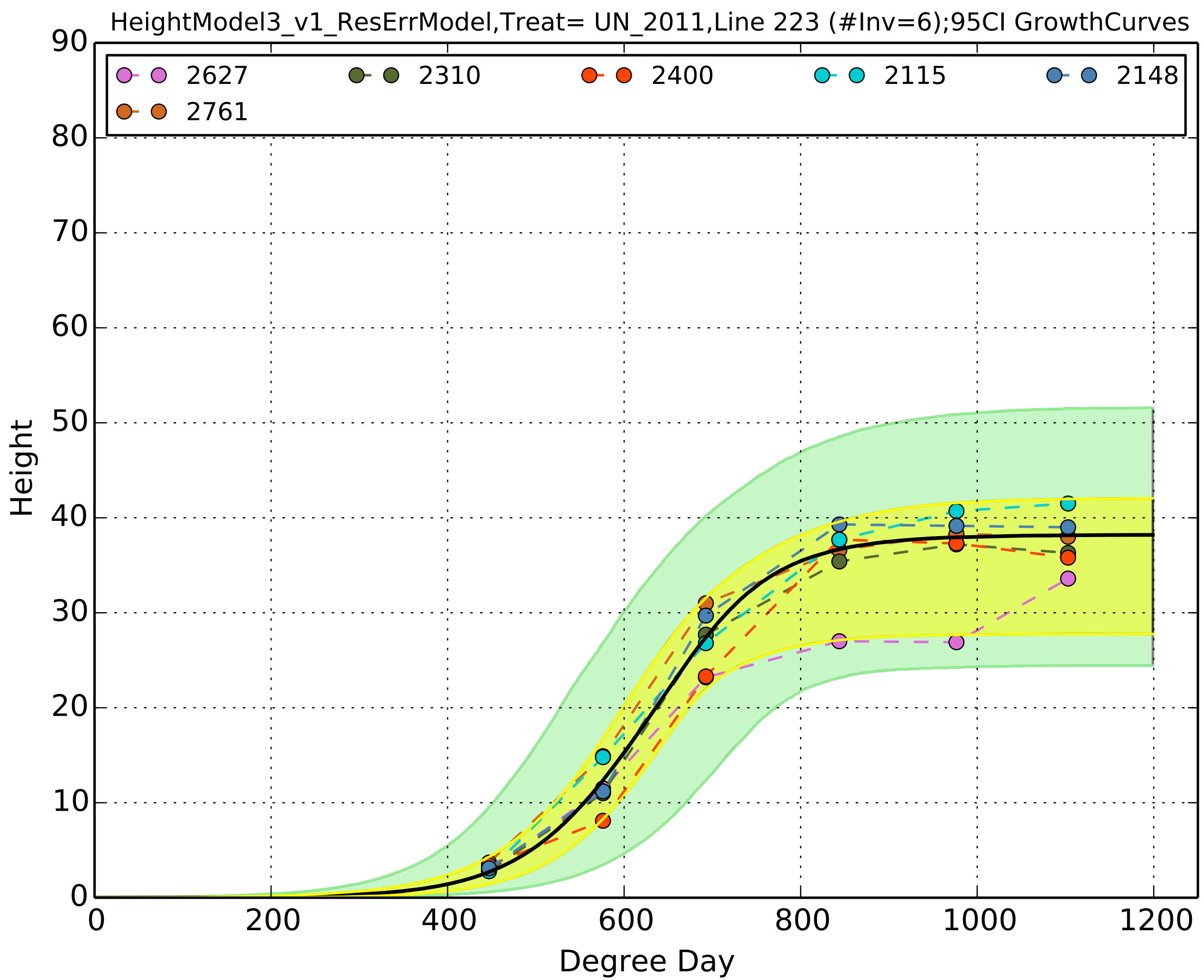

HeightModel3_v1_ResErrModel,Treat= UN_2011,Line 223 (#Inv=6);95CI GrowthCurves
2627
2310
2400
2115
2148
2761
Height
Degree Day
0
10
20
30
40
50
60
70
80
90
0
200
400
600
800
1000
1200

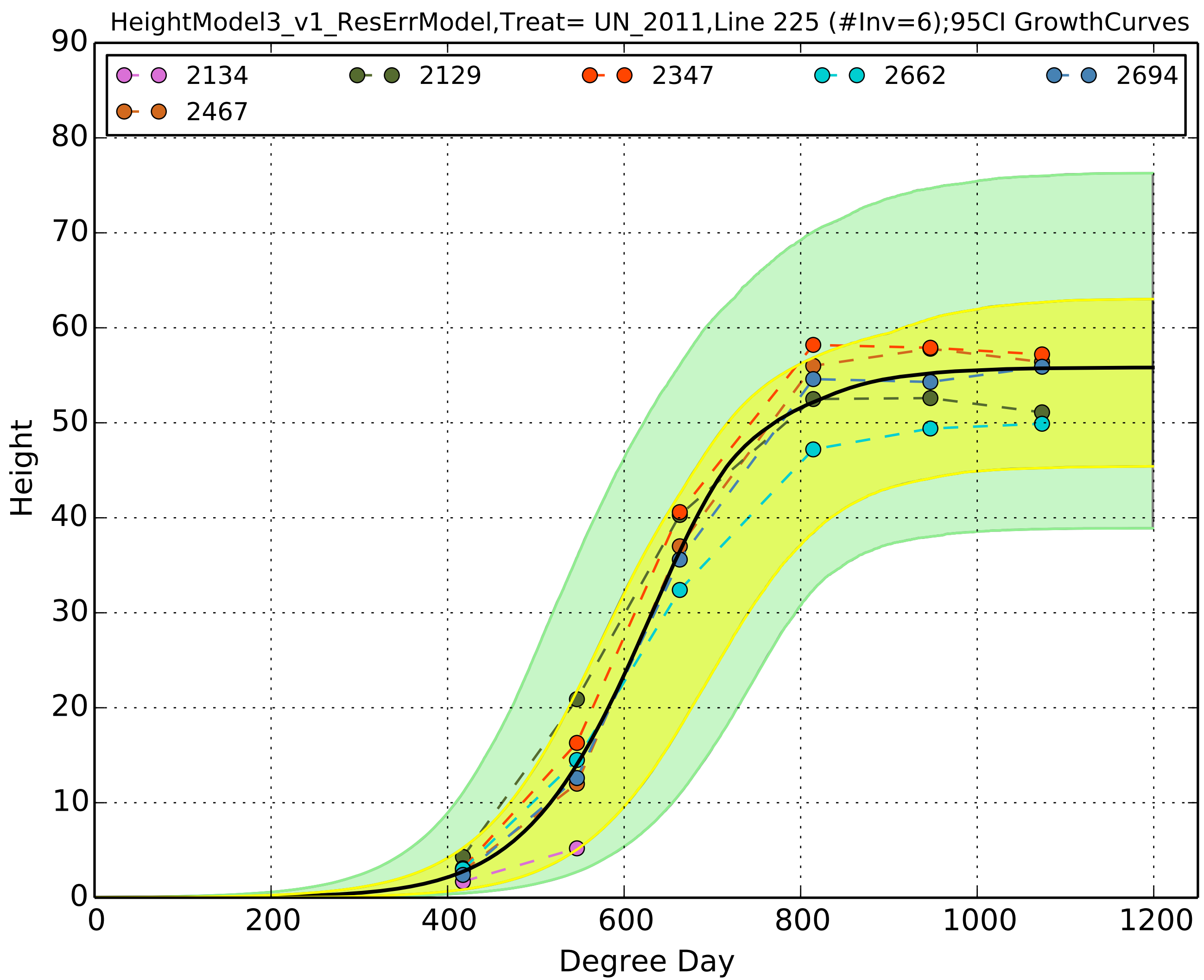
HeightModel3_v1_ResErrModel,Treat= UN_2011,Line 225 (#Inv=6);95CI GrowthCurves
2134
2129
2347
2662
2694
2467
Height
Degree Day
0
10
20
30
40
50
60
70
80
90
0
200
400
600
800
1000
1200

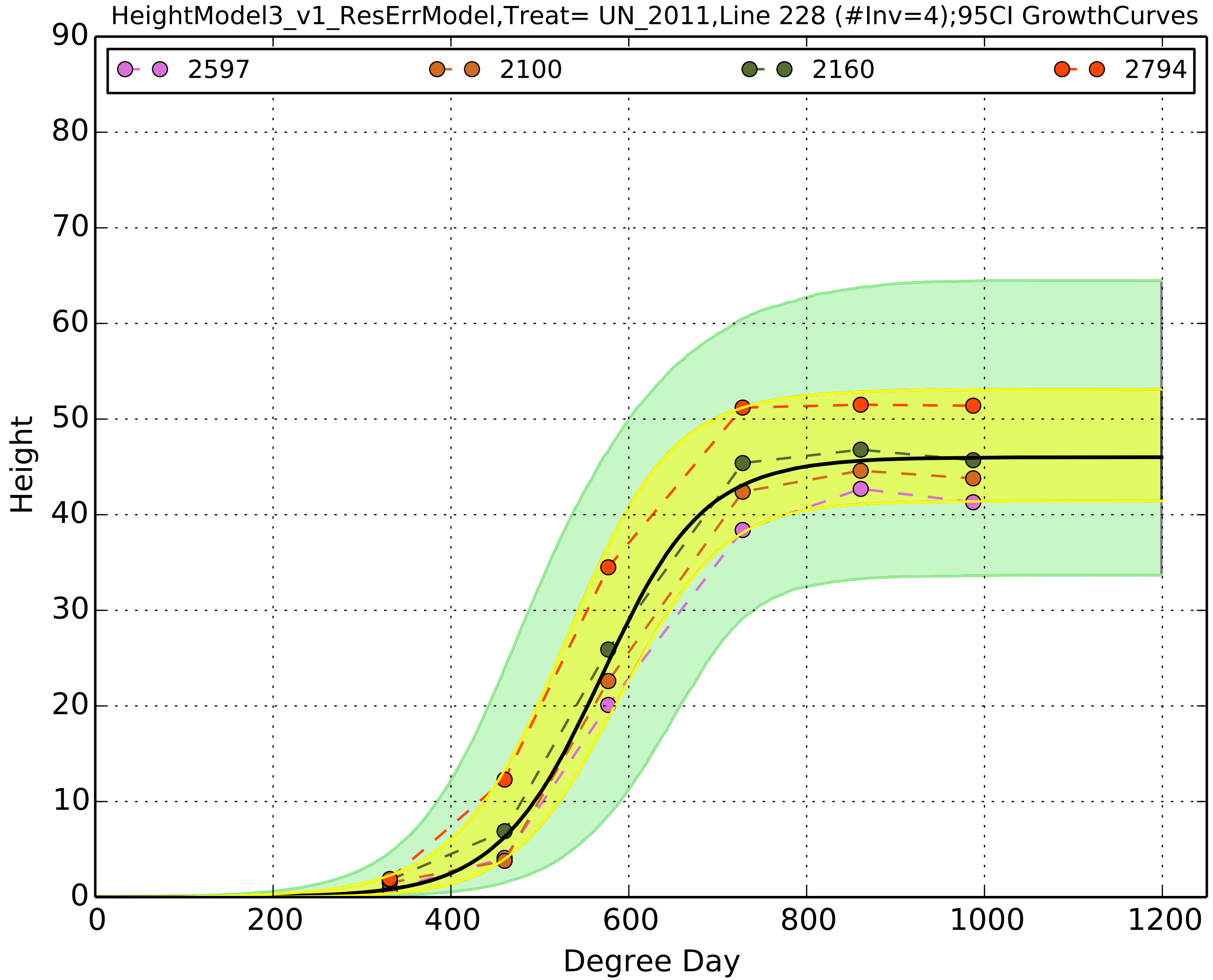
HeightModel3_v1_ResErrModel,Treat= UN_2011,Line 228 (#Inv=4);95CI GrowthCurves
2597
2100
2160
2794
Height
Degree Day
0
10
20
30
40
50
60
70
80
90
0
200
400
600
800
1000
1200

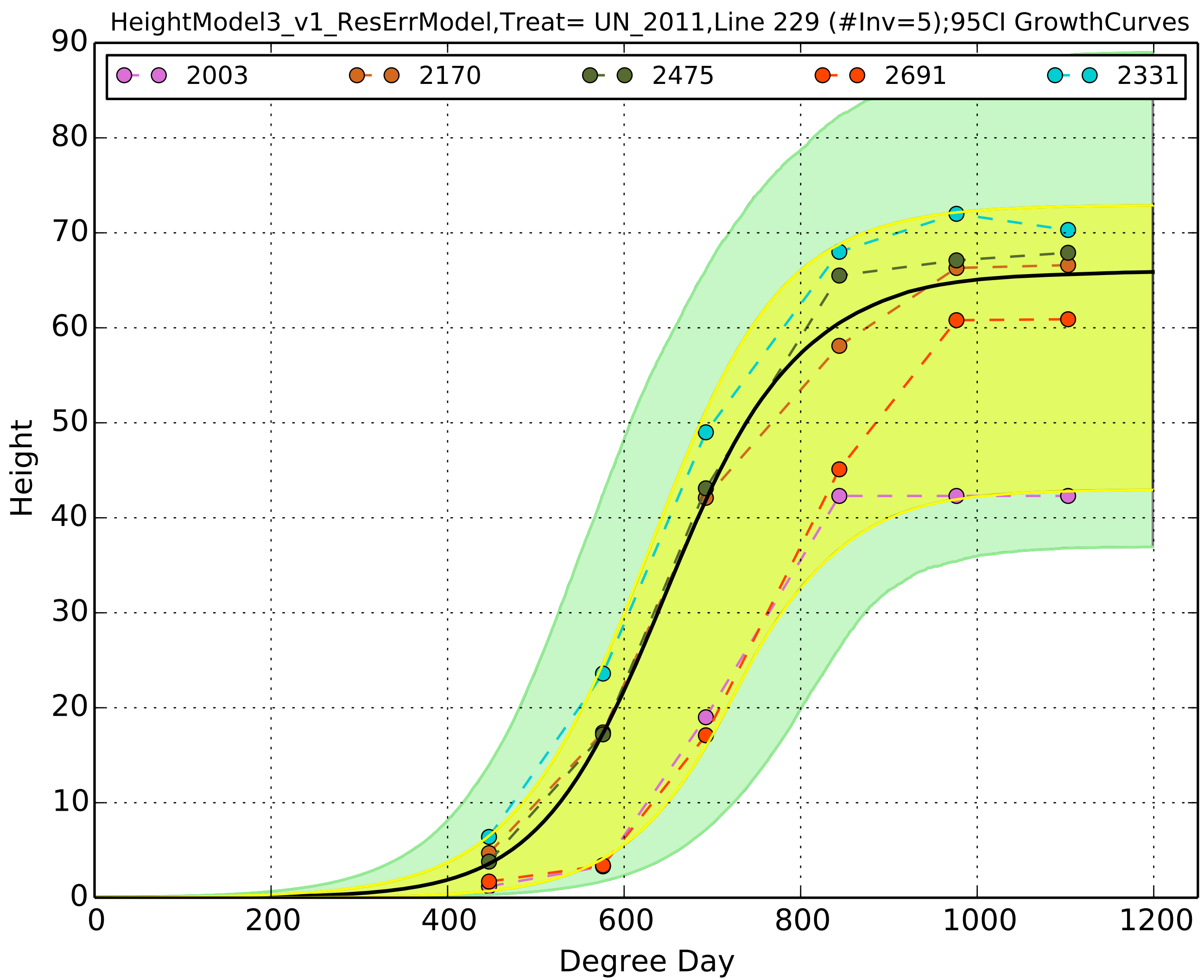

HeightModel3_v1_ResErrModel,Treat= UN_2011,Line 229 (#Inv=5);95CI GrowthCurves
2003
2170
2475
2691
2331
Height
Degree Day
0
10
20
30
40
50
60
70
80
90
0
200
400
600
800
1000
1200

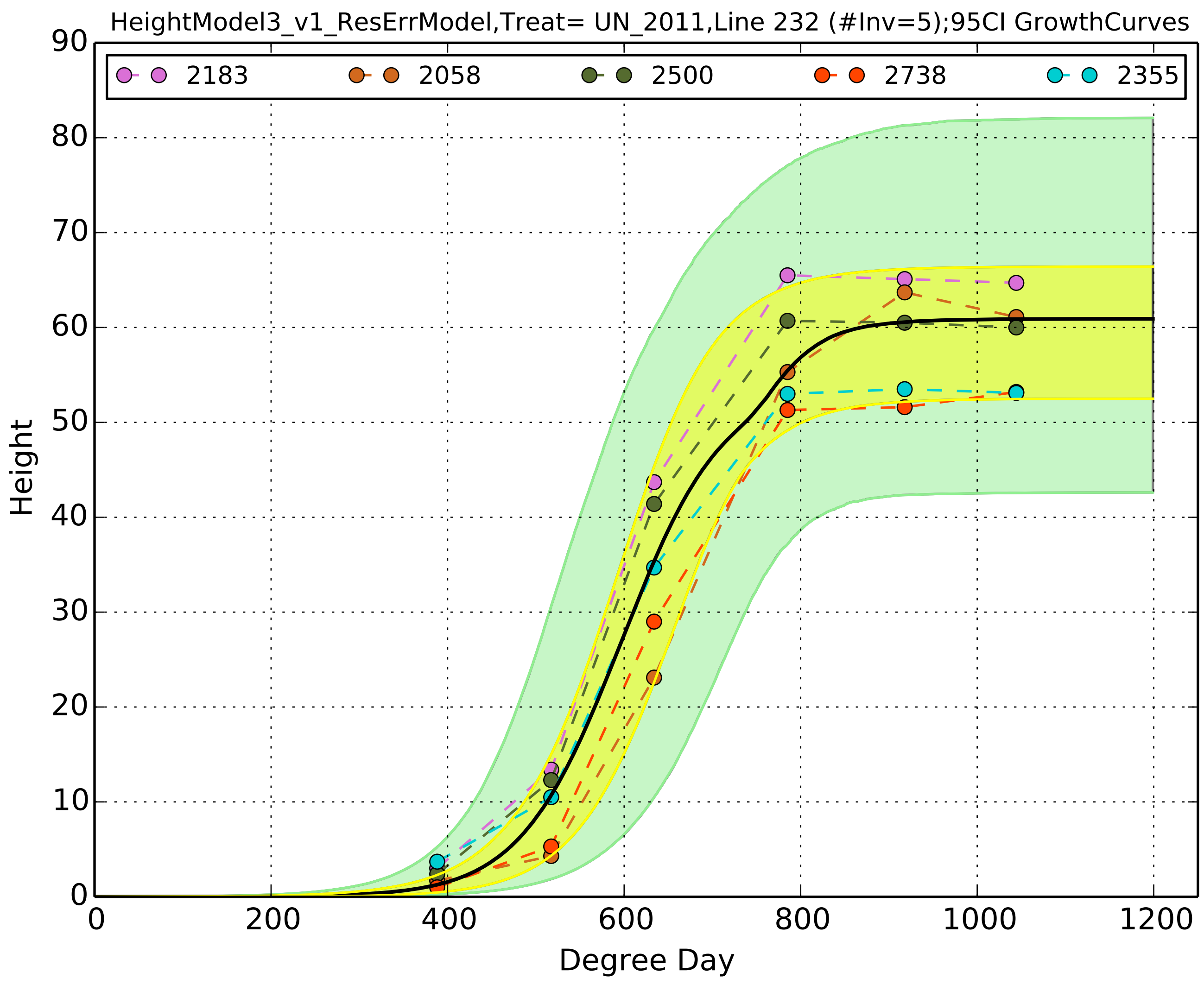
HeightModel3_v1_ResErrModel,Treat= UN_2011,Line 232 (#Inv=5);95CI GrowthCurves
2183
2058
2500
2738
2355
Height
Degree Day
0
10
20
30
40
50
60
70
80
90
0
200
400
600
800
1000
1200

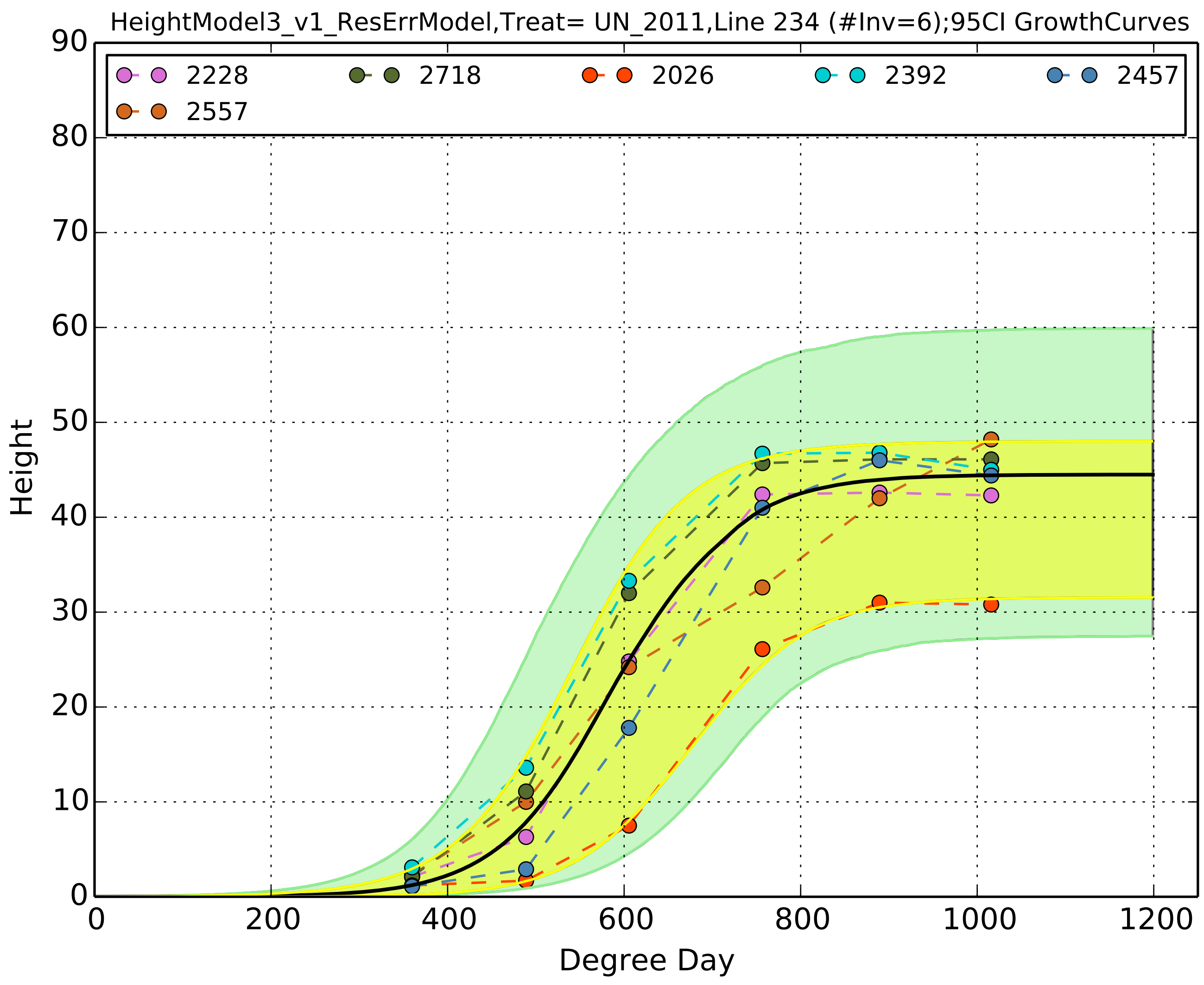

HeightModel3_v1_ResErrModel,Treat= UN_2011,Line 234 (#Inv=6);95CI GrowthCurves
2228
2718
2026
2392
2457
2557
Height
Degree Day
0
10
20
30
40
50
60
70
80
90
0
200
400
600
800
1000
1200

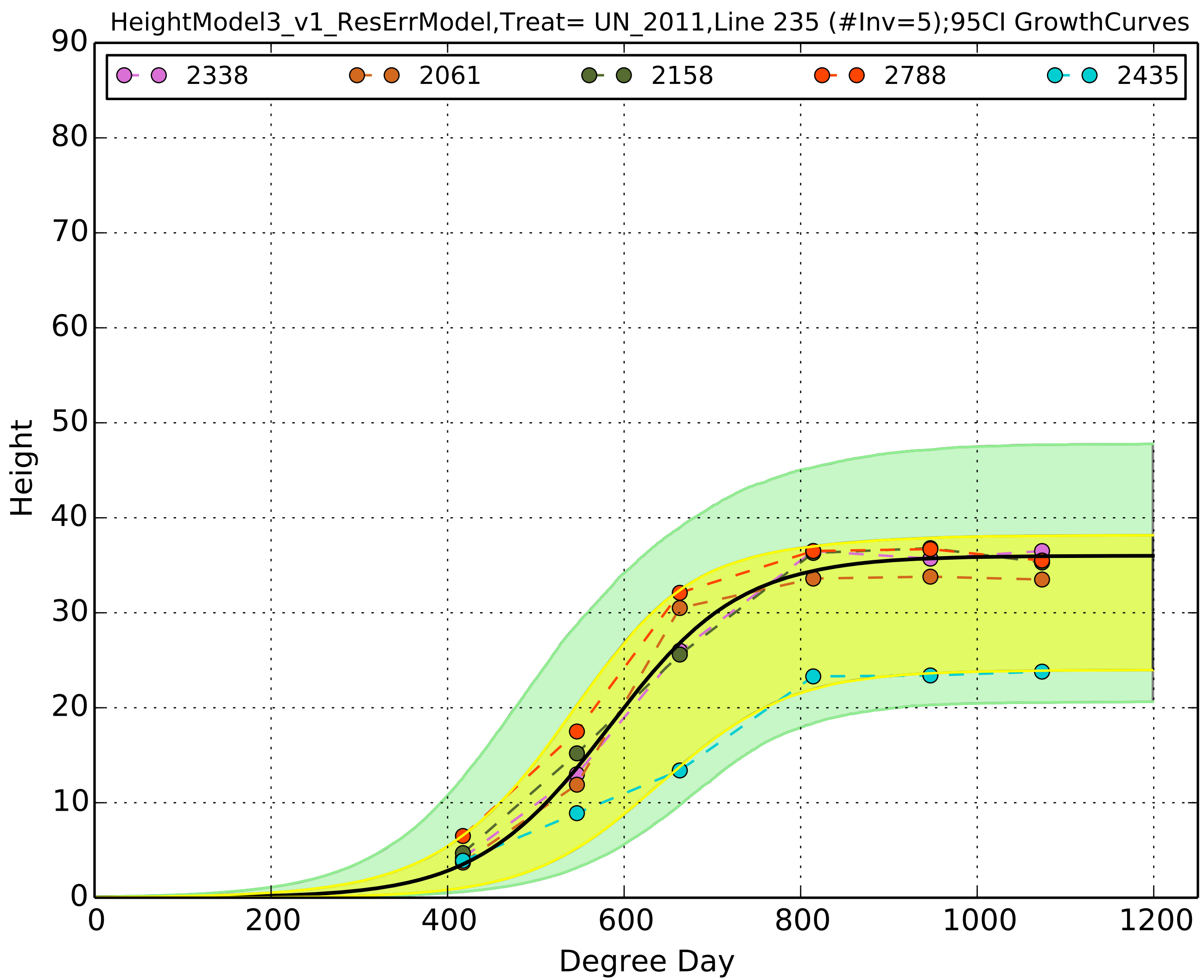

HeightModel3_v1_ResErrModel,Treat= UN_2011,Line 235 (#Inv=5);95CI GrowthCurves
2338
2061
2158
2788
2435
Height
Degree Day
0
10
20
30
40
50
60
70
80
90
0
200
400
600
800
1000
1200

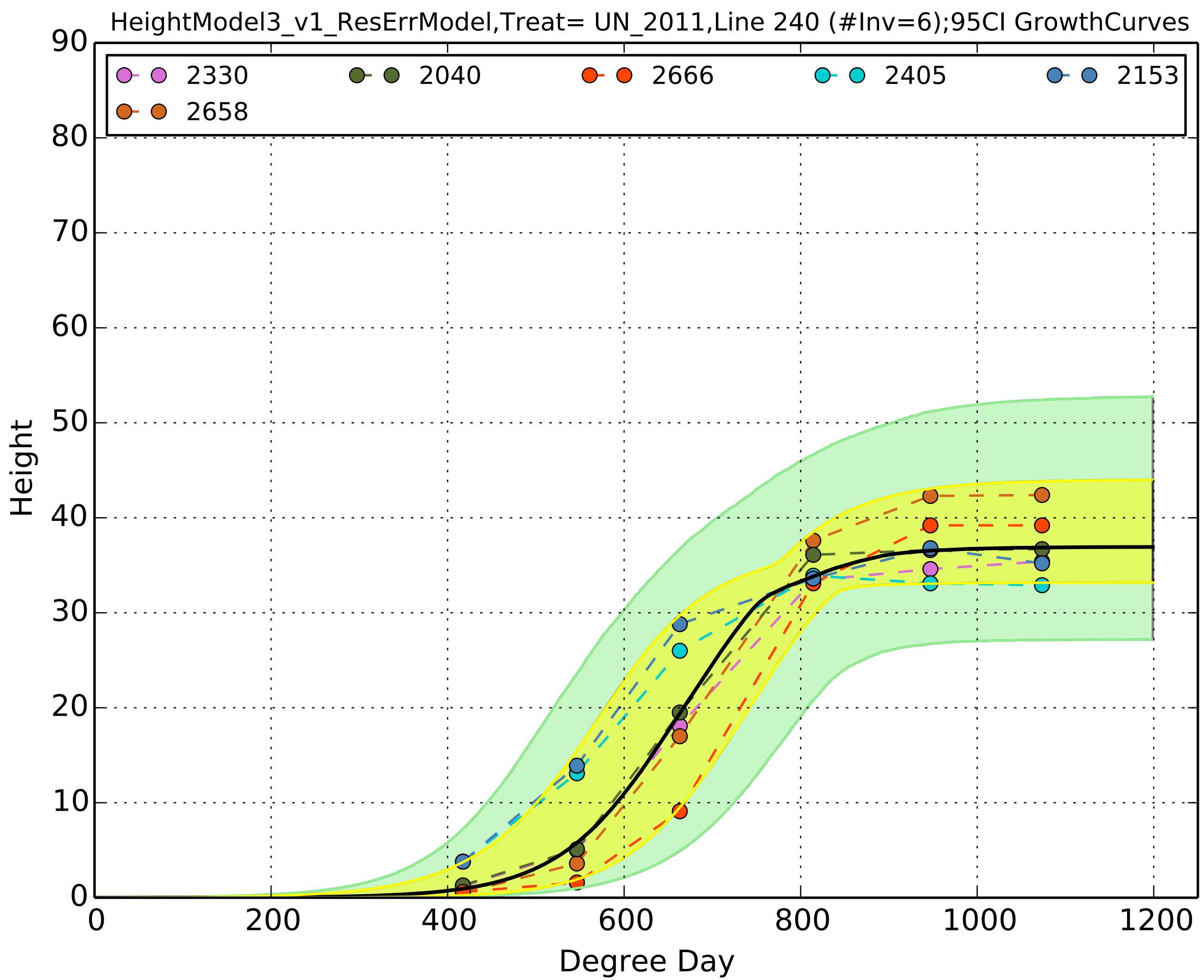

HeightModel3_v1_ResErrModel,Treat= UN_2011,Line 240 (#Inv=6);95CI GrowthCurves
2330
2040
2666
2405
2153
2658
Height
Degree Day
0
10
20
30
40
50
60
70
80
90
0
200
400
600
800
1000
1200

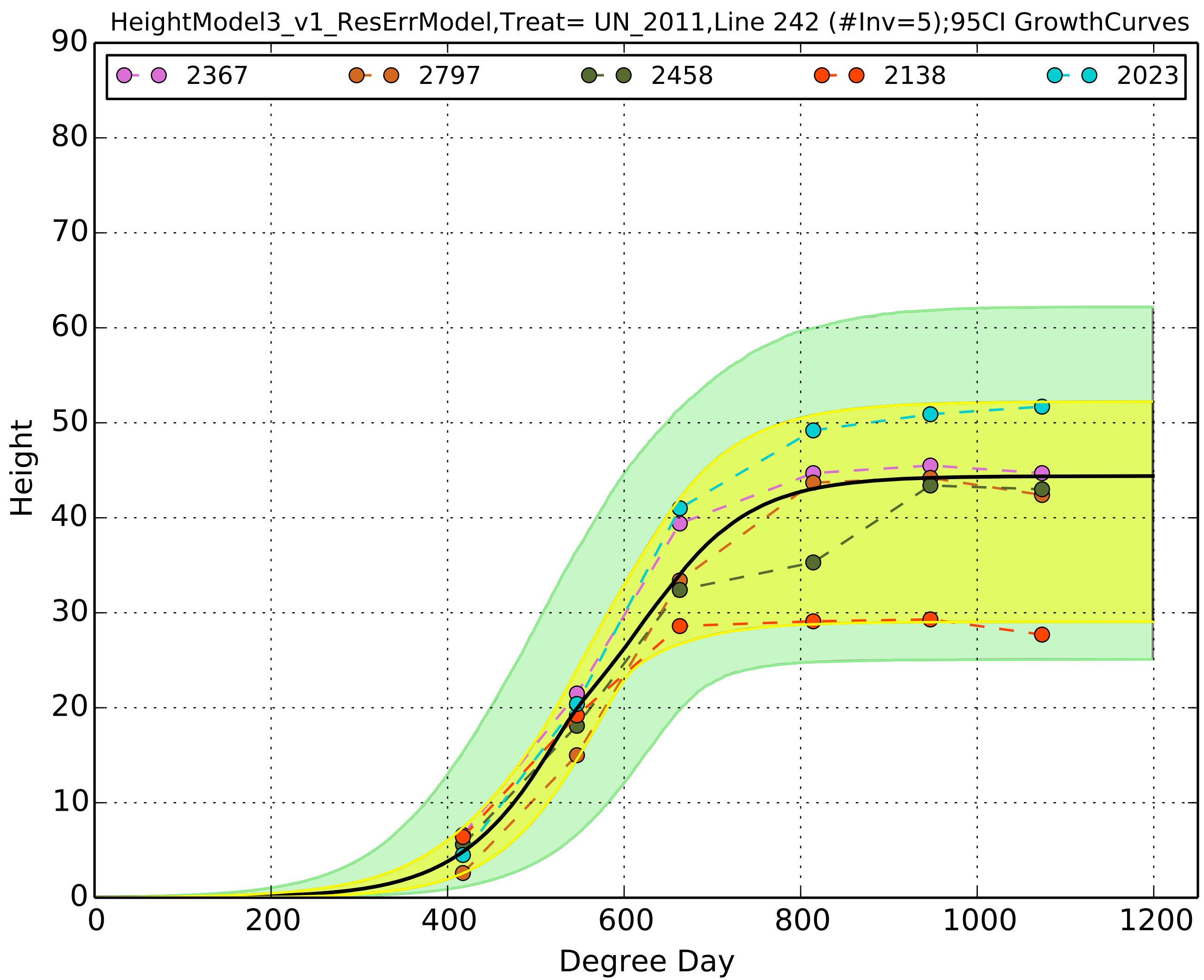

HeightModel3_v1_ResErrModel,Treat= UN_2011,Line 242 (#Inv=5);95CI GrowthCurves
2367
2797
2458
2138
2023
Height
Degree Day
0
10
20
30
40
50
60
70
80
90
0
200
400
600
800
1000
1200

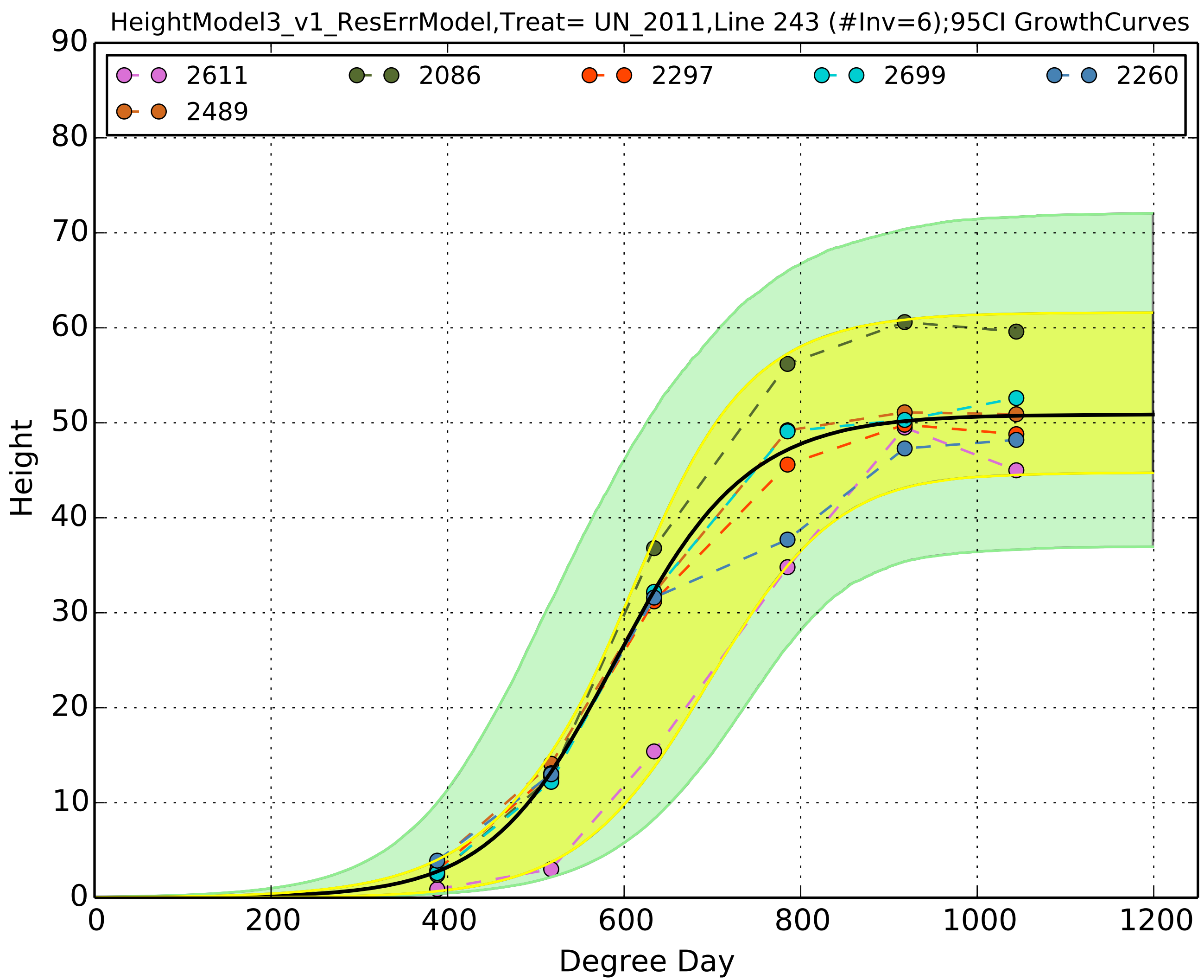

HeightModel3_v1_ResErrModel,Treat= UN_2011,Line 243 (#Inv=6);95CI GrowthCurves
2611
2086
2297
2699
2260
2489
Height
Degree Day
0
10
20
30
40
50
60
70
80
90
0
200
400
600
800
1000
1200

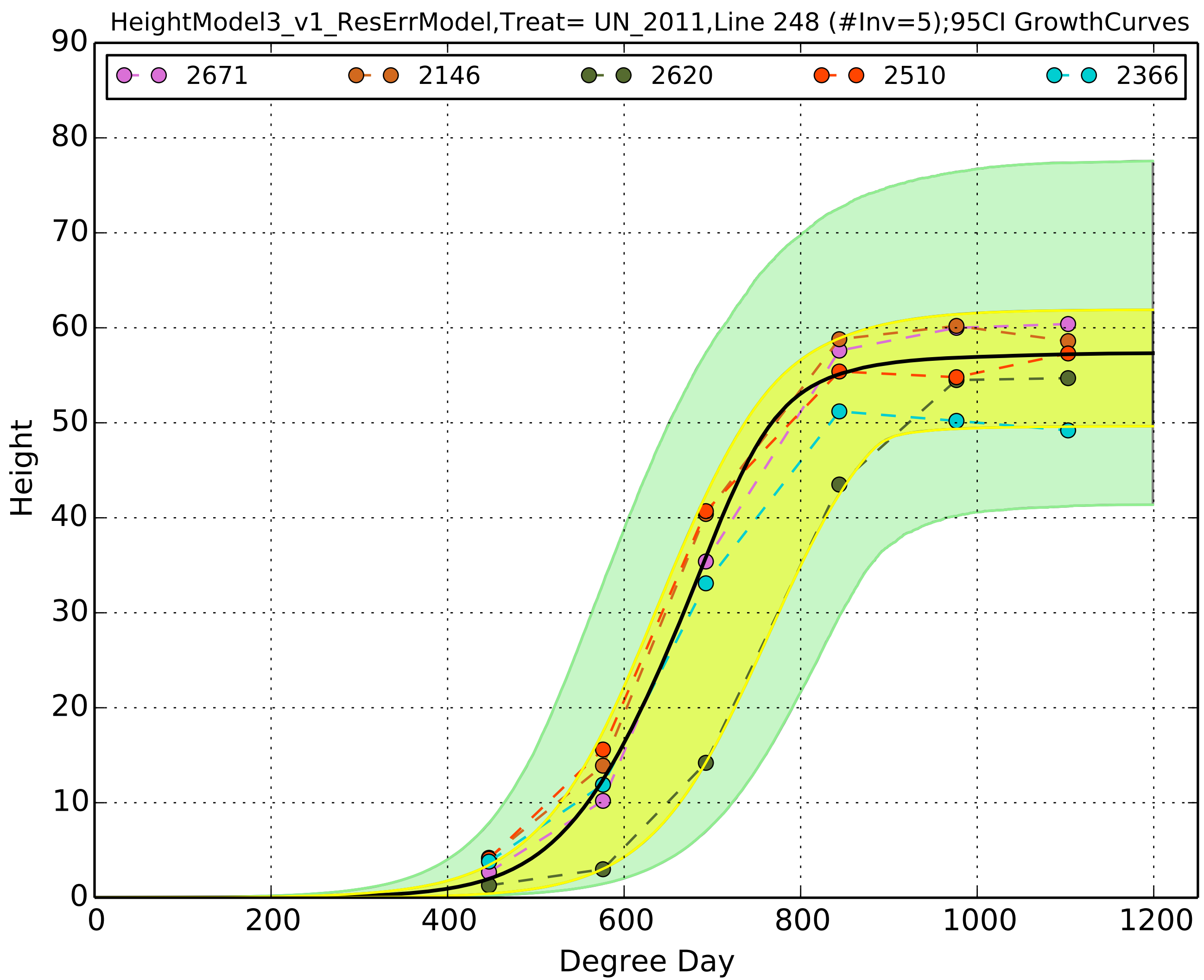

HeightModel3_v1_ResErrModel,Treat= UN_2011,Line 248 (#Inv=5);95CI GrowthCurves
2671
2146
2620
2510
2366
Height
Degree Day
0
10
20
30
40
50
60
70
80
90
0
200
400
600
800
1000
1200

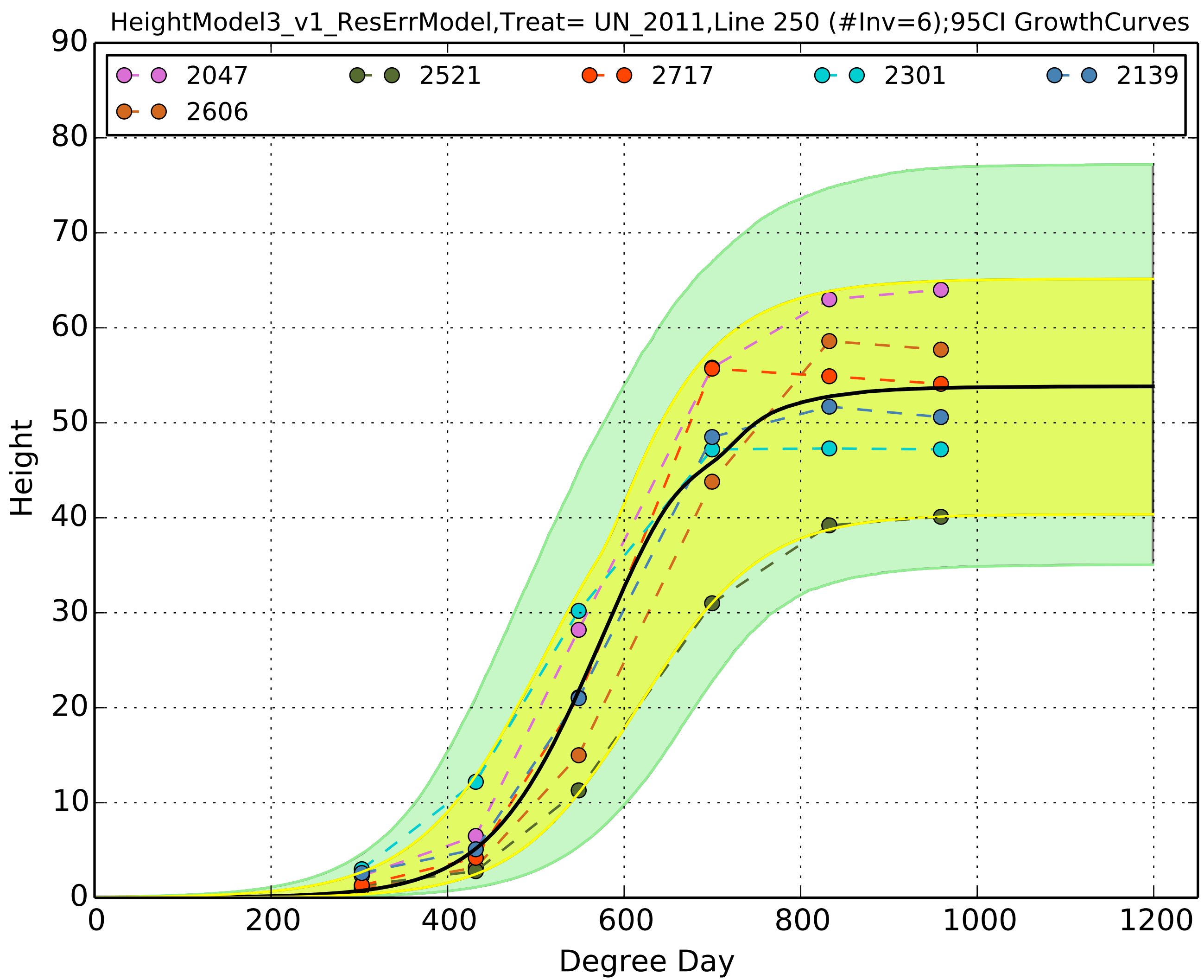
HeightModel3_v1_ResErrModel,Treat= UN_2011,Line 250 (#Inv=6);95CI GrowthCurves
2047
2521
2717
2301
2139
2606
Height
Degree Day
0
10
20
30
40
50
60
70
80
90
0
200
400
600
800
1000
1200

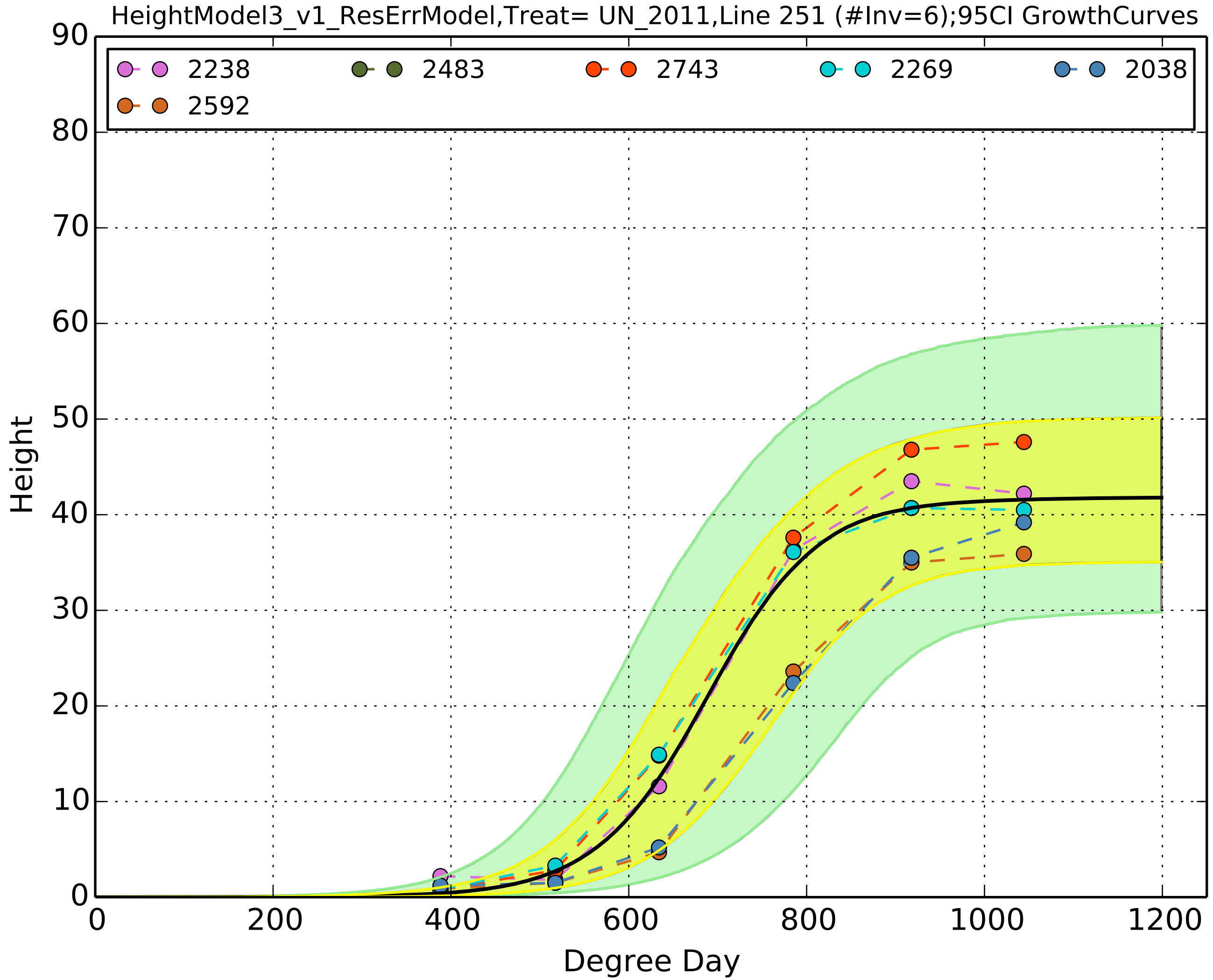
HeightModel3_v1_ResErrModel,Treat= UN_2011,Line 251 (#Inv=6);95CI GrowthCurves
2238
2483
2743
2269
2038
2592
Height
Degree Day
0
10
20
30
40
50
60
70
80
90
0
200
400
600
800
1000
1200

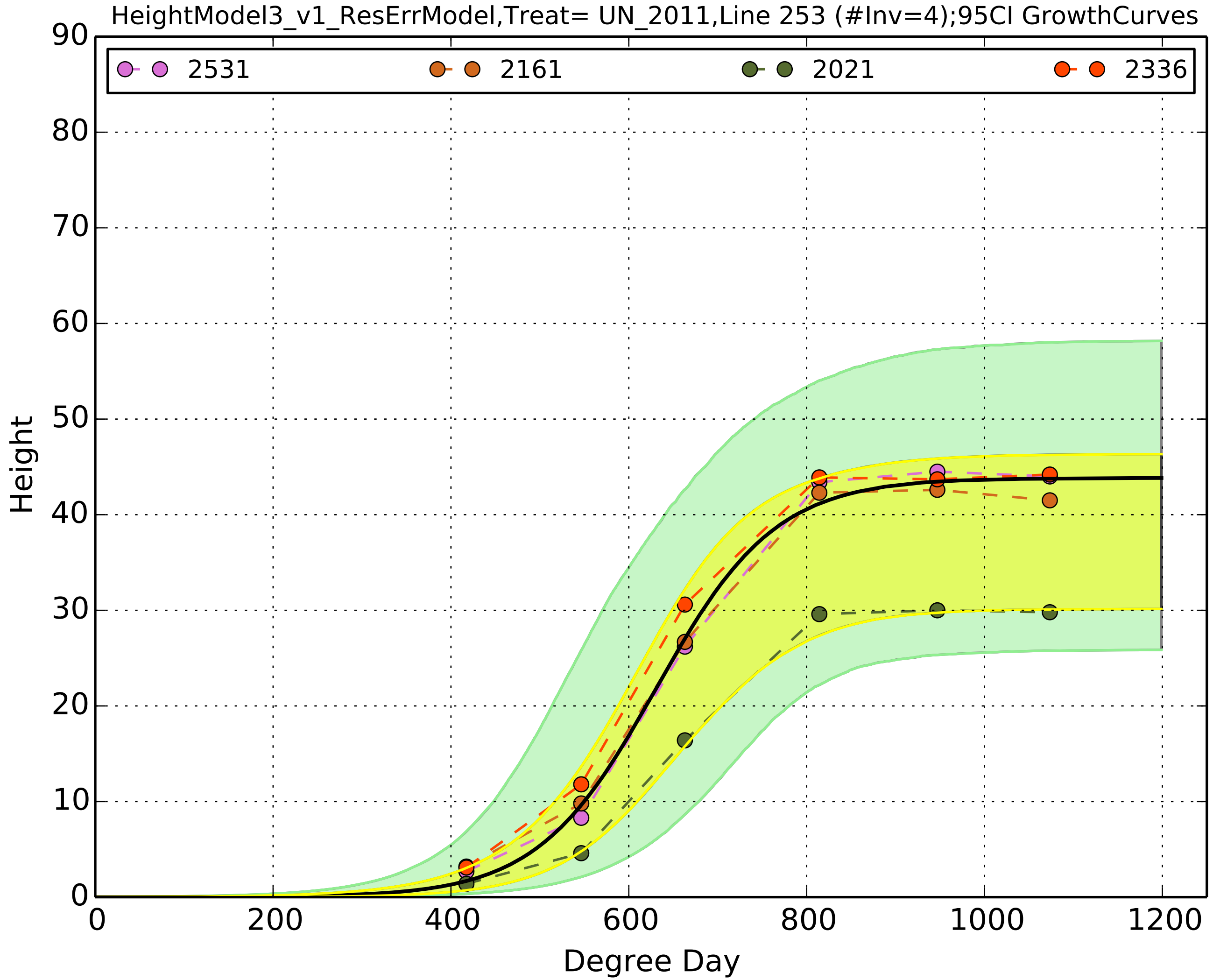

HeightModel3_v1_ResErrModel,Treat= UN_2011,Line 253 (#Inv=4);95CI GrowthCurves
2531
2161
2021
2336
Height
Degree Day
0
10
20
30
40
50
60
70
80
90
0
200
400
600
800
1000
1200

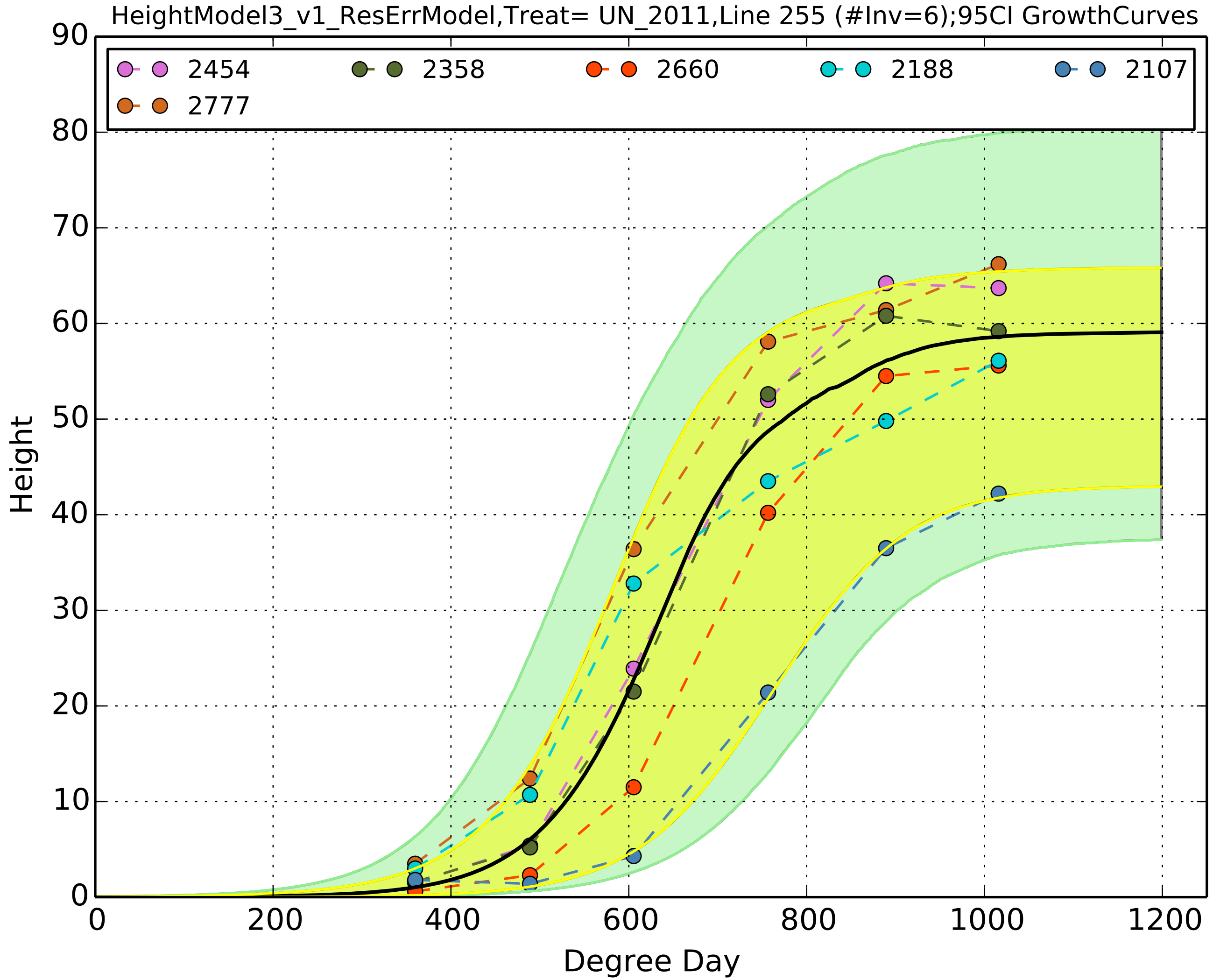

HeightModel3_v1_ResErrModel,Treat= UN_2011,Line 255 (#Inv=6);95CI GrowthCurves
2454
2358
2660
2188
2107
2777
Height
Degree Day
0
10
20
30
40
50
60
70
80
90
0
200
400
600
800
1000
1200

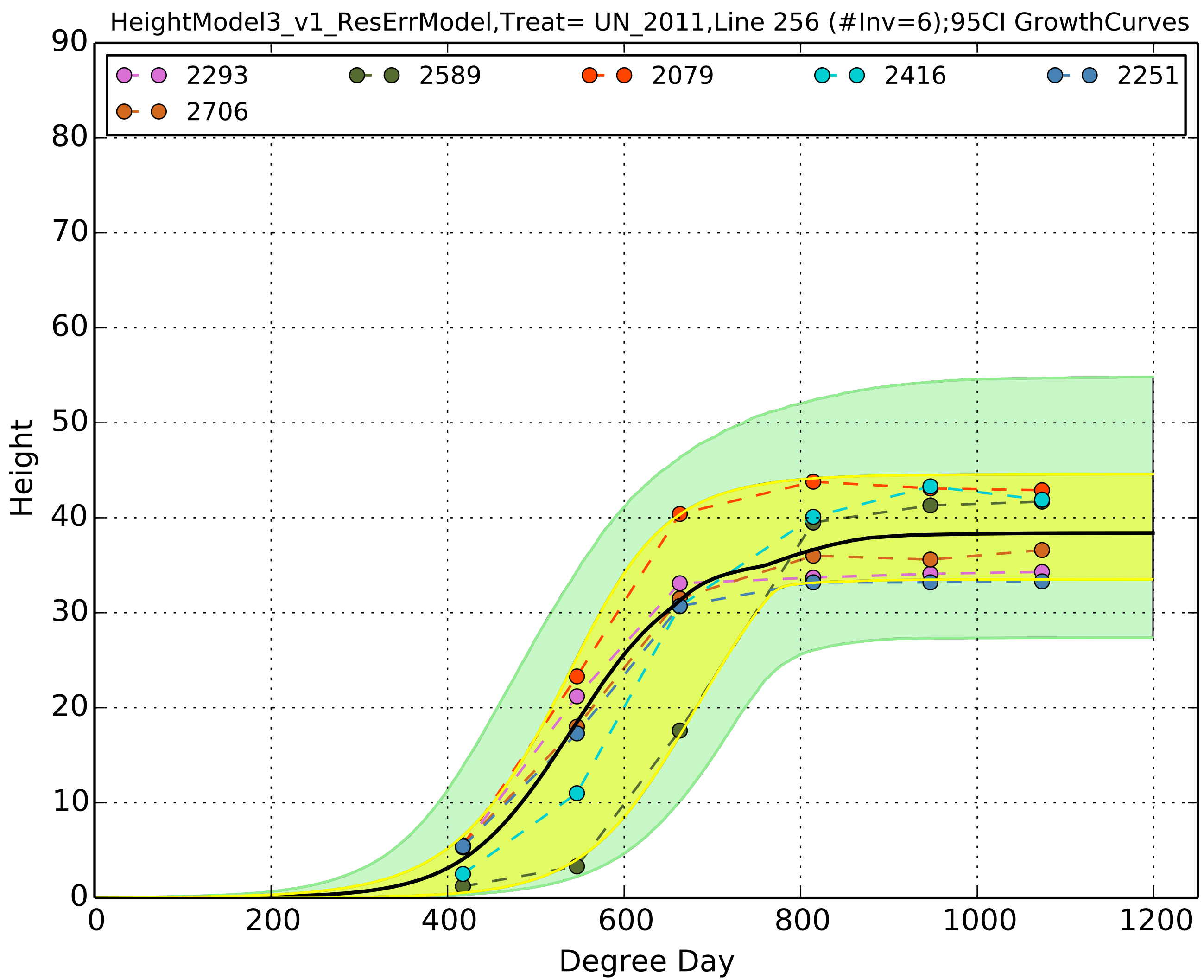
HeightModel3_v1_ResErrModel,Treat= UN_2011,Line 256 (#Inv=6);95CI GrowthCurves
2293
2589
2079
2416
2251
2706
Height
Degree Day
0
10
20
30
40
50
60
70
80
90
0
200
400
600
800
1000
1200

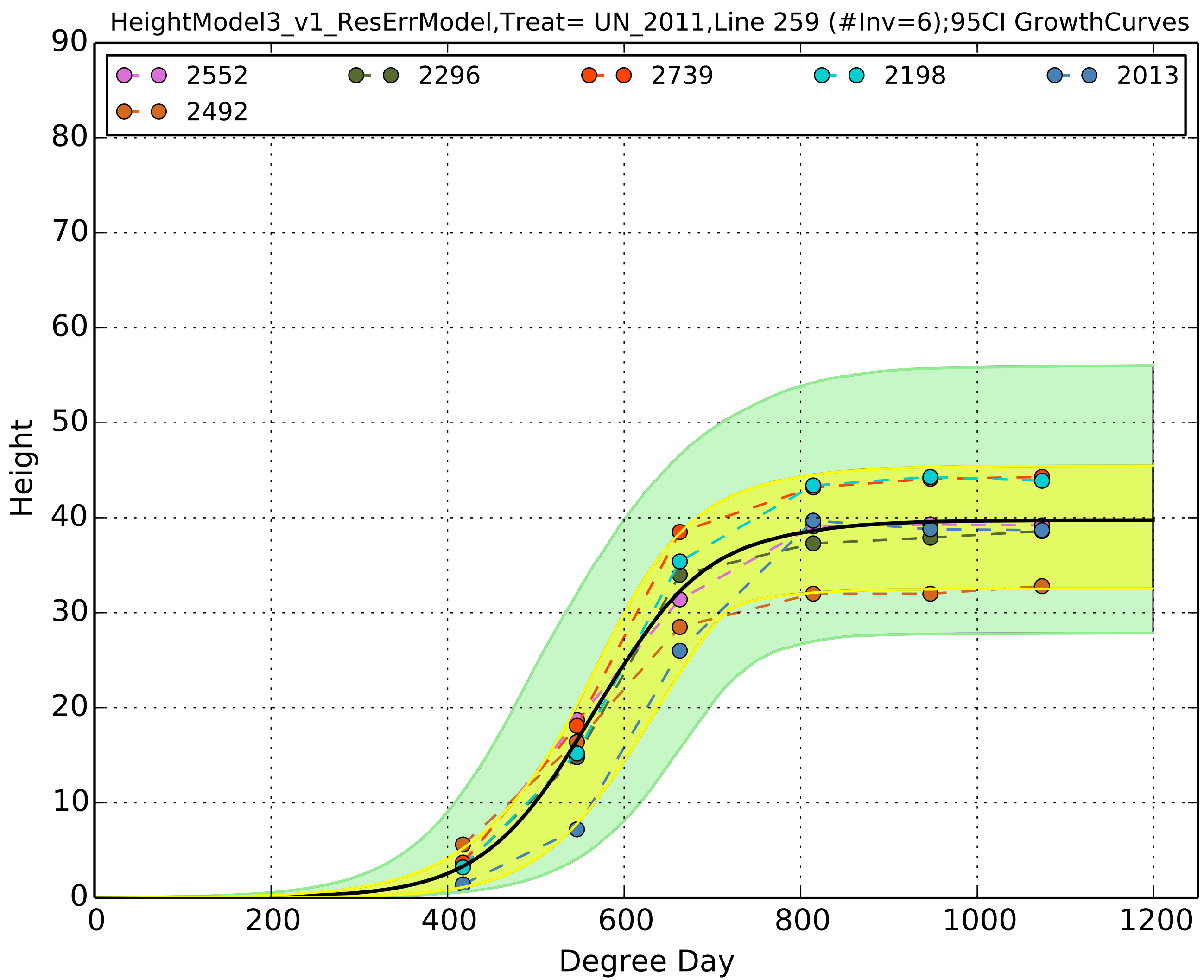
HeightModel3_v1_ResErrModel,Treat= UN_2011,Line 259 (#Inv=6);95CI GrowthCurves
2552
2296
2739
2198
2013
2492
Height
Degree Day
0
10
20
30
40
50
60
70
80
90
0
200
400
600
800
1000
1200

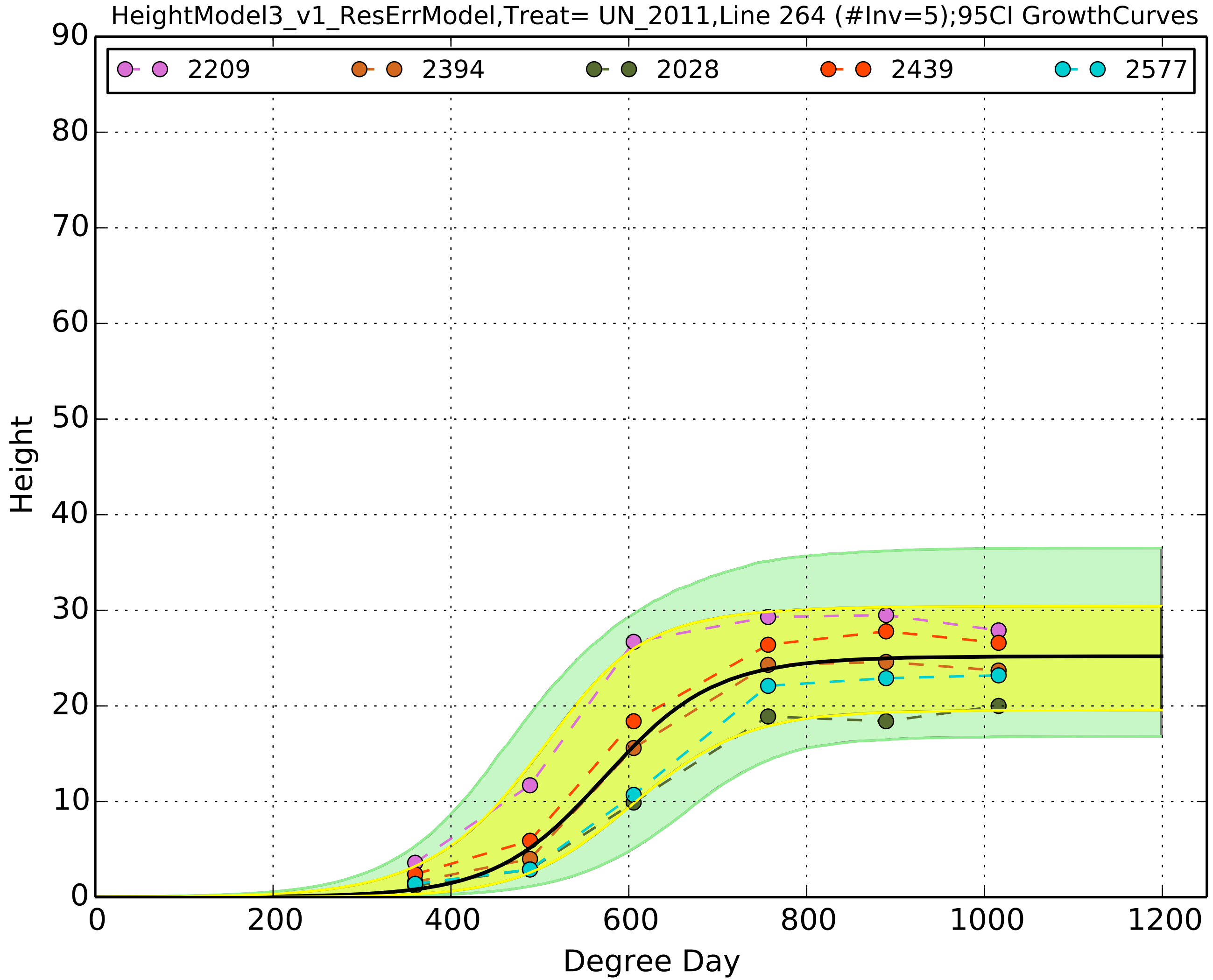

HeightModel3_v1_ResErrModel,Treat= UN_2011,Line 264 (#Inv=5);95CI GrowthCurves
2209
2394
2028
2439
2577
Height
Degree Day
0
10
20
30
40
50
60
70
80
90
0
200
400
600
800
1000
1200

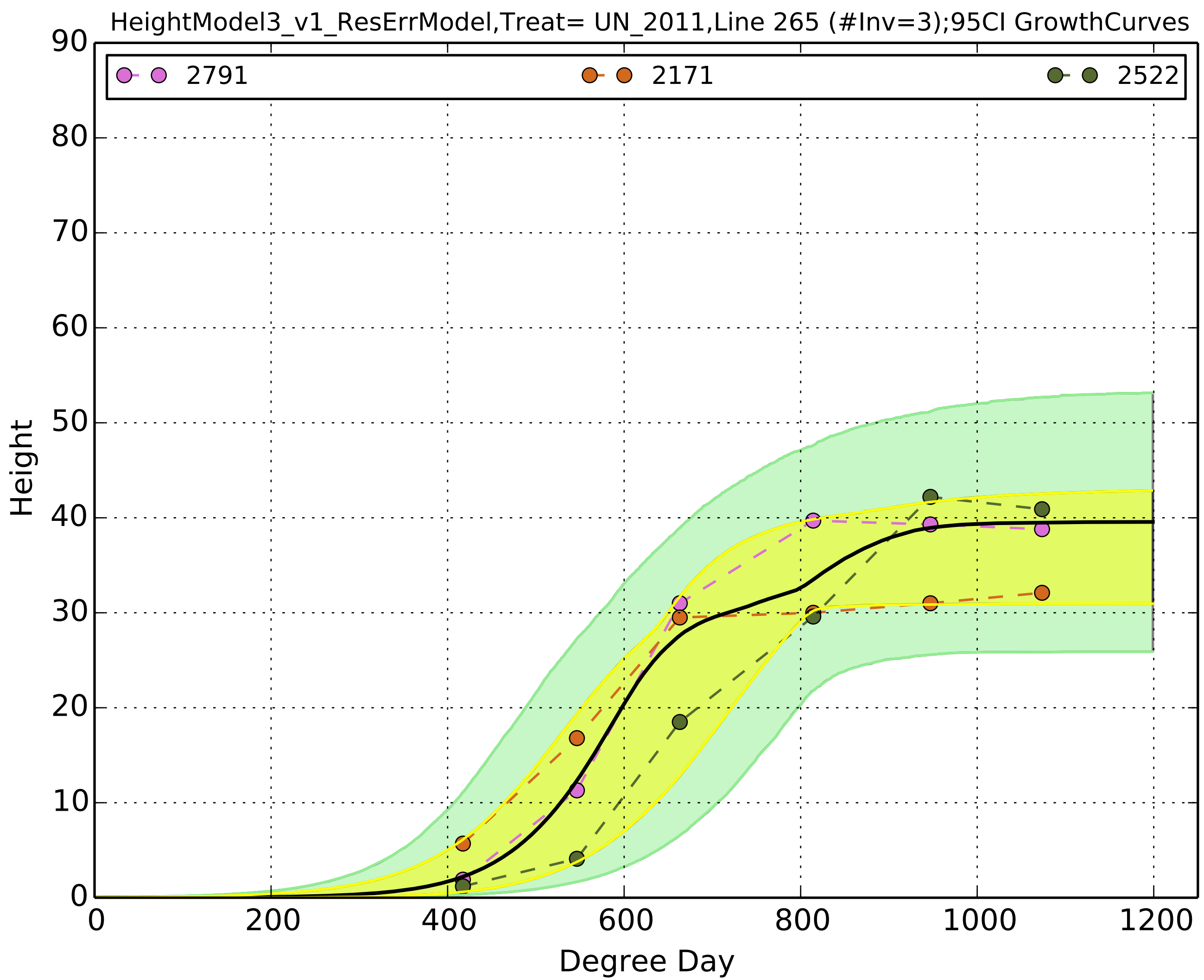
HeightModel3_v1_ResErrModel,Treat= UN_2011,Line 265 (#Inv=3);95CI GrowthCurves
2791
2171
2522
Height
Degree Day
0
10
20
30
40
50
60
70
80
90
0
200
400
600
800
1000
1200

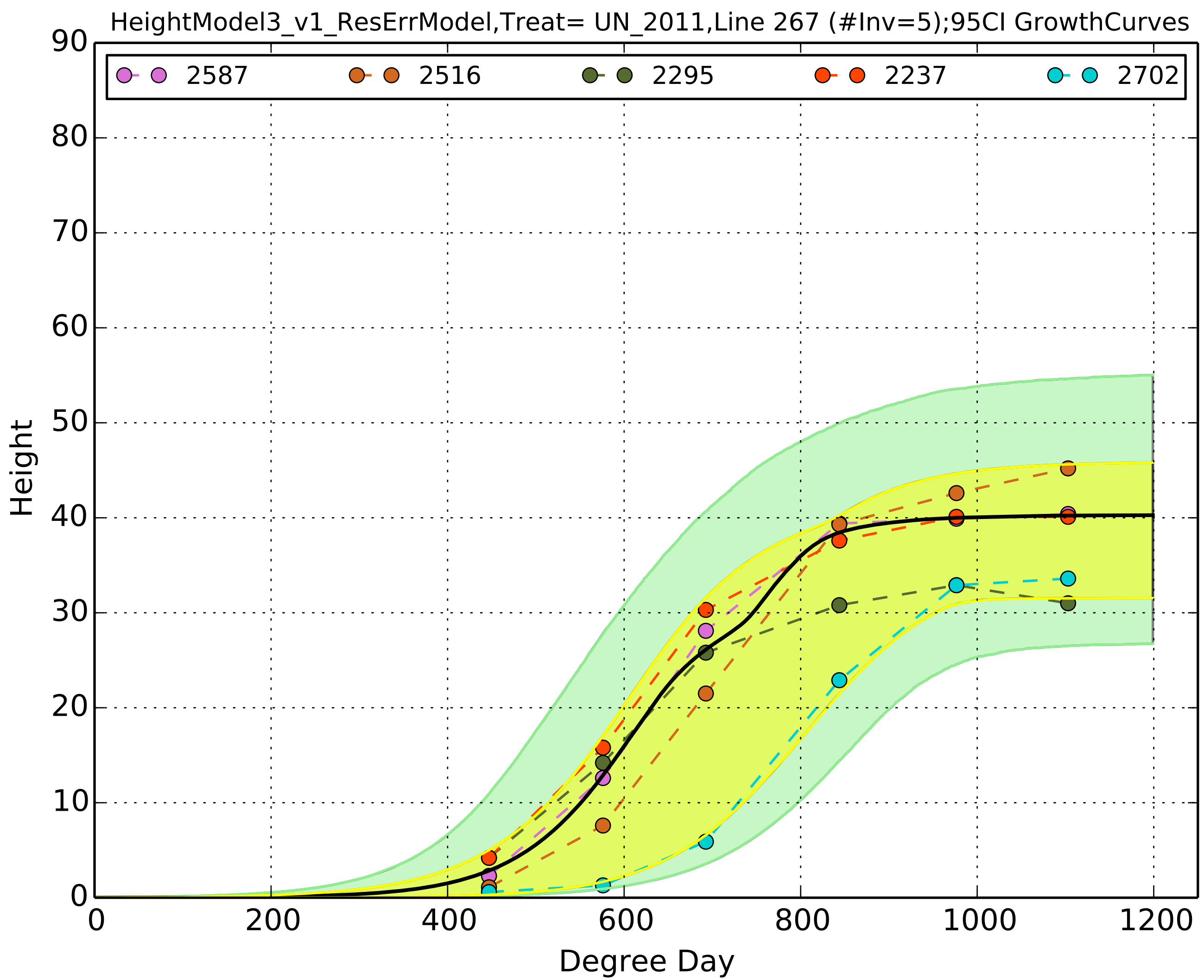
HeightModel3_v1_ResErrModel,Treat= UN_2011,Line 267 (#Inv=5);95CI GrowthCurves
2587
2516
2295
2237
2702
Height
Degree Day
0
10
20
30
40
50
60
70
80
90
0
200
400
600
800
1000
1200

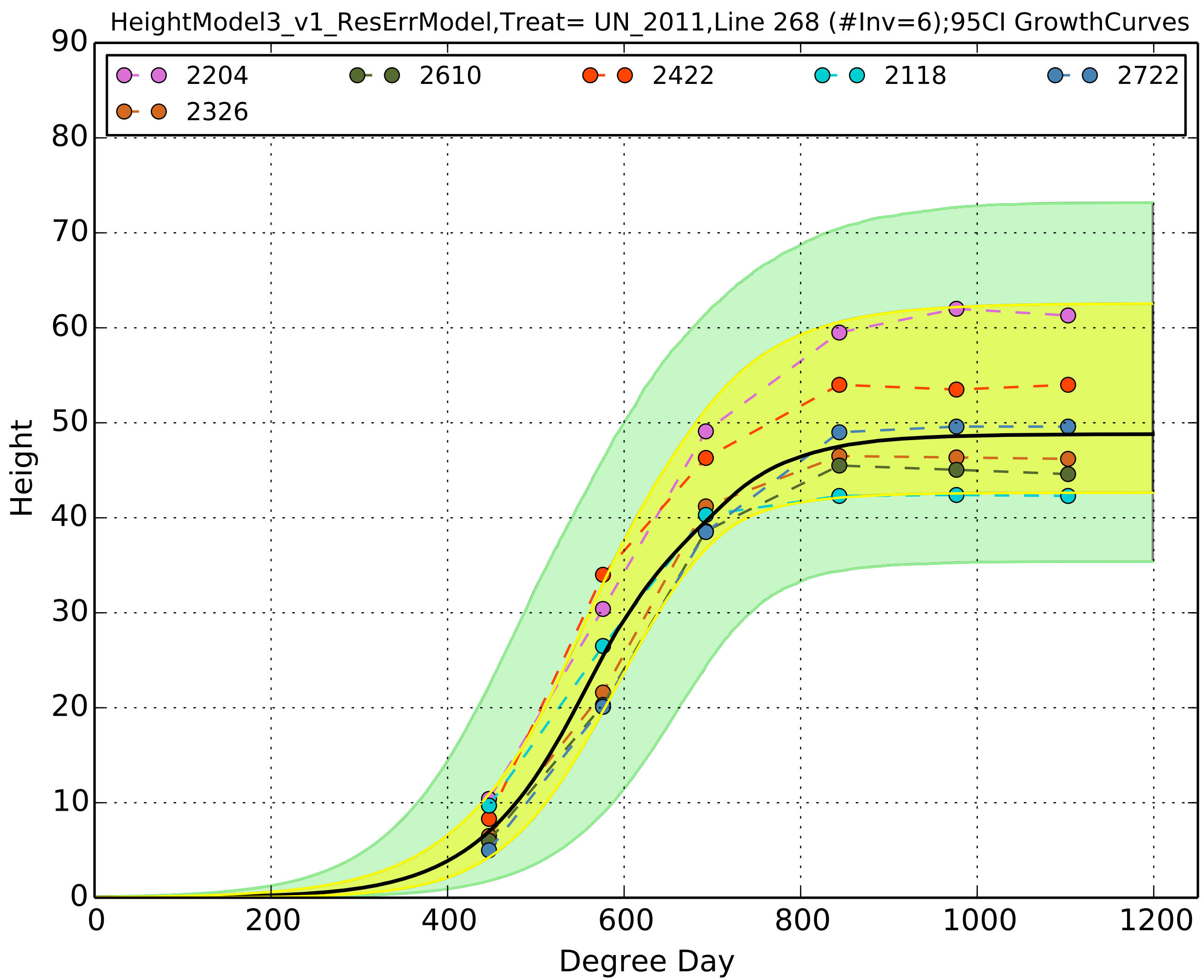
HeightModel3_v1_ResErrModel,Treat= UN_2011,Line 268 (#Inv=6);95CI GrowthCurves
2204
2610
2422
2118
2722
2326
Height
Degree Day
0
10
20
30
40
50
60
70
80
90
0
200
400
600
800
1000
1200

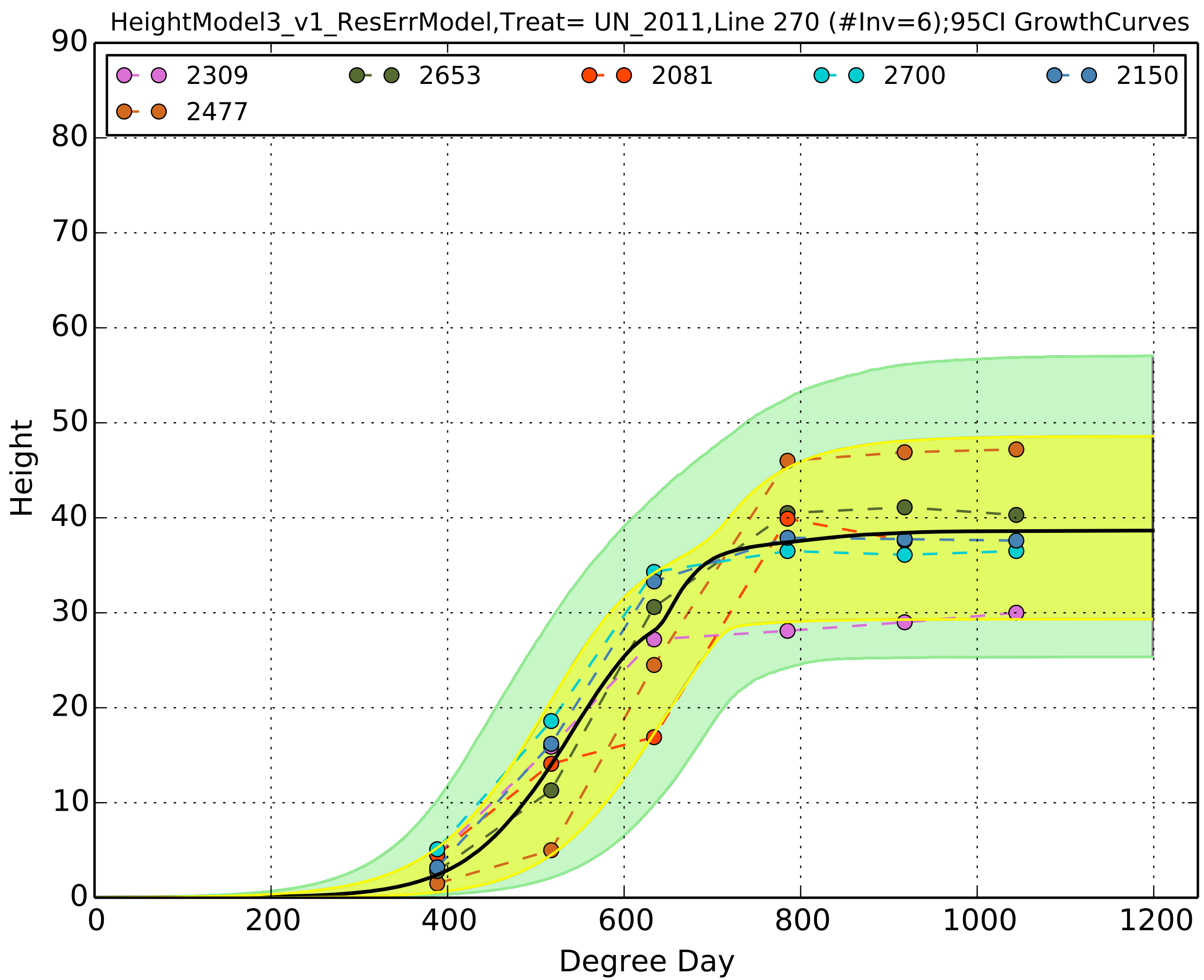
HeightModel3_v1_ResErrModel,Treat= UN_2011,Line 270 (#Inv=6);95CI GrowthCurves
2309
2653
2081
2700
2150
2477
Height
Degree Day
0
10
20
30
40
50
60
70
80
90
0
200
400
600
800
1000
1200

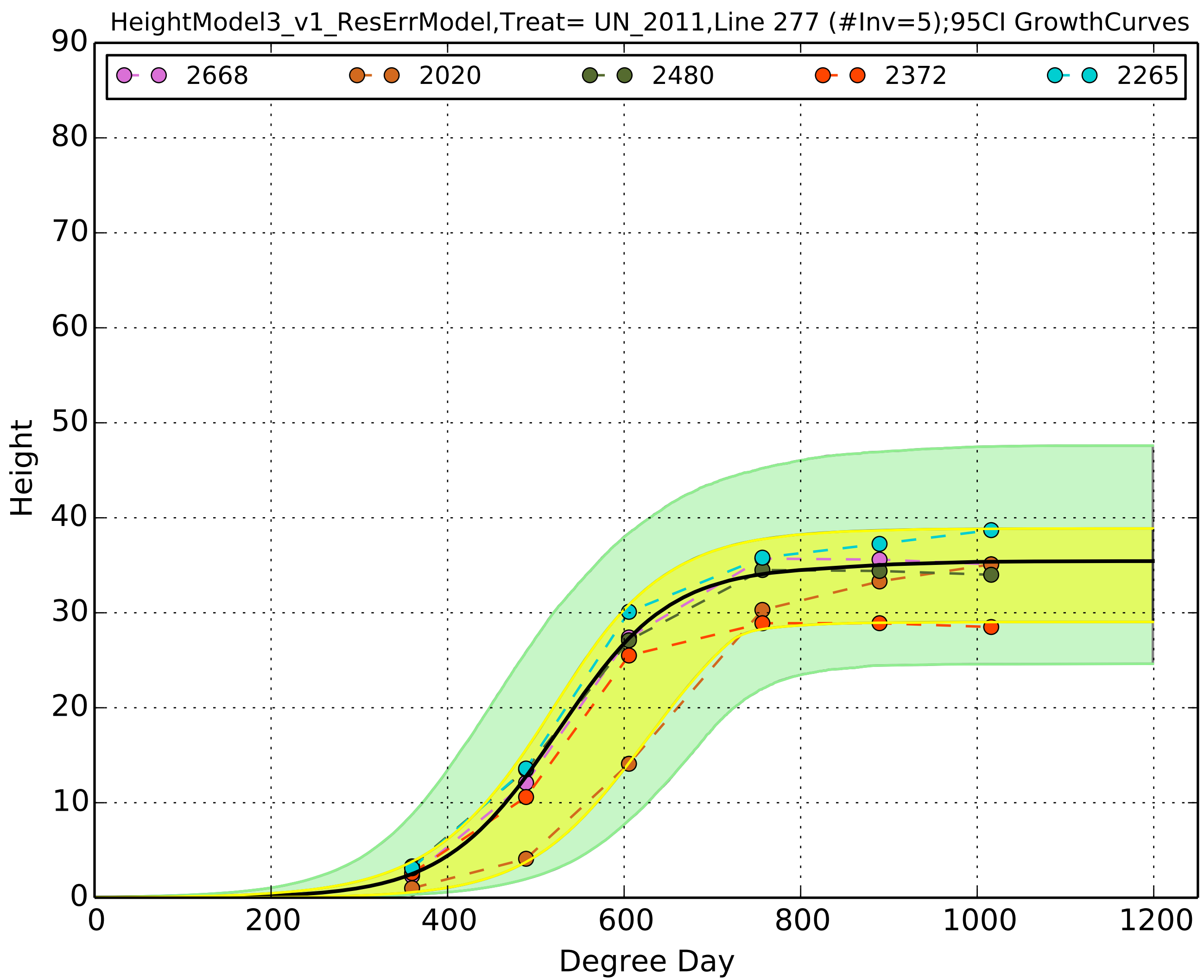

HeightModel3_v1_ResErrModel,Treat= UN_2011,Line 277 (#Inv=5);95CI GrowthCurves
2668
2020
2480
2372
2265
Height
Degree Day
0
10
20
30
40
50
60
70
80
90
0
200
400
600
800
1000
1200

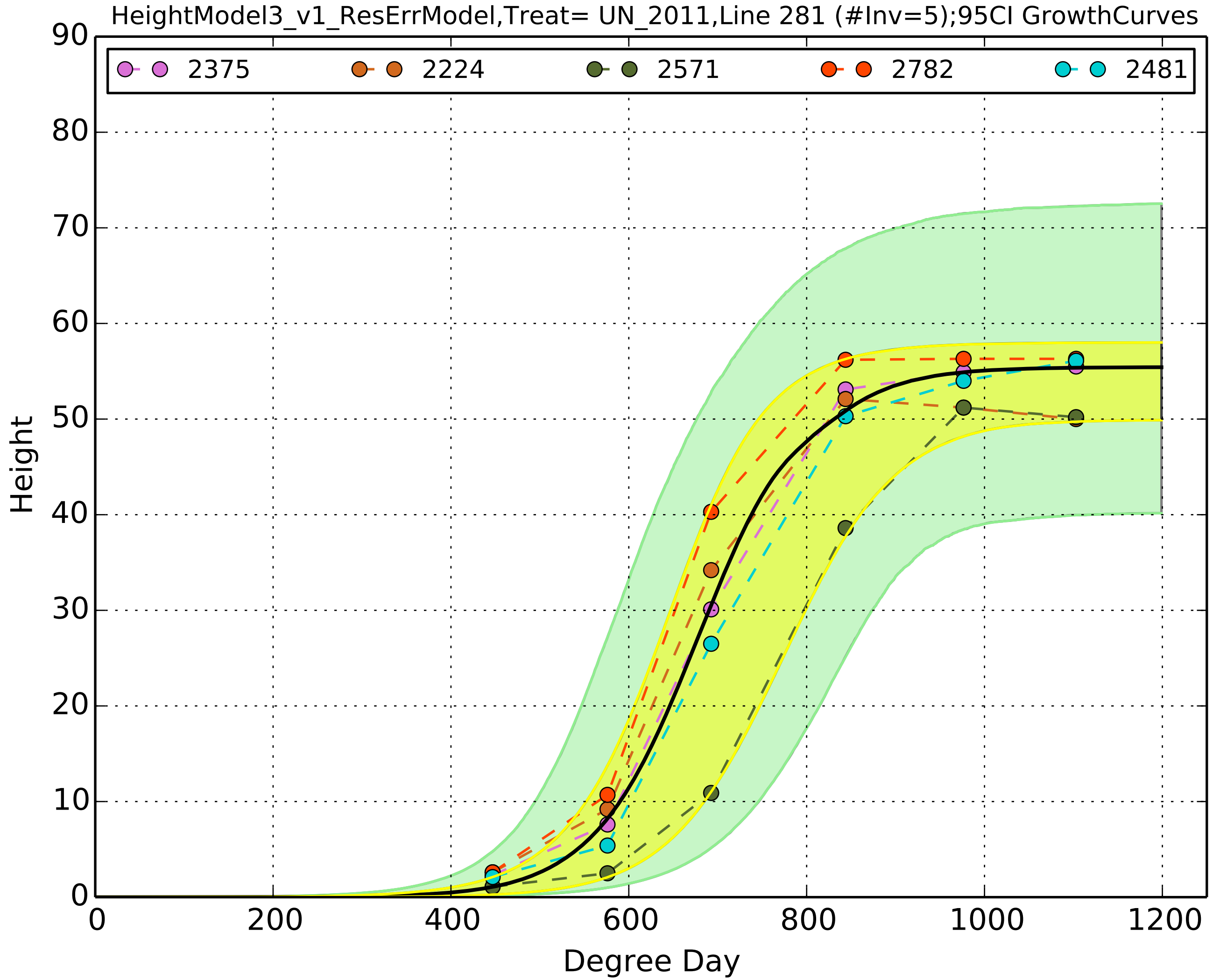

HeightModel3_v1_ResErrModel,Treat= UN_2011,Line 281 (#Inv=5);95CI GrowthCurves
2375
2224
2571
2782
2481
Height
Degree Day
0
10
20
30
40
50
60
70
80
90
0
200
400
600
800
1000
1200

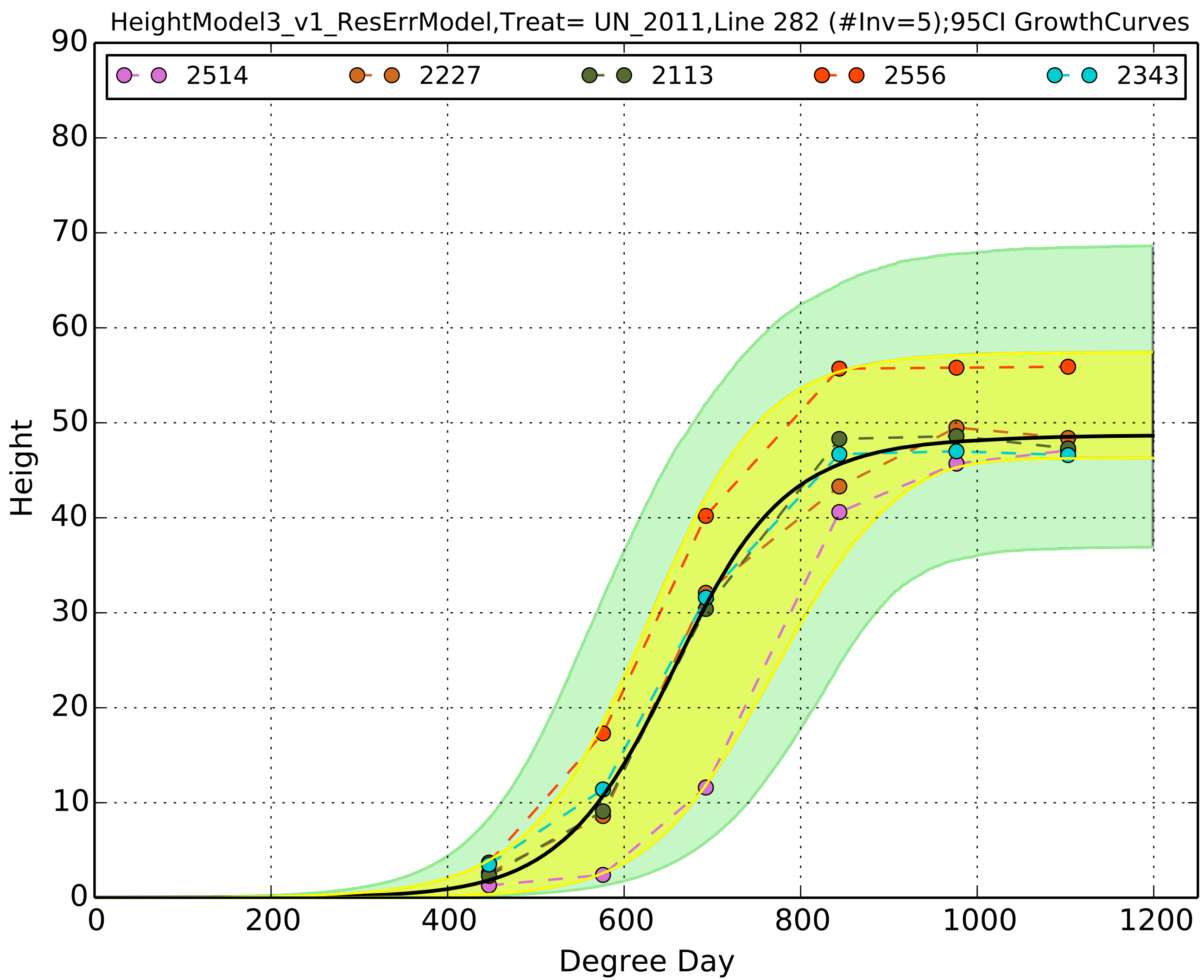
HeightModel3_v1_ResErrModel,Treat= UN_2011,Line 282 (#Inv=5);95CI GrowthCurves
2514
2227
2113
2556
2343
Height
Degree Day
0
10
20
30
40
50
60
70
80
90
0
200
400
600
800
1000
1200

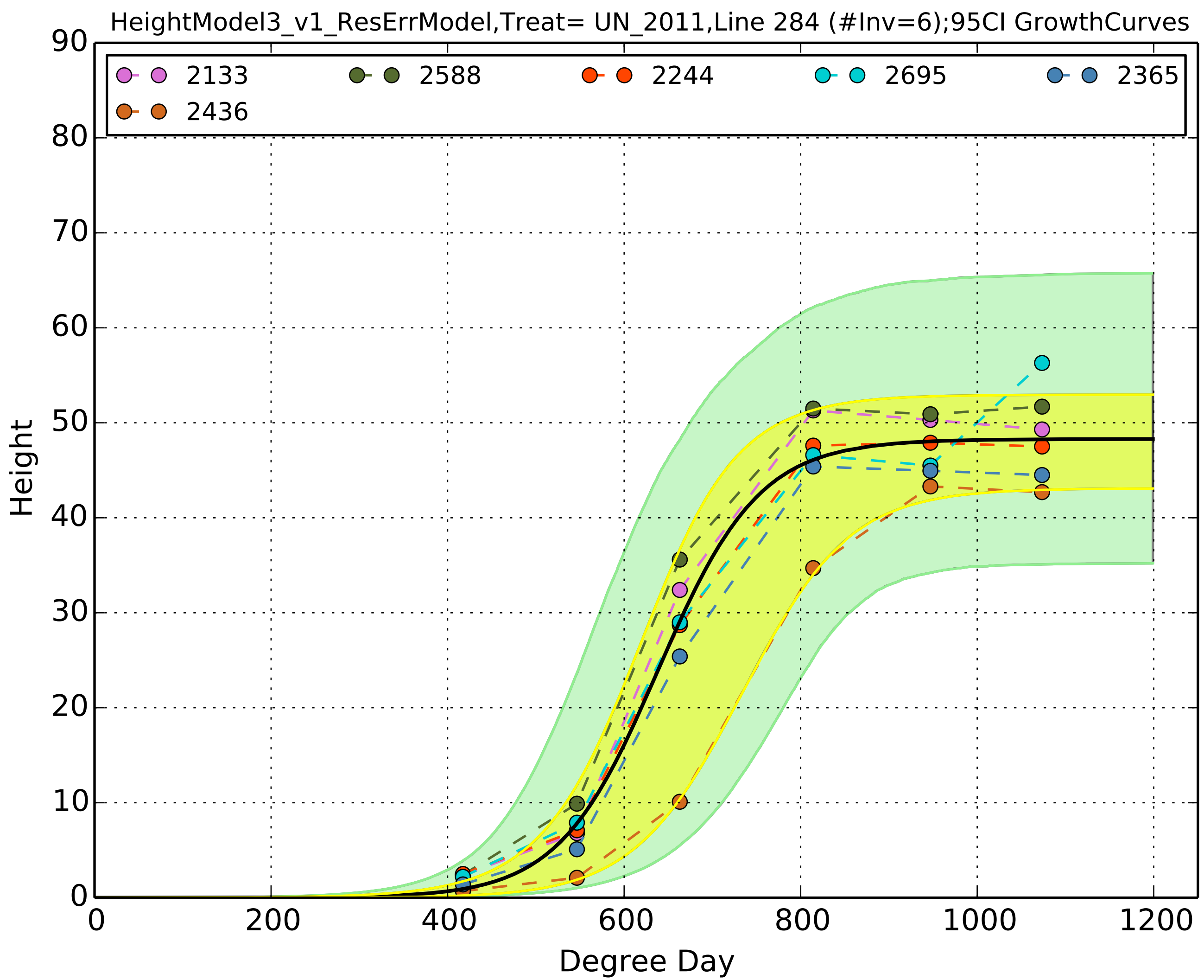

HeightModel3_v1_ResErrModel,Treat= UN_2011,Line 284 (#Inv=6);95CI GrowthCurves
2133
2588
2244
2695
2365
2436
Height
Degree Day
0
10
20
30
40
50
60
70
80
90
0
200
400
600
800
1000
1200

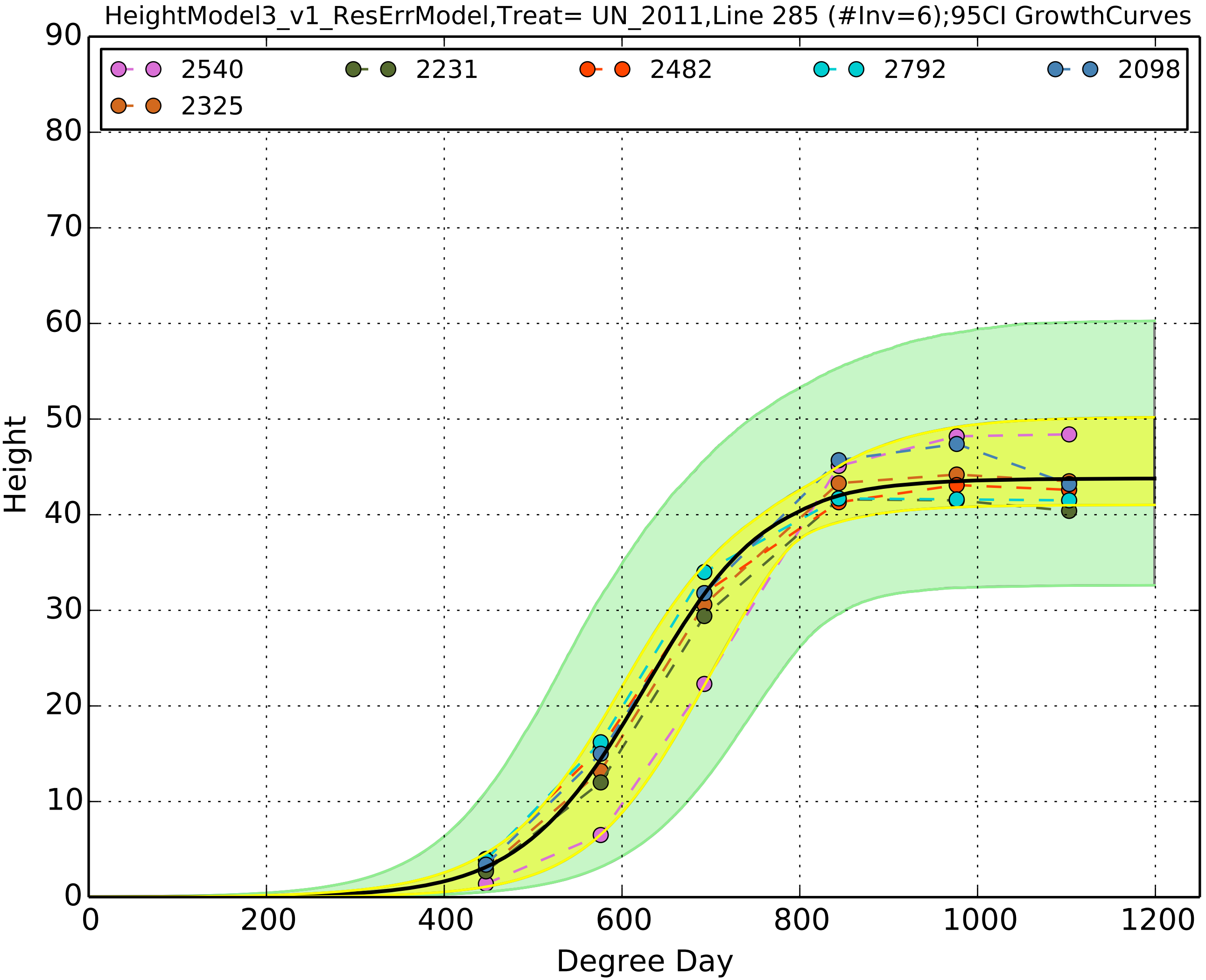
HeightModel3_v1_ResErrModel,Treat= UN_2011,Line 285 (#Inv=6);95CI GrowthCurves
2540
2231
2482
2792
2098
2325
Height
Degree Day
0
10
20
30
40
50
60
70
80
90
0
200
400
600
800
1000
1200

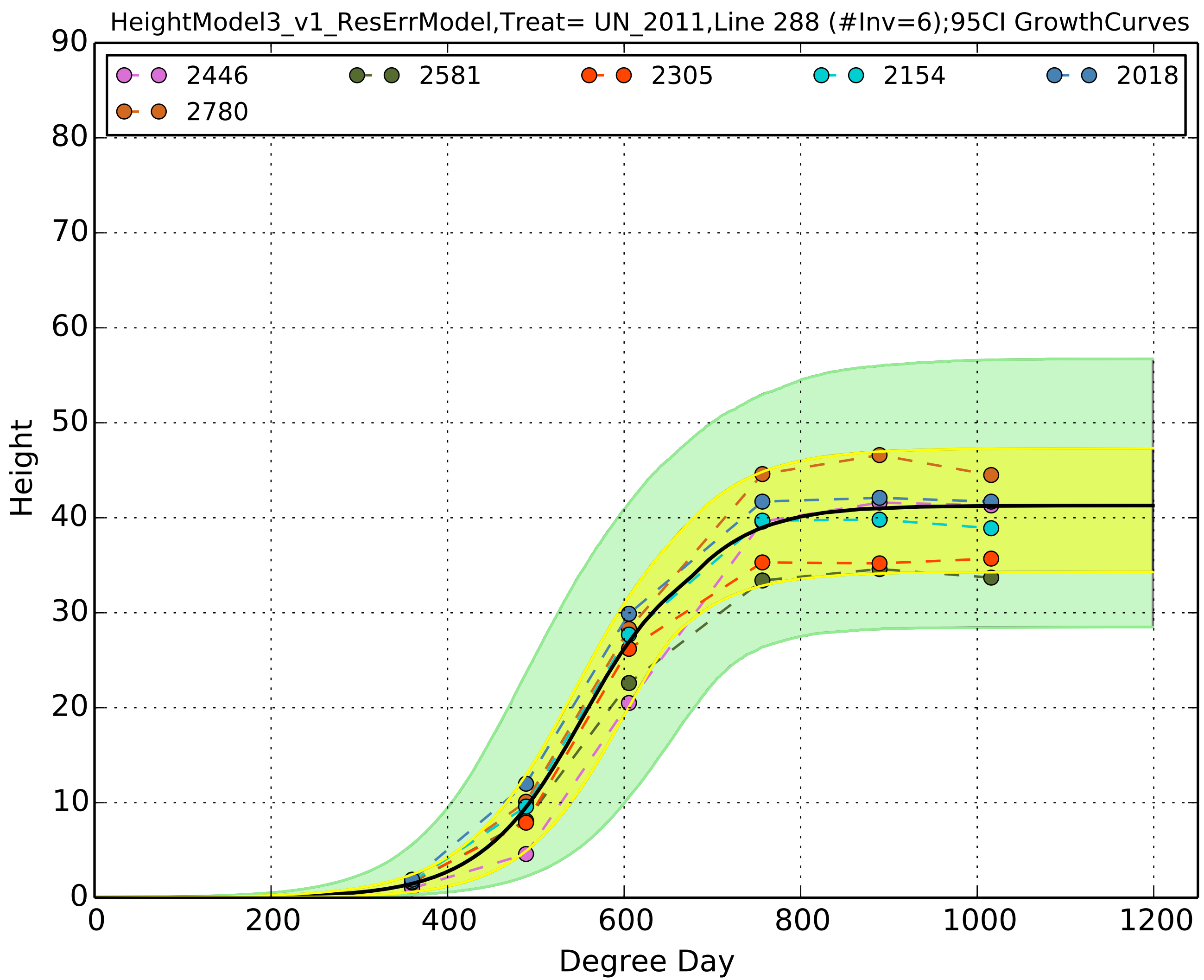
HeightModel3_v1_ResErrModel,Treat= UN_2011,Line 288 (#Inv=6);95CI GrowthCurves
2446
2581
2305
2154
2018
2780
Height
Degree Day
0
10
20
30
40
50
60
70
80
90
0
200
400
600
800
1000
1200

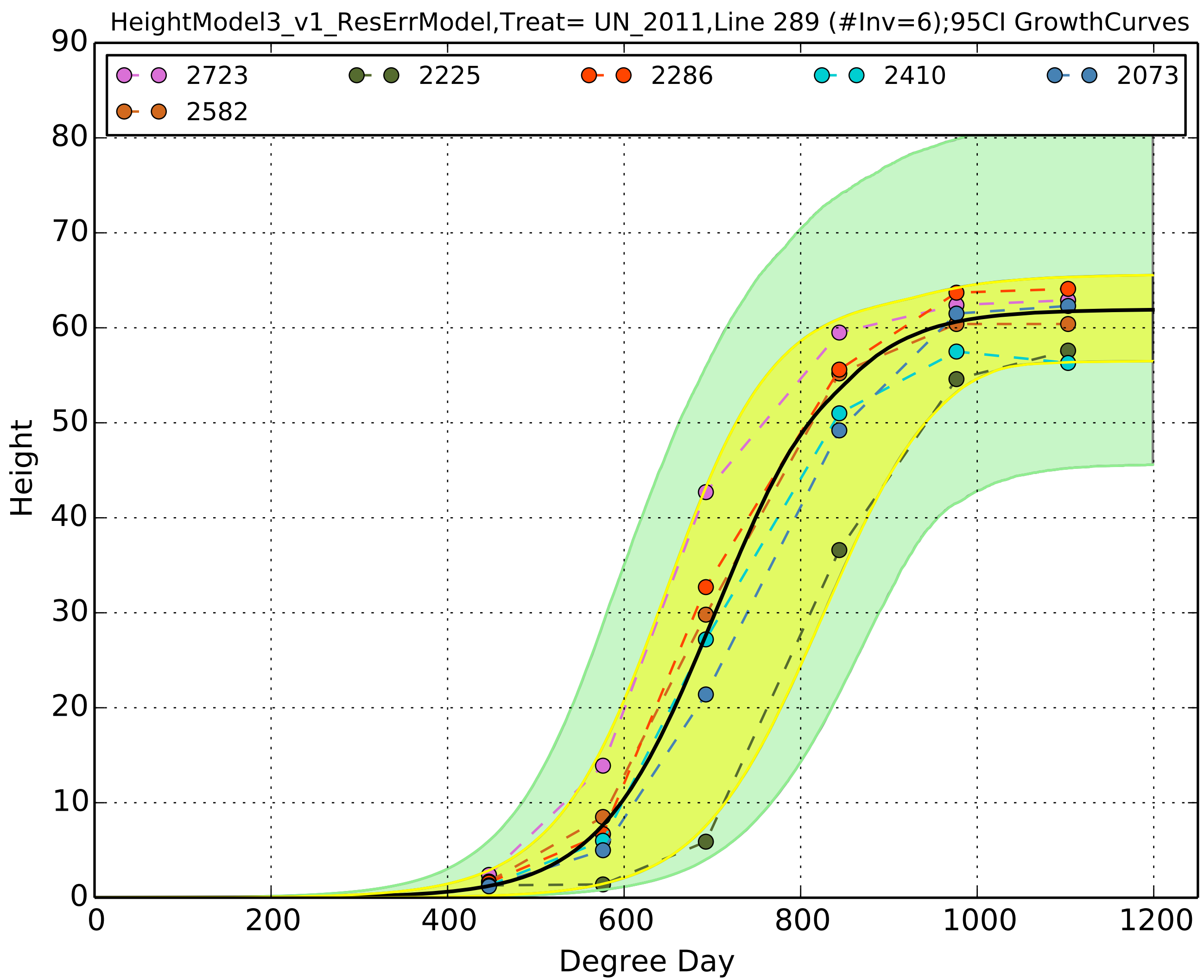

HeightModel3_v1_ResErrModel,Treat= UN_2011,Line 289 (#Inv=6);95CI GrowthCurves
2723
2225
2286
2410
2073
2582
Height
Degree Day
0
10
20
30
40
50
60
70
80
90
0
200
400
600
800
1000
1200

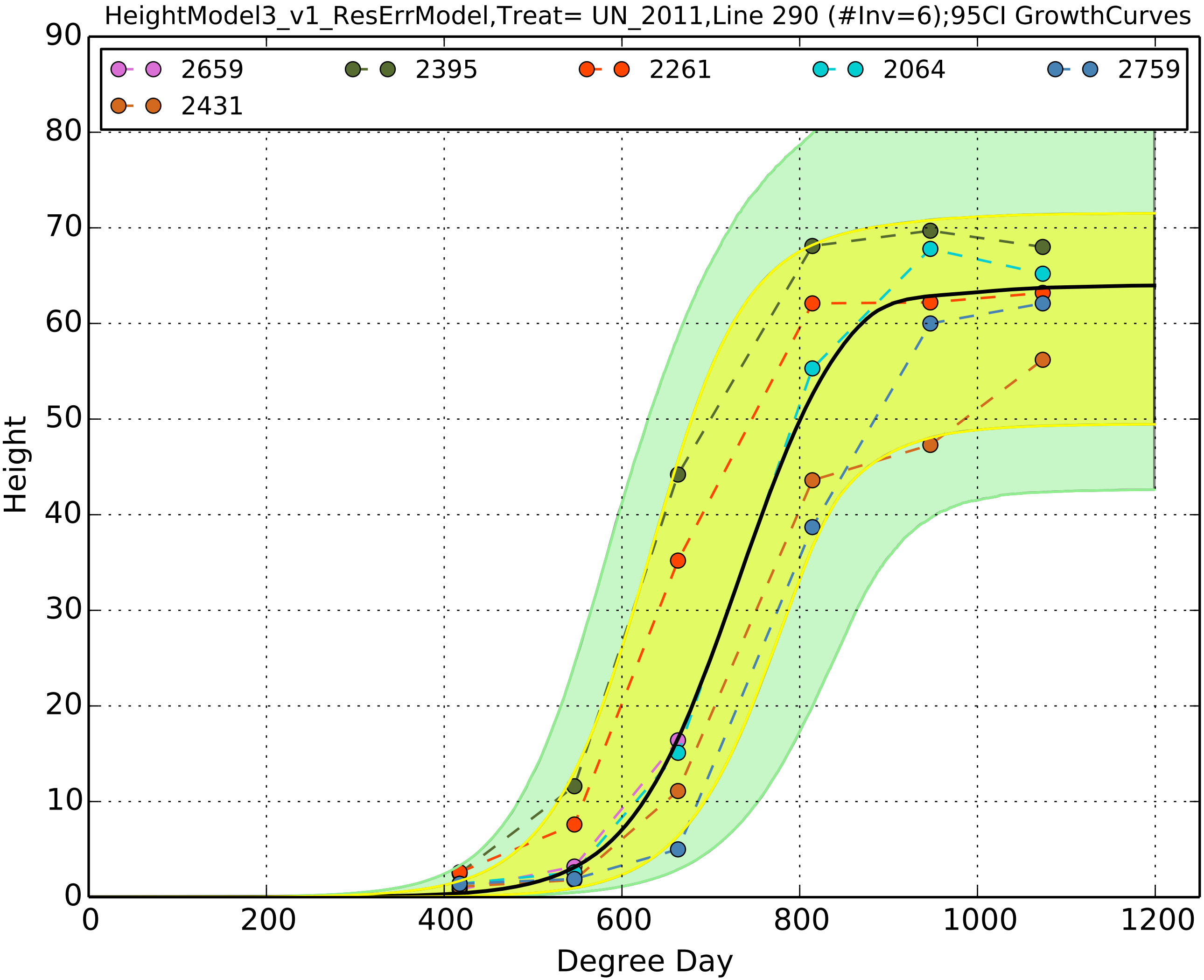

HeightModel3_v1_ResErrModel,Treat= UN_2011,Line 290 (#Inv=6);95CI GrowthCurves
2659
2395
2261
2064
2759
2431
Height
Degree Day
0
10
20
30
40
50
60
70
80
90
0
200
400
600
800
1000
1200

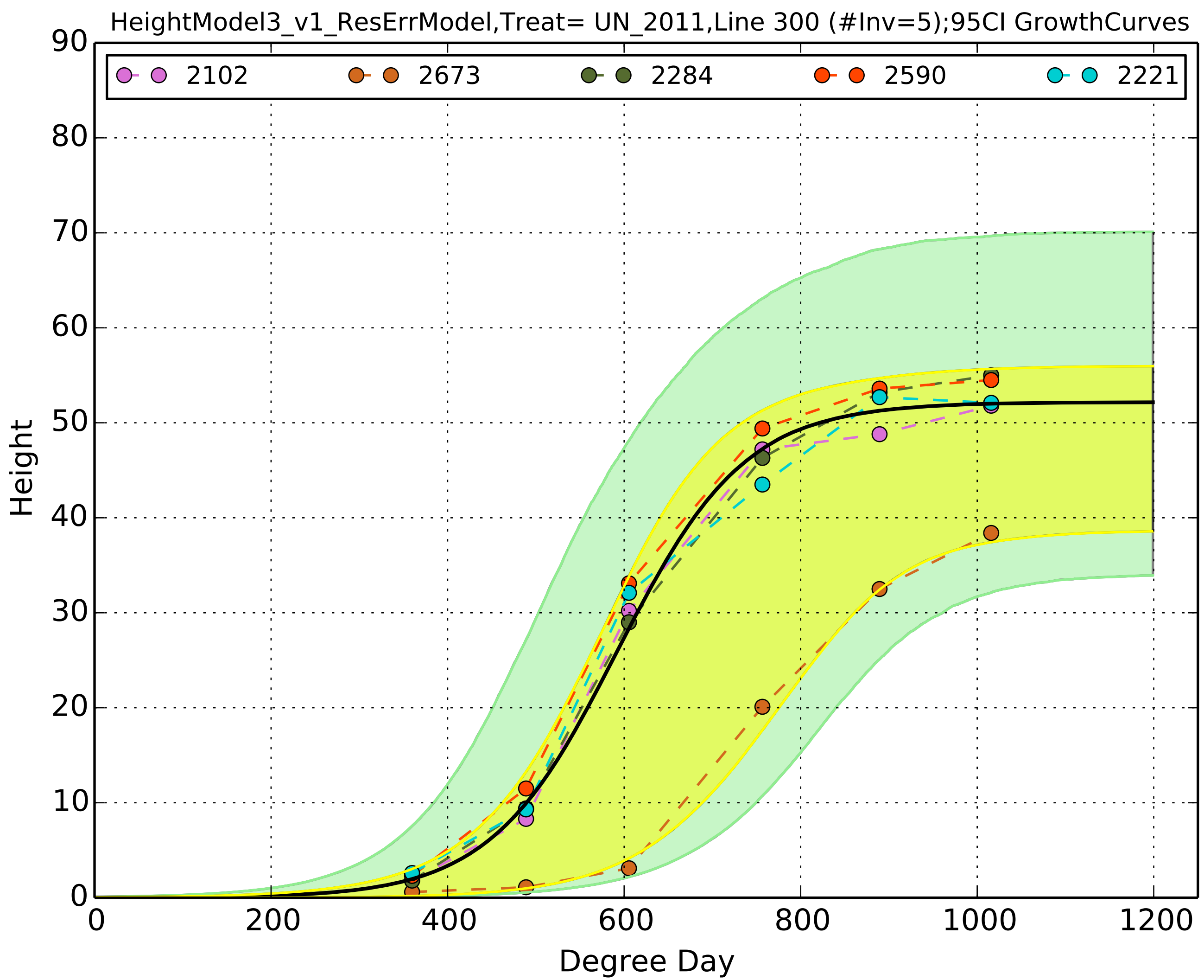

HeightModel3_v1_ResErrModel,Treat= UN_2011,Line 300 (#Inv=5);95CI GrowthCurves
2102
2673
2284
2590
2221
Height
Degree Day
0
10
20
30
40
50
60
70
80
90
0
200
400
600
800
1000
1200

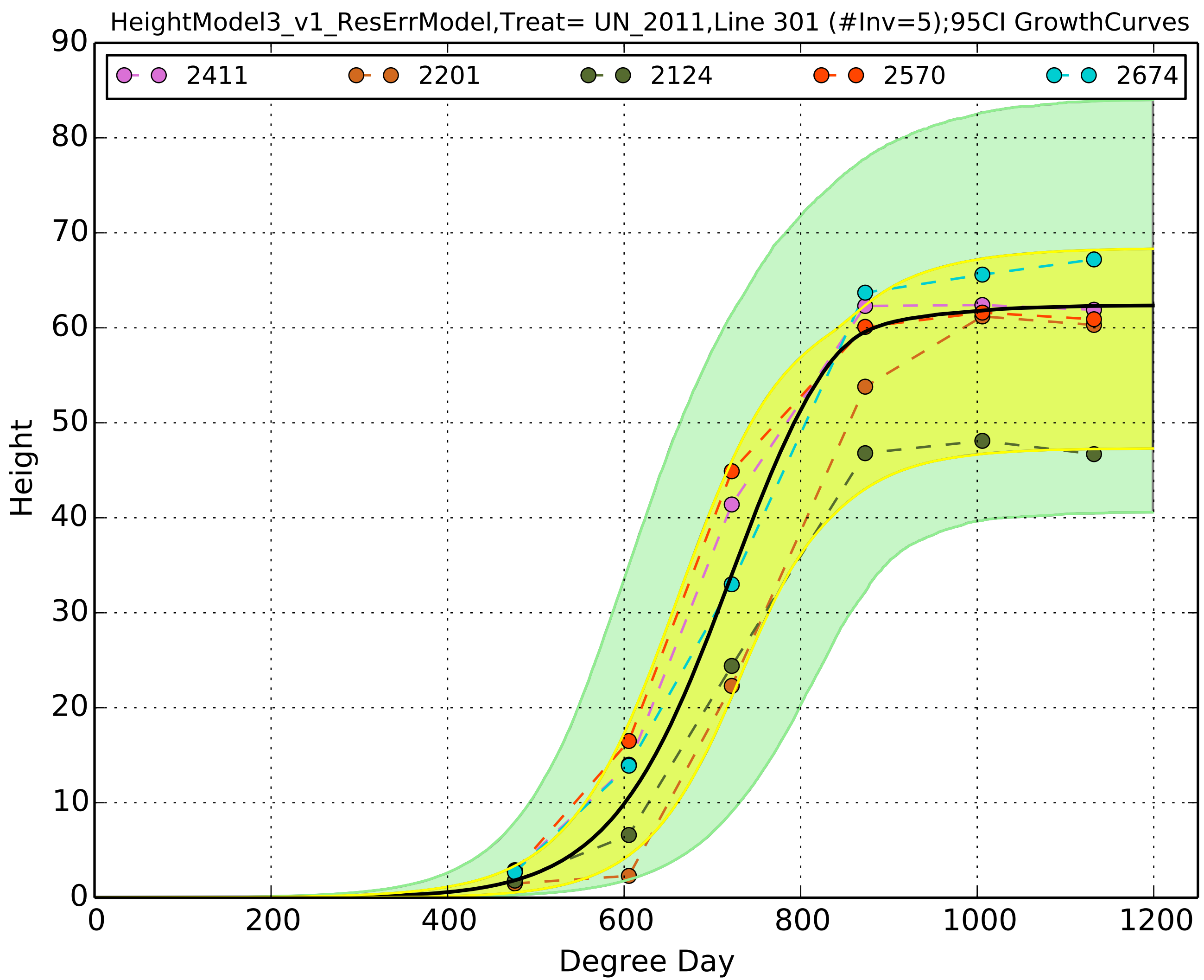

HeightModel3_v1_ResErrModel,Treat= UN_2011,Line 301 (#Inv=5);95CI GrowthCurves
2411
2201
2124
2570
2674
Height
Degree Day
0
10
20
30
40
50
60
70
80
90
0
200
400
600
800
1000
1200

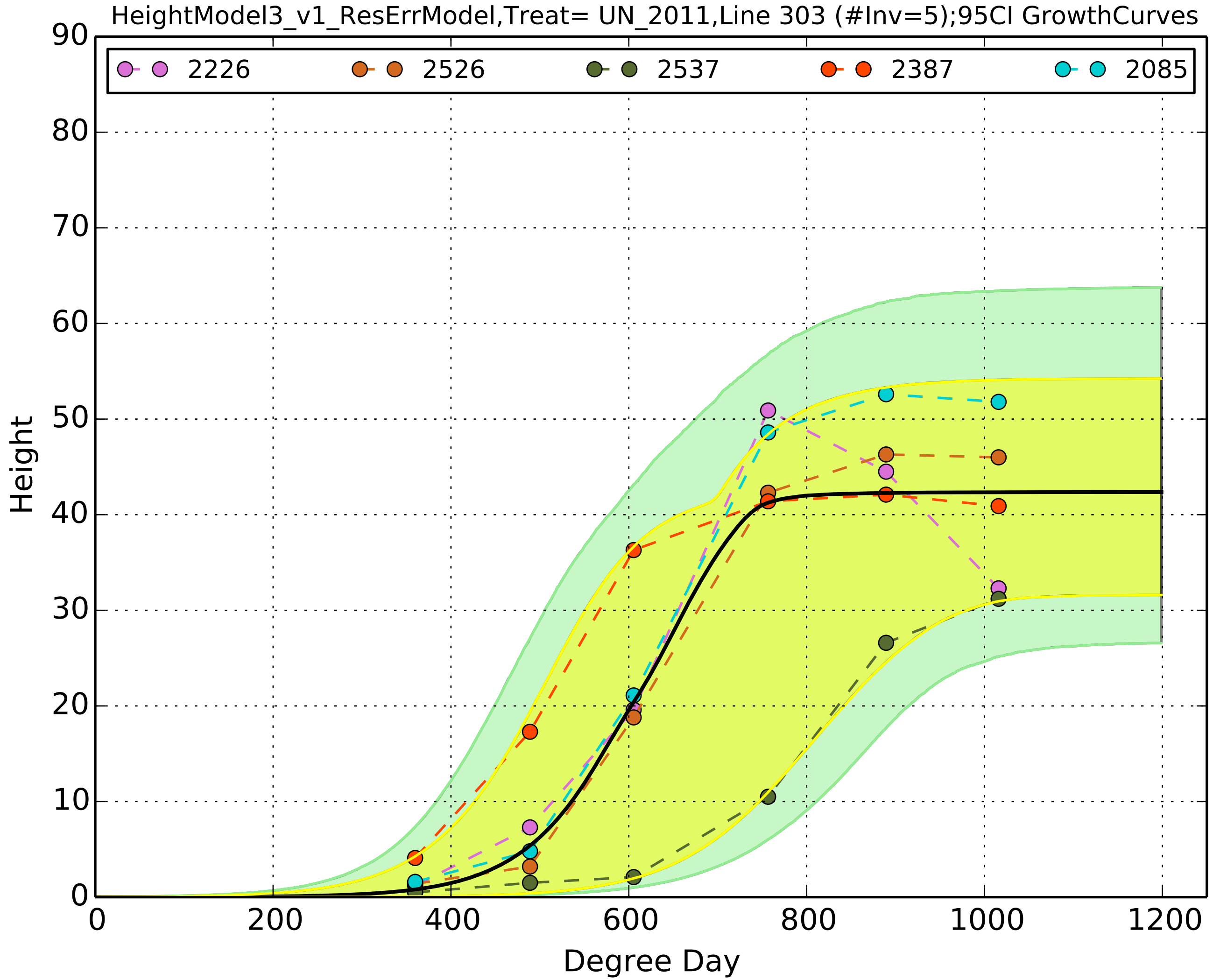
HeightModel3_v1_ResErrModel,Treat= UN_2011,Line 303 (#Inv=5);95CI GrowthCurves
2226
2526
2537
2387
2085
Height
Degree Day
0
10
20
30
40
50
60
70
80
90
0
200
400
600
800
1000
1200

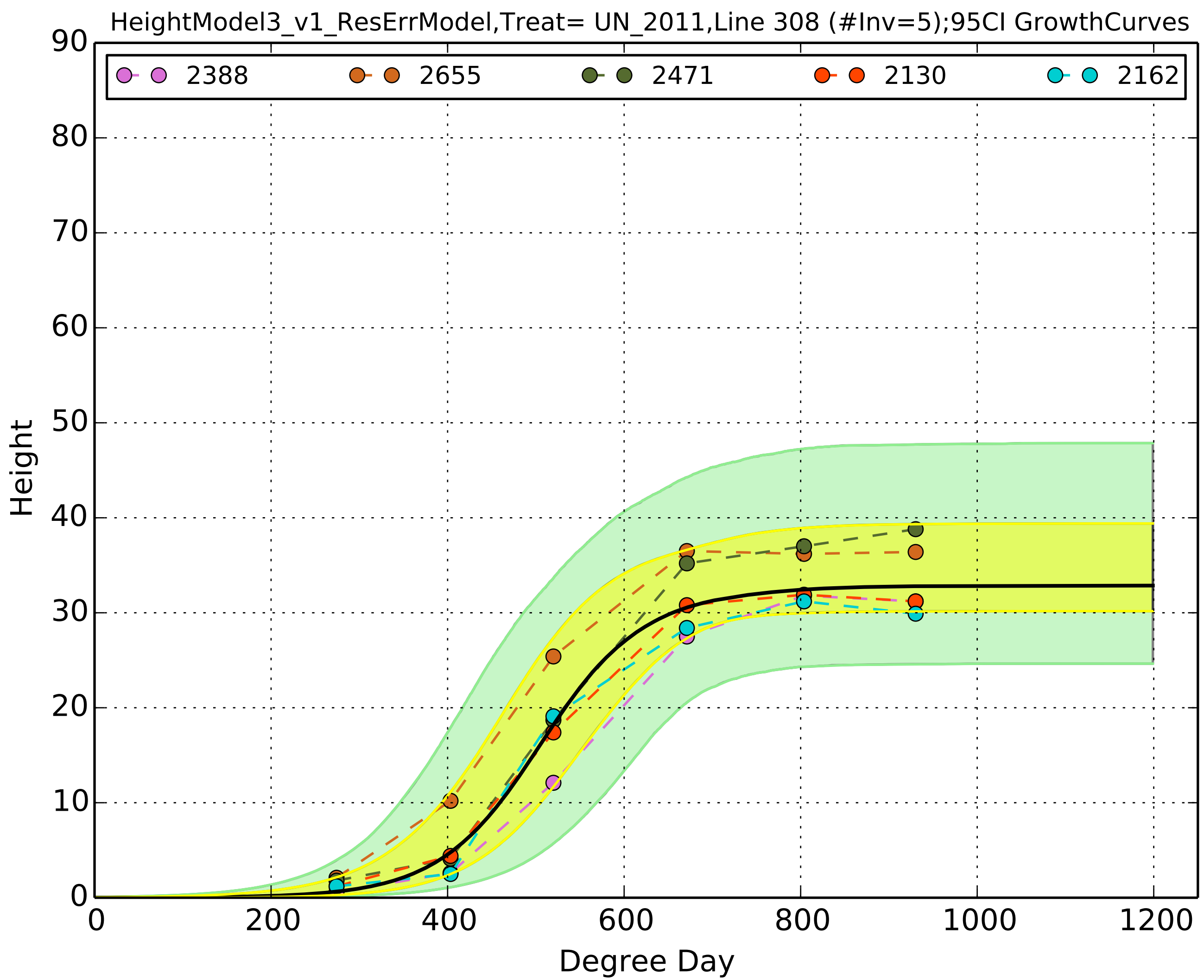
HeightModel3_v1_ResErrModel,Treat= UN_2011,Line 308 (#Inv=5);95CI GrowthCurves
2388
2655
2471
2130
2162
Height
Degree Day
0
10
20
30
40
50
60
70
80
90
0
200
400
600
800
1000
1200

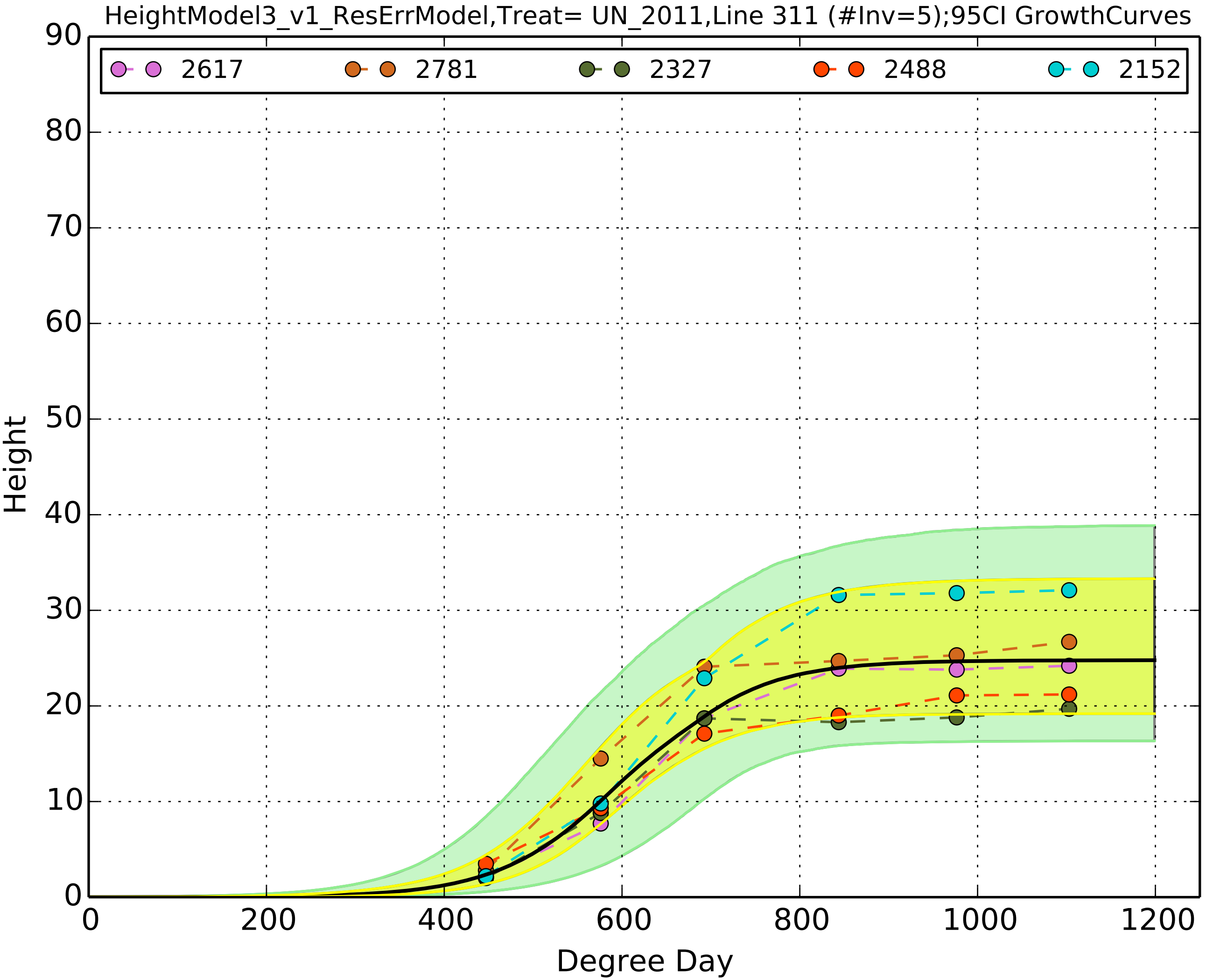

HeightModel3_v1_ResErrModel,Treat= UN_2011,Line 311 (#Inv=5);95CI GrowthCurves
2617
2781
2327
2488
2152
Height
Degree Day
0
10
20
30
40
50
60
70
80
90
0
200
400
600
800
1000
1200

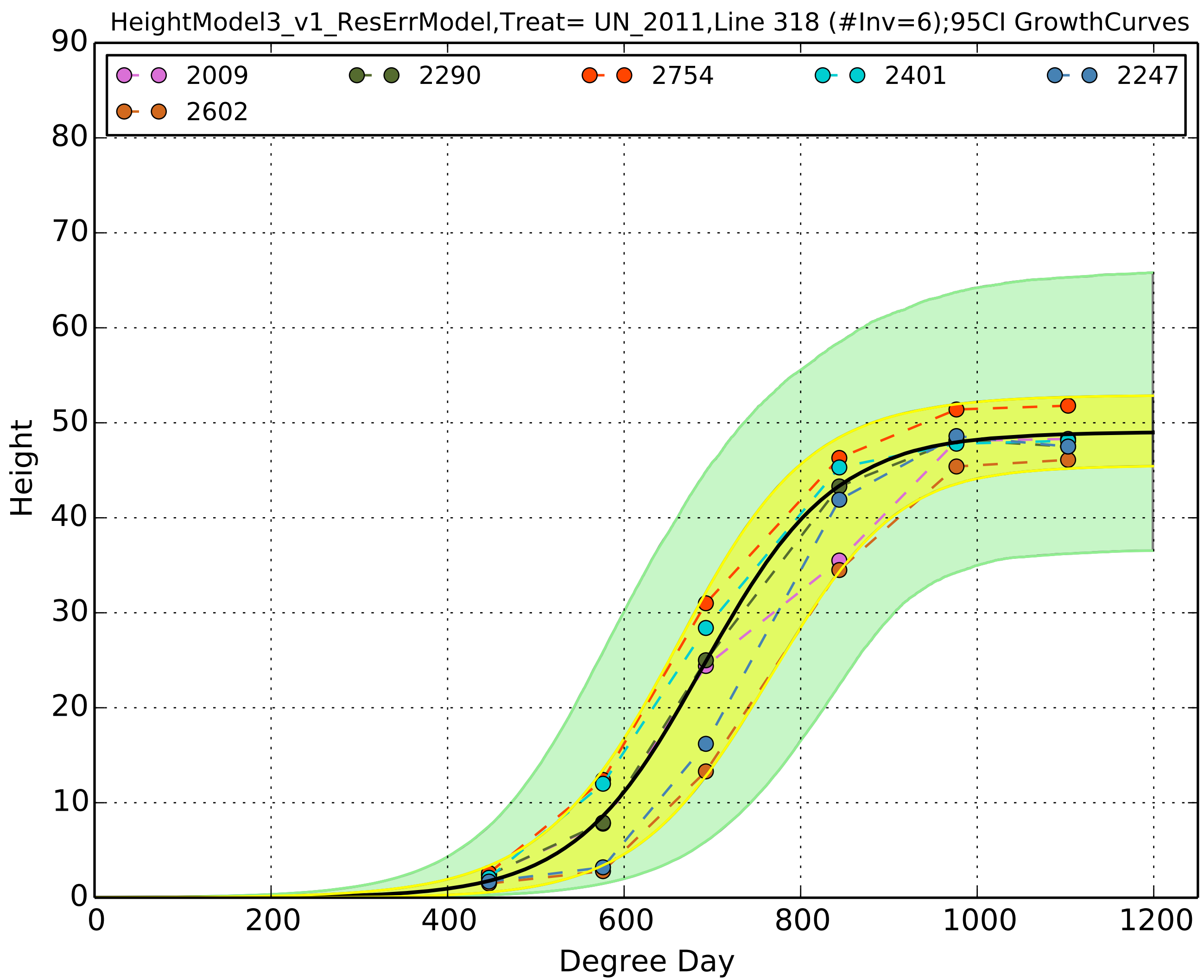

HeightModel3_v1_ResErrModel,Treat= UN_2011,Line 318 (#Inv=6);95CI GrowthCurves
2009
2290
2754
2401
2247
2602
Height
Degree Day
0
10
20
30
40
50
60
70
80
90
0
200
400
600
800
1000
1200

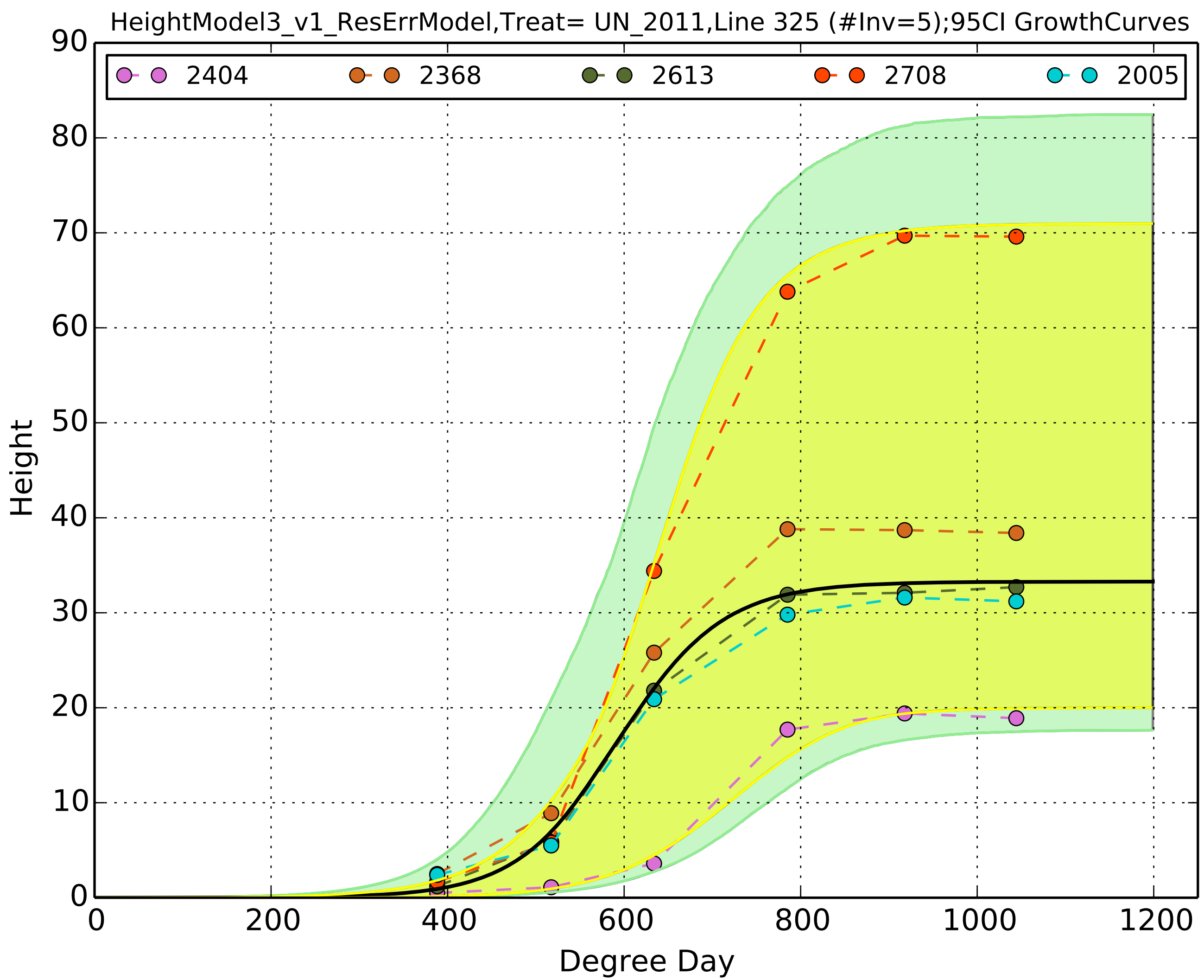

HeightModel3_v1_ResErrModel,Treat= UN_2011,Line 325 (#Inv=5);95CI GrowthCurves
2404
2368
2613
2708
2005
Height
Degree Day
0
10
20
30
40
50
60
70
80
90
0
200
400
600
800
1000
1200

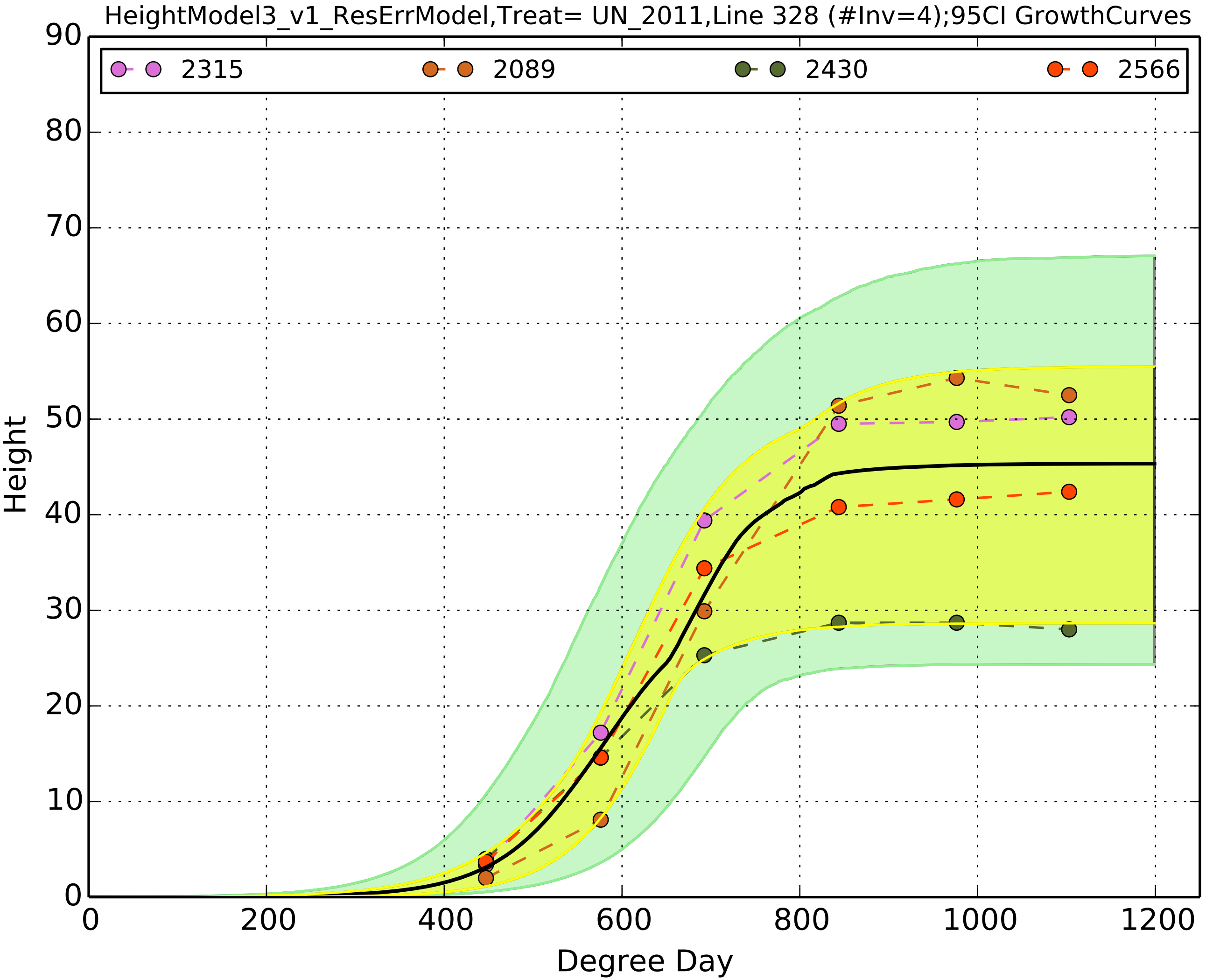
HeightModel3_v1_ResErrModel,Treat= UN_2011,Line 328 (#Inv=4);95CI GrowthCurves
2315
2089
2430
2566
Height
Degree Day
0
10
20
30
40
50
60
70
80
90
0
200
400
600
800
1000
1200

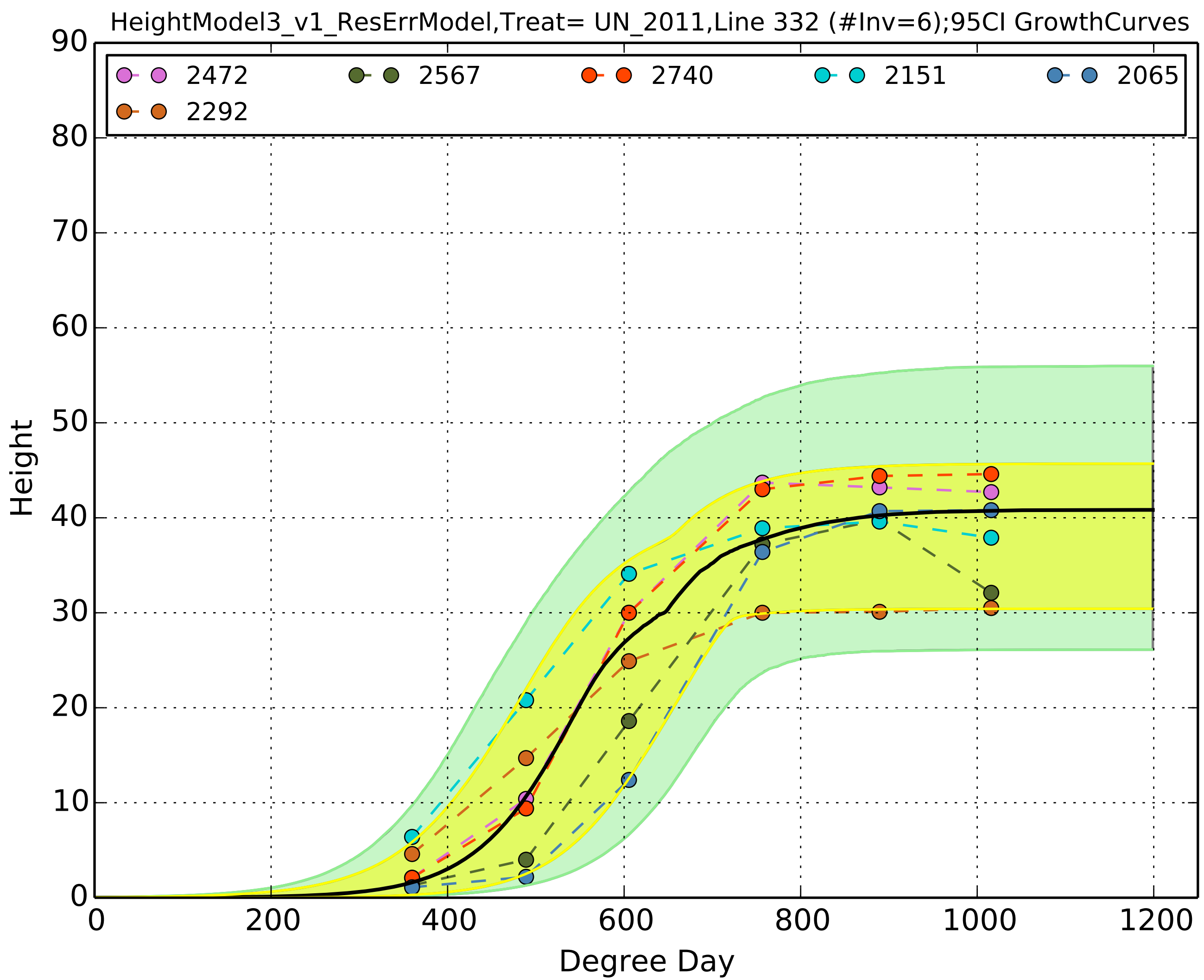

HeightModel3_v1_ResErrModel,Treat= UN_2011,Line 332 (#Inv=6);95CI GrowthCurves
2472
2567
2740
2151
2065
2292
Height
Degree Day
0
10
20
30
40
50
60
70
80
90
0
200
400
600
800
1000
1200

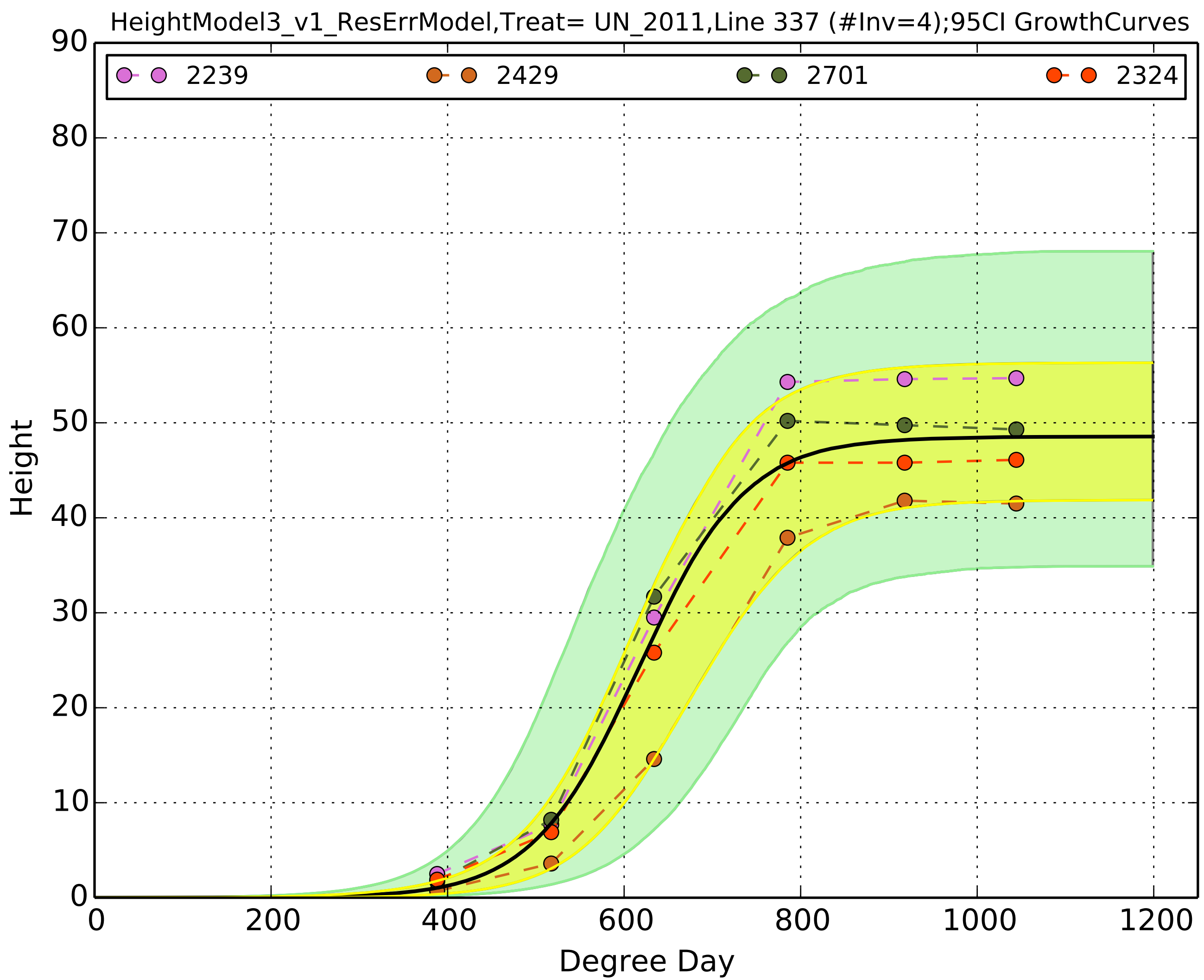

HeightModel3_v1_ResErrModel,Treat= UN_2011,Line 337 (#Inv=4);95CI GrowthCurves
2239
2429
2701
2324
Height
Degree Day
0
10
20
30
40
50
60
70
80
90
0
200
400
600
800
1000
1200

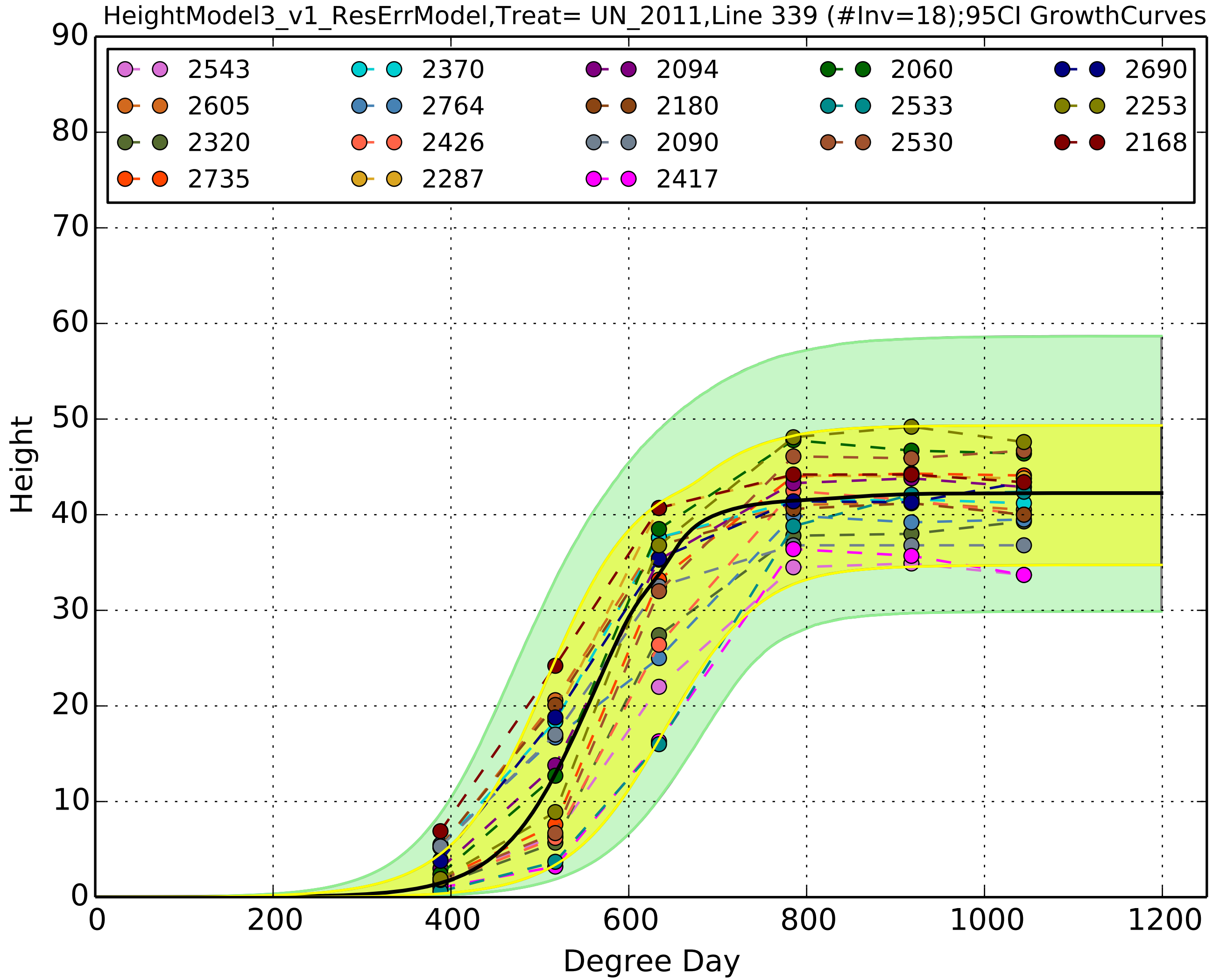

HeightModel3_v1_ResErrModel,Treat= UN_2011,Line 339 (#Inv=18);95CI GrowthCurves
2543
2605
2320
2735
2370
2764
2426
2287
2094
2180
2090
2417
2060
2533
2530
2690
2253
2168
Height
Degree Day
0
10
20
30
40
50
60
70
80
90
0
200
400
600
800
1000
1200

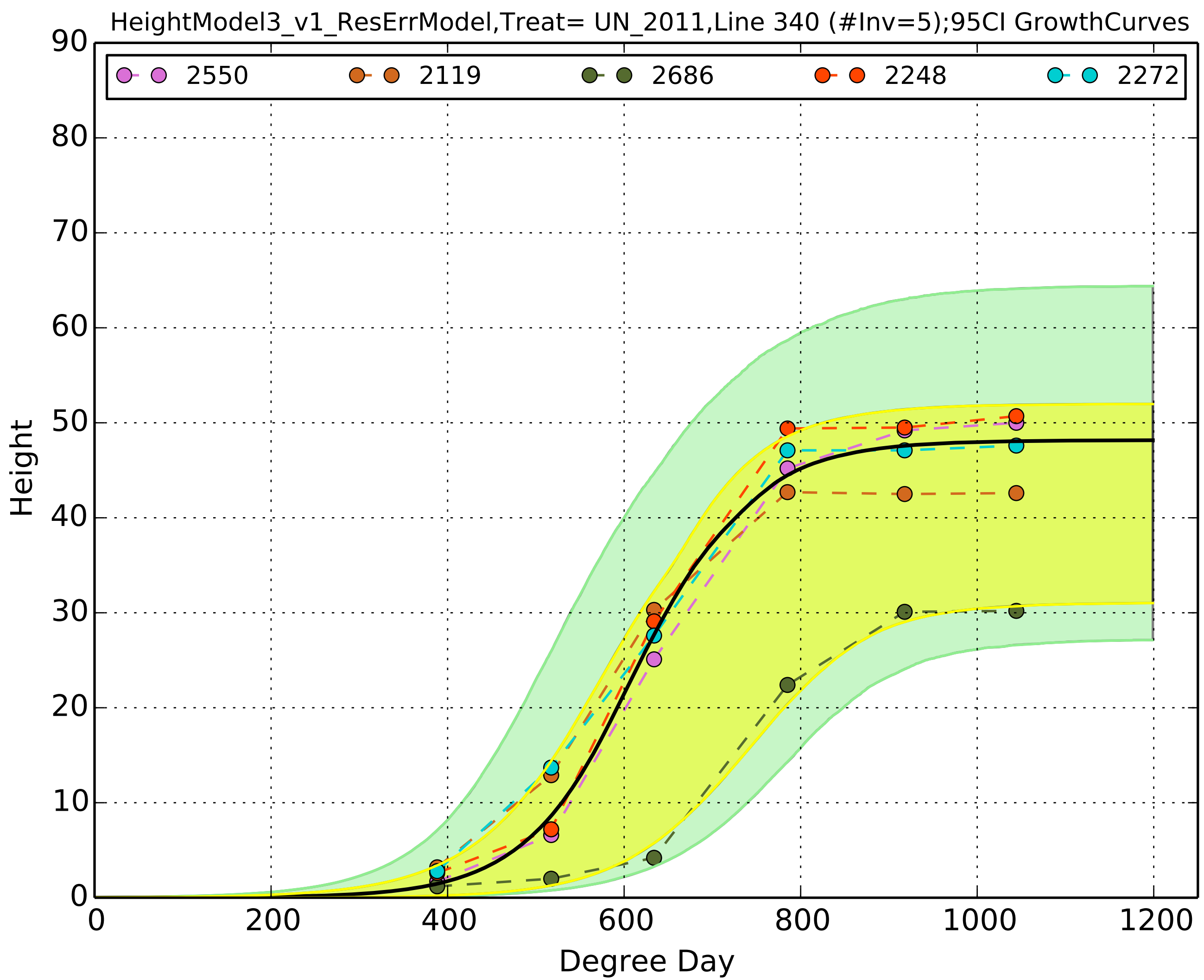

HeightModel3_v1_ResErrModel,Treat= UN_2011,Line 340 (#Inv=5);95CI GrowthCurves
2550
2119
2686
2248
2272
Height
Degree Day
0
10
20
30
40
50
60
70
80
90
0
200
400
600
800
1000
1200

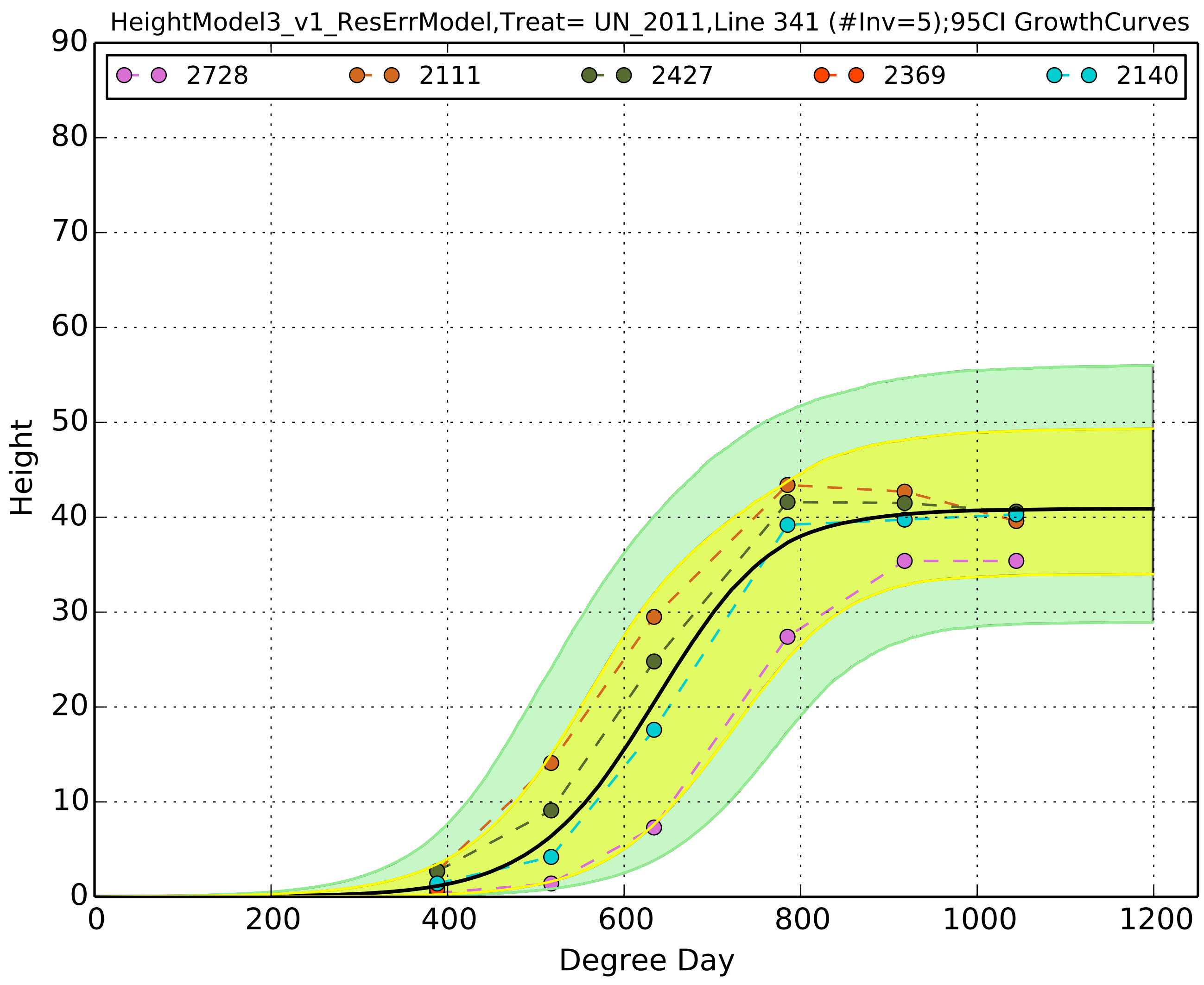
HeightModel3_v1_ResErrModel,Treat= UN_2011,Line 341 (#Inv=5);95CI GrowthCurves
2728
2111
2427
2369
2140
Height
Degree Day
0
10
20
30
40
50
60
70
80
90
0
200
400
600
800
1000
1200

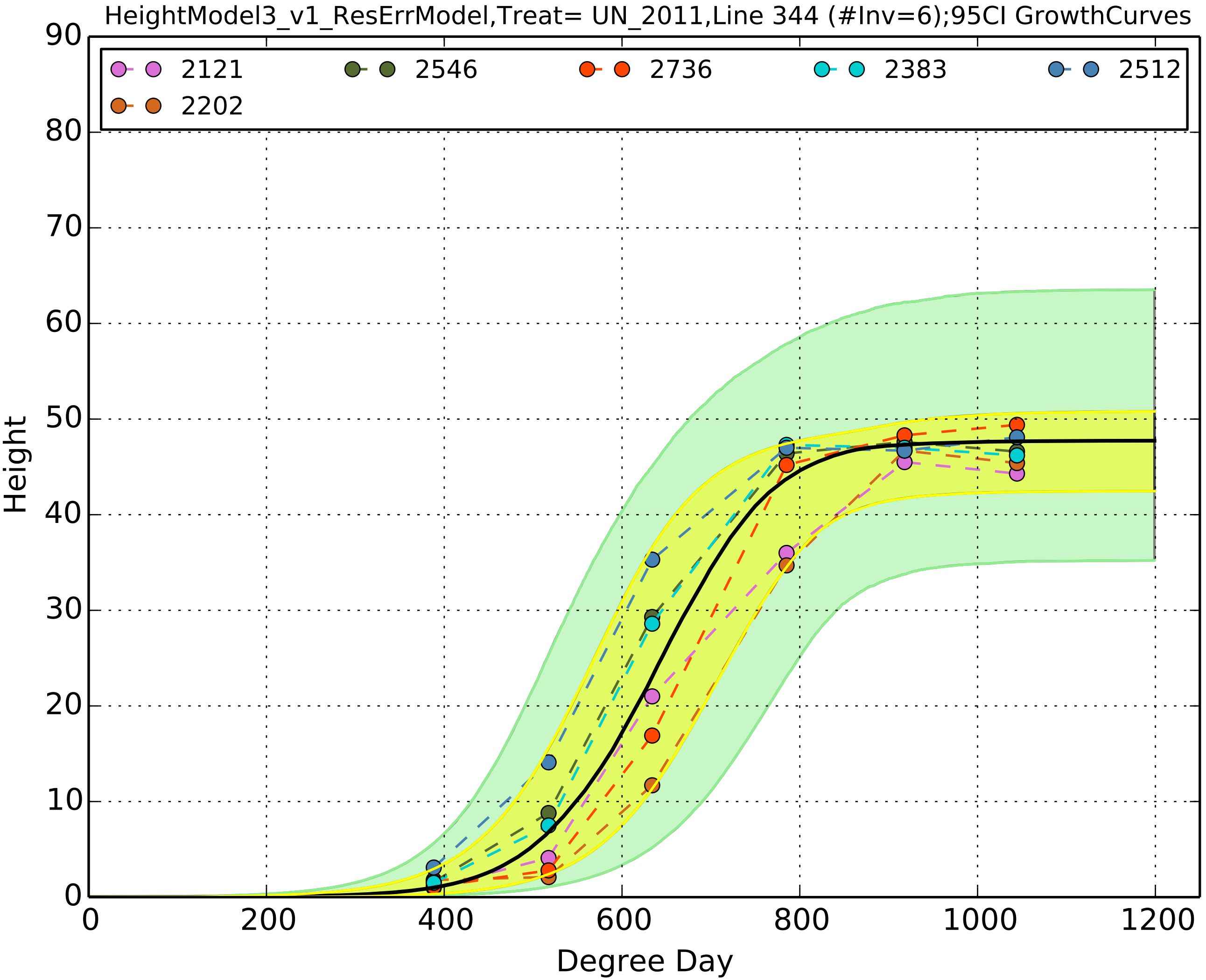
HeightModel3_v1_ResErrModel,Treat= UN_2011,Line 344 (#Inv=6);95CI GrowthCurves
2121
2546
2736
2383
2512
2202
Height
Degree Day
0
10
20
30
40
50
60
70
80
90
0
200
400
600
800
1000
1200

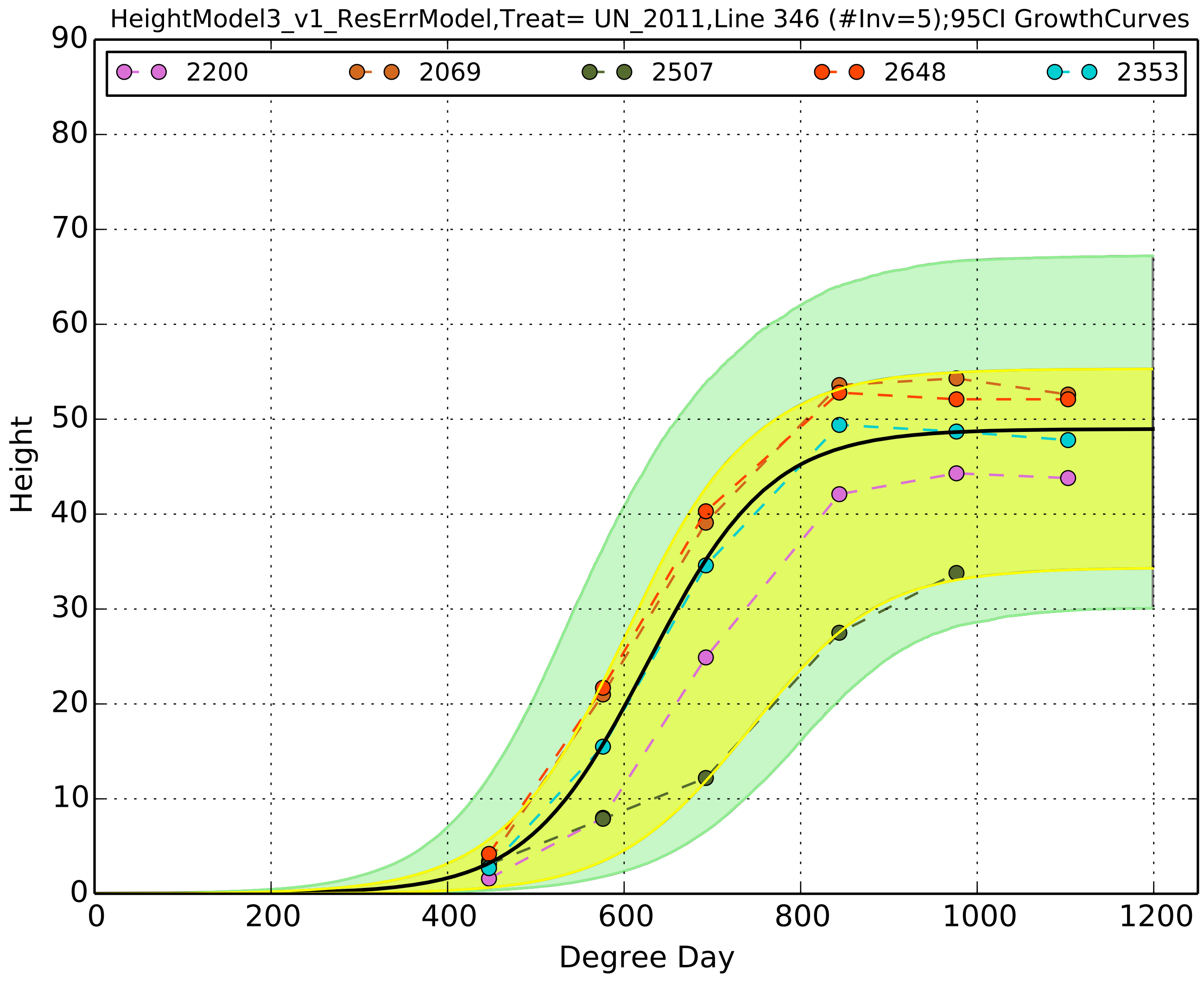

HeightModel3_v1_ResErrModel,Treat= UN_2011,Line 346 (#Inv=5);95CI GrowthCurves
2200
2069
2507
2648
2353
Height
Degree Day
0
10
20
30
40
50
60
70
80
90
0
200
400
600
800
1000
1200

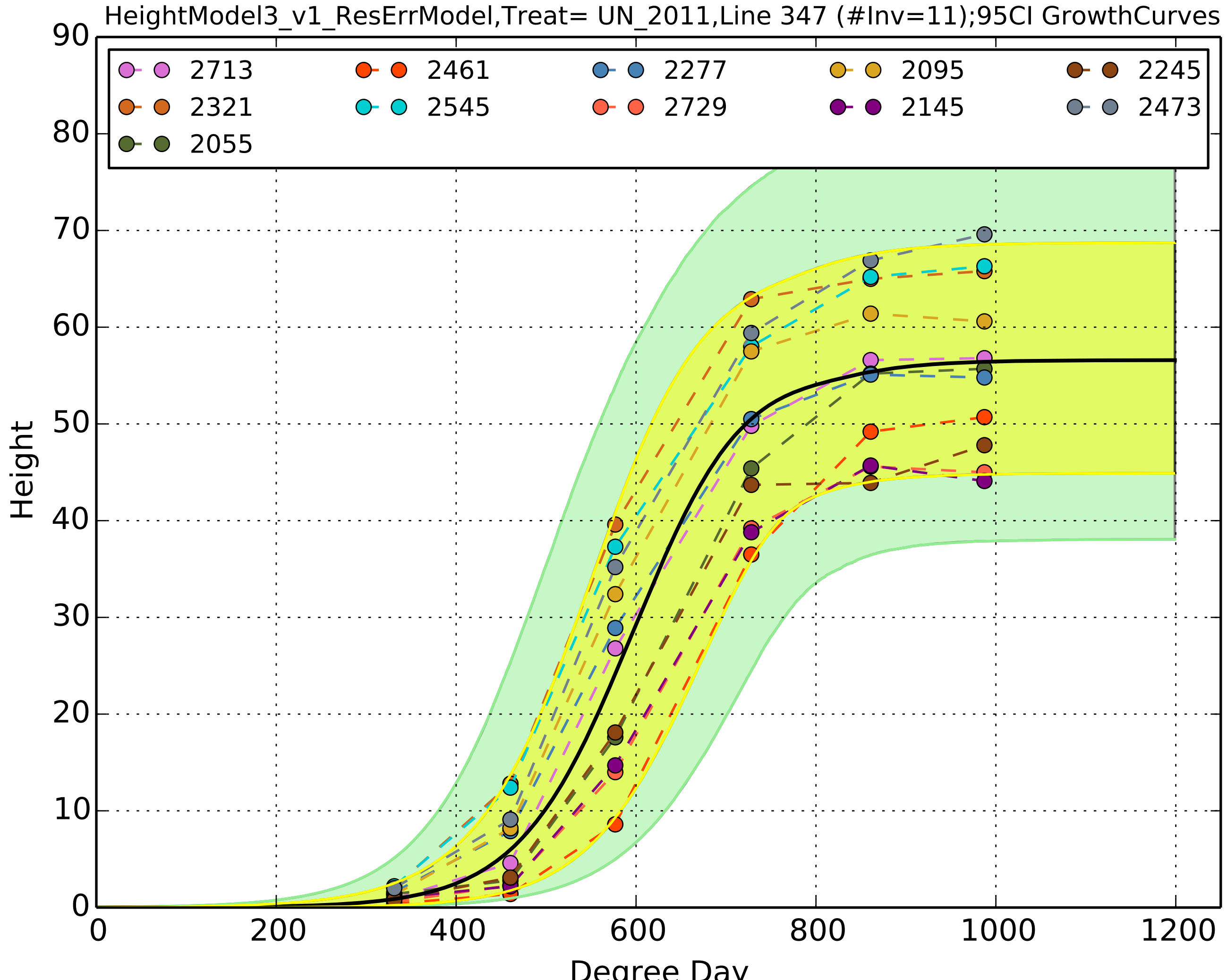
HeightModel3_v1_ResErrModel,Treat= UN_2011,Line 347 (#Inv=11);95CI GrowthCurves
2713
2461
2277
2095
2245
2321
2545
2729
2145
2473
2055
Height
Degree Day
0
10
20
30
40
50
60
70
80
90
0
200
400
600
800
1000
1200

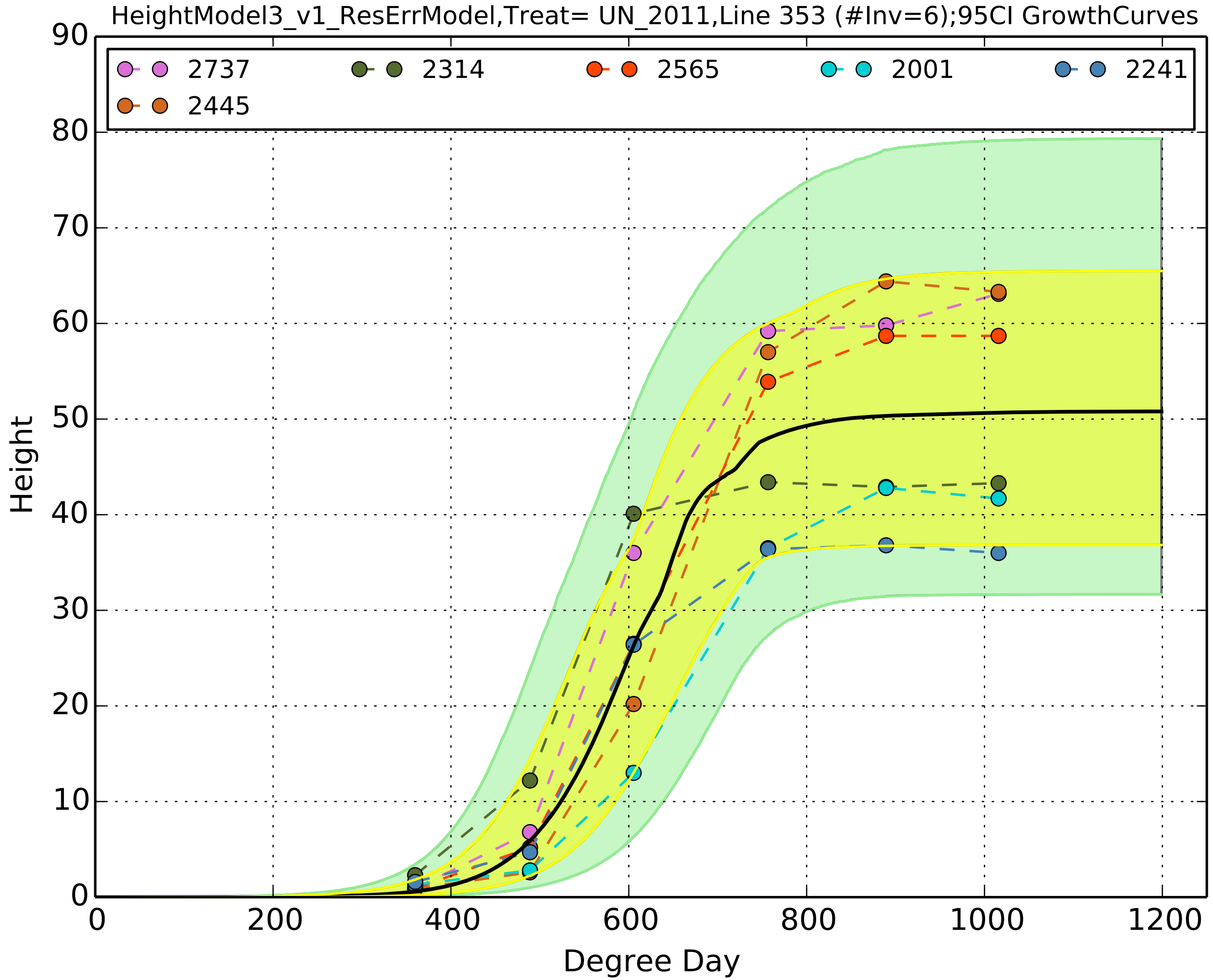
HeightModel3_v1_ResErrModel,Treat= UN_2011,Line 353 (#Inv=6);95CI GrowthCurves
2737
2314
2565
2001
2241
2445
Height
Degree Day
0
10
20
30
40
50
60
70
80
90
0
200
400
600
800
1000
1200

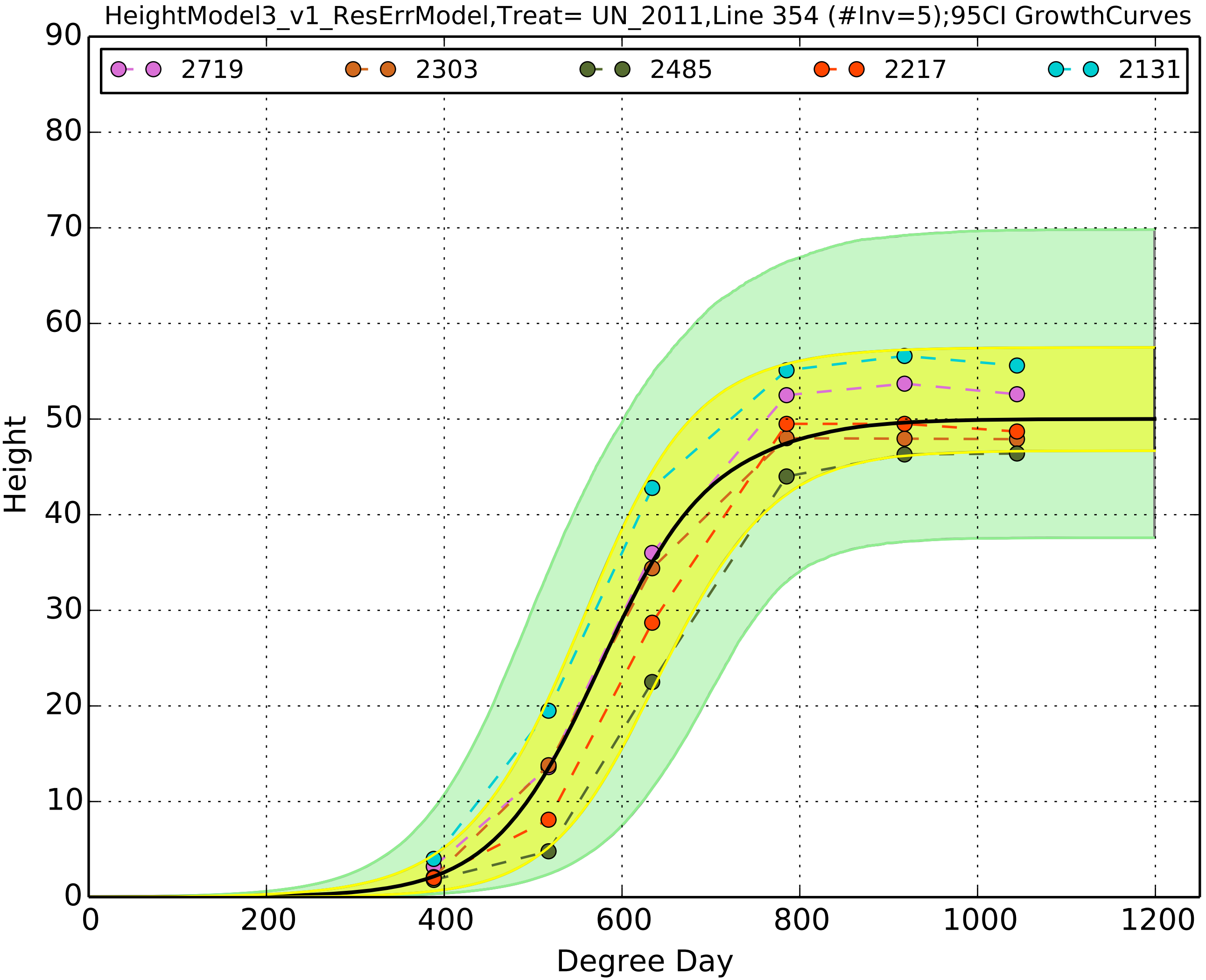

HeightModel3_v1_ResErrModel,Treat= UN_2011,Line 354 (#Inv=5);95CI GrowthCurves
2719
2303
2485
2217
2131
Height
Degree Day
0
10
20
30
40
50
60
70
80
90
0
200
400
600
800
1000
1200

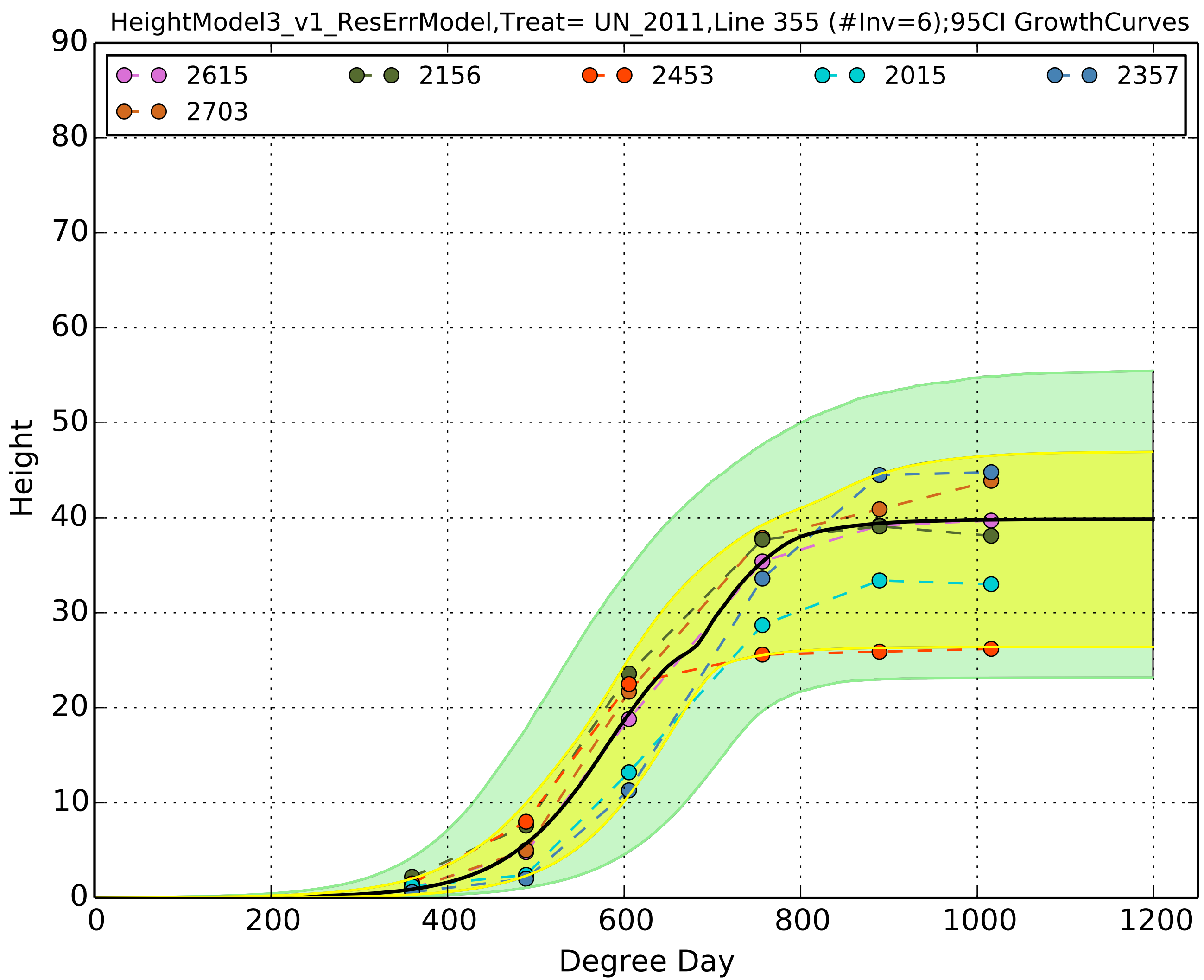

HeightModel3_v1_ResErrModel,Treat= UN_2011,Line 355 (#Inv=6);95CI GrowthCurves
2615
2156
2453
2015
2357
2703
Height
Degree Day
0
10
20
30
40
50
60
70
80
90
0
200
400
600
800
1000
1200

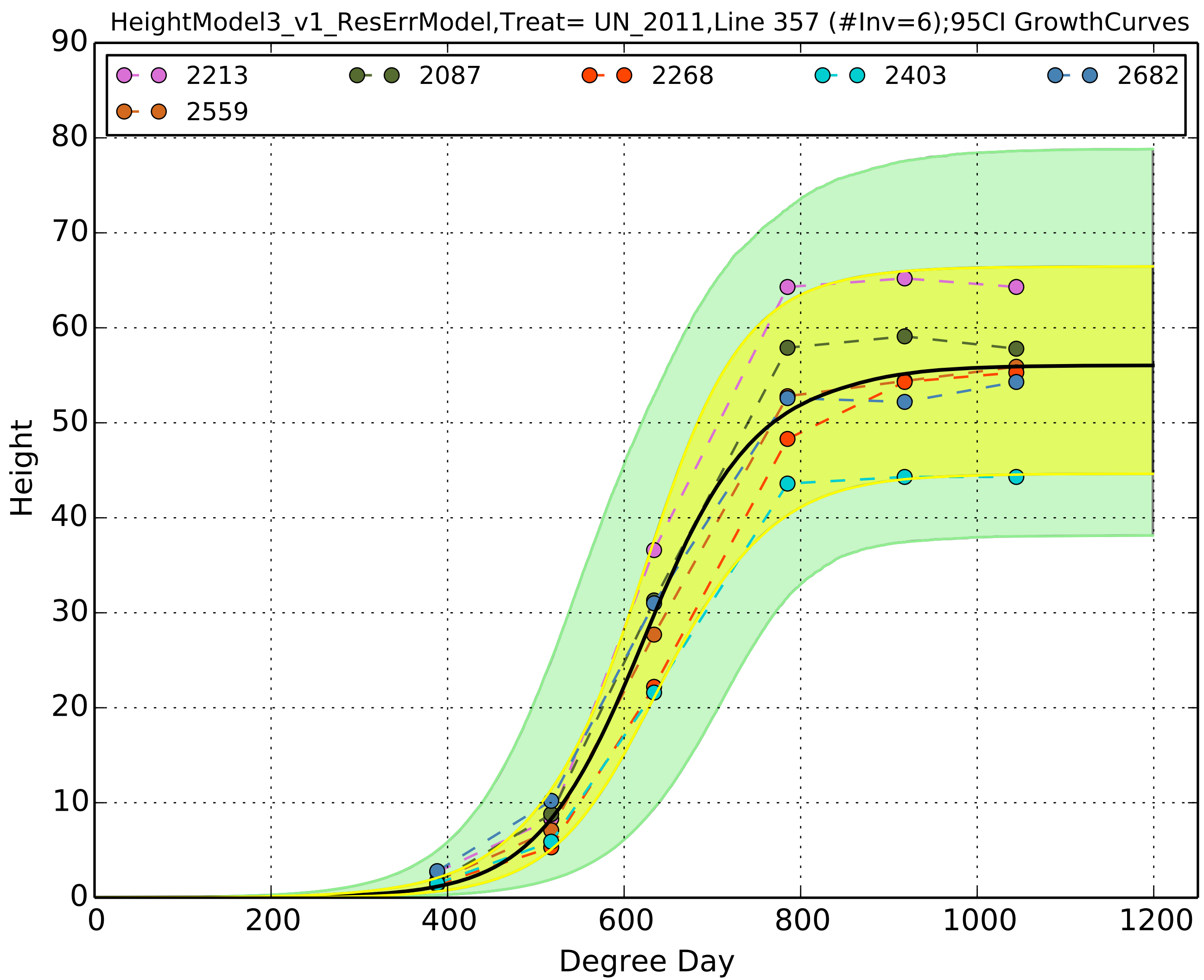
HeightModel3_v1_ResErrModel,Treat= UN_2011,Line 357 (#Inv=6);95CI GrowthCurves
2213
2087
2268
2403
2682
2559
Height
Degree Day
0
10
20
30
40
50
60
70
80
90
0
200
400
600
800
1000
1200

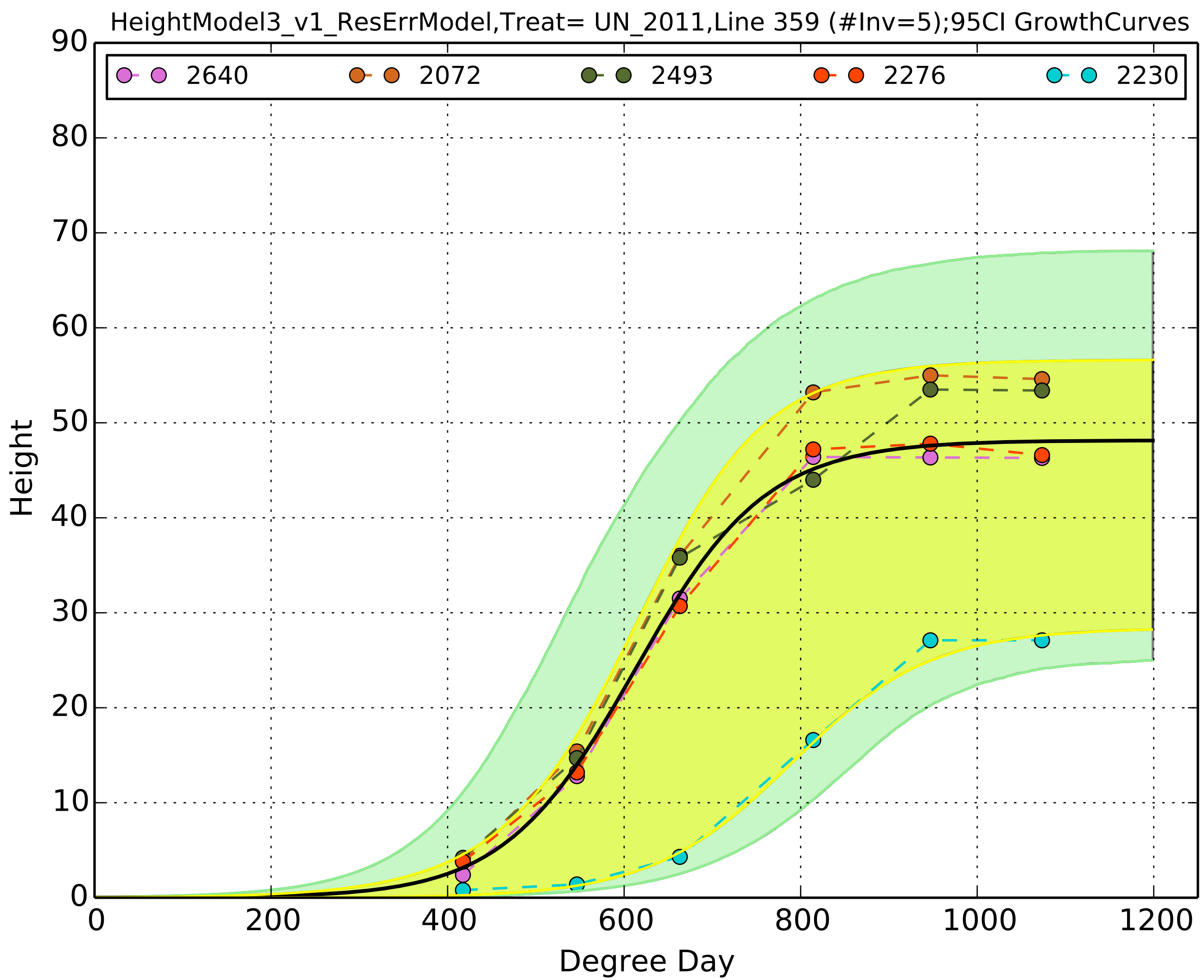

HeightModel3_v1_ResErrModel,Treat= UN_2011,Line 359 (#Inv=5);95CI GrowthCurves
2640
2072
2493
2276
2230
Height
Degree Day
0
10
20
30
40
50
60
70
80
90
0
200
400
600
800
1000
1200

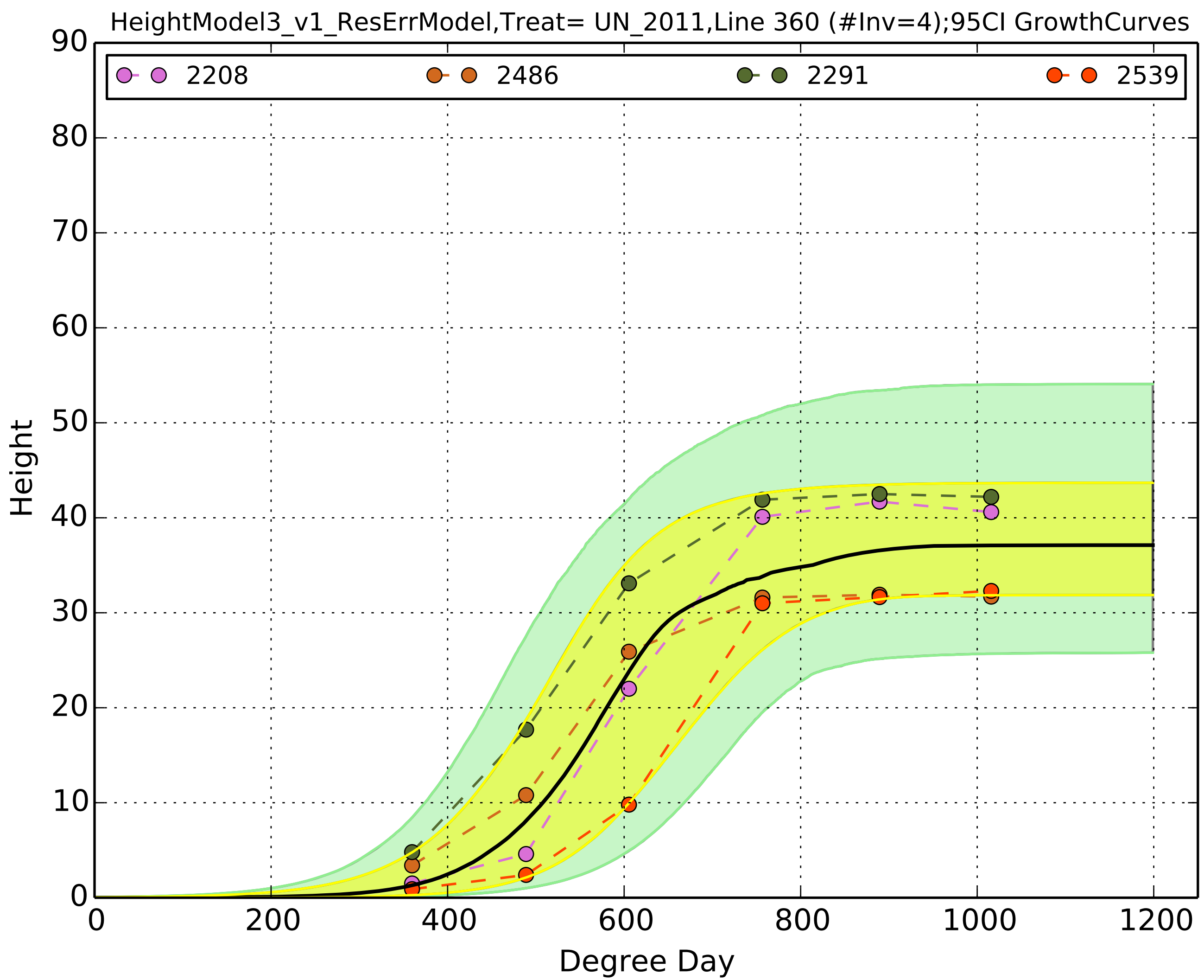
HeightModel3_v1_ResErrModel,Treat= UN_2011,Line 360 (#Inv=4);95CI GrowthCurves
2208
2486
2291
2539
Height
Degree Day
0
10
20
30
40
50
60
70
80
90
0
200
400
600
800
1000
1200

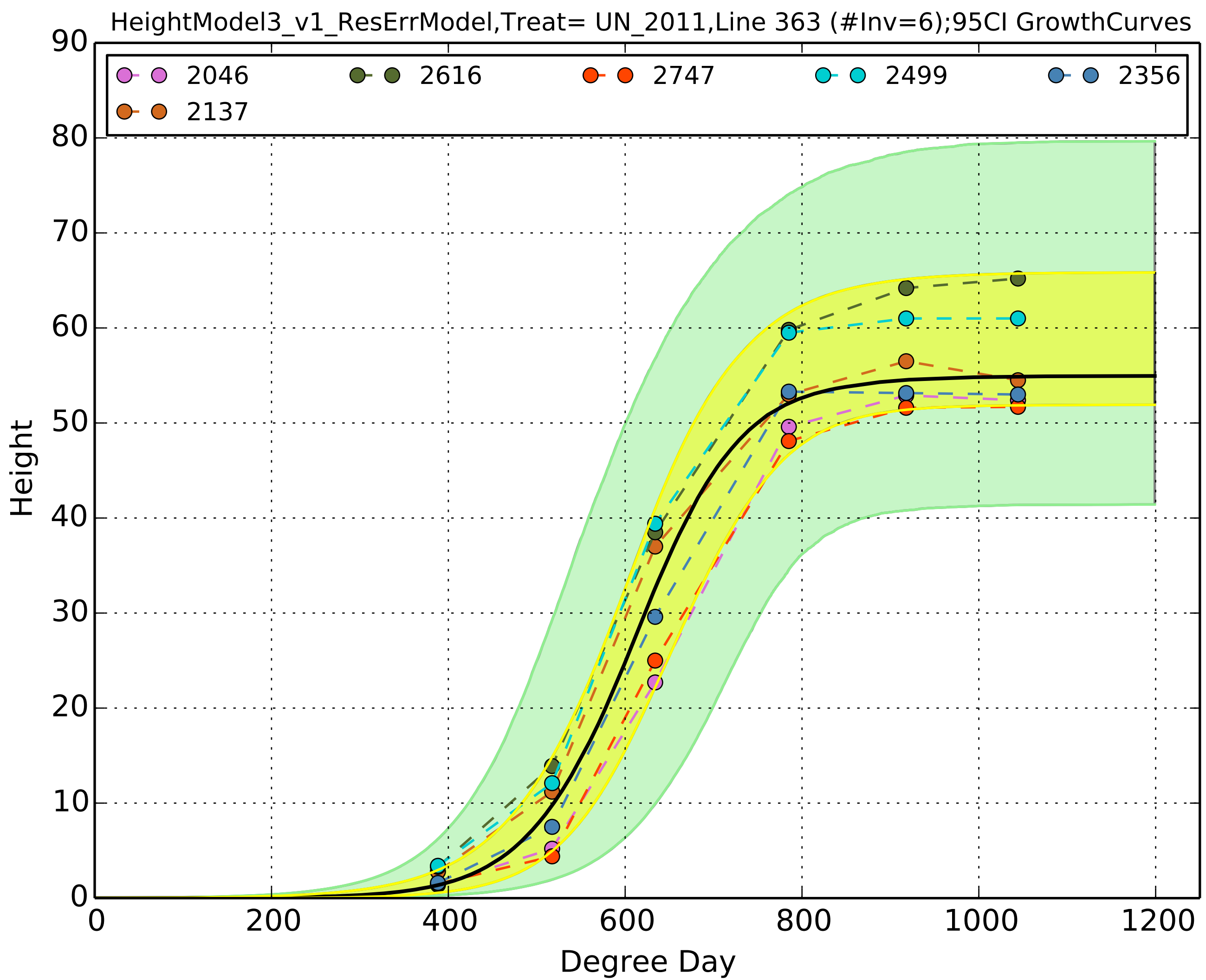

HeightModel3_v1_ResErrModel,Treat= UN_2011,Line 363 (#Inv=6);95CI GrowthCurves
2046
2616
2747
2499
2356
2137
Height
Degree Day
0
10
20
30
40
50
60
70
80
90
0
200
400
600
800
1000
1200

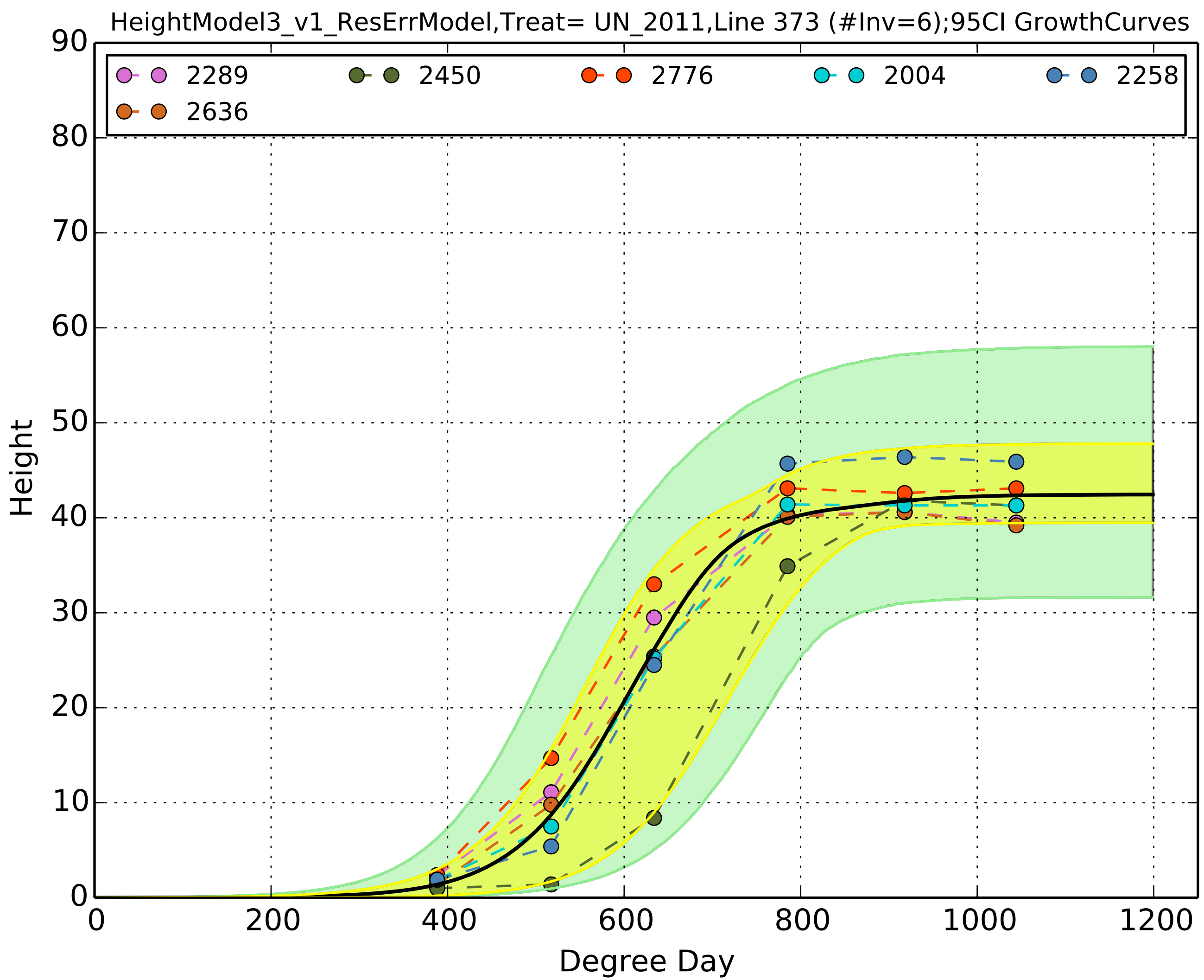

HeightModel3_v1_ResErrModel,Treat= UN_2011,Line 373 (#Inv=6);95CI GrowthCurves
2289
2450
2776
2004
2258
2636
Height
Degree Day
0
10
20
30
40
50
60
70
80
90
0
200
400
600
800
1000
1200

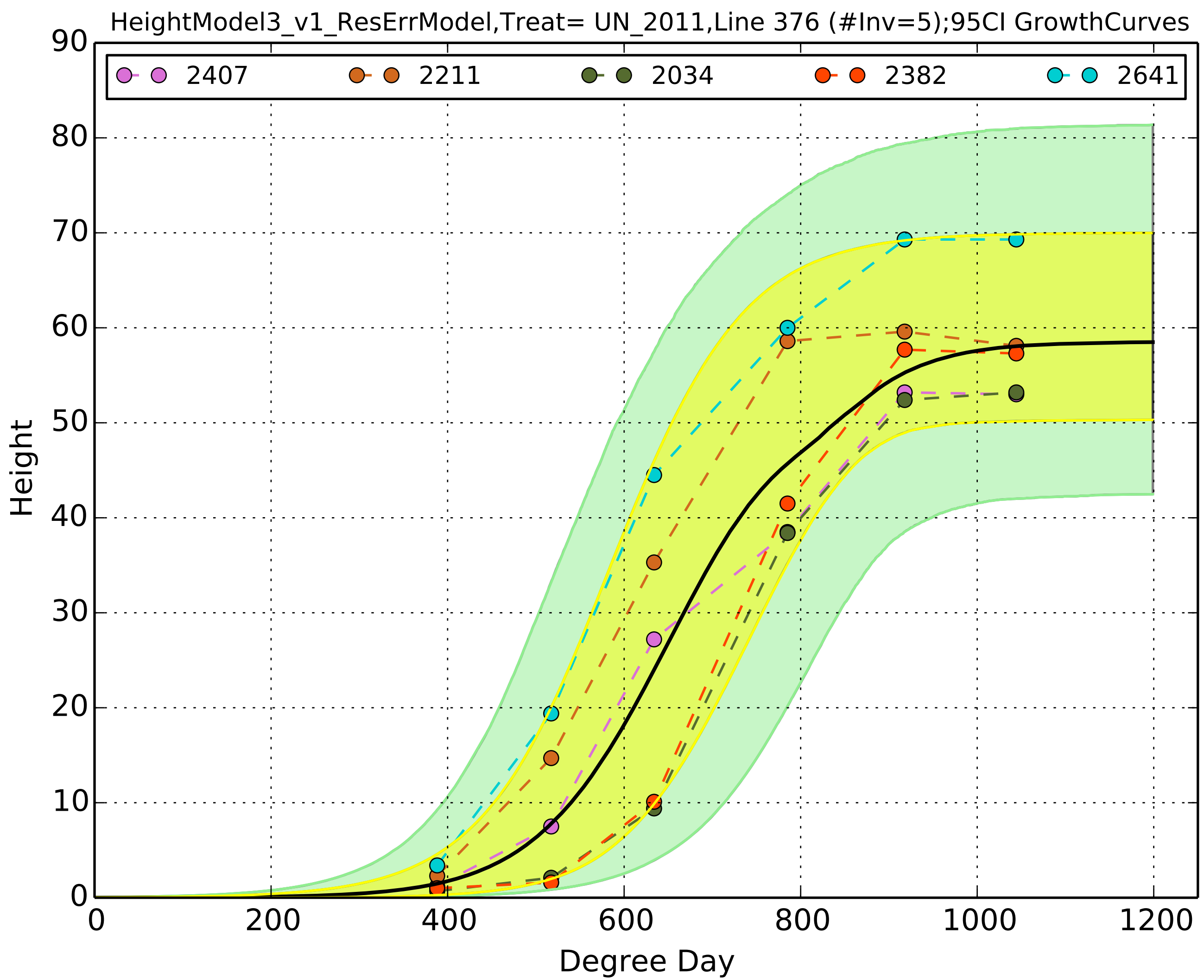

HeightModel3_v1_ResErrModel,Treat= UN_2011,Line 376 (#Inv=5);95CI GrowthCurves
2407
2211
2034
2382
2641
Height
Degree Day
0
10
20
30
40
50
60
70
80
90
0
200
400
600
800
1000
1200

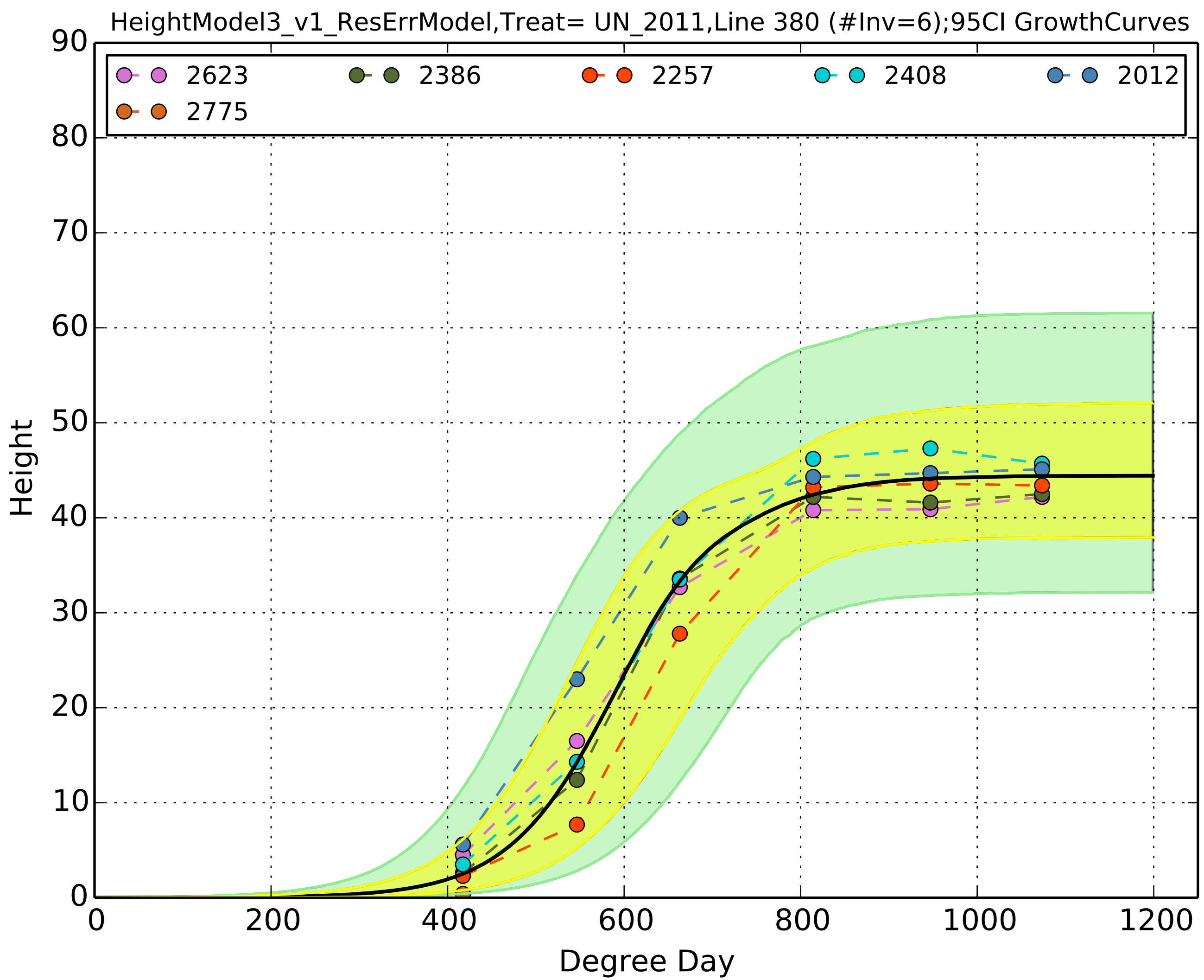
HeightModel3_v1_ResErrModel,Treat= UN_2011,Line 380 (#Inv=6);95CI GrowthCurves
2623
2386
2257
2408
2012
2775
Height
Degree Day
0
10
20
30
40
50
60
70
80
90
0
200
400
600
800
1000
1200

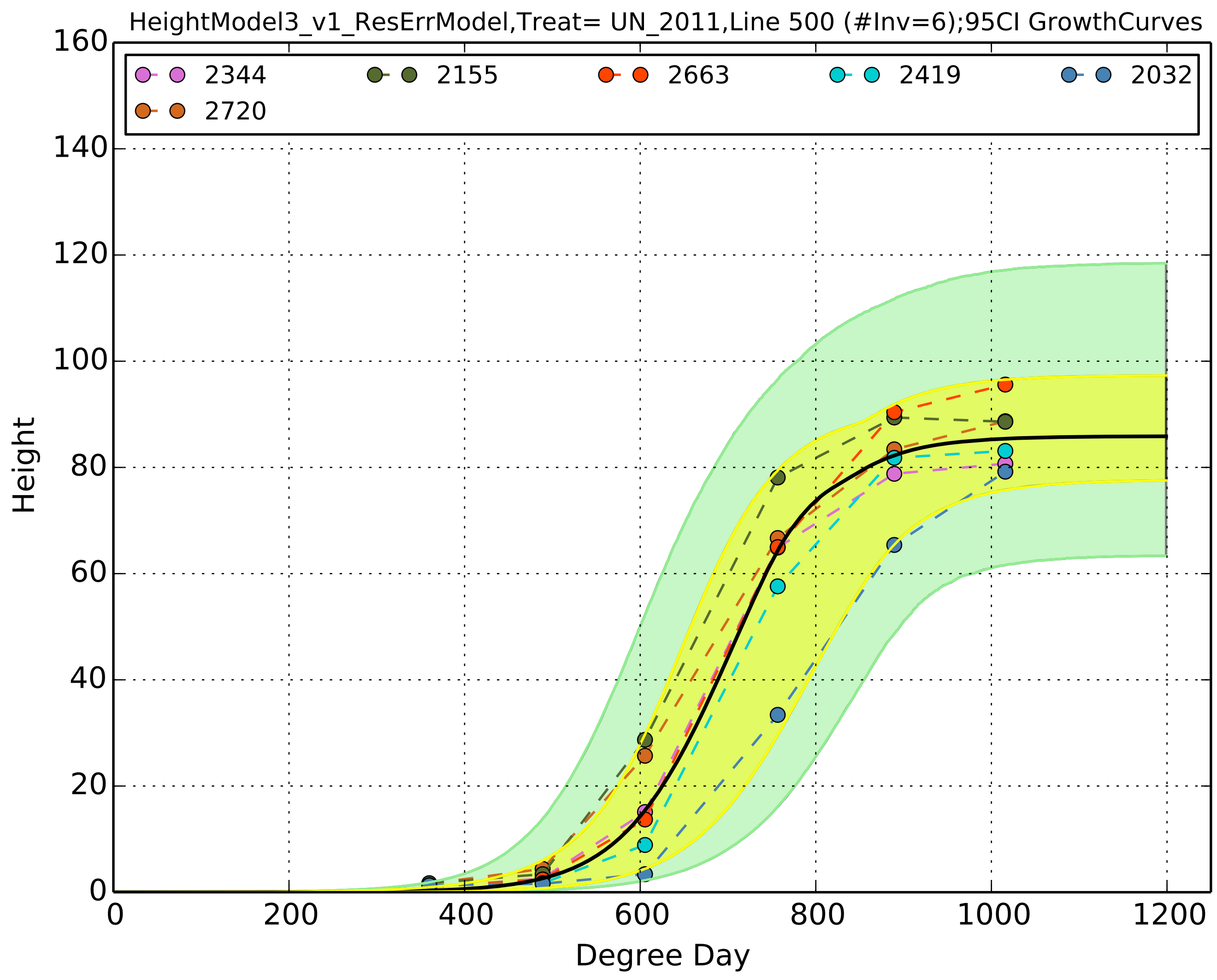
HeightModel3_v1_ResErrModel,Treat= UN_2011,Line 500 (#Inv=6);95CI GrowthCurves
2344
2155
2663
2419
2032
2720
Height
Degree Day
0
20
40
60
80
100
120
140
160
0
200
400
600
800
1000
1200

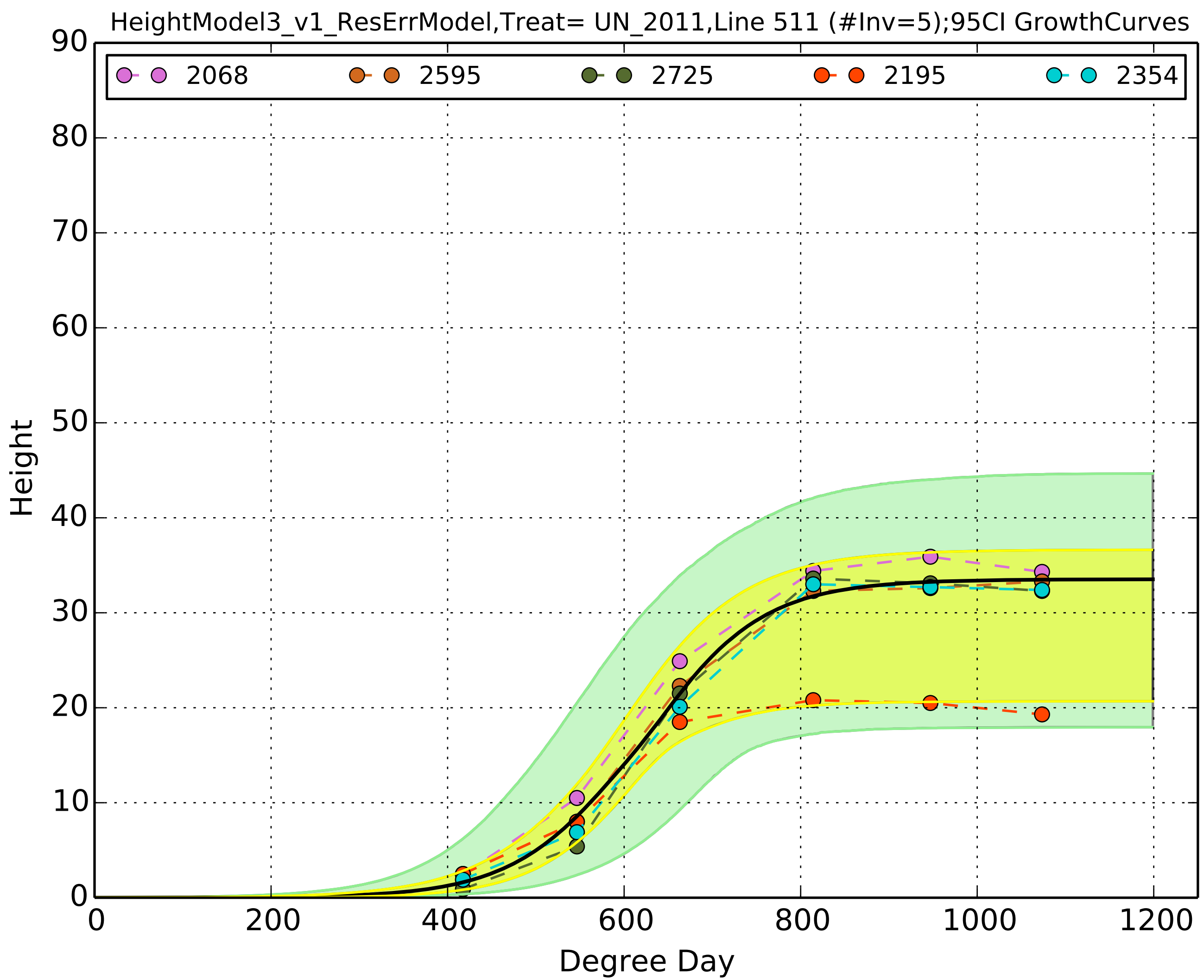
HeightModel3_v1_ResErrModel,Treat= UN_2011,Line 511 (#Inv=5);95CI GrowthCurves
2068
2595
2725
2195
2354
Height
Degree Day
0
10
20
30
40
50
60
70
80
90
0
200
400
600
800
1000
1200

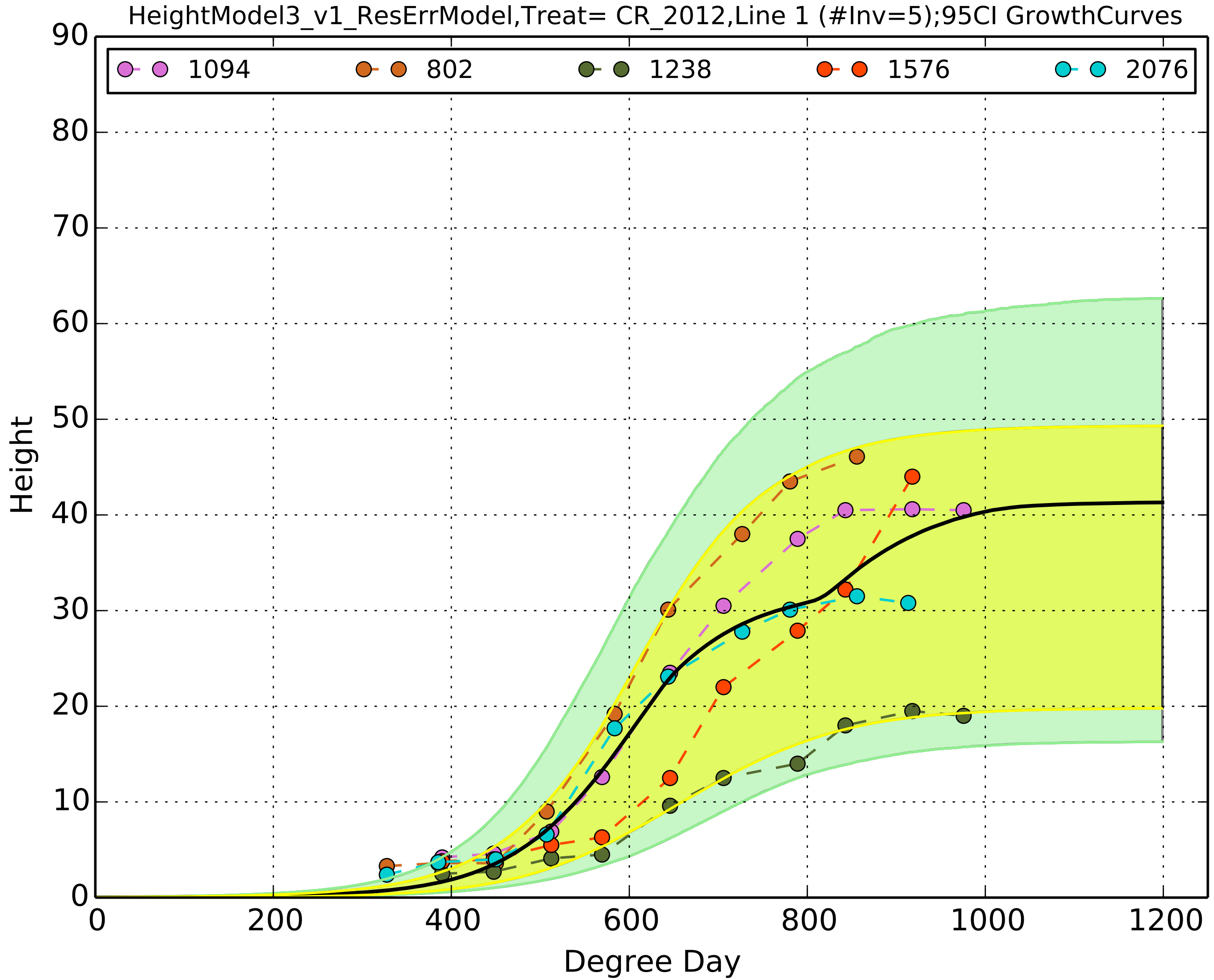

HeightModel3_v1_ResErrModel,Treat= CR_2012,Line 1 (#Inv=5);95CI GrowthCurves
1094
802
1238
1576
2076
Height
Degree Day
0
10
20
30
40
50
60
70
80
90
0
200
400
600
800
1000
1200

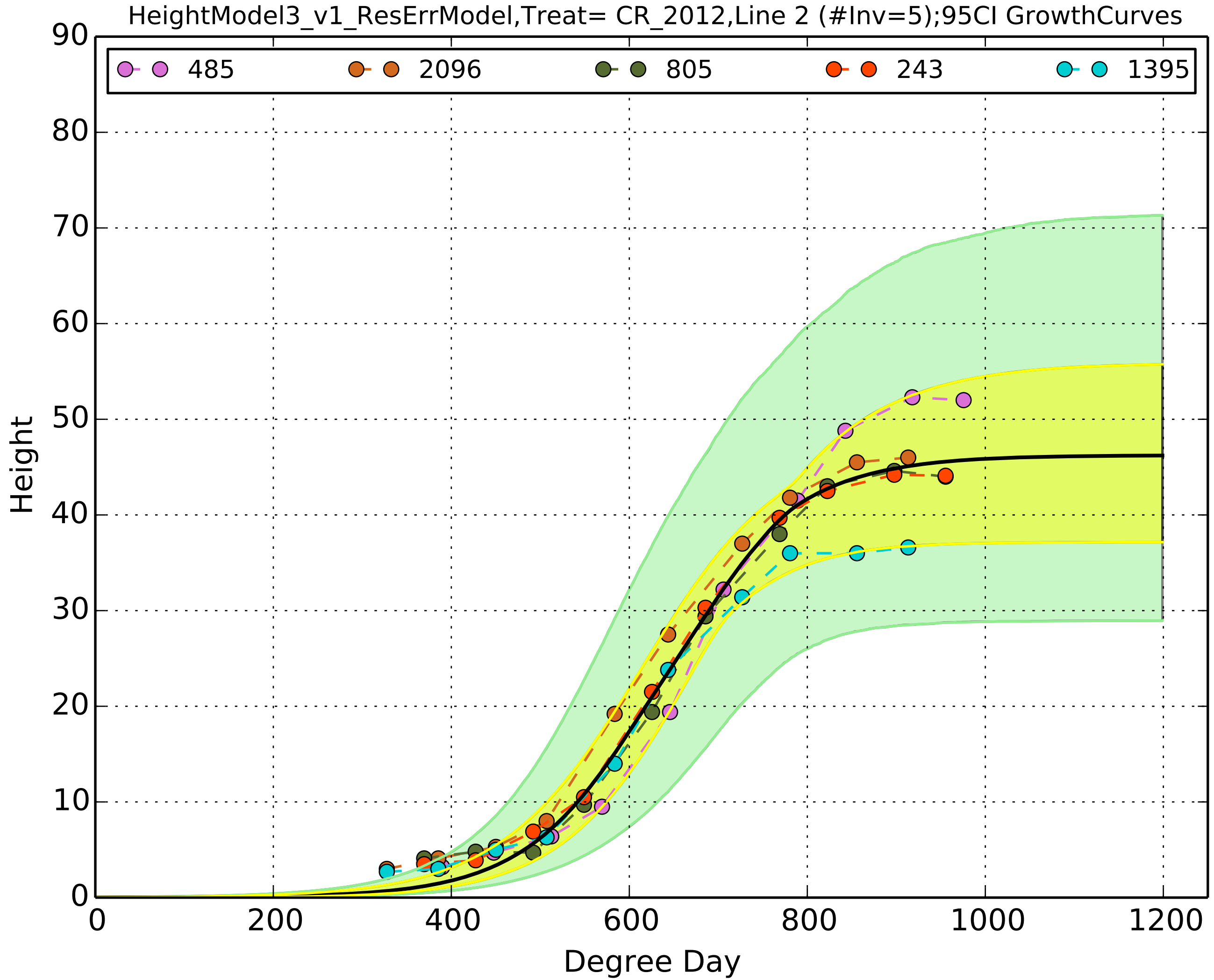

HeightModel3_v1_ResErrModel,Treat= CR_2012,Line 2 (#Inv=5);95CI GrowthCurves
485
2096
805
243
1395
Height
Degree Day
0
10
20
30
40
50
60
70
80
90
0
200
400
600
800
1000
1200

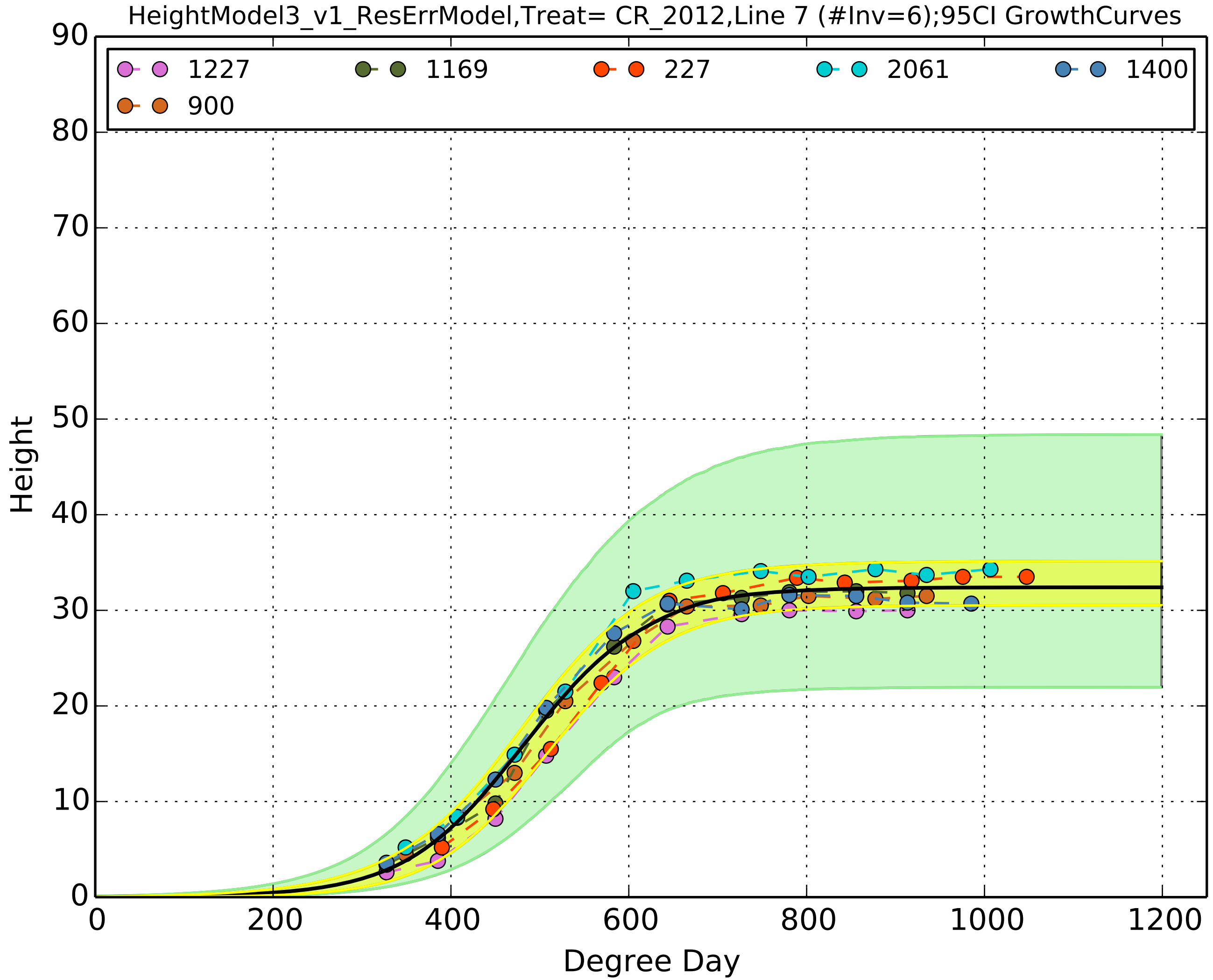

HeightModel3_v1_ResErrModel,Treat= CR_2012,Line 7 (#Inv=6);95CI GrowthCurves
1227
1169
227
2061
1400
900
Height
Degree Day
0
10
20
30
40
50
60
70
80
90
0
200
400
600
800
1000
1200

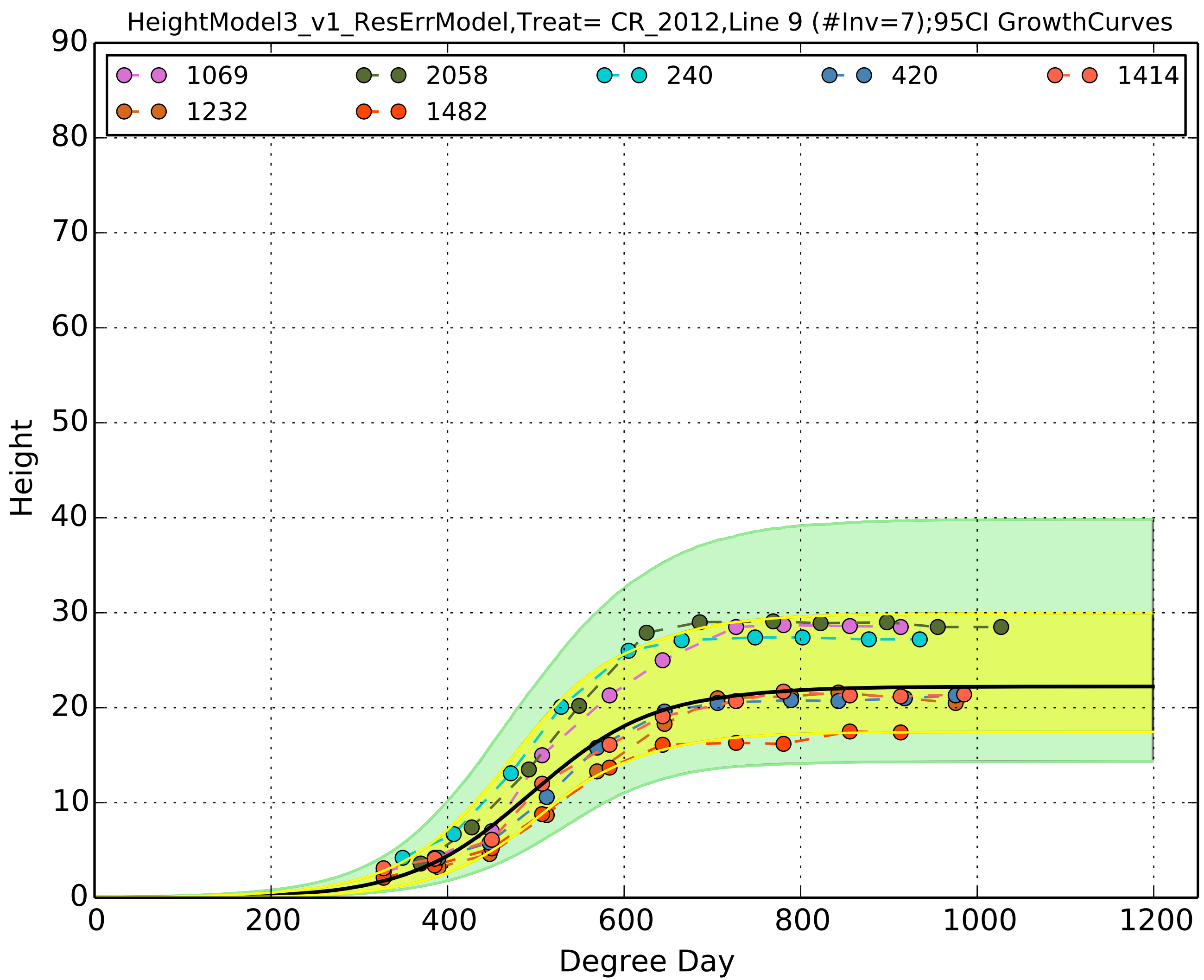

HeightModel3_v1_ResErrModel,Treat= CR_2012,Line 9 (#Inv=7);95CI GrowthCurves
1069
2058
240
420
1414
1232
1482
Height
Degree Day
0
10
20
30
40
50
60
70
80
90
0
200
400
600
800
1000
1200

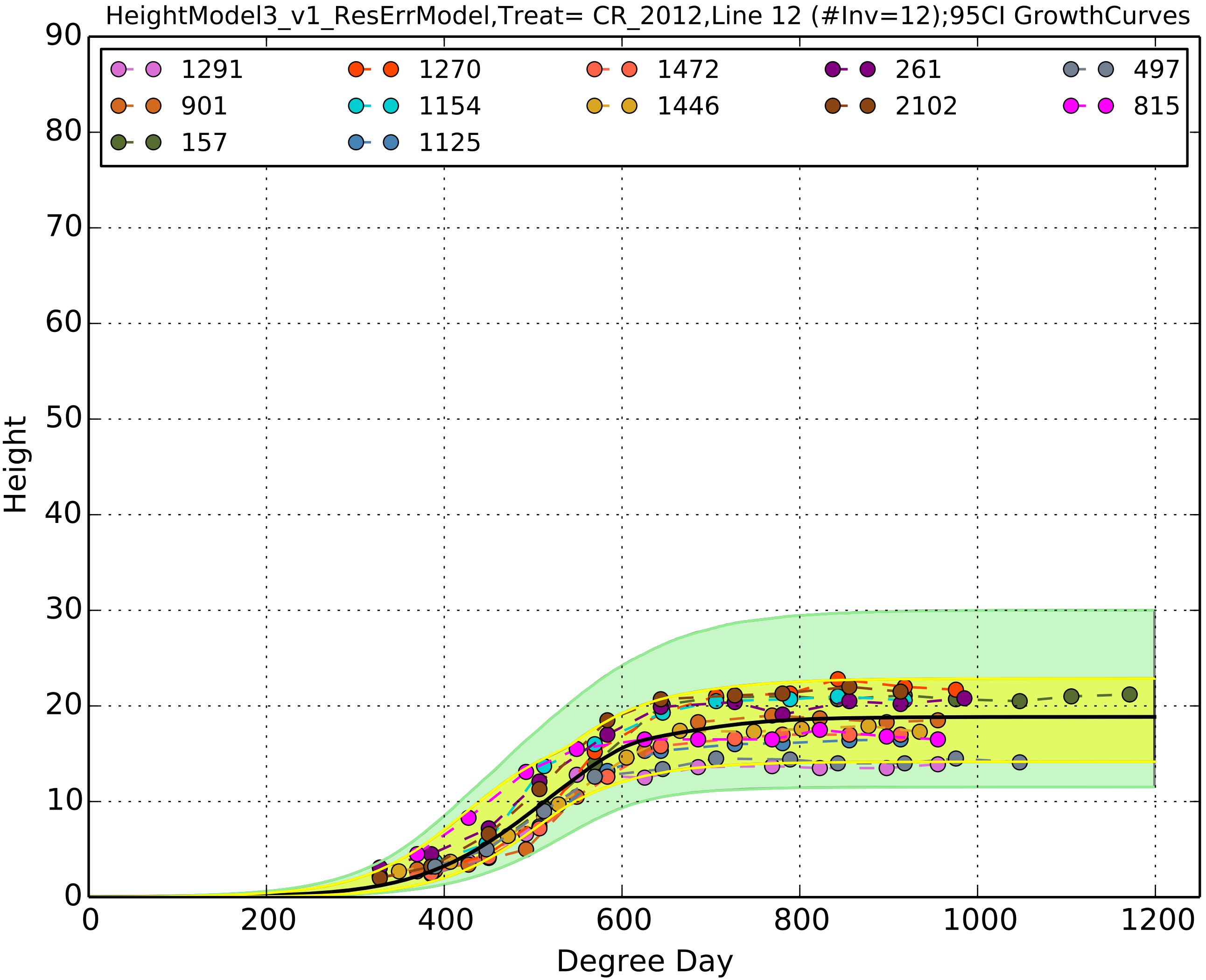

HeightModel3_v1_ResErrModel,Treat= CR_2012,Line 12 (#Inv=12);95CI GrowthCurves
1291
1270
1472
261
497
901
1154
1446
2102
815
157
1125
Height
Degree Day
0
10
20
30
40
50
60
70
80
90
0
200
400
600
800
1000
1200

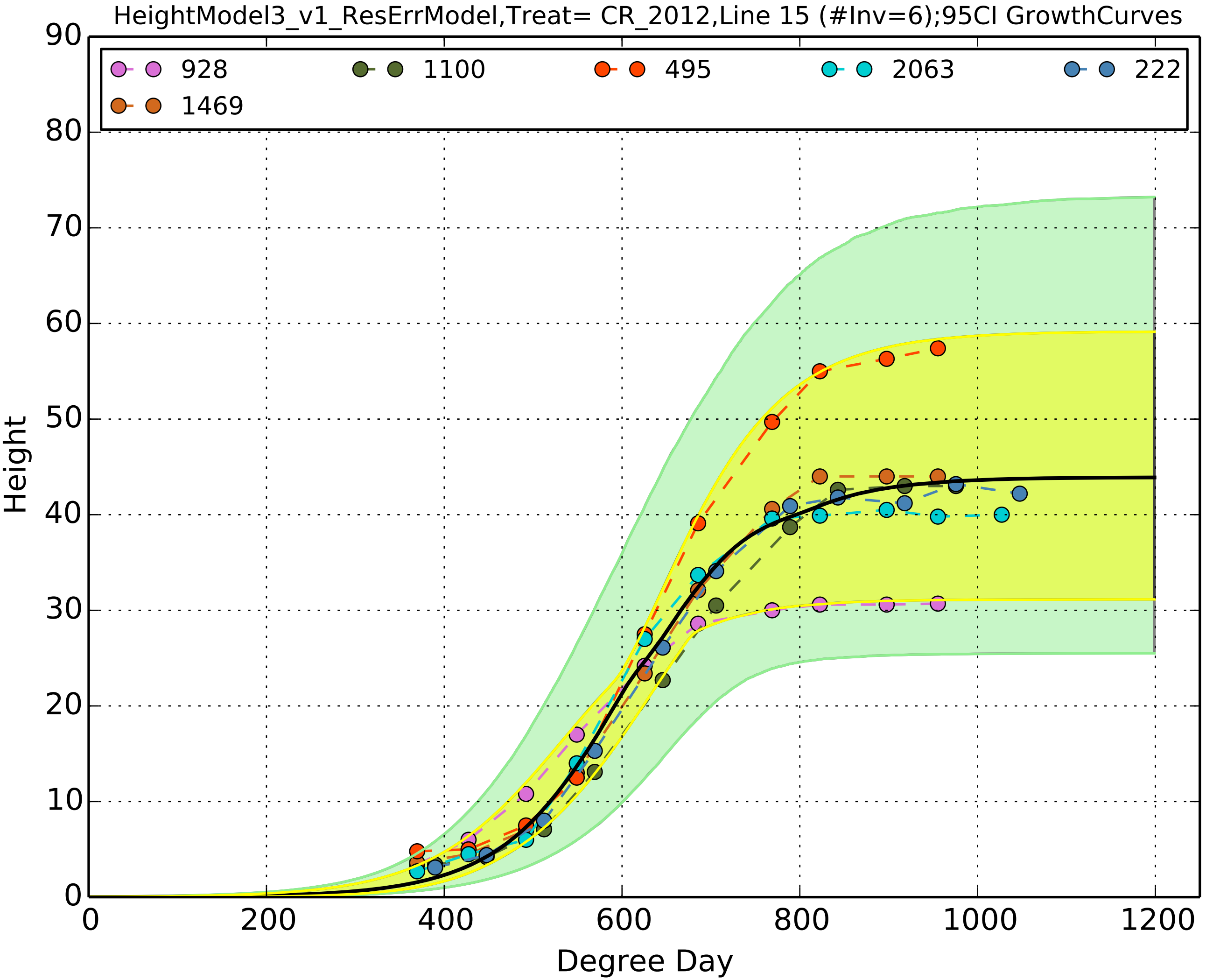

HeightModel3_v1_ResErrModel,Treat= CR_2012,Line 15 (#Inv=6);95CI GrowthCurves
928
1100
495
2063
222
1469
Height
Degree Day
0
10
20
30
40
50
60
70
80
90
0
200
400
600
800
1000
1200

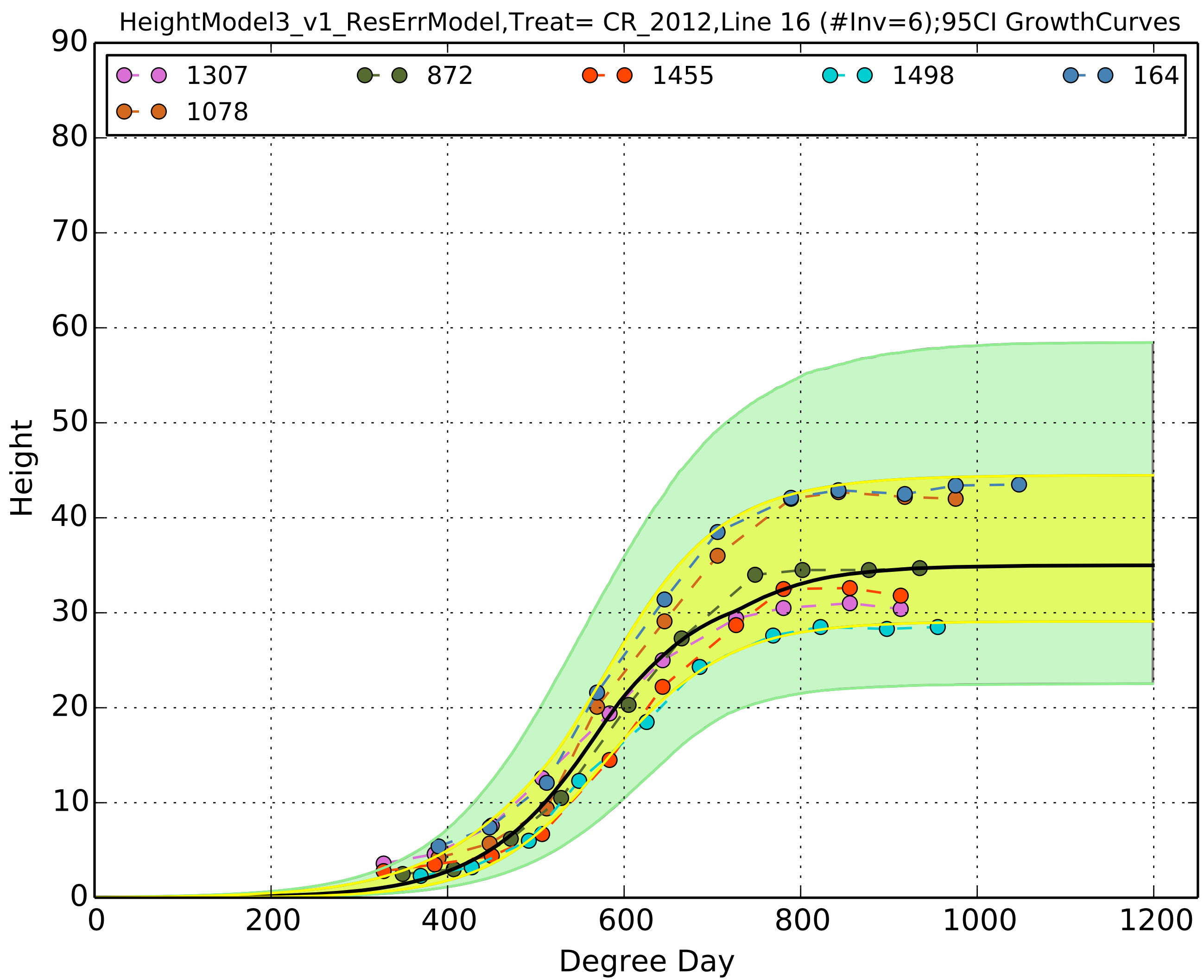

HeightModel3_v1_ResErrModel,Treat= CR_2012,Line 16 (#Inv=6);95CI GrowthCurves
1307
872
1455
1498
164
1078
Height
Degree Day
0
10
20
30
40
50
60
70
80
90
0
200
400
600
800
1000
1200

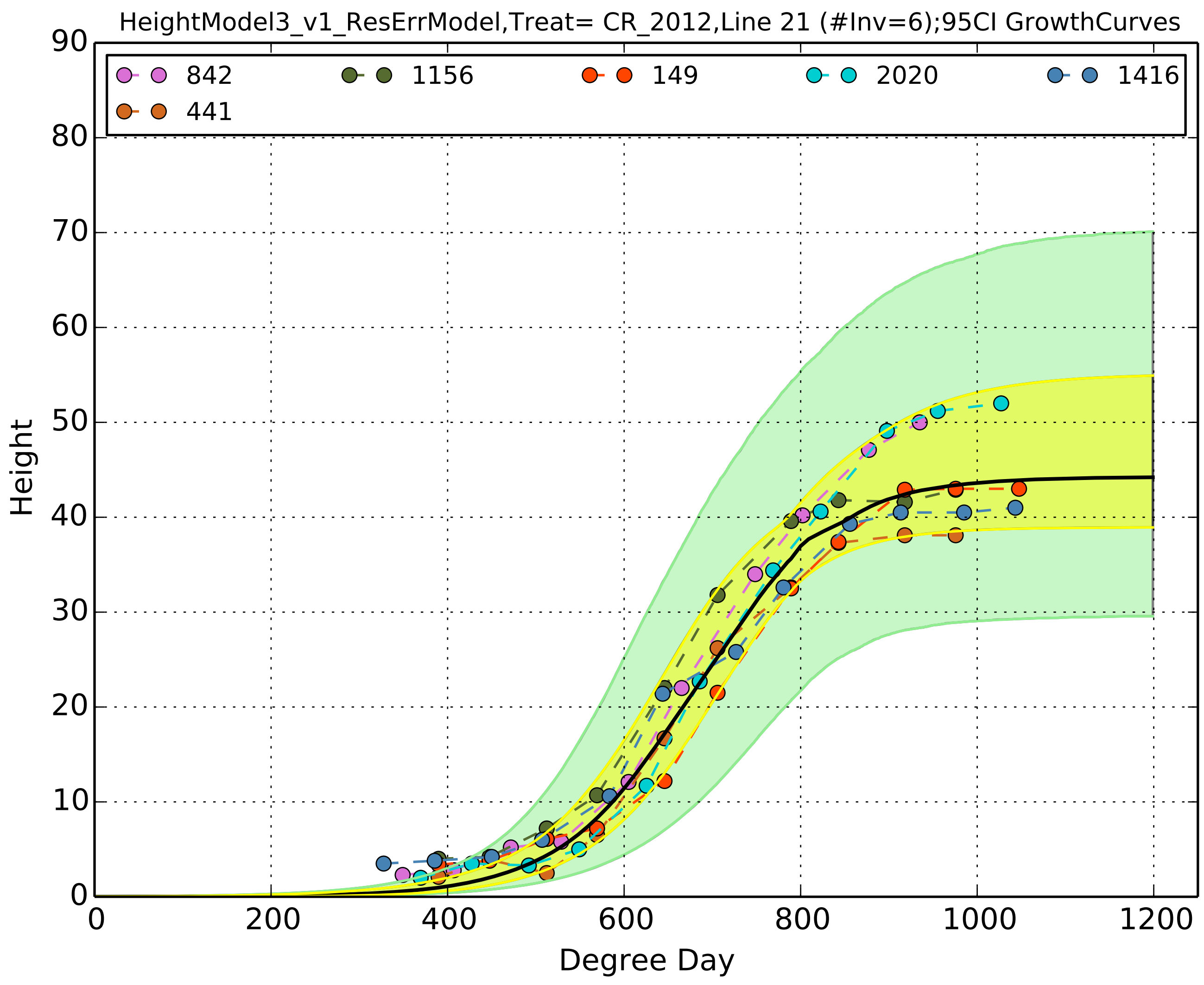
HeightModel3_v1_ResErrModel,Treat= CR_2012,Line 21 (#Inv=6);95CI GrowthCurves
842
1156
149
2020
1416
441
Height
Degree Day
0
10
20
30
40
50
60
70
80
90
0
200
400
600
800
1000
1200

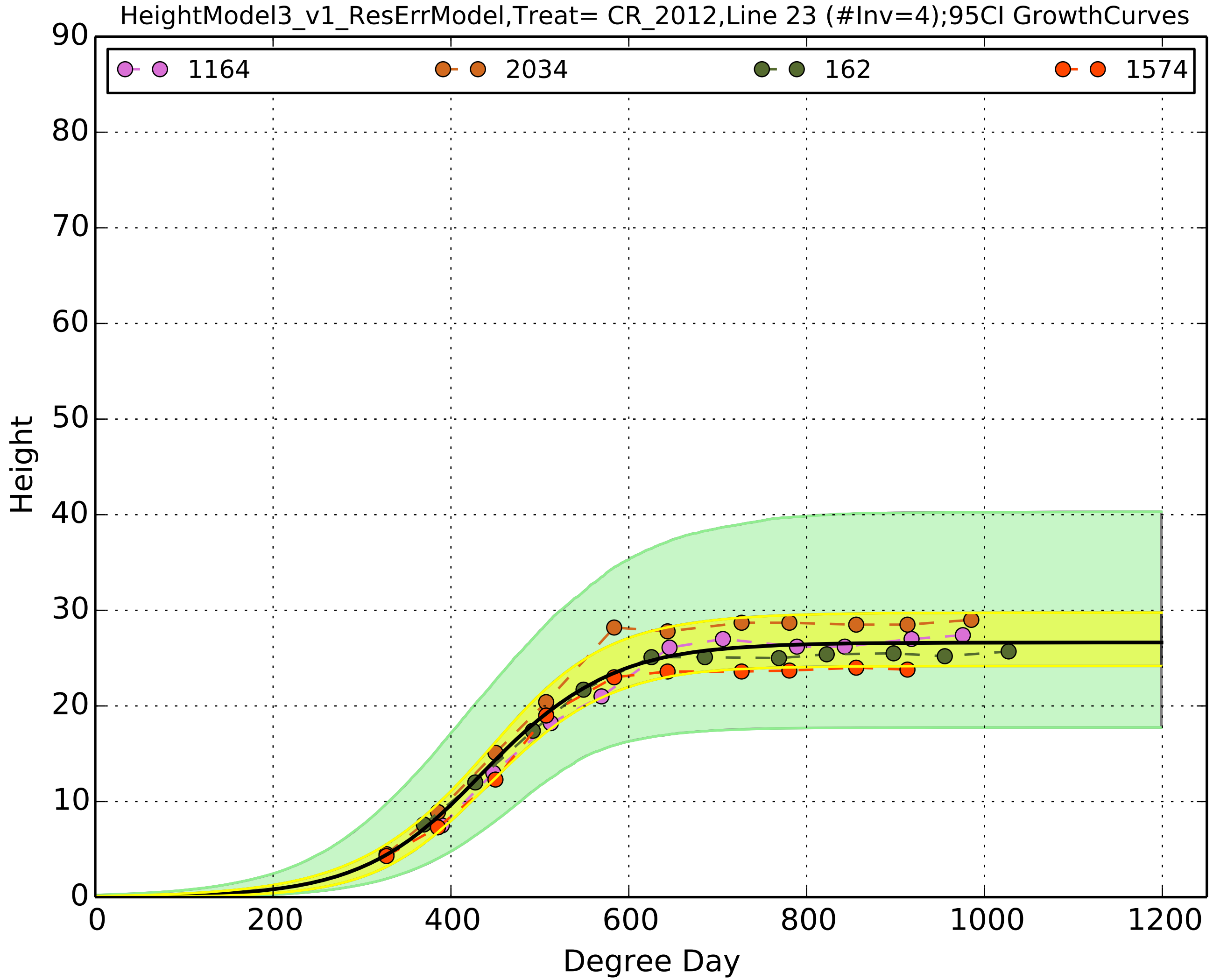
HeightModel3_v1_ResErrModel,Treat= CR_2012,Line 23 (#Inv=4);95CI GrowthCurves
1164
2034
162
1574
Height
Degree Day
0
10
20
30
40
50
60
70
80
90
0
200
400
600
800
1000
1200

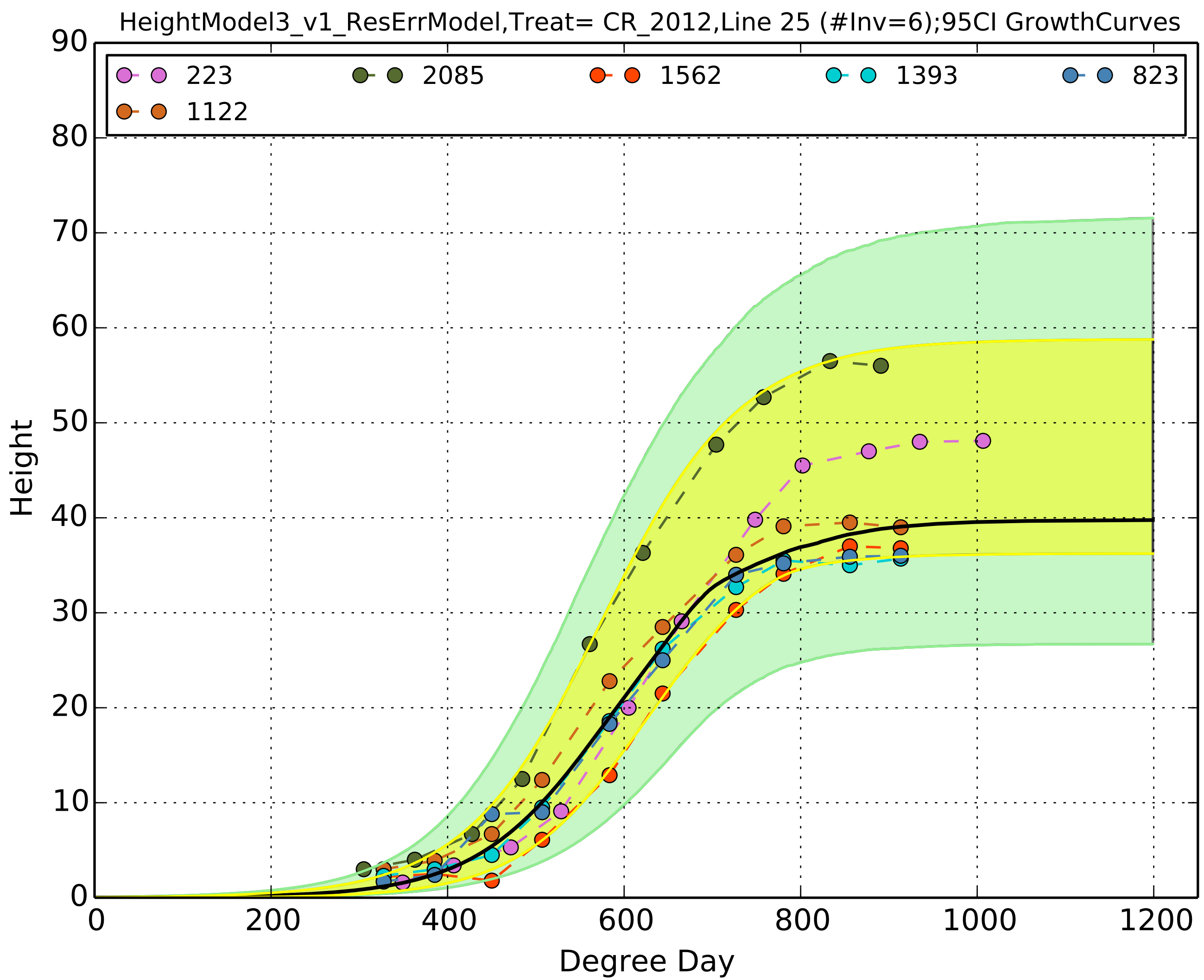

HeightModel3_v1_ResErrModel,Treat= CR_2012,Line 25 (#Inv=6);95CI GrowthCurves
223
2085
1562
1393
823
1122
Height
Degree Day
0
10
20
30
40
50
60
70
80
90
0
200
400
600
800
1000
1200

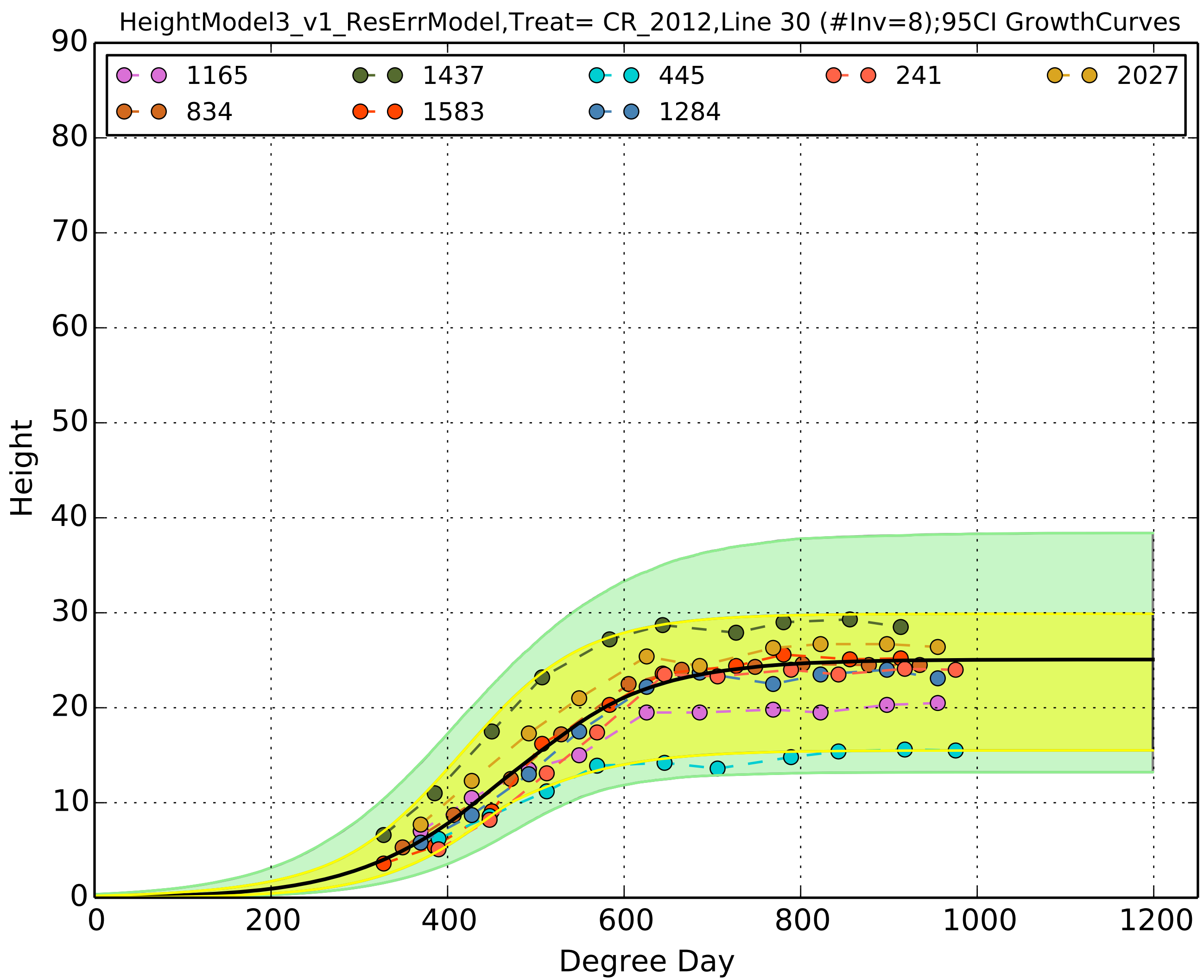

HeightModel3_v1_ResErrModel,Treat= CR_2012,Line 30 (#Inv=8);95CI GrowthCurves
1165
1437
445
241
2027
834
1583
1284
Height
Degree Day
0
10
20
30
40
50
60
70
80
90
0
200
400
600
800
1000
1200

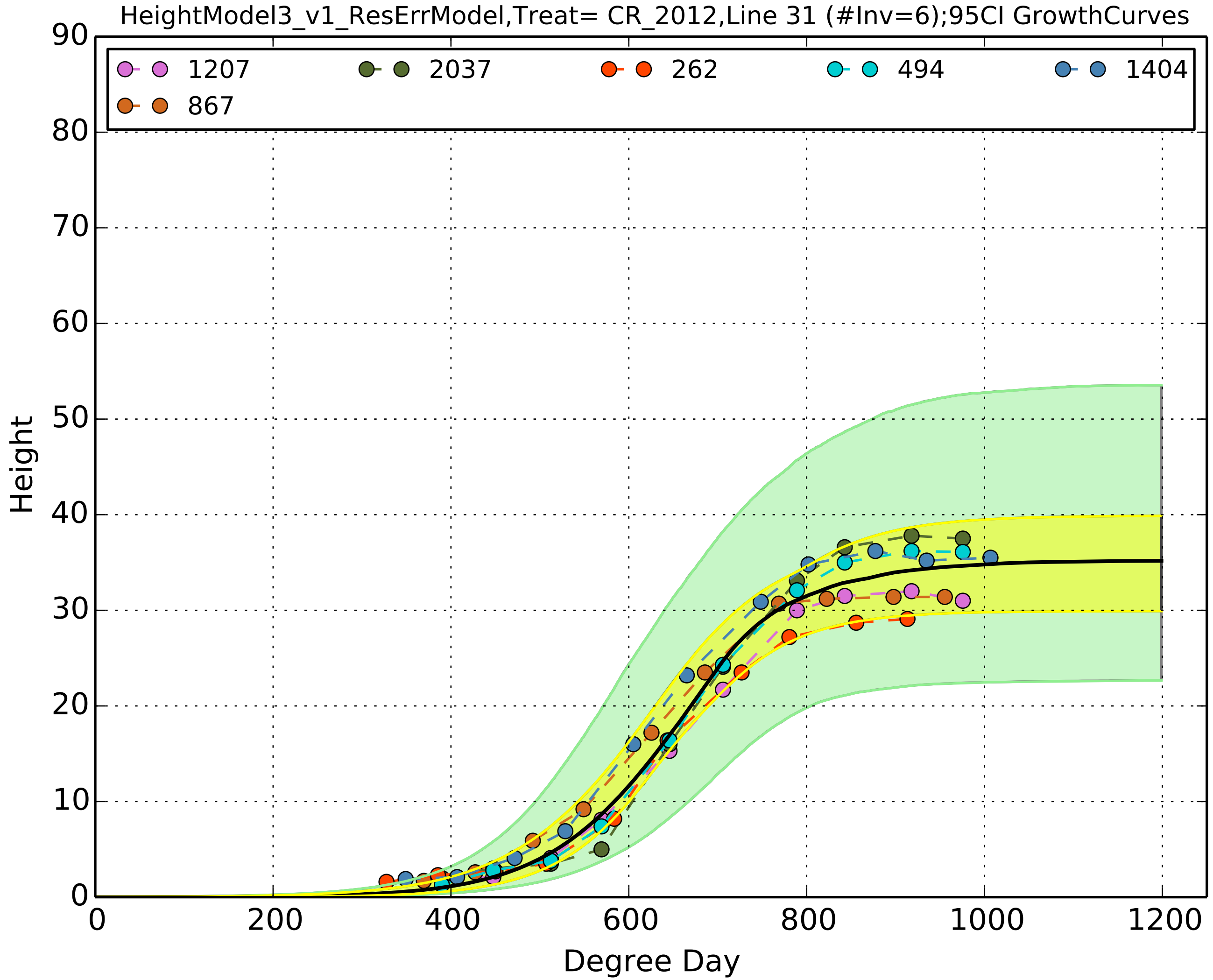
HeightModel3_v1_ResErrModel,Treat= CR_2012,Line 31 (#Inv=6);95CI GrowthCurves
1207
2037
262
494
1404
867
Height
Degree Day
0
10
20
30
40
50
60
70
80
90
0
200
400
600
800
1000
1200

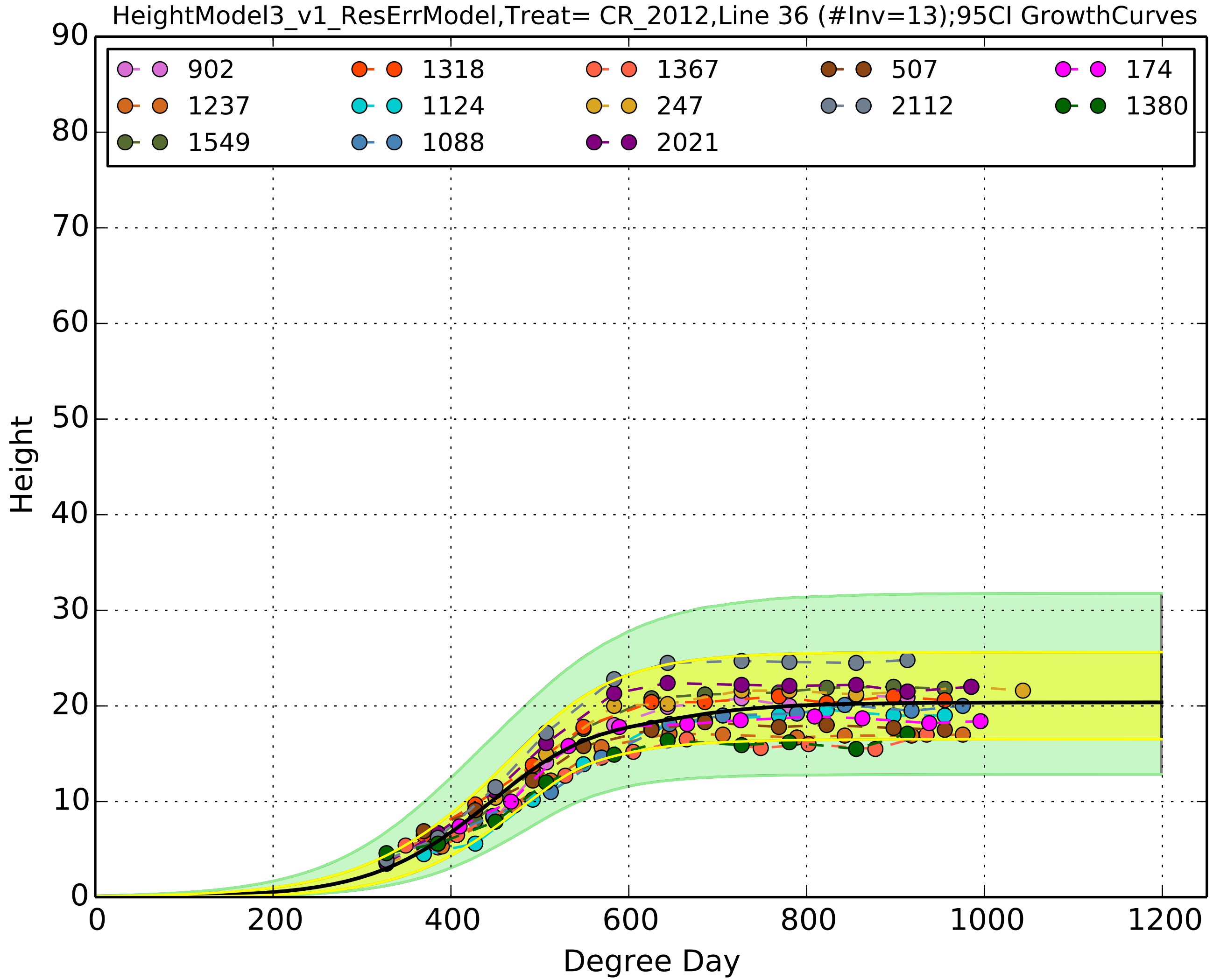

HeightModel3_v1_ResErrModel,Treat= CR_2012,Line 36 (#Inv=13);95CI GrowthCurves
902
1318
1367
507
174
1237
1124
247
2112
1380
1549
1088
2021
Height
Degree Day
0
10
20
30
40
50
60
70
80
90
0
200
400
600
800
1000
1200

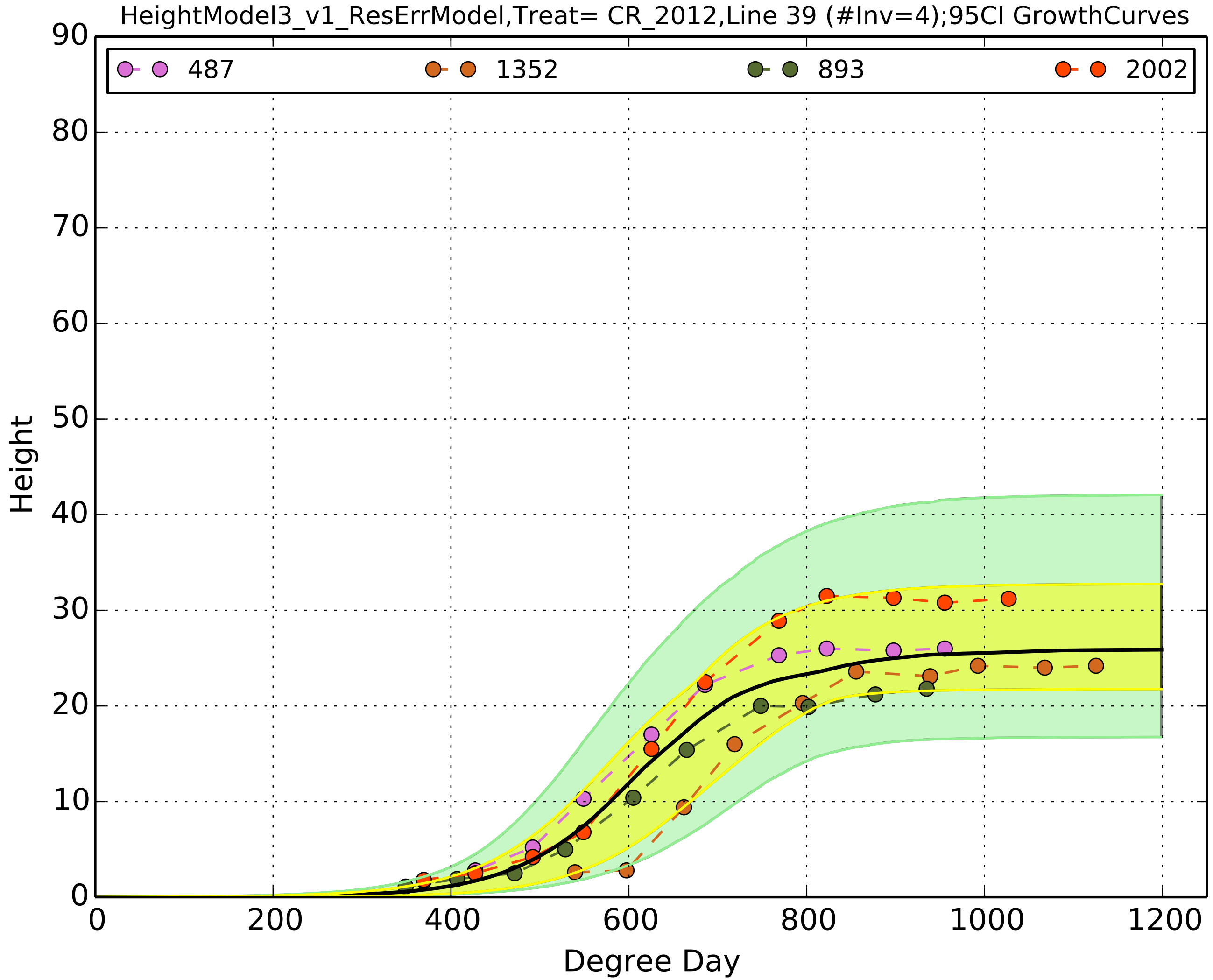

HeightModel3_v1_ResErrModel,Treat= CR_2012,Line 39 (#Inv=4);95CI GrowthCurves
487
1352
893
2002
Height
Degree Day
0
10
20
30
40
50
60
70
80
90
0
200
400
600
800
1000
1200

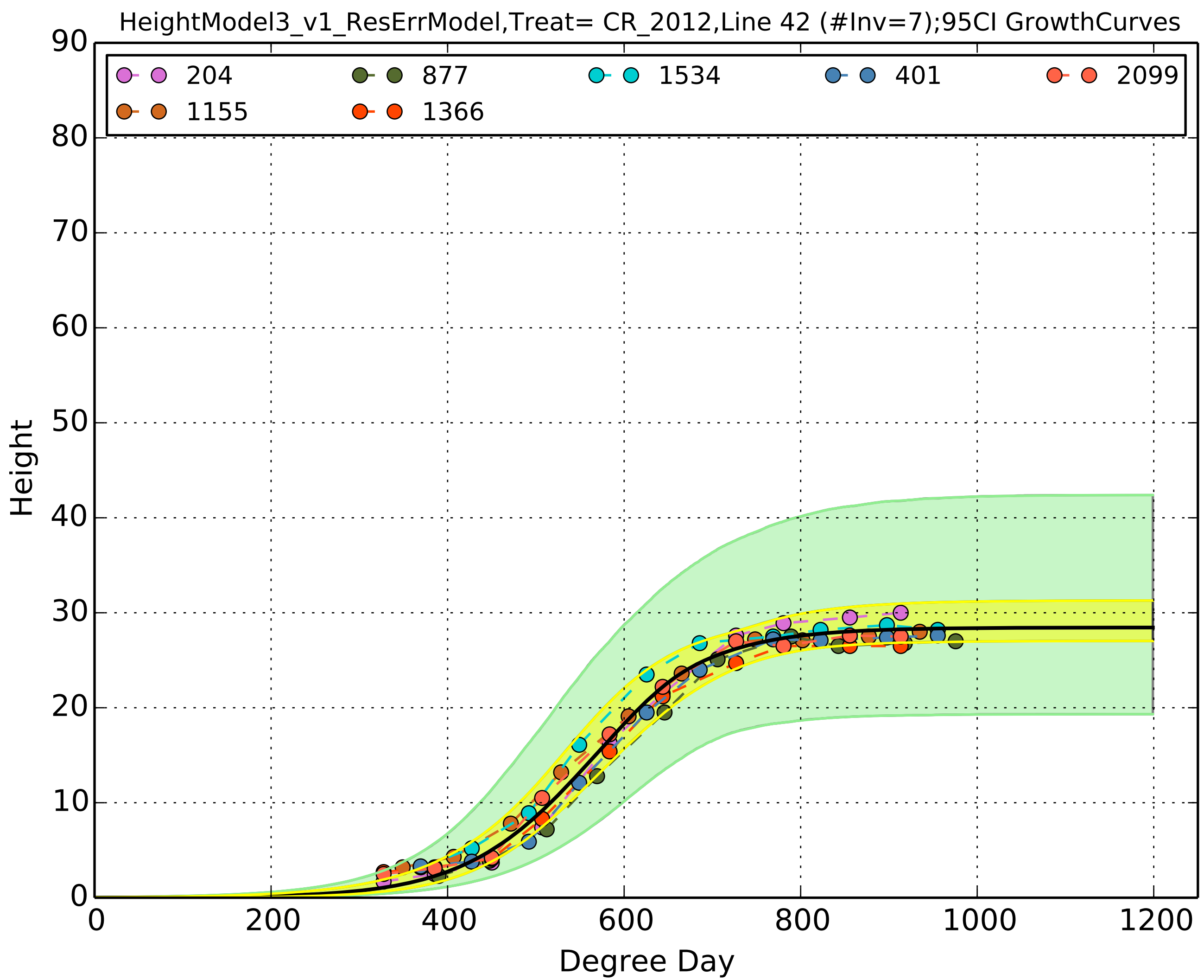

HeightModel3_v1_ResErrModel,Treat= CR_2012,Line 42 (#Inv=7);95CI GrowthCurves
204
877
1534
401
2099
1155
1366
Height
Degree Day
0
10
20
30
40
50
60
70
80
90
0
200
400
600
800
1000
1200

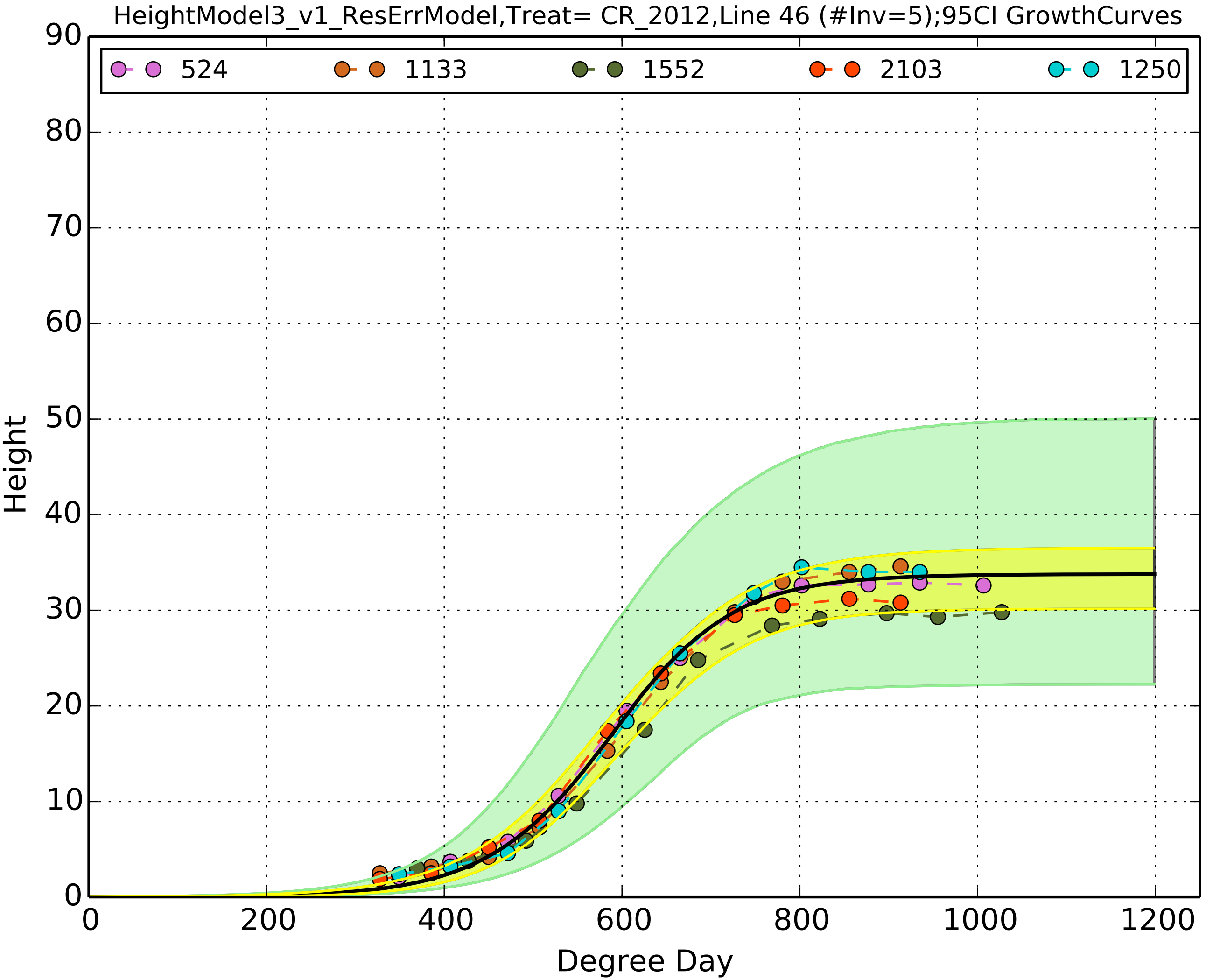

HeightModel3_v1_ResErrModel,Treat= CR_2012,Line 46 (#Inv=5);95CI GrowthCurves
524
1133
1552
2103
1250
Height
Degree Day
0
10
20
30
40
50
60
70
80
90
0
200
400
600
800
1000
1200

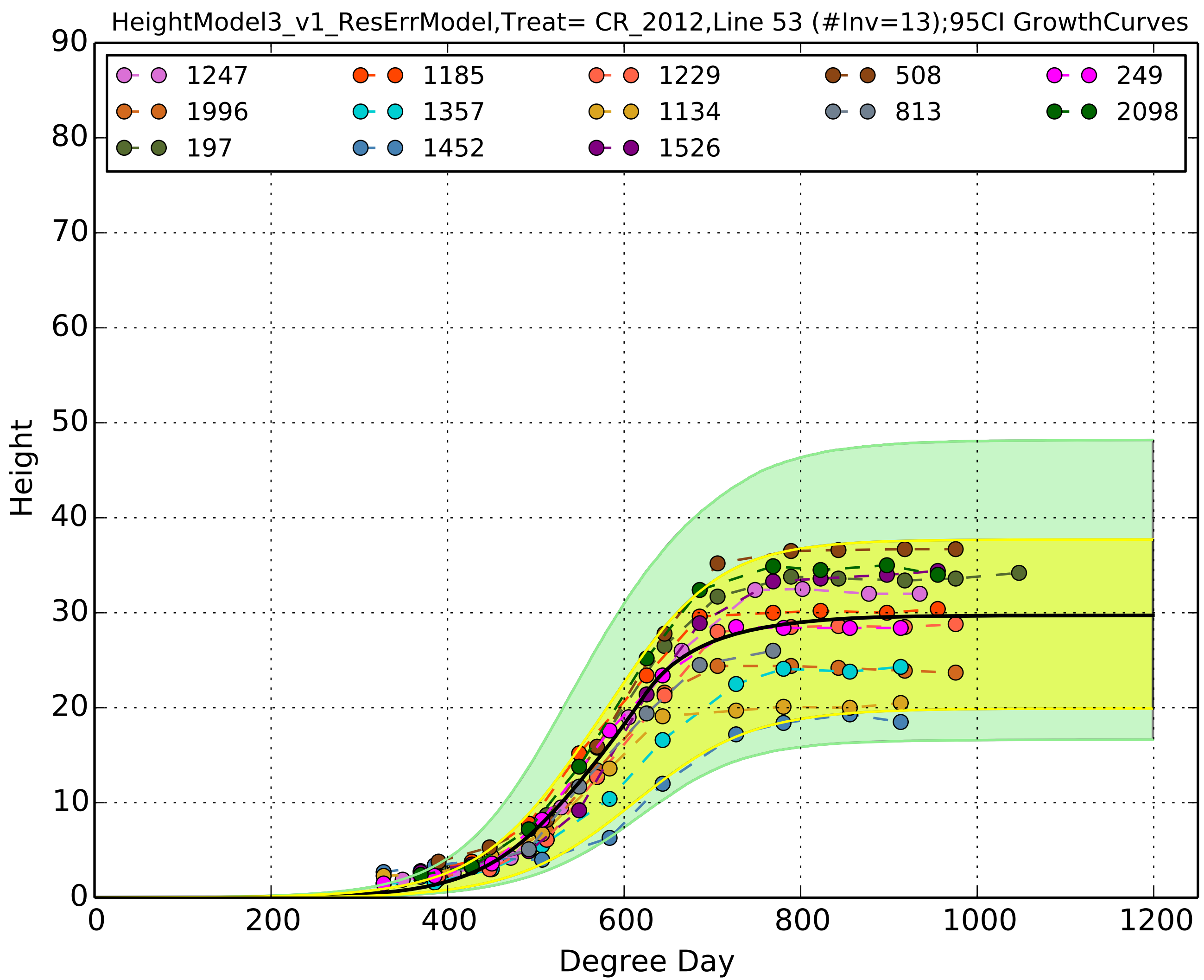

HeightModel3_v1_ResErrModel,Treat= CR_2012,Line 53 (#Inv=13);95CI GrowthCurves
1247
1185
1229
508
249
1996
1357
1134
813
2098
197
1452
1526
Height
Degree Day
0
10
20
30
40
50
60
70
80
90
0
200
400
600
800
1000
1200

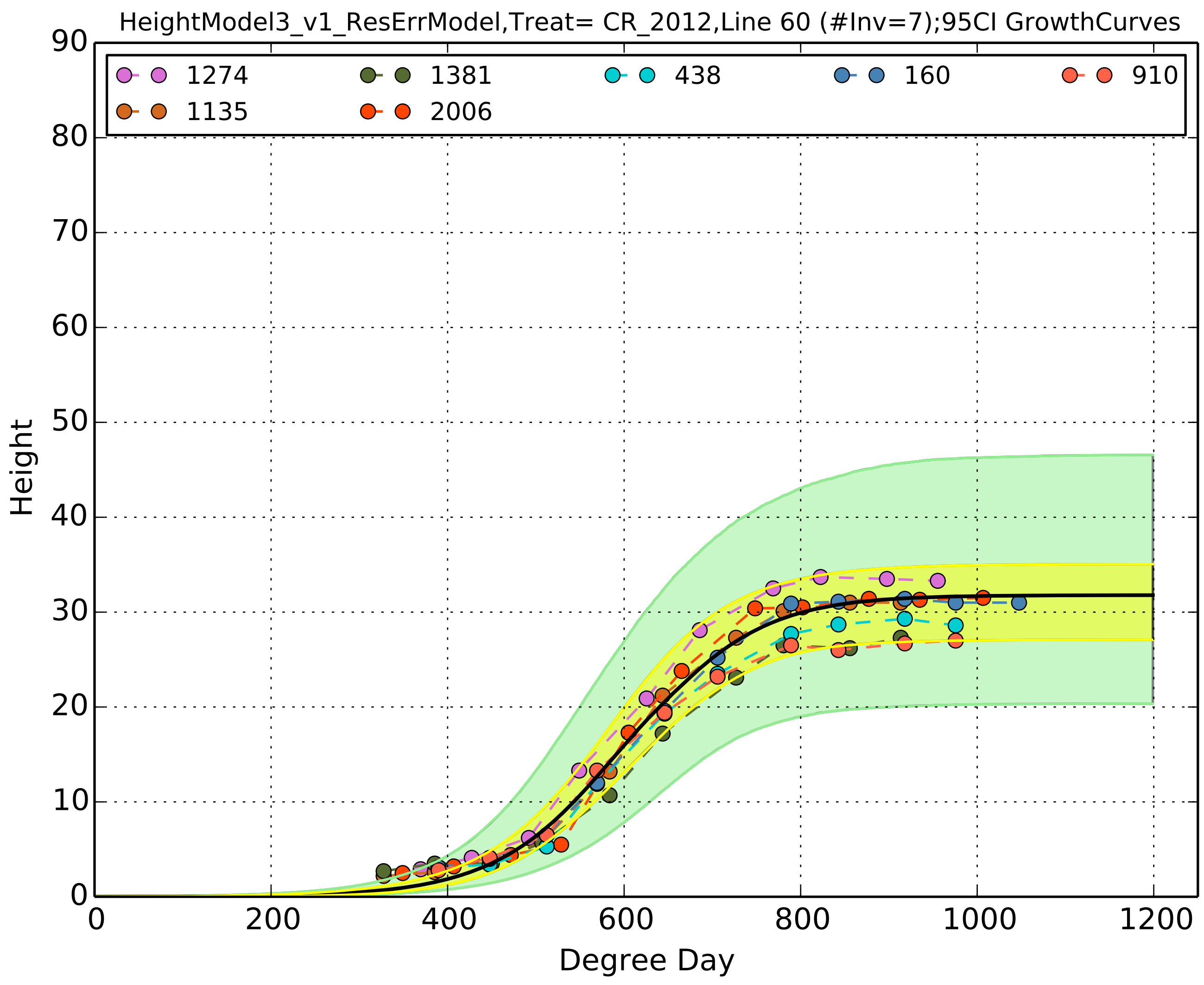

HeightModel3_v1_ResErrModel,Treat= CR_2012,Line 60 (#Inv=7);95CI GrowthCurves
1274
1381
438
160
910
1135
2006
Height
Degree Day
0
10
20
30
40
50
60
70
80
90
0
200
400
600
800
1000
1200

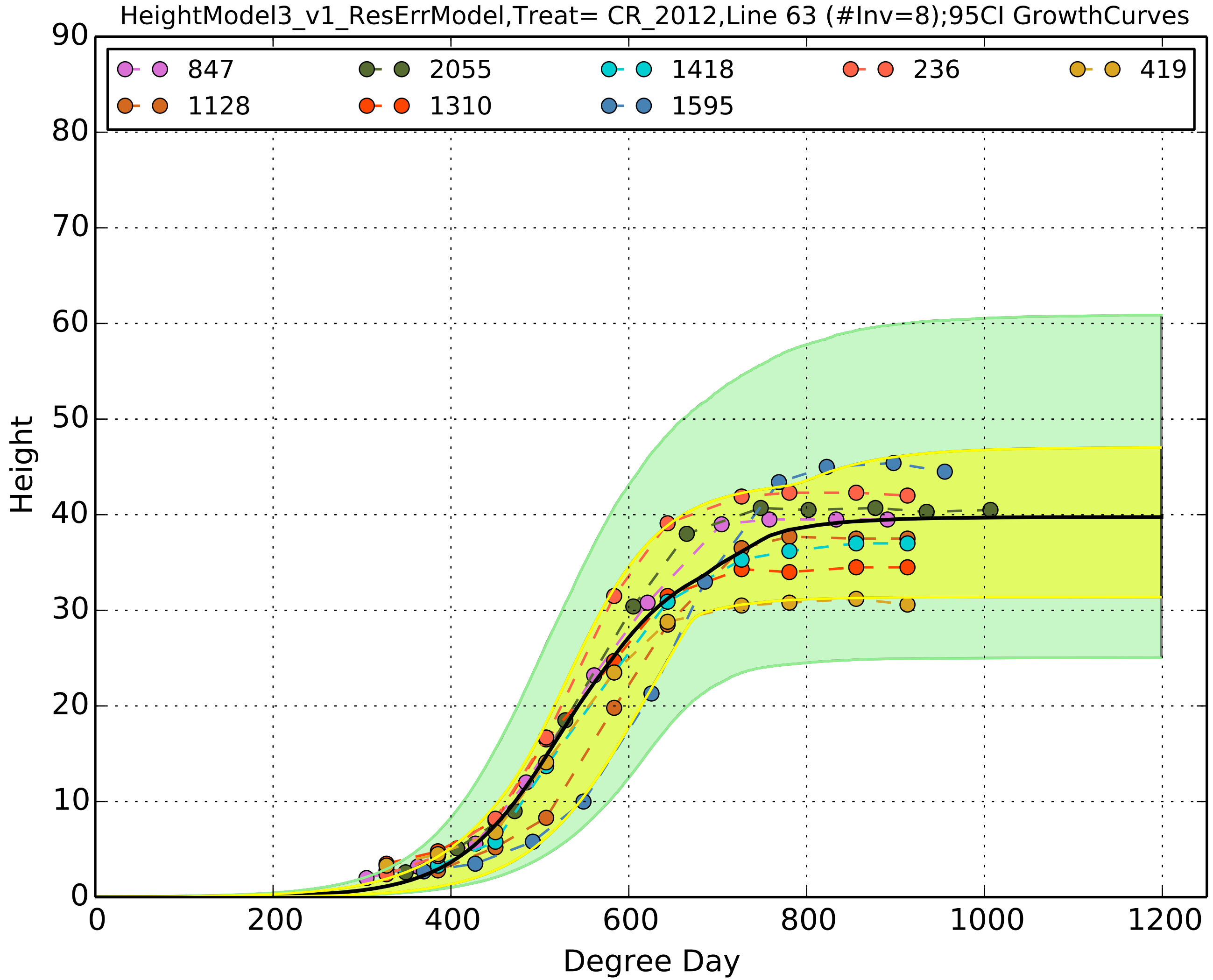

HeightModel3_v1_ResErrModel,Treat= CR_2012,Line 63 (#Inv=8);95CI GrowthCurves
847
2055
1418
236
419
1128
1310
1595
Height
Degree Day
0
10
20
30
40
50
60
70
80
90
0
200
400
600
800
1000
1200

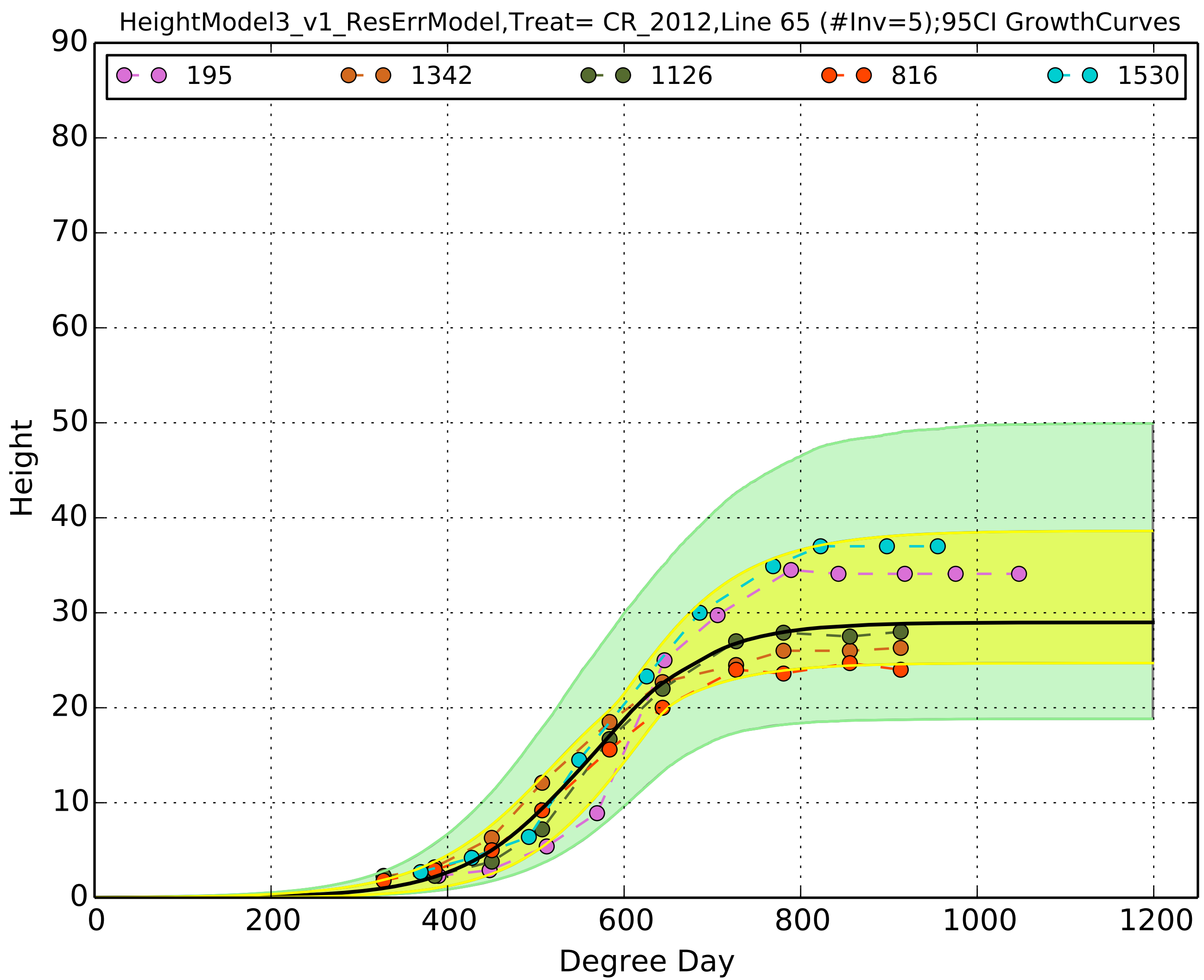
HeightModel3_v1_ResErrModel,Treat= CR_2012,Line 65 (#Inv=5);95CI GrowthCurves
195
1342
1126
816
1530
Height
Degree Day
0
10
20
30
40
50
60
70
80
90
0
200
400
600
800
1000
1200

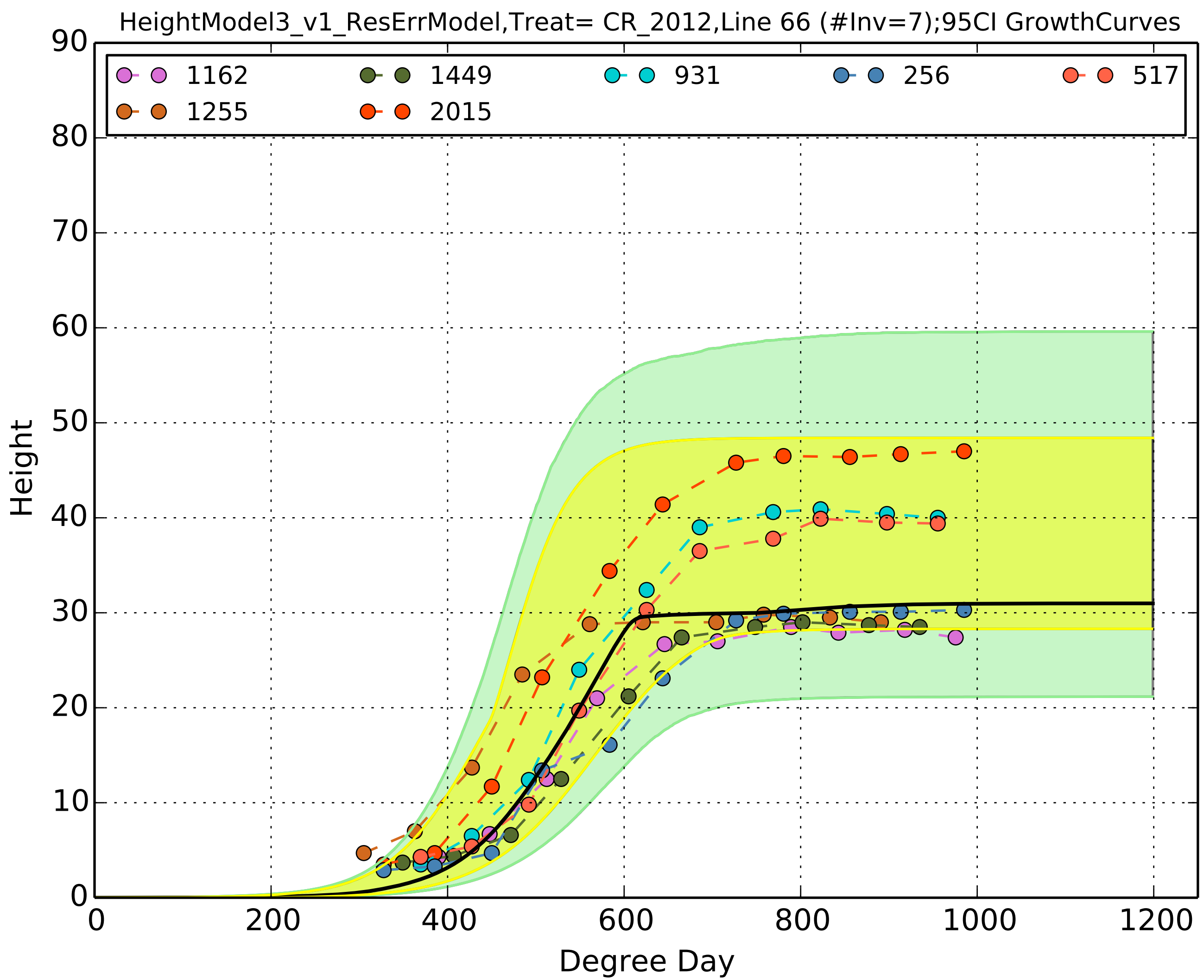

HeightModel3_v1_ResErrModel,Treat= CR_2012,Line 66 (#Inv=7);95CI GrowthCurves
1162
1449
931
256
517
1255
2015
Height
Degree Day
0
10
20
30
40
50
60
70
80
90
0
200
400
600
800
1000
1200

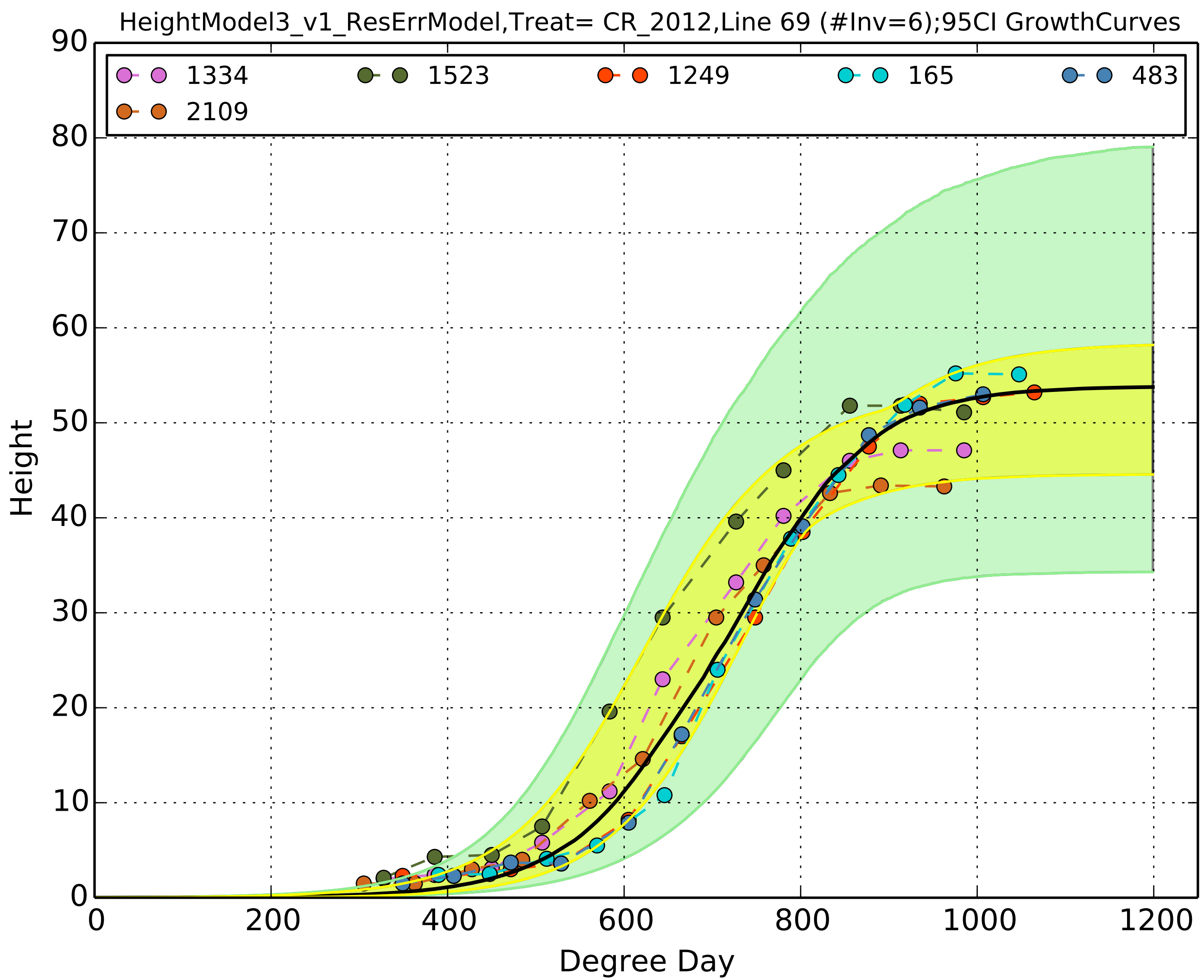
HeightModel3_v1_ResErrModel,Treat= CR_2012,Line 69 (#Inv=6);95CI GrowthCurves
1334
1523
1249
165
483
2109
Height
Degree Day
0
10
20
30
40
50
60
70
80
90
0
200
400
600
800
1000
1200

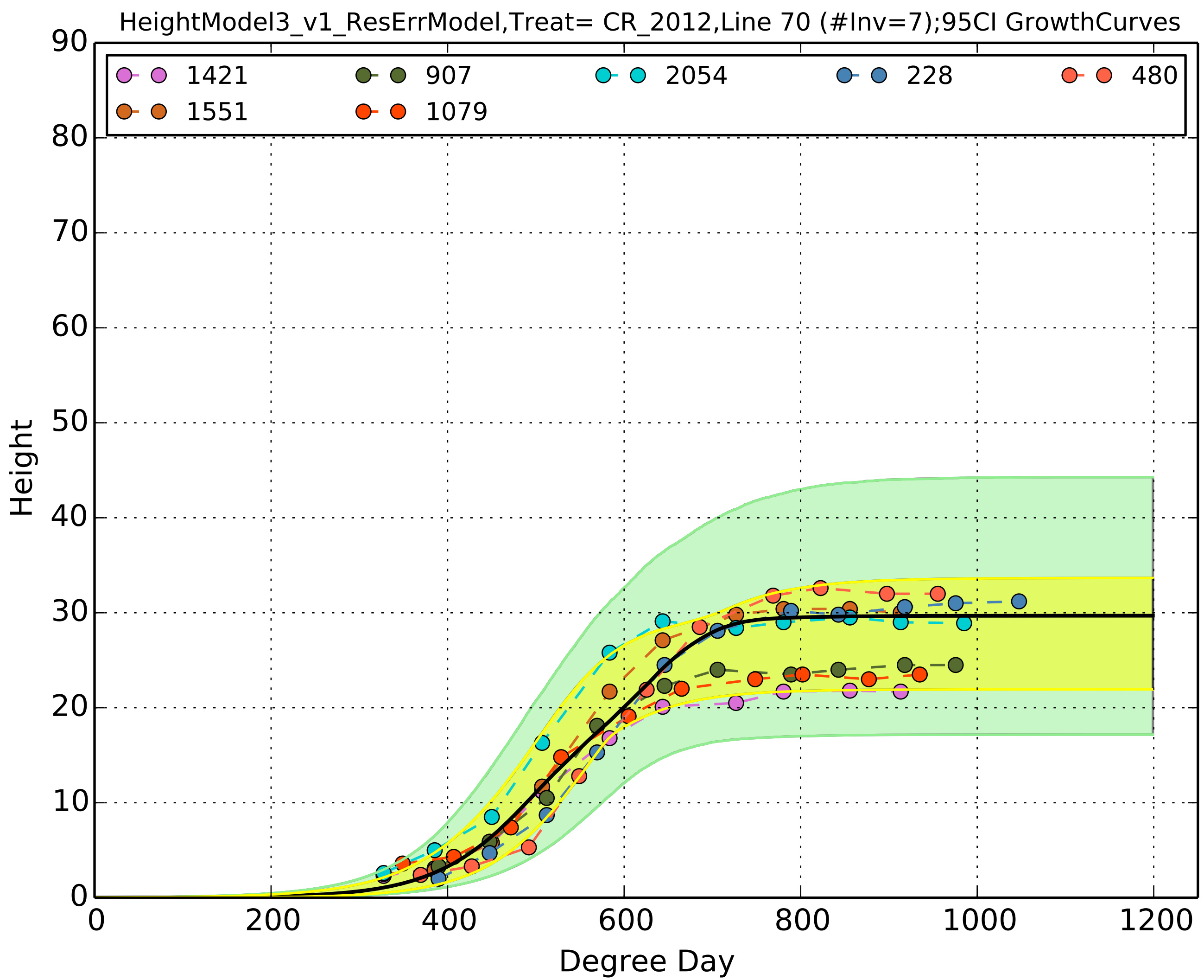

HeightModel3_v1_ResErrModel,Treat= CR_2012,Line 70 (#Inv=7);95CI GrowthCurves
1421
907
2054
228
480
1551
1079
Height
Degree Day
0
10
20
30
40
50
60
70
80
90
0
200
400
600
800
1000
1200

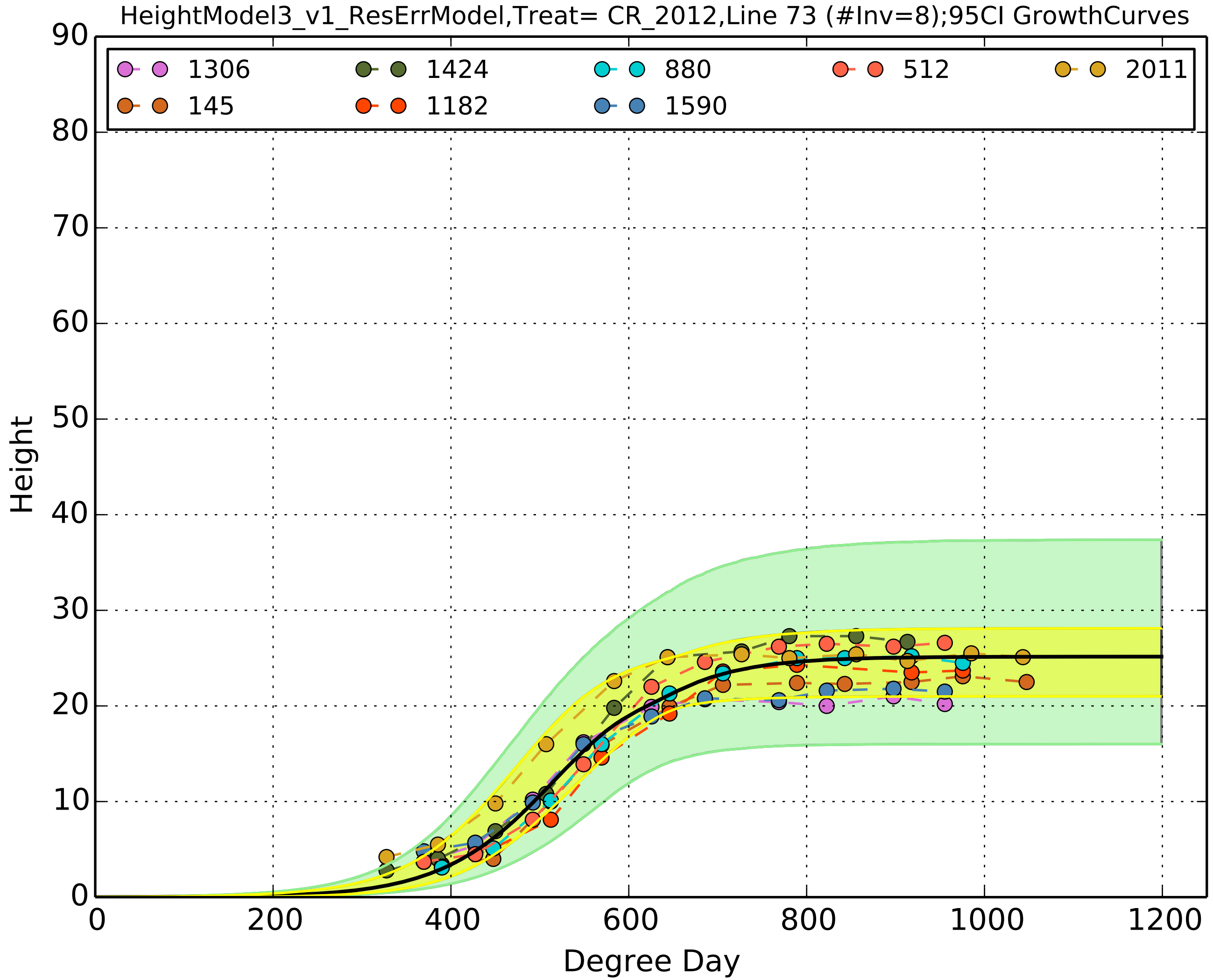

HeightModel3_v1_ResErrModel,Treat= CR_2012,Line 73 (#Inv=8);95CI GrowthCurves
1306
1424
880
512
2011
145
1182
1590
Height
Degree Day
0
10
20
30
40
50
60
70
80
90
0
200
400
600
800
1000
1200

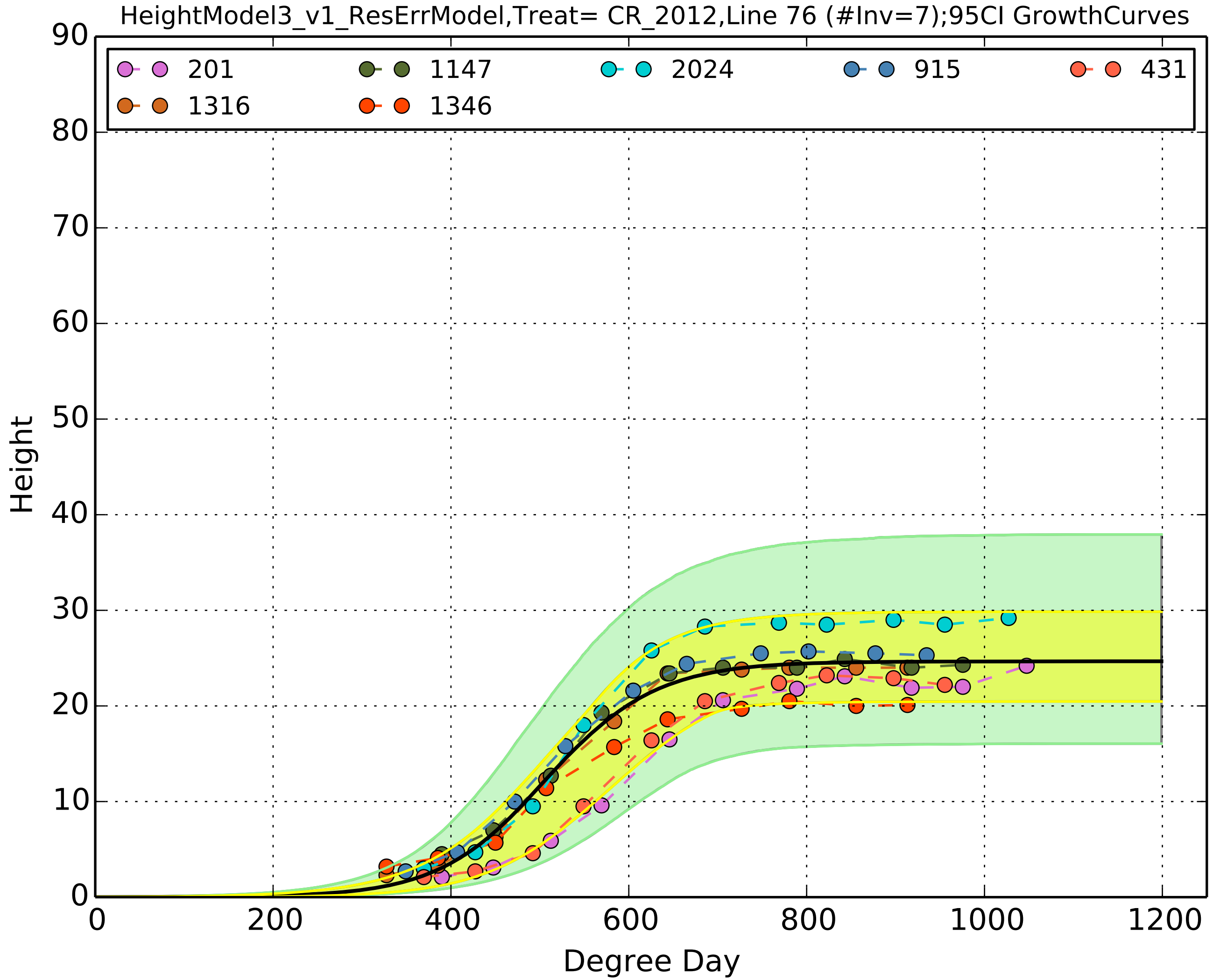

HeightModel3_v1_ResErrModel,Treat= CR_2012,Line 76 (#Inv=7);95CI GrowthCurves
201
1147
2024
915
431
1316
1346
Height
Degree Day
0
10
20
30
40
50
60
70
80
90
0
200
400
600
800
1000
1200

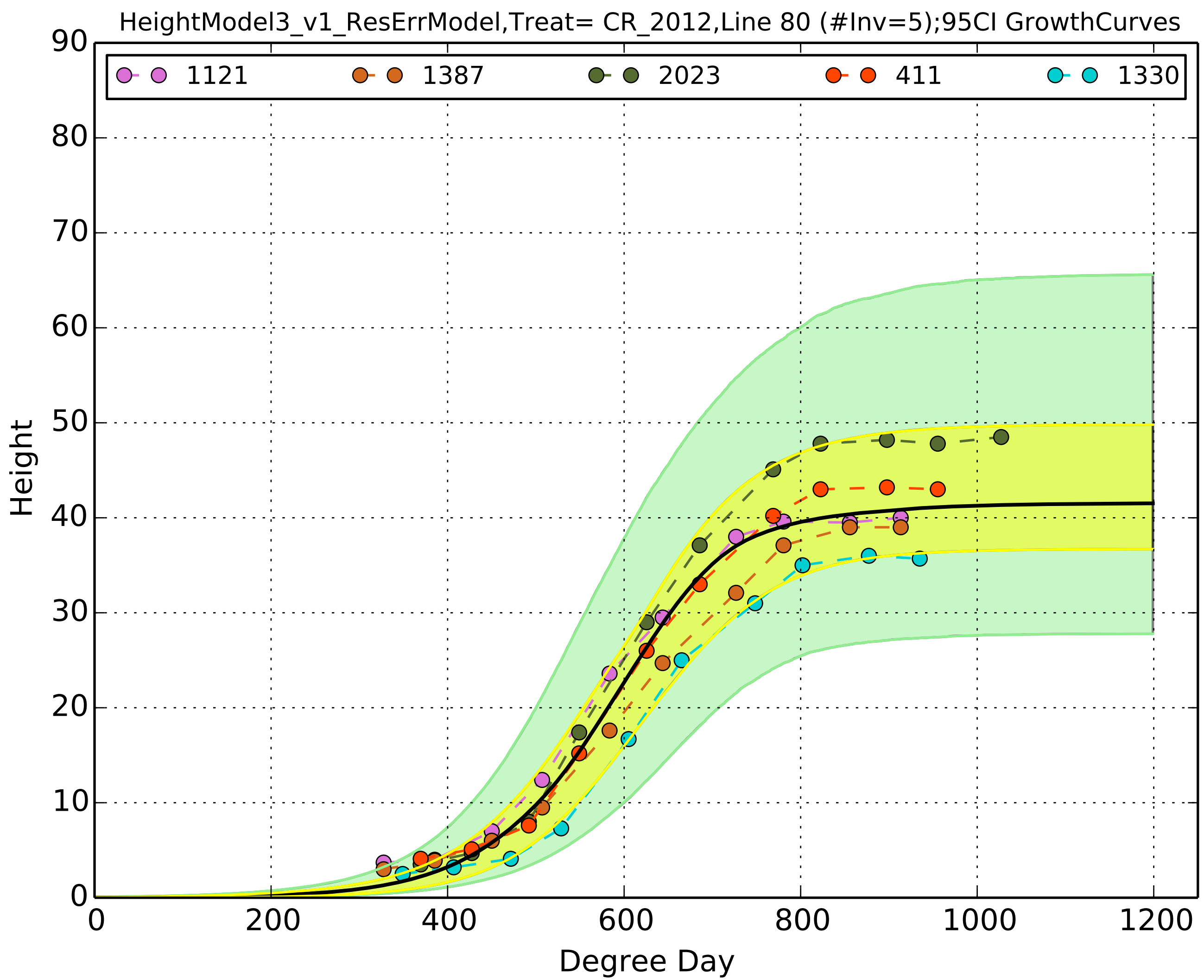

HeightModel3_v1_ResErrModel,Treat= CR_2012,Line 80 (#Inv=5);95CI GrowthCurves
1121
1387
2023
411
1330
Height
Degree Day
0
10
20
30
40
50
60
70
80
90
0
200
400
600
800
1000
1200

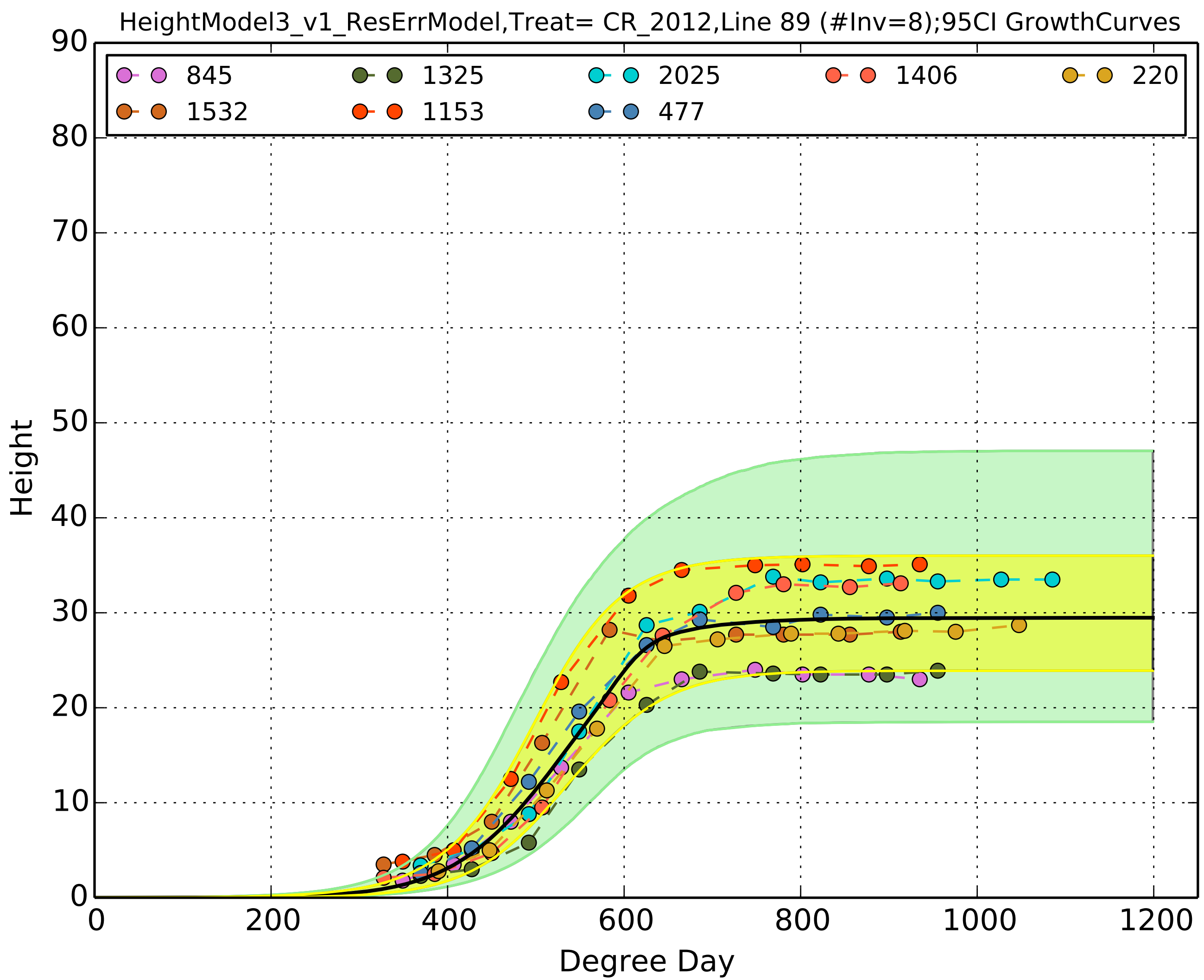

HeightModel3_v1_ResErrModel,Treat= CR_2012,Line 89 (#Inv=8);95CI GrowthCurves
845
1325
2025
1406
220
1532
1153
477
Height
Degree Day
0
10
20
30
40
50
60
70
80
90
0
200
400
600
800
1000
1200

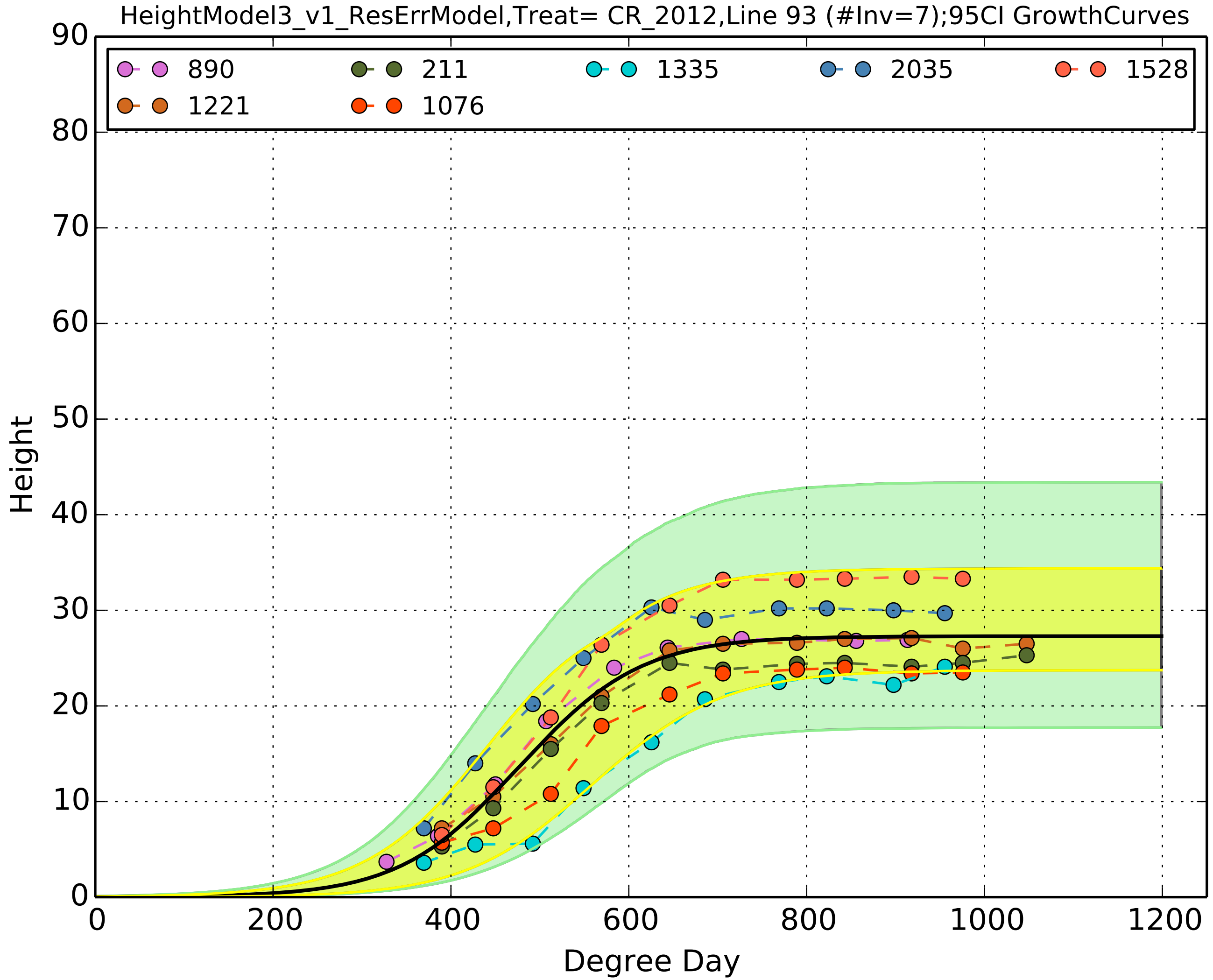

HeightModel3_v1_ResErrModel,Treat= CR_2012,Line 93 (#Inv=7);95CI GrowthCurves
890
211
1335
2035
1528
1221
1076
Height
Degree Day
0
10
20
30
40
50
60
70
80
90
0
200
400
600
800
1000
1200

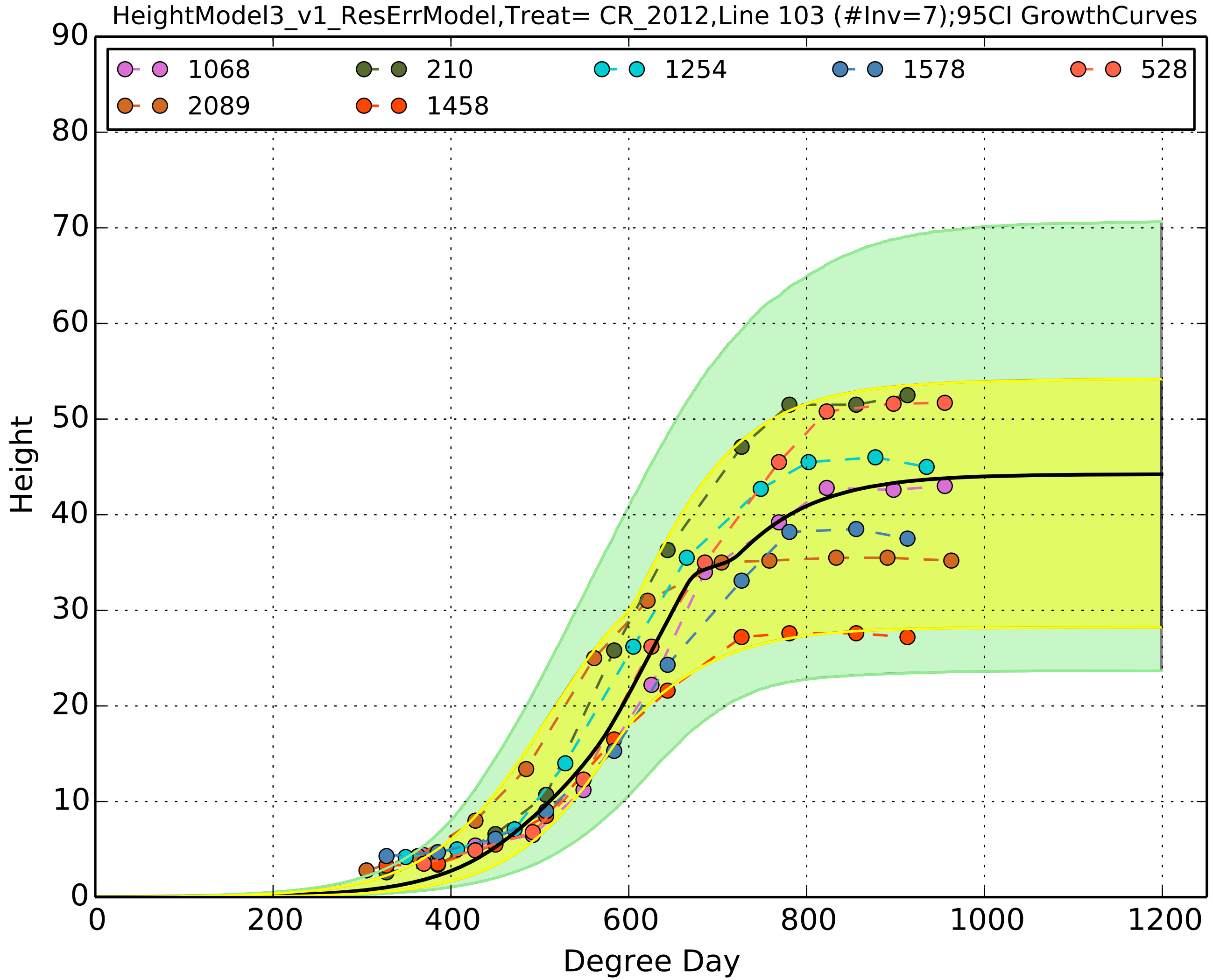

HeightModel3_v1_ResErrModel,Treat= CR_2012,Line 103 (#Inv=7);95CI GrowthCurves
1068
210
1254
1578
528
2089
1458
Height
Degree Day
0
10
20
30
40
50
60
70
80
90
0
200
400
600
800
1000
1200

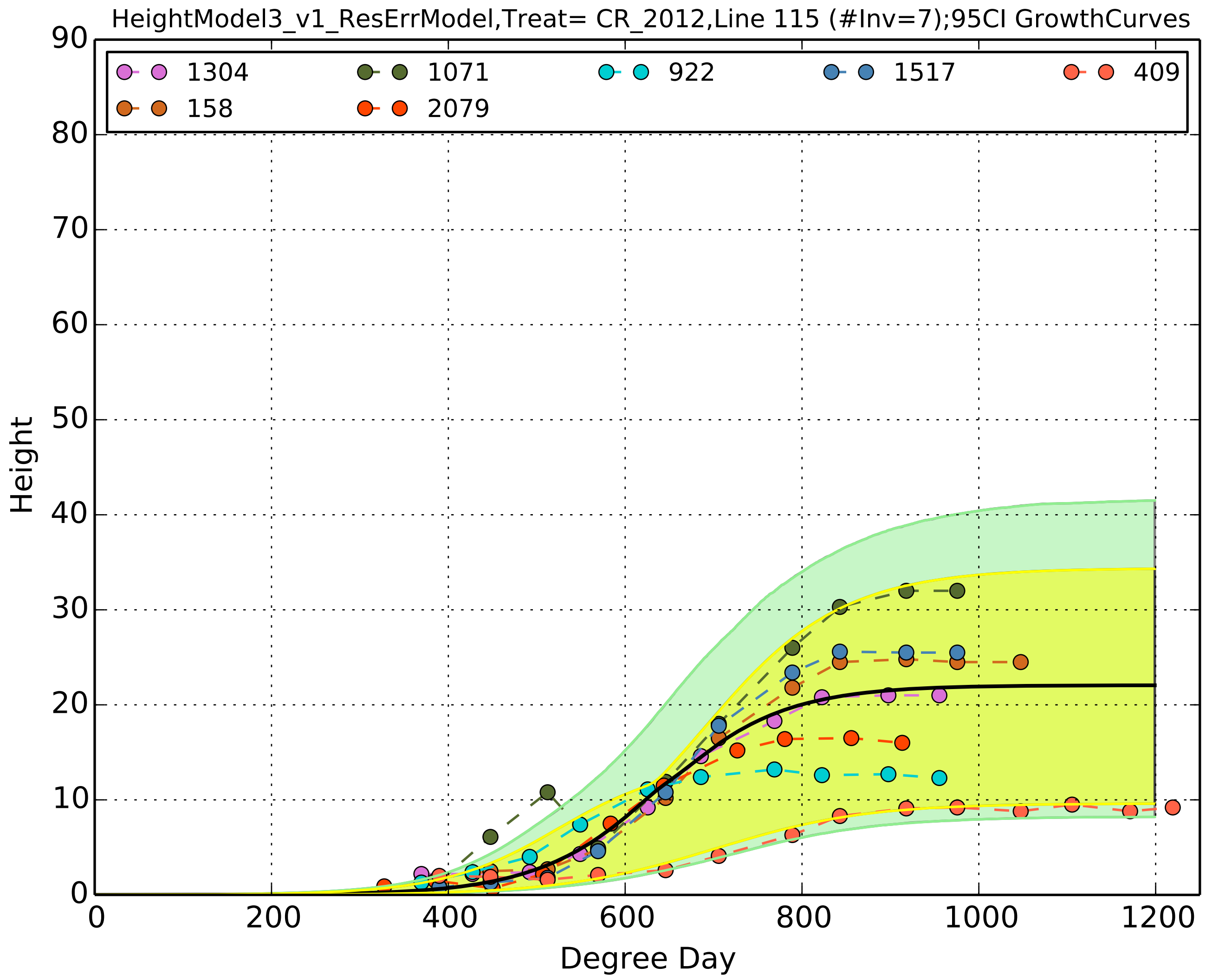
HeightModel3_v1_ResErrModel,Treat= CR_2012,Line 115 (#Inv=7);95CI GrowthCurves
1304
1071
922
1517
409
158
2079
Height
Degree Day
0
10
20
30
40
50
60
70
80
90
0
200
400
600
800
1000
1200

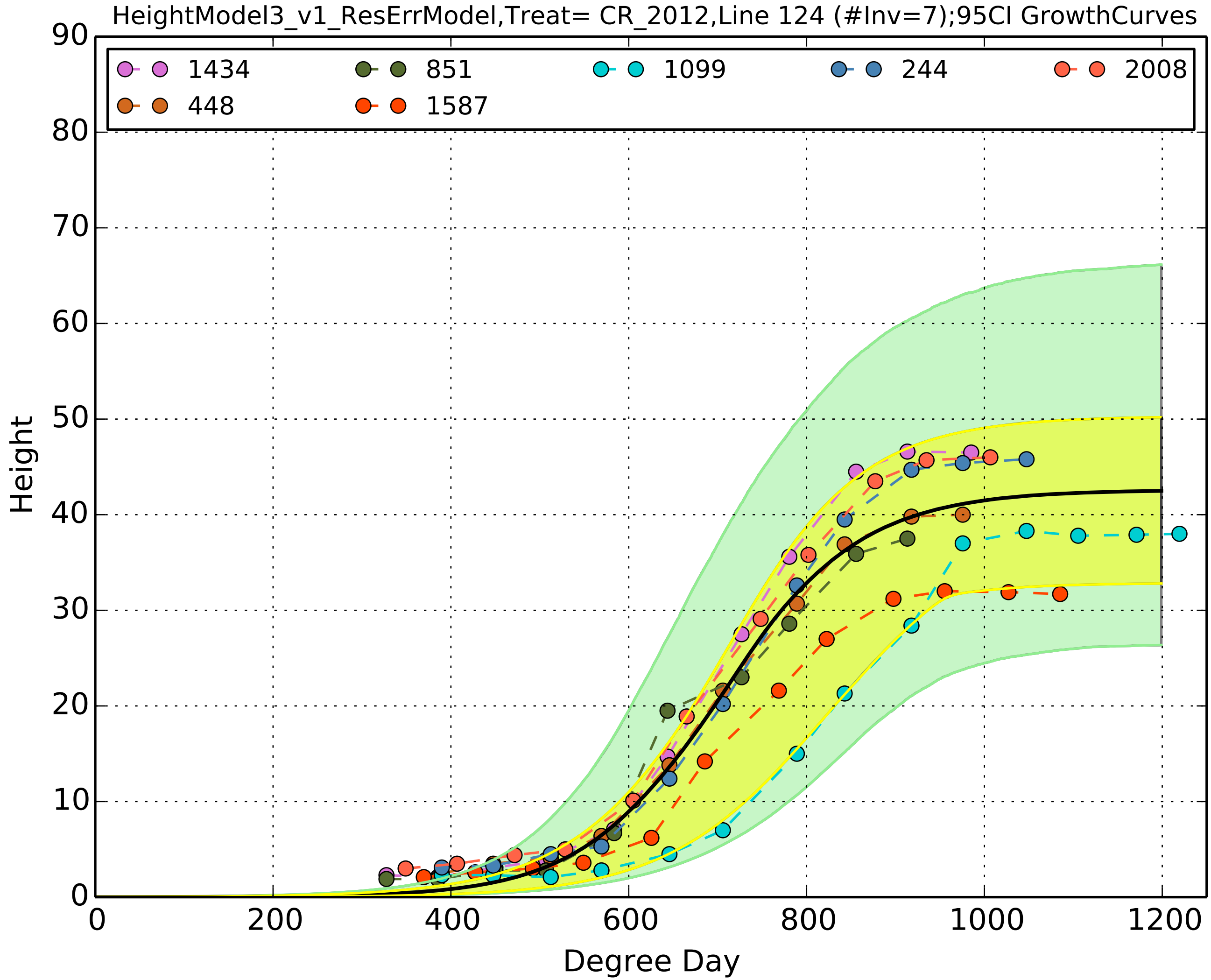
HeightModel3_v1_ResErrModel,Treat= CR_2012,Line 124 (#Inv=7);95CI GrowthCurves
1434
851
1099
244
2008
448
1587
Height
Degree Day
0
10
20
30
40
50
60
70
80
90
0
200
400
600
800
1000
1200

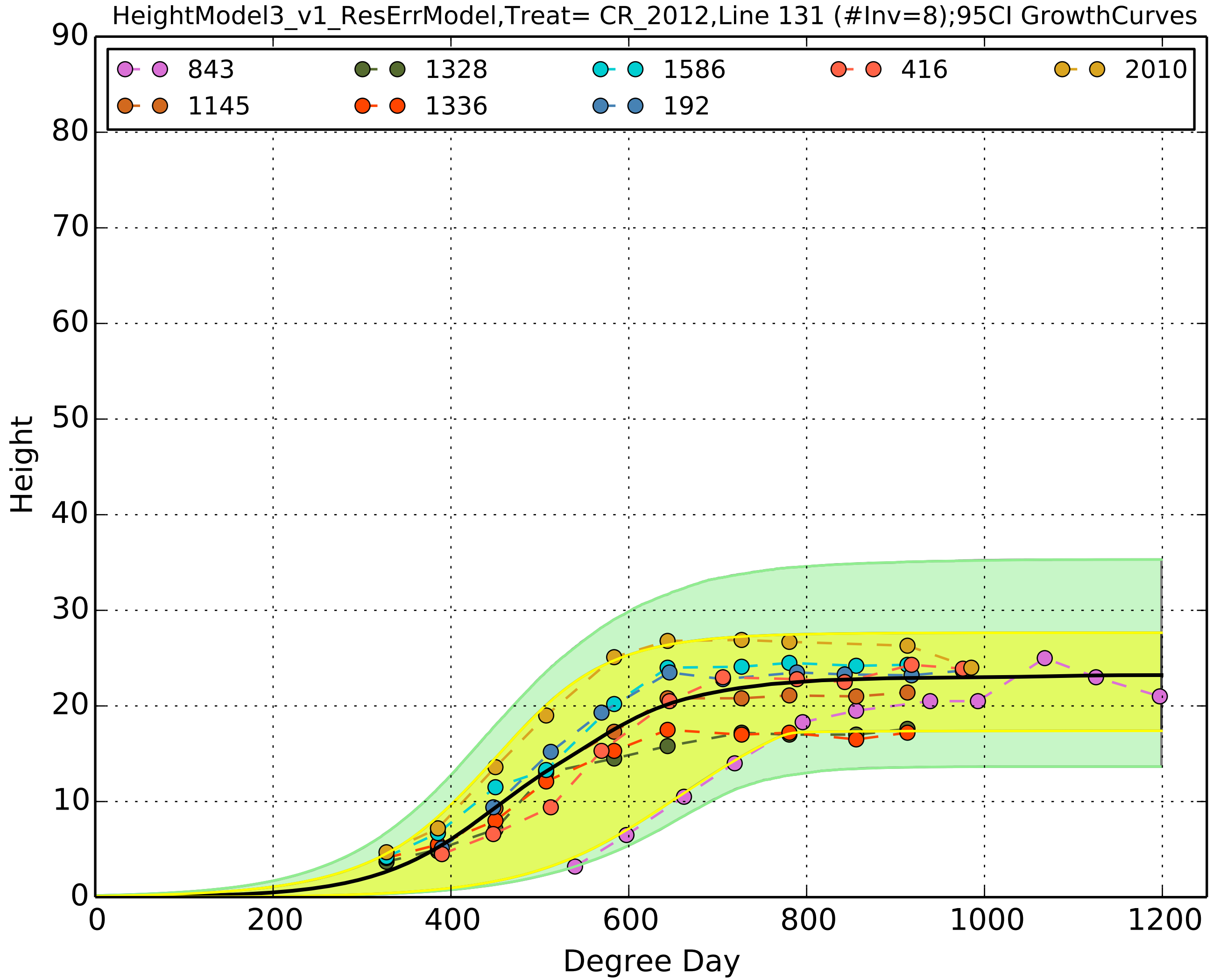

HeightModel3_v1_ResErrModel,Treat= CR_2012,Line 131 (#Inv=8);95CI GrowthCurves
843
1328
1586
416
2010
1145
1336
192
Height
Degree Day
0
10
20
30
40
50
60
70
80
90
0
200
400
600
800
1000
1200

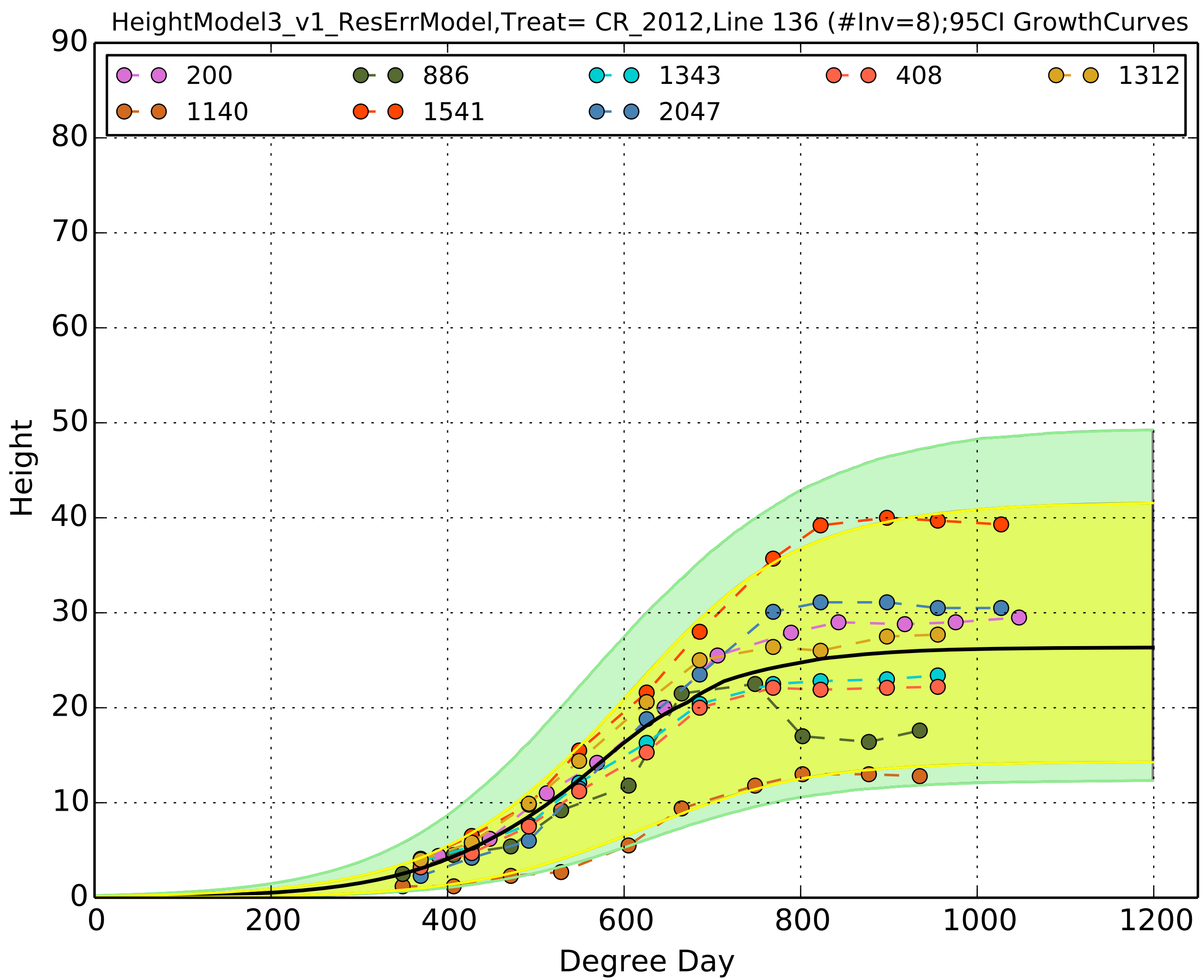

HeightModel3_v1_ResErrModel,Treat= CR_2012,Line 136 (#Inv=8);95CI GrowthCurves
200
886
1343
408
1312
1140
1541
2047
Height
Degree Day
0
10
20
30
40
50
60
70
80
90
0
200
400
600
800
1000
1200

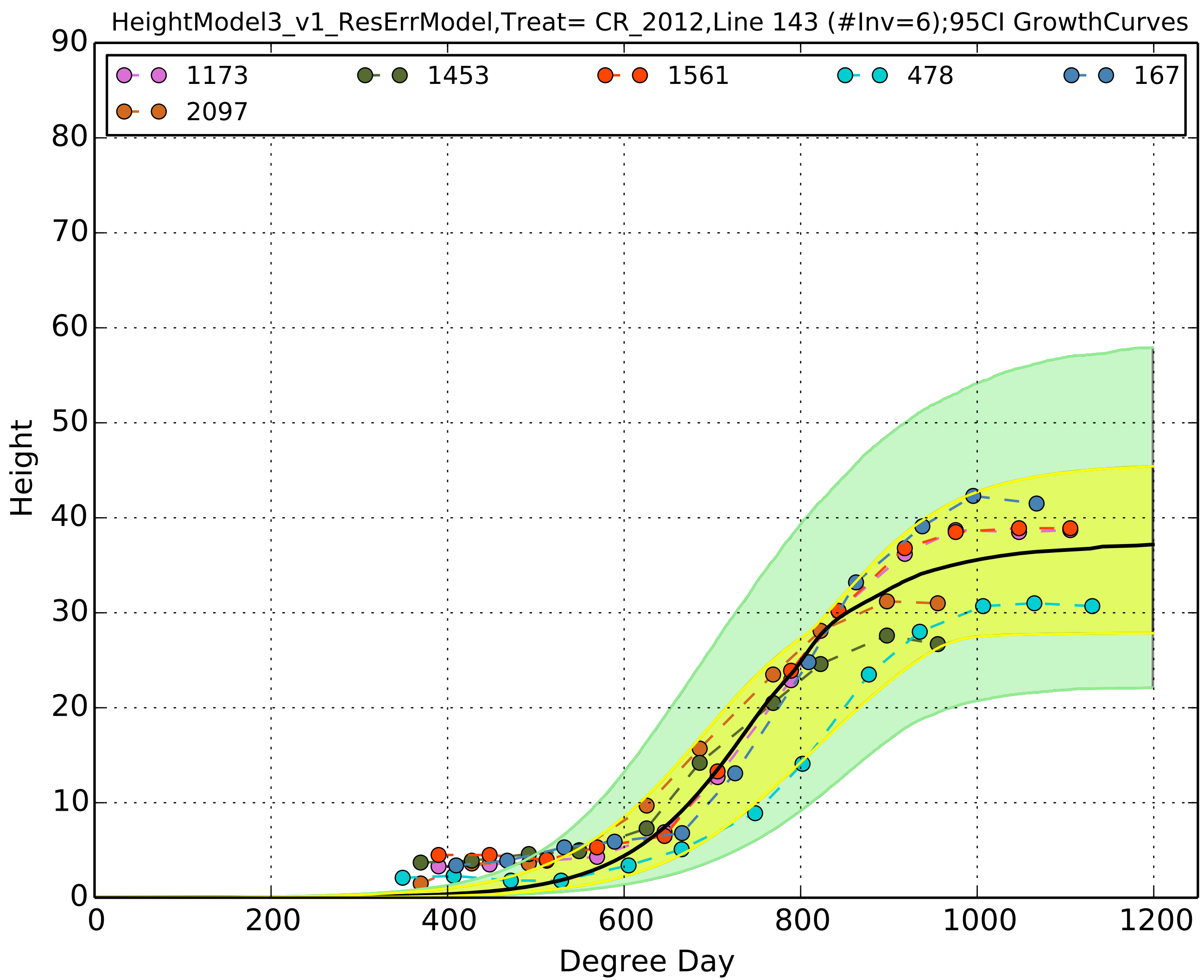
HeightModel3_v1_ResErrModel,Treat= CR_2012,Line 143 (#Inv=6);95CI GrowthCurves
1173
1453
1561
478
167
2097
Height
Degree Day
0
10
20
30
40
50
60
70
80
90
0
200
400
600
800
1000
1200

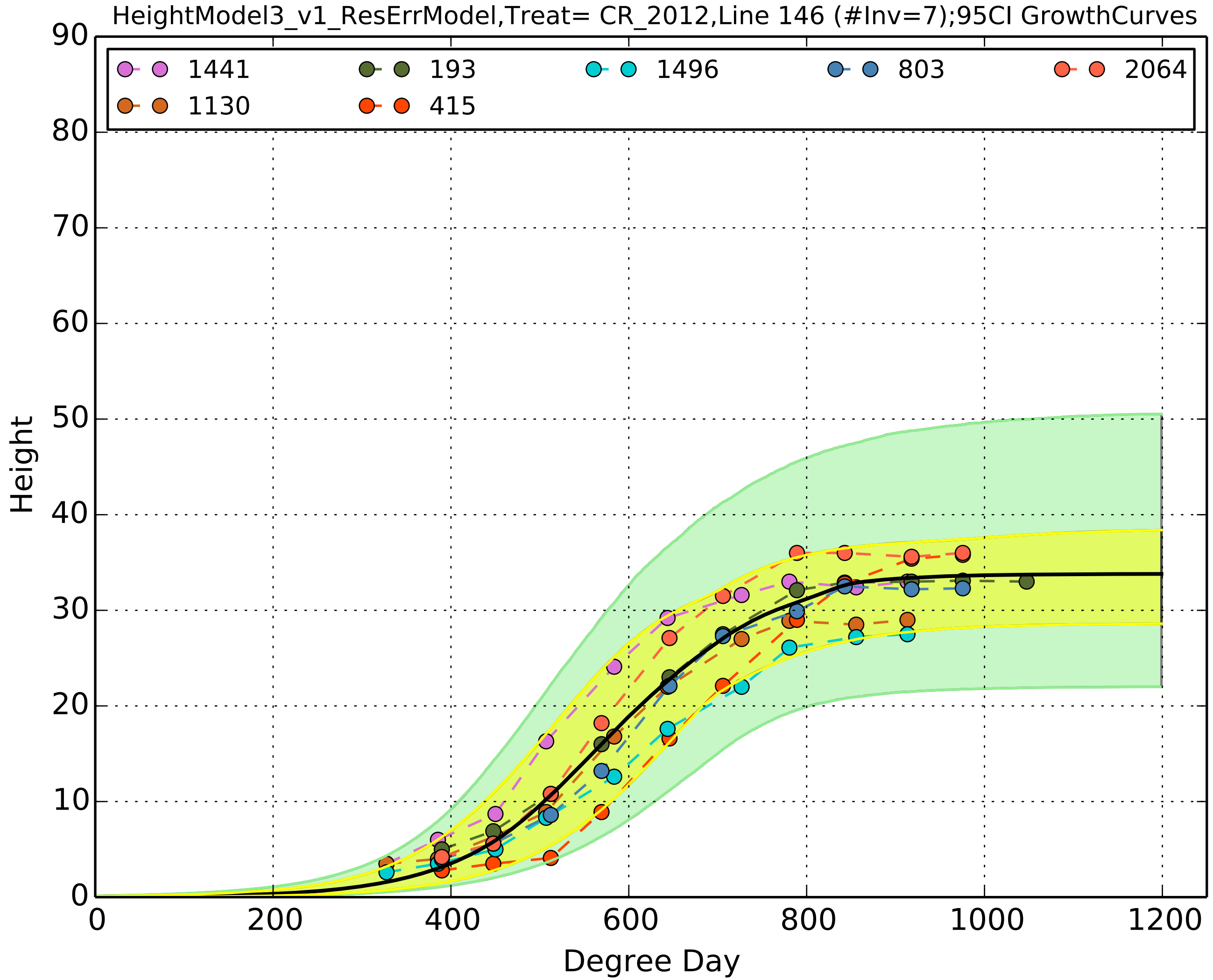

HeightModel3_v1_ResErrModel,Treat= CR_2012,Line 146 (#Inv=7);95CI GrowthCurves
1441
193
1496
803
2064
1130
415
Height
Degree Day
0
10
20
30
40
50
60
70
80
90
0
200
400
600
800
1000
1200

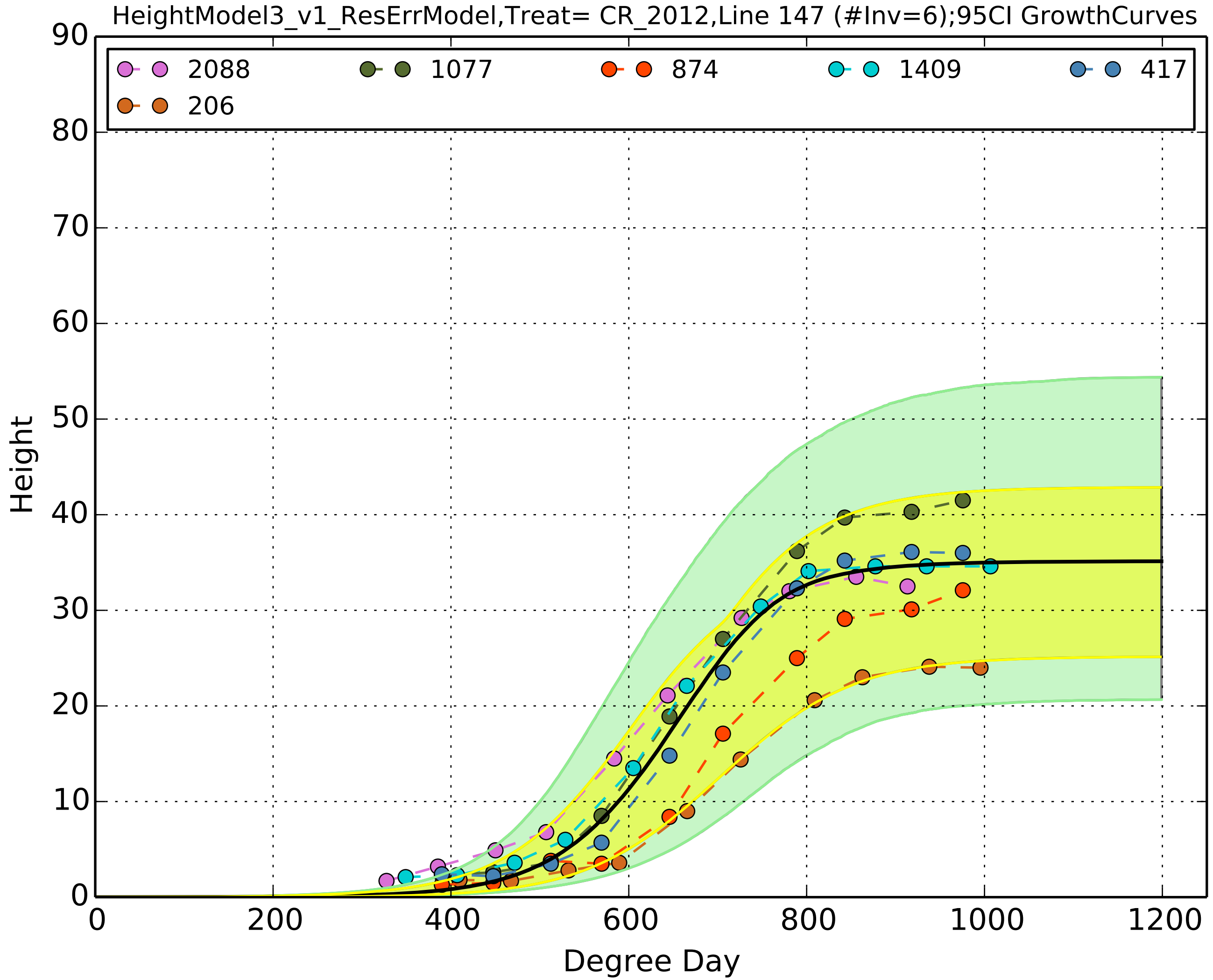

HeightModel3_v1_ResErrModel,Treat= CR_2012,Line 147 (#Inv=6);95CI GrowthCurves
2088
1077
874
1409
417
206
Height
Degree Day
0
10
20
30
40
50
60
70
80
90
0
200
400
600
800
1000
1200

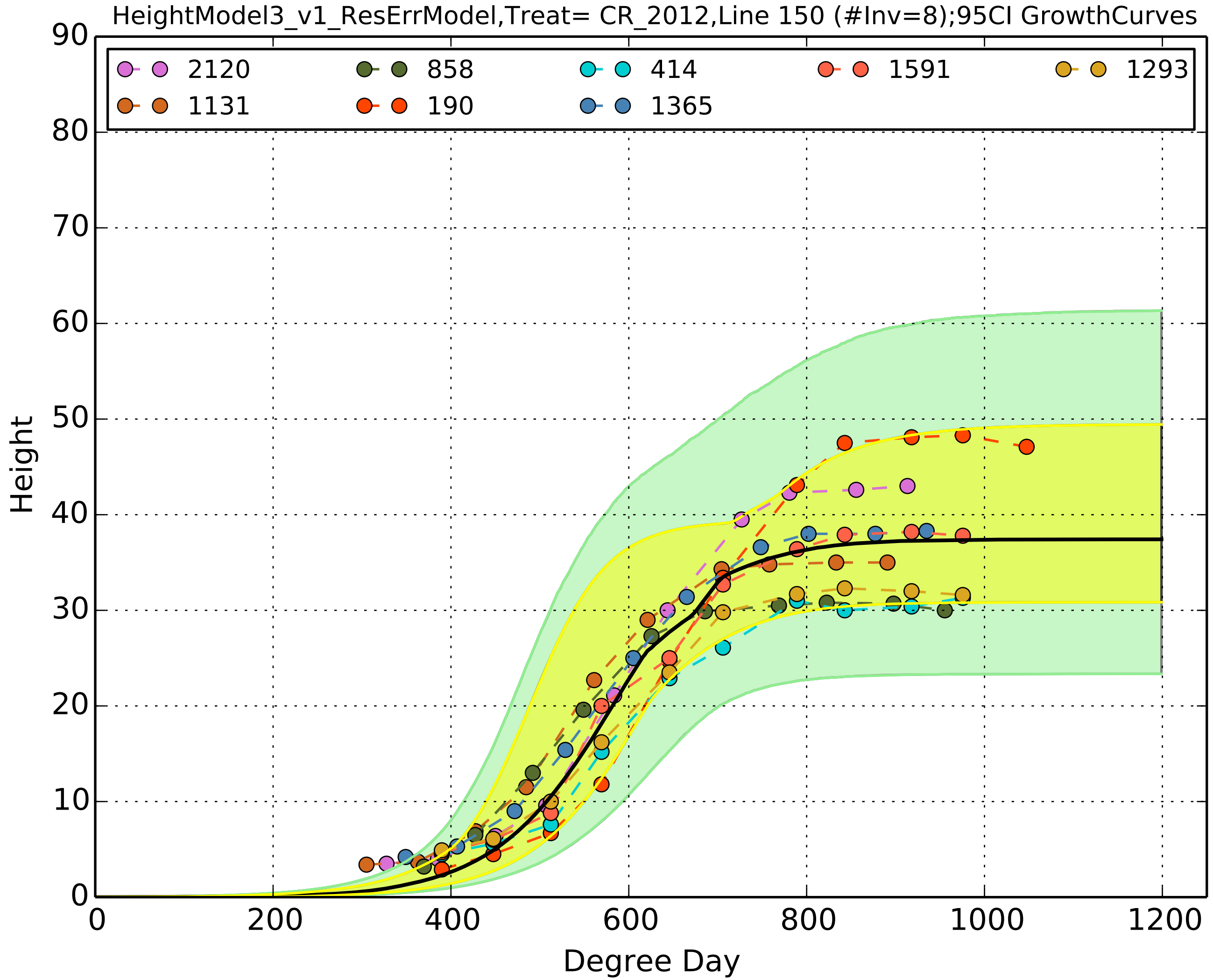
HeightModel3_v1_ResErrModel,Treat= CR_2012,Line 150 (#Inv=8);95CI GrowthCurves
2120
858
414
1591
1293
1131
190
1365
Height
Degree Day
0
10
20
30
40
50
60
70
80
90
0
200
400
600
800
1000
1200

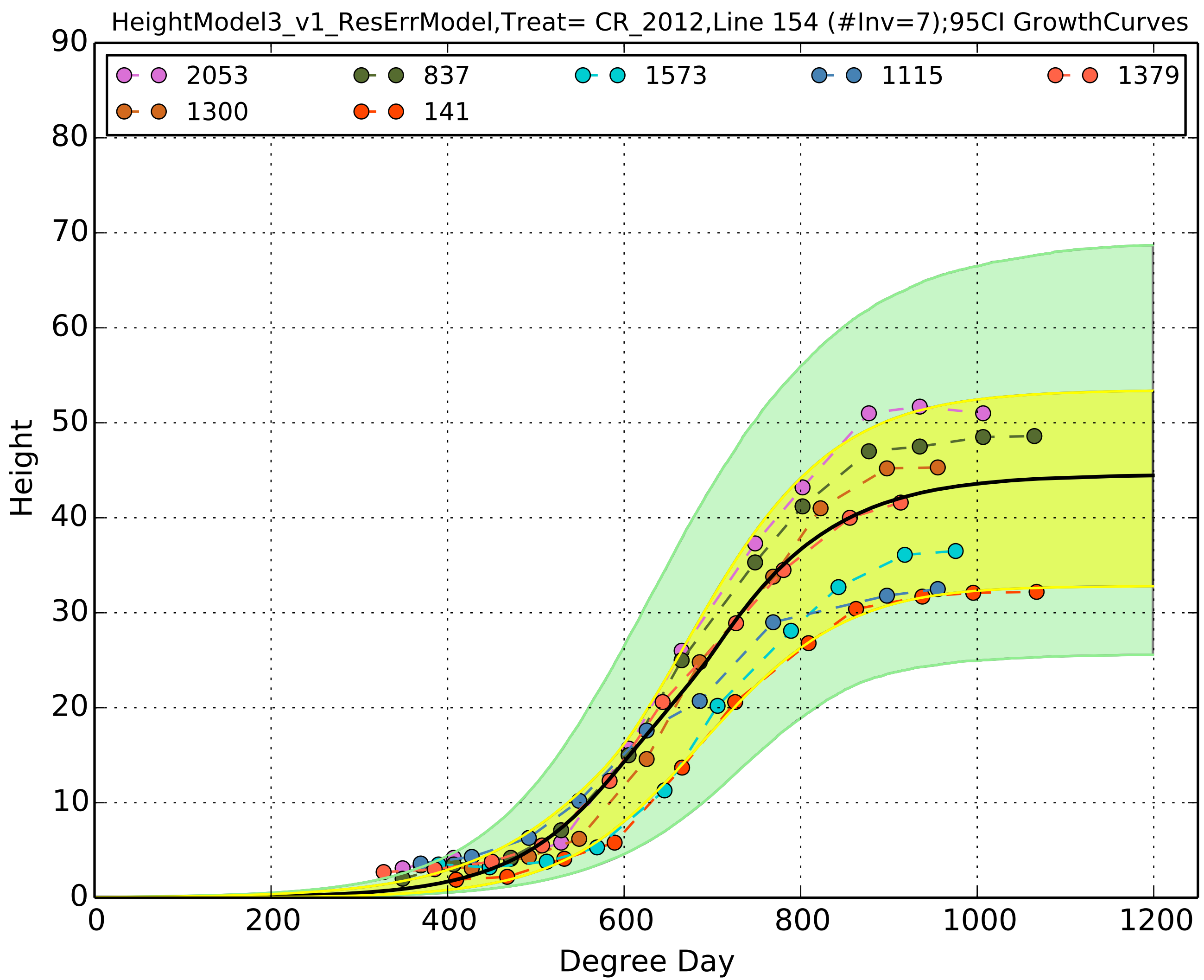
HeightModel3_v1_ResErrModel,Treat= CR_2012,Line 154 (#Inv=7);95CI GrowthCurves
2053
837
1573
1115
1379
1300
141
Height
Degree Day
0
10
20
30
40
50
60
70
80
90
0
200
400
600
800
1000
1200

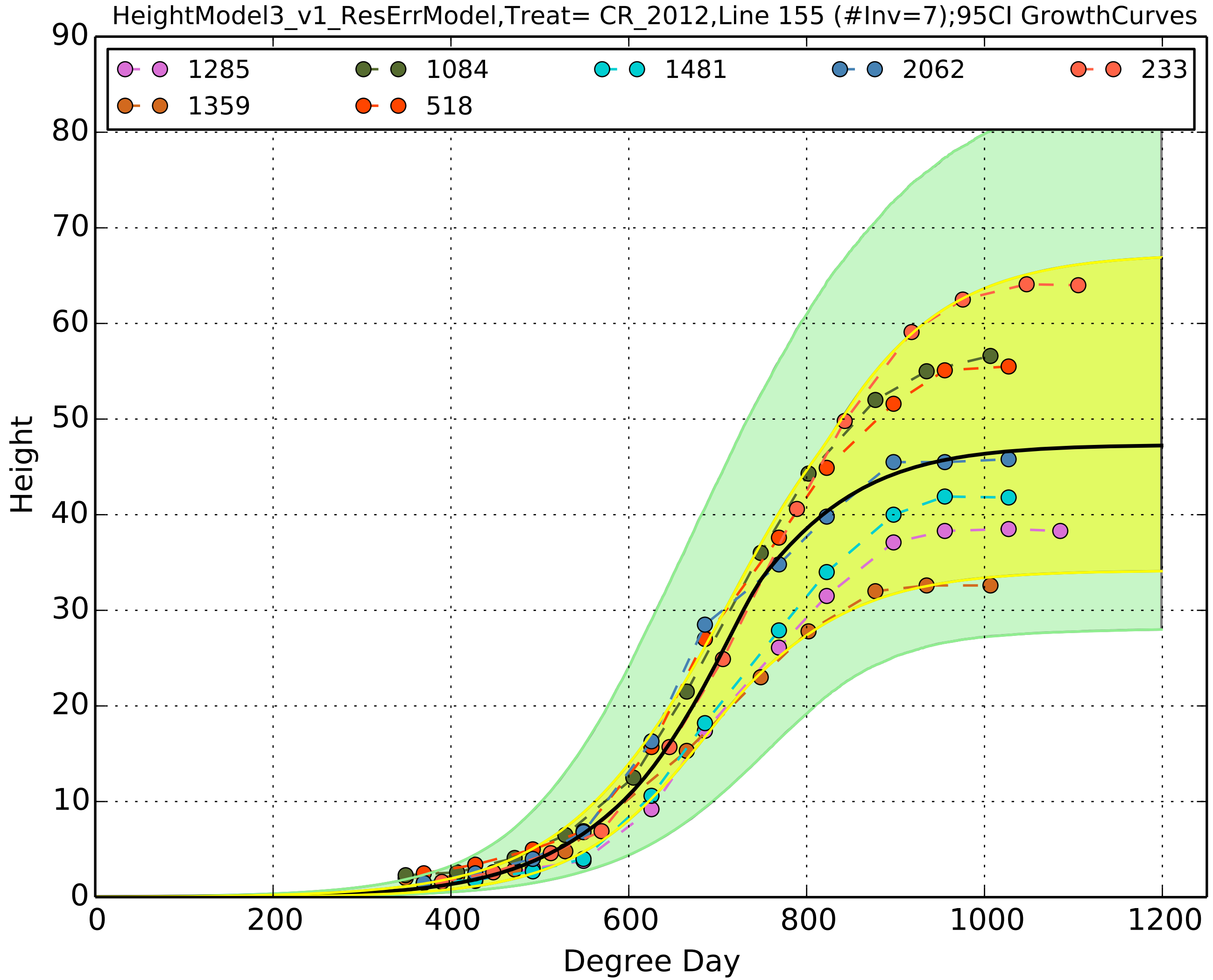

HeightModel3_v1_ResErrModel,Treat= CR_2012,Line 155 (#Inv=7);95CI GrowthCurves
1285
1084
1481
2062
233
1359
518
Height
Degree Day
0
10
20
30
40
50
60
70
80
90
0
200
400
600
800
1000
1200

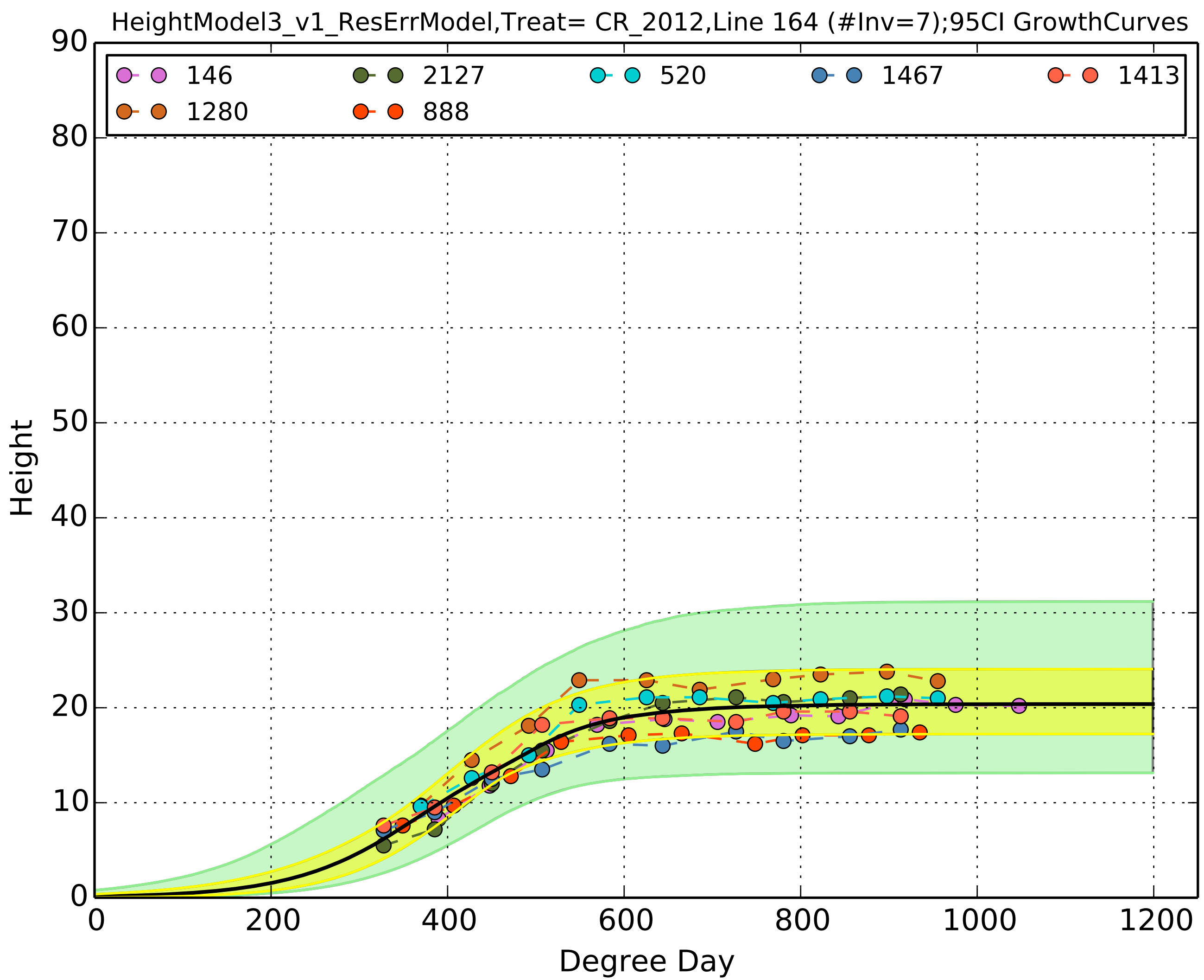

HeightModel3_v1_ResErrModel,Treat= CR_2012,Line 164 (#Inv=7);95CI GrowthCurves
146
2127
520
1467
1413
1280
888
Height
Degree Day
0
10
20
30
40
50
60
70
80
90
0
200
400
600
800
1000
1200

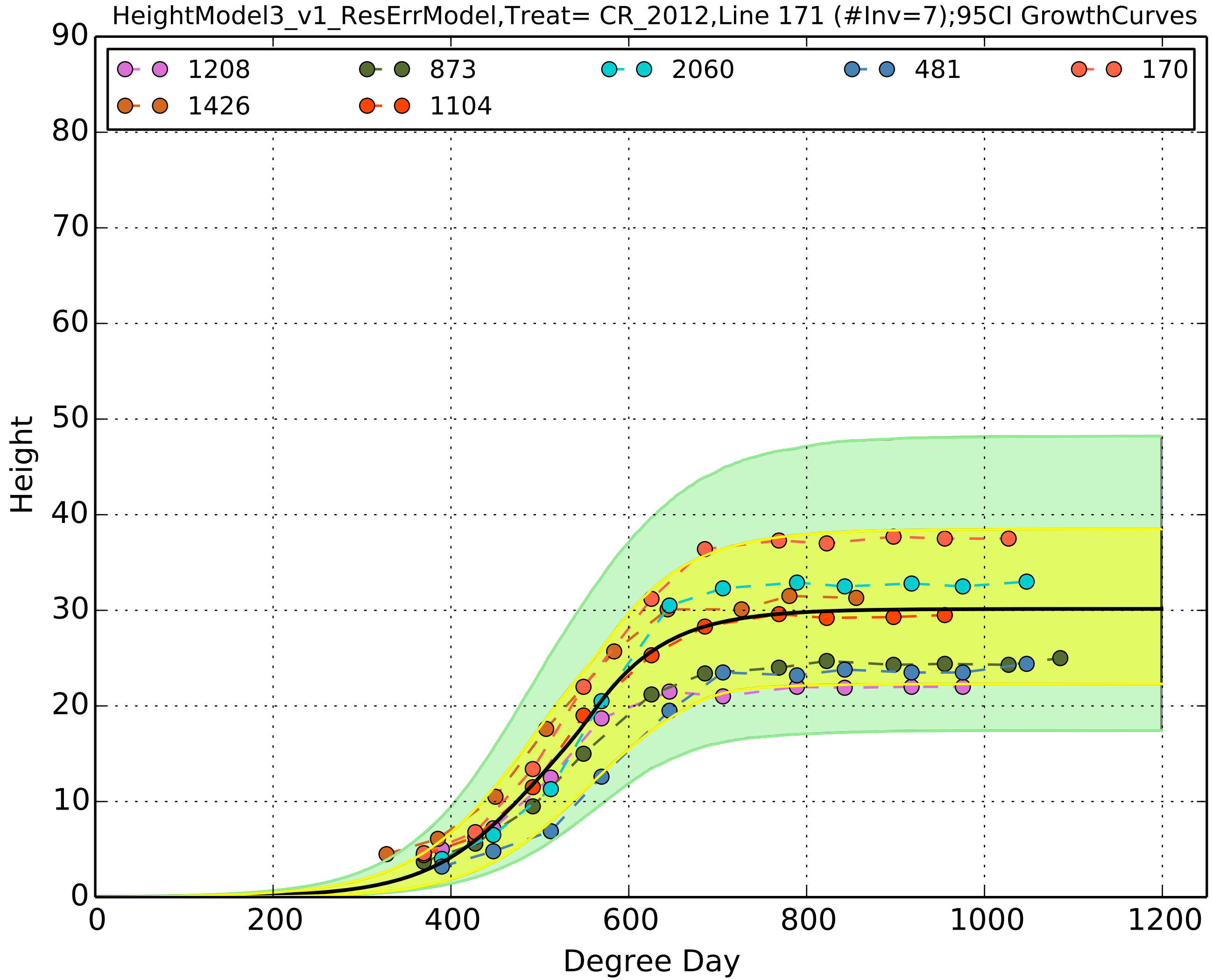

HeightModel3_v1_ResErrModel,Treat= CR_2012,Line 171 (#Inv=7);95CI GrowthCurves
1208
873
2060
481
170
1426
1104
Height
Degree Day
0
10
20
30
40
50
60
70
80
90
0
200
400
600
800
1000
1200

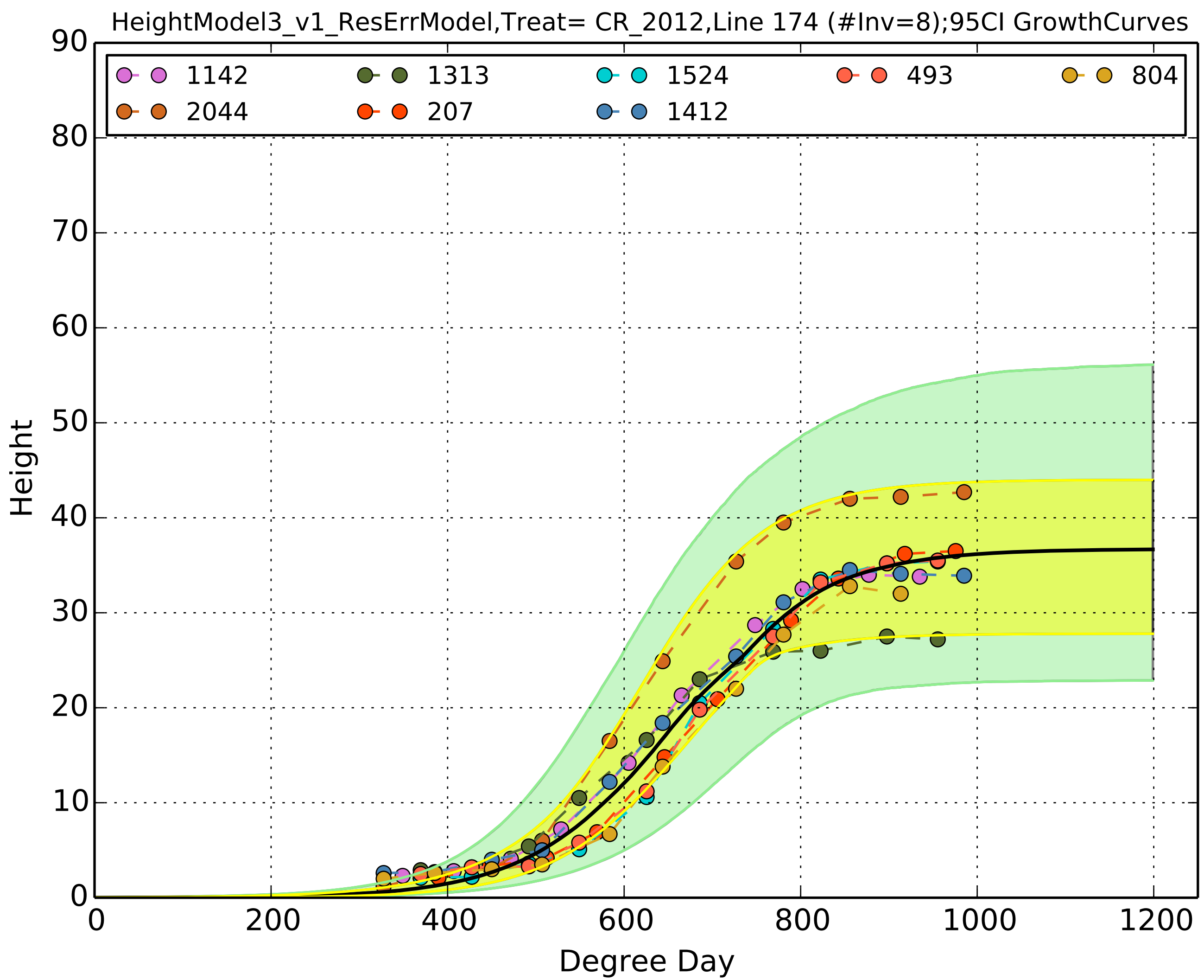

HeightModel3_v1_ResErrModel,Treat= CR_2012,Line 174 (#Inv=8);95CI GrowthCurves
1142
1313
1524
493
804
2044
207
1412
Height
Degree Day
0
10
20
30
40
50
60
70
80
90
0
200
400
600
800
1000
1200

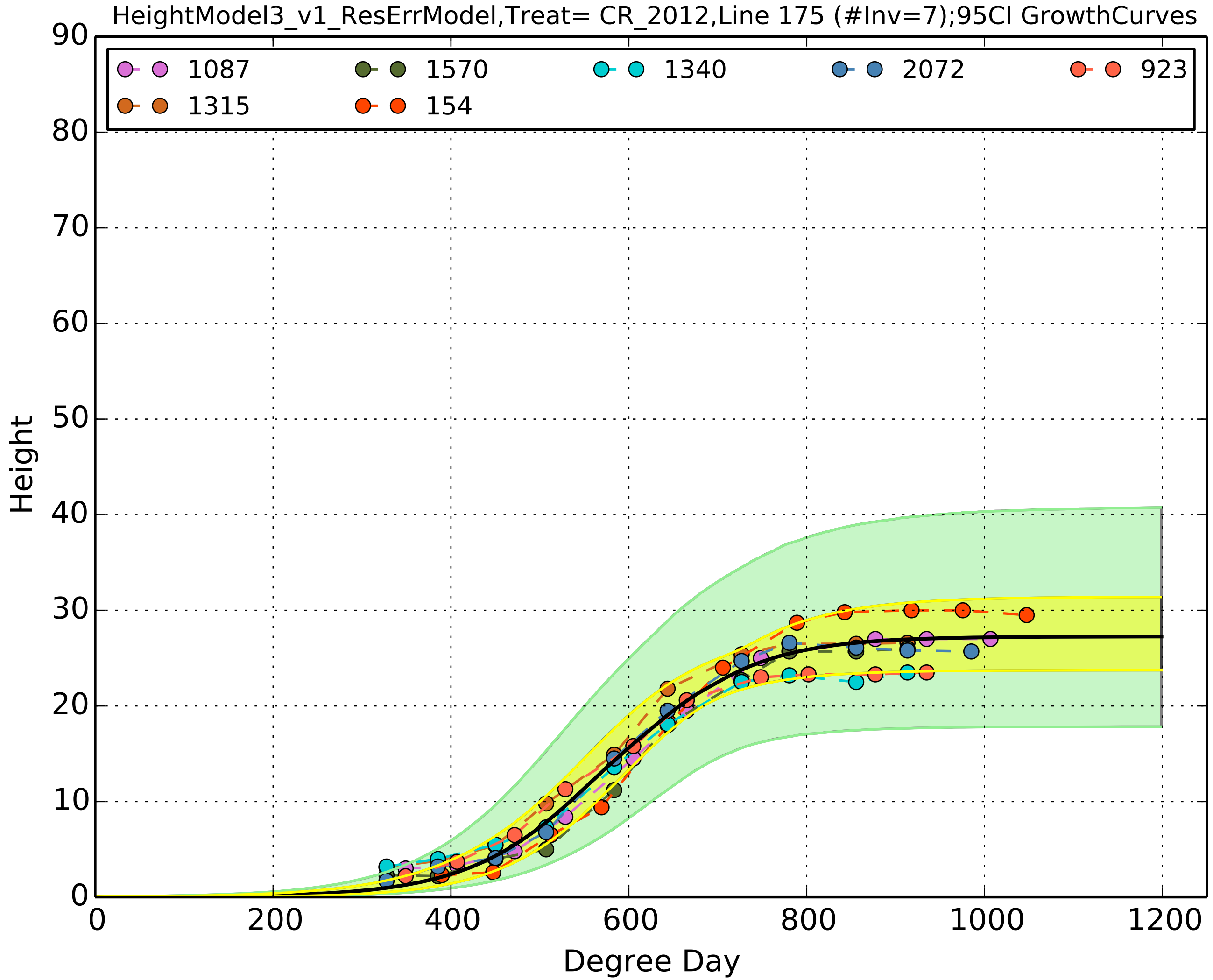
HeightModel3_v1_ResErrModel,Treat= CR_2012,Line 175 (#Inv=7);95CI GrowthCurves
1087
1570
1340
2072
923
1315
154
Height
Degree Day
0
10
20
30
40
50
60
70
80
90
0
200
400
600
800
1000
1200

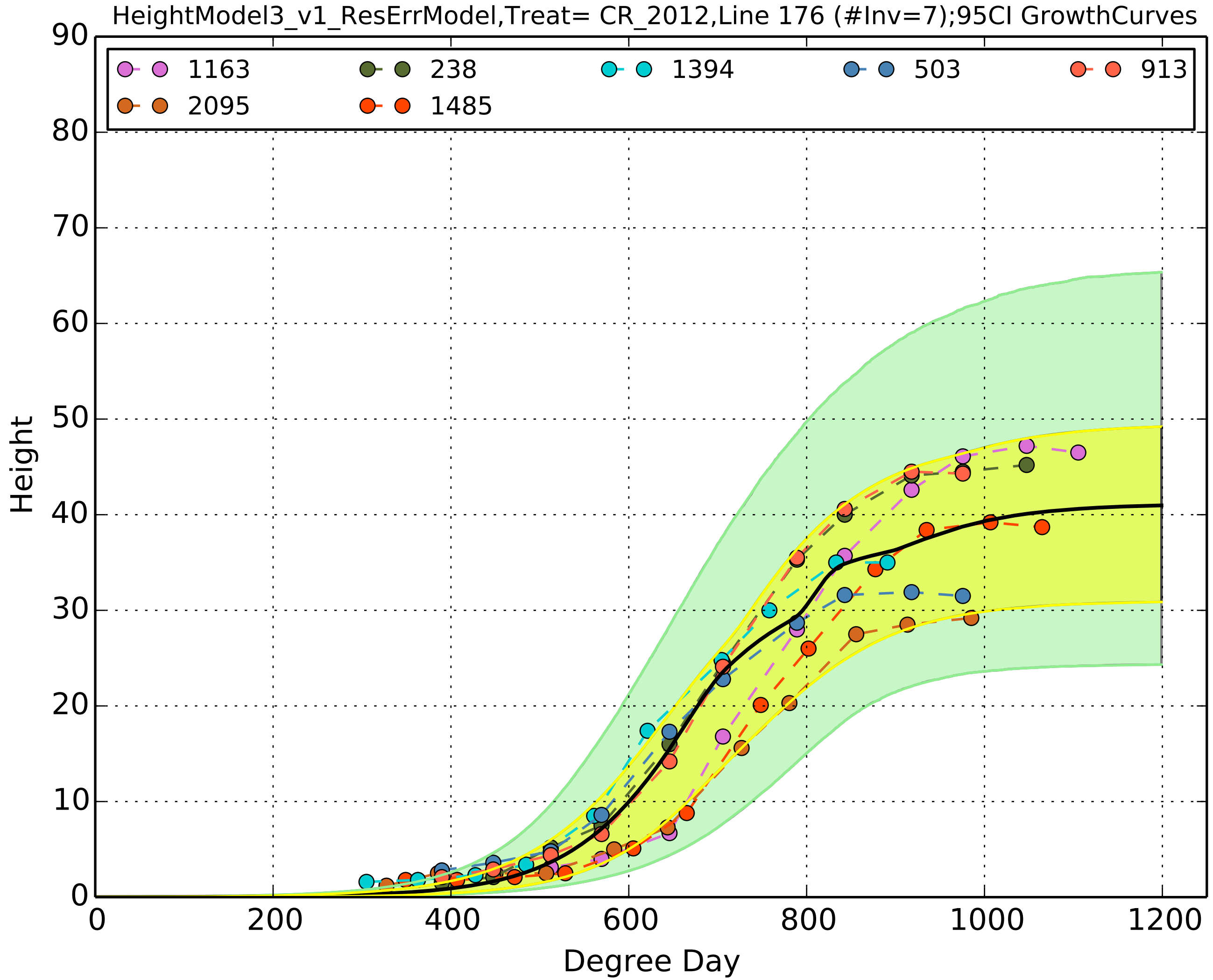

HeightModel3_v1_ResErrModel,Treat= CR_2012,Line 176 (#Inv=7);95CI GrowthCurves
1163
238
1394
503
913
2095
1485
Height
Degree Day
0
10
20
30
40
50
60
70
80
90
0
200
400
600
800
1000
1200

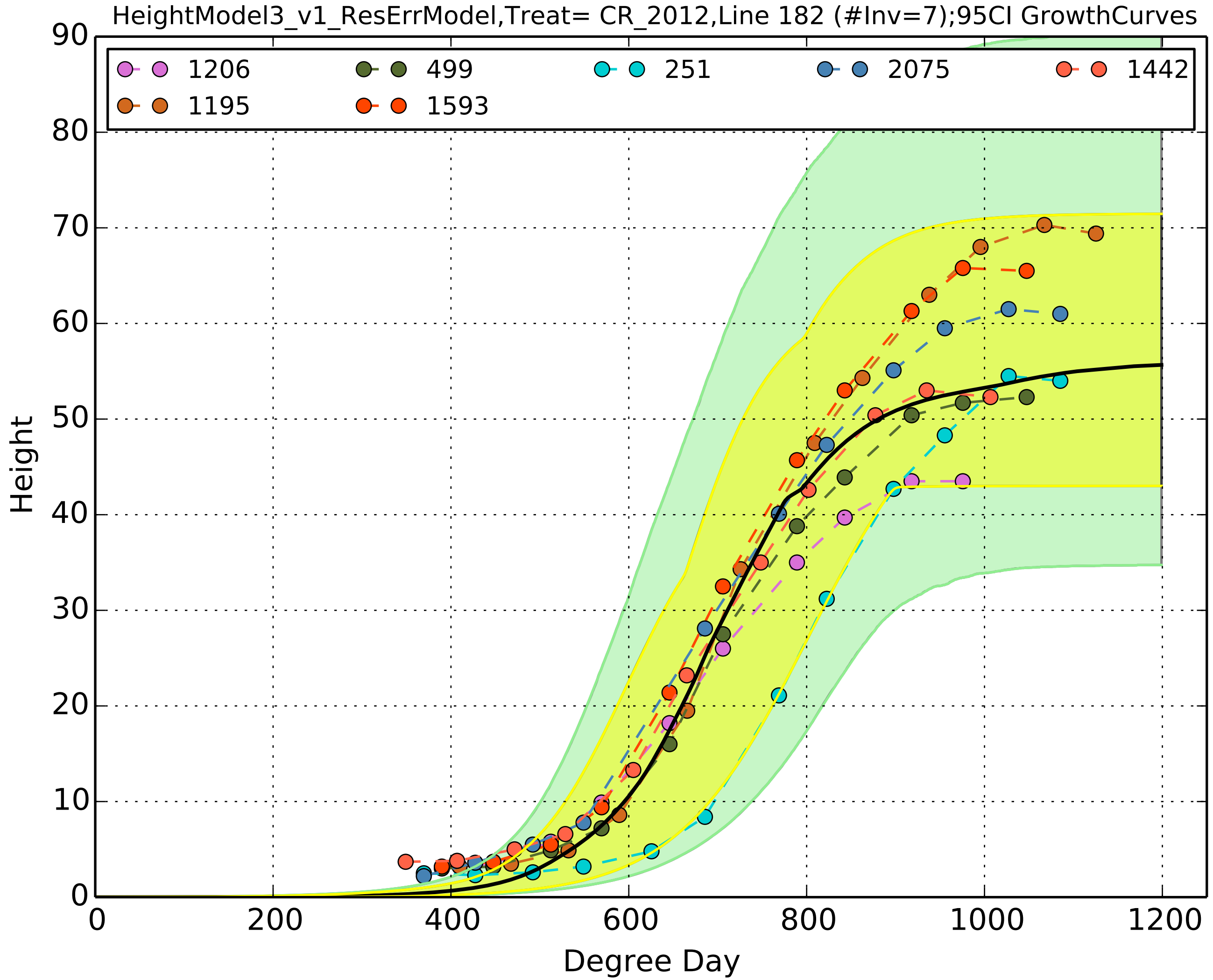

HeightModel3_v1_ResErrModel,Treat= CR_2012,Line 182 (#Inv=7);95CI GrowthCurves
1206
499
251
2075
1442
1195
1593
Height
Degree Day
0
10
20
30
40
50
60
70
80
90
0
200
400
600
800
1000
1200

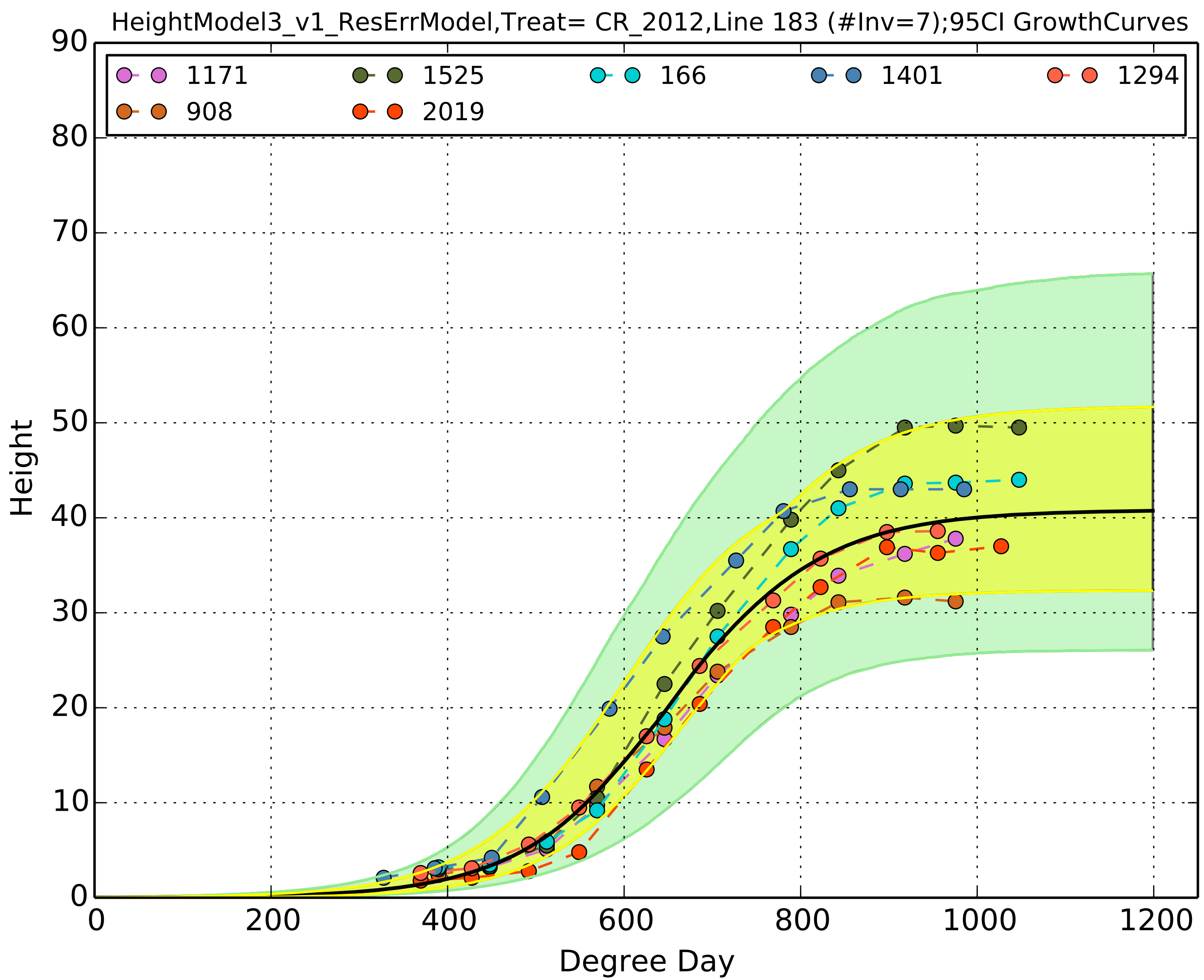

HeightModel3_v1_ResErrModel,Treat= CR_2012,Line 183 (#Inv=7);95CI GrowthCurves
1171
1525
166
1401
1294
908
2019
Height
Degree Day
0
10
20
30
40
50
60
70
80
90
0
200
400
600
800
1000
1200

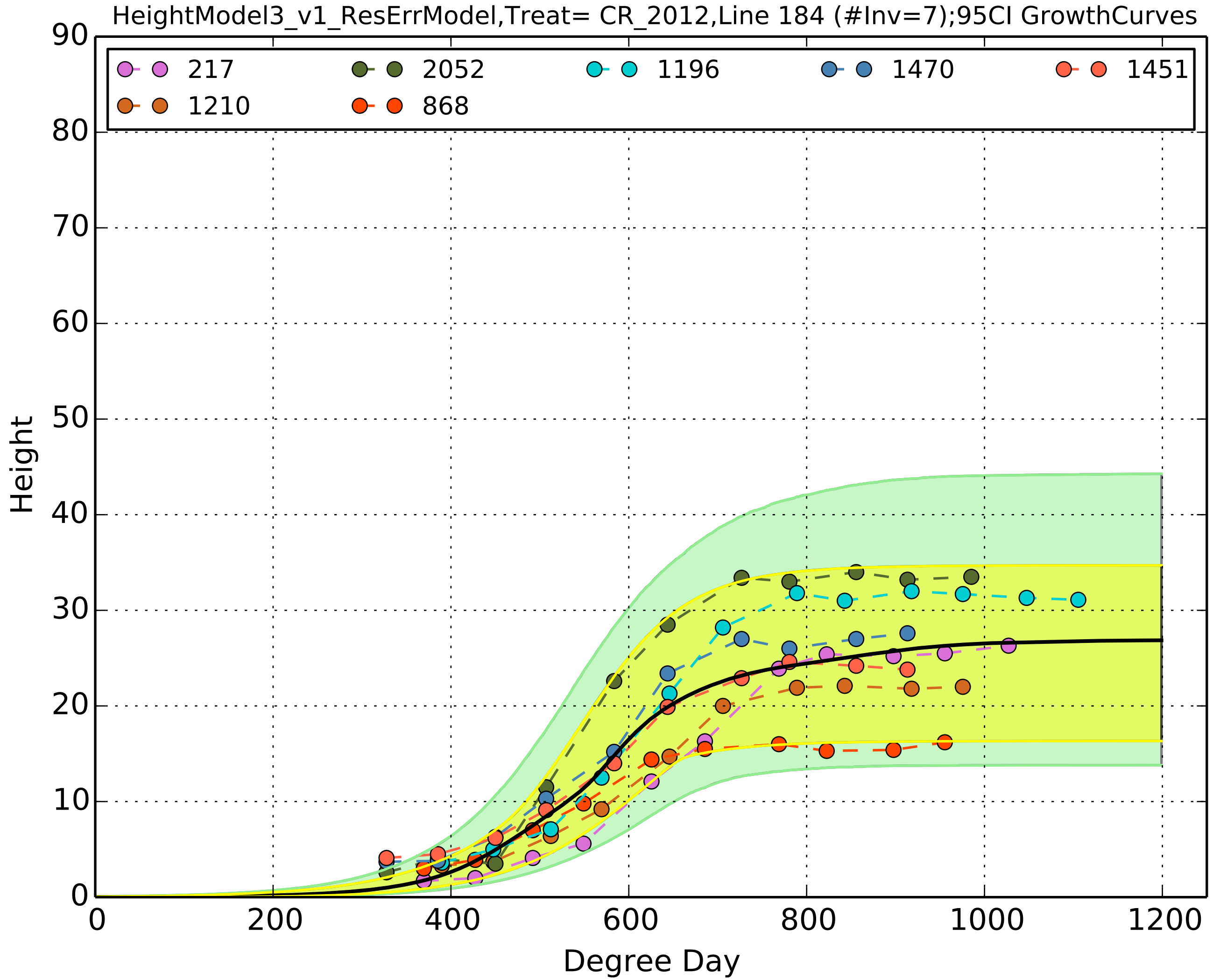

HeightModel3_v1_ResErrModel,Treat= CR_2012,Line 184 (#Inv=7);95CI GrowthCurves
217
2052
1196
1470
1451
1210
868
Height
Degree Day
0
10
20
30
40
50
60
70
80
90
0
200
400
600
800
1000
1200

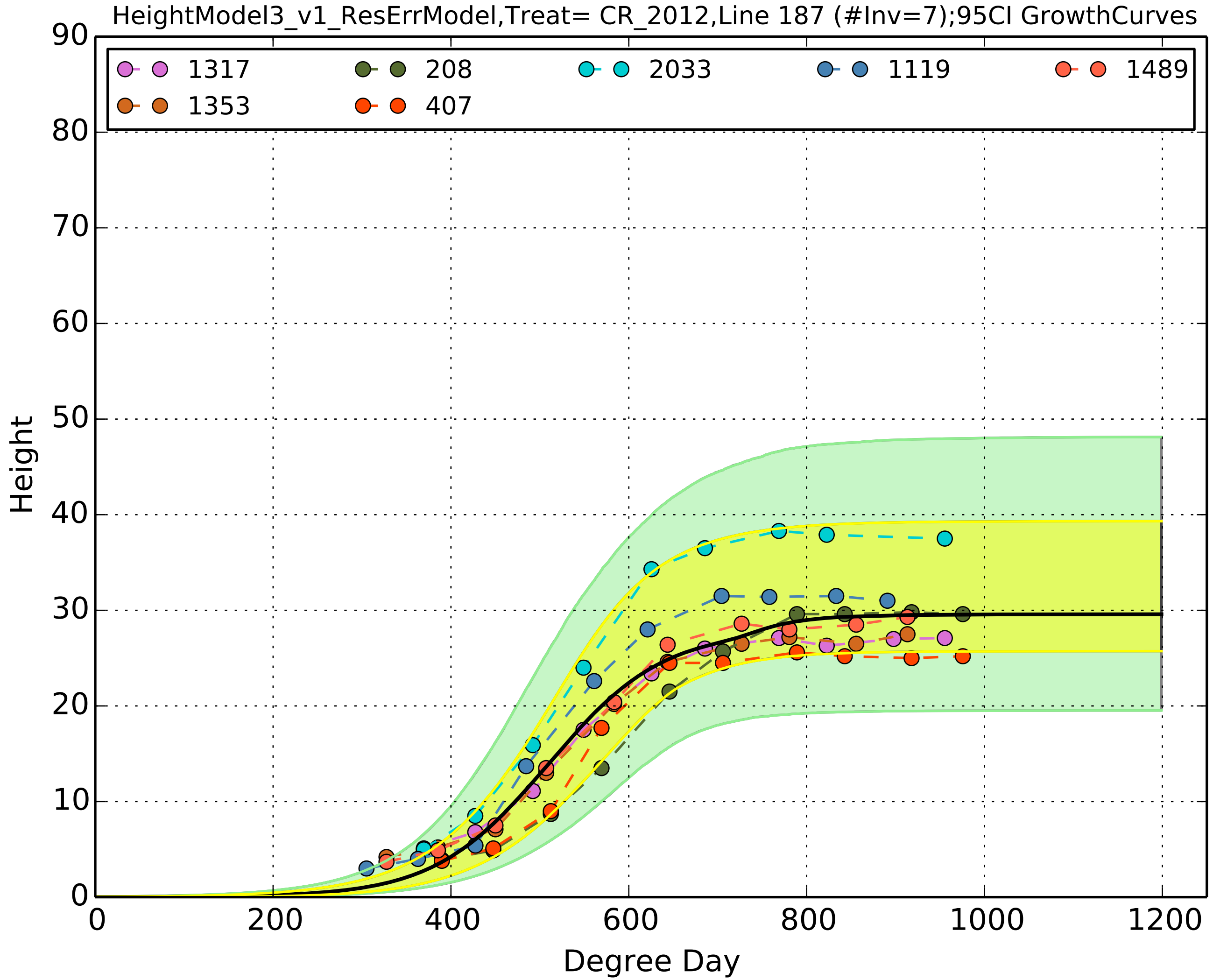

HeightModel3_v1_ResErrModel,Treat= CR_2012,Line 187 (#Inv=7);95CI GrowthCurves
1317
208
2033
1119
1489
1353
407
Height
Degree Day
0
10
20
30
40
50
60
70
80
90
0
200
400
600
800
1000
1200

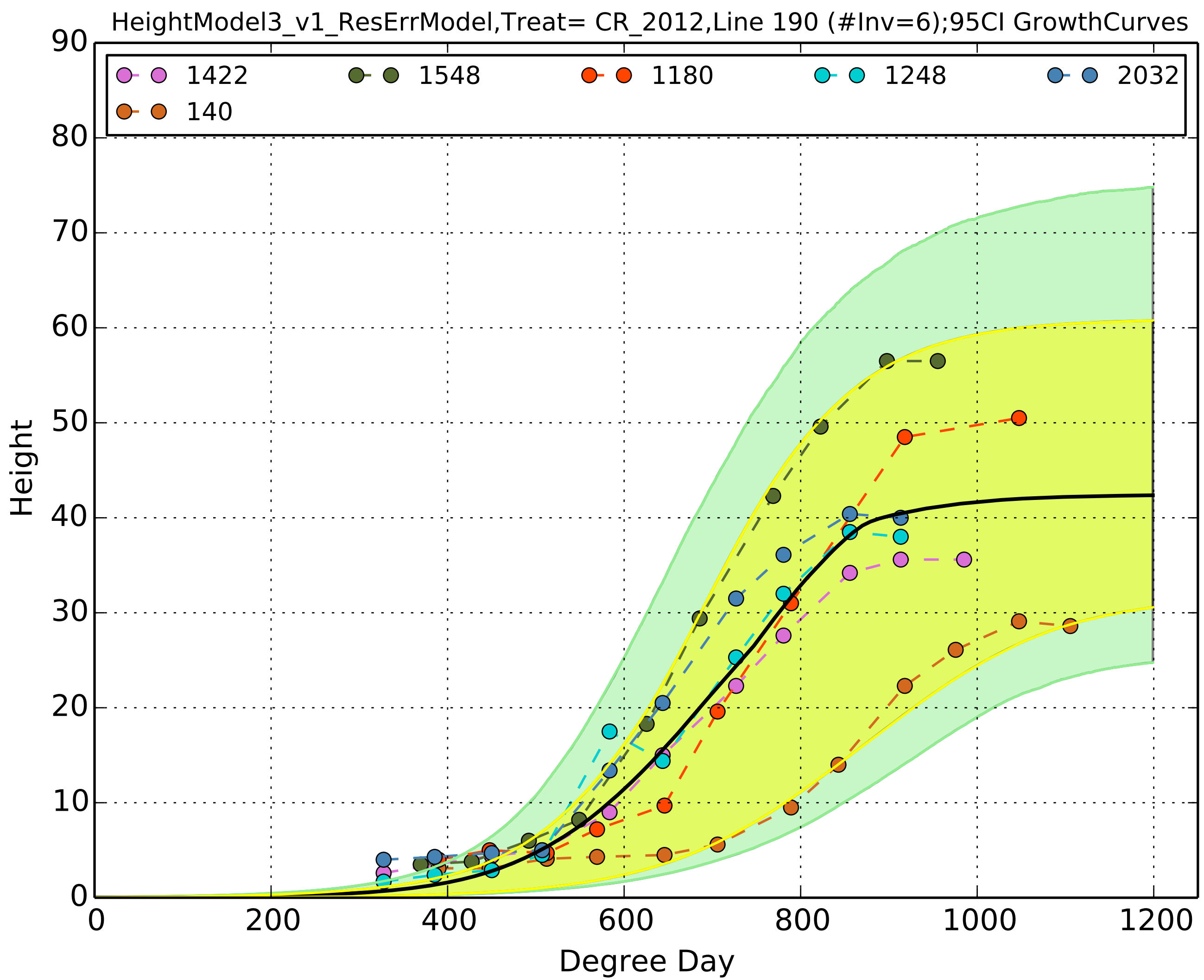
HeightModel3_v1_ResErrModel,Treat= CR_2012,Line 190 (#Inv=6);95CI GrowthCurves
1422
1548
1180
1248
2032
140
Height
Degree Day
0
10
20
30
40
50
60
70
80
90
0
200
400
600
800
1000
1200

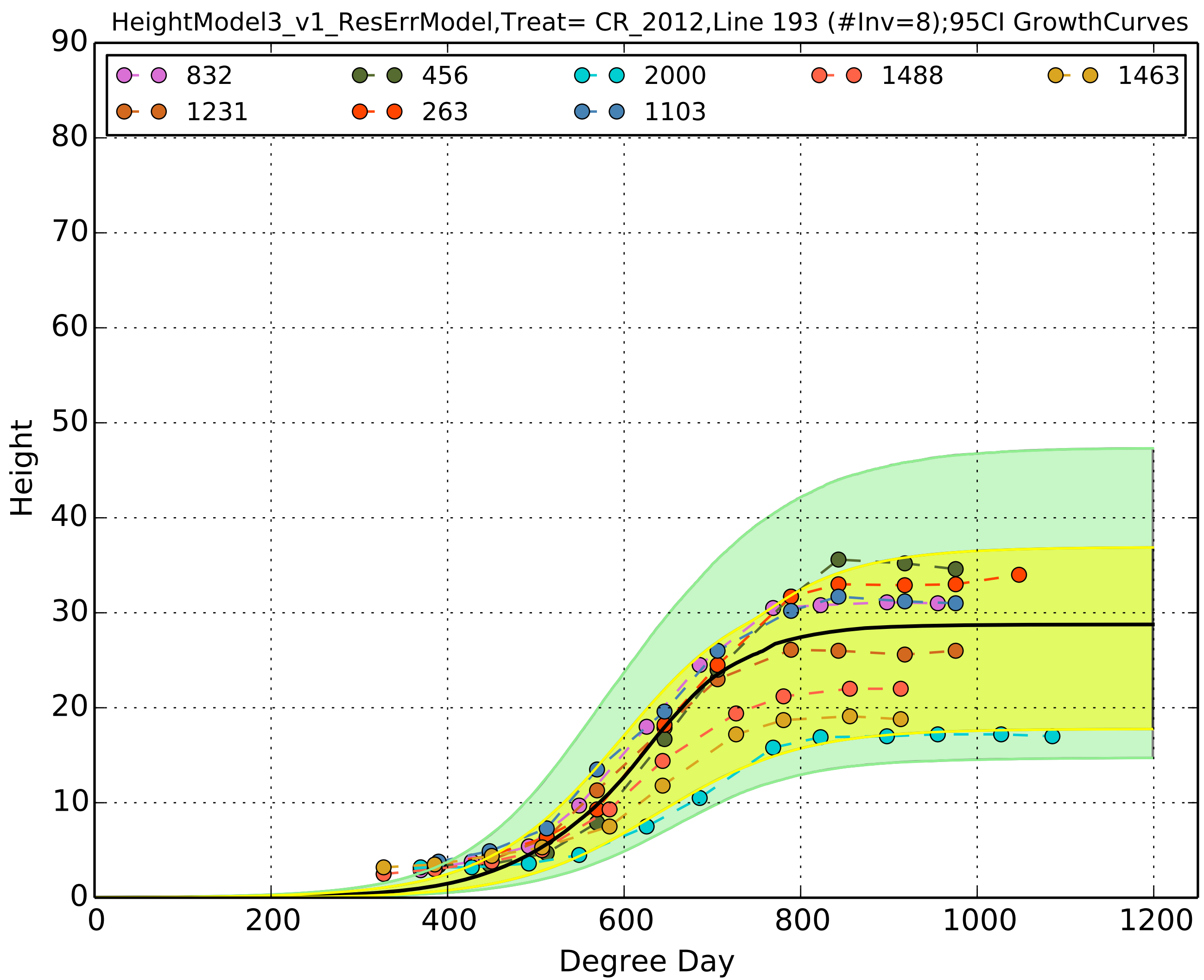
HeightModel3_v1_ResErrModel,Treat= CR_2012,Line 193 (#Inv=8);95CI GrowthCurves
832
456
2000
1488
1463
1231
263
1103
Height
Degree Day
0
10
20
30
40
50
60
70
80
90
0
200
400
600
800
1000
1200

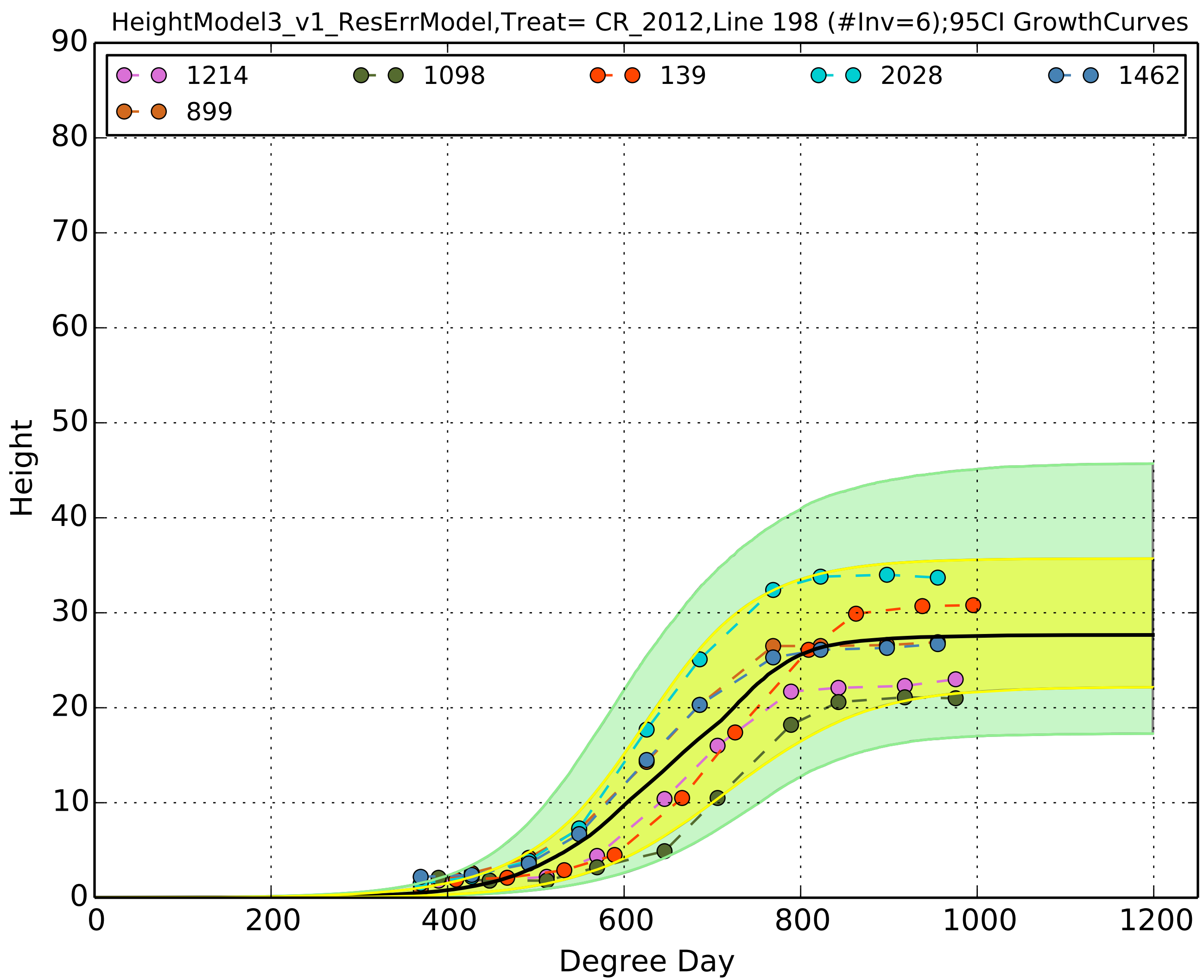

HeightModel3_v1_ResErrModel,Treat= CR_2012,Line 198 (#Inv=6);95CI GrowthCurves
1214
1098
139
2028
1462
899
Height
Degree Day
0
10
20
30
40
50
60
70
80
90
0
200
400
600
800
1000
1200

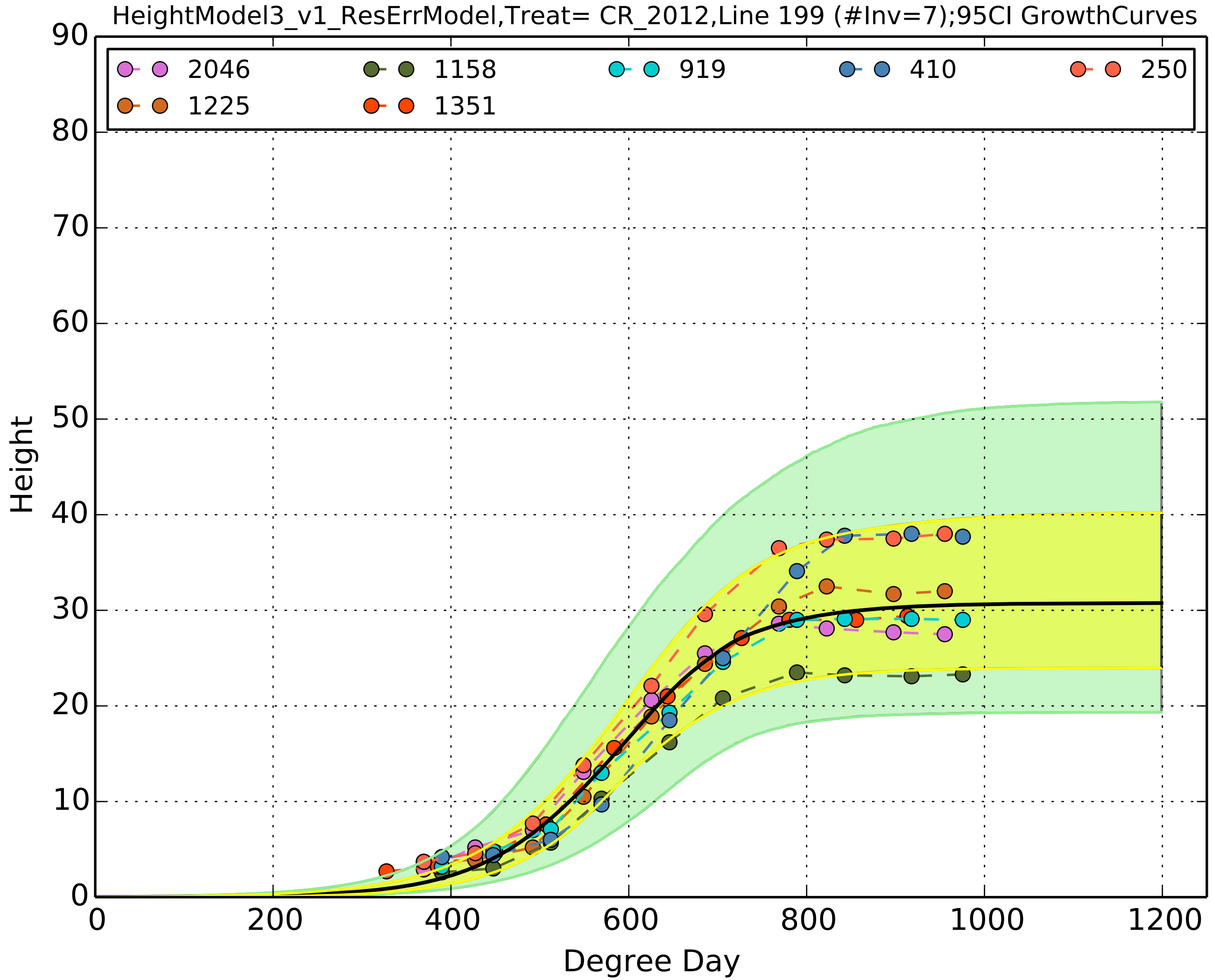

HeightModel3_v1_ResErrModel,Treat= CR_2012,Line 199 (#Inv=7);95CI GrowthCurves
2046
1158
919
410
250
1225
1351
Height
Degree Day
0
10
20
30
40
50
60
70
80
90
0
200
400
600
800
1000
1200

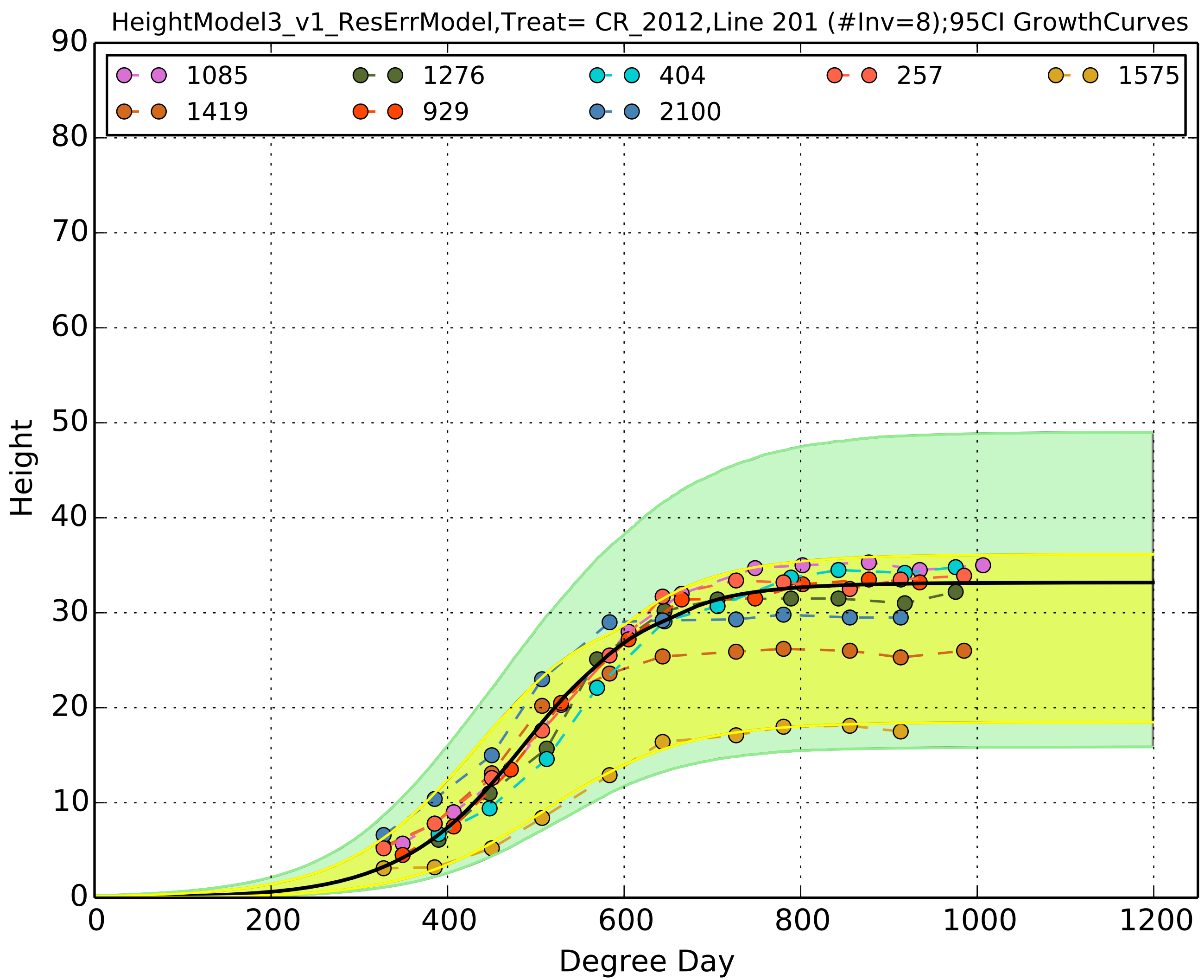

HeightModel3_v1_ResErrModel,Treat= CR_2012,Line 201 (#Inv=8);95CI GrowthCurves
1085
1276
404
257
1575
1419
929
2100
Height
Degree Day
0
10
20
30
40
50
60
70
80
90
0
200
400
600
800
1000
1200

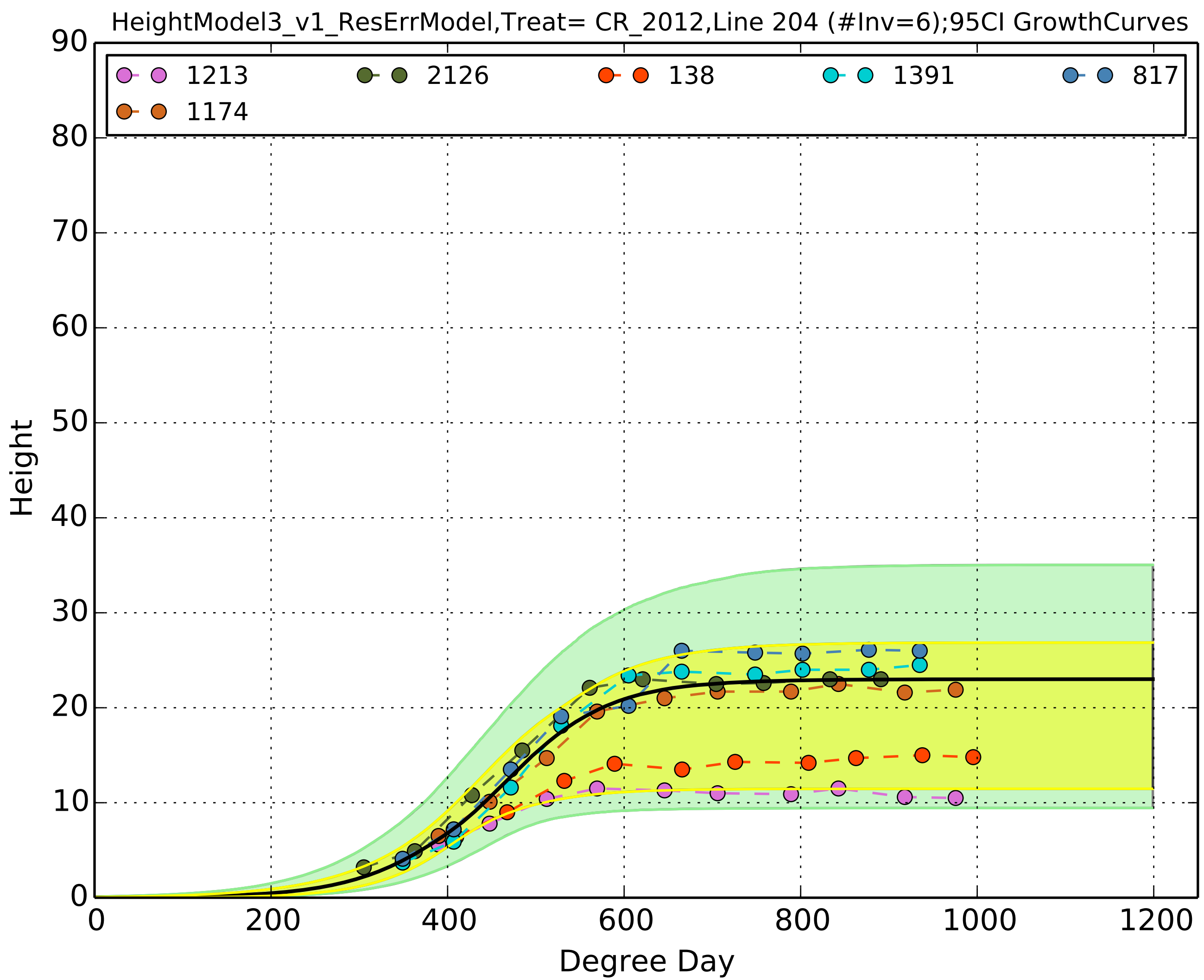

HeightModel3_v1_ResErrModel,Treat= CR_2012,Line 204 (#Inv=6);95CI GrowthCurves
1213
2126
138
1391
817
1174
Height
Degree Day
0
10
20
30
40
50
60
70
80
90
0
200
400
600
800
1000
1200

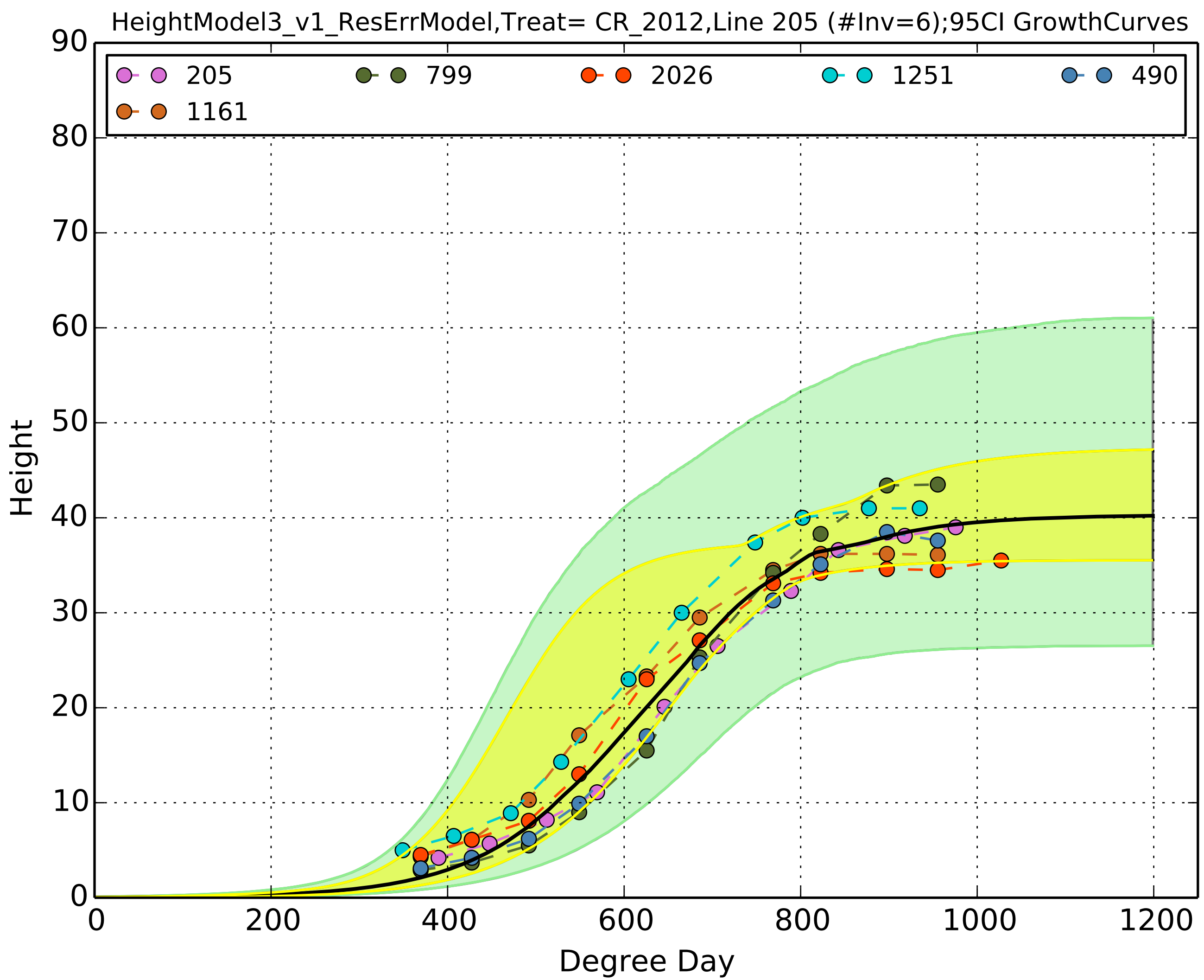

HeightModel3_v1_ResErrModel,Treat= CR_2012,Line 205 (#Inv=6);95CI GrowthCurves
205
799
2026
1251
490
1161
Height
Degree Day
0
10
20
30
40
50
60
70
80
90
0
200
400
600
800
1000
1200

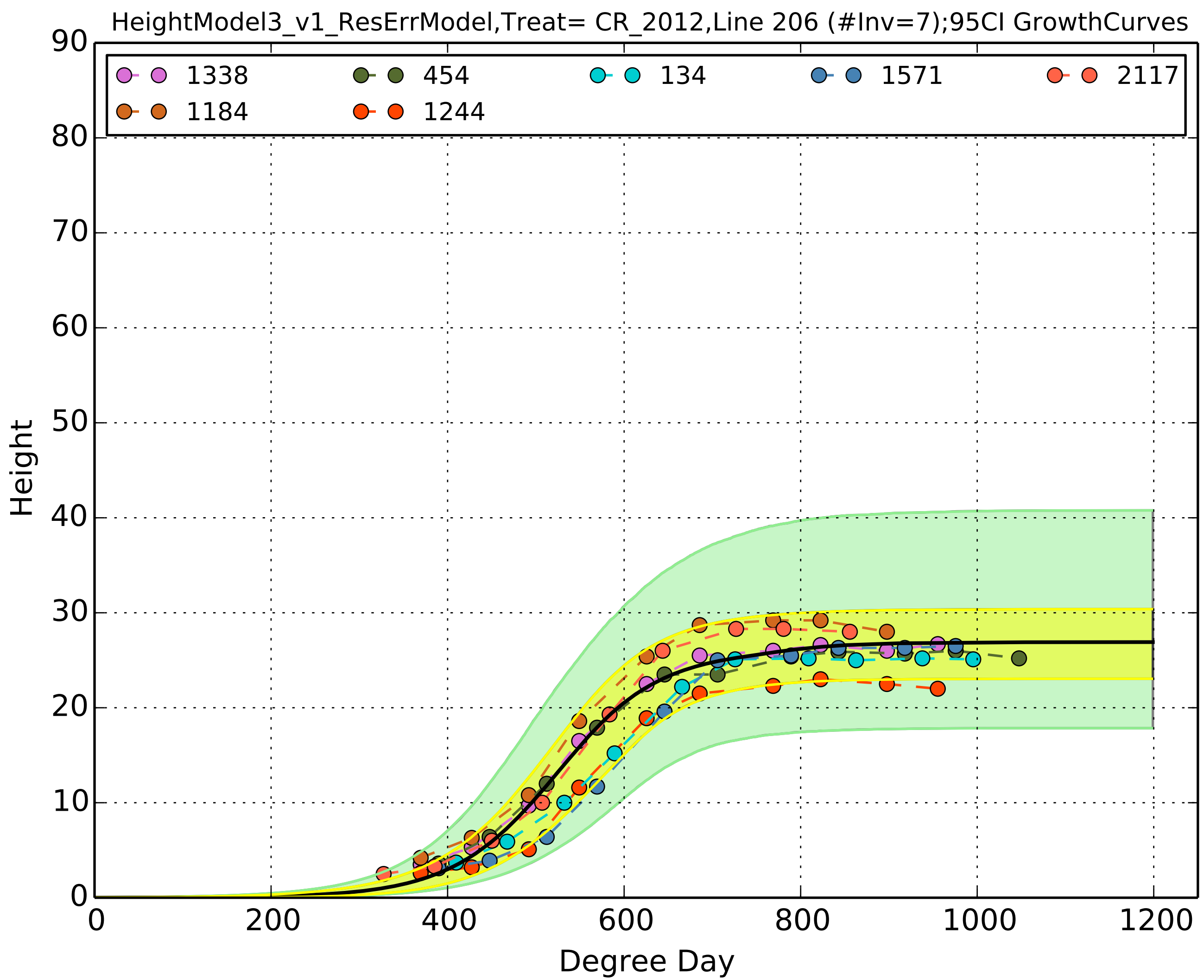

HeightModel3_v1_ResErrModel,Treat= CR_2012,Line 206 (#Inv=7);95CI GrowthCurves
1338
454
134
1571
2117
1184
1244
Height
Degree Day
0
10
20
30
40
50
60
70
80
90
0
200
400
600
800
1000
1200

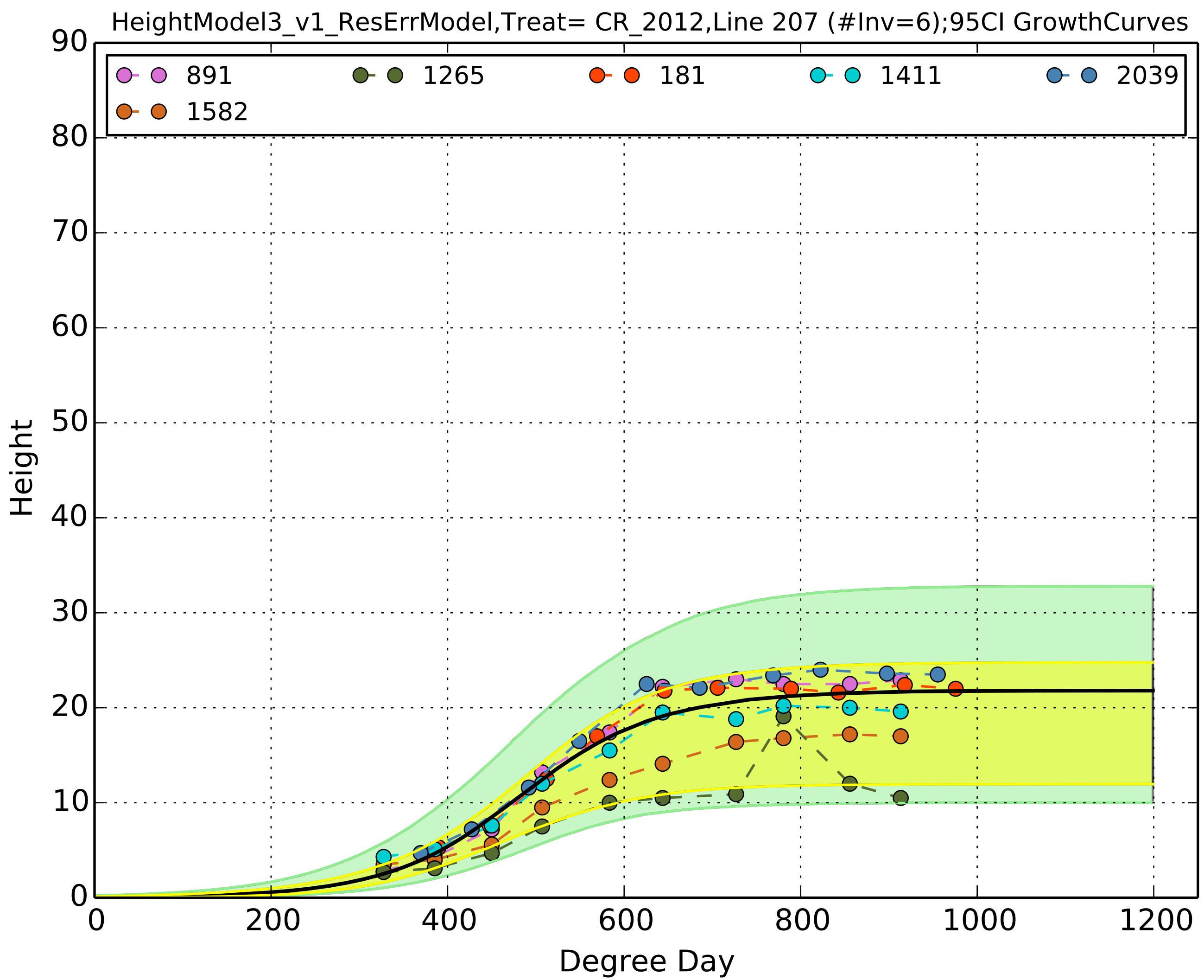
HeightModel3_v1_ResErrModel,Treat= CR_2012,Line 207 (#Inv=6);95CI GrowthCurves
891
1265
181
1411
2039
1582
Height
Degree Day
0
10
20
30
40
50
60
70
80
90
0
200
400
600
800
1000
1200

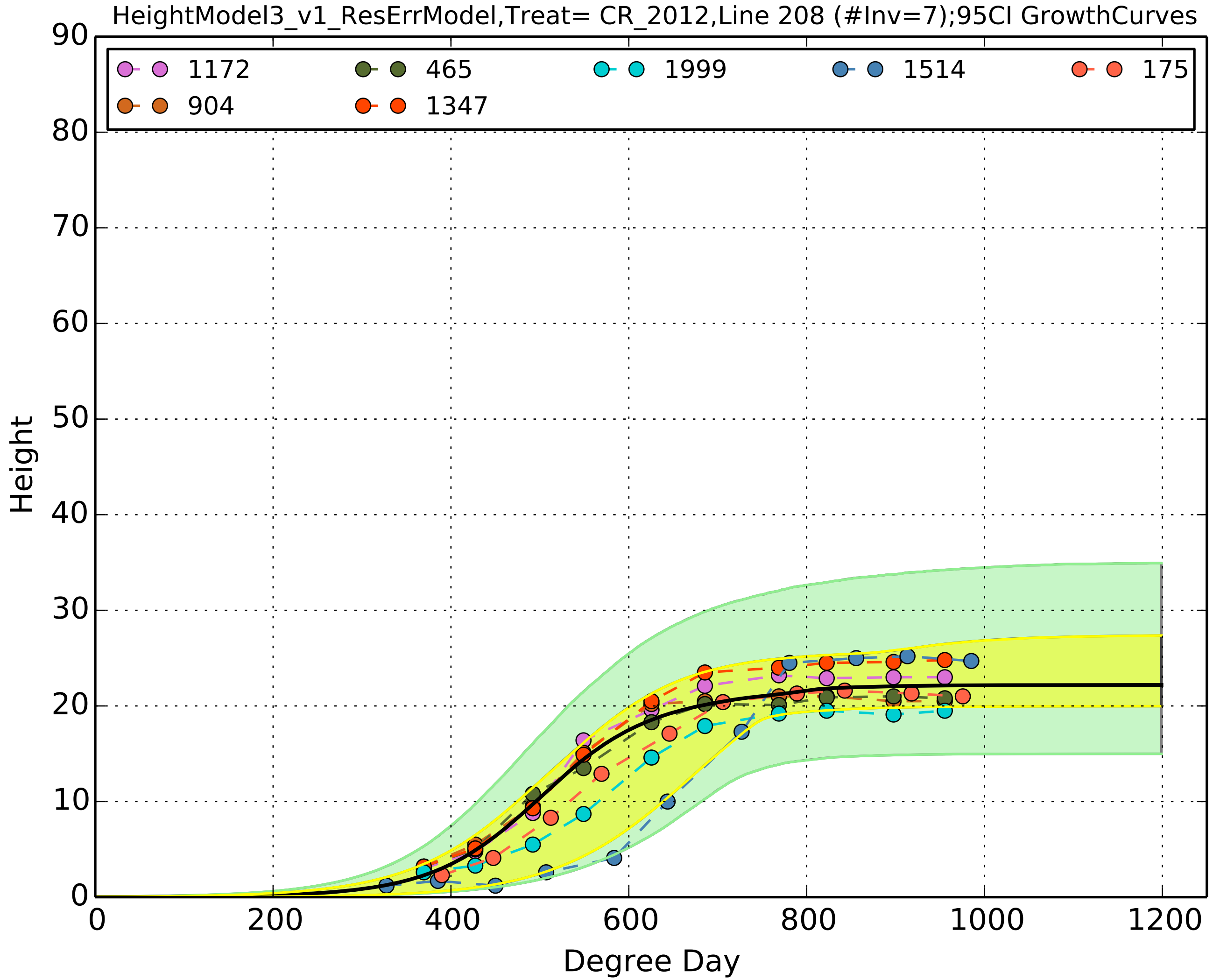

HeightModel3_v1_ResErrModel,Treat= CR_2012,Line 208 (#Inv=7);95CI GrowthCurves
1172
465
1999
1514
175
904
1347
Height
Degree Day
0
10
20
30
40
50
60
70
80
90
0
200
400
600
800
1000
1200

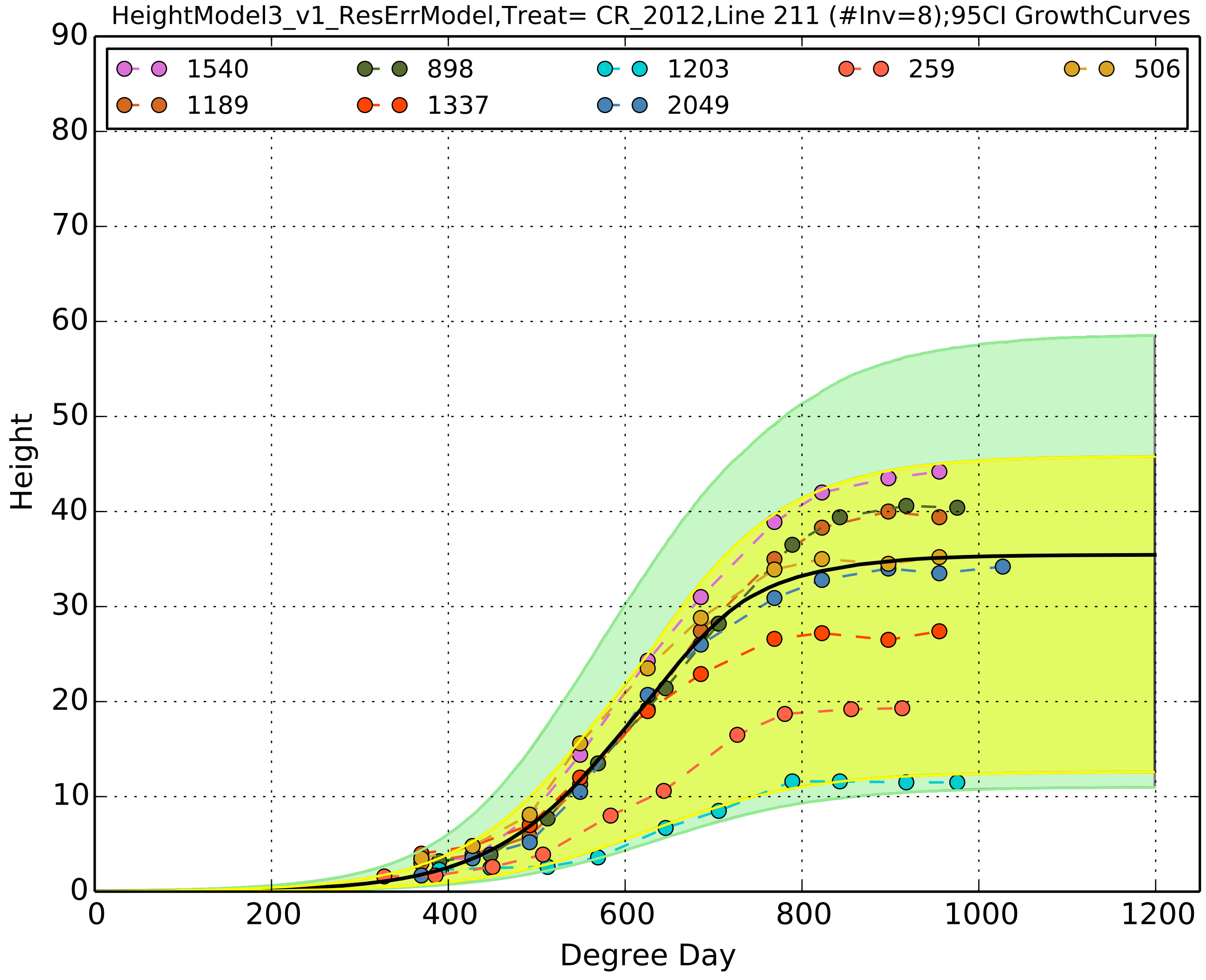
HeightModel3_v1_ResErrModel,Treat= CR_2012,Line 211 (#Inv=8);95CI GrowthCurves
1540
898
1203
259
506
1189
1337
2049
Height
Degree Day
0
10
20
30
40
50
60
70
80
90
0
200
400
600
800
1000
1200

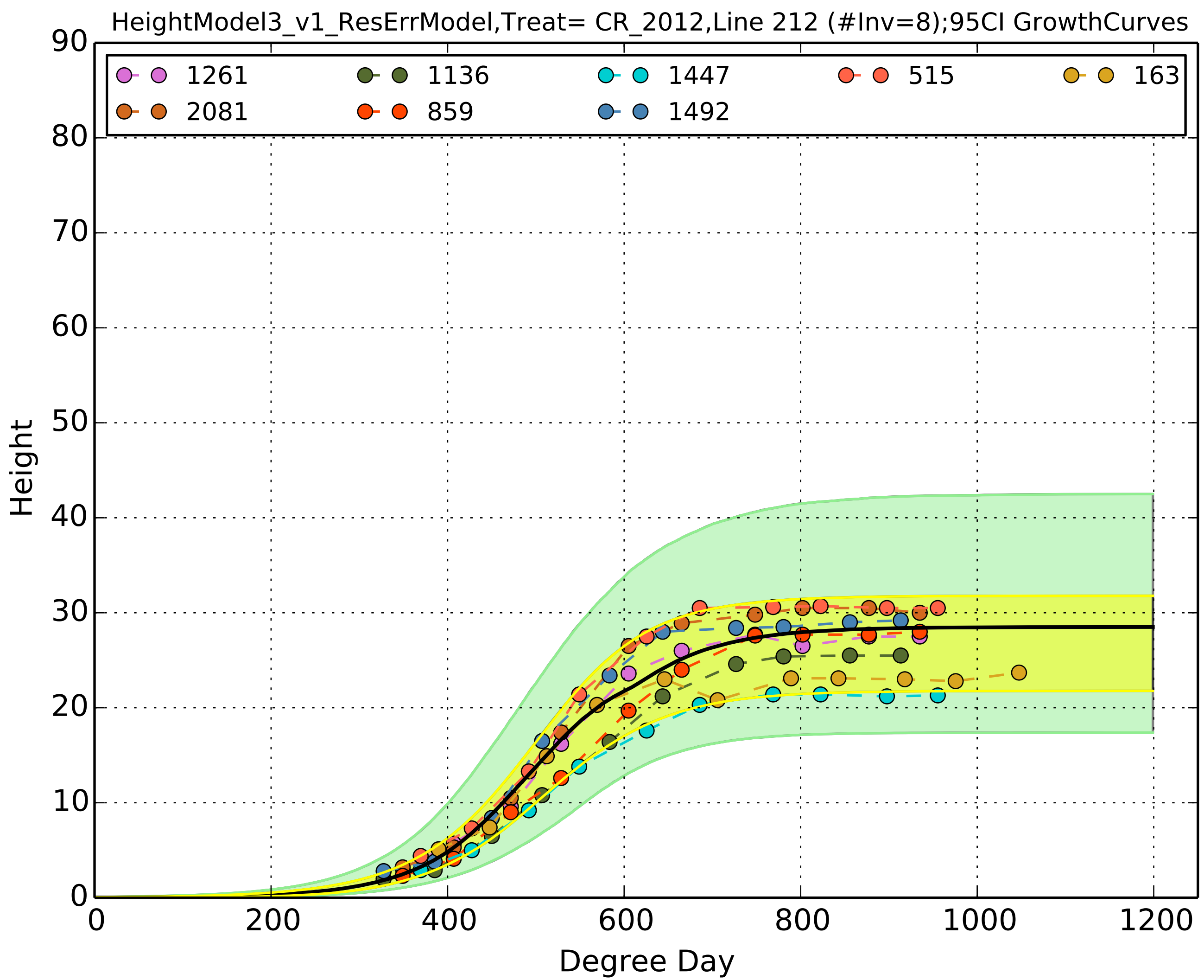

HeightModel3_v1_ResErrModel,Treat= CR_2012,Line 212 (#Inv=8);95CI GrowthCurves
1261
1136
1447
515
163
2081
859
1492
Height
Degree Day
0
10
20
30
40
50
60
70
80
90
0
200
400
600
800
1000
1200

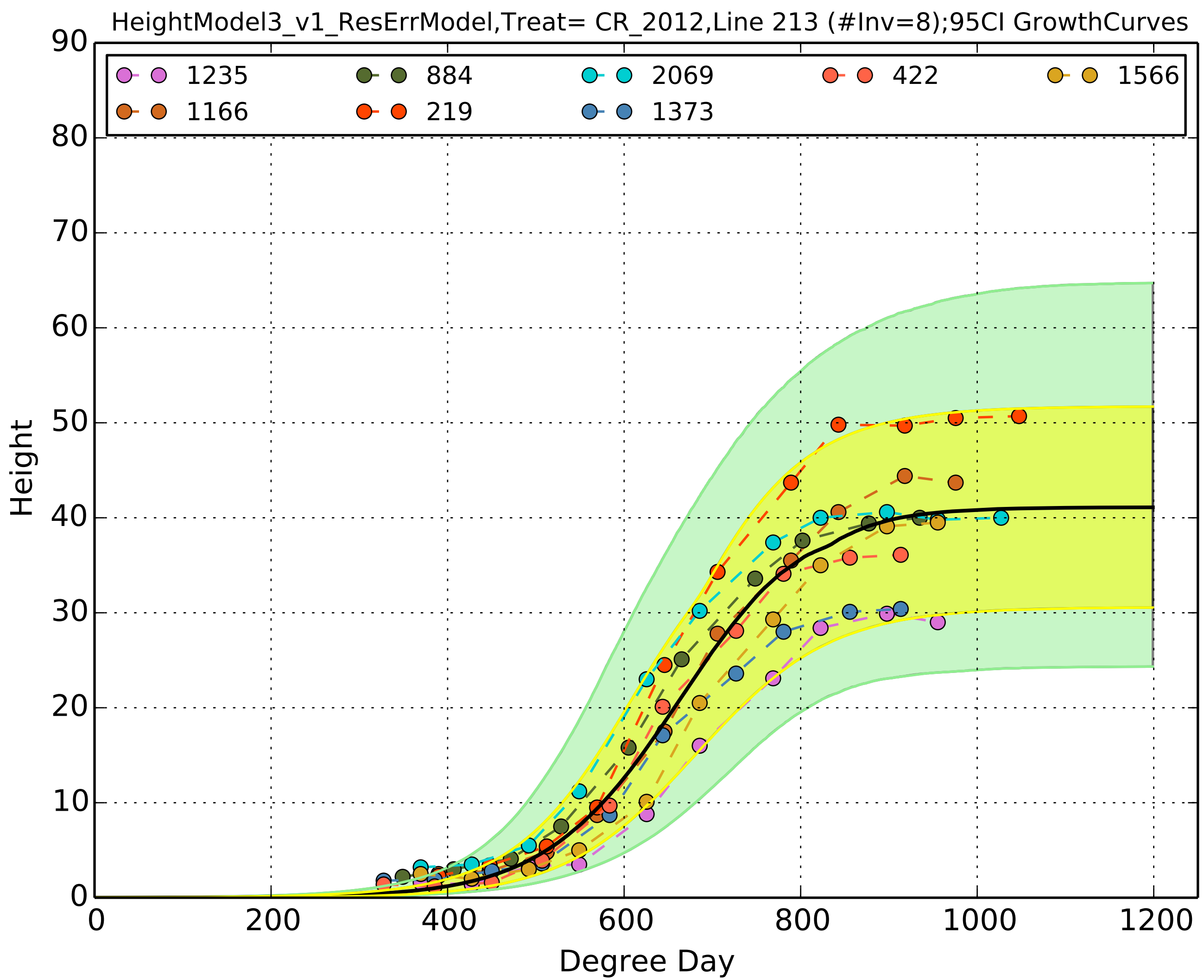

HeightModel3_v1_ResErrModel,Treat= CR_2012,Line 213 (#Inv=8);95CI GrowthCurves
1235
884
2069
422
1566
1166
219
1373
Height
Degree Day
0
10
20
30
40
50
60
70
80
90
0
200
400
600
800
1000
1200

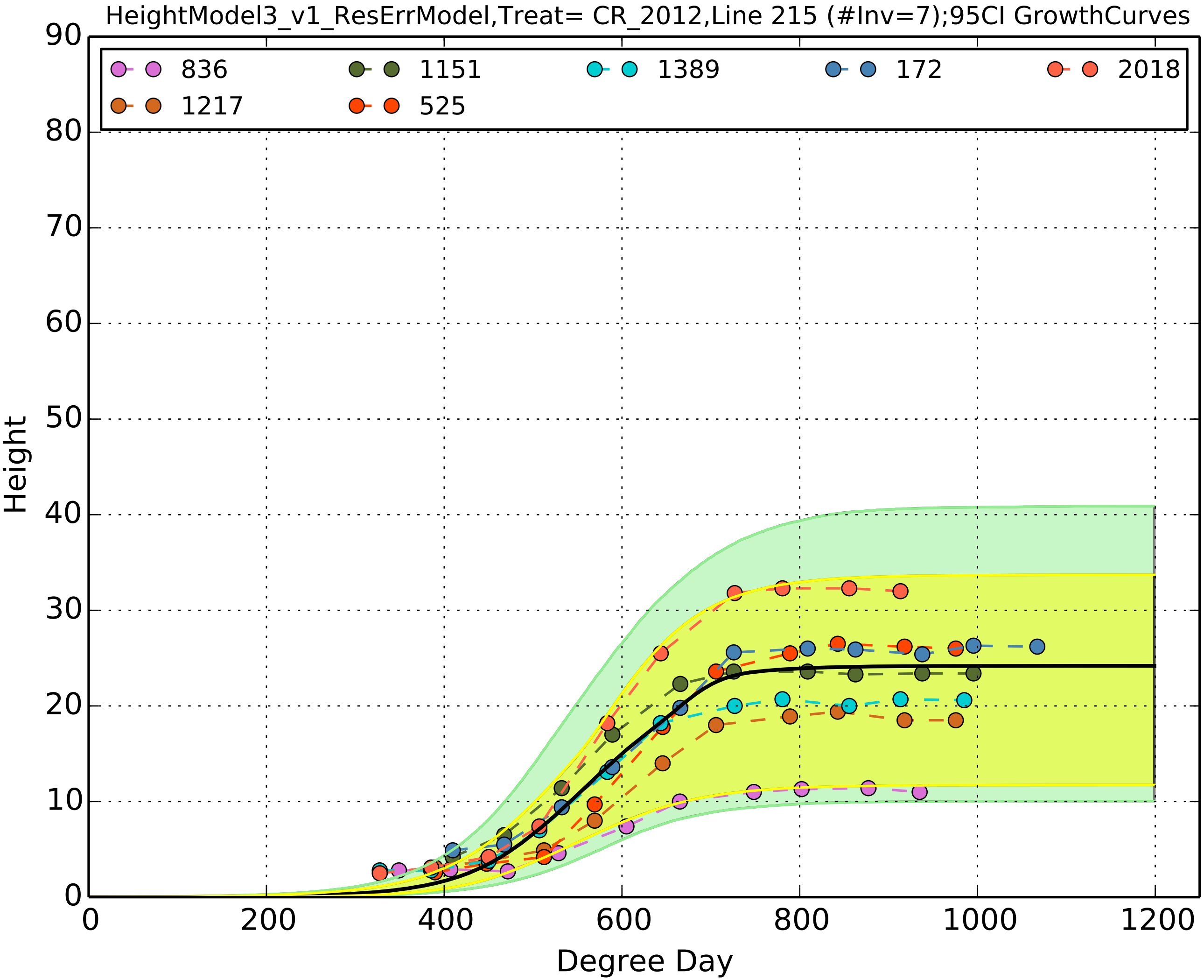
HeightModel3_v1_ResErrModel,Treat= CR_2012,Line 215 (#Inv=7);95CI GrowthCurves
836
1151
1389
172
2018
1217
525
Height
Degree Day
0
10
20
30
40
50
60
70
80
90
0
200
400
600
800
1000
1200

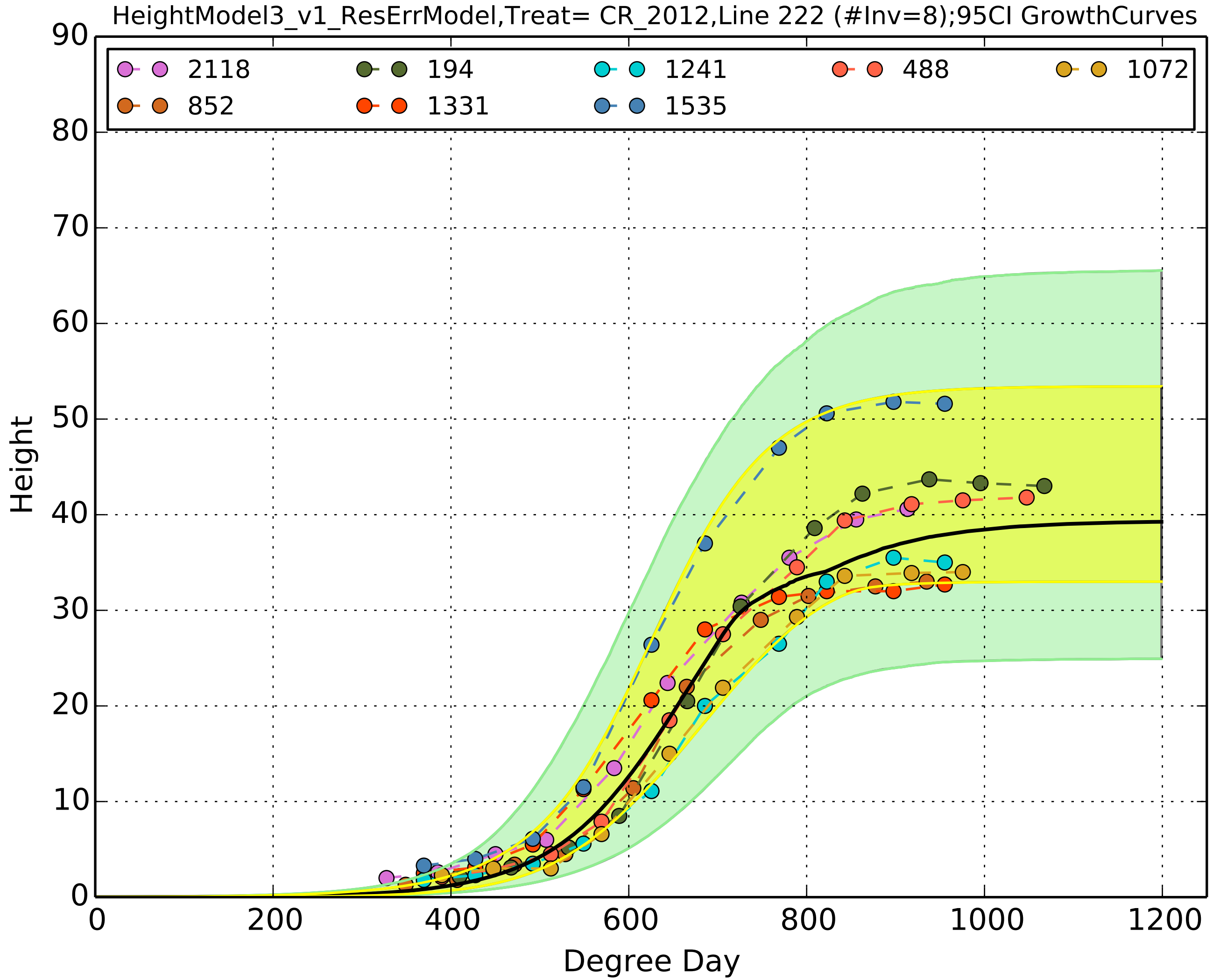

HeightModel3_v1_ResErrModel,Treat= CR_2012,Line 222 (#Inv=8);95CI GrowthCurves
2118
194
1241
488
1072
852
1331
1535
Height
Degree Day
0
10
20
30
40
50
60
70
80
90
0
200
400
600
800
1000
1200

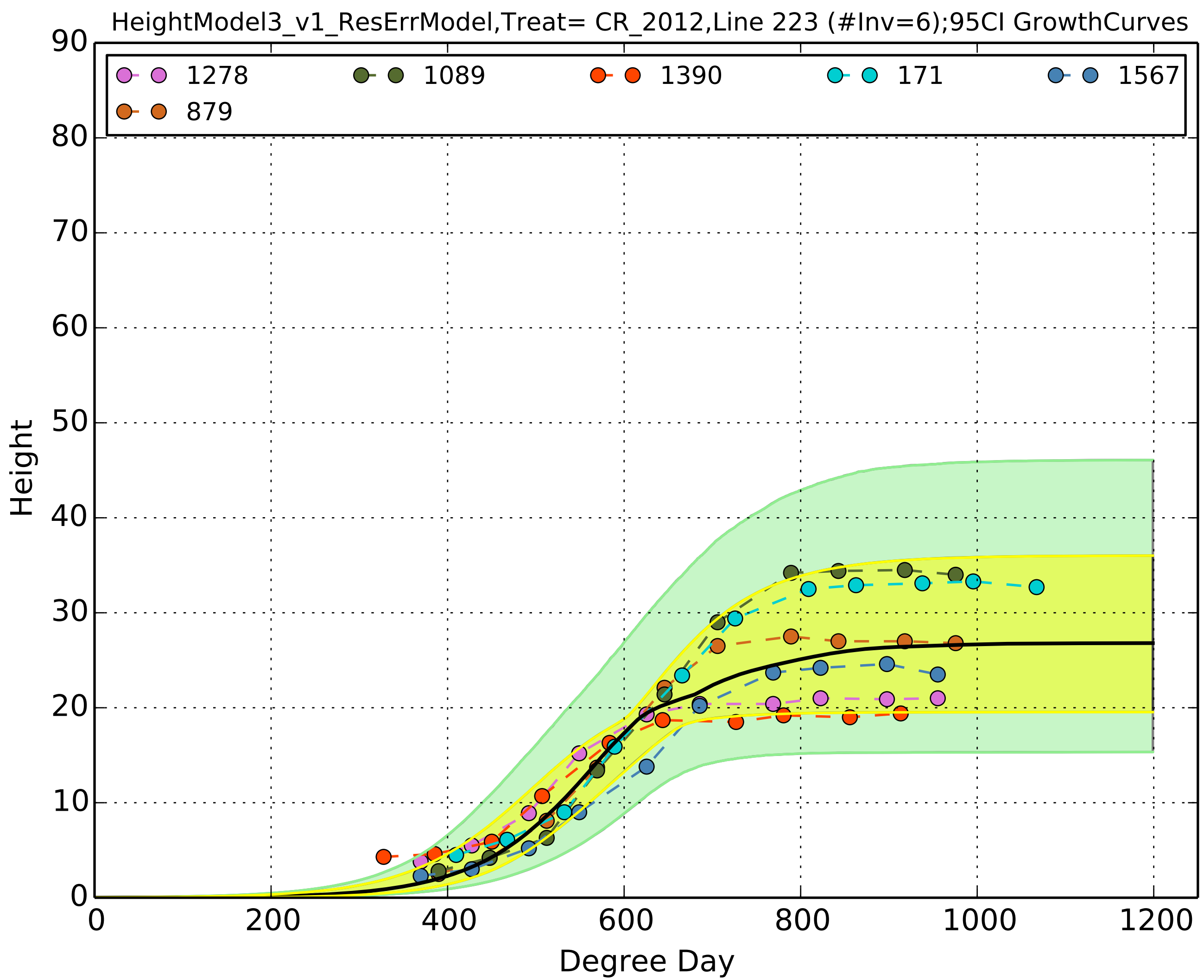

HeightModel3_v1_ResErrModel,Treat= CR_2012,Line 223 (#Inv=6);95CI GrowthCurves
1278
1089
1390
171
1567
879
Height
Degree Day
0
10
20
30
40
50
60
70
80
90
0
200
400
600
800
1000
1200

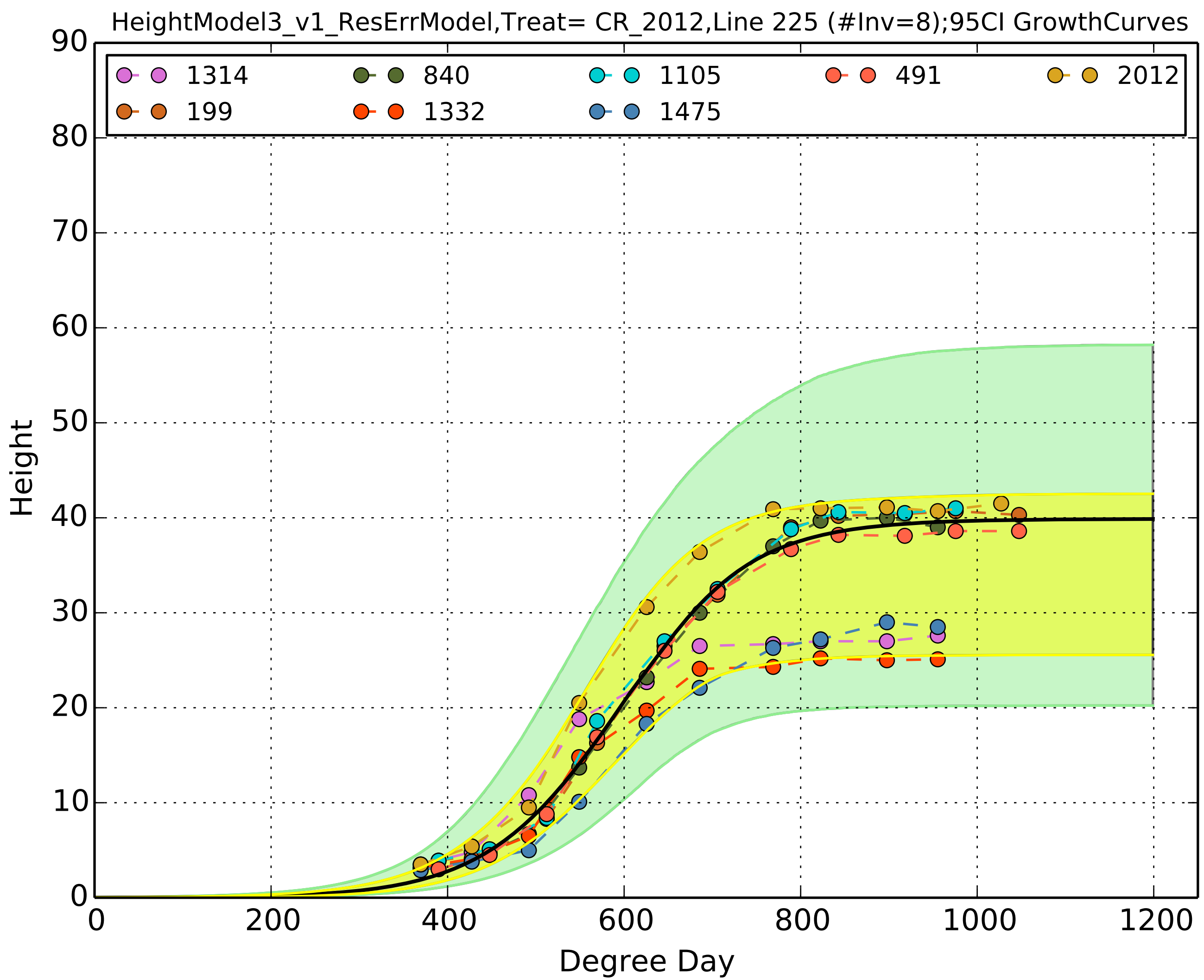
HeightModel3_v1_ResErrModel,Treat= CR_2012,Line 225 (#Inv=8);95CI GrowthCurves
1314
840
1105
491
2012
199
1332
1475
Height
Degree Day
0
10
20
30
40
50
60
70
80
90
0
200
400
600
800
1000
1200

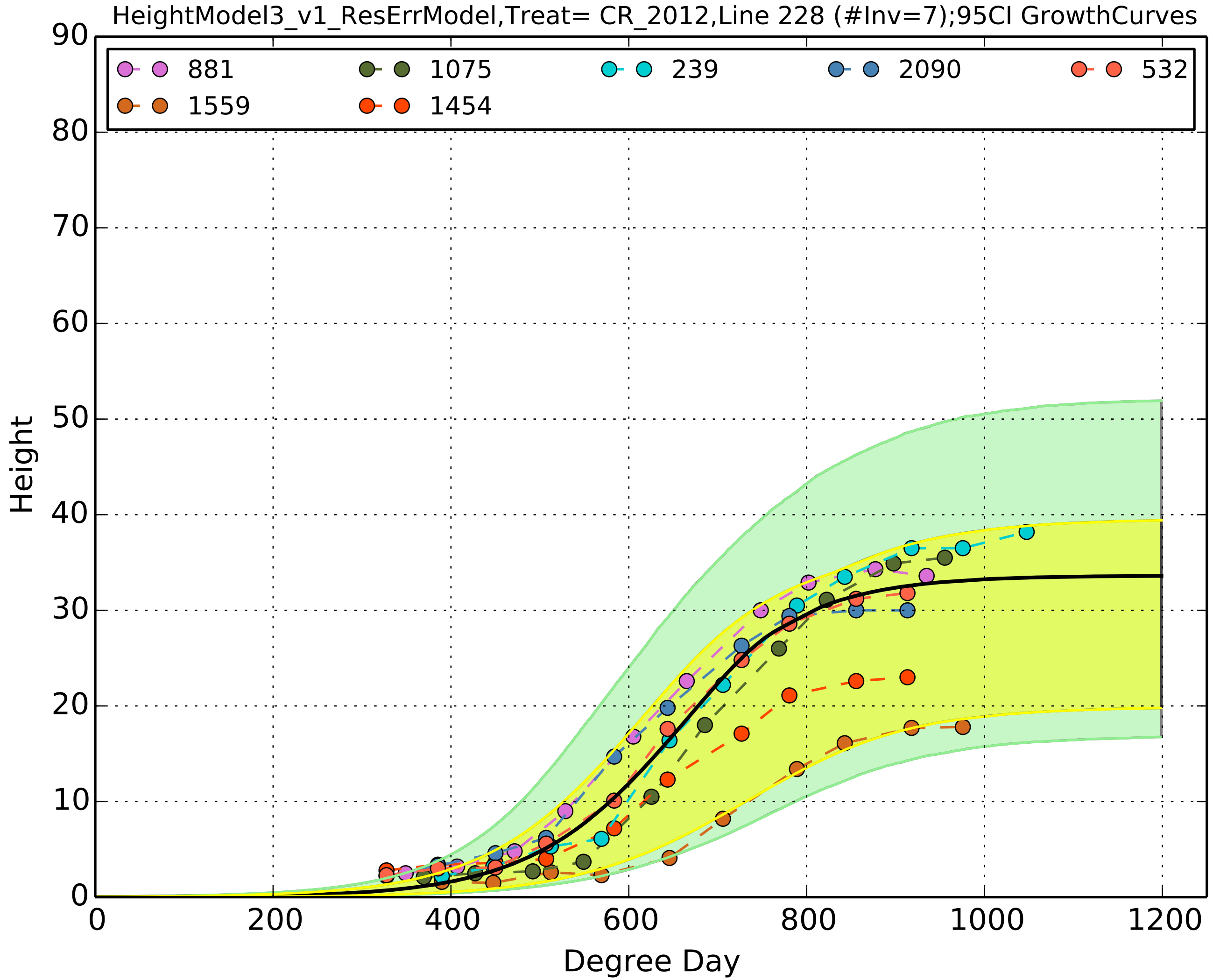
HeightModel3_v1_ResErrModel,Treat= CR_2012,Line 228 (#Inv=7);95CI GrowthCurves
881
1075
239
2090
532
1559
1454
Height
Degree Day
0
10
20
30
40
50
60
70
80
90
0
200
400
600
800
1000
1200

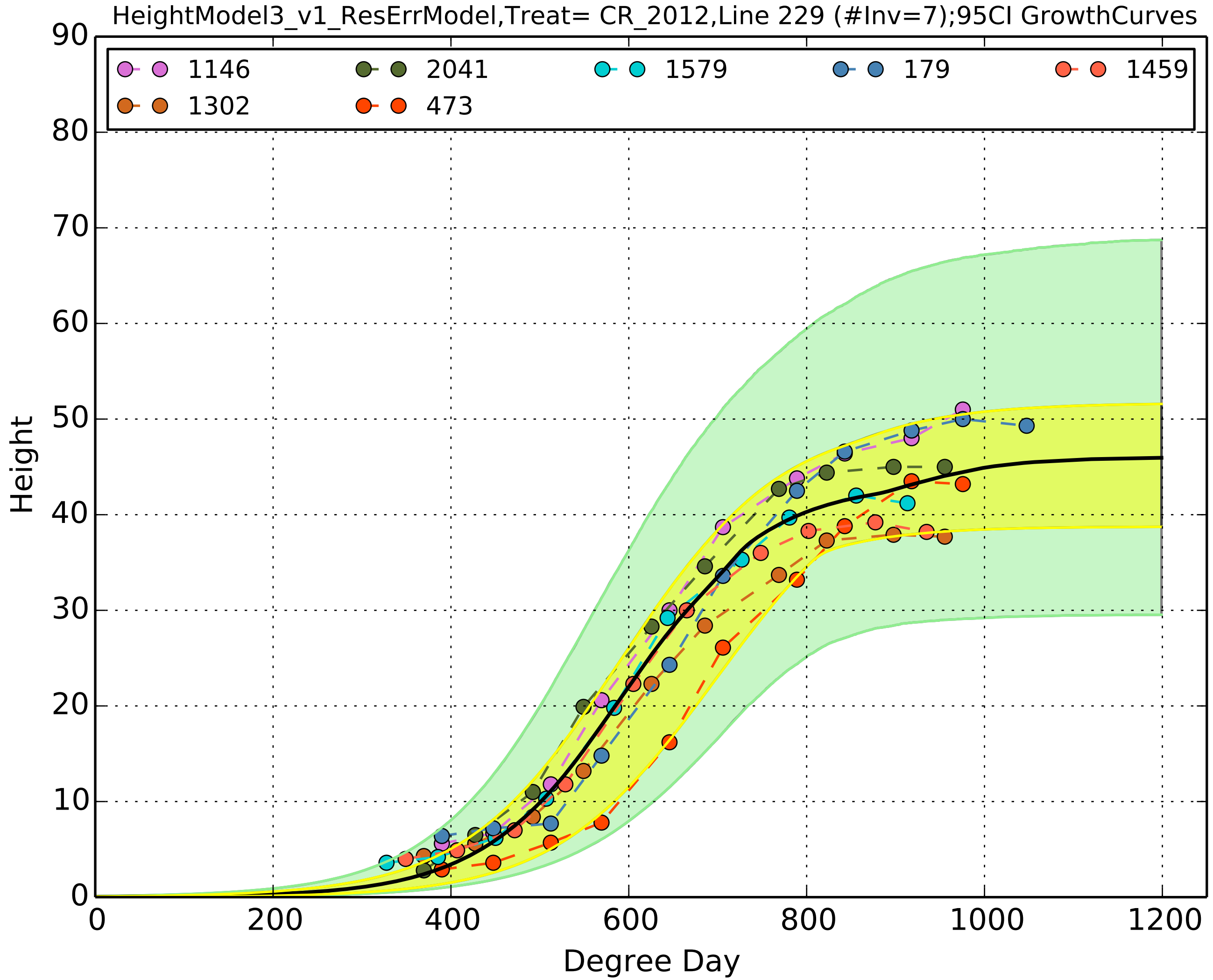

HeightModel3_v1_ResErrModel,Treat= CR_2012,Line 229 (#Inv=7);95CI GrowthCurves
1146
2041
1579
179
1459
1302
473
Height
Degree Day
0
10
20
30
40
50
60
70
80
90
0
200
400
600
800
1000
1200

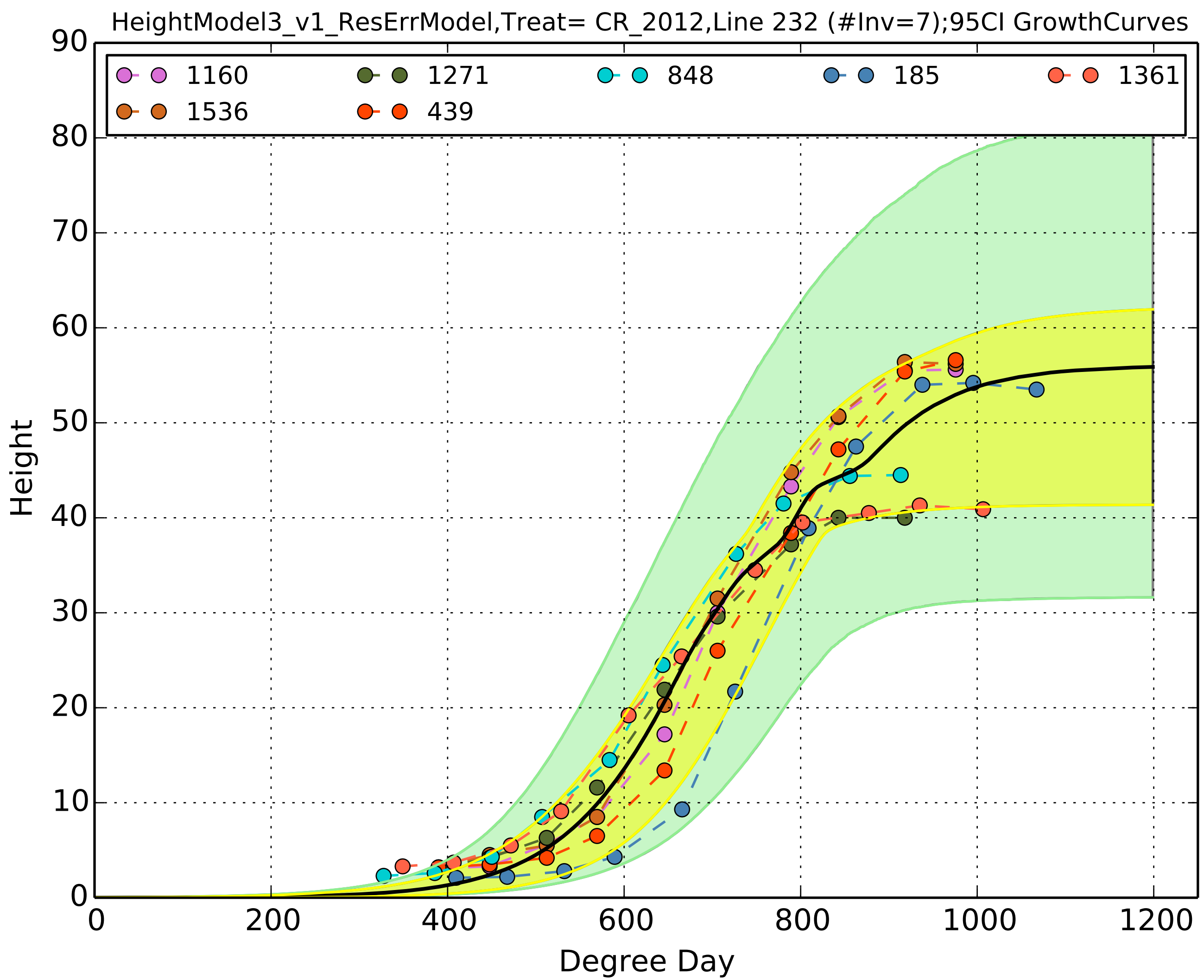
HeightModel3_v1_ResErrModel,Treat= CR_2012,Line 232 (#Inv=7);95CI GrowthCurves
1160
1271
848
185
1361
1536
439
Height
Degree Day
0
10
20
30
40
50
60
70
80
90
0
200
400
600
800
1000
1200

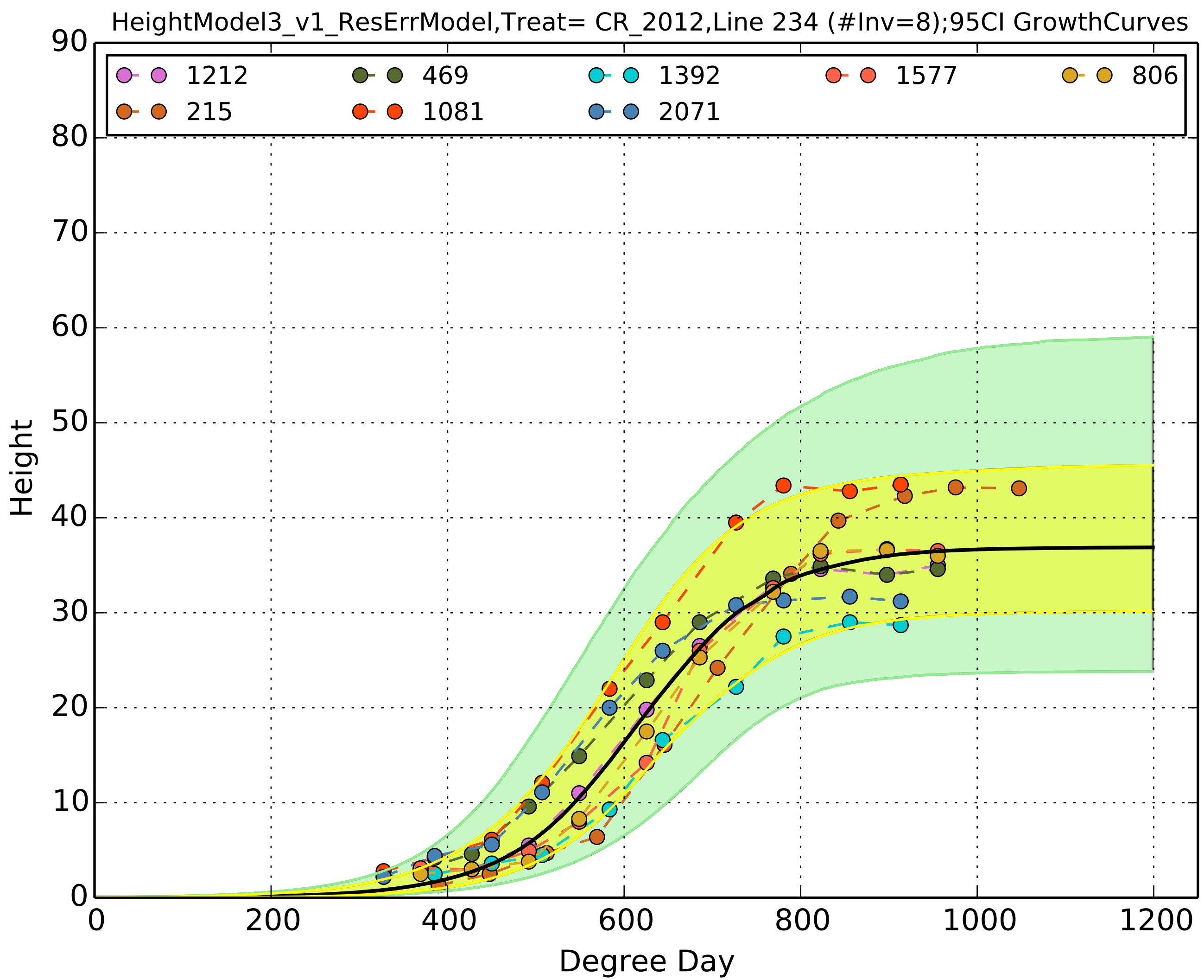

HeightModel3_v1_ResErrModel,Treat= CR_2012,Line 234 (#Inv=8);95CI GrowthCurves
1212
469
1392
1577
806
215
1081
2071
Height
Degree Day
0
10
20
30
40
50
60
70
80
90
0
200
400
600
800
1000
1200

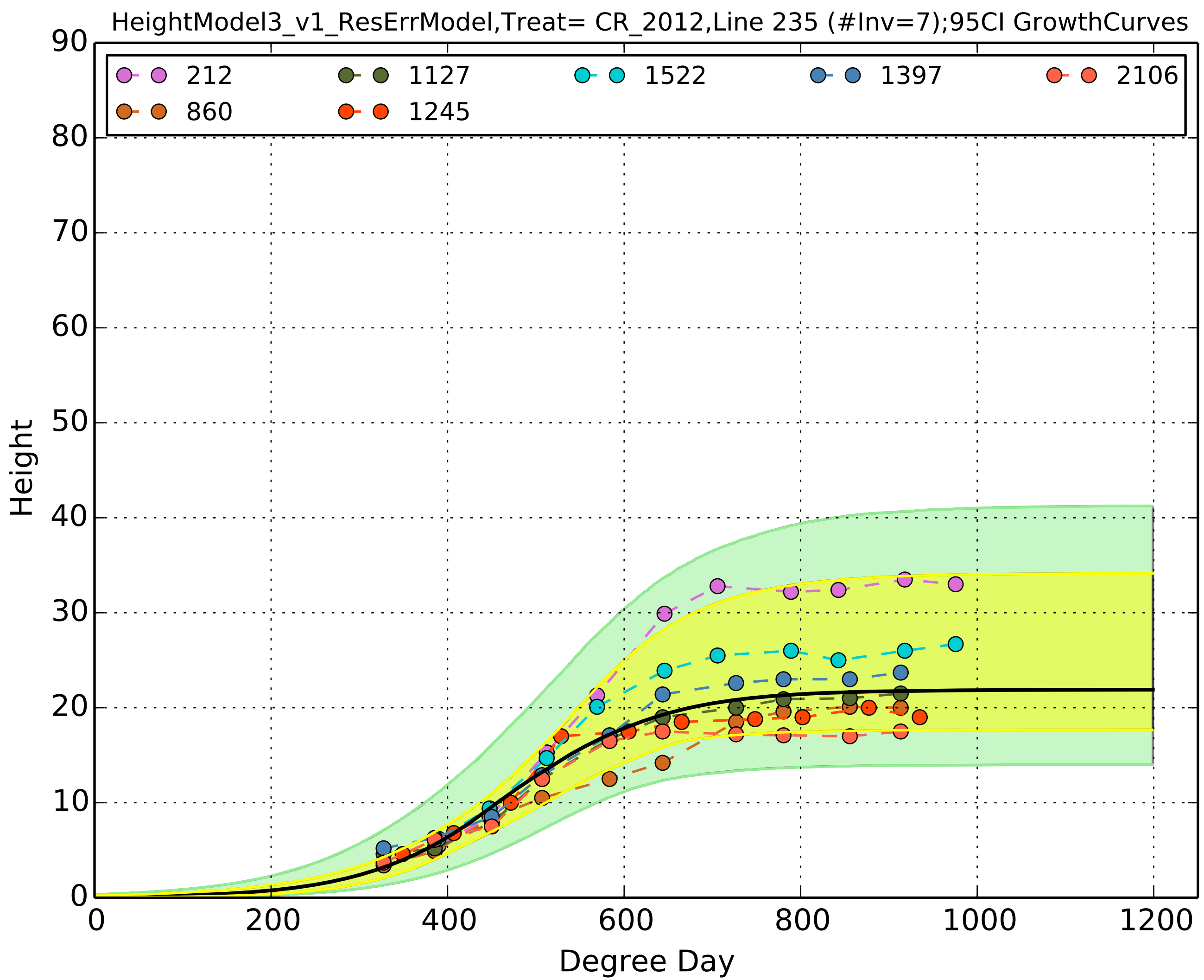
HeightModel3_v1_ResErrModel,Treat= CR_2012,Line 235 (#Inv=7);95CI GrowthCurves
212
1127
1522
1397
2106
860
1245
Height
Degree Day
0
10
20
30
40
50
60
70
80
90
0
200
400
600
800
1000
1200

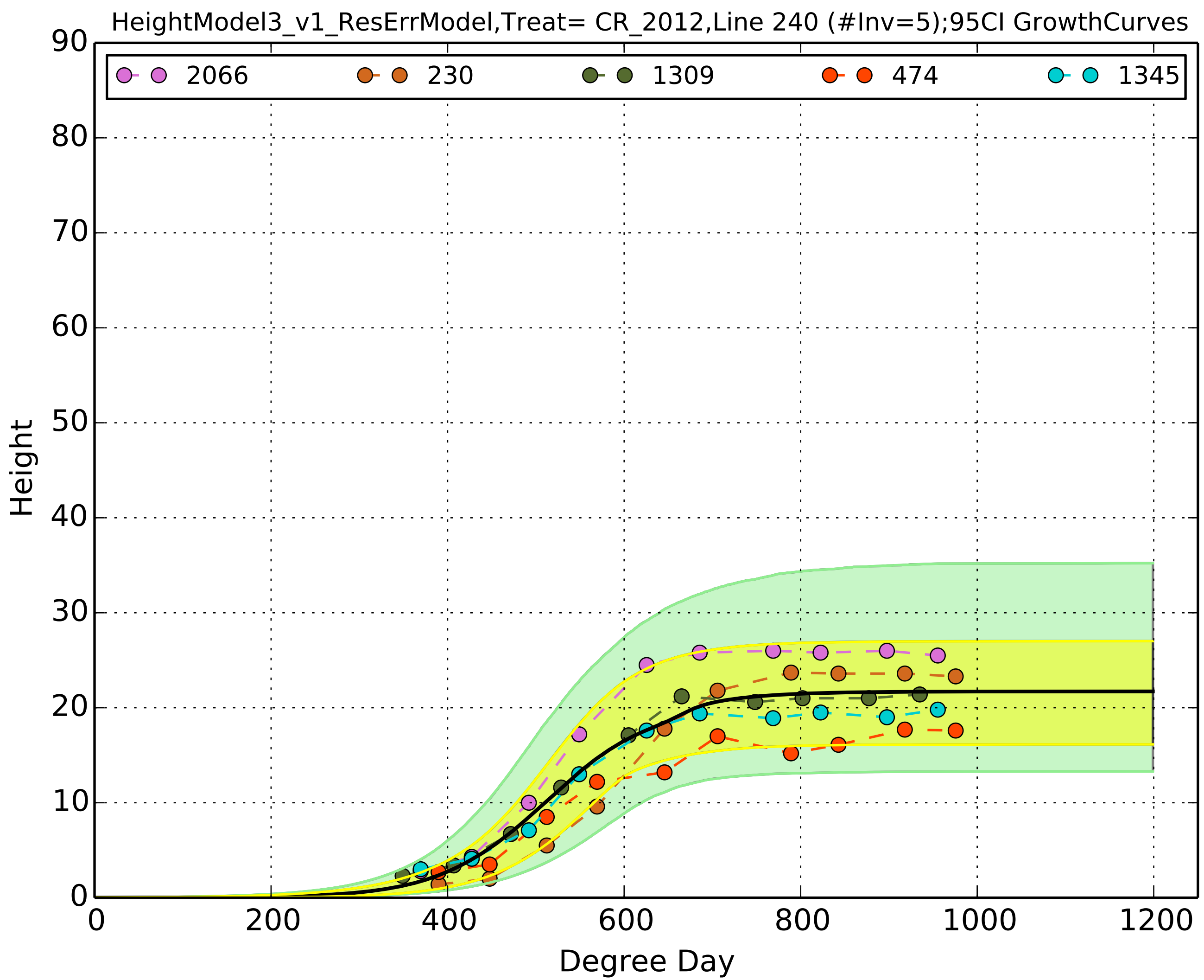

HeightModel3_v1_ResErrModel,Treat= CR_2012,Line 240 (#Inv=5);95CI GrowthCurves
2066
230
1309
474
1345
Height
Degree Day
0
10
20
30
40
50
60
70
80
90
0
200
400
600
800
1000
1200

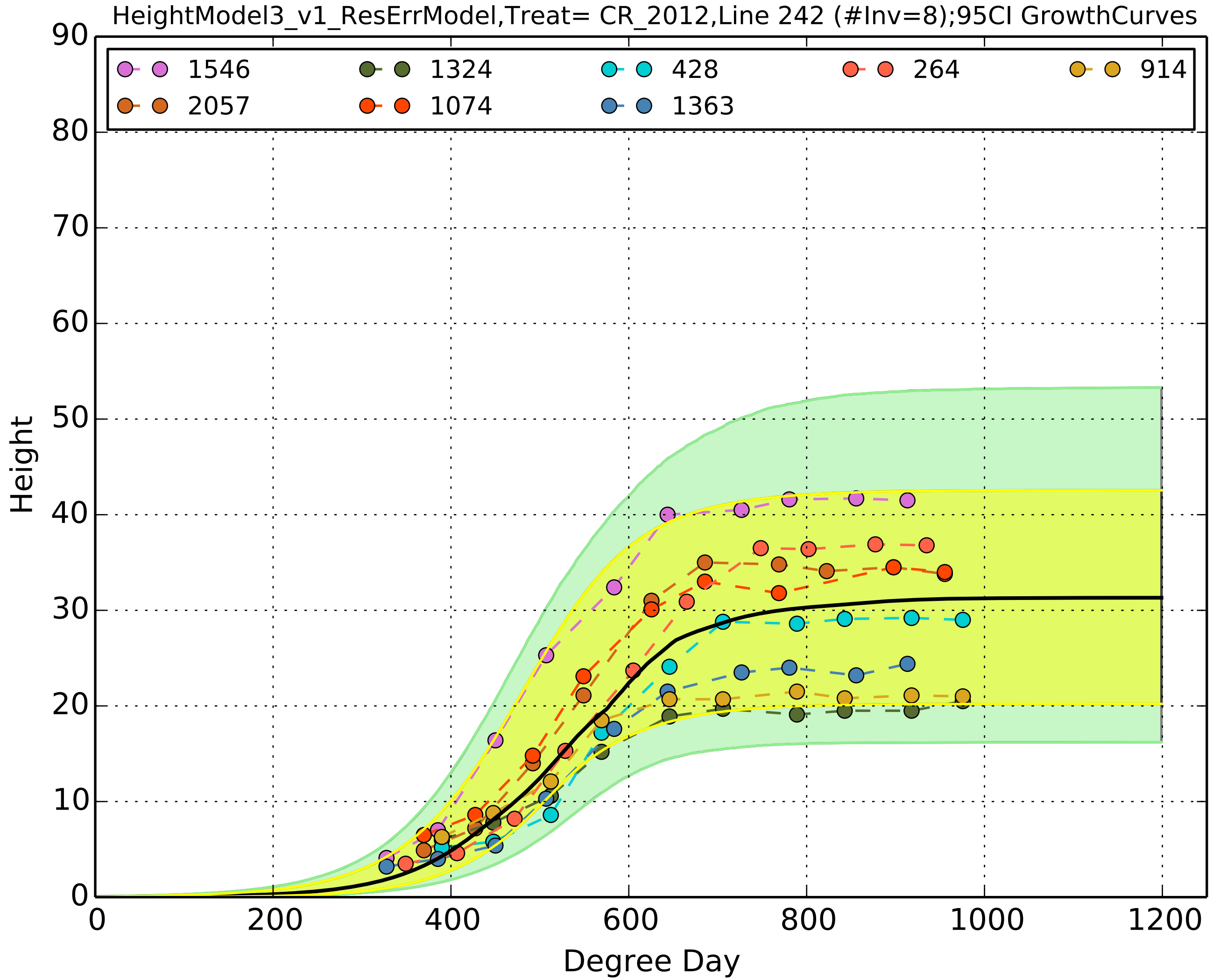
HeightModel3_v1_ResErrModel,Treat= CR_2012,Line 242 (#Inv=8);95CI GrowthCurves
1546
1324
428
264
914
2057
1074
1363
Height
Degree Day
0
10
20
30
40
50
60
70
80
90
0
200
400
600
800
1000
1200

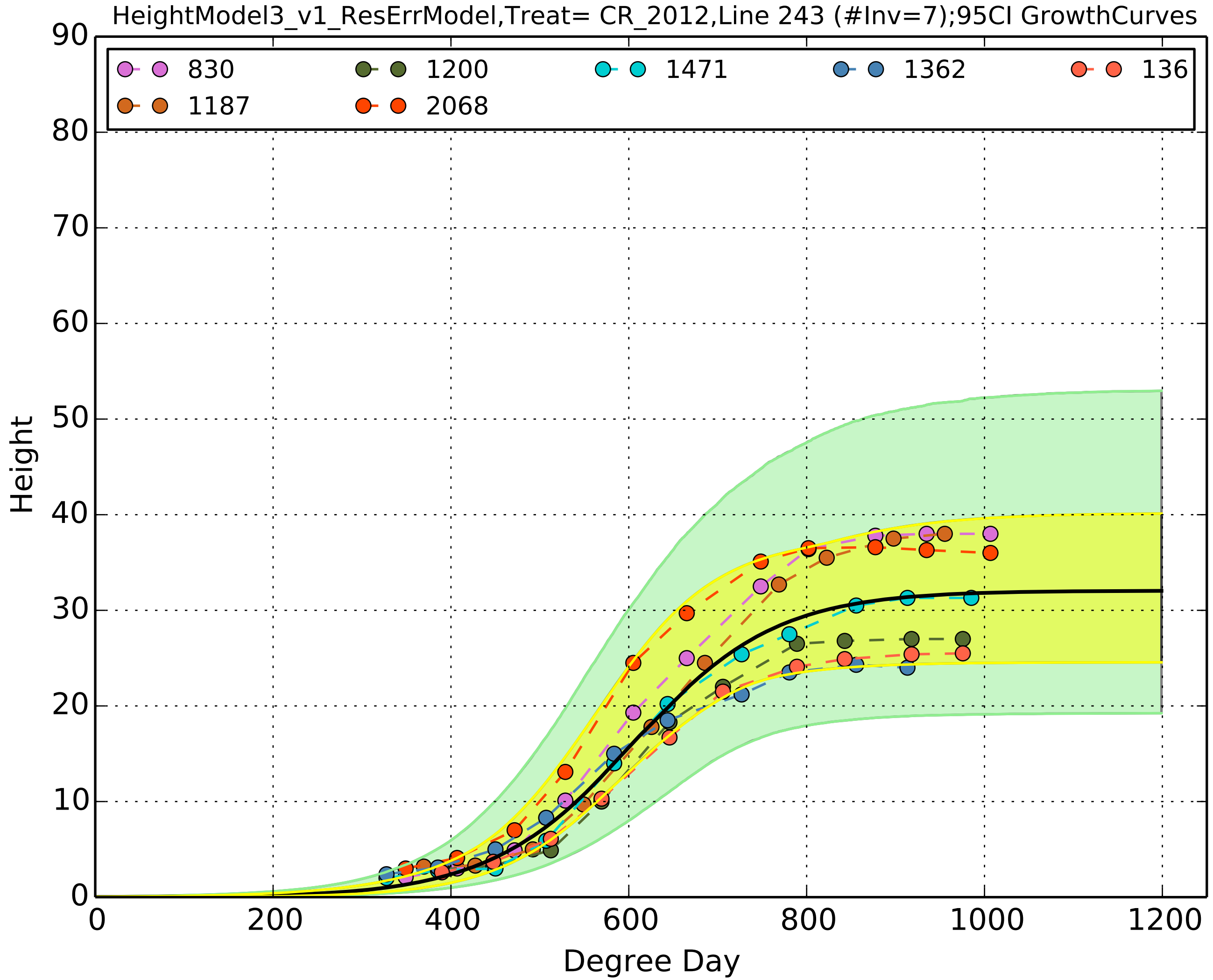

HeightModel3_v1_ResErrModel,Treat= CR_2012,Line 243 (#Inv=7);95CI GrowthCurves
830
1200
1471
1362
136
1187
2068
Height
Degree Day
0
10
20
30
40
50
60
70
80
90
0
200
400
600
800
1000
1200

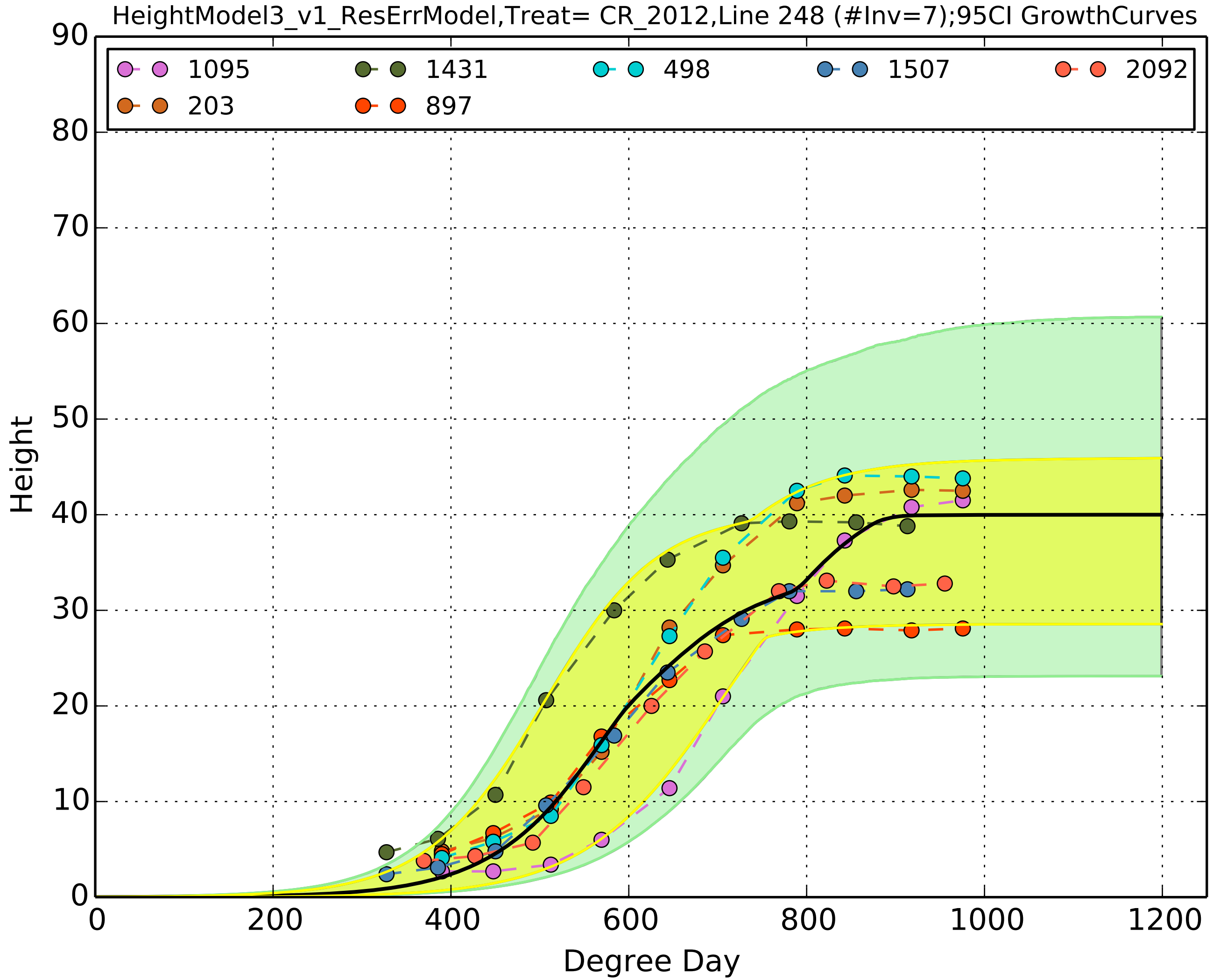
HeightModel3_v1_ResErrModel,Treat= CR_2012,Line 248 (#Inv=7);95CI GrowthCurves
1095
1431
498
1507
2092
203
897
Height
Degree Day
0
10
20
30
40
50
60
70
80
90
0
200
400
600
800
1000
1200

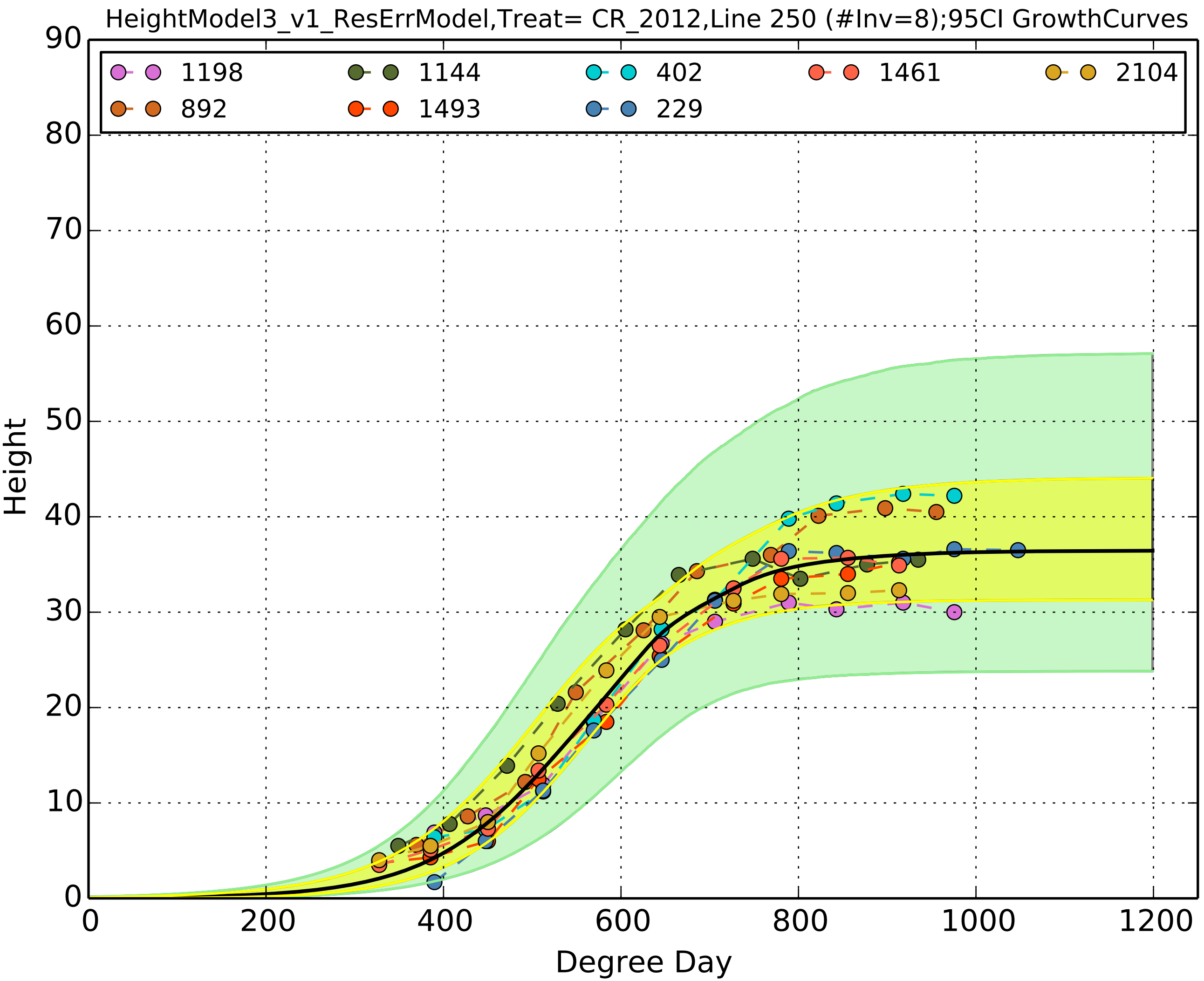

HeightModel3_v1_ResErrModel,Treat= CR_2012,Line 250 (#Inv=8);95CI GrowthCurves
1198
1144
402
1461
2104
892
1493
229
Height
Degree Day
0
10
20
30
40
50
60
70
80
90
0
200
400
600
800
1000
1200

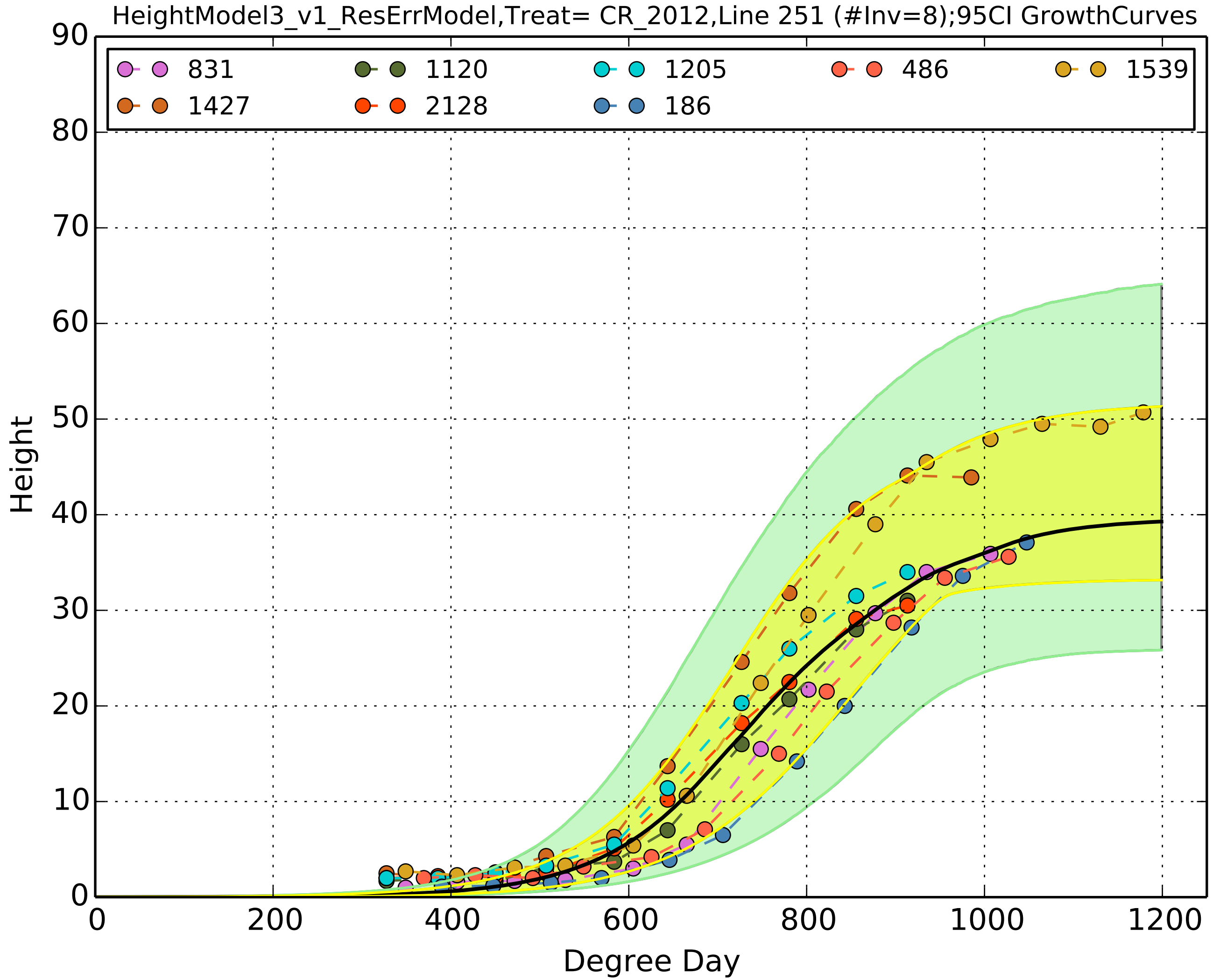

HeightModel3_v1_ResErrModel,Treat= CR_2012,Line 251 (#Inv=8);95CI GrowthCurves
831
1120
1205
486
1539
1427
2128
186
Height
Degree Day
0
10
20
30
40
50
60
70
80
90
0
200
400
600
800
1000
1200

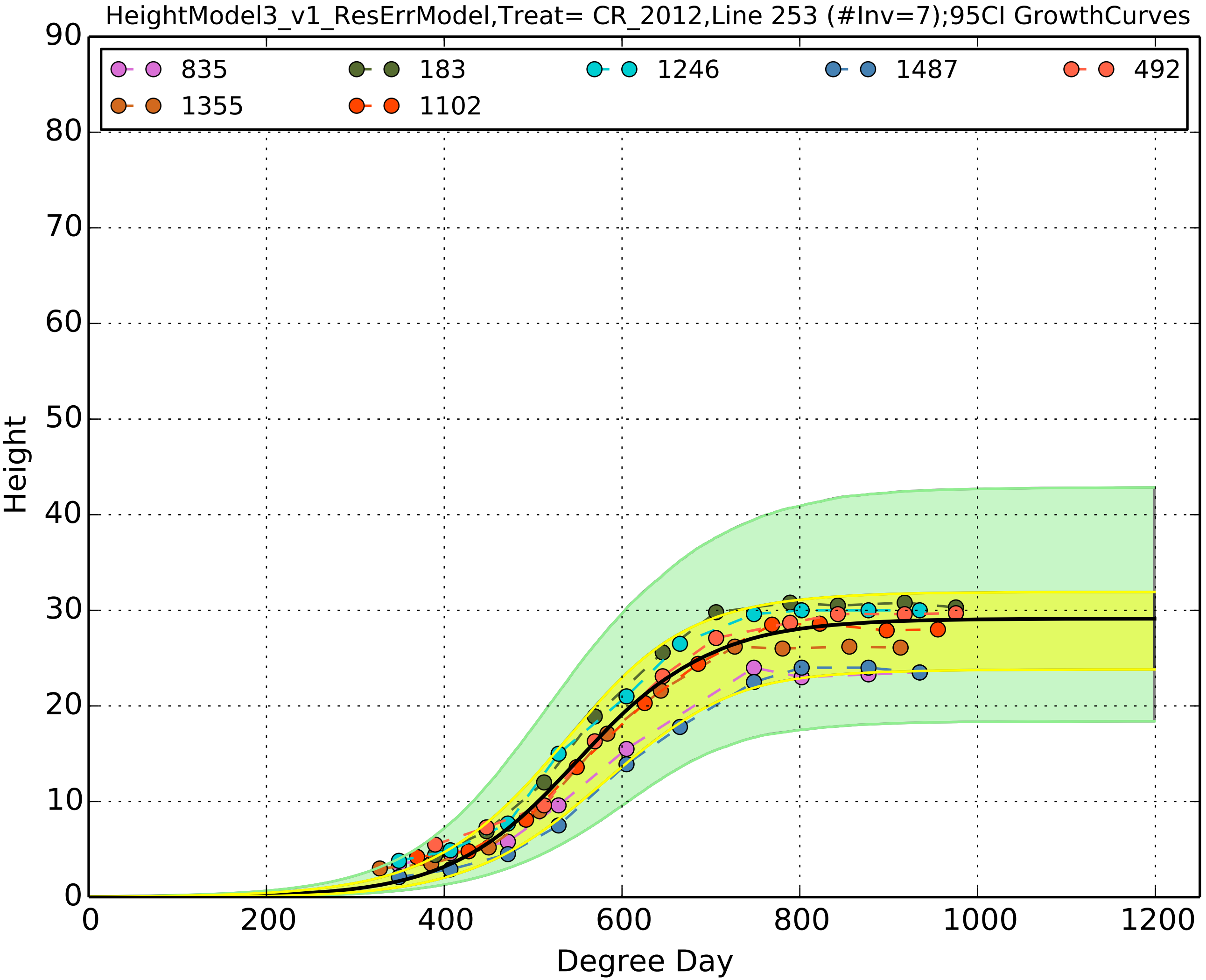

HeightModel3_v1_ResErrModel,Treat= CR_2012,Line 253 (#Inv=7);95CI GrowthCurves
835
183
1246
1487
492
1355
1102
Height
Degree Day
0
10
20
30
40
50
60
70
80
90
0
200
400
600
800
1000
1200

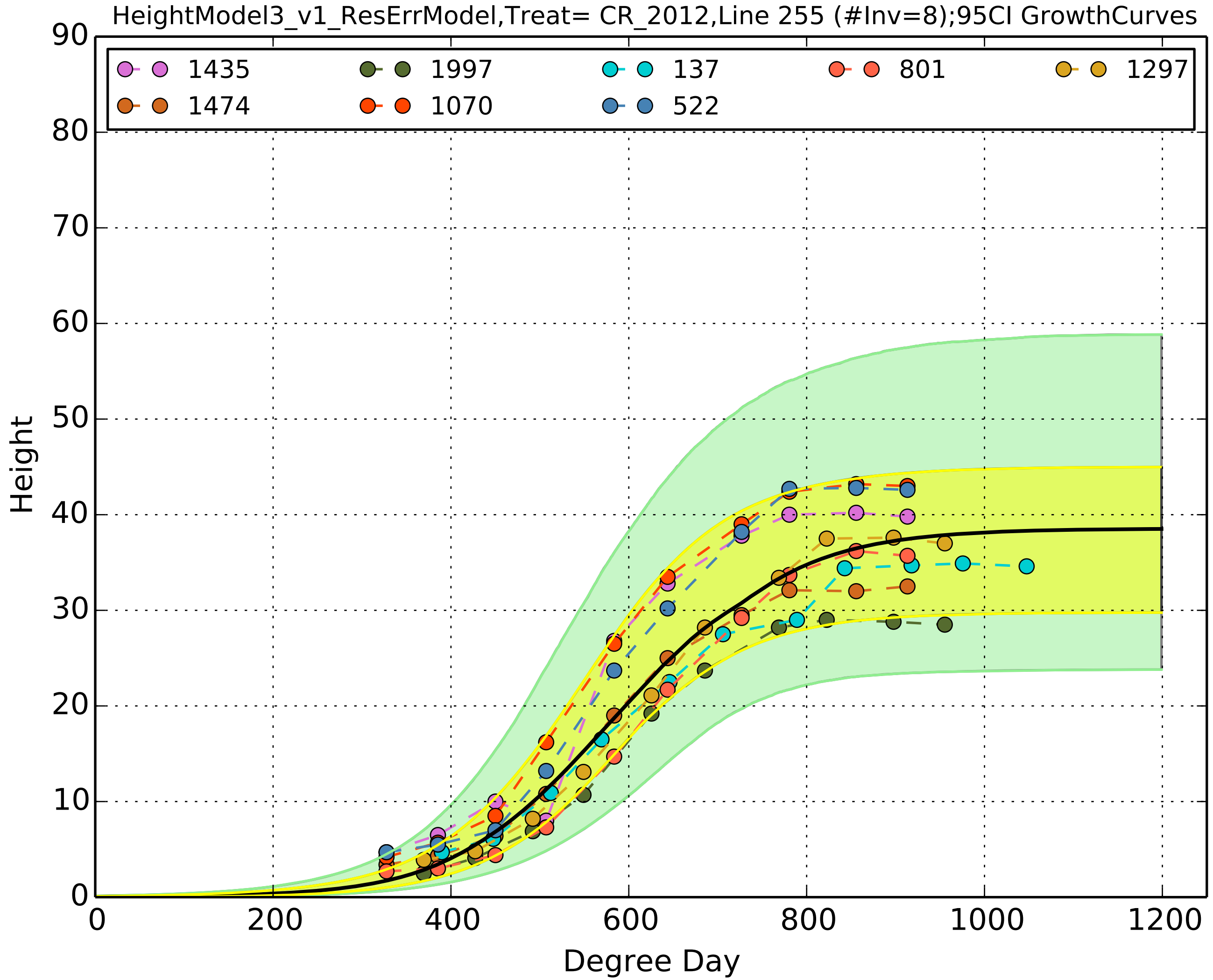

HeightModel3_v1_ResErrModel,Treat= CR_2012,Line 255 (#Inv=8);95CI GrowthCurves
1435
1997
137
801
1297
1474
1070
522
Height
Degree Day
0
10
20
30
40
50
60
70
80
90
0
200
400
600
800
1000
1200

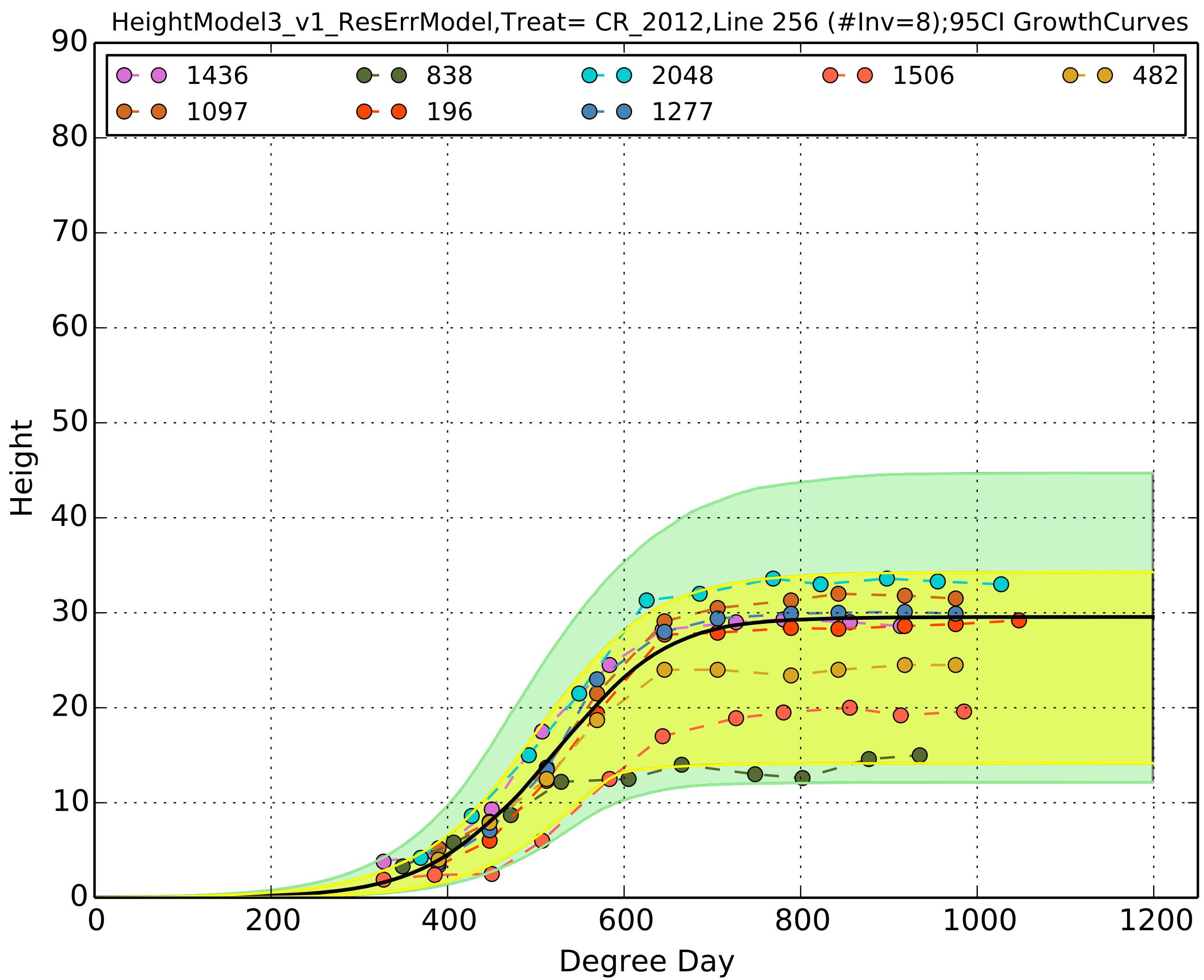
HeightModel3_v1_ResErrModel,Treat= CR_2012,Line 256 (#Inv=8);95CI GrowthCurves
1436
838
2048
1506
482
1097
196
1277
Height
Degree Day
0
10
20
30
40
50
60
70
80
90
0
200
400
600
800
1000
1200

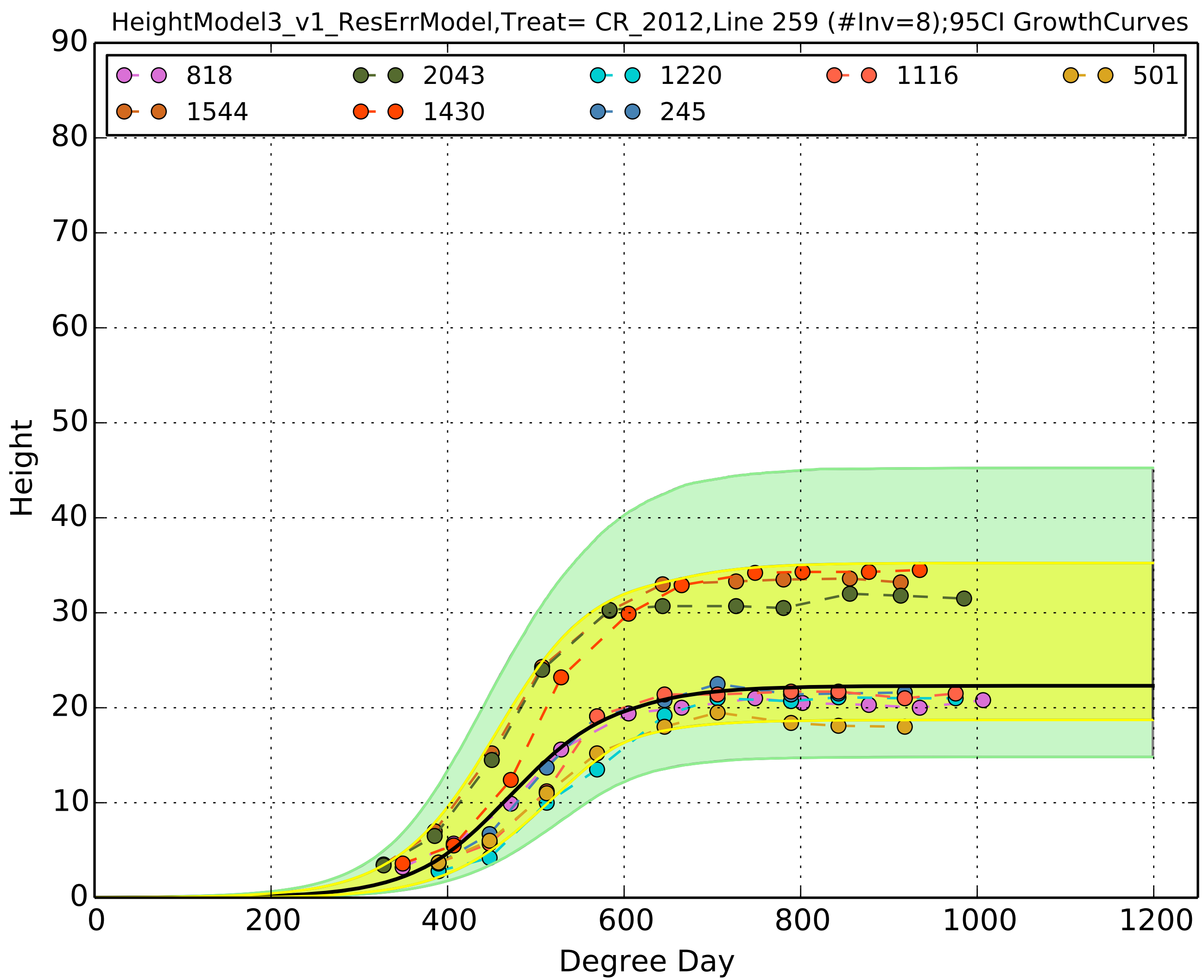

HeightModel3_v1_ResErrModel,Treat= CR_2012,Line 259 (#Inv=8);95CI GrowthCurves
818
2043
1220
1116
501
1544
1430
245
Height
Degree Day
0
10
20
30
40
50
60
70
80
90
0
200
400
600
800
1000
1200

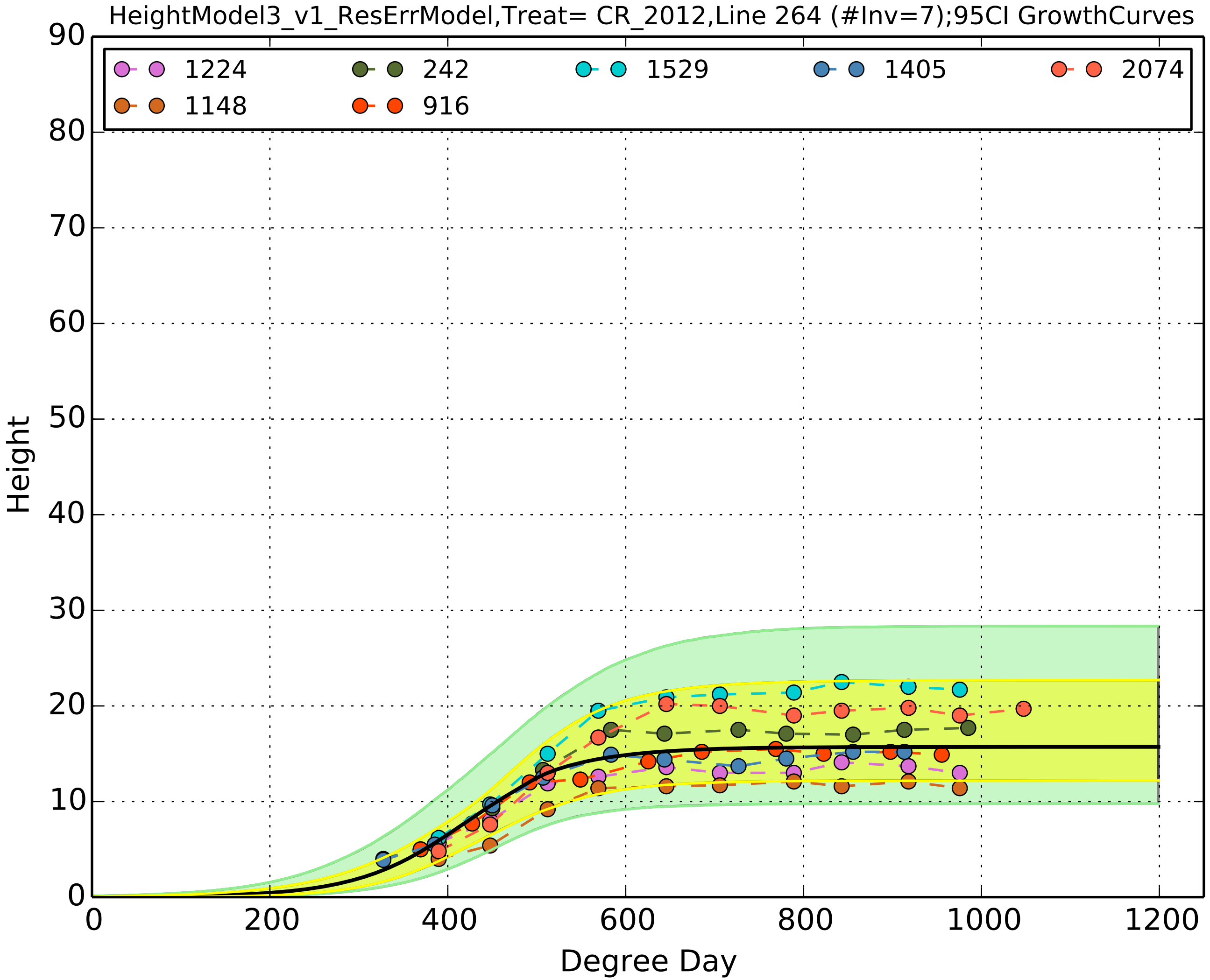
HeightModel3_v1_ResErrModel,Treat= CR_2012,Line 264 (#Inv=7);95CI GrowthCurves
1224
242
1529
1405
2074
1148
916
Height
Degree Day
0
10
20
30
40
50
60
70
80
90
0
200
400
600
800
1000
1200

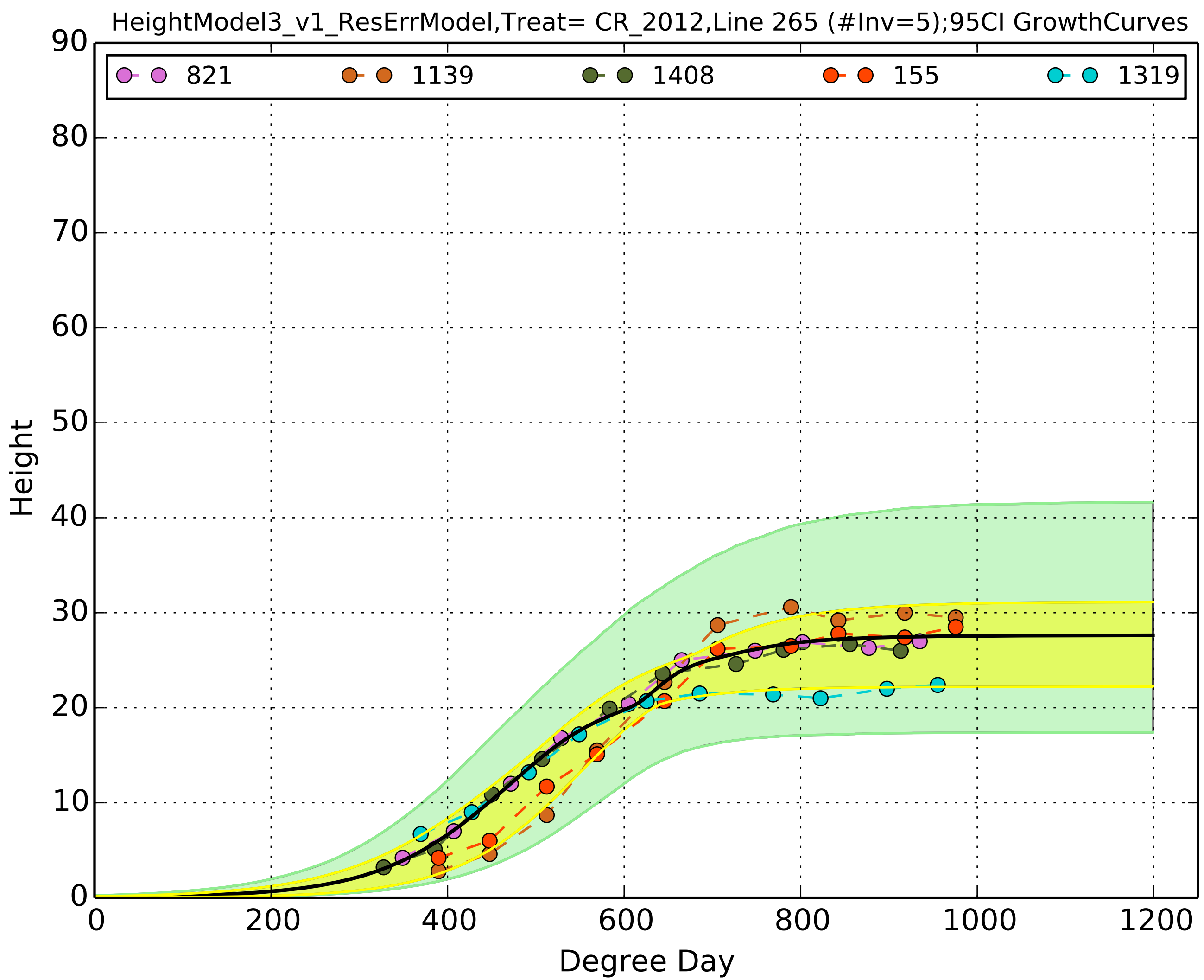

HeightModel3_v1_ResErrModel,Treat= CR_2012,Line 265 (#Inv=5);95CI GrowthCurves
821
1139
1408
155
1319
Height
Degree Day
0
10
20
30
40
50
60
70
80
90
0
200
400
600
800
1000
1200

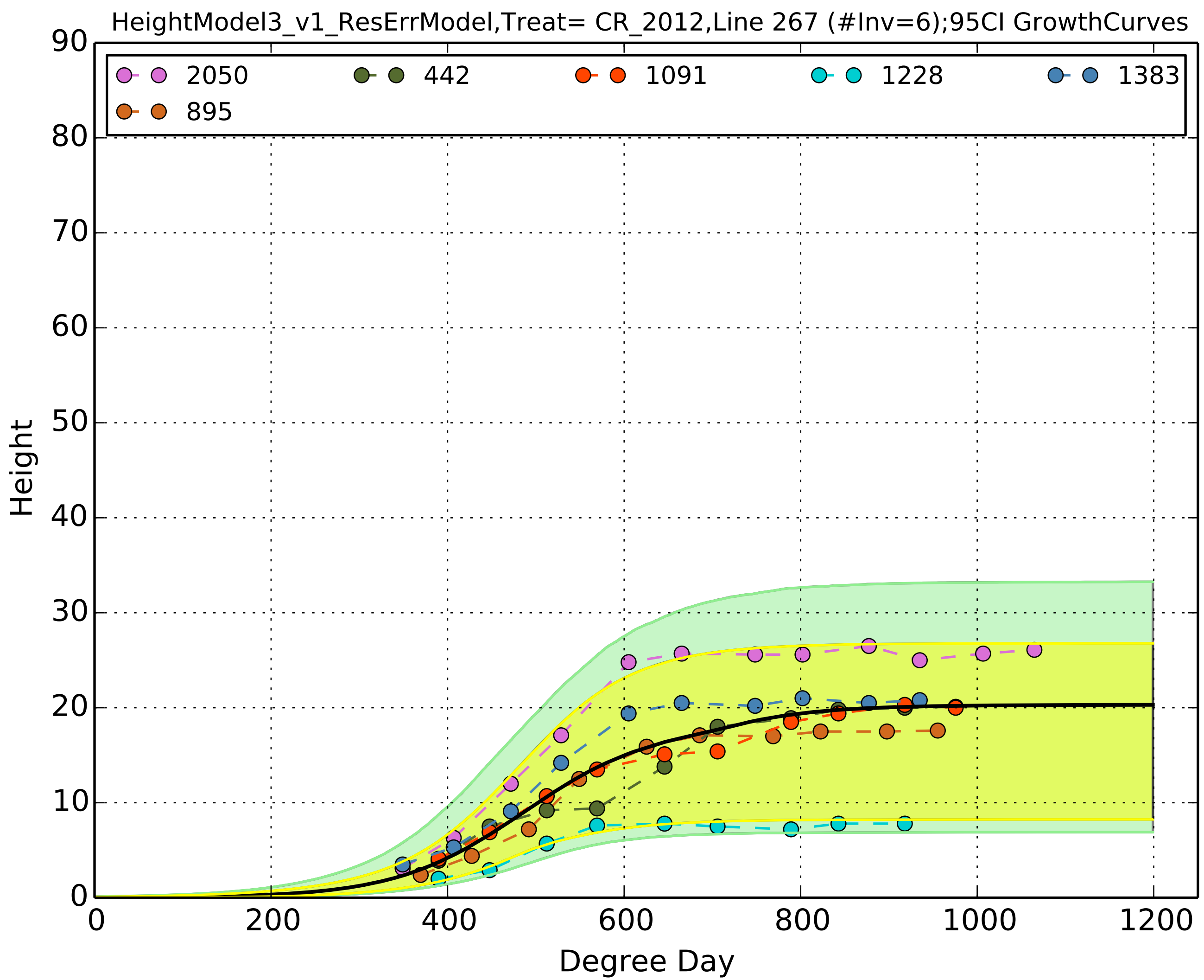

HeightModel3_v1_ResErrModel,Treat= CR_2012,Line 267 (#Inv=6);95CI GrowthCurves
2050
442
1091
1228
1383
895
Height
Degree Day
0
10
20
30
40
50
60
70
80
90
0
200
400
600
800
1000
1200

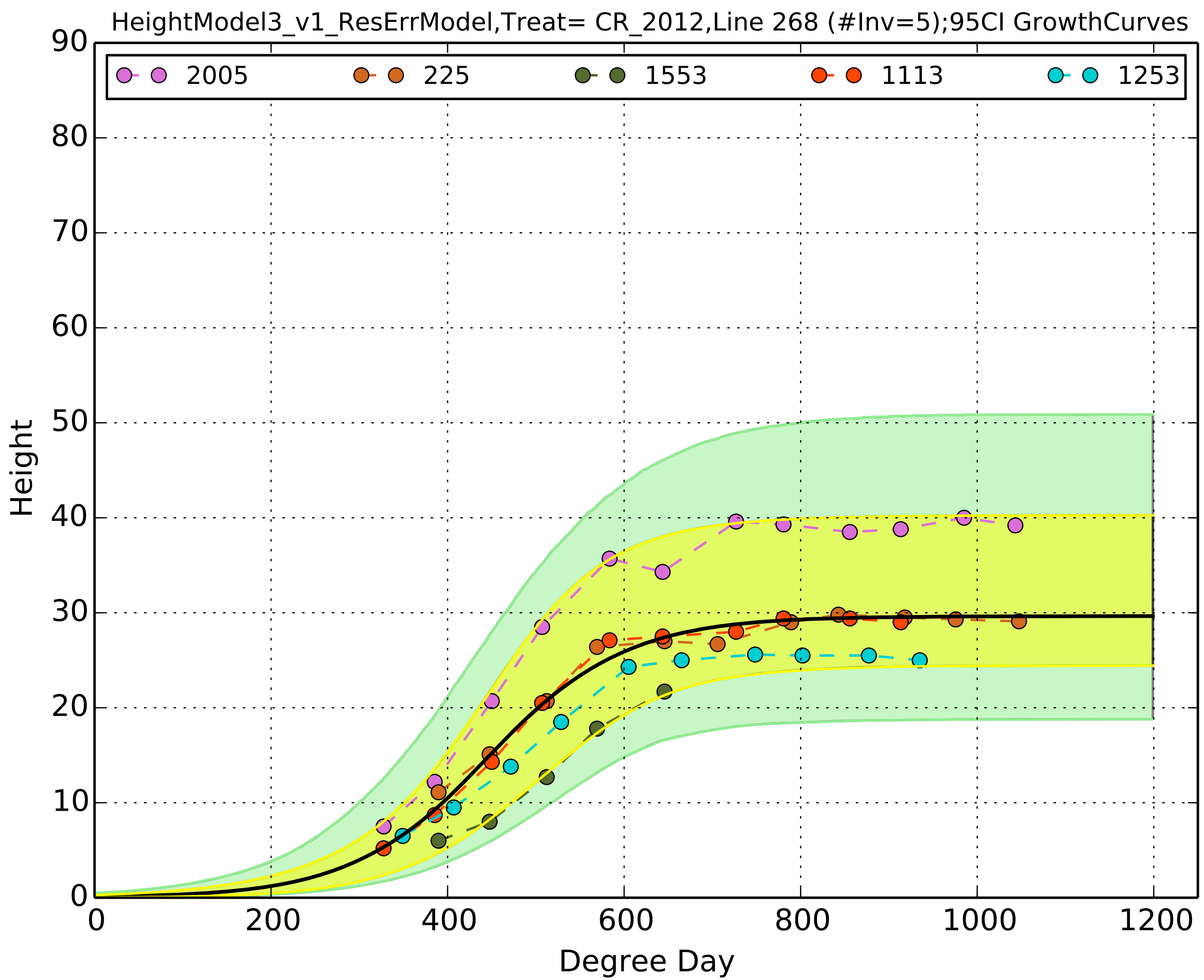

HeightModel3_v1_ResErrModel,Treat= CR_2012,Line 268 (#Inv=5);95CI GrowthCurves
2005
225
1553
1113
1253
Height
Degree Day
0
10
20
30
40
50
60
70
80
90
0
200
400
600
800
1000
1200

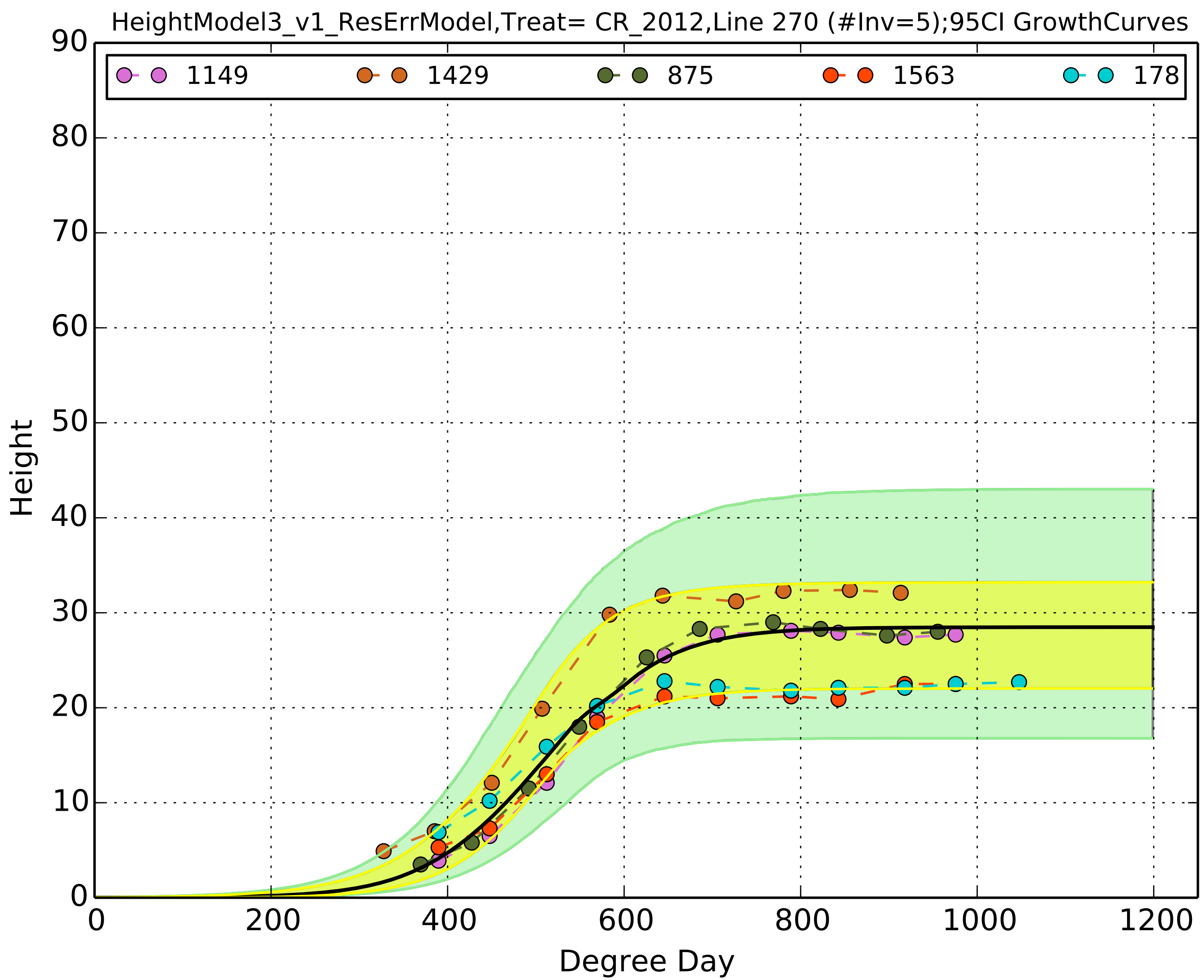

HeightModel3_v1_ResErrModel,Treat= CR_2012,Line 270 (#Inv=5);95CI GrowthCurves
1149
1429
875
1563
178
Height
Degree Day
0
10
20
30
40
50
60
70
80
90
0
200
400
600
800
1000
1200

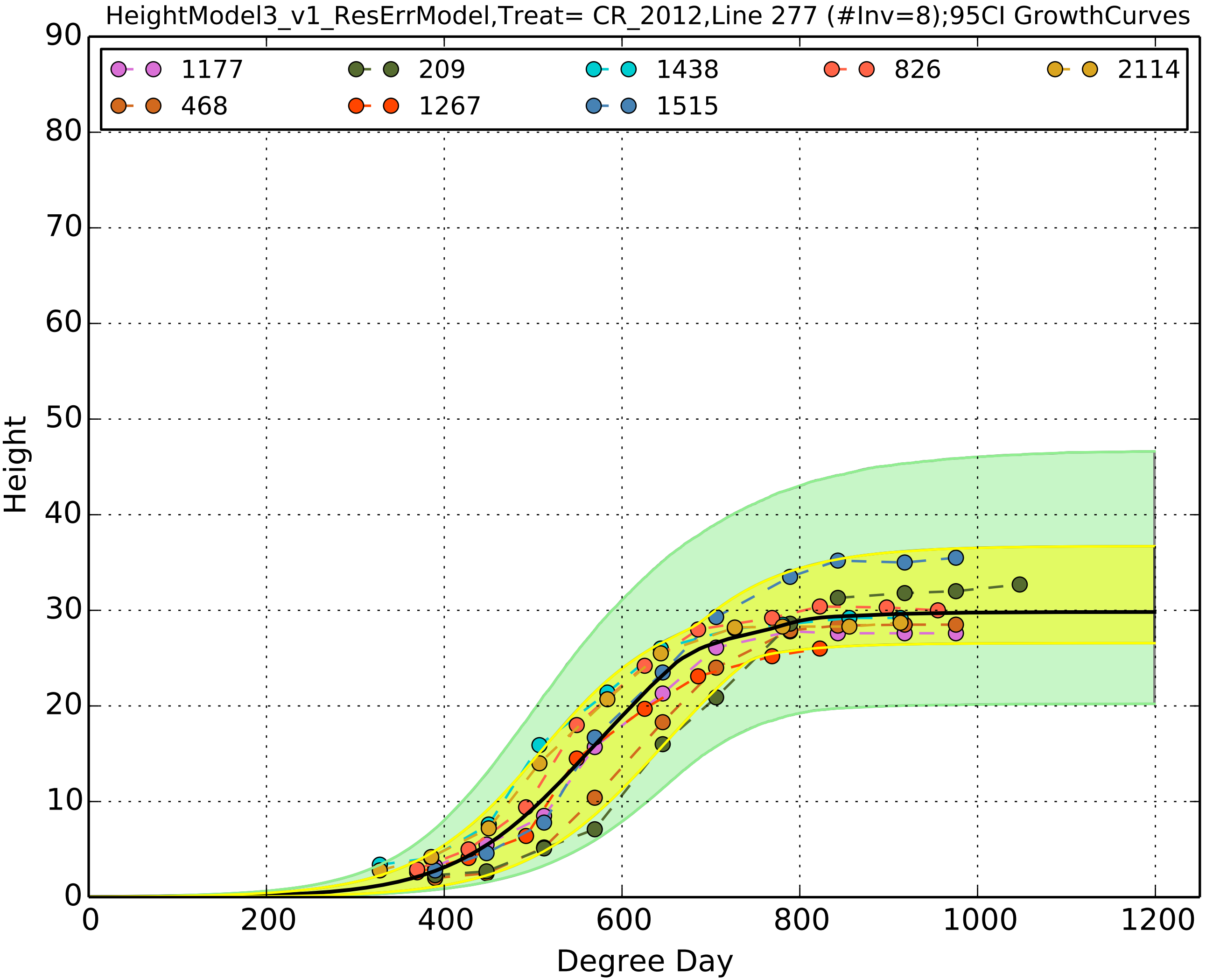

HeightModel3_v1_ResErrModel,Treat= CR_2012,Line 277 (#Inv=8);95CI GrowthCurves
1177
209
1438
826
2114
468
1267
1515
Height
Degree Day
0
10
20
30
40
50
60
70
80
90
0
200
400
600
800
1000
1200

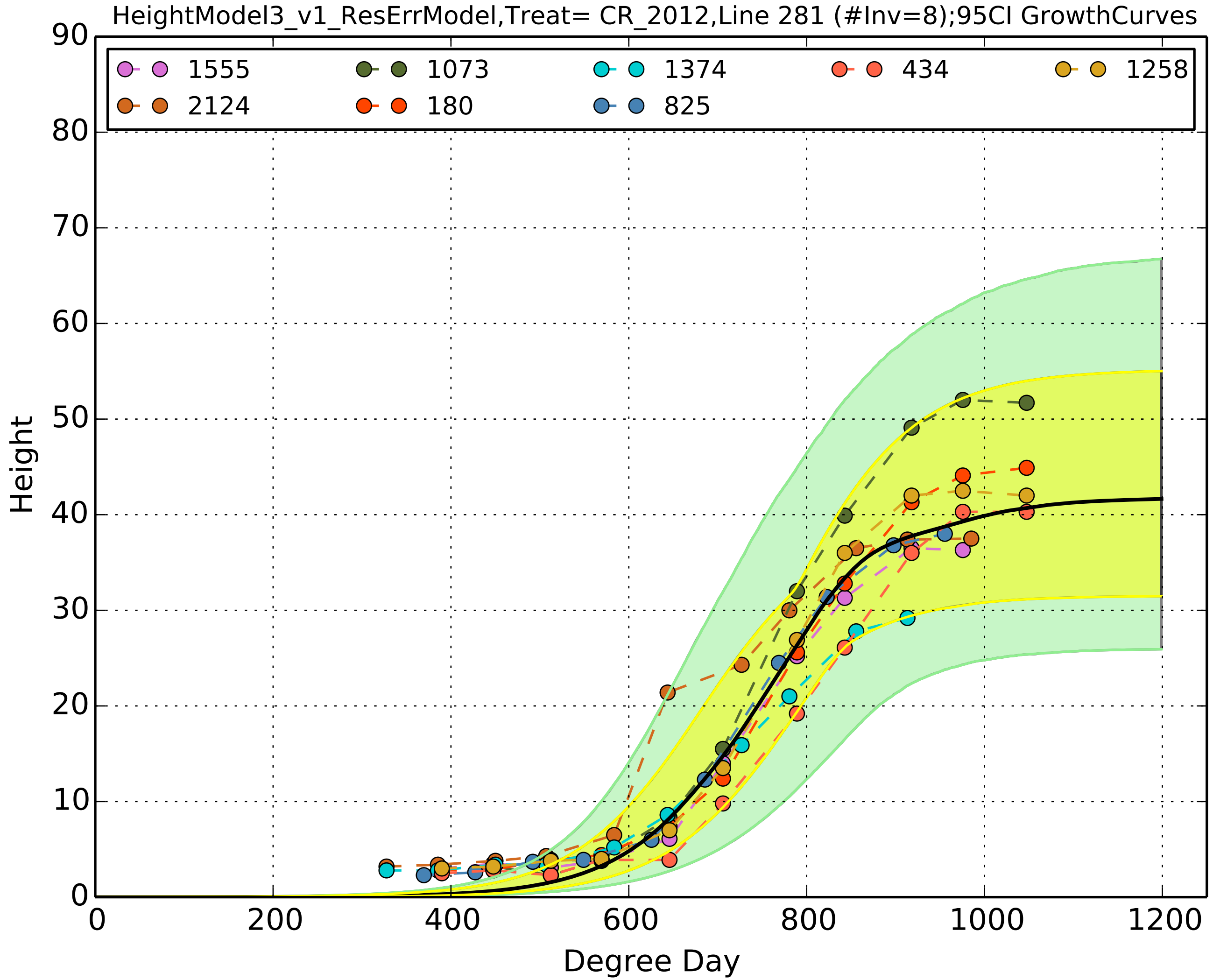

HeightModel3_v1_ResErrModel,Treat= CR_2012,Line 281 (#Inv=8);95CI GrowthCurves
1555
1073
1374
434
1258
2124
180
825
Height
Degree Day
0
10
20
30
40
50
60
70
80
90
0
200
400
600
800
1000
1200

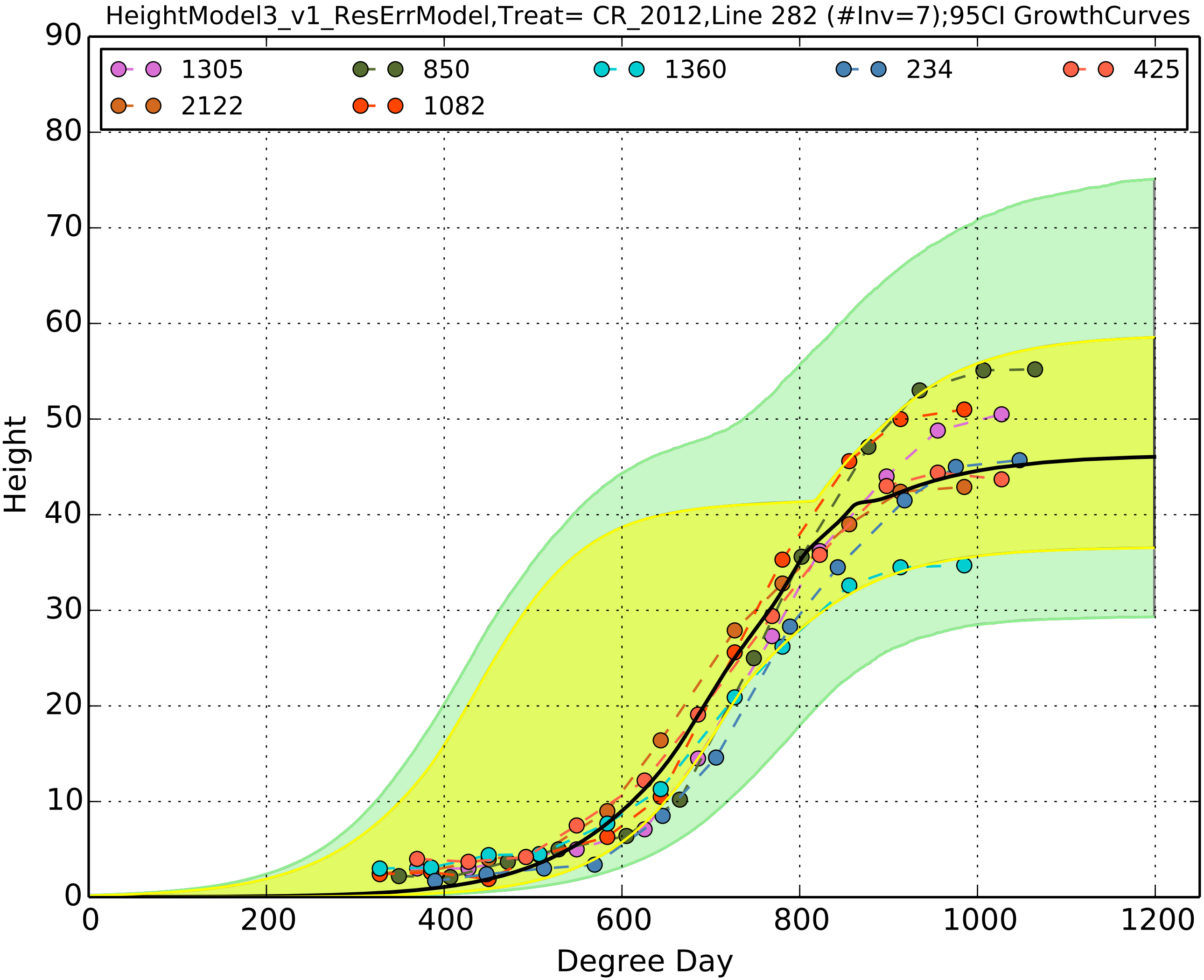

HeightModel3_v1_ResErrModel,Treat= CR_2012,Line 282 (#Inv=7);95CI GrowthCurves
1305
850
1360
234
425
2122
1082
Height
Degree Day
0
10
20
30
40
50
60
70
80
90
0
200
400
600
800
1000
1200

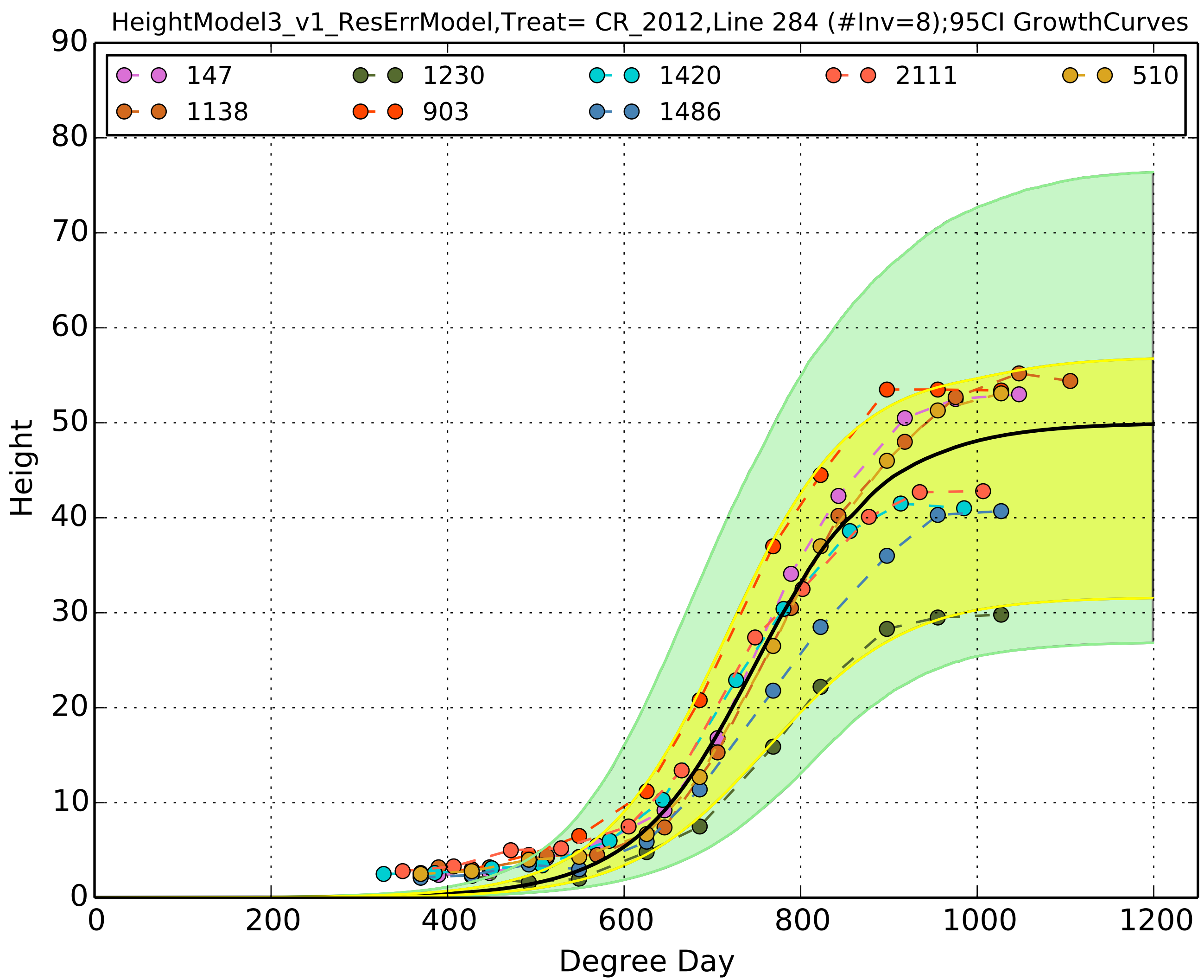

HeightModel3_v1_ResErrModel,Treat= CR_2012,Line 284 (#Inv=8);95CI GrowthCurves
147
1230
1420
2111
510
1138
903
1486
Height
Degree Day
0
10
20
30
40
50
60
70
80
90
0
200
400
600
800
1000
1200

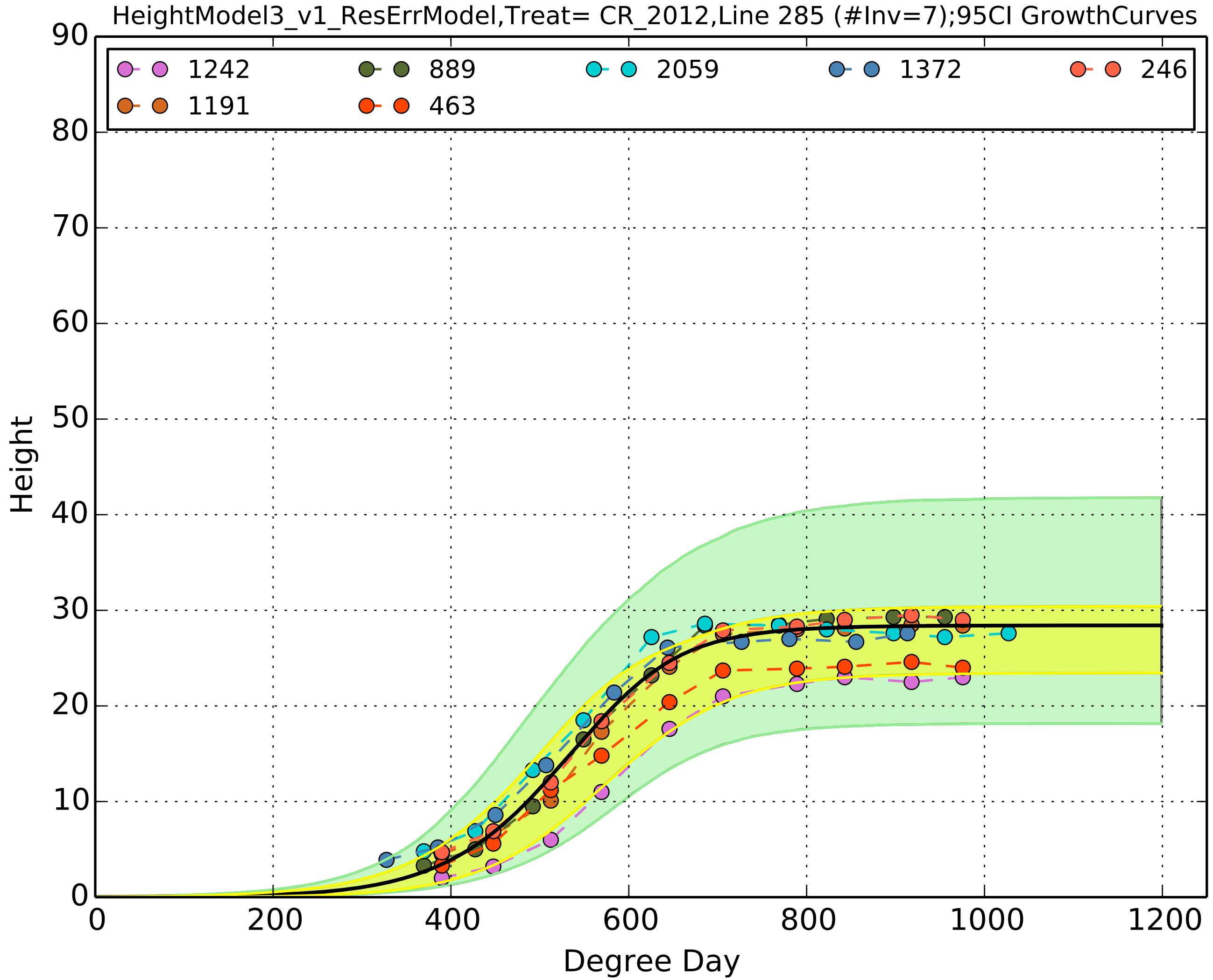
HeightModel3_v1_ResErrModel,Treat= CR_2012,Line 285 (#Inv=7);95CI GrowthCurves
1242
889
2059
1372
246
1191
463
Height
Degree Day
0
10
20
30
40
50
60
70
80
90
0
200
400
600
800
1000
1200

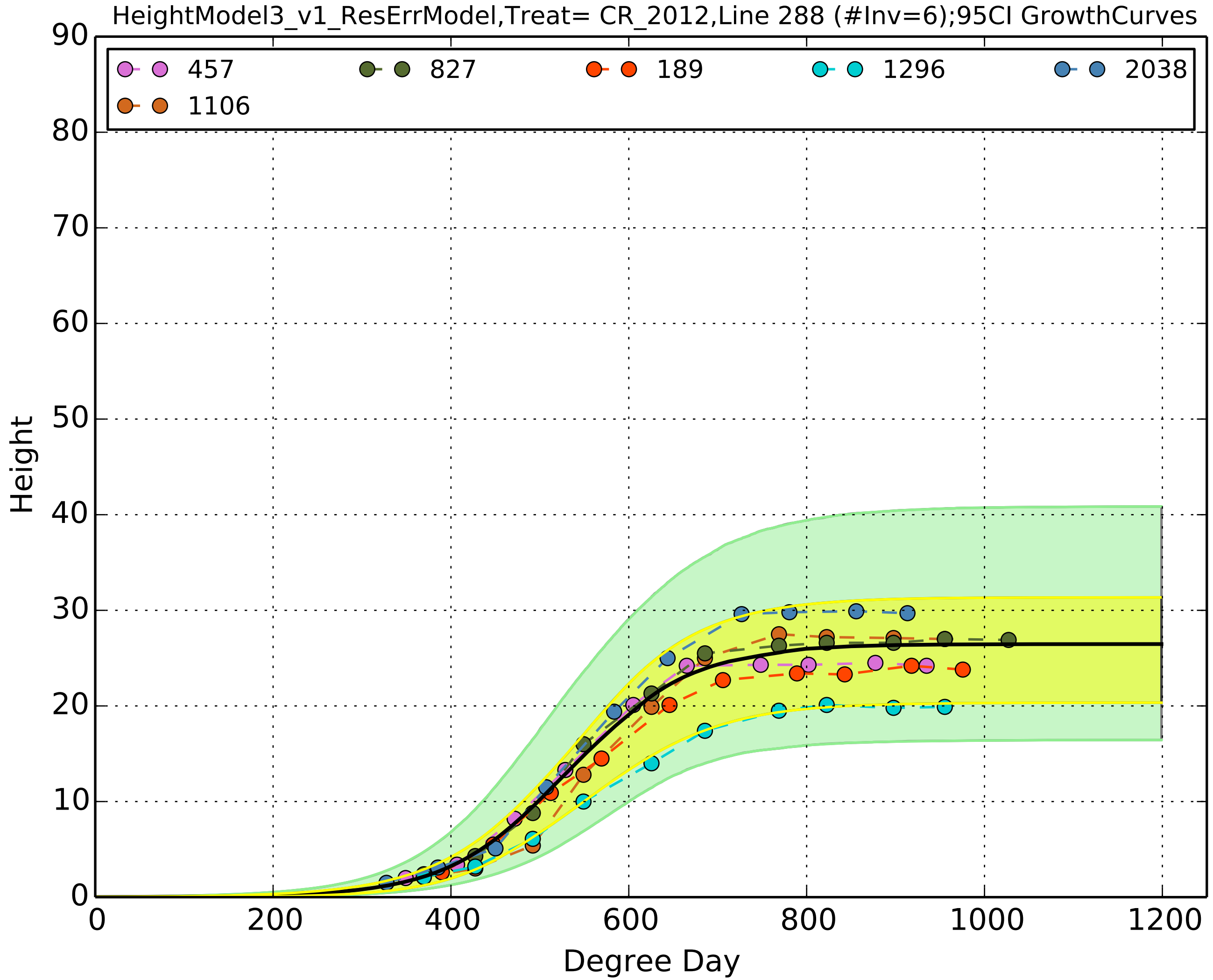

HeightModel3_v1_ResErrModel,Treat= CR_2012,Line 288 (#Inv=6);95CI GrowthCurves
457
827
189
1296
2038
1106
Height
Degree Day
0
10
20
30
40
50
60
70
80
90
0
200
400
600
800
1000
1200

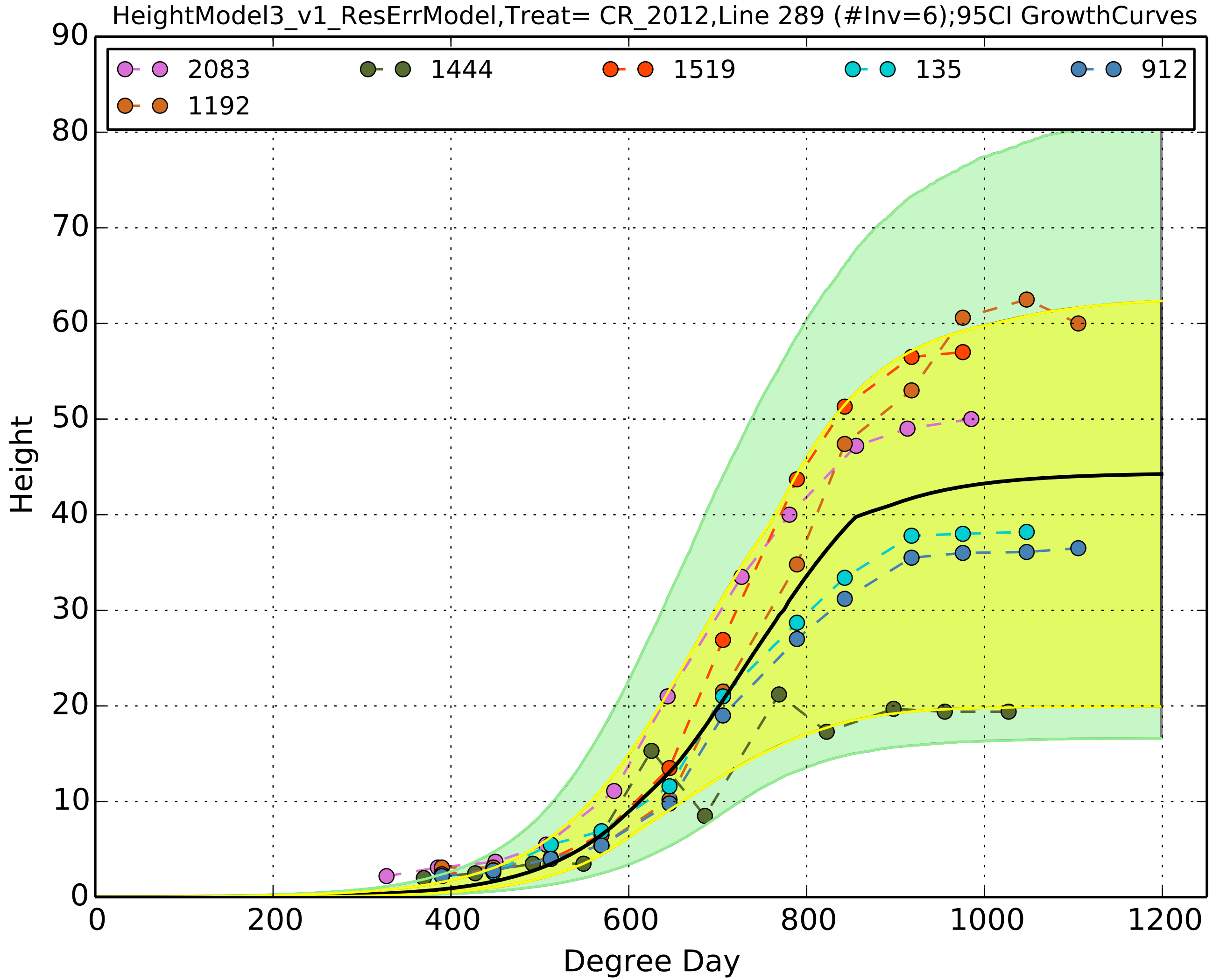

HeightModel3_v1_ResErrModel,Treat= CR_2012,Line 289 (#Inv=6);95CI GrowthCurves
2083
1444
1519
135
912
1192
Height
Degree Day
0
10
20
30
40
50
60
70
80
90
0
200
400
600
800
1000
1200

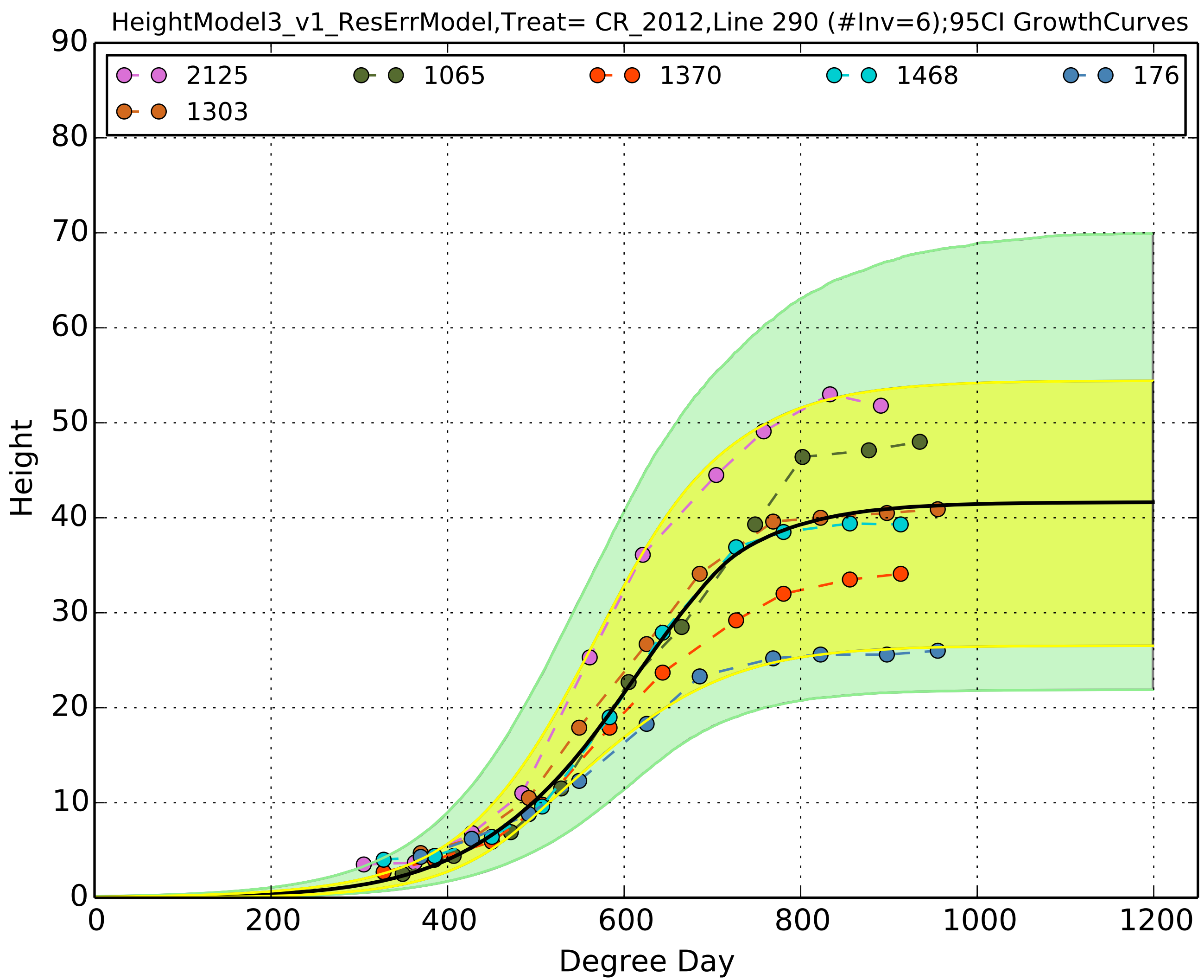

HeightModel3_v1_ResErrModel,Treat= CR_2012,Line 290 (#Inv=6);95CI GrowthCurves
2125
1065
1370
1468
176
1303
Height
Degree Day
0
10
20
30
40
50
60
70
80
90
0
200
400
600
800
1000
1200

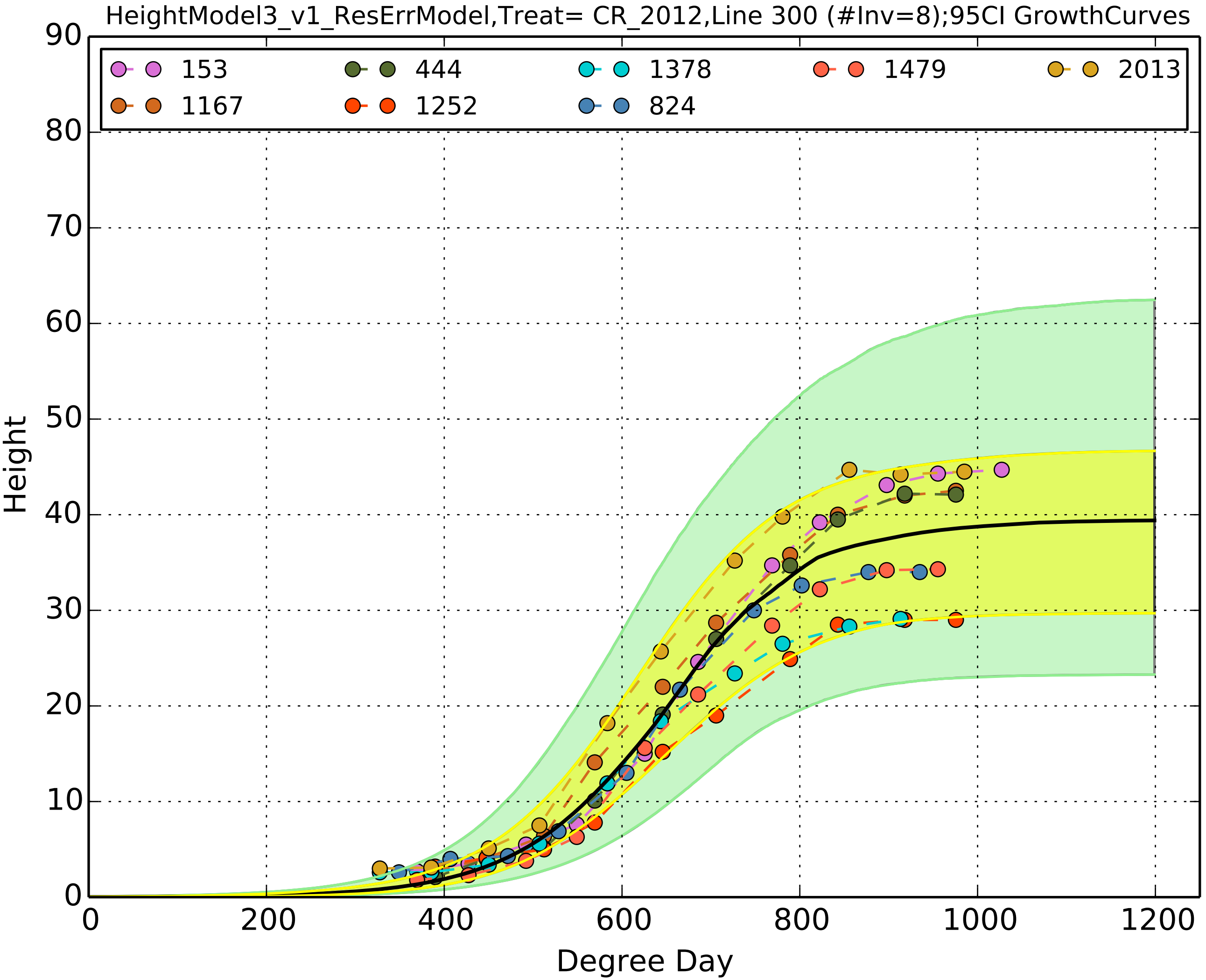

HeightModel3_v1_ResErrModel,Treat= CR_2012,Line 300 (#Inv=8);95CI GrowthCurves
153
444
1378
1479
2013
1167
1252
824
Height
Degree Day
0
10
20
30
40
50
60
70
80
90
0
200
400
600
800
1000
1200

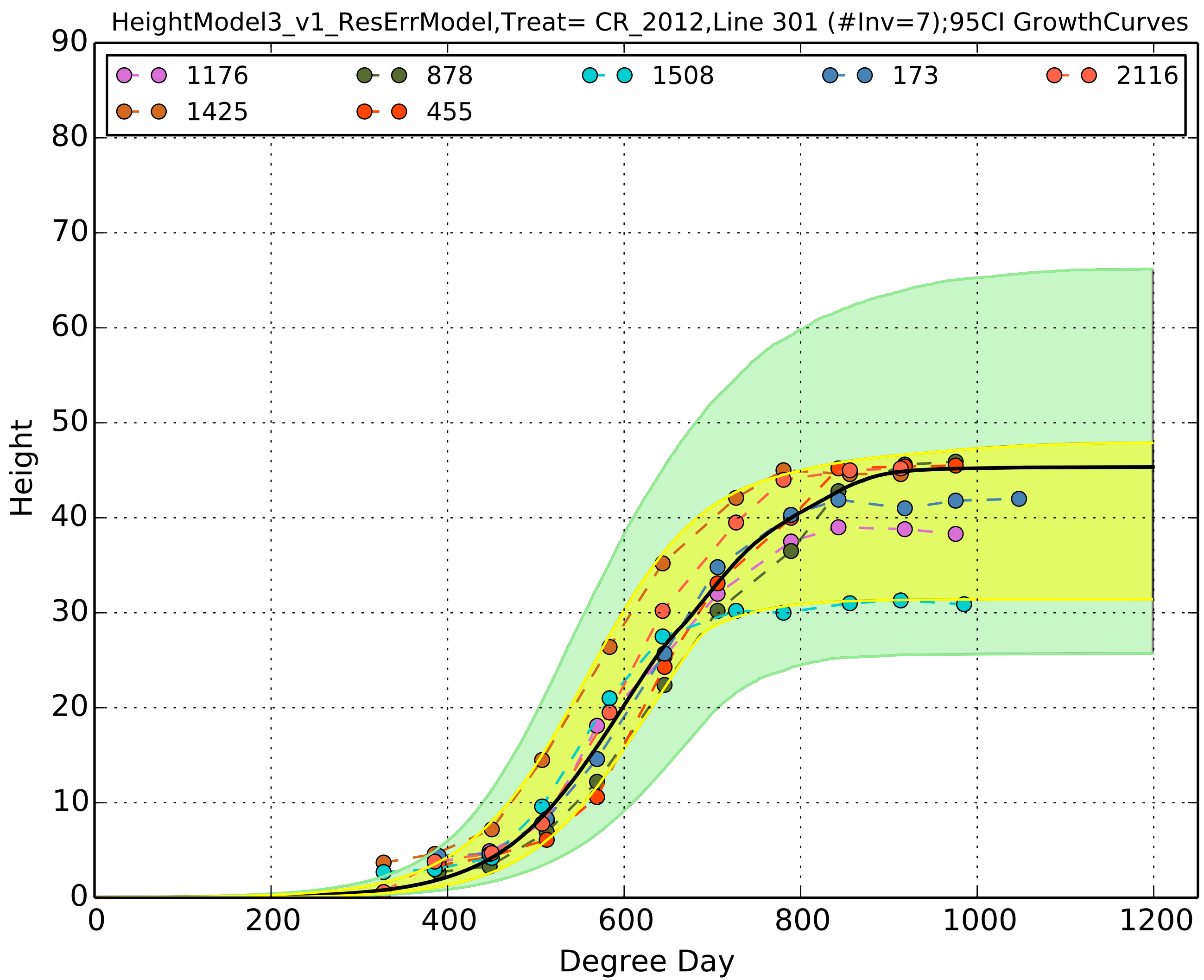
HeightModel3_v1_ResErrModel,Treat= CR_2012,Line 301 (#Inv=7);95CI GrowthCurves
1176
878
1508
173
2116
1425
455
Height
Degree Day
0
10
20
30
40
50
60
70
80
90
0
200
400
600
800
1000
1200

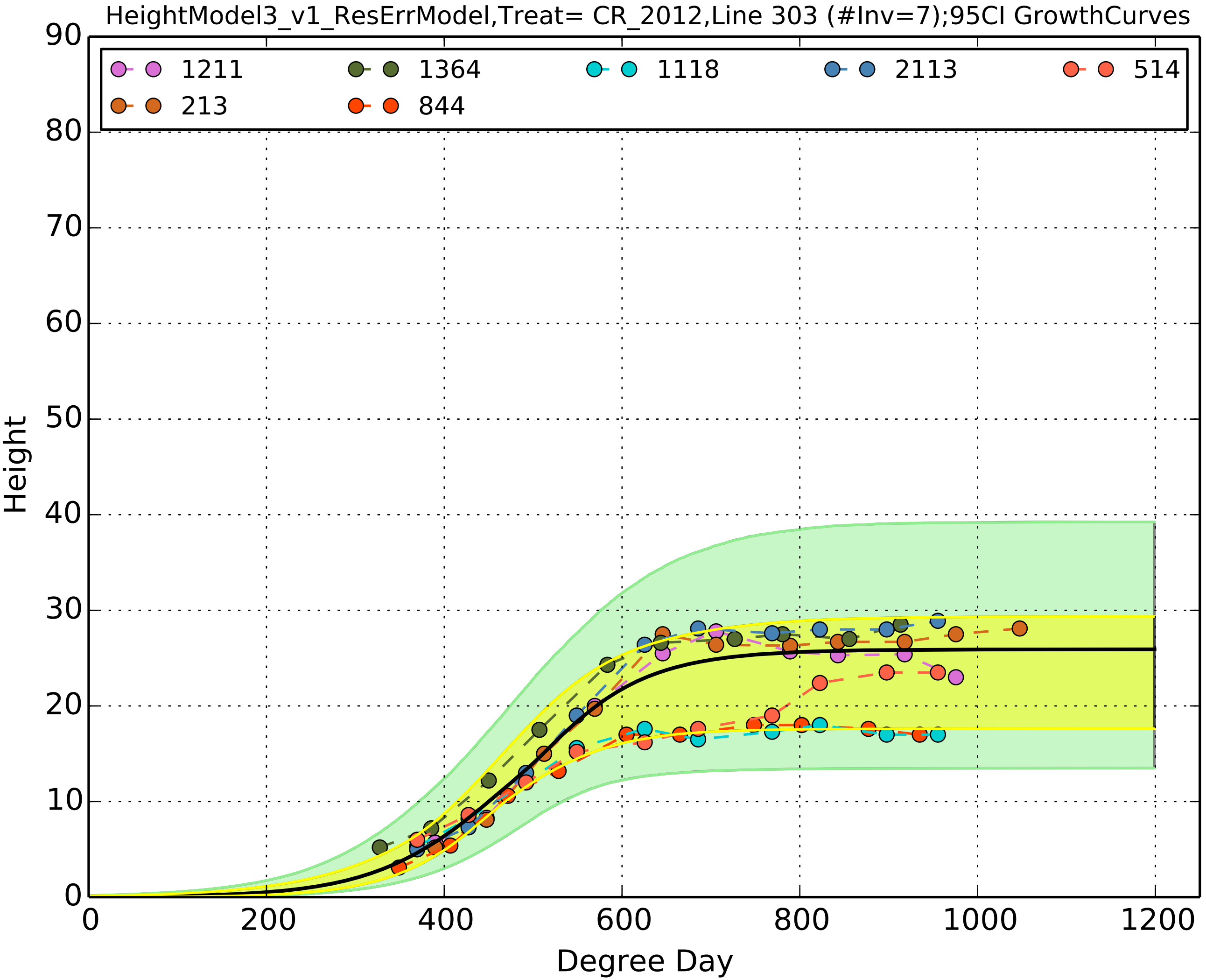
HeightModel3_v1_ResErrModel,Treat= CR_2012,Line 303 (#Inv=7);95CI GrowthCurves
1211
1364
1118
2113
514
213
844
Height
Degree Day
0
10
20
30
40
50
60
70
80
90
0
200
400
600
800
1000
1200

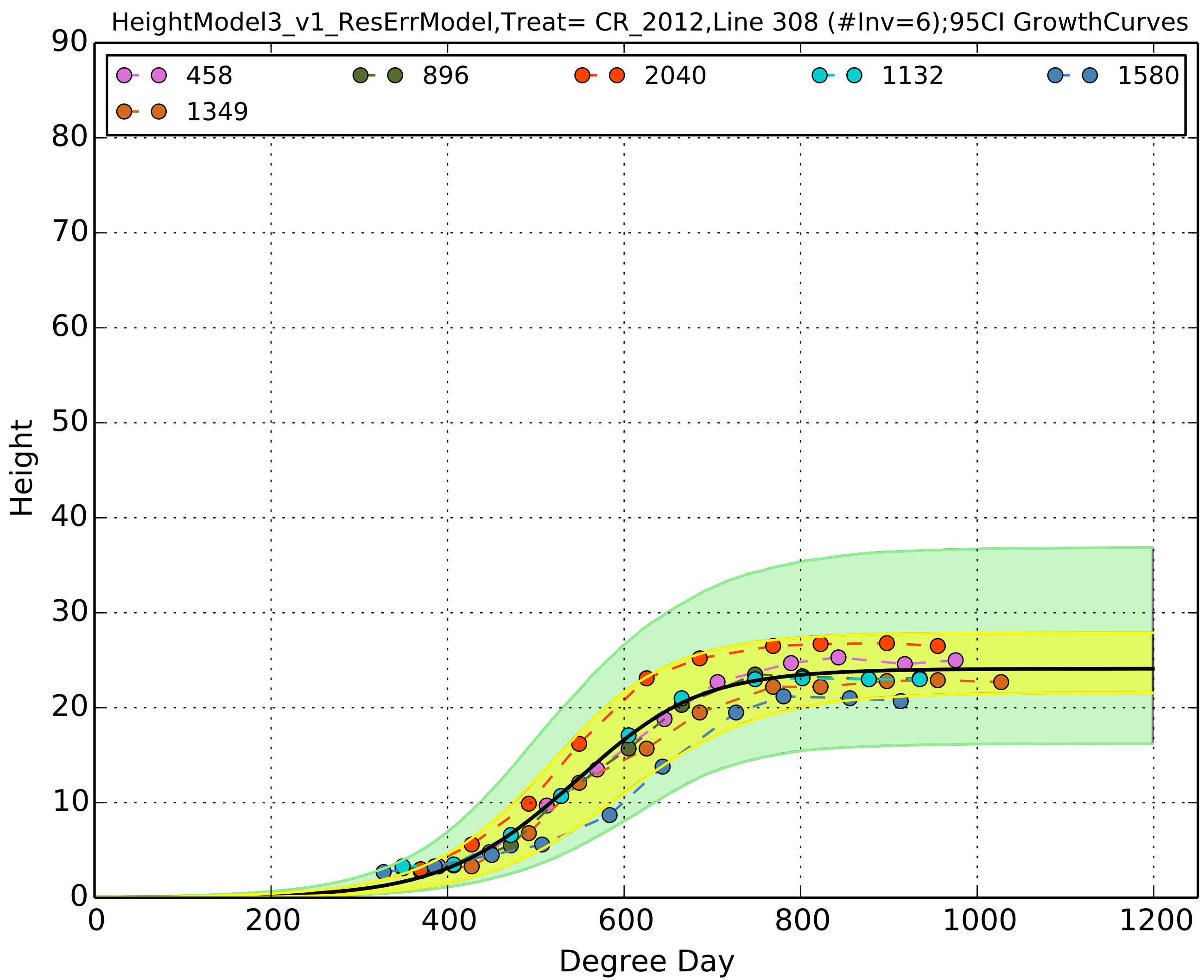

HeightModel3_v1_ResErrModel,Treat= CR_2012,Line 308 (#Inv=6);95CI GrowthCurves
458
896
2040
1132
1580
1349
Height
Degree Day
0
10
20
30
40
50
60
70
80
90
0
200
400
600
800
1000
1200

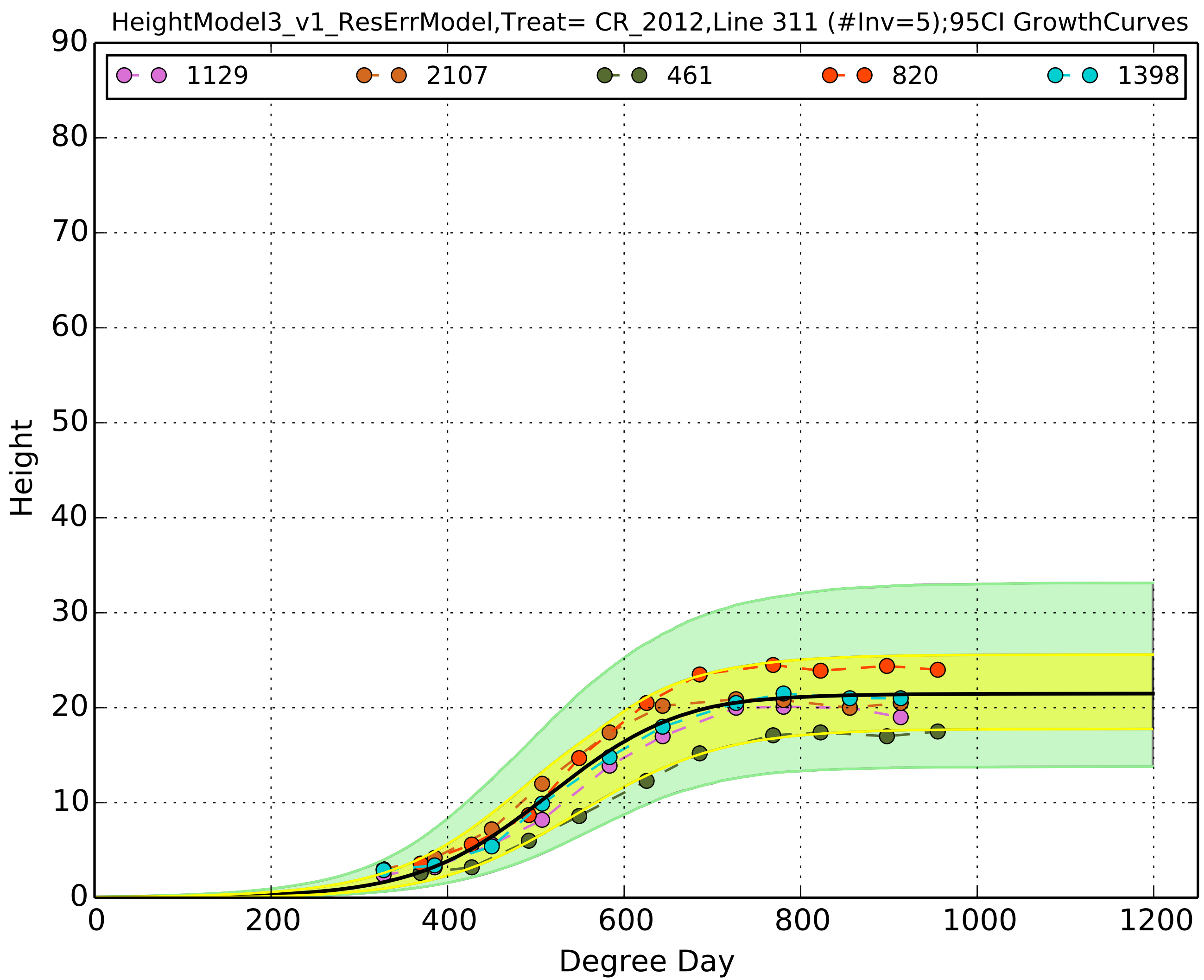

HeightModel3_v1_ResErrModel,Treat= CR_2012,Line 311 (#Inv=5);95CI GrowthCurves
1129
2107
461
820
1398
Height
Degree Day
0
10
20
30
40
50
60
70
80
90
0
200
400
600
800
1000
1200

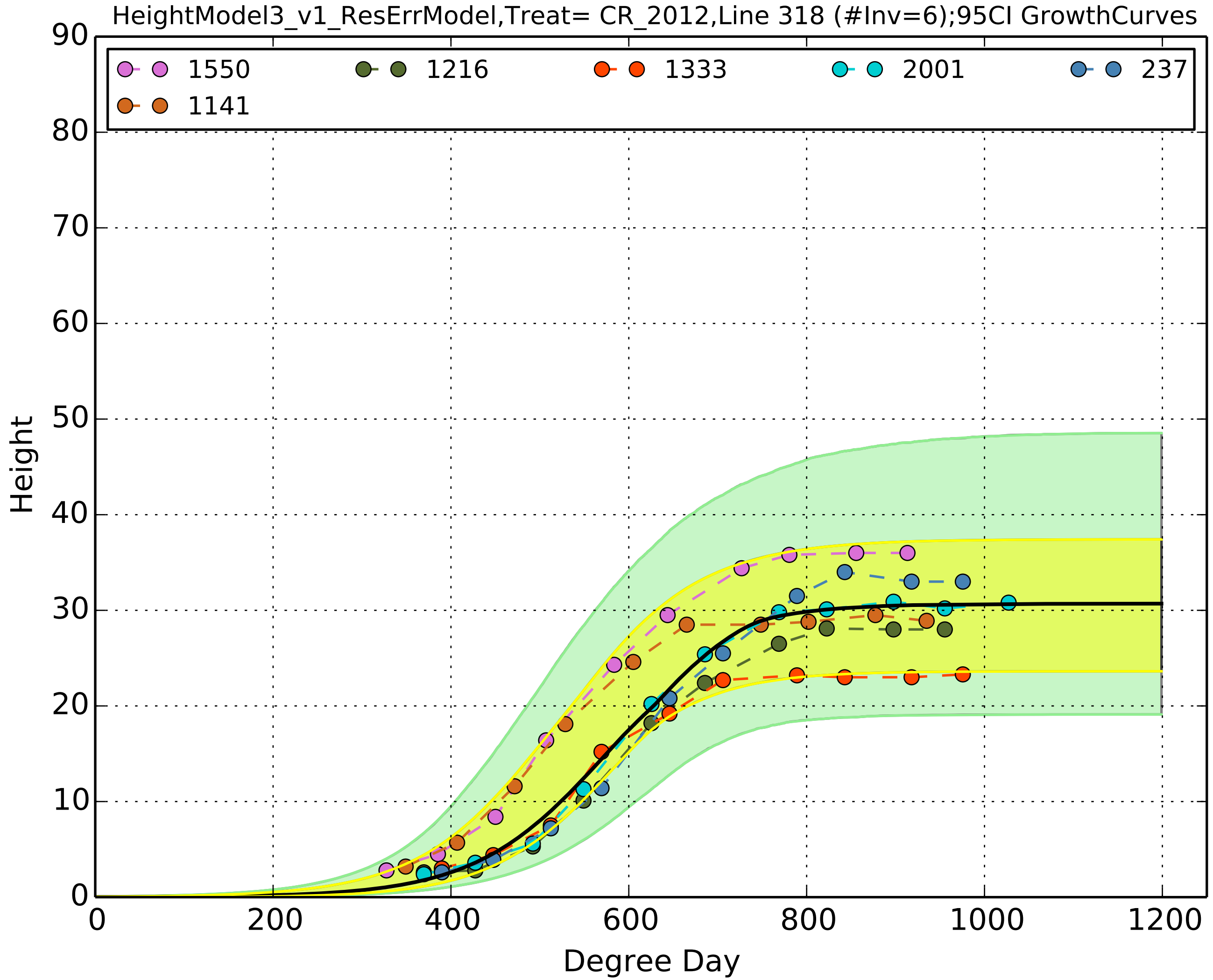
HeightModel3_v1_ResErrModel,Treat= CR_2012,Line 318 (#Inv=6);95CI GrowthCurves
1550
1216
1333
2001
237
1141
Height
Degree Day
0
10
20
30
40
50
60
70
80
90
0
200
400
600
800
1000
1200

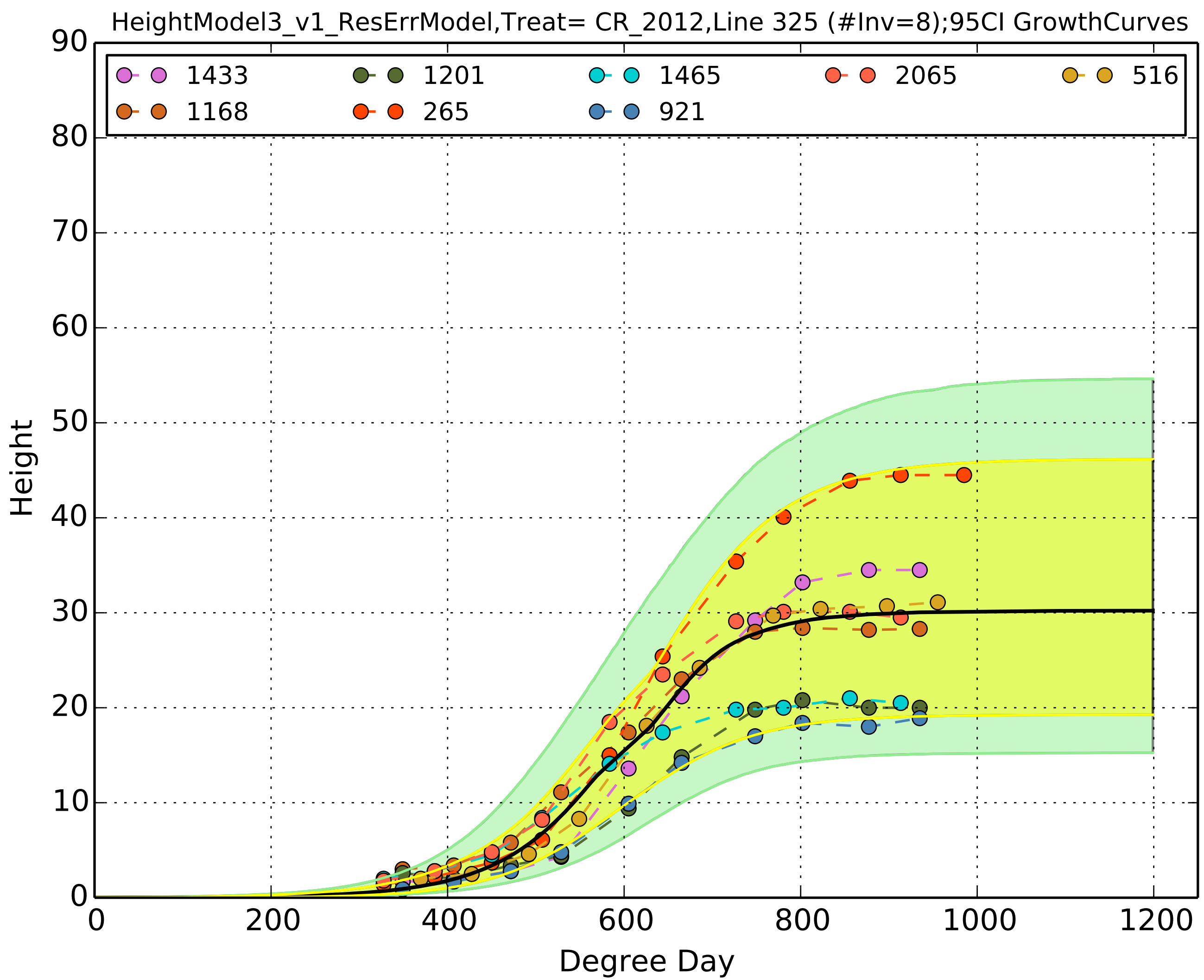

HeightModel3_v1_ResErrModel,Treat= CR_2012,Line 325 (#Inv=8);95CI GrowthCurves
1433
1201
1465
2065
516
1168
265
921
Height
Degree Day
0
10
20
30
40
50
60
70
80
90
0
200
400
600
800
1000
1200

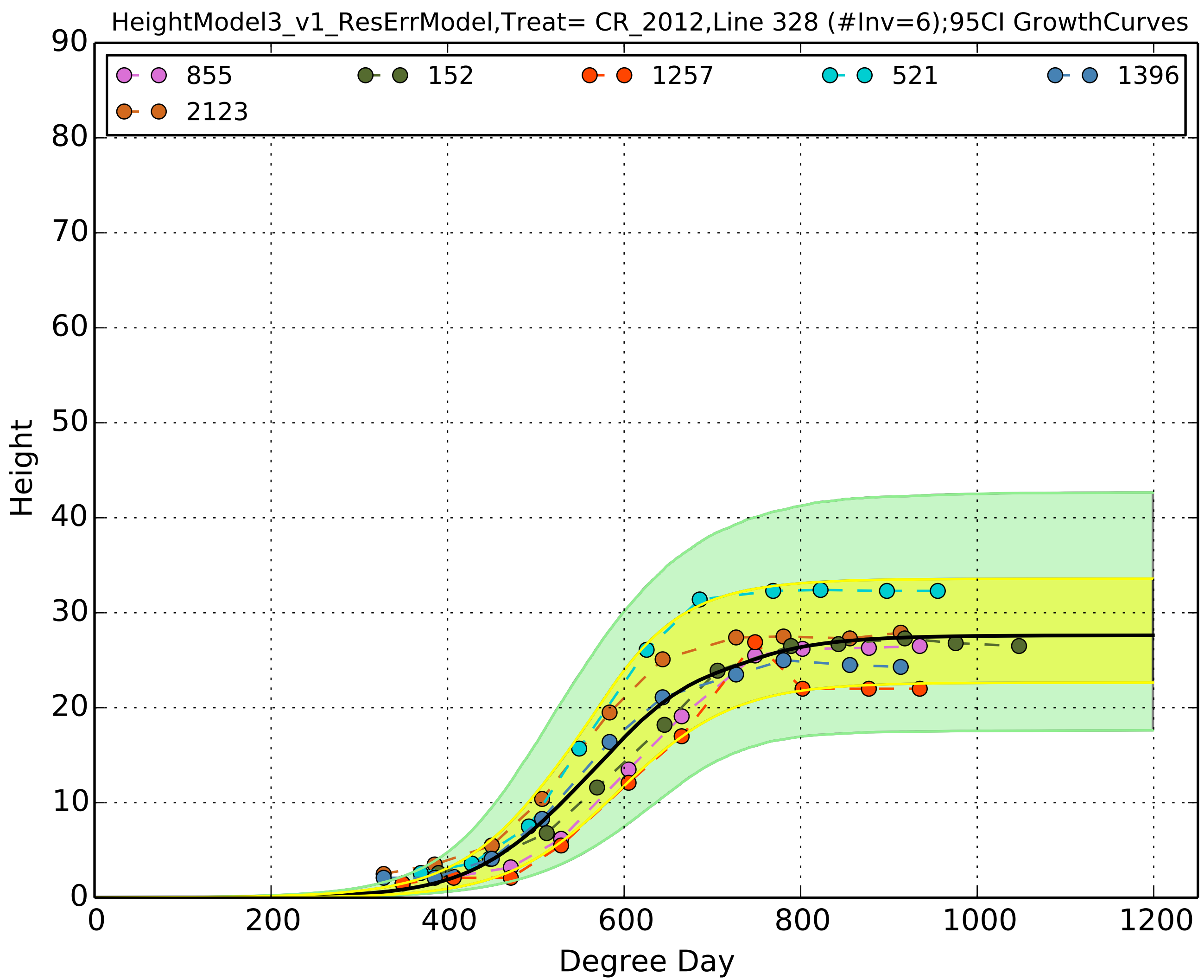

HeightModel3_v1_ResErrModel,Treat= CR_2012,Line 328 (#Inv=6);95CI GrowthCurves
855
152
1257
521
1396
2123
Height
Degree Day
0
10
20
30
40
50
60
70
80
90
0
200
400
600
800
1000
1200

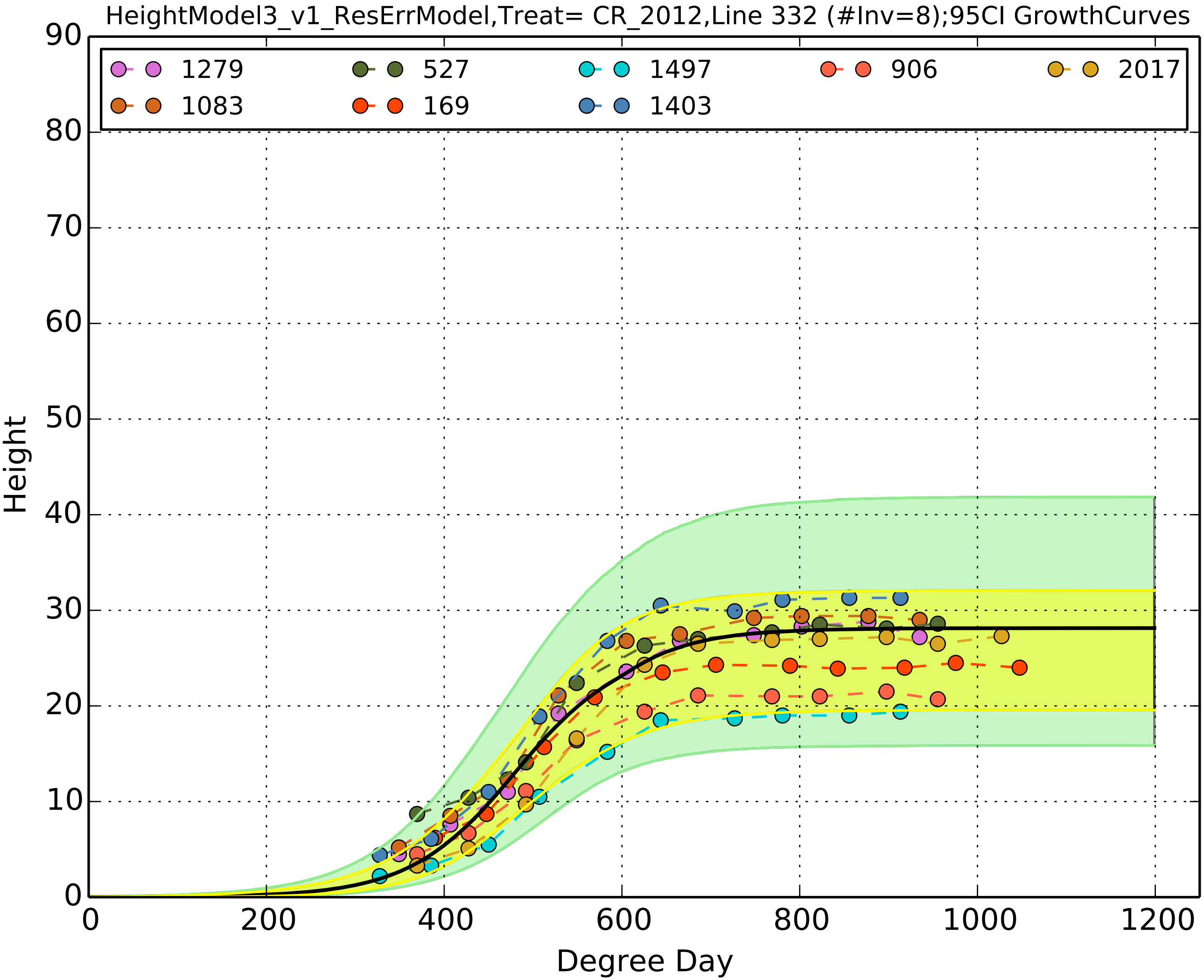

HeightModel3_v1_ResErrModel,Treat= CR_2012,Line 332 (#Inv=8);95CI GrowthCurves
1279
527
1497
906
2017
1083
169
1403
Height
Degree Day
0
10
20
30
40
50
60
70
80
90
0
200
400
600
800
1000
1200

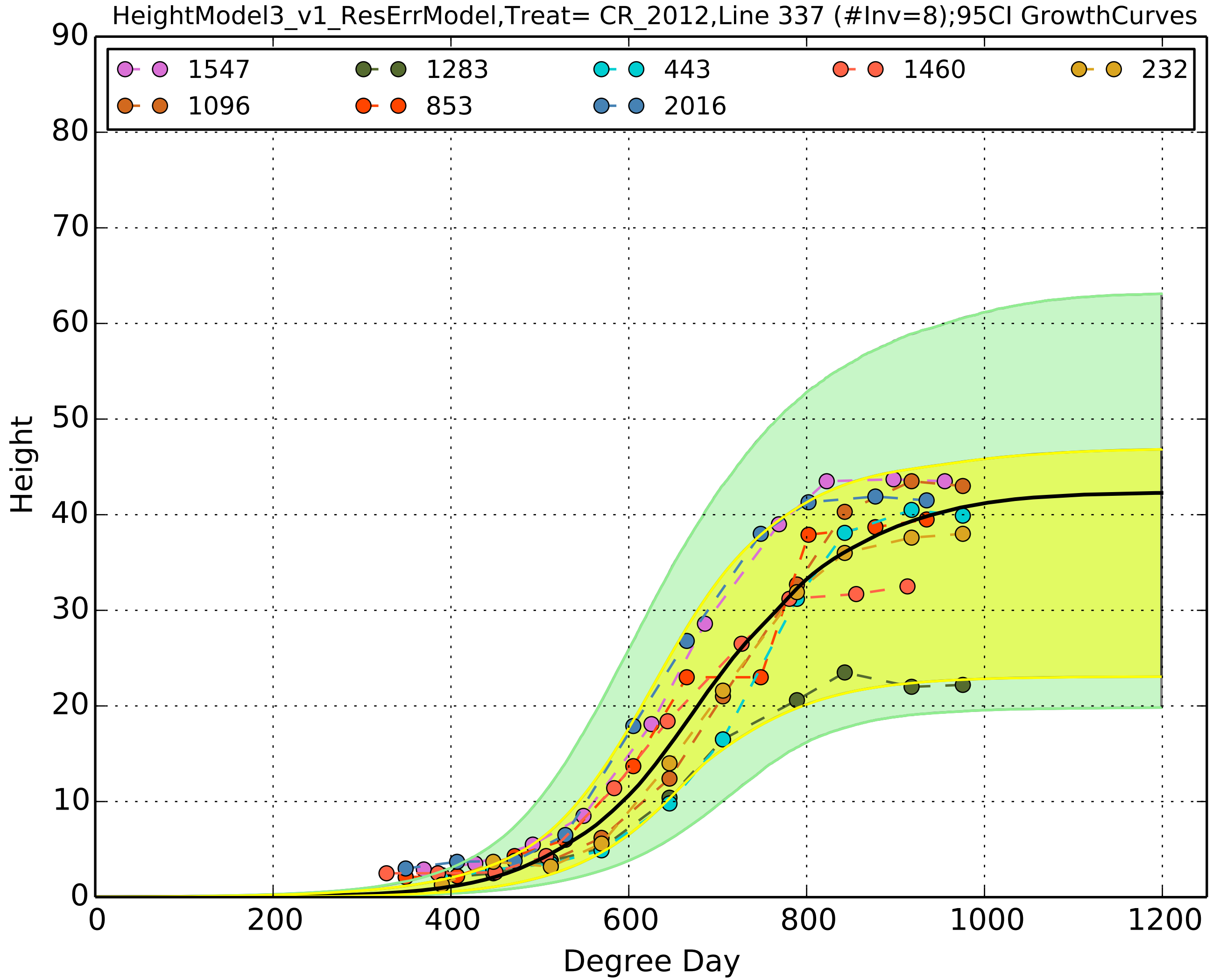

HeightModel3_v1_ResErrModel,Treat= CR_2012,Line 337 (#Inv=8);95CI GrowthCurves
1547
1283
443
1460
232
1096
853
2016
Height
Degree Day
0
10
20
30
40
50
60
70
80
90
0
200
400
600
800
1000
1200

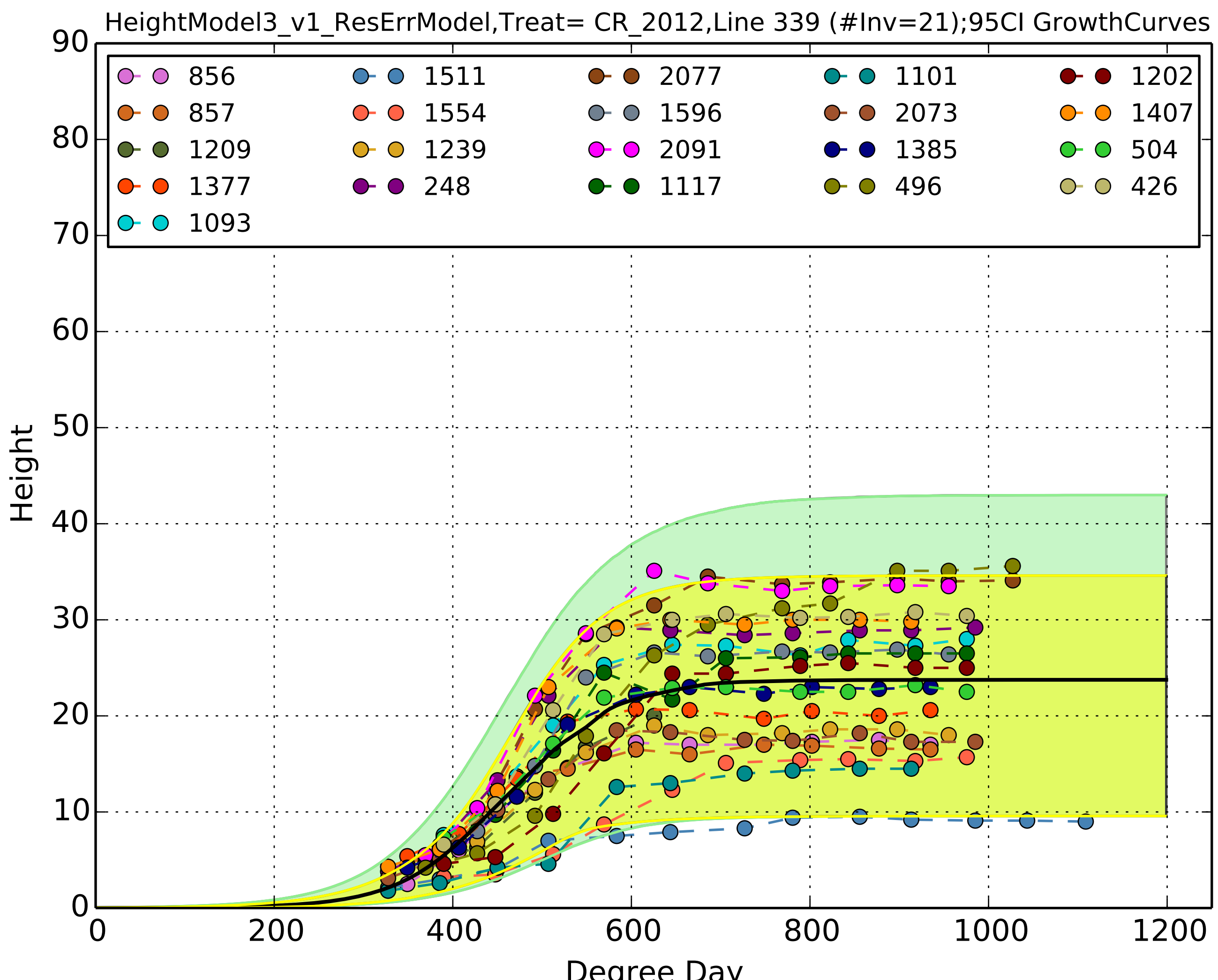
HeightModel3_v1_ResErrModel,Treat= CR_2012,Line 339 (#Inv=21);95CI GrowthCurves
856
857
1209
1377
1093
1511
1554
1239
248
2077
1596
2091
1117
1101
2073
1385
496
1202
1407
504
426
Height
Degree Day
0
10
20
30
40
50
60
70
80
90
0
200
400
600
800
1000
1200

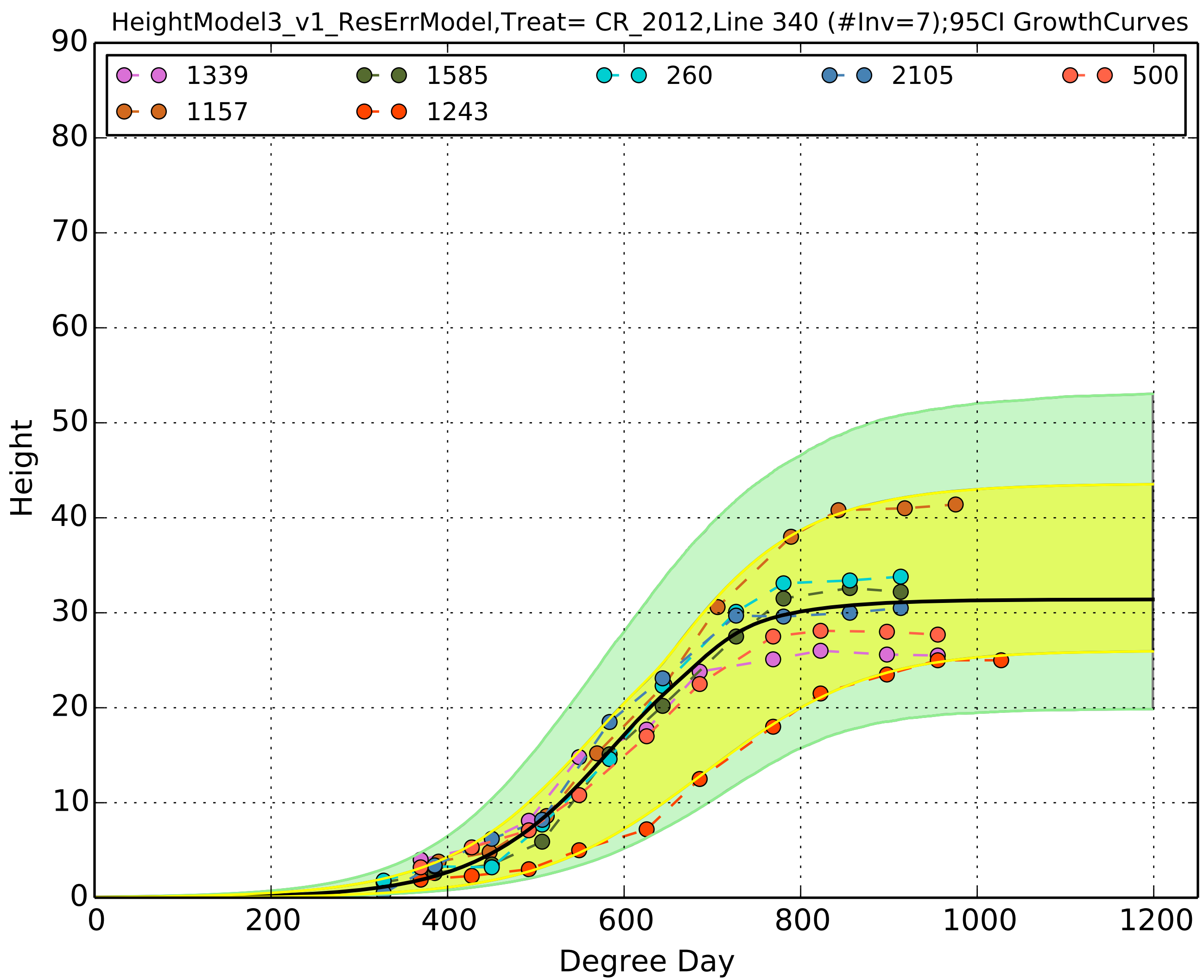

HeightModel3_v1_ResErrModel,Treat= CR_2012,Line 340 (#Inv=7);95CI GrowthCurves
1339
1585
260
2105
500
1157
1243
Height
Degree Day
0
10
20
30
40
50
60
70
80
90
0
200
400
600
800
1000
1200

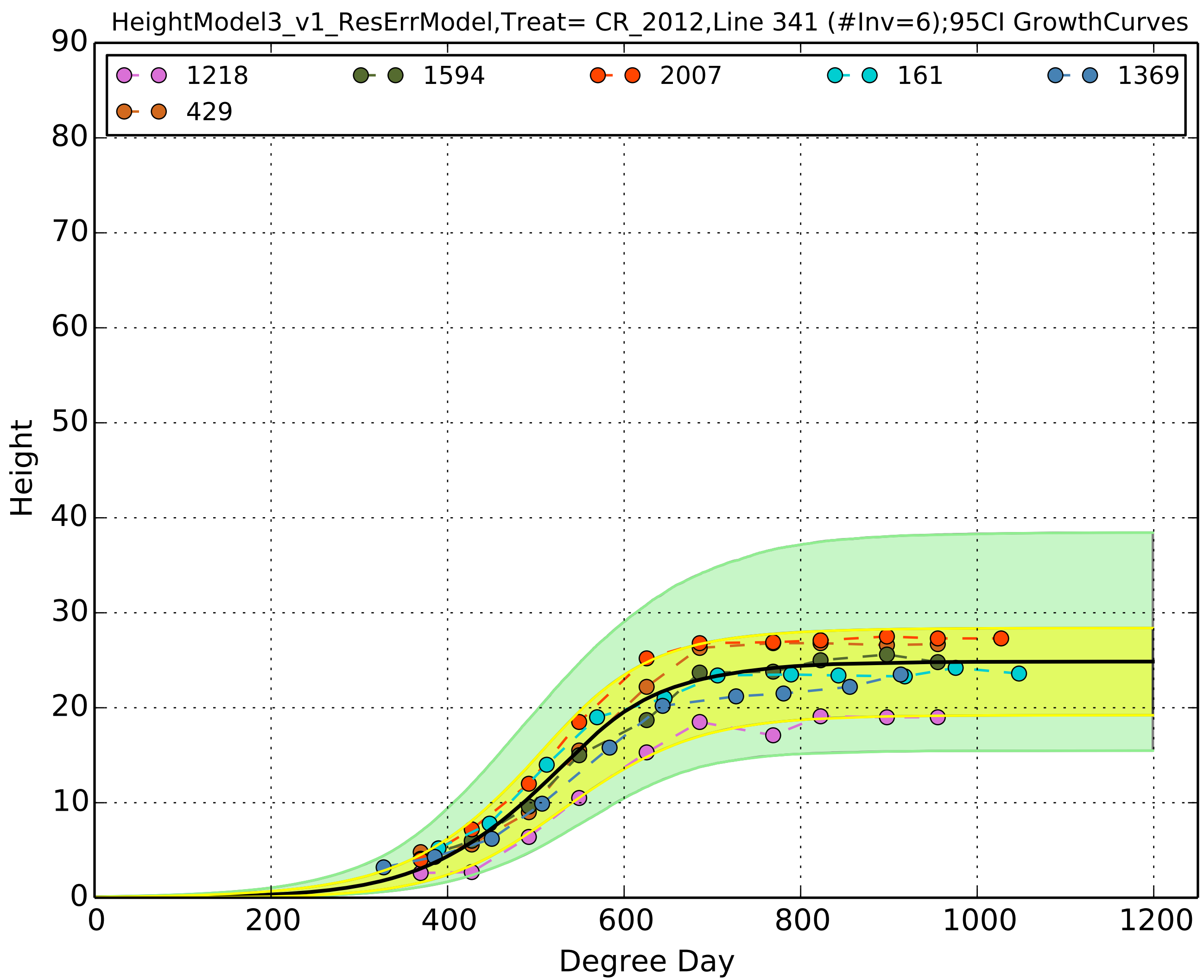

HeightModel3_v1_ResErrModel,Treat= CR_2012,Line 341 (#Inv=6);95CI GrowthCurves
1218
1594
2007
161
1369
429
Height
Degree Day
0
10
20
30
40
50
60
70
80
90
0
200
400
600
800
1000
1200

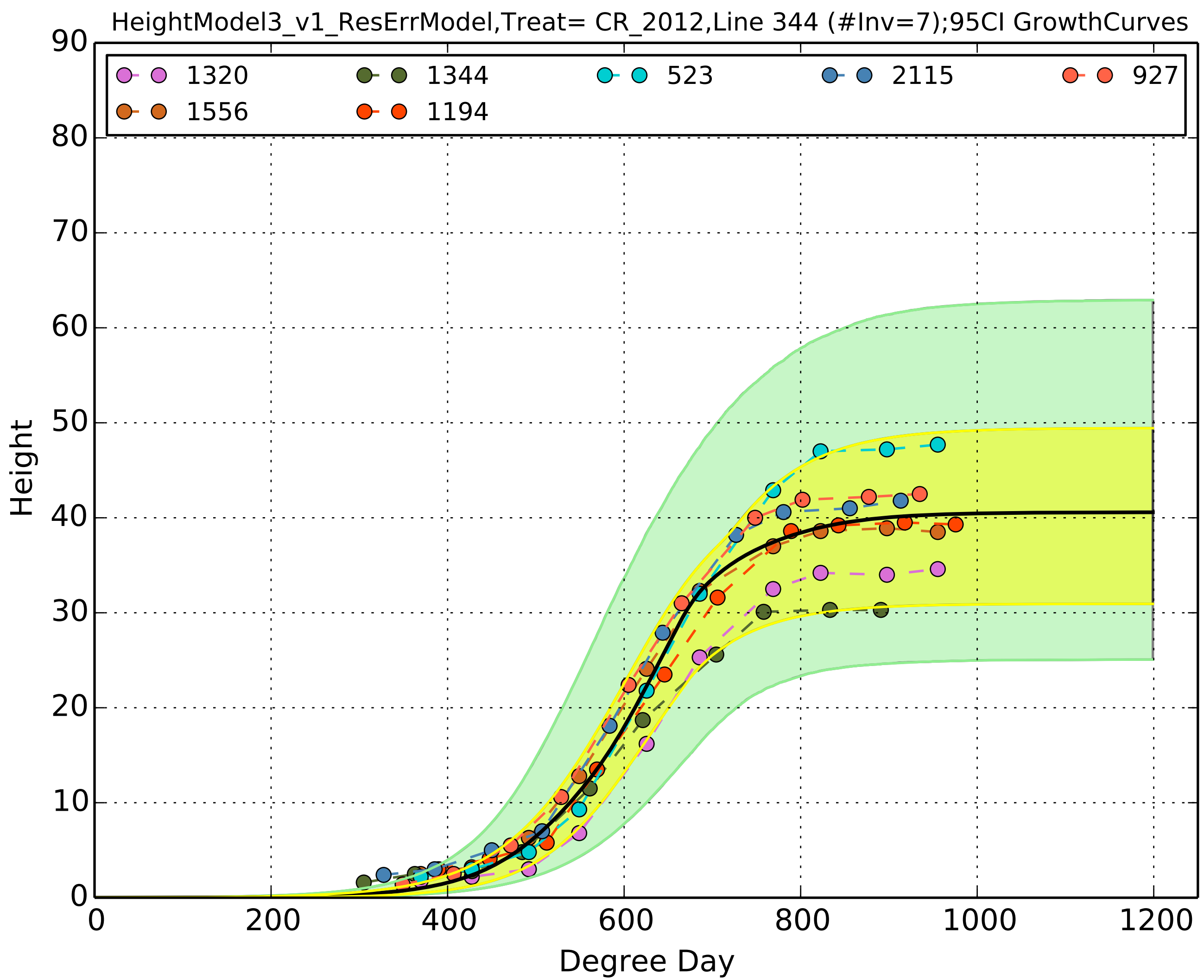

HeightModel3_v1_ResErrModel,Treat= CR_2012,Line 344 (#Inv=7);95CI GrowthCurves
1320
1344
523
2115
927
1556
1194
Height
Degree Day
0
10
20
30
40
50
60
70
80
90
0
200
400
600
800
1000
1200

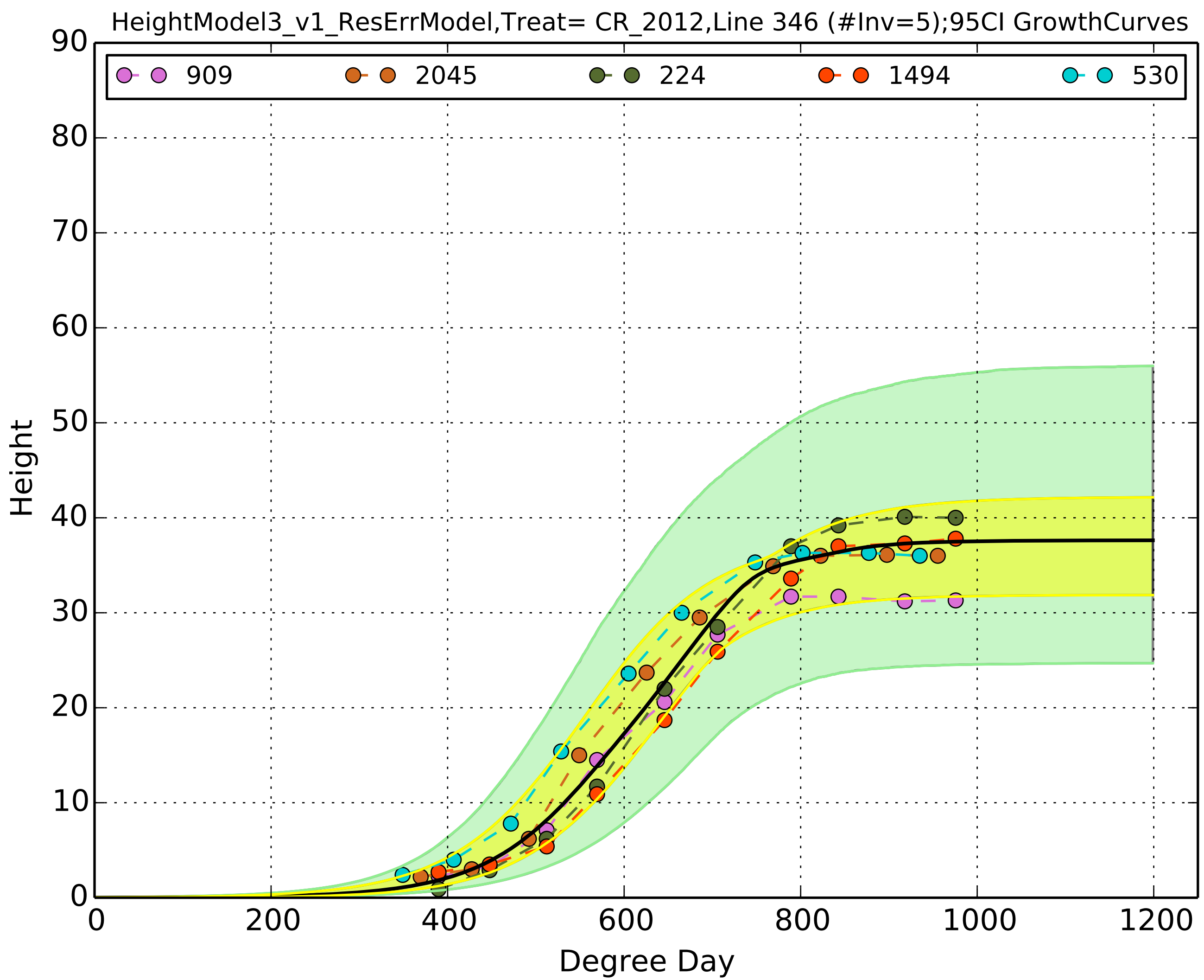
HeightModel3_v1_ResErrModel,Treat= CR_2012,Line 346 (#Inv=5);95CI GrowthCurves
909
2045
224
1494
530
Height
Degree Day
0
10
20
30
40
50
60
70
80
90
0
200
400
600
800
1000
1200

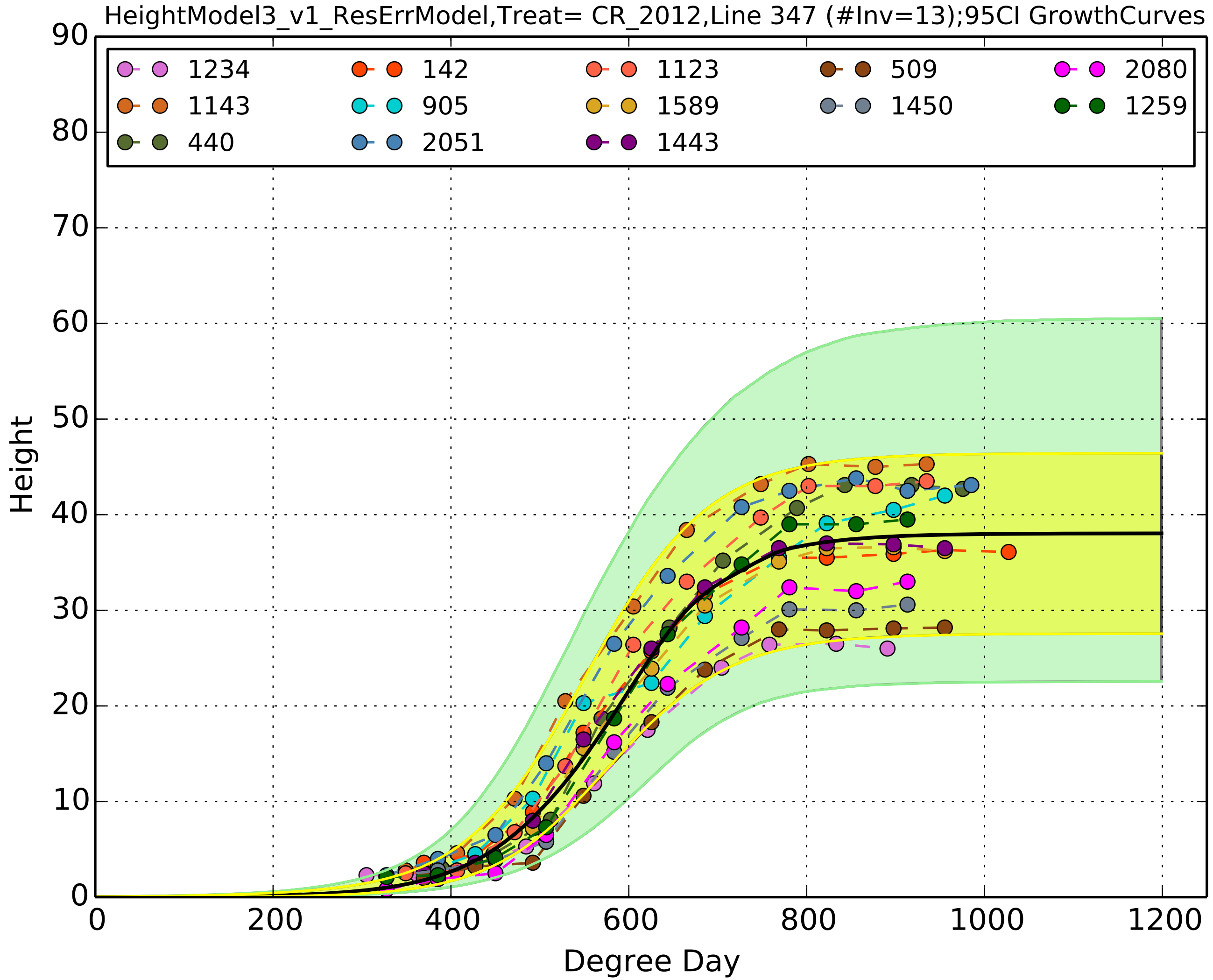
HeightModel3_v1_ResErrModel,Treat= CR_2012,Line 347 (#Inv=13);95CI GrowthCurves
1234
142
1123
509
2080
1143
905
1589
1450
1259
440
2051
1443
Height
Degree Day
0
10
20
30
40
50
60
70
80
90
0
200
400
600
800
1000
1200

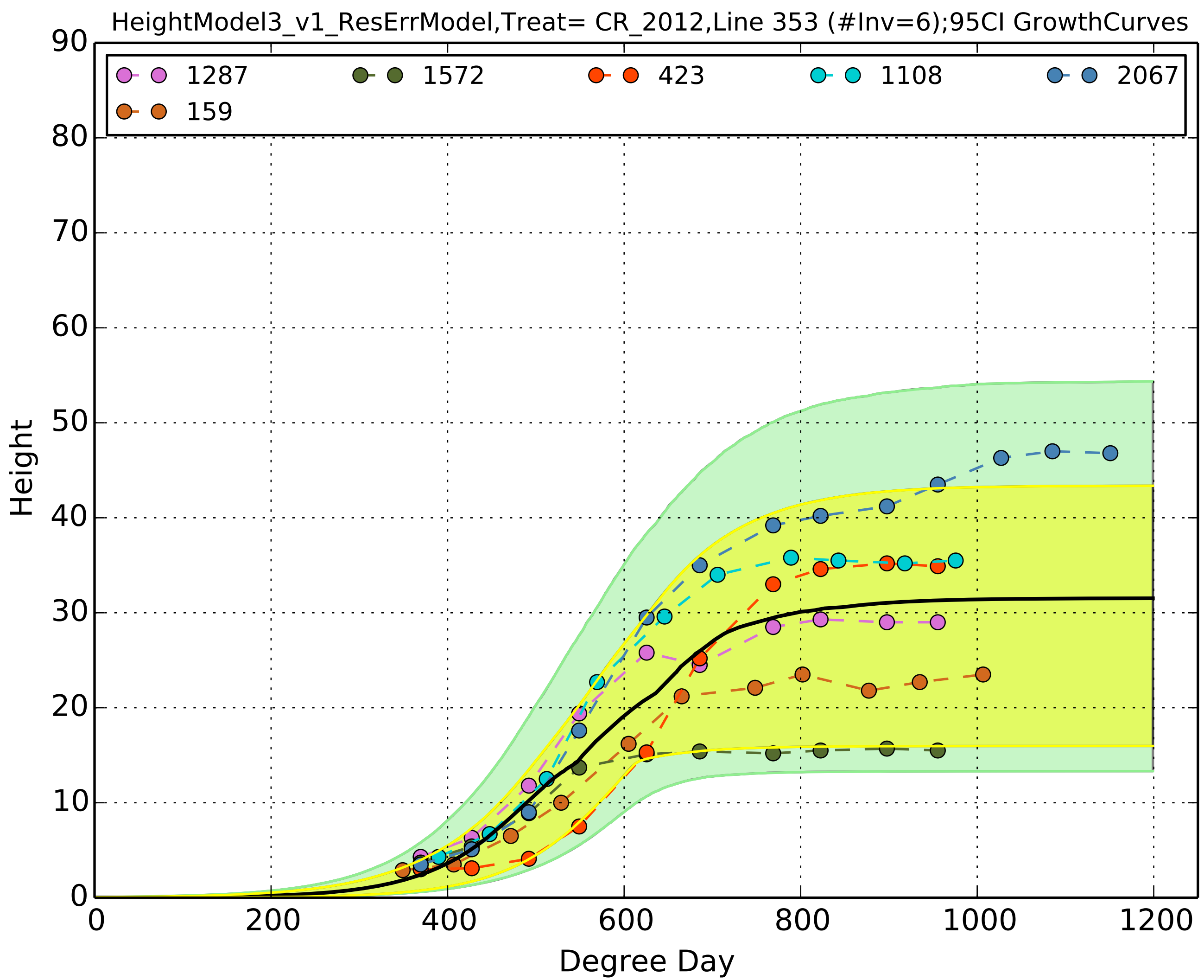

HeightModel3_v1_ResErrModel,Treat= CR_2012,Line 353 (#Inv=6);95CI GrowthCurves
1287
1572
423
1108
2067
159
Height
Degree Day
0
10
20
30
40
50
60
70
80
90
0
200
400
600
800
1000
1200

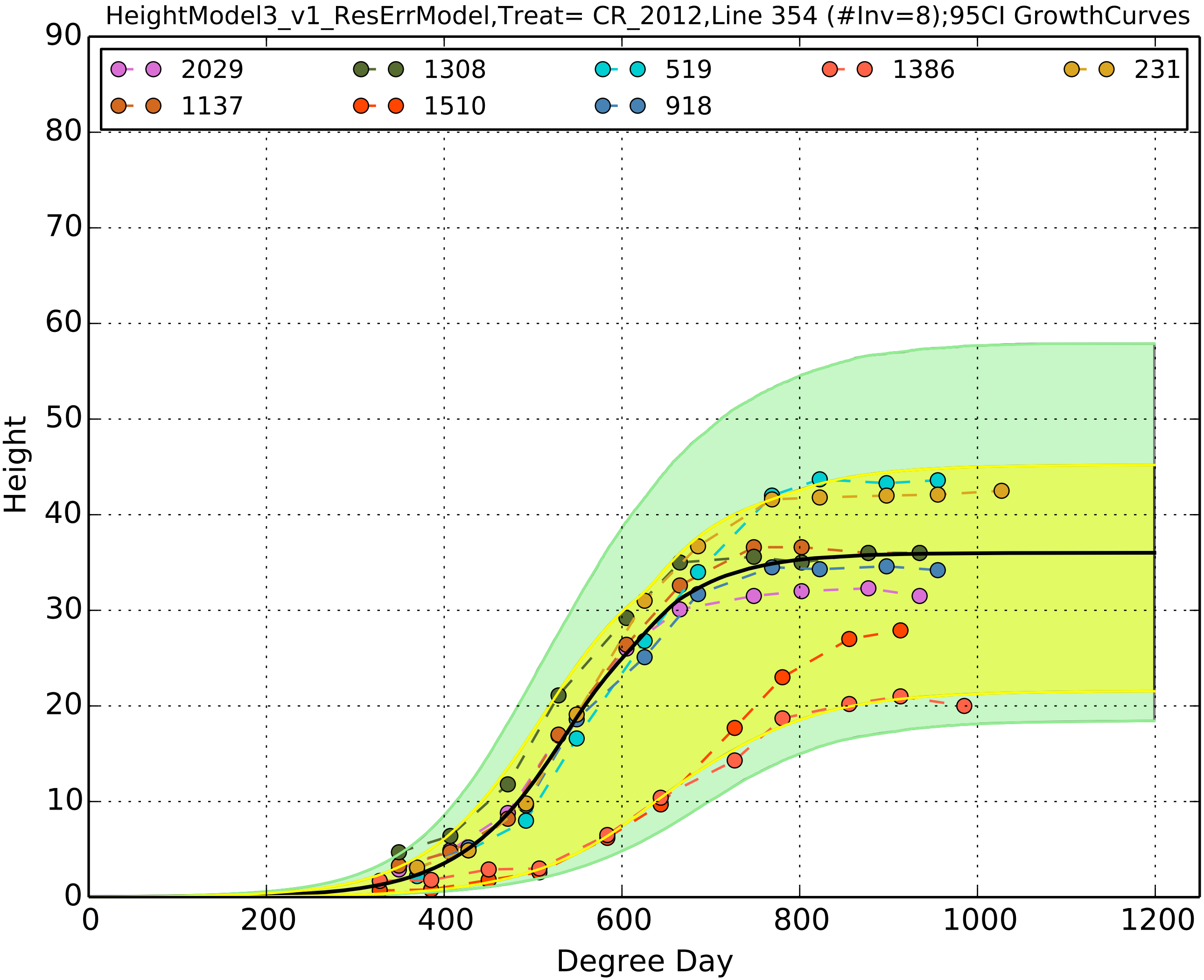

HeightModel3_v1_ResErrModel,Treat= CR_2012,Line 354 (#Inv=8);95CI GrowthCurves
2029
1308
519
1386
231
1137
1510
918
Height
Degree Day
0
10
20
30
40
50
60
70
80
90
0
200
400
600
800
1000
1200

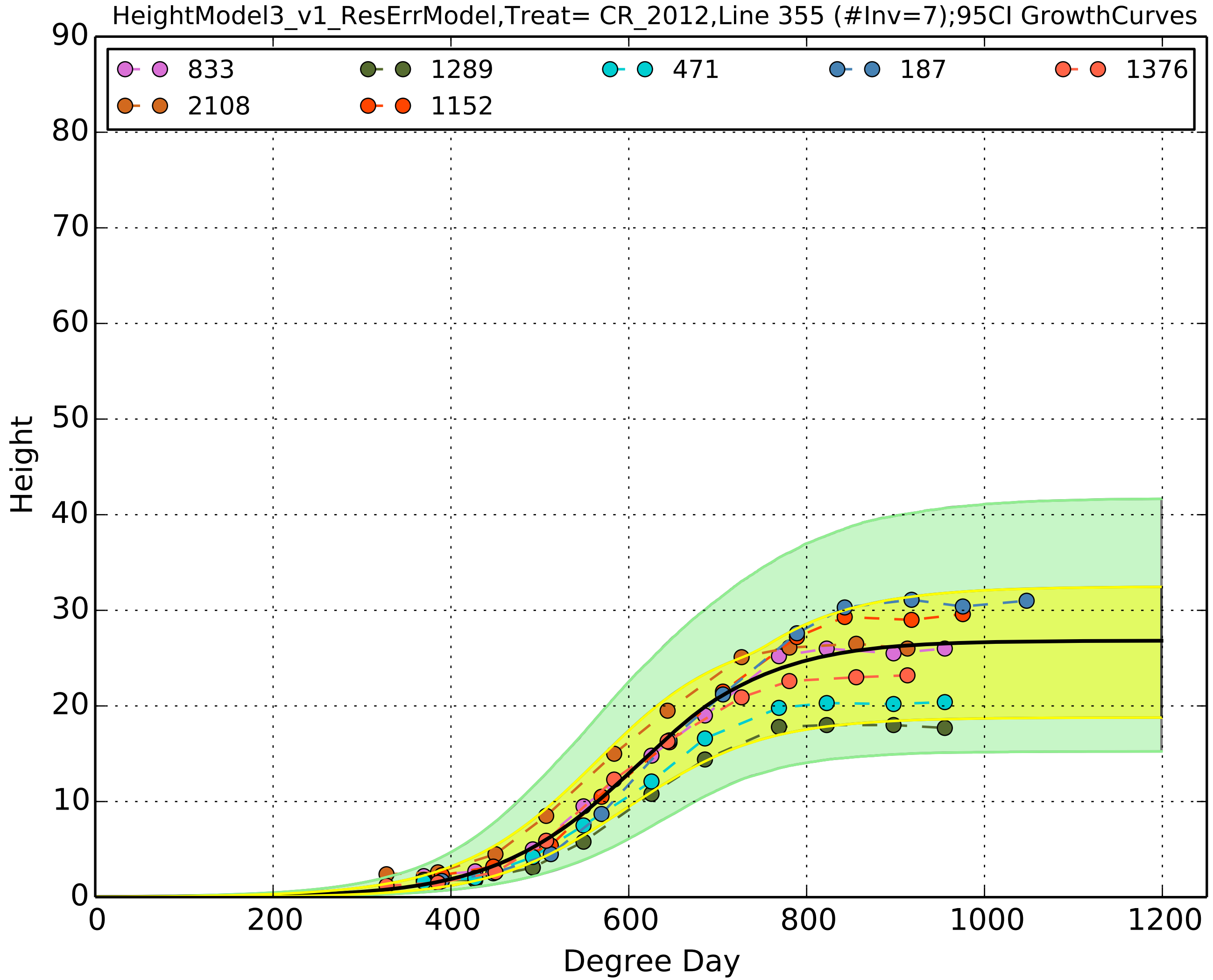

HeightModel3_v1_ResErrModel,Treat= CR_2012,Line 355 (#Inv=7);95CI GrowthCurves
833
1289
471
187
1376
2108
1152
Height
Degree Day
0
10
20
30
40
50
60
70
80
90
0
200
400
600
800
1000
1200

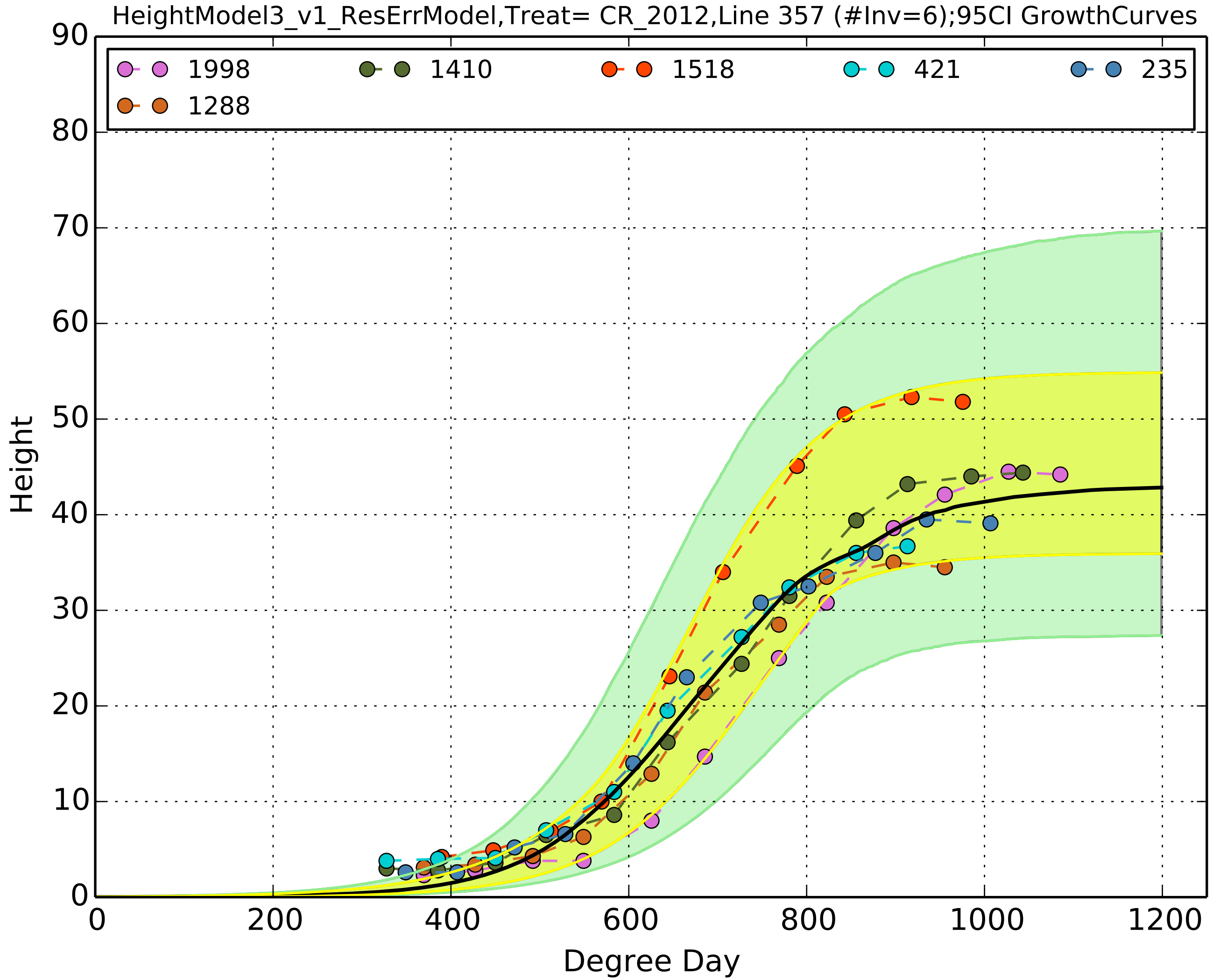

HeightModel3_v1_ResErrModel,Treat= CR_2012,Line 357 (#Inv=6);95CI GrowthCurves
1998
1410
1518
421
235
1288
Height
Degree Day
0
10
20
30
40
50
60
70
80
90
0
200
400
600
800
1000
1200

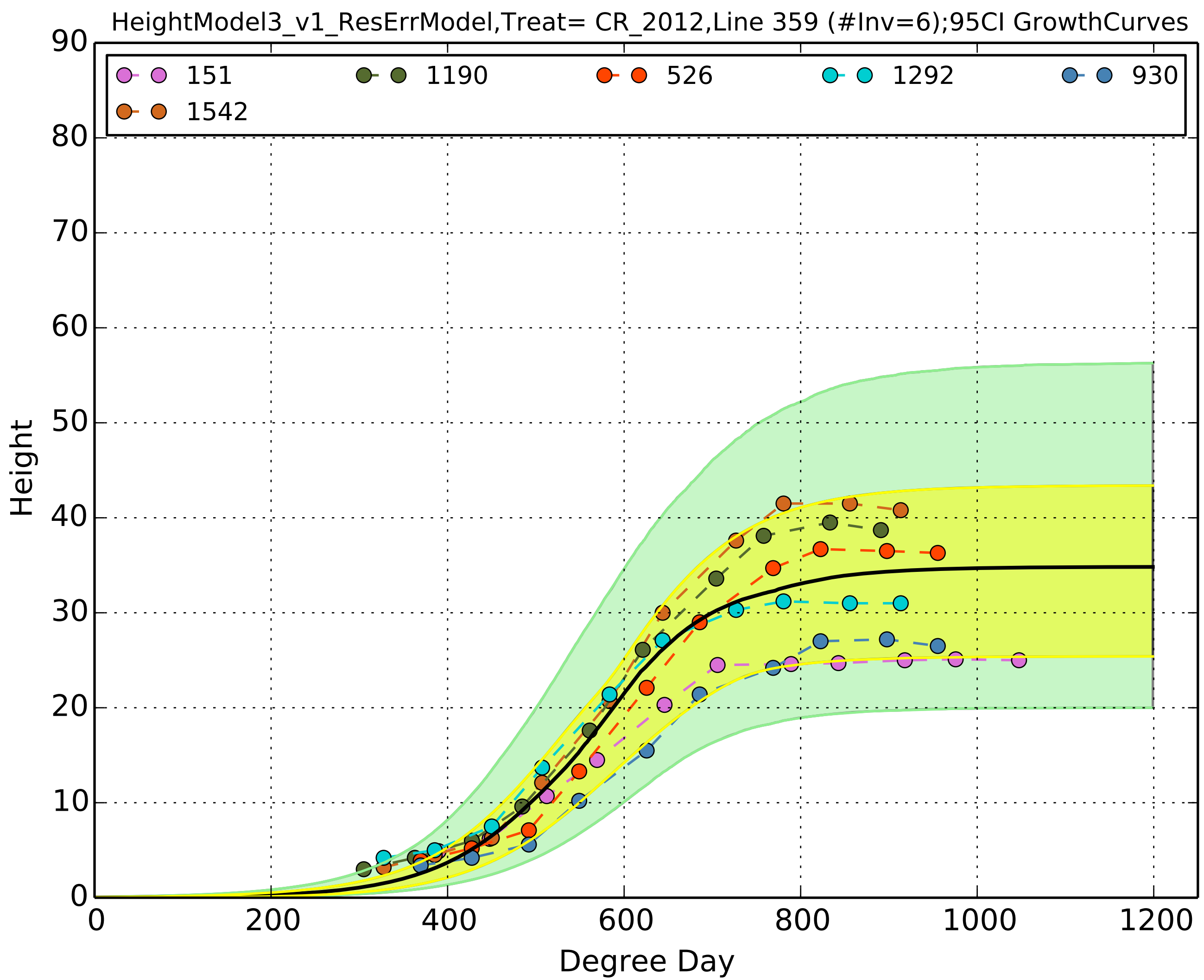
HeightModel3_v1_ResErrModel,Treat= CR_2012,Line 359 (#Inv=6);95CI GrowthCurves
151
1190
526
1292
930
1542
Height
Degree Day
0
10
20
30
40
50
60
70
80
90
0
200
400
600
800
1000
1200

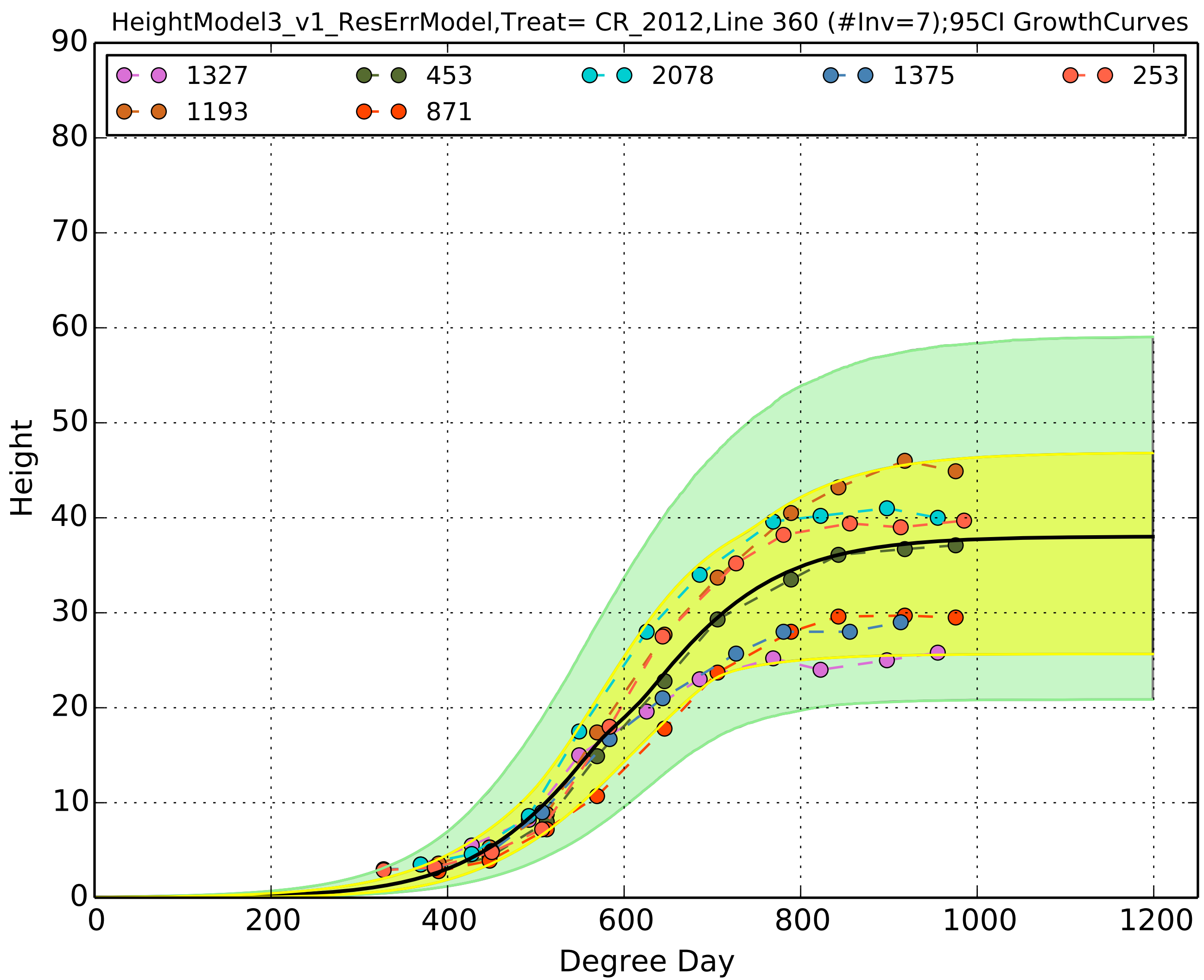

HeightModel3_v1_ResErrModel,Treat= CR_2012,Line 360 (#Inv=7);95CI GrowthCurves
1327
453
2078
1375
253
1193
871
Height
Degree Day
0
10
20
30
40
50
60
70
80
90
0
200
400
600
800
1000
1200

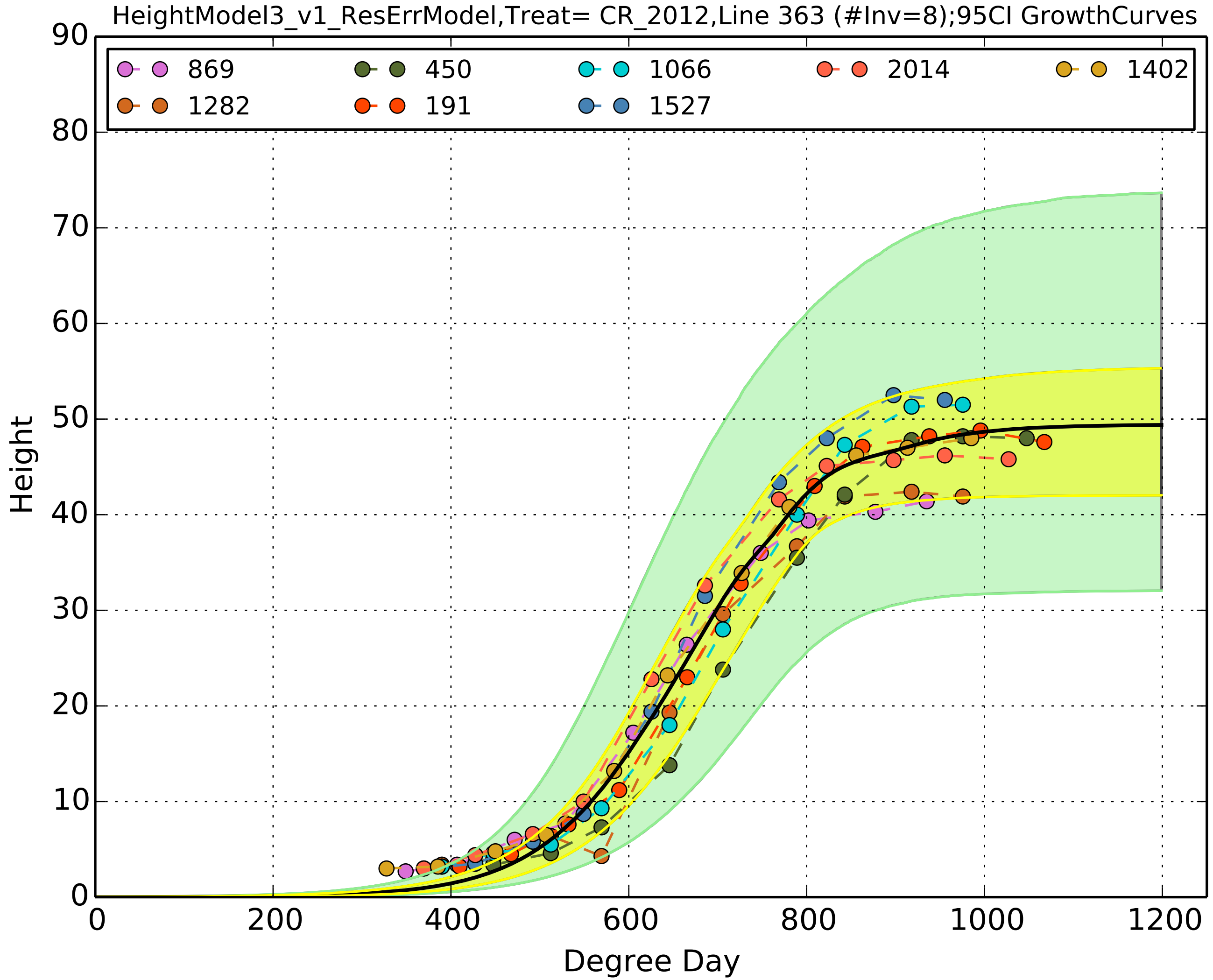

HeightModel3_v1_ResErrModel,Treat= CR_2012,Line 363 (#Inv=8);95CI GrowthCurves
869
450
1066
2014
1402
1282
191
1527
Height
Degree Day
0
10
20
30
40
50
60
70
80
90
0
200
400
600
800
1000
1200

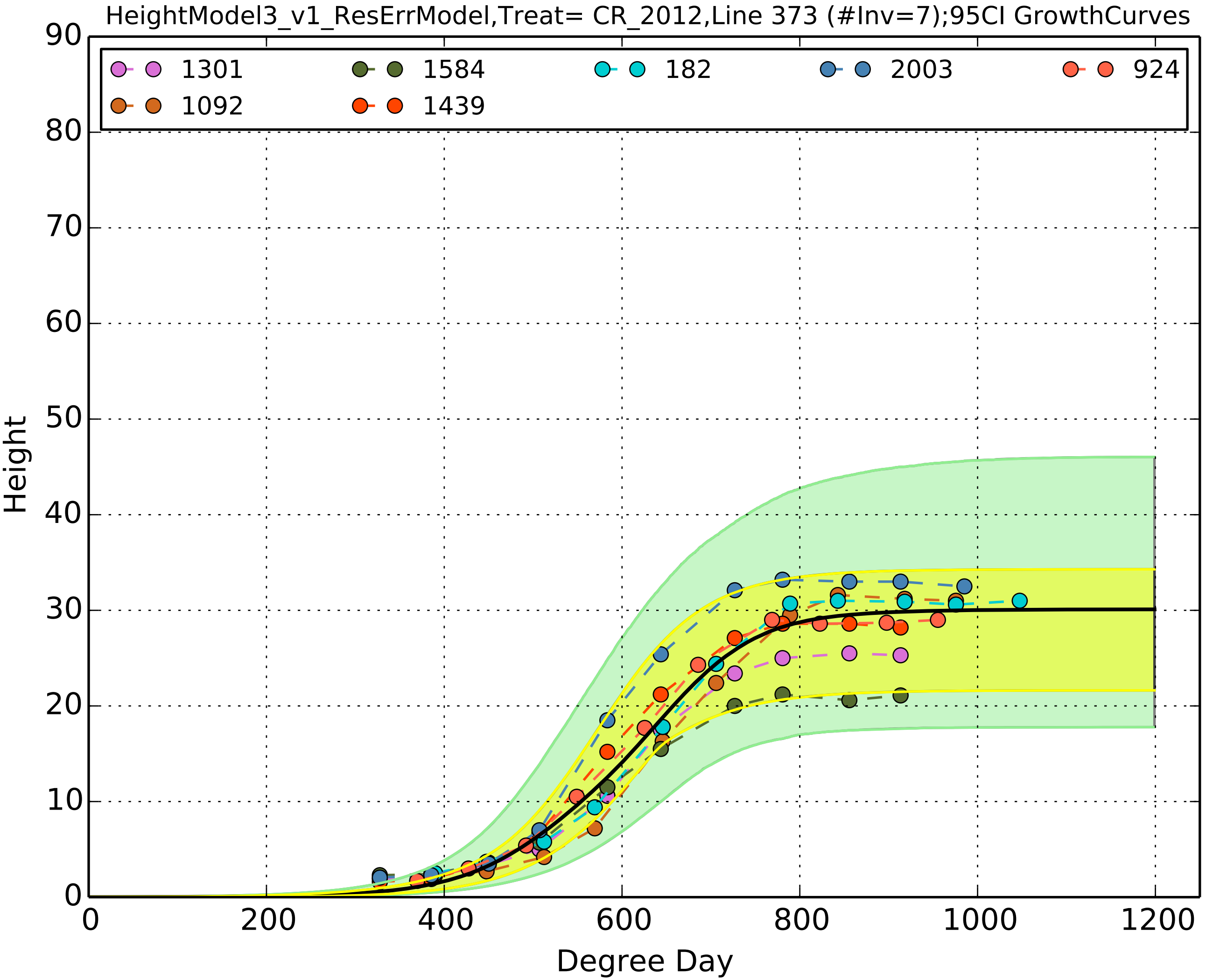

HeightModel3_v1_ResErrModel,Treat= CR_2012,Line 373 (#Inv=7);95CI GrowthCurves
1301
1584
182
2003
924
1092
1439
Height
Degree Day
0
10
20
30
40
50
60
70
80
90
0
200
400
600
800
1000
1200

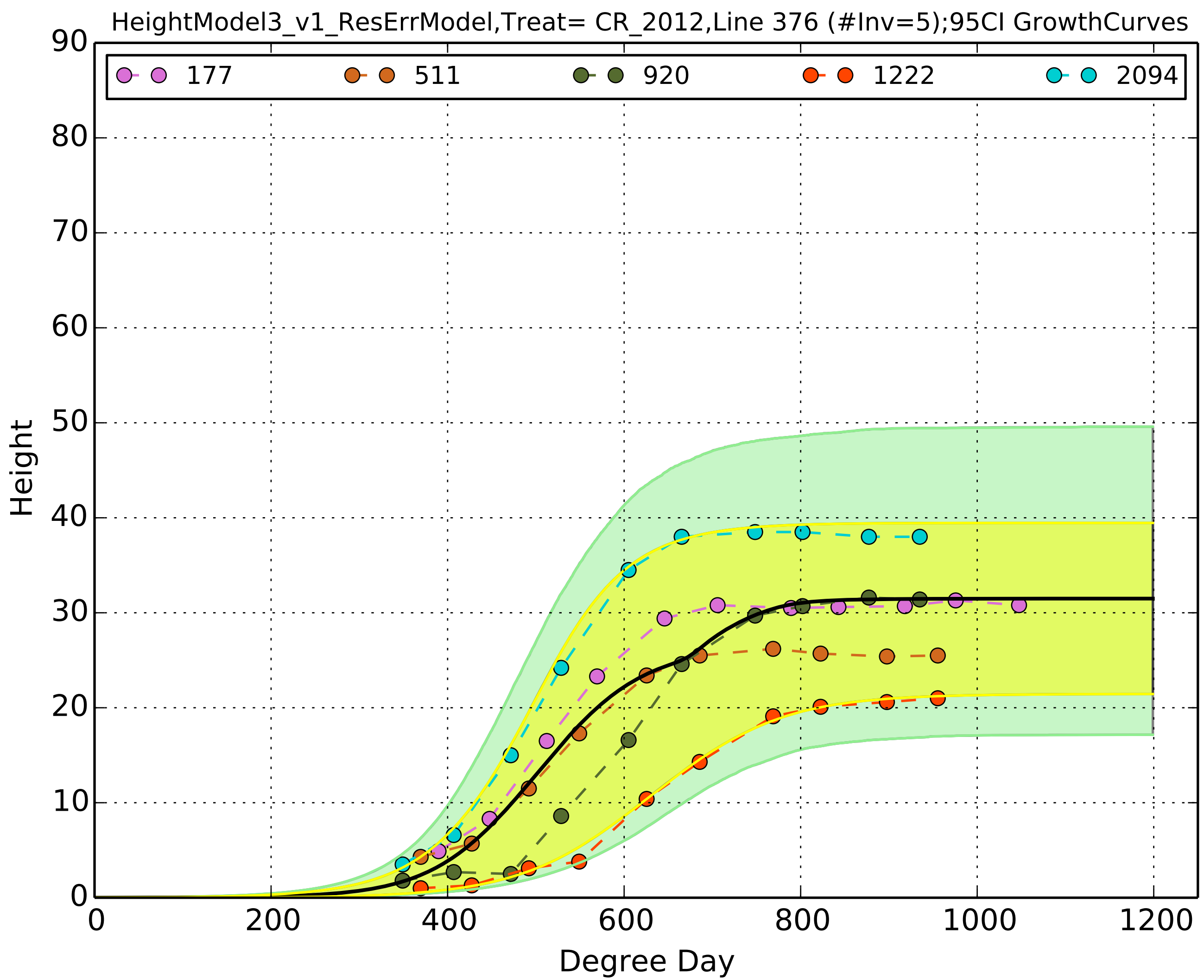
HeightModel3_v1_ResErrModel,Treat= CR_2012,Line 376 (#Inv=5);95CI GrowthCurves
177
511
920
1222
2094
Height
Degree Day
0
10
20
30
40
50
60
70
80
90
0
200
400
600
800
1000
1200

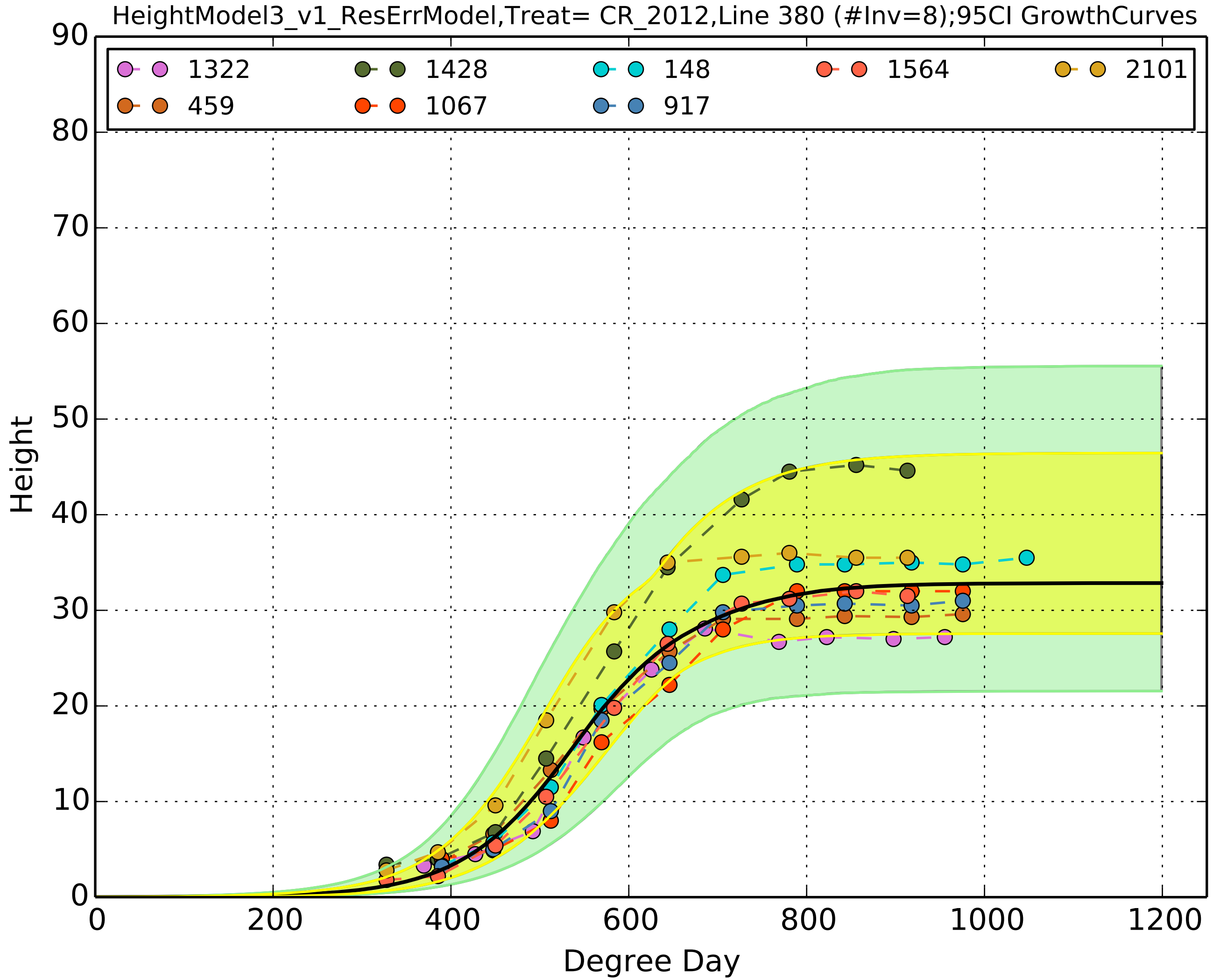
HeightModel3_v1_ResErrModel,Treat= CR_2012,Line 380 (#Inv=8);95CI GrowthCurves
1322
1428
148
1564
2101
459
1067
917
Height
Degree Day
0
10
20
30
40
50
60
70
80
90
0
200
400
600
800
1000
1200

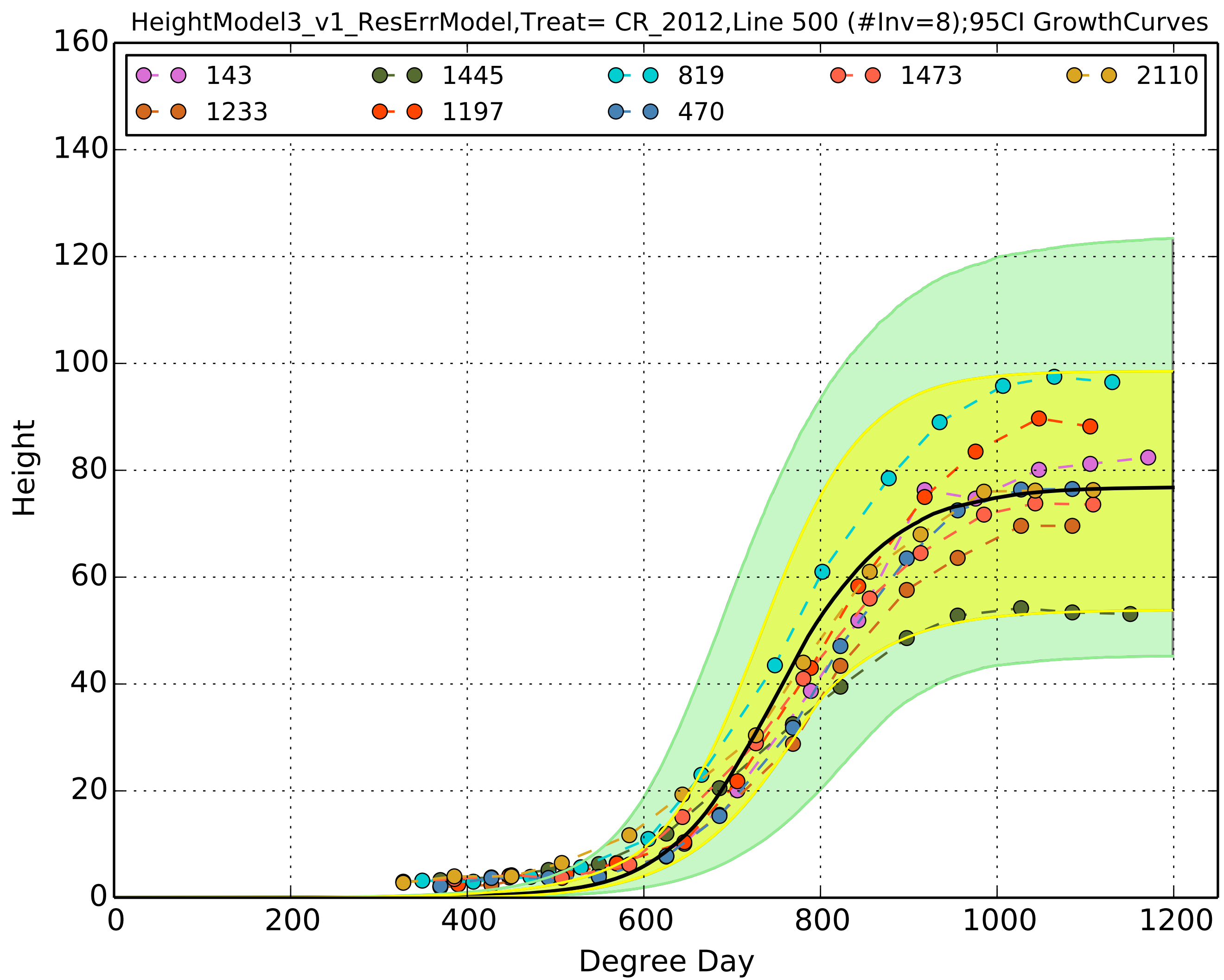
HeightModel3_v1_ResErrModel,Treat= CR_2012,Line 500 (#Inv=8);95CI GrowthCurves
143
1445
819
1473
2110
1233
1197
470
Height
Degree Day
0
20
40
60
80
100
120
140
160
0
200
400
600
800
1000
1200

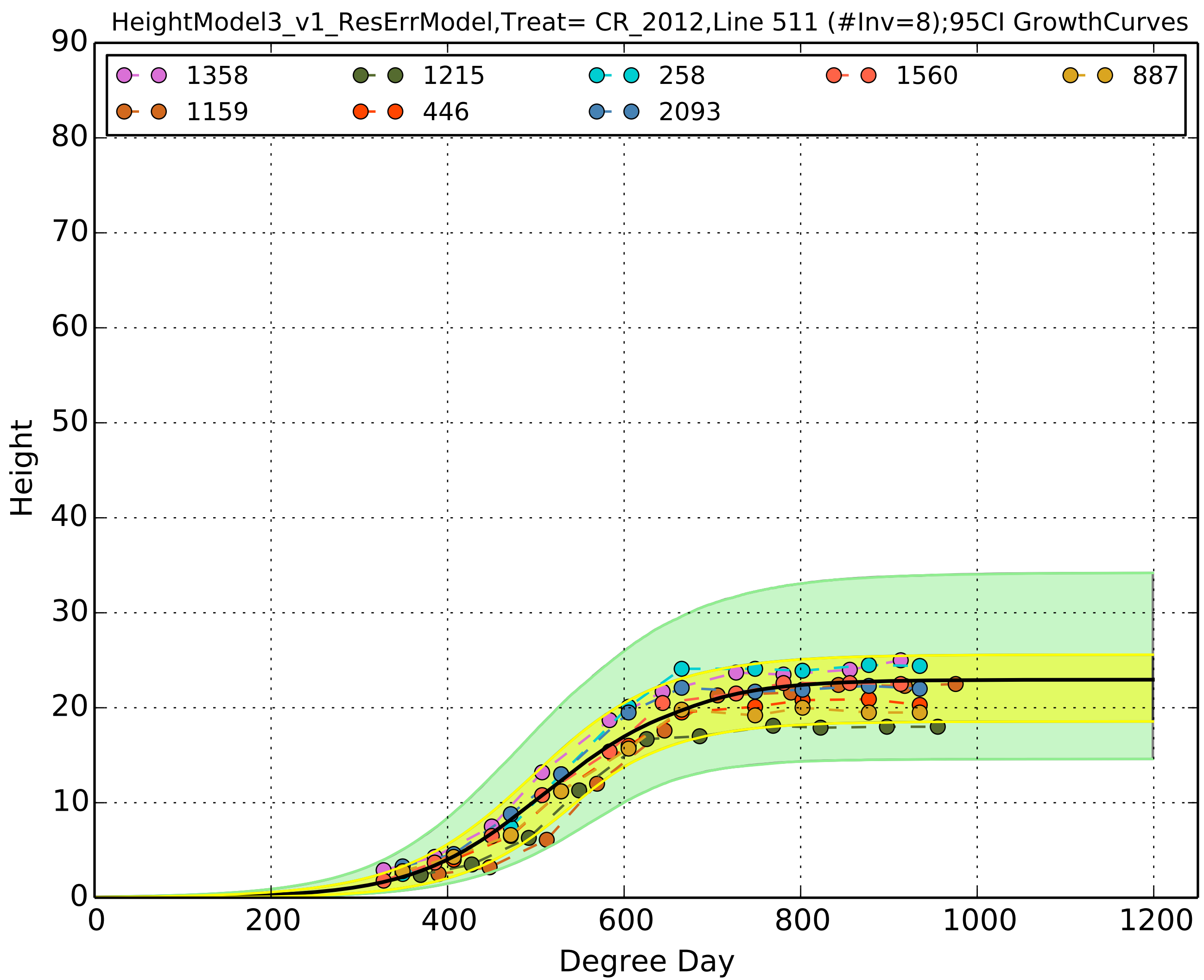

HeightModel3_v1_ResErrModel,Treat= CR_2012,Line 511 (#Inv=8);95CI GrowthCurves
1358
1215
258
1560
887
1159
446
2093
Height
Degree Day
0
10
20
30
40
50
60
70
80
90
0
200
400
600
800
1000
1200

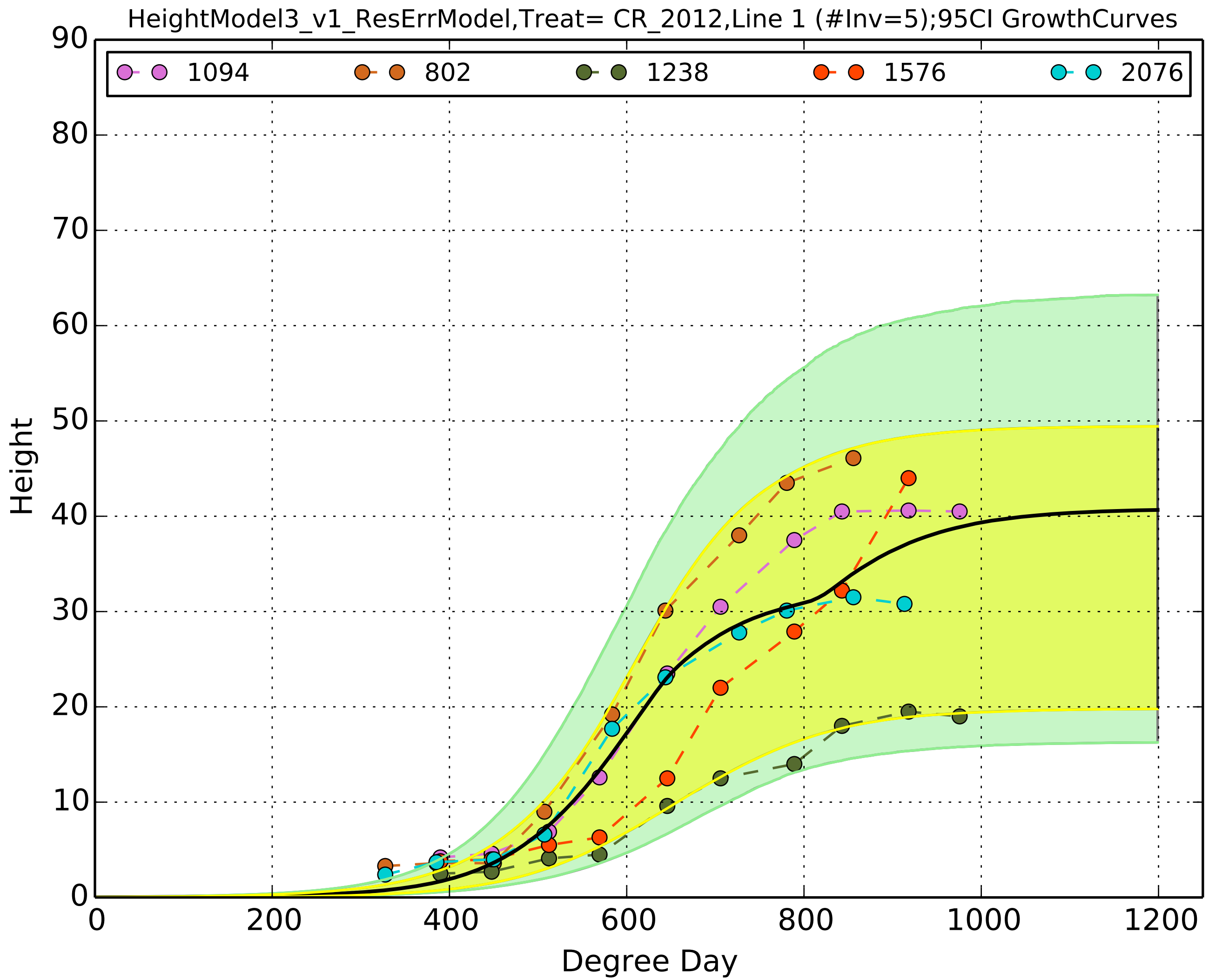

HeightModel3_v1_ResErrModel,Treat= CR_2012,Line 1 (#Inv=5);95CI GrowthCurves
1094
802
1238
1576
2076
Height
Degree Day
0
10
20
30
40
50
60
70
80
90
0
200
400
600
800
1000
1200

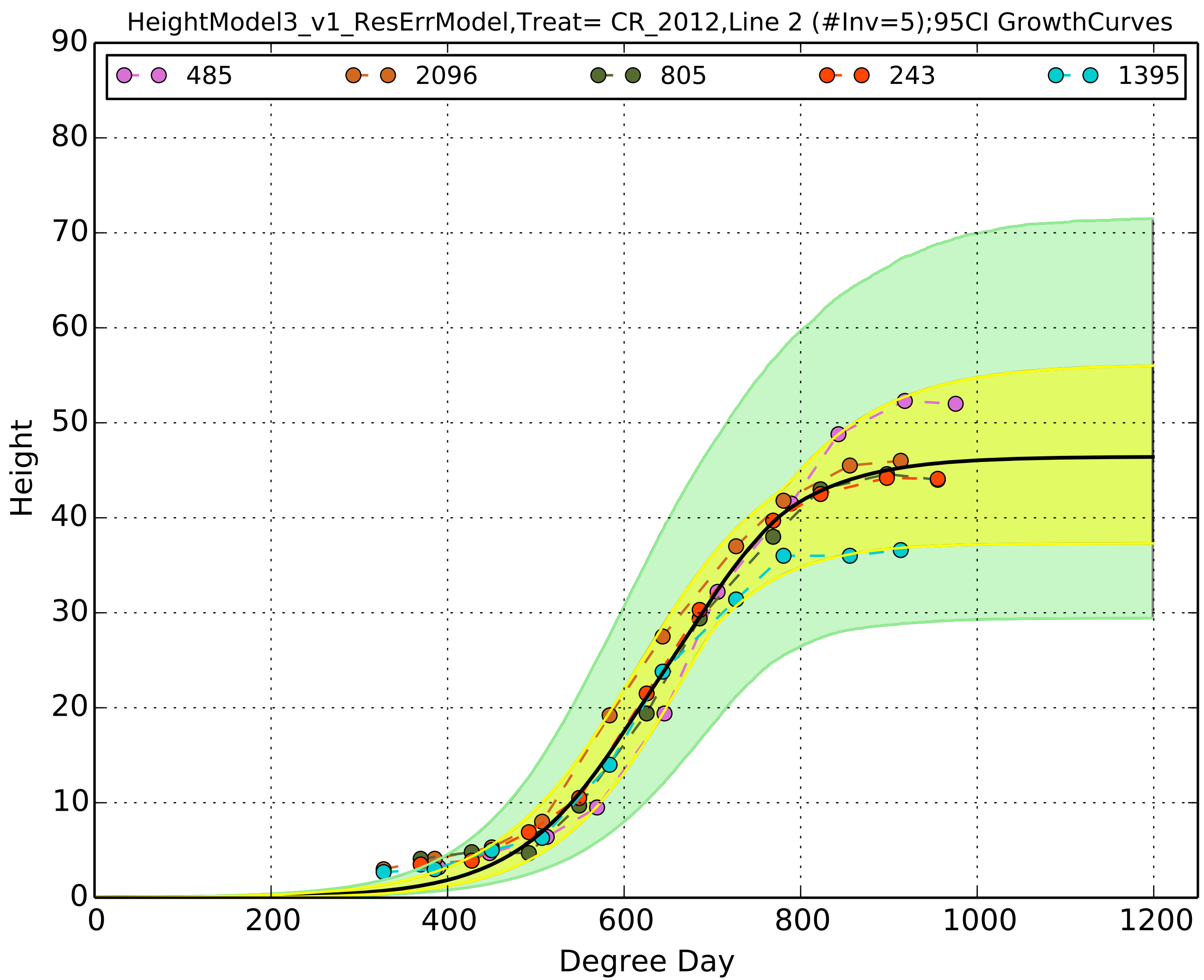

HeightModel3_v1_ResErrModel,Treat= CR_2012,Line 2 (#Inv=5);95CI GrowthCurves
485
2096
805
243
1395
Height
Degree Day
0
10
20
30
40
50
60
70
80
90
0
200
400
600
800
1000
1200

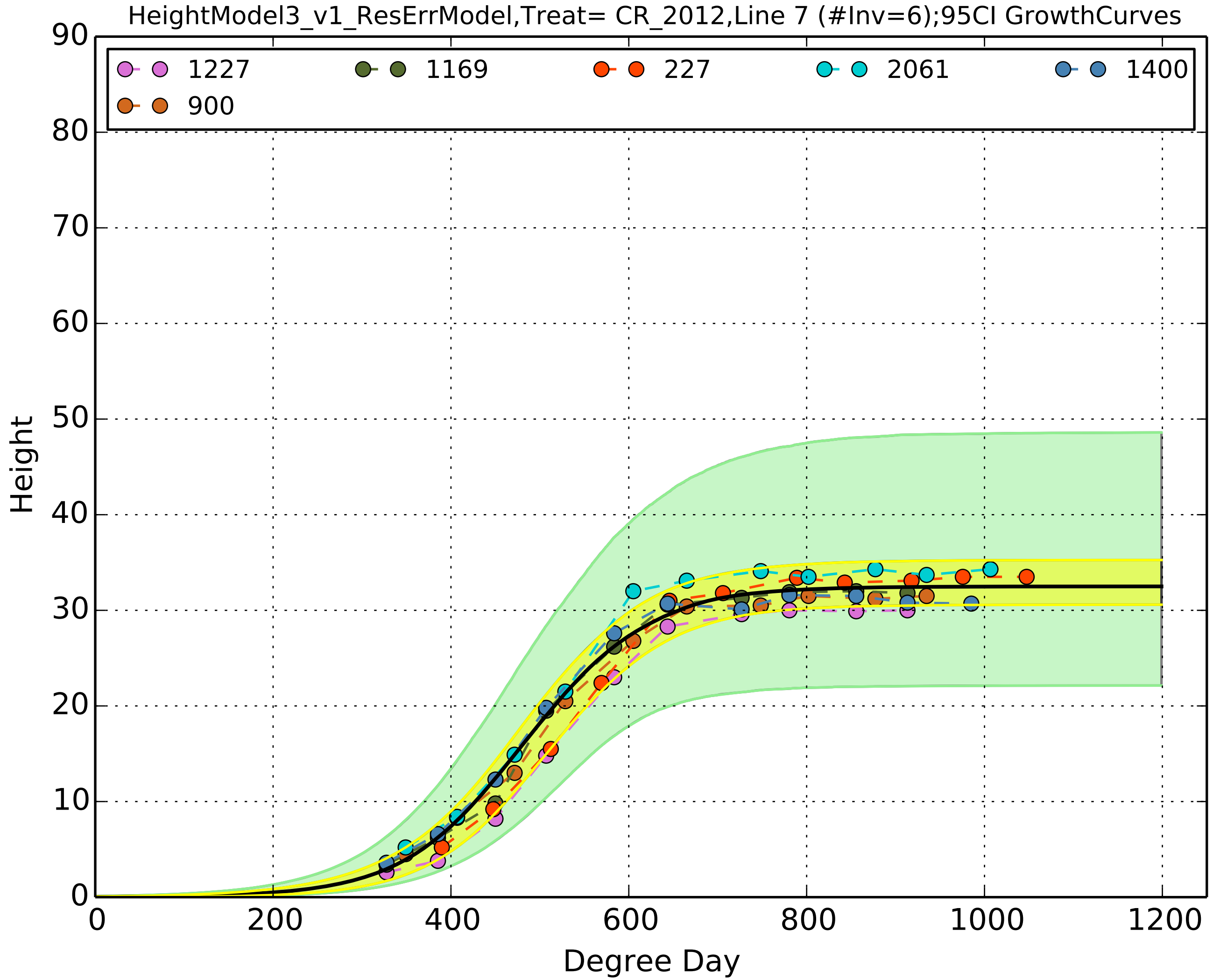
HeightModel3_v1_ResErrModel,Treat= CR_2012,Line 7 (#Inv=6);95CI GrowthCurves
1227
1169
227
2061
1400
900
Height
Degree Day
0
10
20
30
40
50
60
70
80
90
0
200
400
600
800
1000
1200

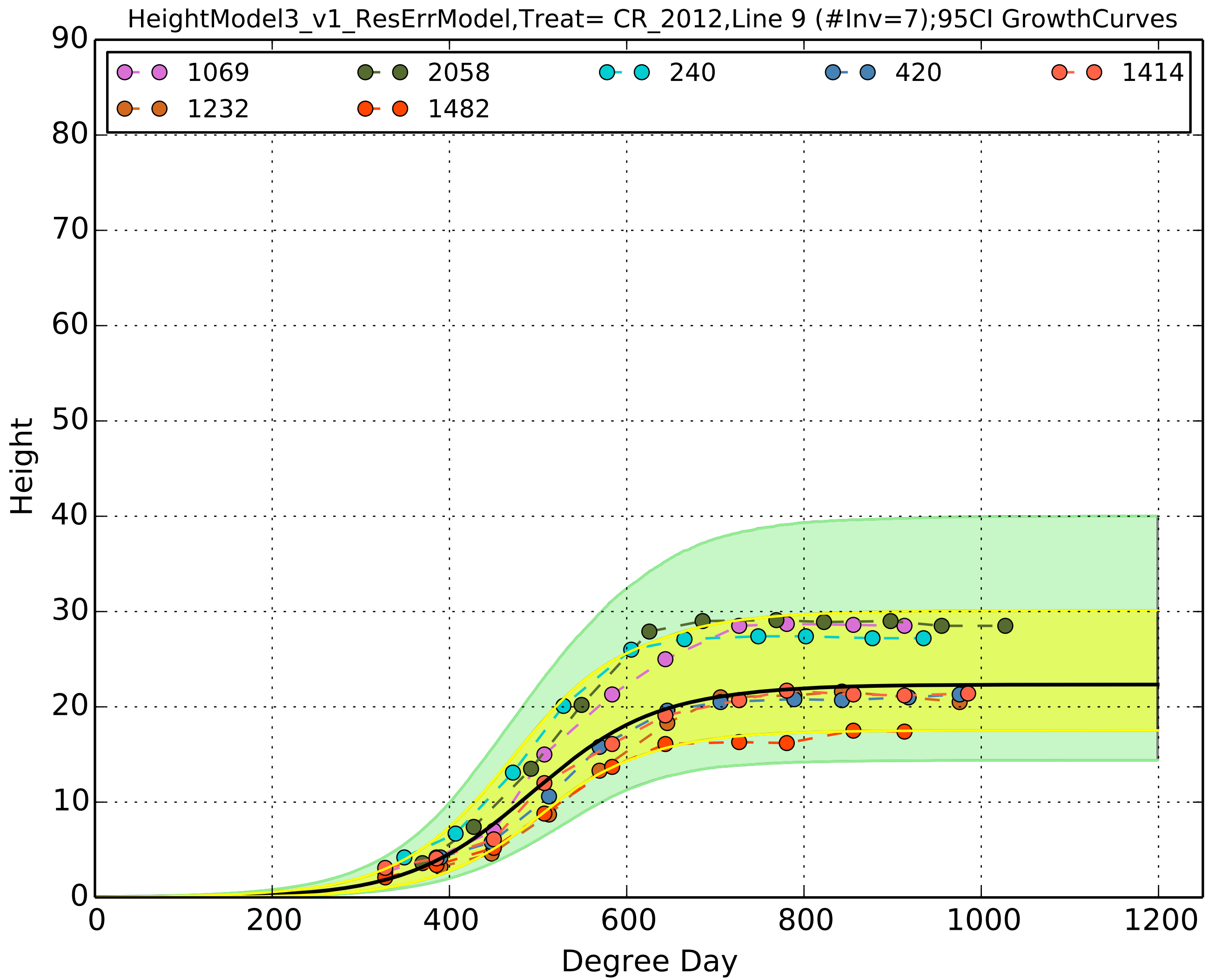

HeightModel3_v1_ResErrModel,Treat= CR_2012,Line 9 (#Inv=7);95CI GrowthCurves
1069
2058
240
420
1414
1232
1482
Height
Degree Day
0
10
20
30
40
50
60
70
80
90
0
200
400
600
800
1000
1200

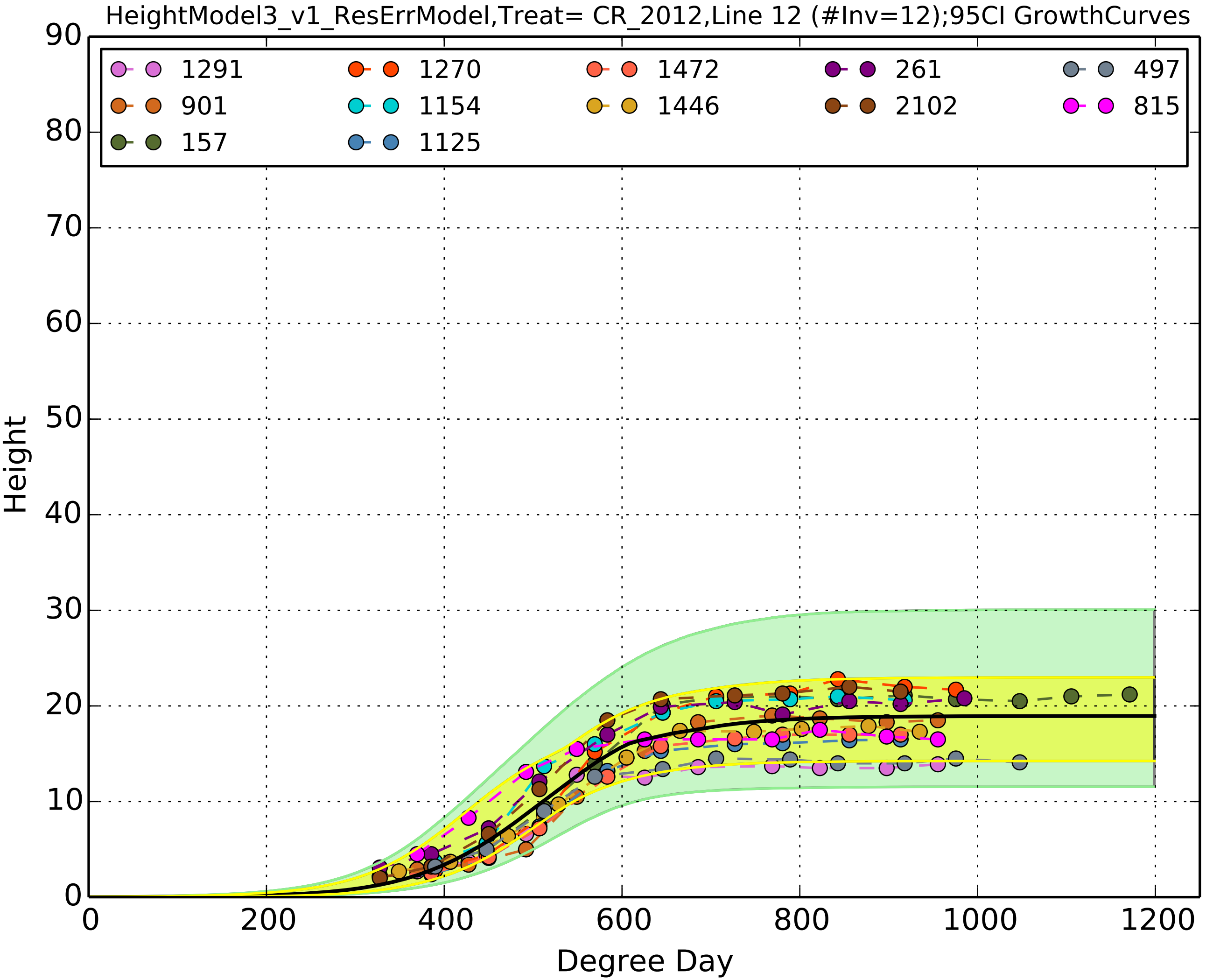

HeightModel3_v1_ResErrModel,Treat= CR_2012,Line 12 (#Inv=12);95CI GrowthCurves
1291
1270
1472
261
497
901
1154
1446
2102
815
157
1125
Height
Degree Day
0
200
400
600
800
1000
1200
0
10
20
30
40
50
60
70
80
90

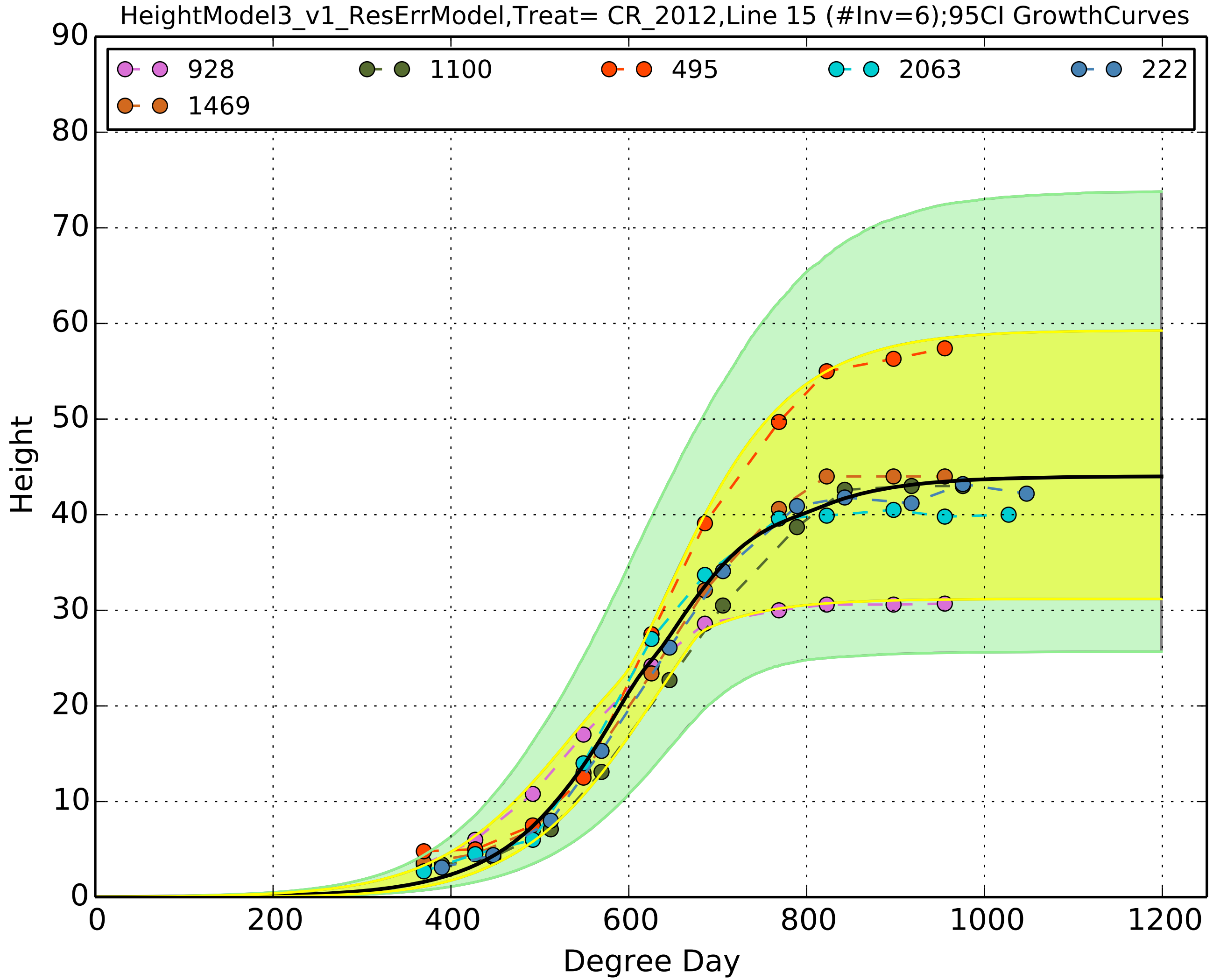

HeightModel3_v1_ResErrModel,Treat= CR_2012,Line 15 (#Inv=6);95CI GrowthCurves
928
1100
495
2063
222
1469
Height
Degree Day
0
10
20
30
40
50
60
70
80
90
0
200
400
600
800
1000
1200

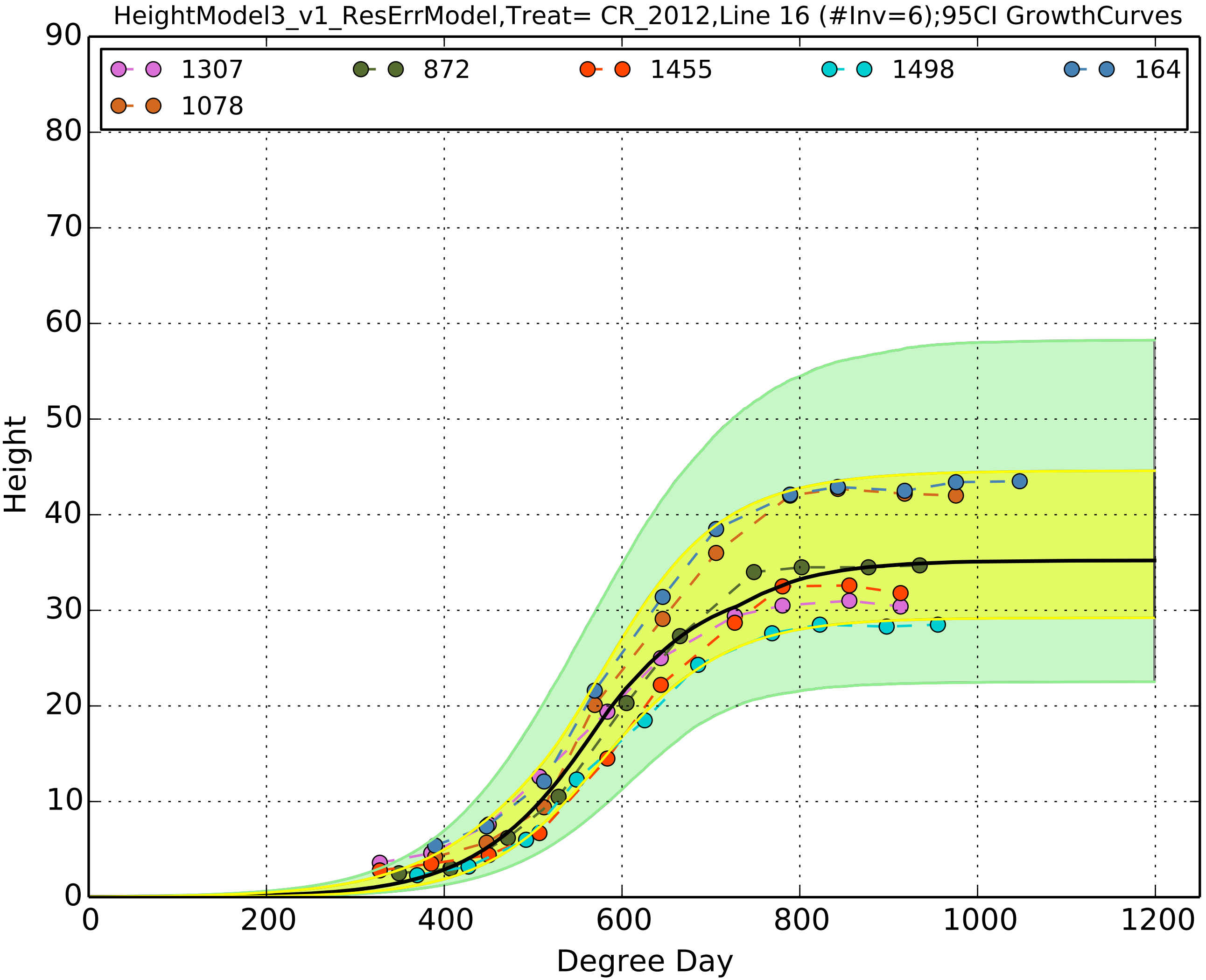
HeightModel3_v1_ResErrModel,Treat= CR_2012,Line 16 (#Inv=6);95CI GrowthCurves
1307
872
1455
1498
164
1078
Height
Degree Day
0
10
20
30
40
50
60
70
80
90
0
200
400
600
800
1000
1200

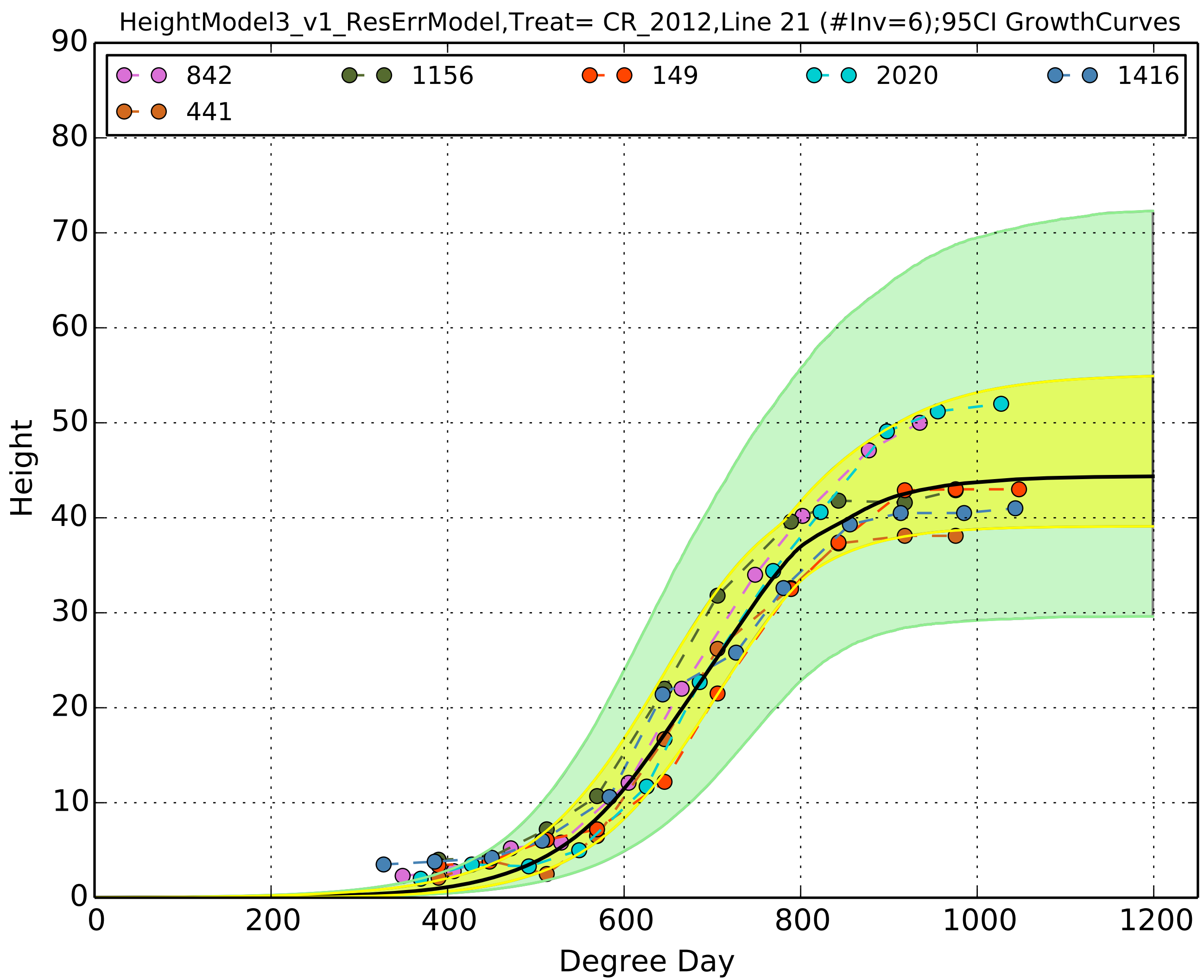
HeightModel3_v1_ResErrModel,Treat= CR_2012,Line 21 (#Inv=6);95CI GrowthCurves
842
1156
149
2020
1416
441
Height
Degree Day
0
10
20
30
40
50
60
70
80
90
0
200
400
600
800
1000
1200

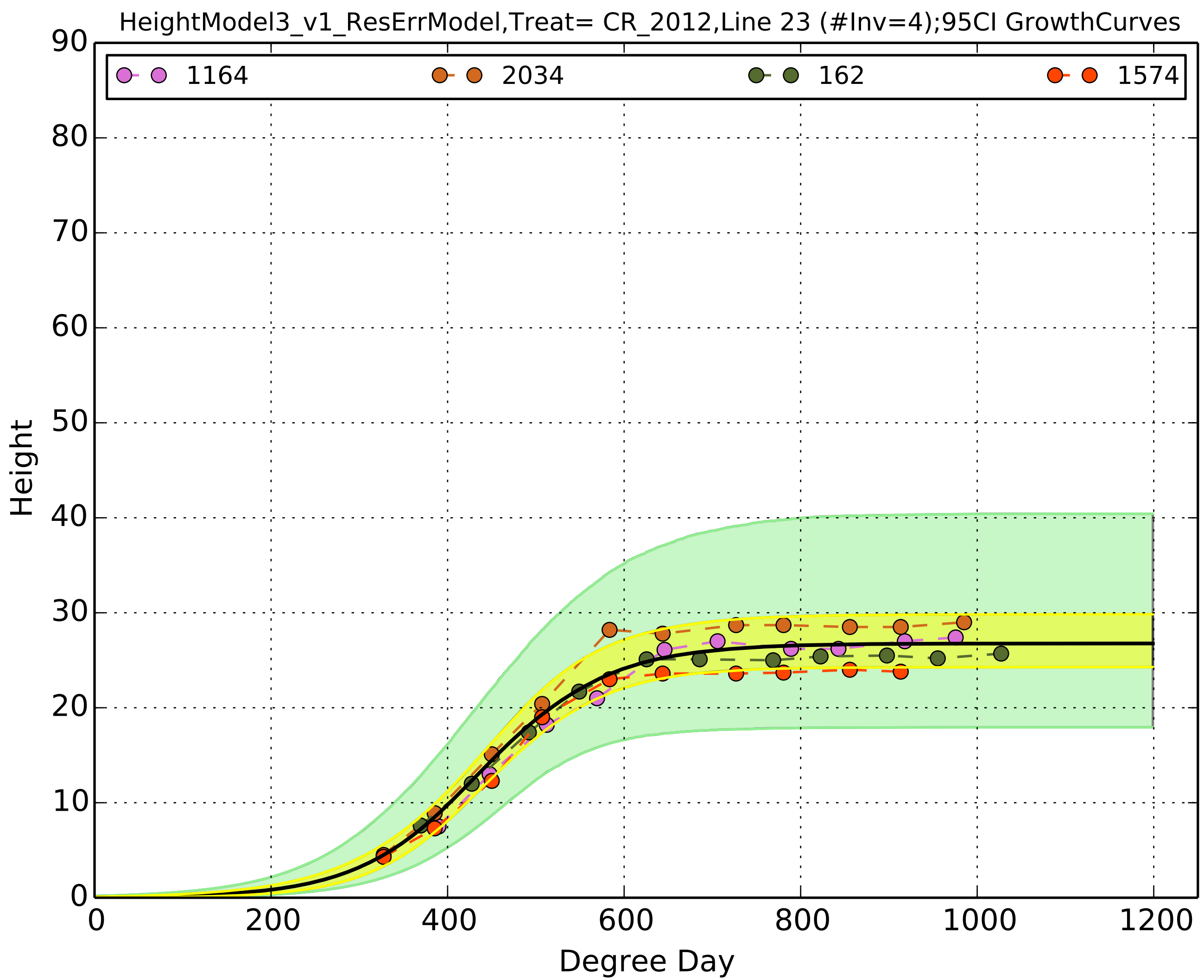
HeightModel3_v1_ResErrModel,Treat= CR_2012,Line 23 (#Inv=4);95CI GrowthCurves
1164
2034
162
1574
Height
Degree Day
0
10
20
30
40
50
60
70
80
90
0
200
400
600
800
1000
1200

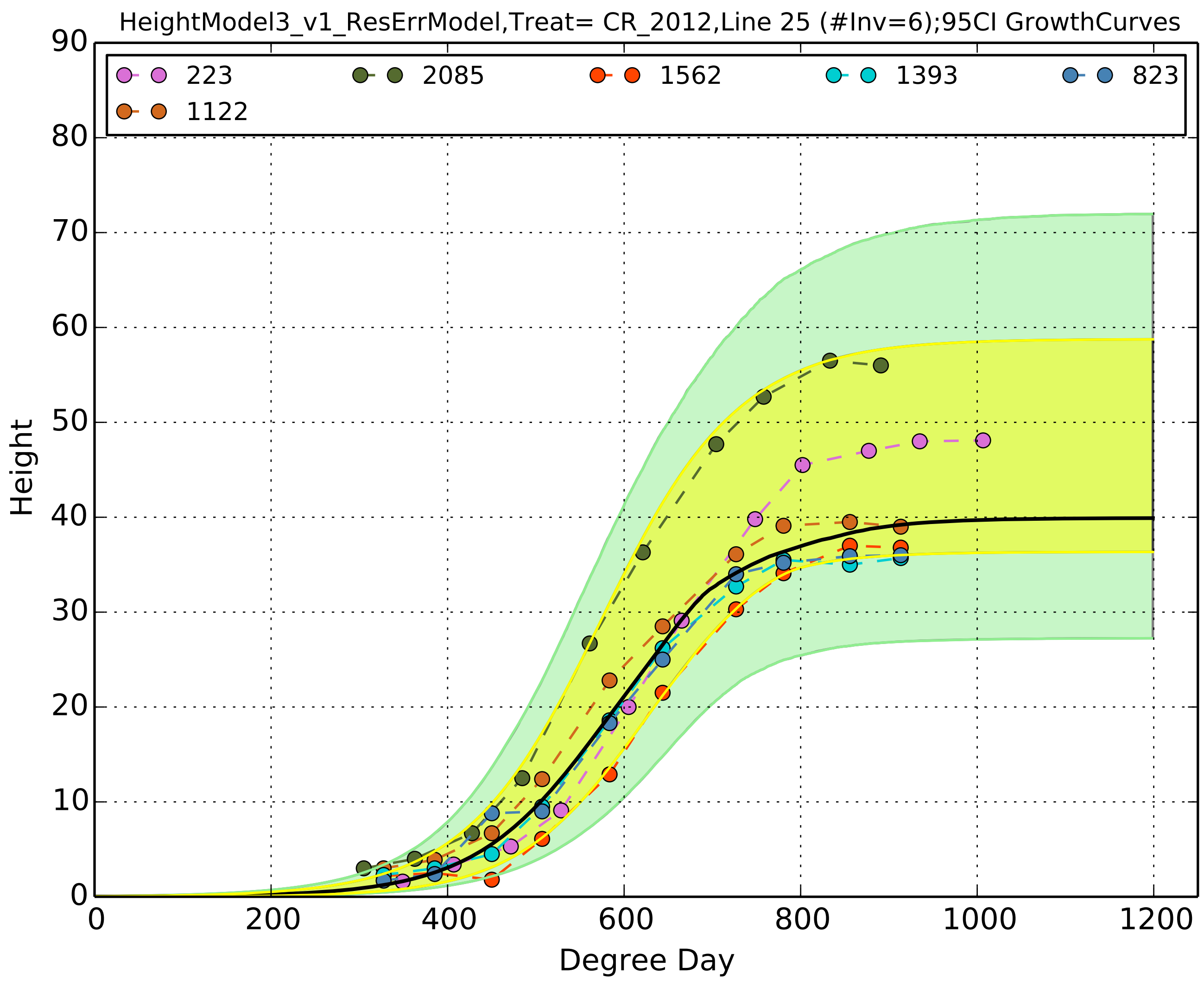

HeightModel3_v1_ResErrModel,Treat= CR_2012,Line 25 (#Inv=6);95CI GrowthCurves
223
2085
1562
1393
823
1122
Height
Degree Day
0
10
20
30
40
50
60
70
80
90
0
200
400
600
800
1000
1200

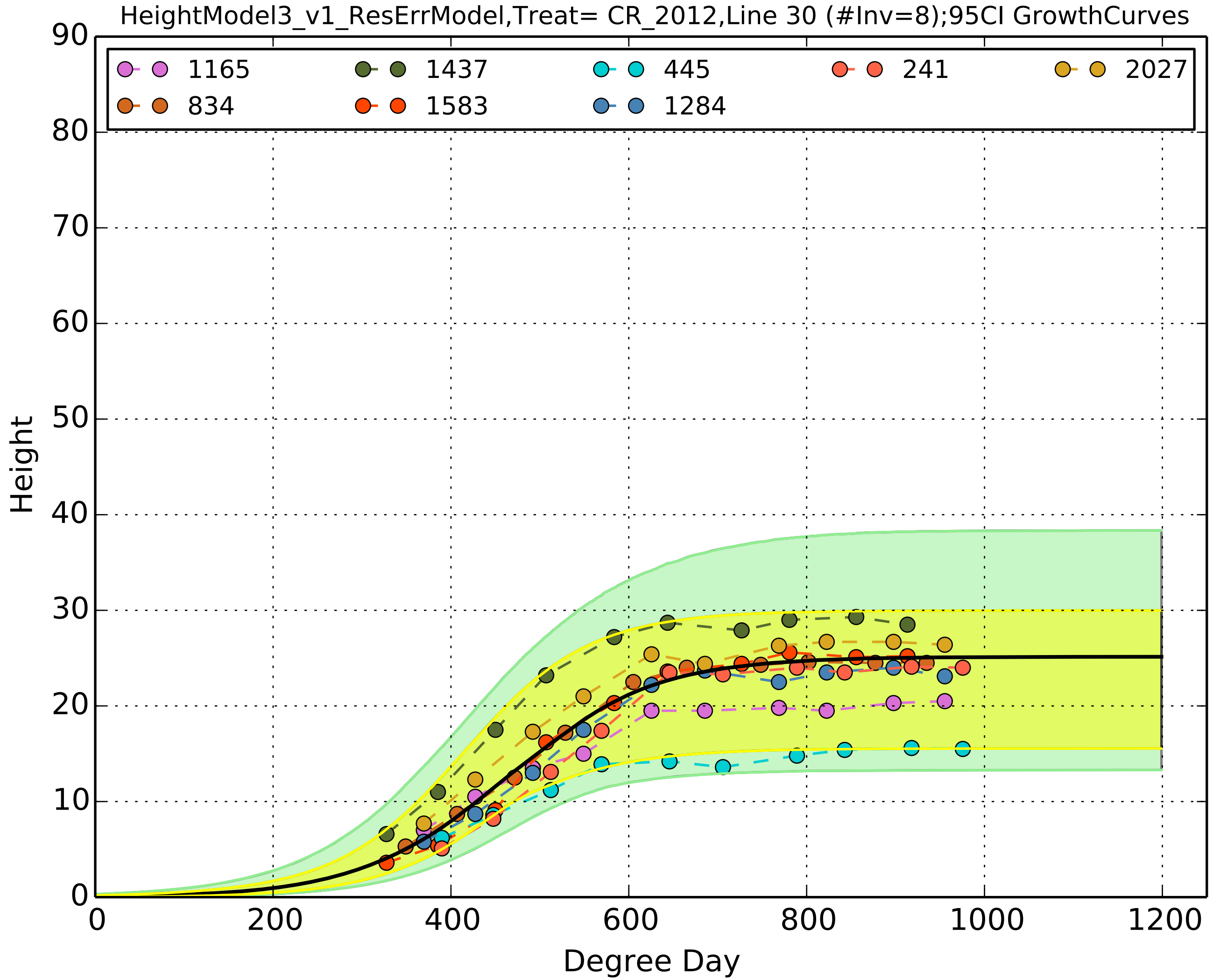
HeightModel3_v1_ResErrModel,Treat= CR_2012,Line 30 (#Inv=8);95CI GrowthCurves
1165
1437
445
241
2027
834
1583
1284
Height
Degree Day
0
10
20
30
40
50
60
70
80
90
0
200
400
600
800
1000
1200

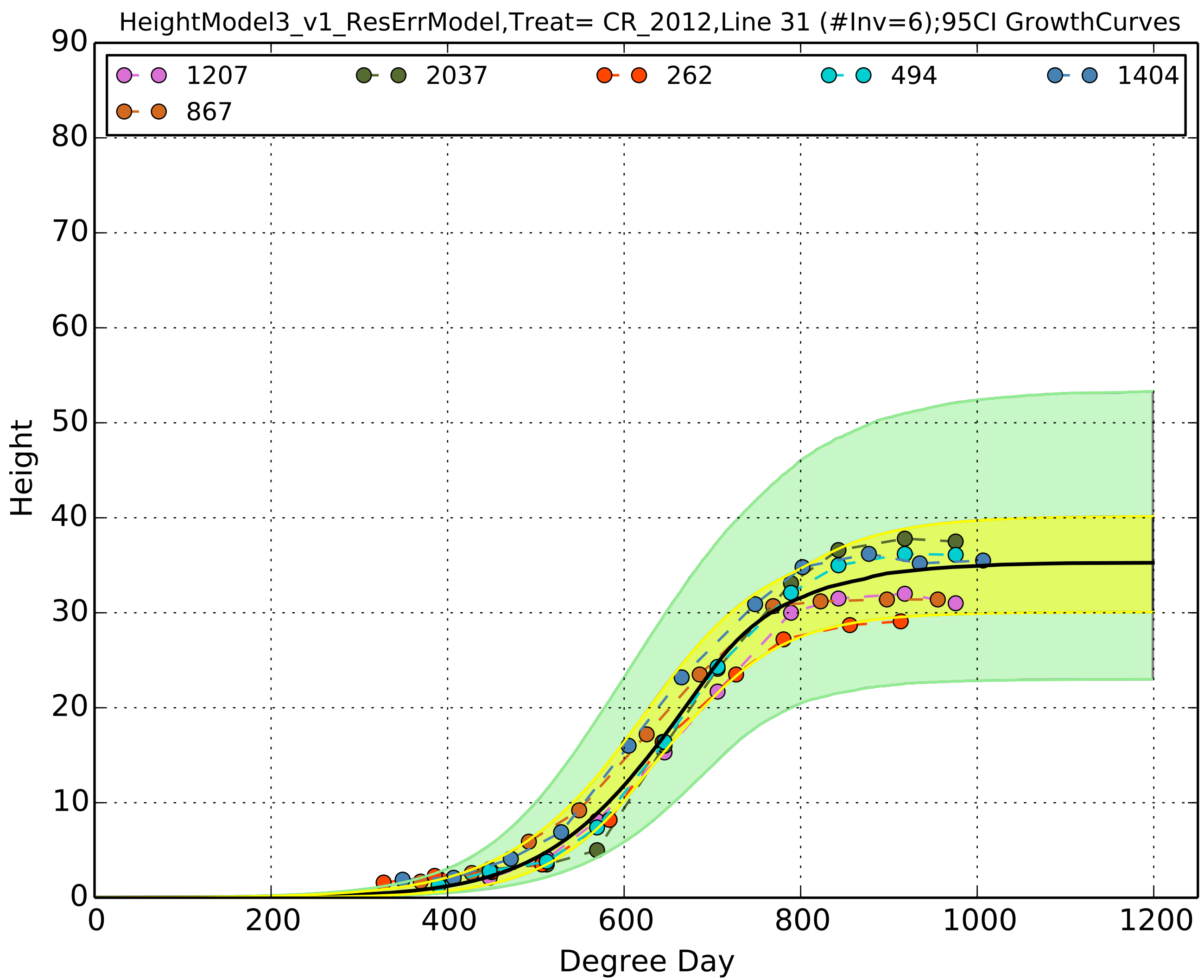

HeightModel3_v1_ResErrModel,Treat= CR_2012,Line 31 (#Inv=6);95CI GrowthCurves
1207
2037
262
494
1404
867
Height
Degree Day
0
10
20
30
40
50
60
70
80
90
0
200
400
600
800
1000
1200

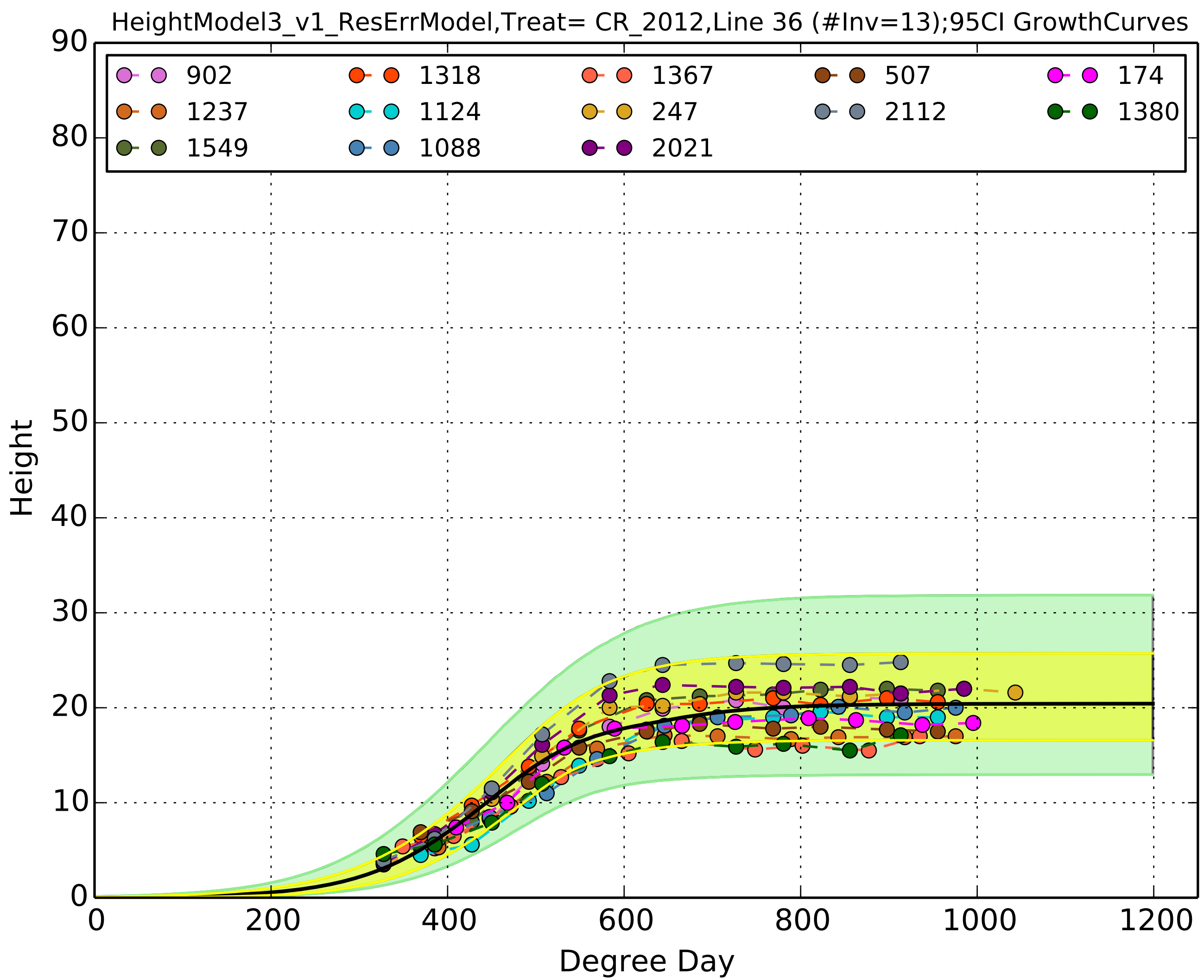

HeightModel3_v1_ResErrModel,Treat= CR_2012,Line 36 (#Inv=13);95CI GrowthCurves
902
1318
1367
507
174
1237
1124
247
2112
1380
1549
1088
2021
Height
Degree Day
0
10
20
30
40
50
60
70
80
90
0
200
400
600
800
1000
1200

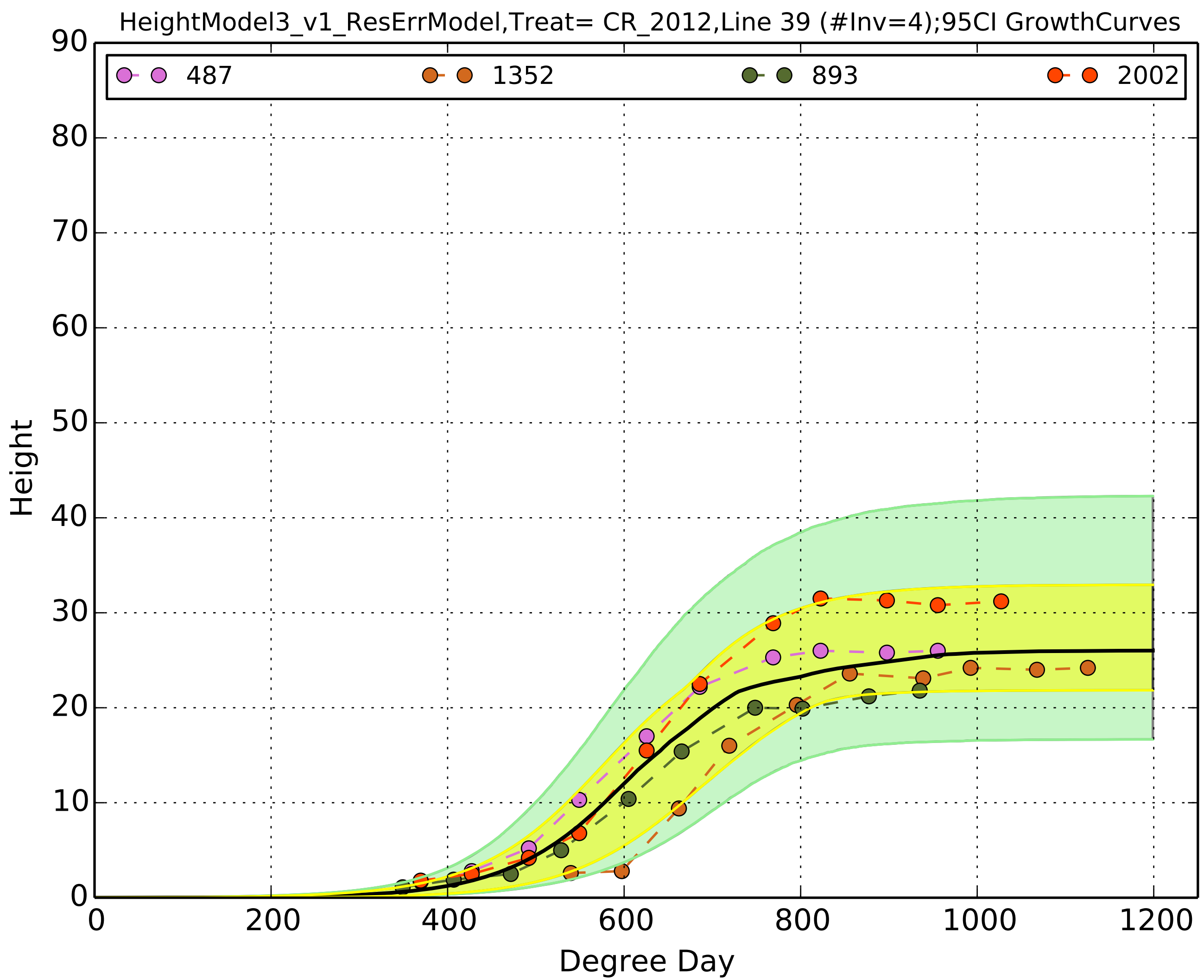
HeightModel3_v1_ResErrModel,Treat= CR_2012,Line 39 (#Inv=4);95CI GrowthCurves
487
1352
893
2002
Height
Degree Day
0
10
20
30
40
50
60
70
80
90
0
200
400
600
800
1000
1200

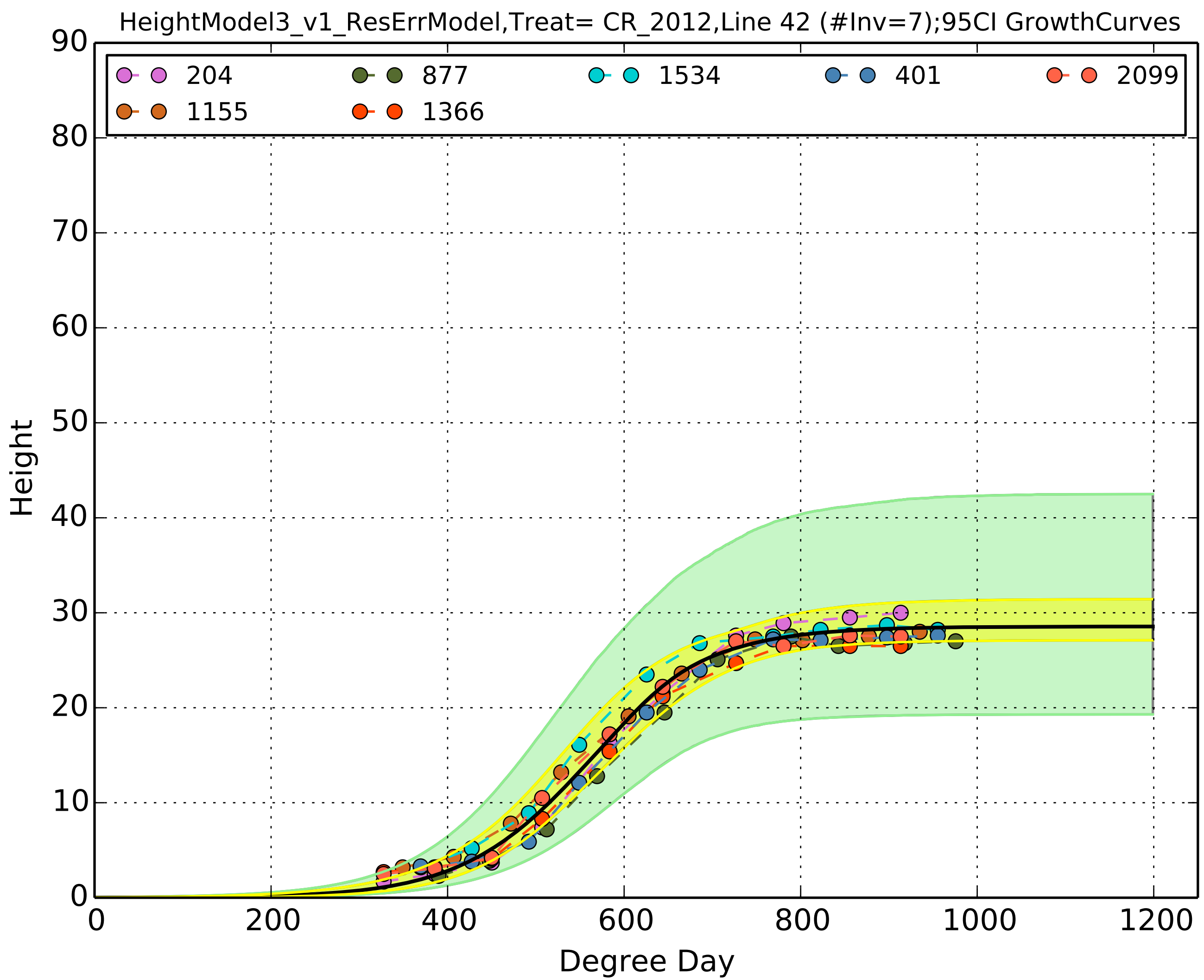

HeightModel3_v1_ResErrModel,Treat= CR_2012,Line 42 (#Inv=7);95CI GrowthCurves
204
877
1534
401
2099
1155
1366
Height
Degree Day
0
10
20
30
40
50
60
70
80
90
0
200
400
600
800
1000
1200

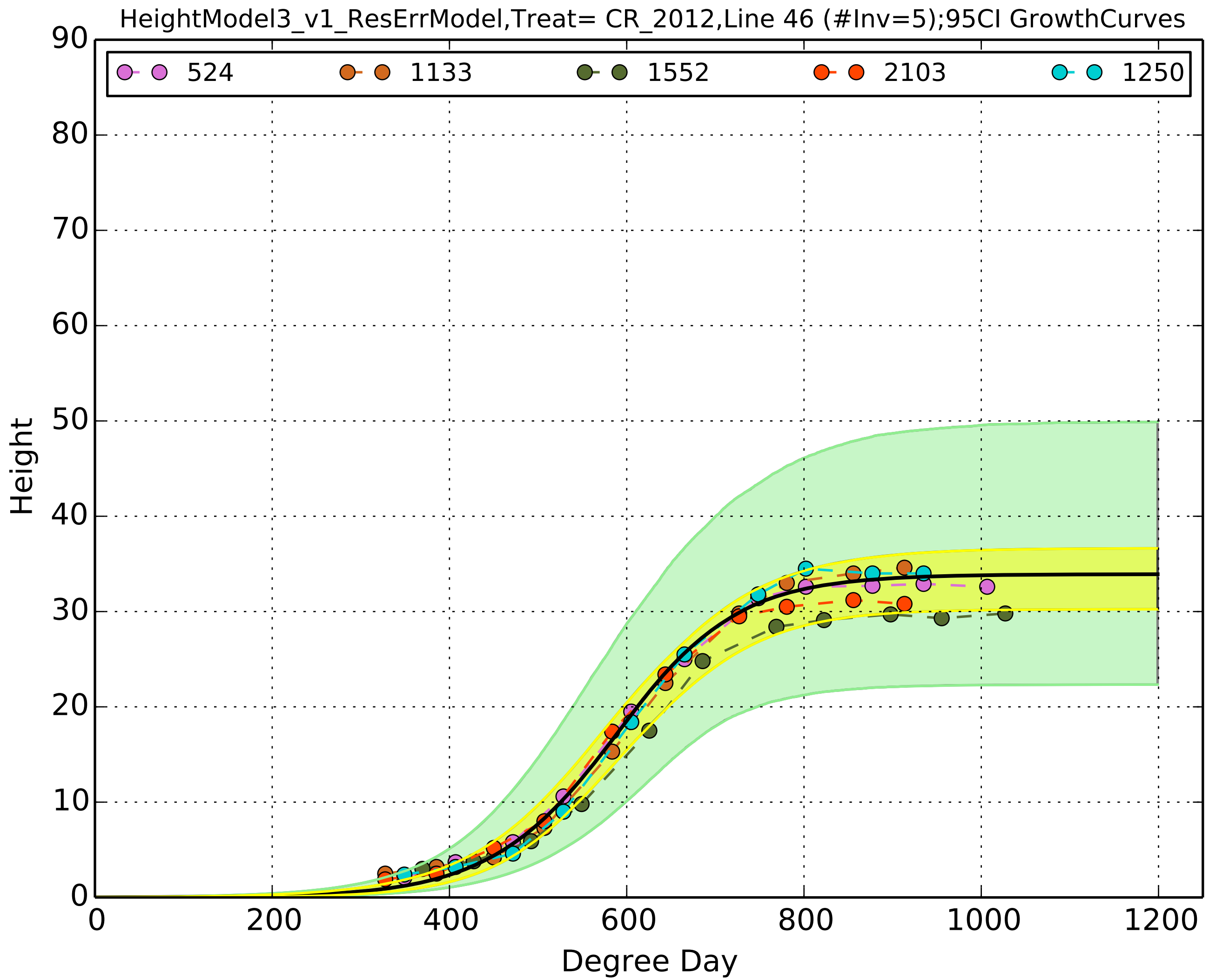
HeightModel3_v1_ResErrModel,Treat= CR_2012,Line 46 (#Inv=5);95CI GrowthCurves
524
1133
1552
2103
1250
Height
Degree Day
0
10
20
30
40
50
60
70
80
90
0
200
400
600
800
1000
1200

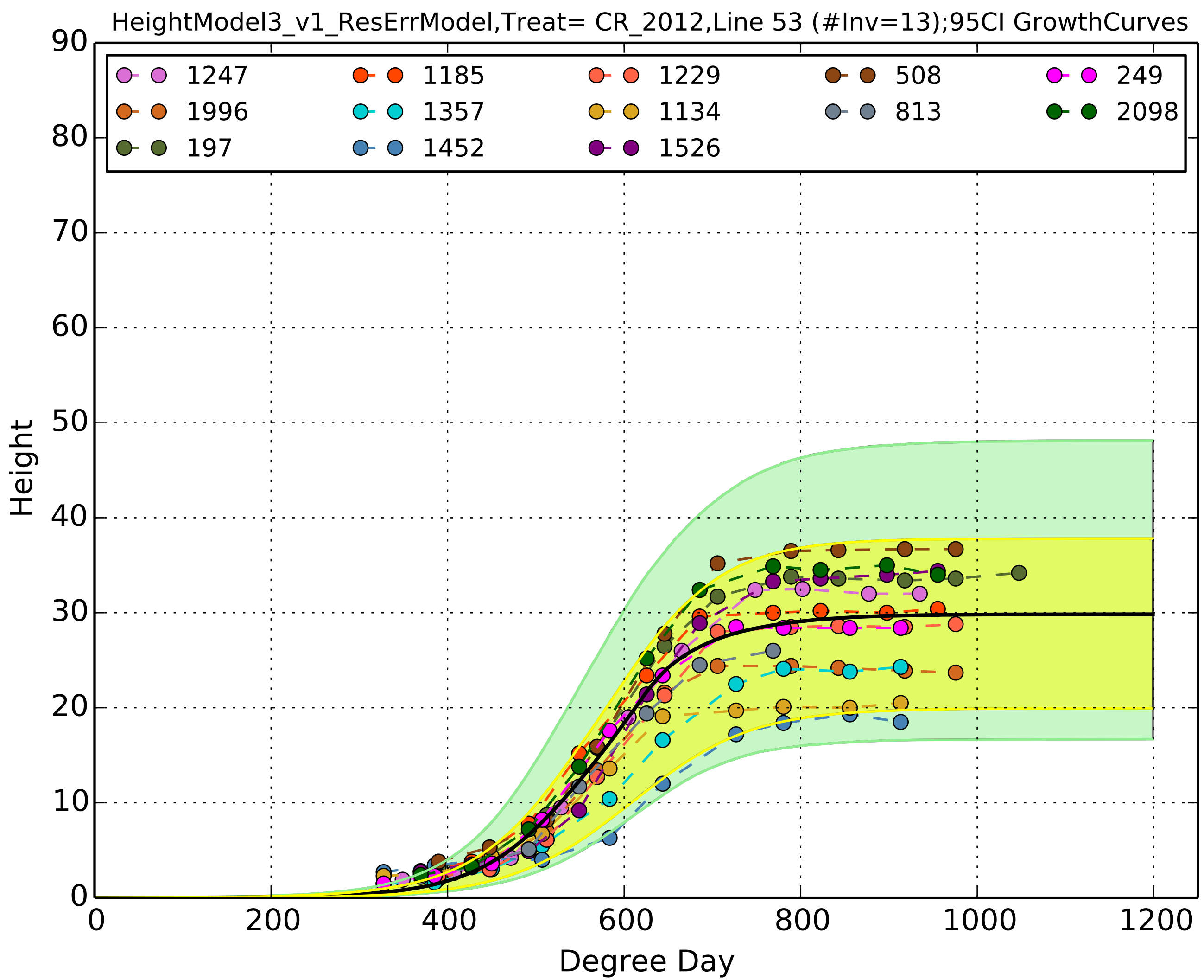

HeightModel3_v1_ResErrModel,Treat= CR_2012,Line 53 (#Inv=13);95CI GrowthCurves
1247
1185
1229
508
249
1996
1357
1134
813
2098
197
1452
1526
Height
Degree Day
0
10
20
30
40
50
60
70
80
90
0
200
400
600
800
1000
1200

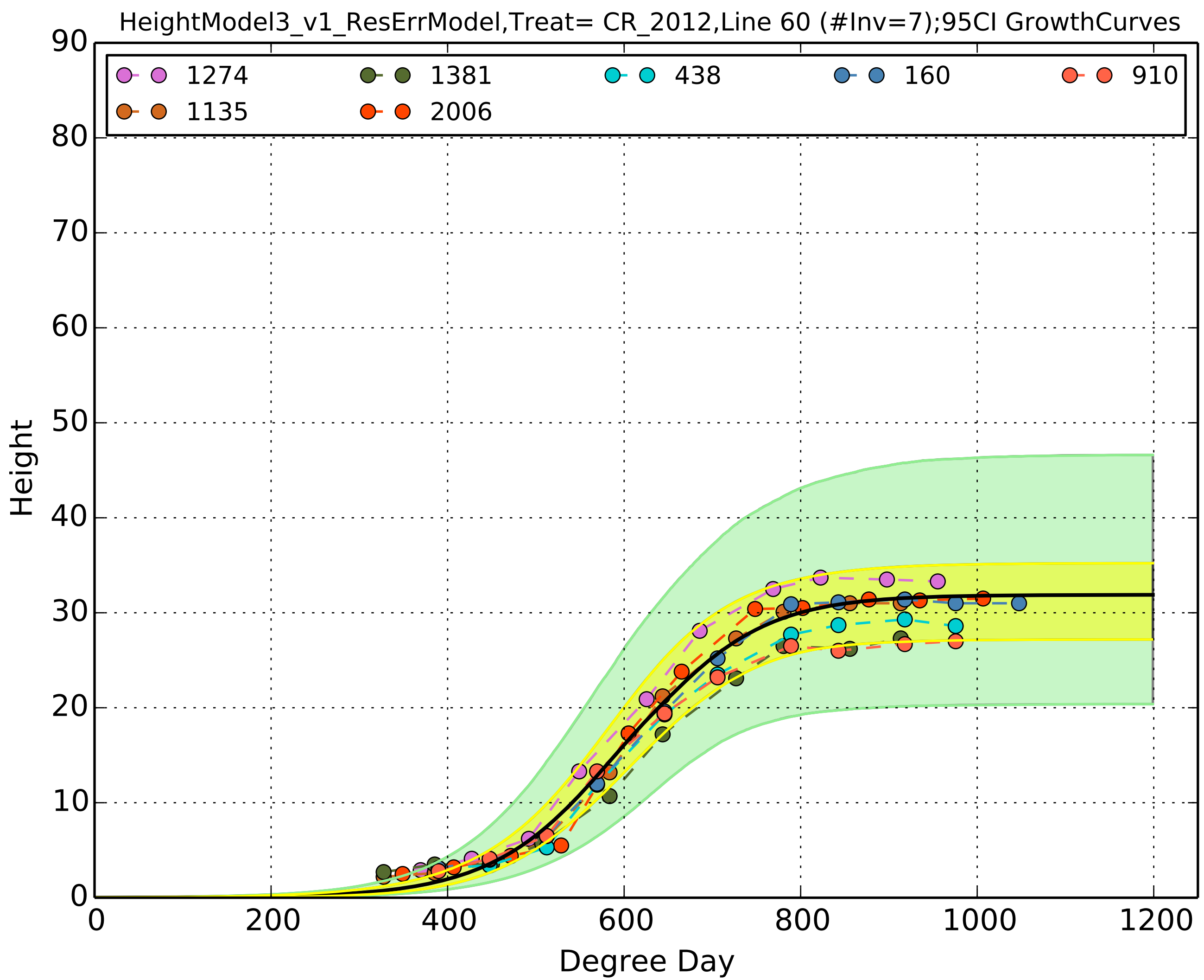

HeightModel3_v1_ResErrModel,Treat= CR_2012,Line 60 (#Inv=7);95CI GrowthCurves
1274
1381
438
160
910
1135
2006
Height
Degree Day
0
10
20
30
40
50
60
70
80
90
0
200
400
600
800
1000
1200

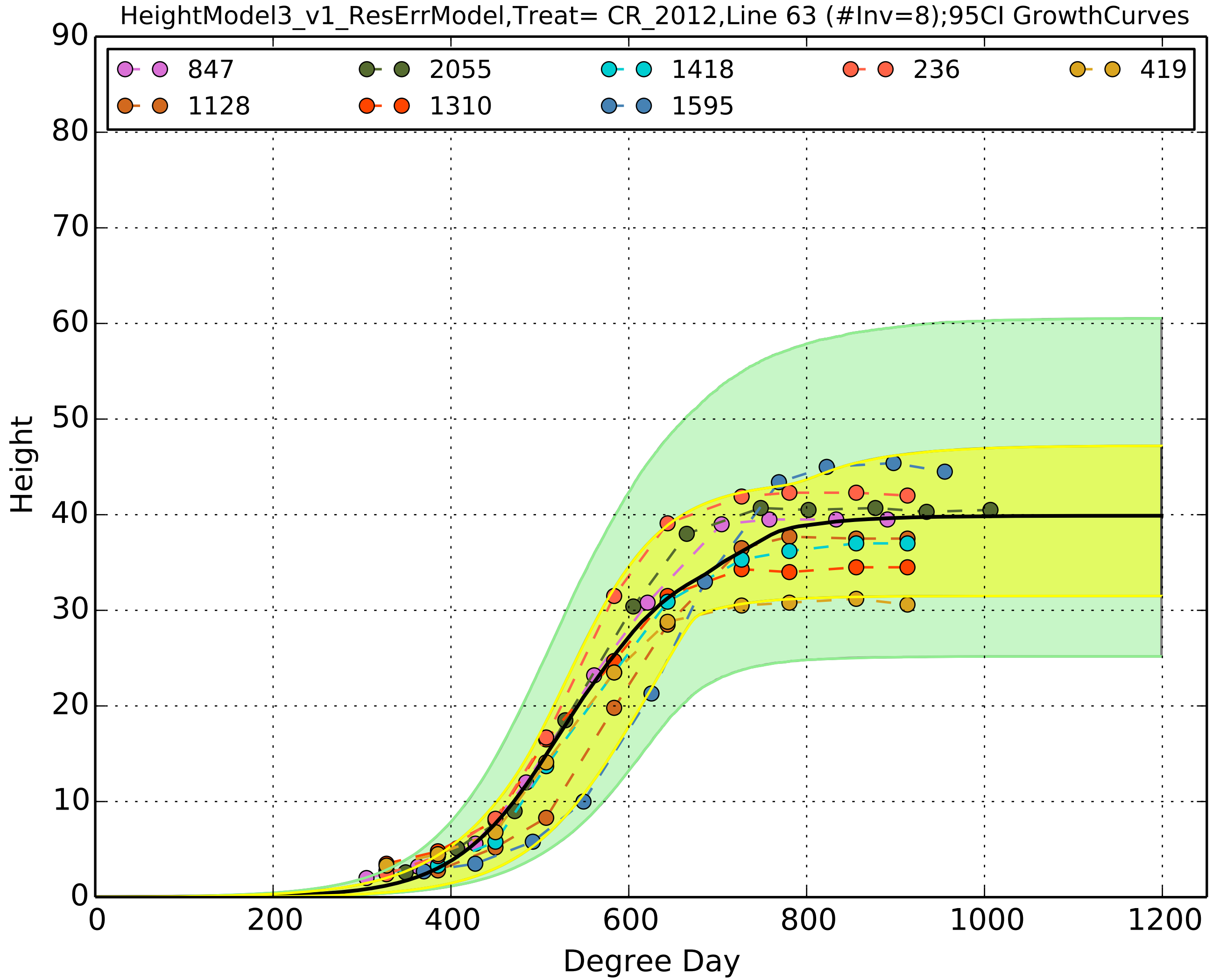
HeightModel3_v1_ResErrModel,Treat= CR_2012,Line 63 (#Inv=8);95CI GrowthCurves
847
2055
1418
236
419
1128
1310
1595
Height
Degree Day
0
10
20
30
40
50
60
70
80
90
0
200
400
600
800
1000
1200

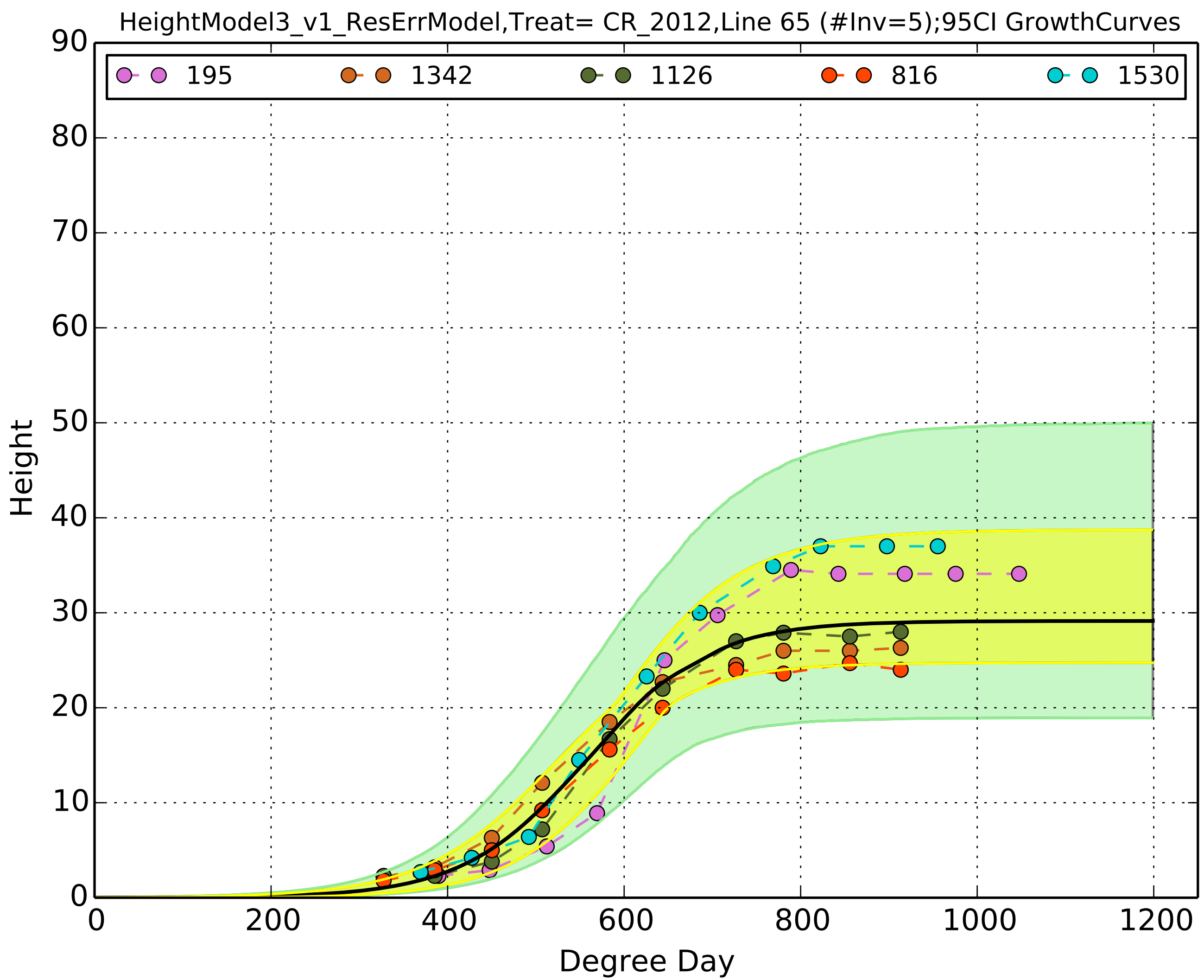

HeightModel3_v1_ResErrModel,Treat= CR_2012,Line 65 (#Inv=5);95CI GrowthCurves
195
1342
1126
816
1530
Height
Degree Day
0
10
20
30
40
50
60
70
80
90
0
200
400
600
800
1000
1200

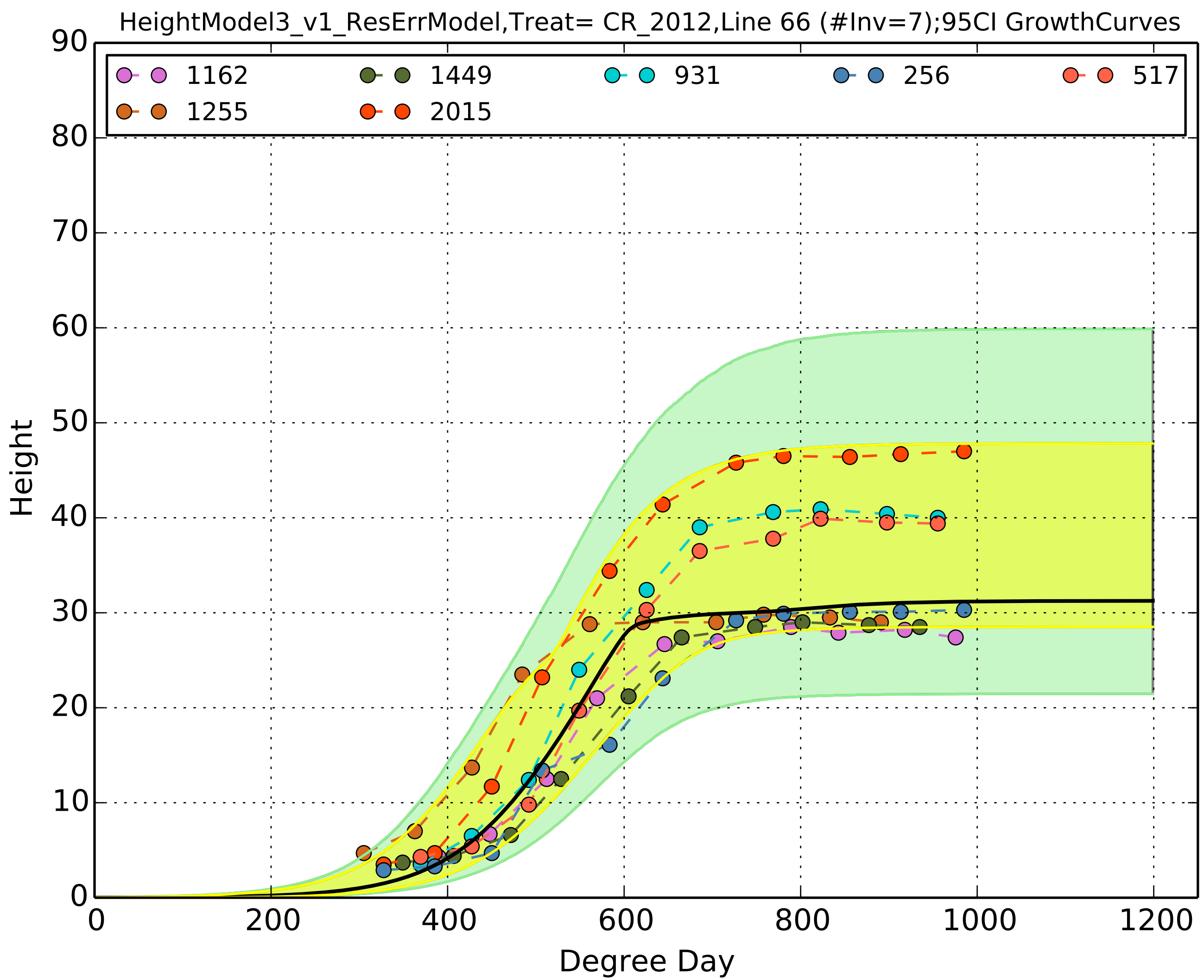

HeightModel3_v1_ResErrModel,Treat= CR_2012,Line 66 (#Inv=7);95CI GrowthCurves
1162
1449
931
256
517
1255
2015
Height
Degree Day
0
10
20
30
40
50
60
70
80
90
0
200
400
600
800
1000
1200

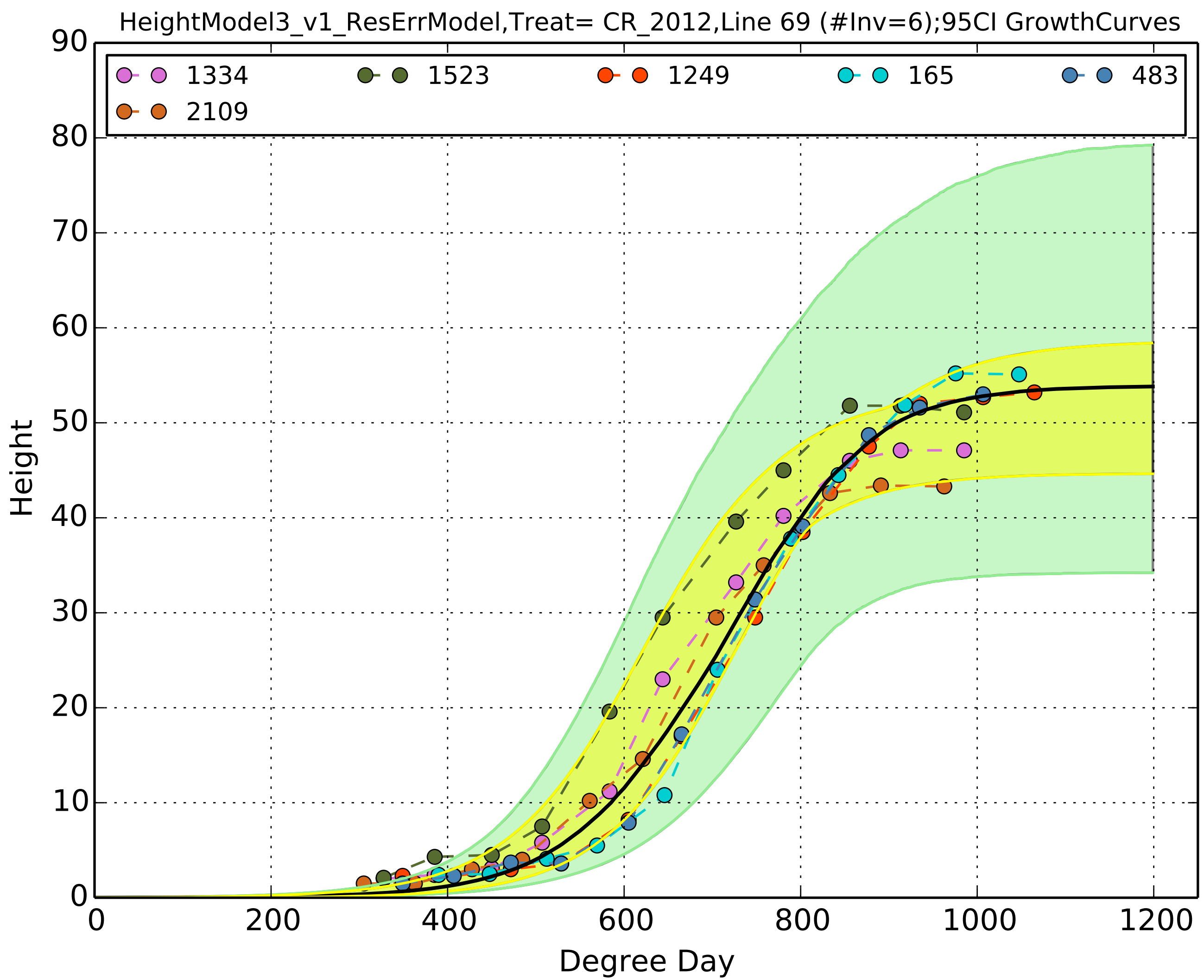

HeightModel3_v1_ResErrModel,Treat= CR_2012,Line 69 (#Inv=6);95CI GrowthCurves
1334
1523
1249
165
483
2109
Height
Degree Day
0
10
20
30
40
50
60
70
80
90
0
200
400
600
800
1000
1200

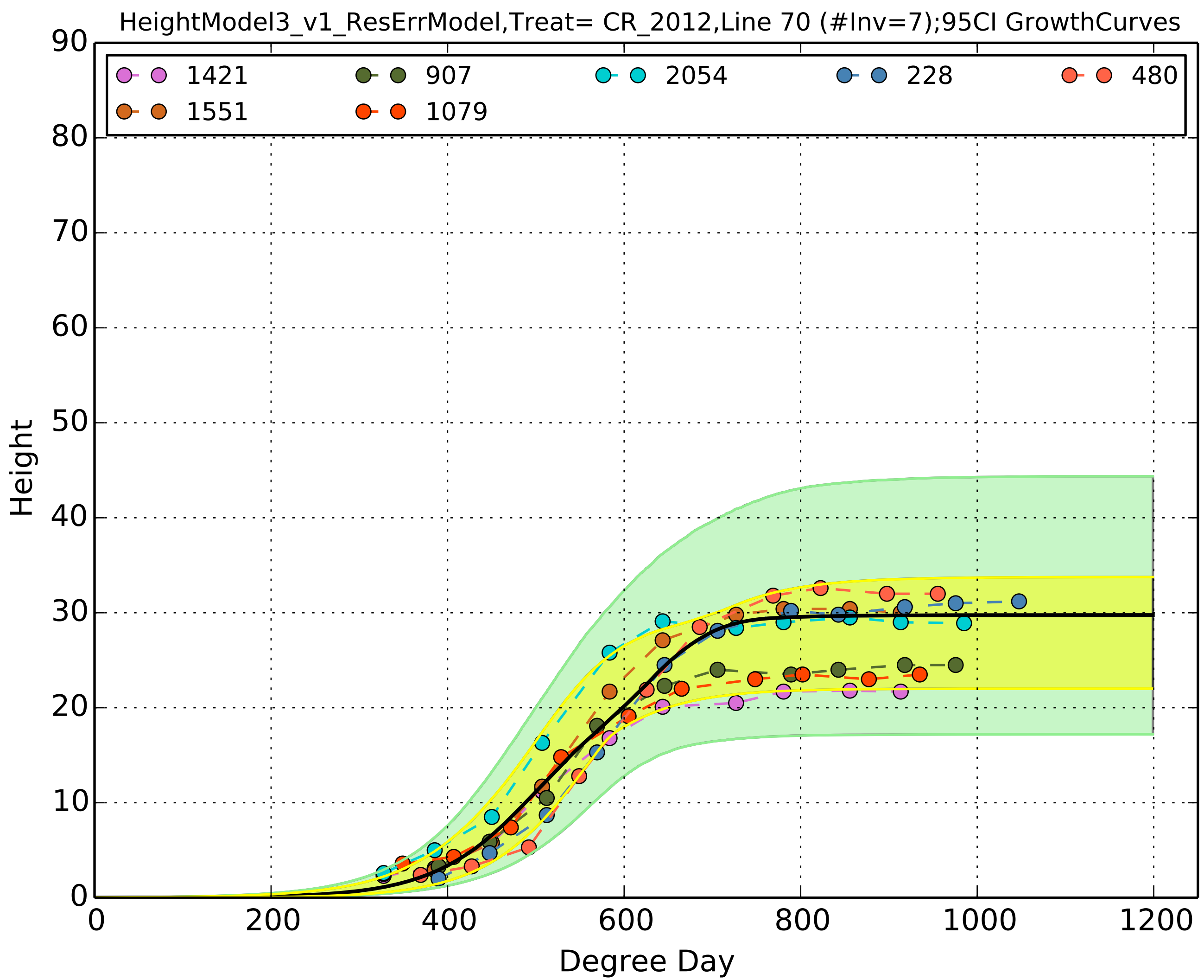

HeightModel3_v1_ResErrModel,Treat= CR_2012,Line 70 (#Inv=7);95CI GrowthCurves
1421
907
2054
228
480
1551
1079
Height
Degree Day
0
10
20
30
40
50
60
70
80
90
0
200
400
600
800
1000
1200

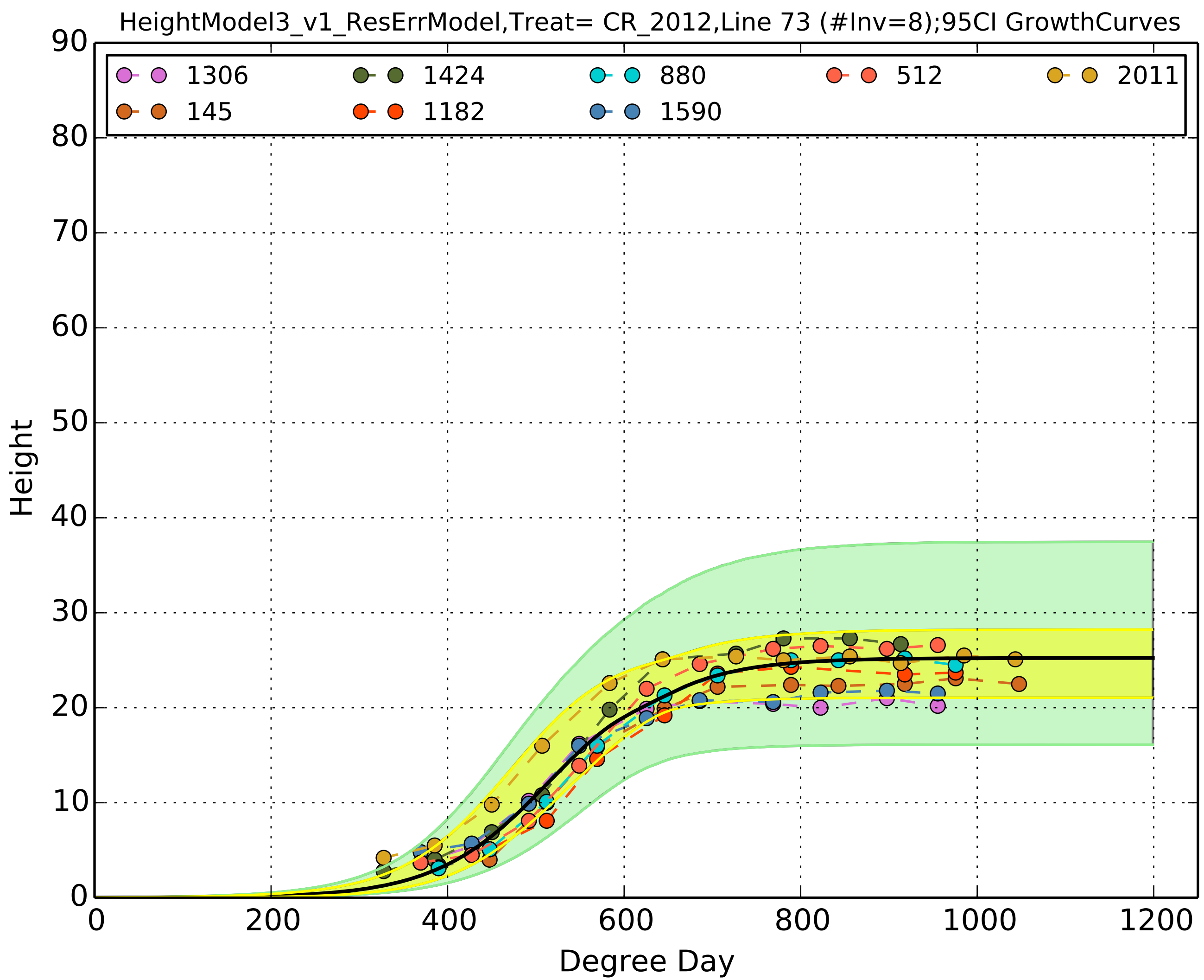

HeightModel3_v1_ResErrModel,Treat= CR_2012,Line 73 (#Inv=8);95CI GrowthCurves
1306
1424
880
512
2011
145
1182
1590
Height
Degree Day
0
10
20
30
40
50
60
70
80
90
0
200
400
600
800
1000
1200

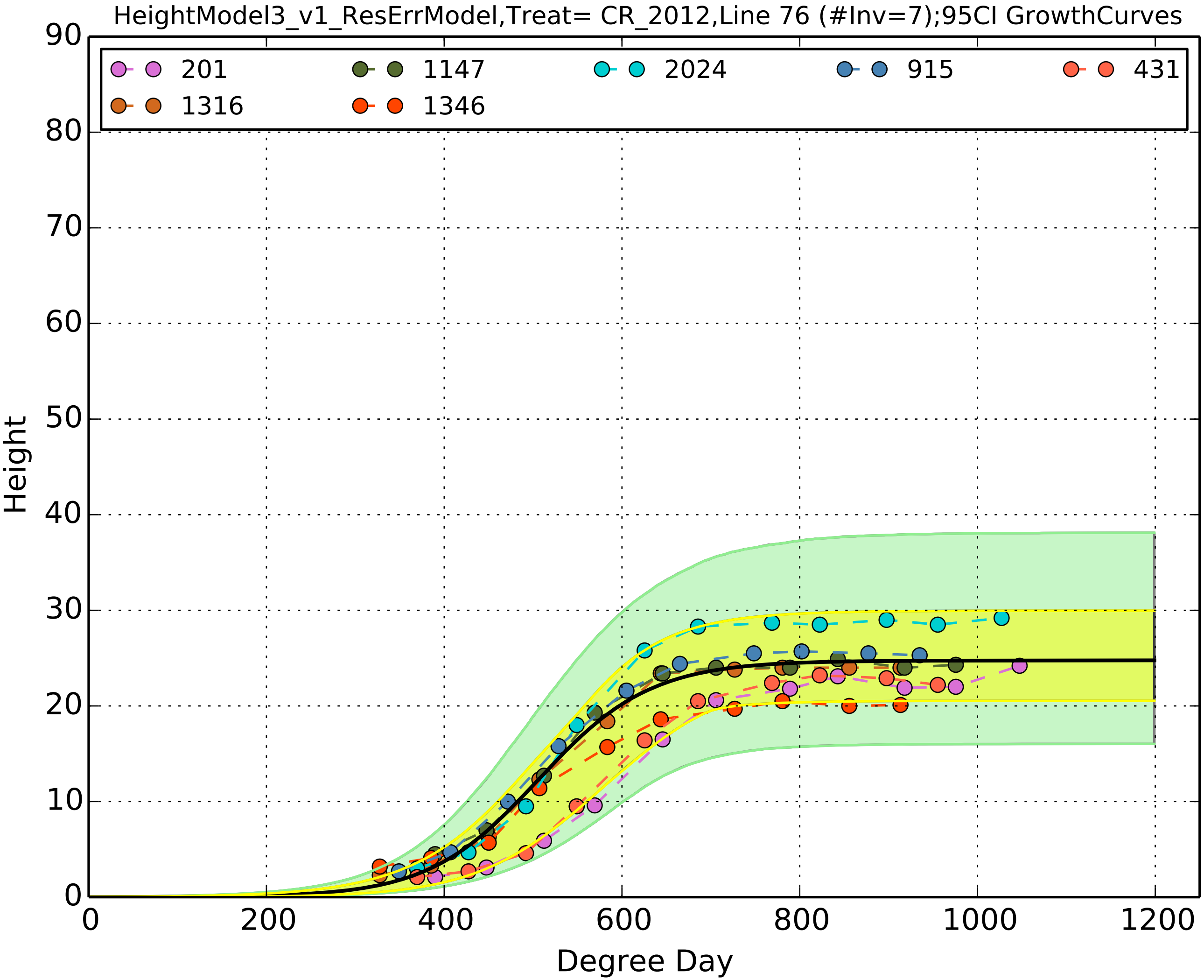
HeightModel3_v1_ResErrModel,Treat= CR_2012,Line 76 (#Inv=7);95CI GrowthCurves
201
1147
2024
915
431
1316
1346
Height
Degree Day
0
10
20
30
40
50
60
70
80
90
0
200
400
600
800
1000
1200

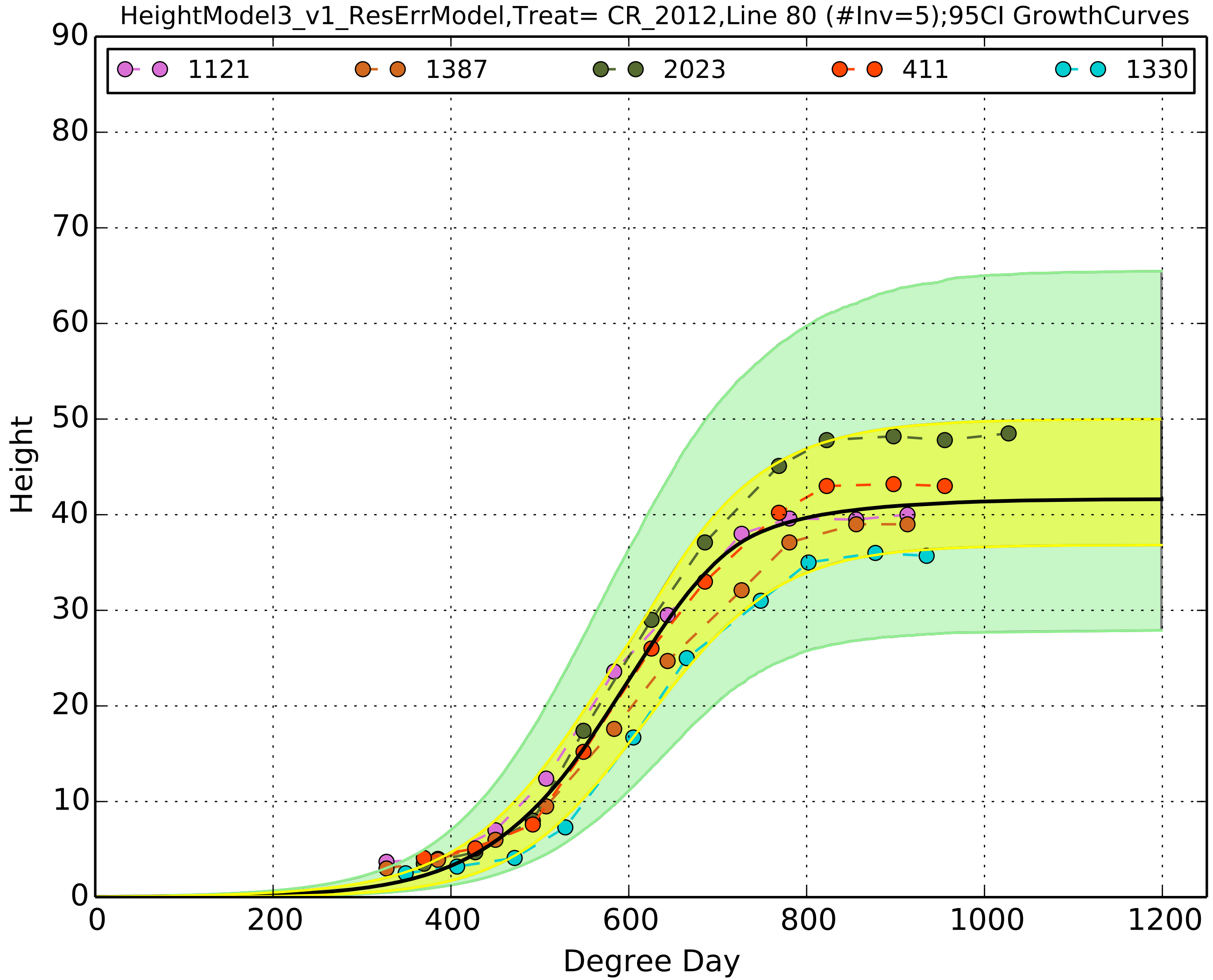

HeightModel3_v1_ResErrModel,Treat= CR_2012,Line 80 (#Inv=5);95CI GrowthCurves
1121
1387
2023
411
1330
Height
Degree Day
0
10
20
30
40
50
60
70
80
90
0
200
400
600
800
1000
1200

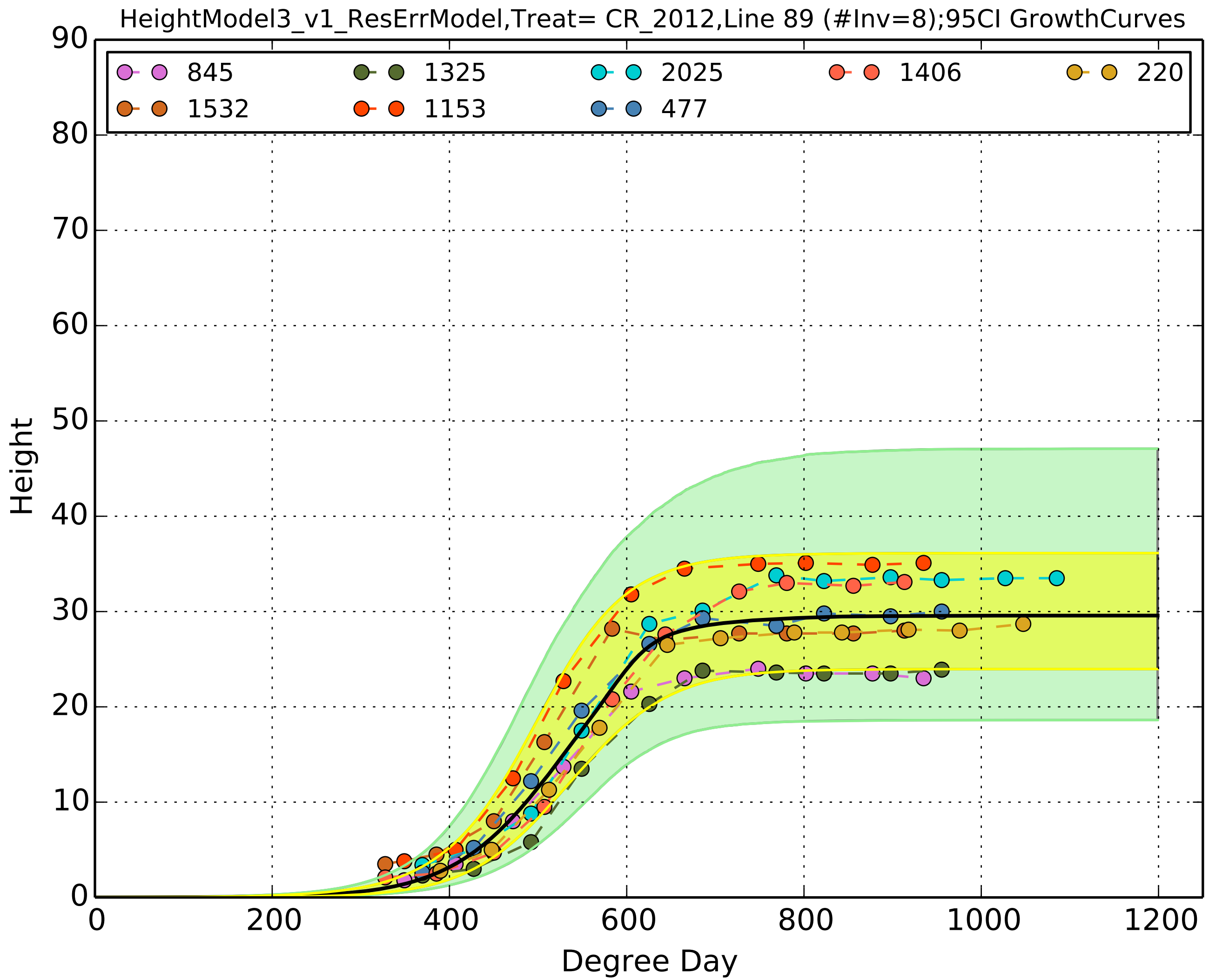

HeightModel3_v1_ResErrModel,Treat= CR_2012,Line 89 (#Inv=8);95CI GrowthCurves
845
1325
2025
1406
220
1532
1153
477
Height
Degree Day
0
10
20
30
40
50
60
70
80
90
0
200
400
600
800
1000
1200

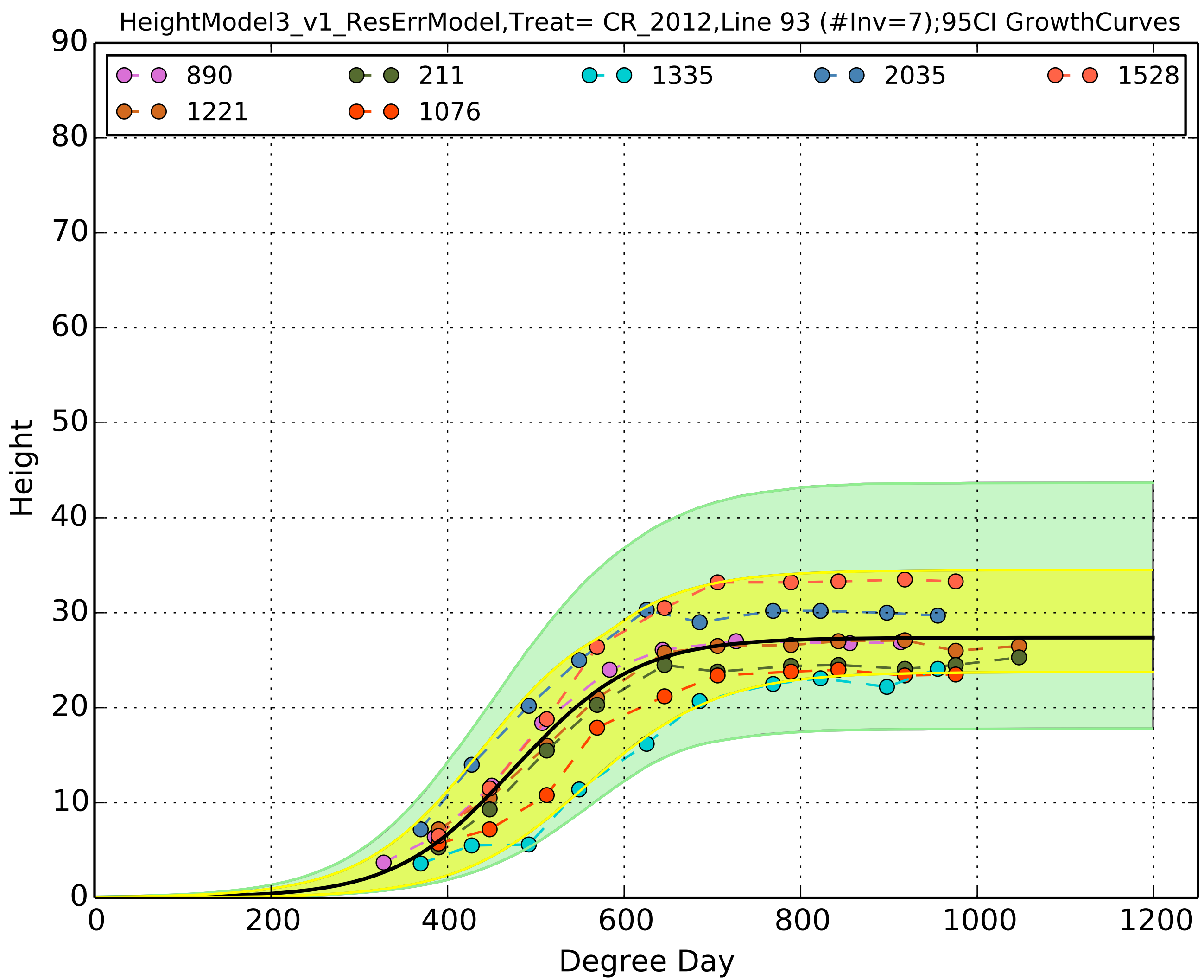

HeightModel3_v1_ResErrModel,Treat= CR_2012,Line 93 (#Inv=7);95CI GrowthCurves
890
211
1335
2035
1528
1221
1076
Height
Degree Day
0
10
20
30
40
50
60
70
80
90
0
200
400
600
800
1000
1200

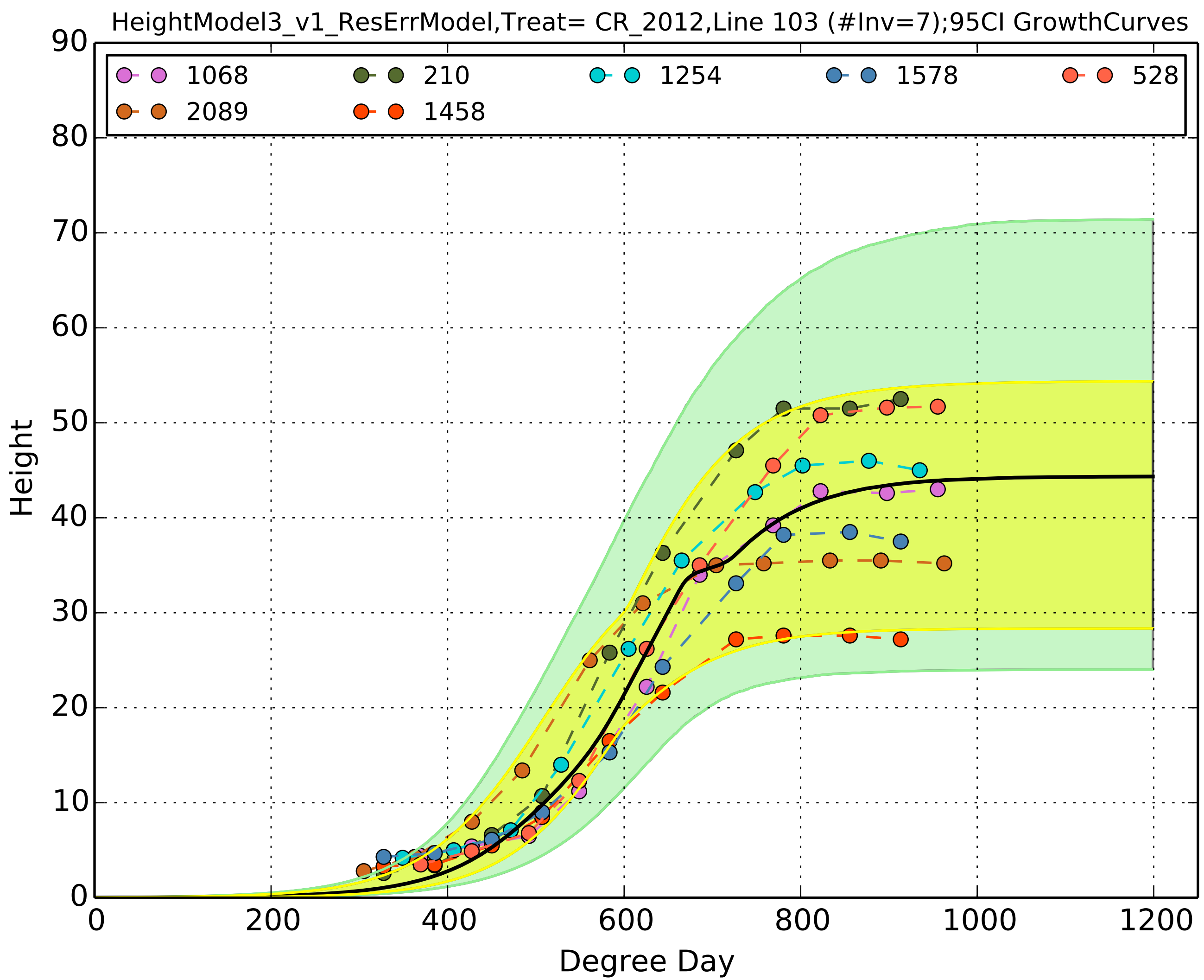

HeightModel3_v1_ResErrModel,Treat= CR_2012,Line 103 (#Inv=7);95CI GrowthCurves
1068
210
1254
1578
528
2089
1458
Height
Degree Day
0
10
20
30
40
50
60
70
80
90
0
200
400
600
800
1000
1200

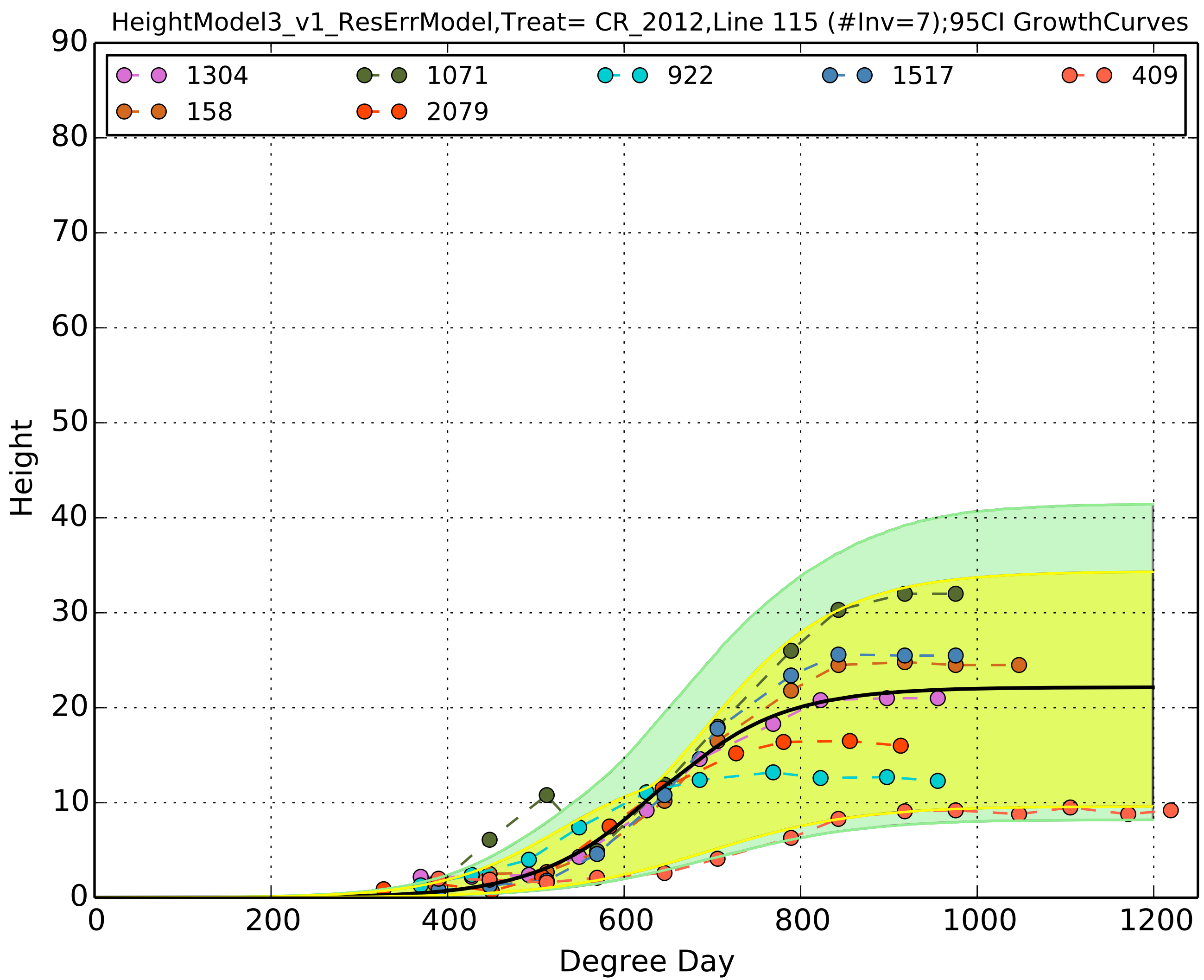

HeightModel3_v1_ResErrModel,Treat= CR_2012,Line 115 (#Inv=7);95CI GrowthCurves
1304
1071
922
1517
409
158
2079
Height
Degree Day
0
10
20
30
40
50
60
70
80
90
0
200
400
600
800
1000
1200

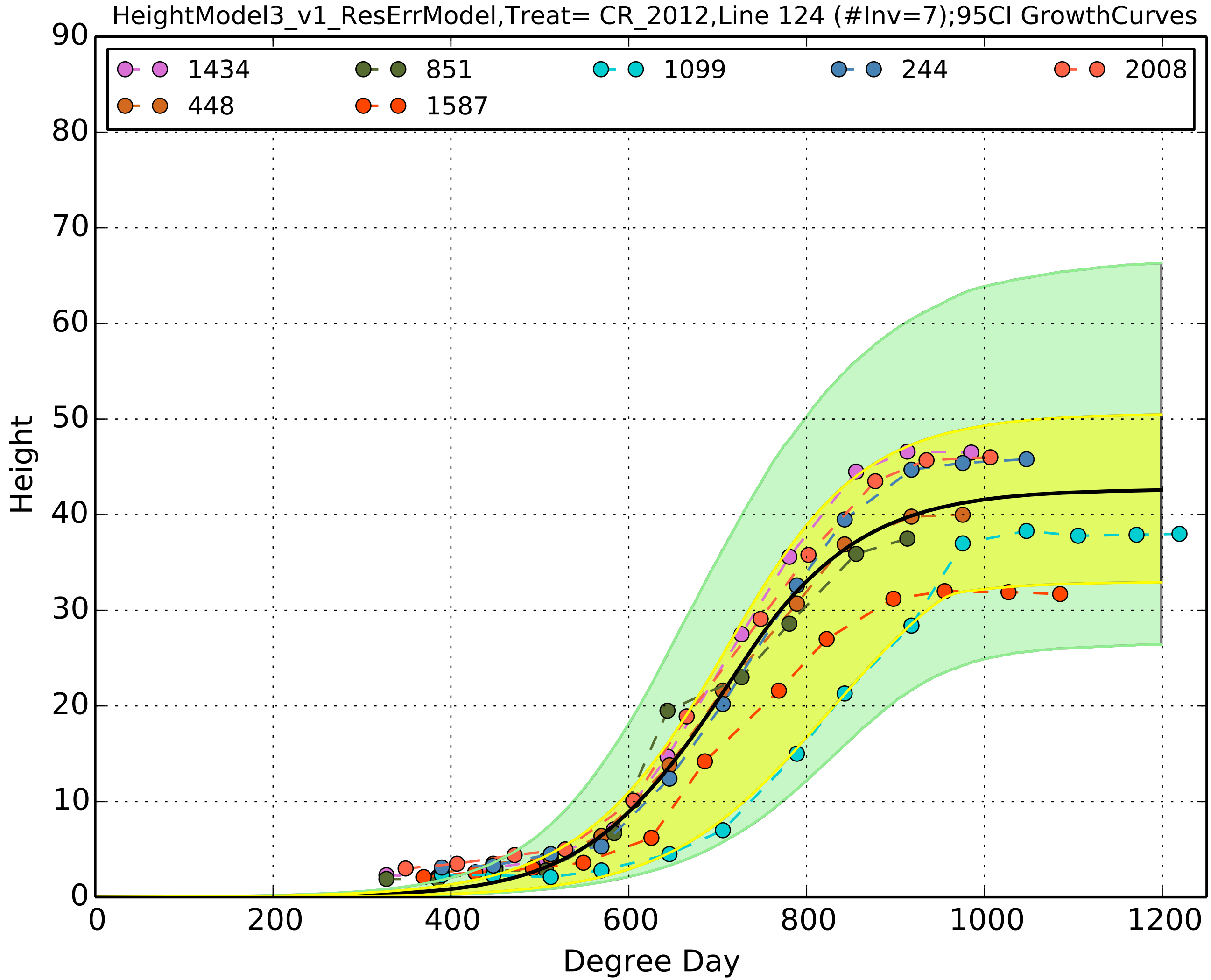

HeightModel3_v1_ResErrModel,Treat= CR_2012,Line 124 (#Inv=7);95CI GrowthCurves
1434
851
1099
244
2008
448
1587
Height
Degree Day
0
10
20
30
40
50
60
70
80
90
0
200
400
600
800
1000
1200

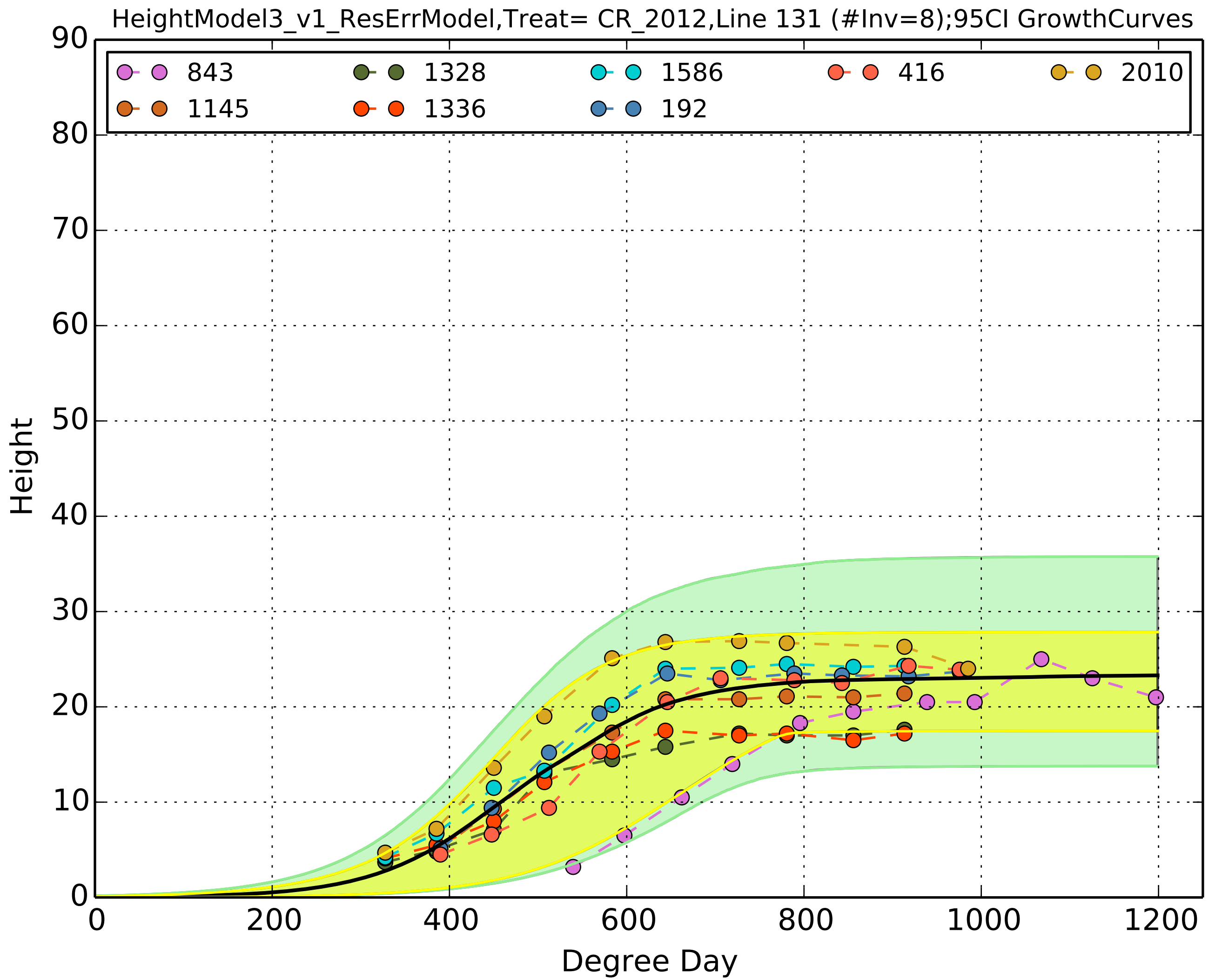

HeightModel3_v1_ResErrModel,Treat= CR_2012,Line 131 (#Inv=8);95CI GrowthCurves
843
1328
1586
416
2010
1145
1336
192
Height
Degree Day
0
10
20
30
40
50
60
70
80
90
0
200
400
600
800
1000
1200

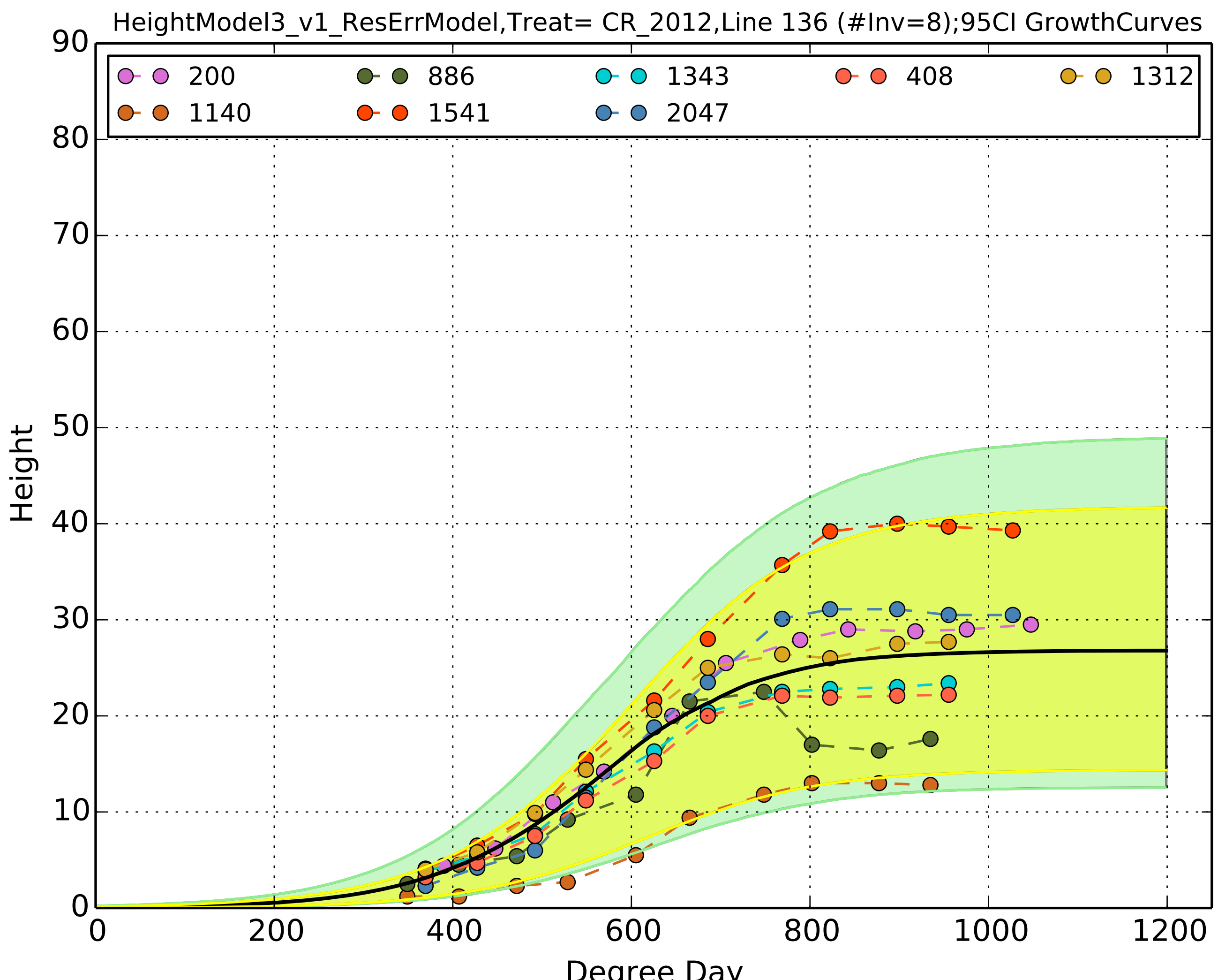

HeightModel3_v1_ResErrModel,Treat= CR_2012,Line 136 (#Inv=8);95CI GrowthCurves
200
886
1343
408
1312
1140
1541
2047
Height
Degree Day
0
10
20
30
40
50
60
70
80
90
0
200
400
600
800
1000
1200

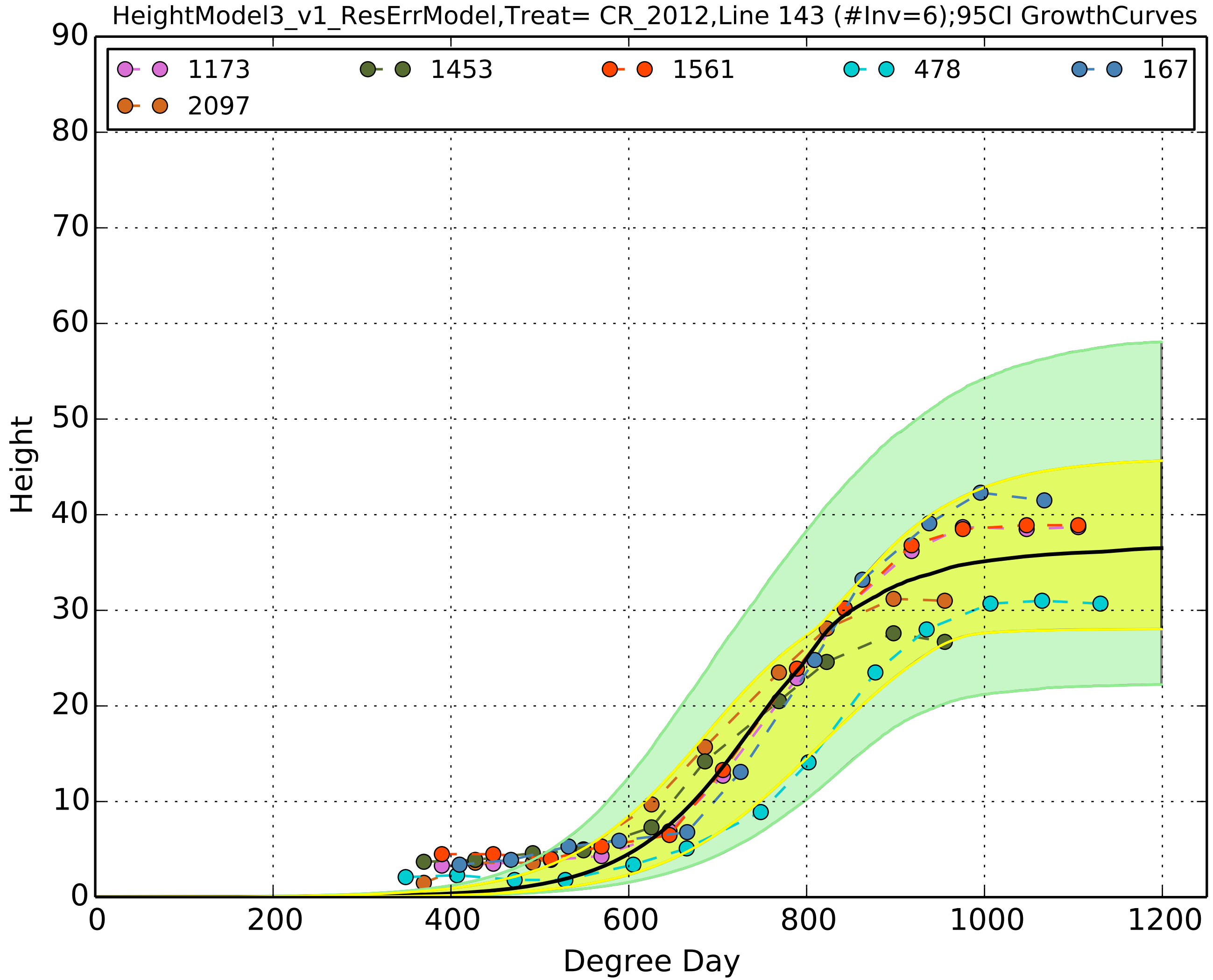
HeightModel3_v1_ResErrModel,Treat= CR_2012,Line 143 (#Inv=6);95CI GrowthCurves
1173
1453
1561
478
167
2097
Height
Degree Day
0
10
20
30
40
50
60
70
80
90
0
200
400
600
800
1000
1200

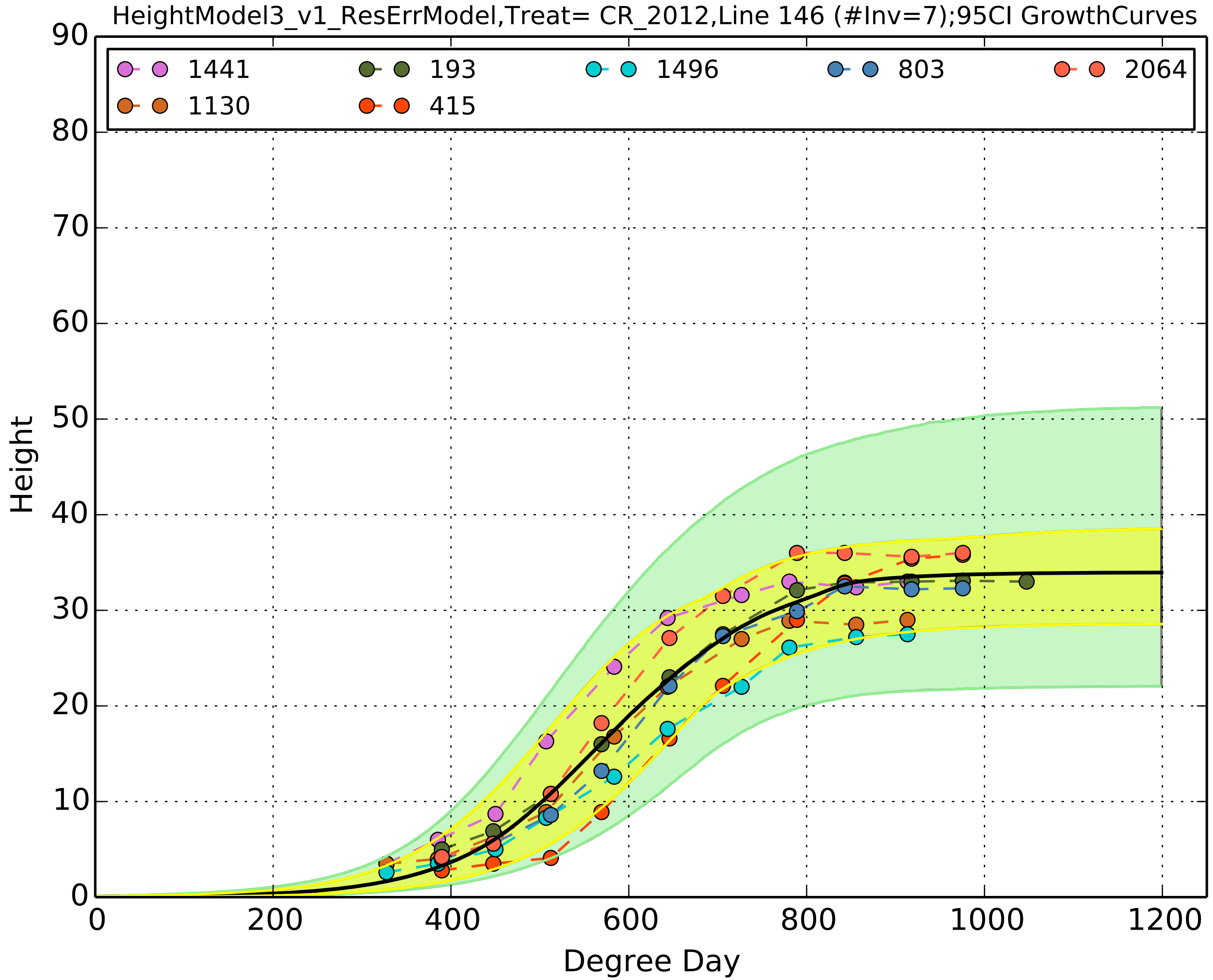

HeightModel3_v1_ResErrModel,Treat= CR_2012,Line 146 (#Inv=7);95CI GrowthCurves
1441
193
1496
803
2064
1130
415
Height
Degree Day
0
10
20
30
40
50
60
70
80
90
0
200
400
600
800
1000
1200

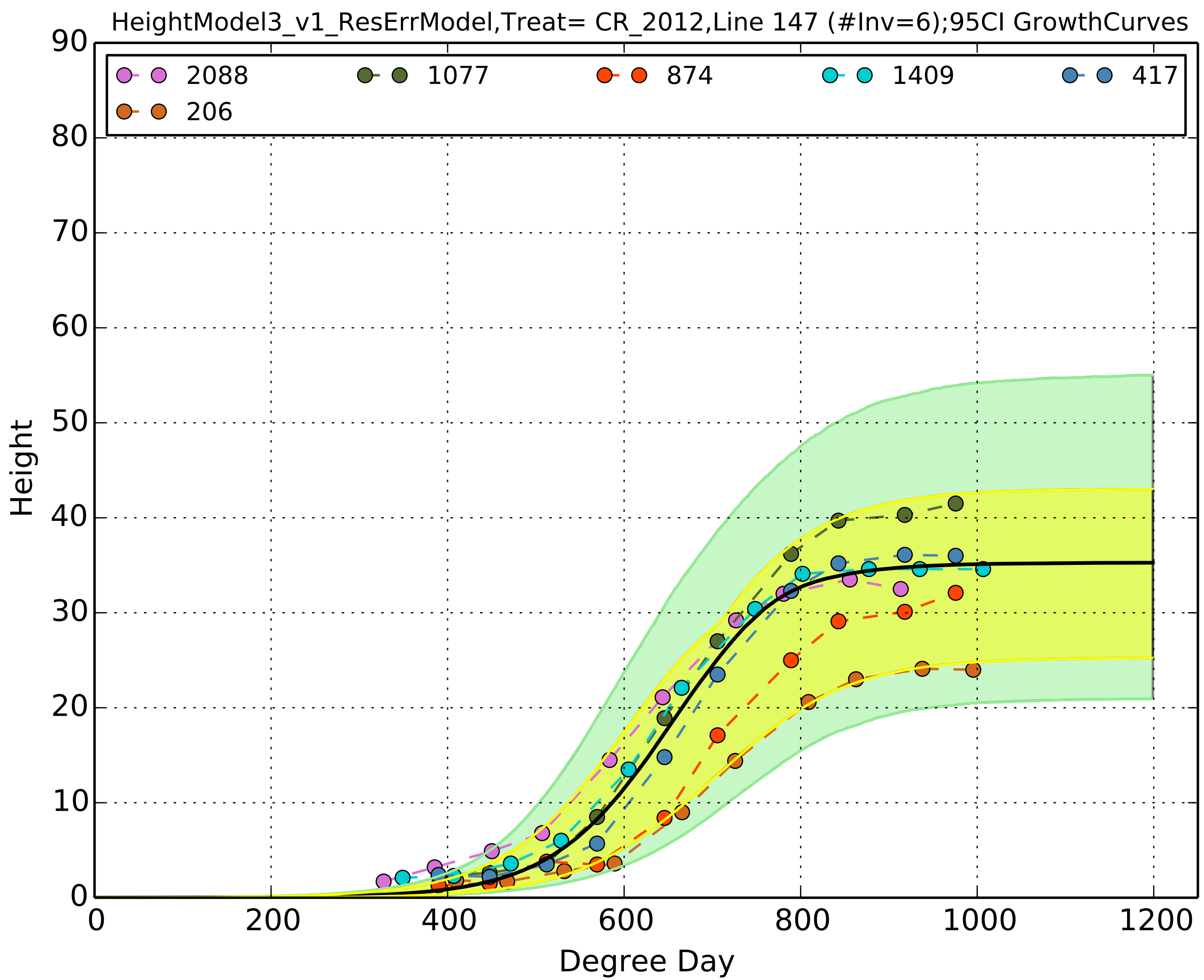

HeightModel3_v1_ResErrModel,Treat= CR_2012,Line 147 (#Inv=6);95CI GrowthCurves
2088
1077
874
1409
417
206
Height
Degree Day
0
10
20
30
40
50
60
70
80
90
0
200
400
600
800
1000
1200

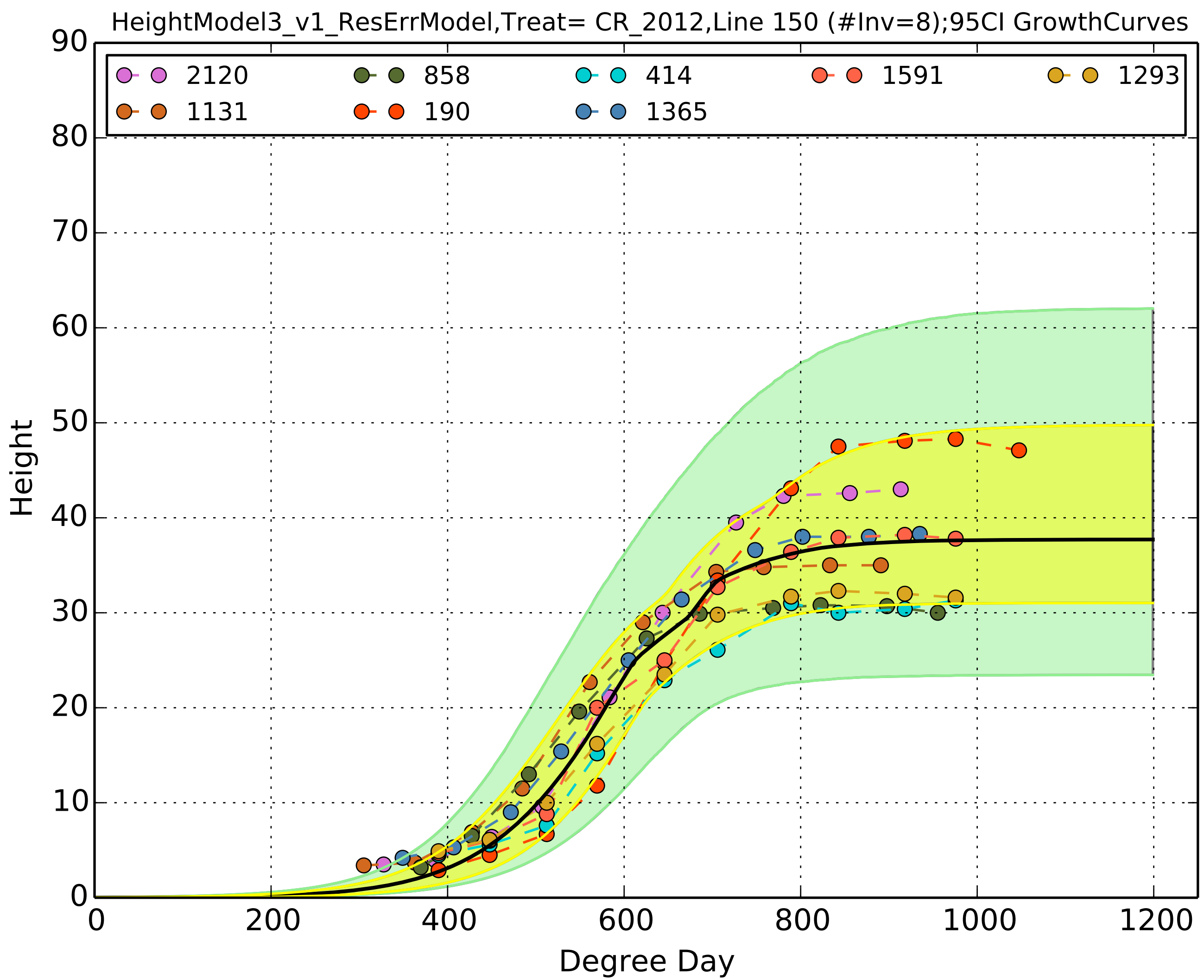

HeightModel3_v1_ResErrModel,Treat= CR_2012,Line 150 (#Inv=8);95CI GrowthCurves
2120
858
414
1591
1293
1131
190
1365
Height
Degree Day
0
10
20
30
40
50
60
70
80
90
0
200
400
600
800
1000
1200

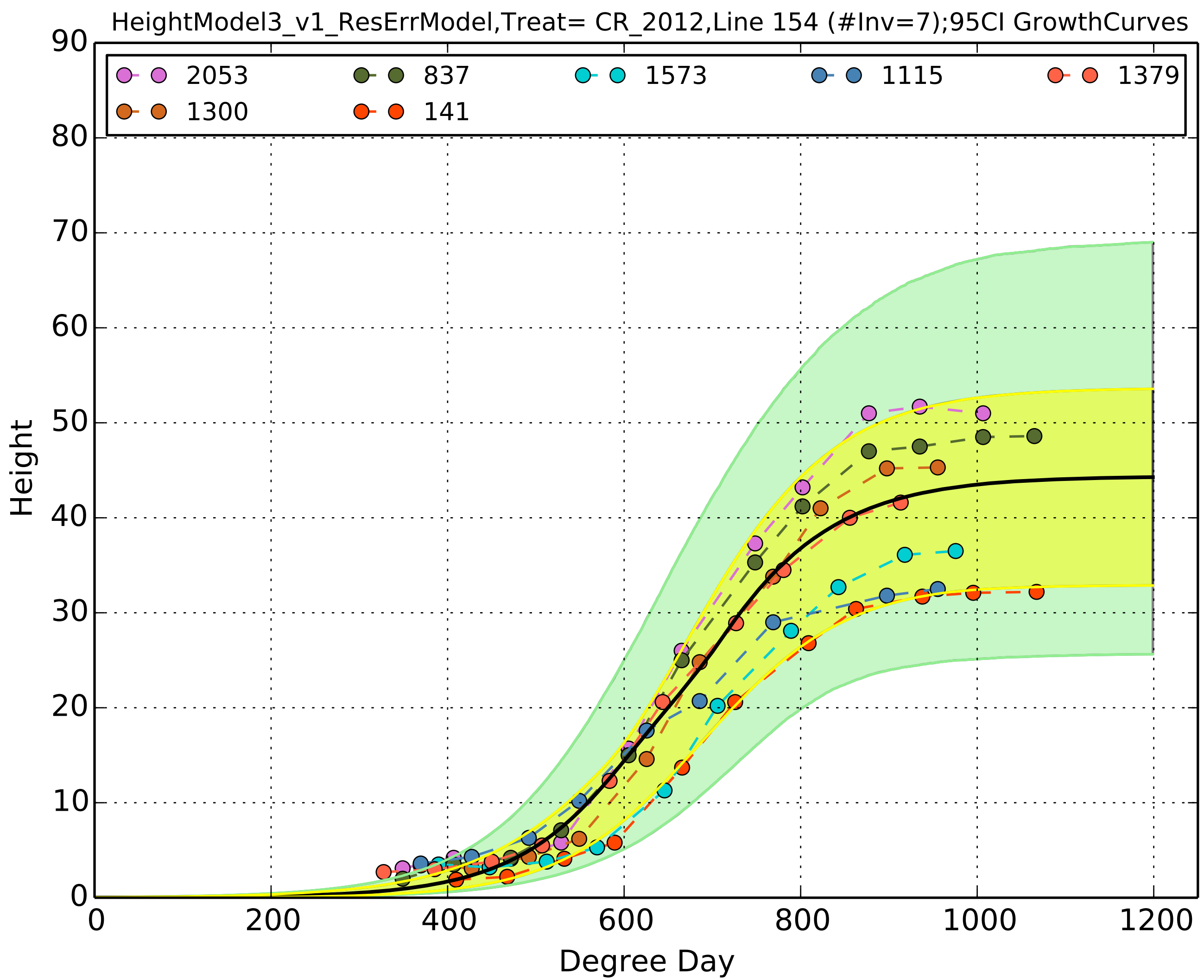
HeightModel3_v1_ResErrModel,Treat= CR_2012,Line 154 (#Inv=7);95CI GrowthCurves
2053
837
1573
1115
1379
1300
141
Height
Degree Day
0
10
20
30
40
50
60
70
80
90
0
200
400
600
800
1000
1200

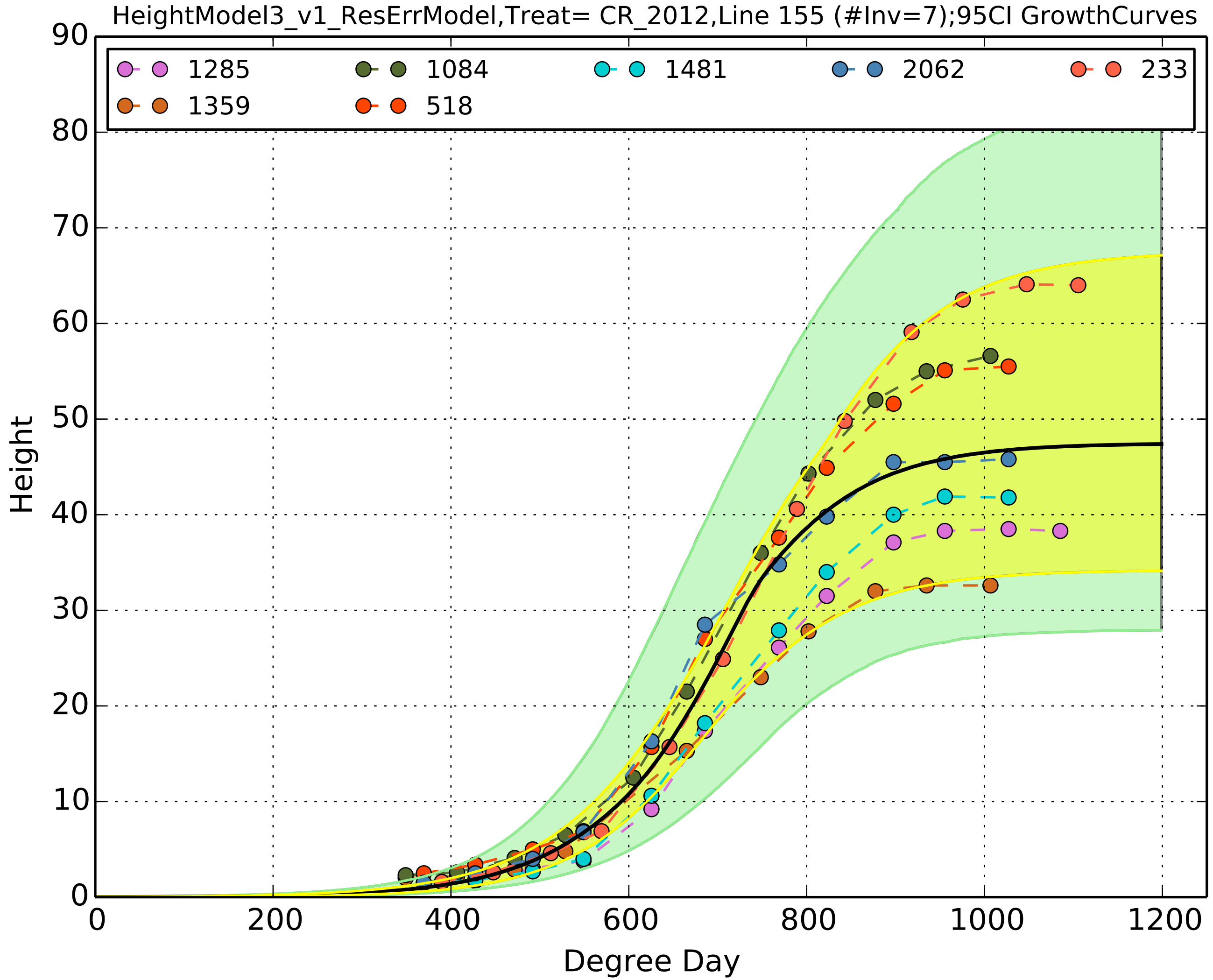

HeightModel3_v1_ResErrModel,Treat= CR_2012,Line 155 (#Inv=7);95CI GrowthCurves
1285
1084
1481
2062
233
1359
518
Height
Degree Day
0
10
20
30
40
50
60
70
80
90
0
200
400
600
800
1000
1200

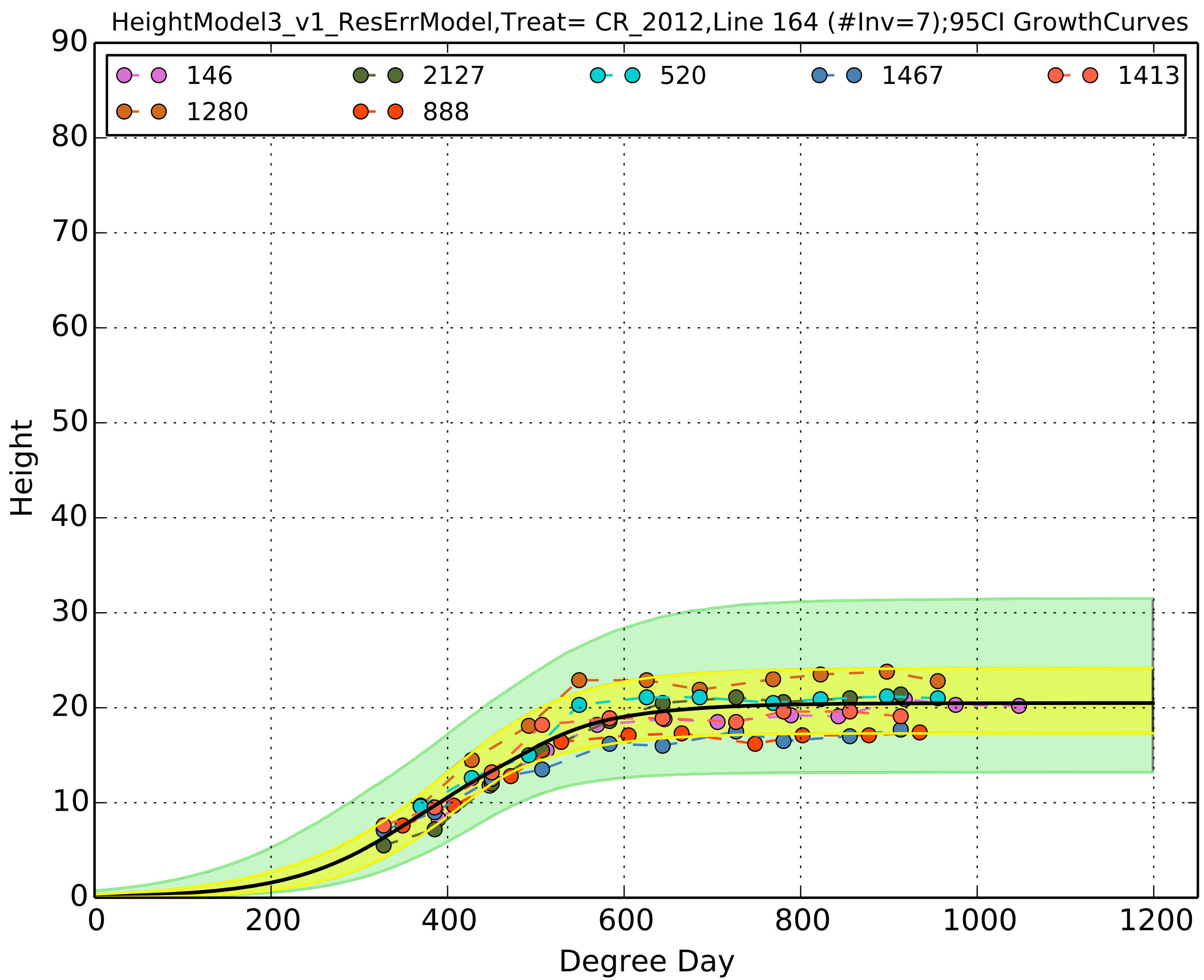
HeightModel3_v1_ResErrModel,Treat= CR_2012,Line 164 (#Inv=7);95CI GrowthCurves
146
2127
520
1467
1413
1280
888
Height
Degree Day
0
10
20
30
40
50
60
70
80
90
0
200
400
600
800
1000
1200

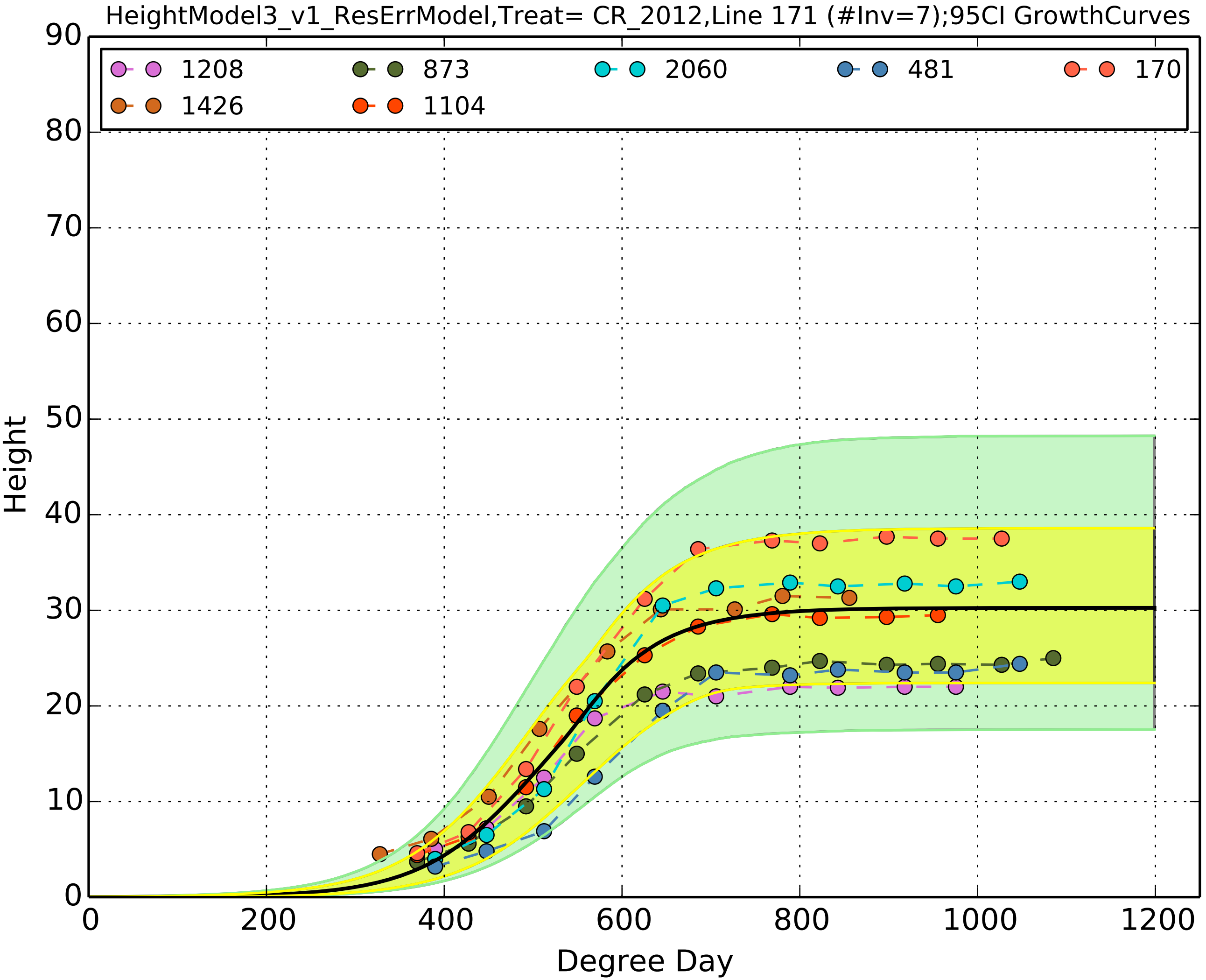
HeightModel3_v1_ResErrModel,Treat= CR_2012,Line 171 (#Inv=7);95CI GrowthCurves
1208
873
2060
481
170
1426
1104
Height
Degree Day
0
10
20
30
40
50
60
70
80
90
0
200
400
600
800
1000
1200

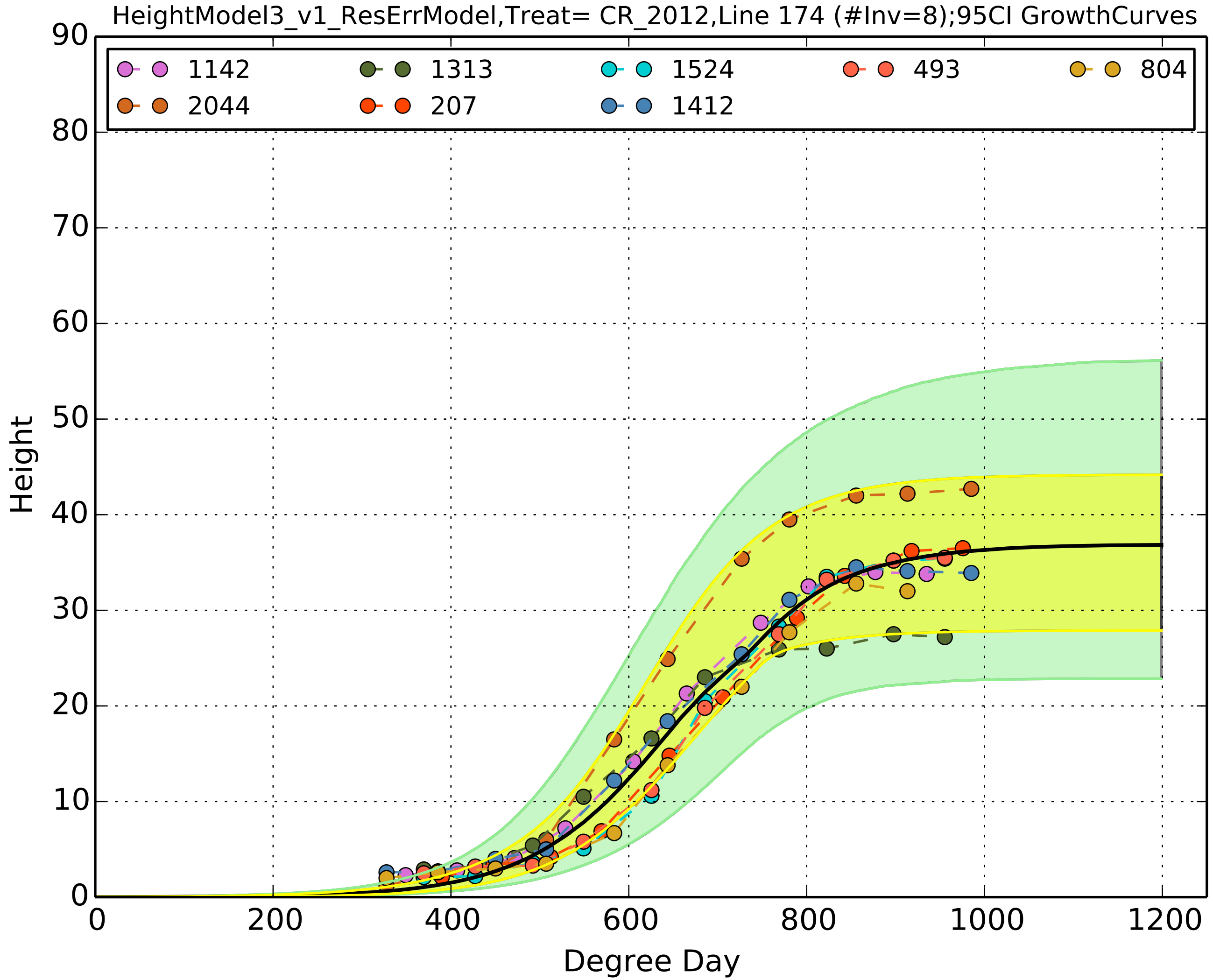

HeightModel3_v1_ResErrModel,Treat= CR_2012,Line 174 (#Inv=8);95CI GrowthCurves
1142
1313
1524
493
804
2044
207
1412
Height
Degree Day
0
10
20
30
40
50
60
70
80
90
0
200
400
600
800
1000
1200

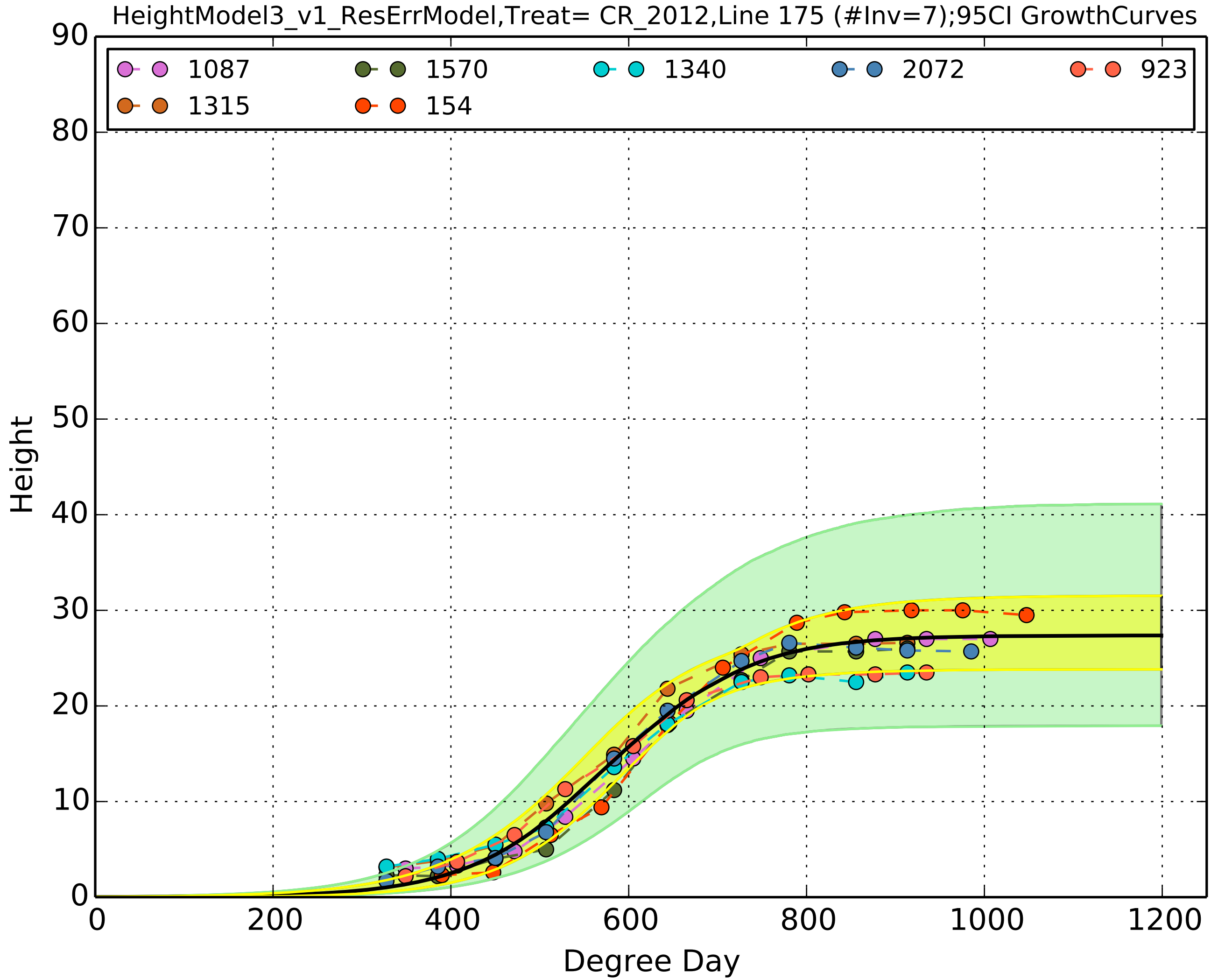

HeightModel3_v1_ResErrModel,Treat= CR_2012,Line 175 (#Inv=7);95CI GrowthCurves
1087
1570
1340
2072
923
1315
154
Height
Degree Day
0
10
20
30
40
50
60
70
80
90
0
200
400
600
800
1000
1200

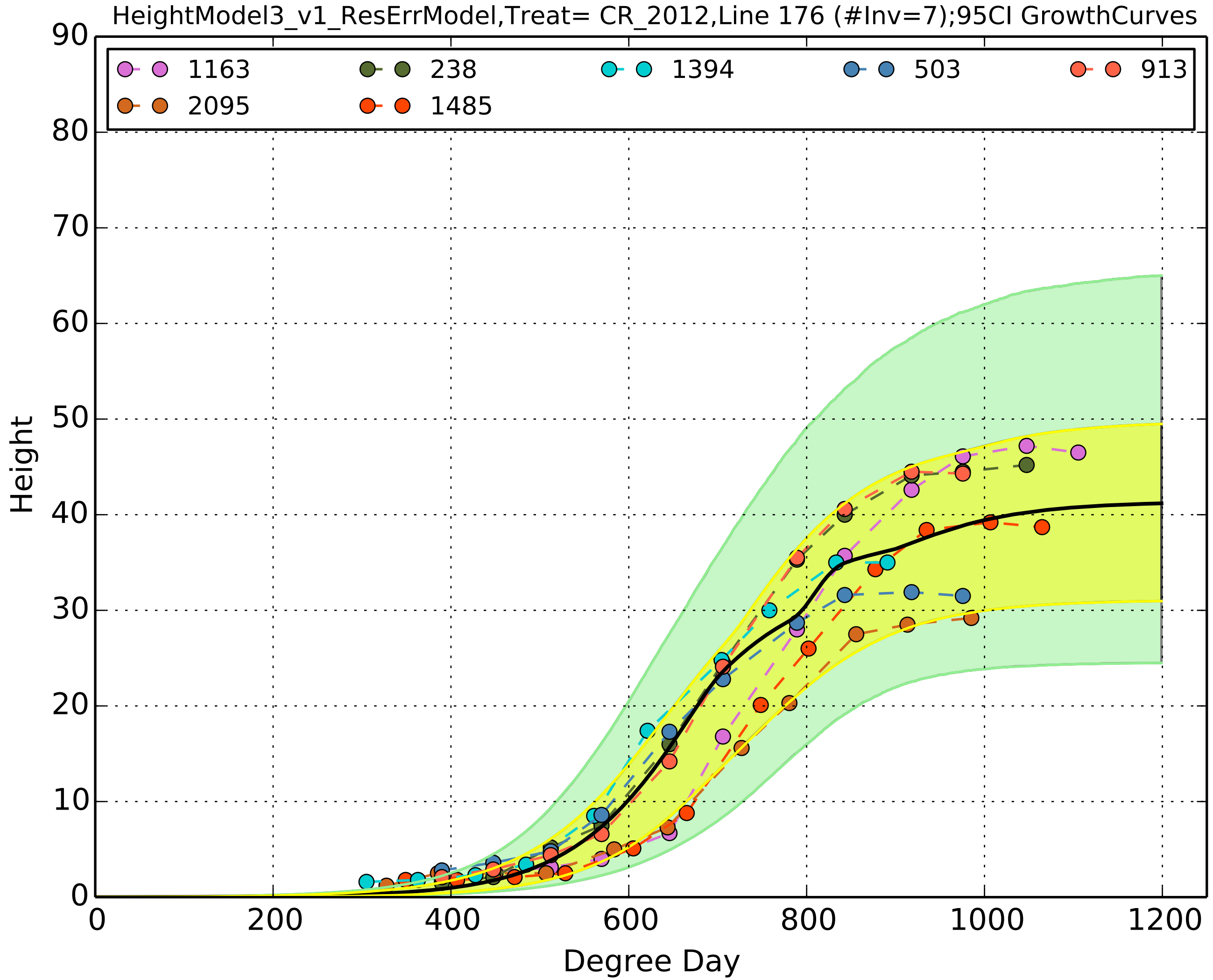
HeightModel3_v1_ResErrModel,Treat= CR_2012,Line 176 (#Inv=7);95CI GrowthCurves
1163
238
1394
503
913
2095
1485
Height
Degree Day
0
10
20
30
40
50
60
70
80
90
0
200
400
600
800
1000
1200

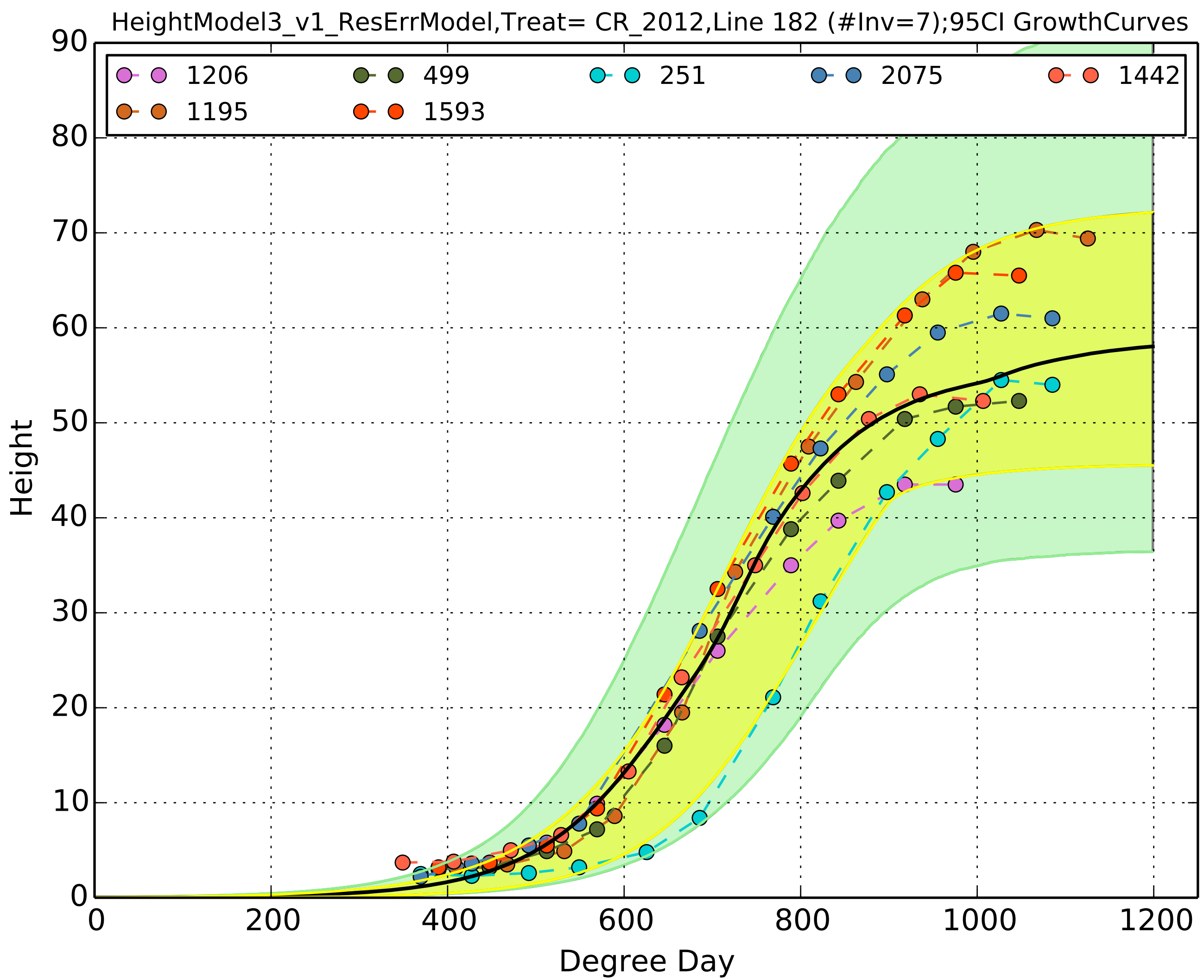

HeightModel3_v1_ResErrModel,Treat= CR_2012,Line 182 (#Inv=7);95CI GrowthCurves
1206
499
251
2075
1442
1195
1593
Height
Degree Day
0
10
20
30
40
50
60
70
80
90
0
200
400
600
800
1000
1200

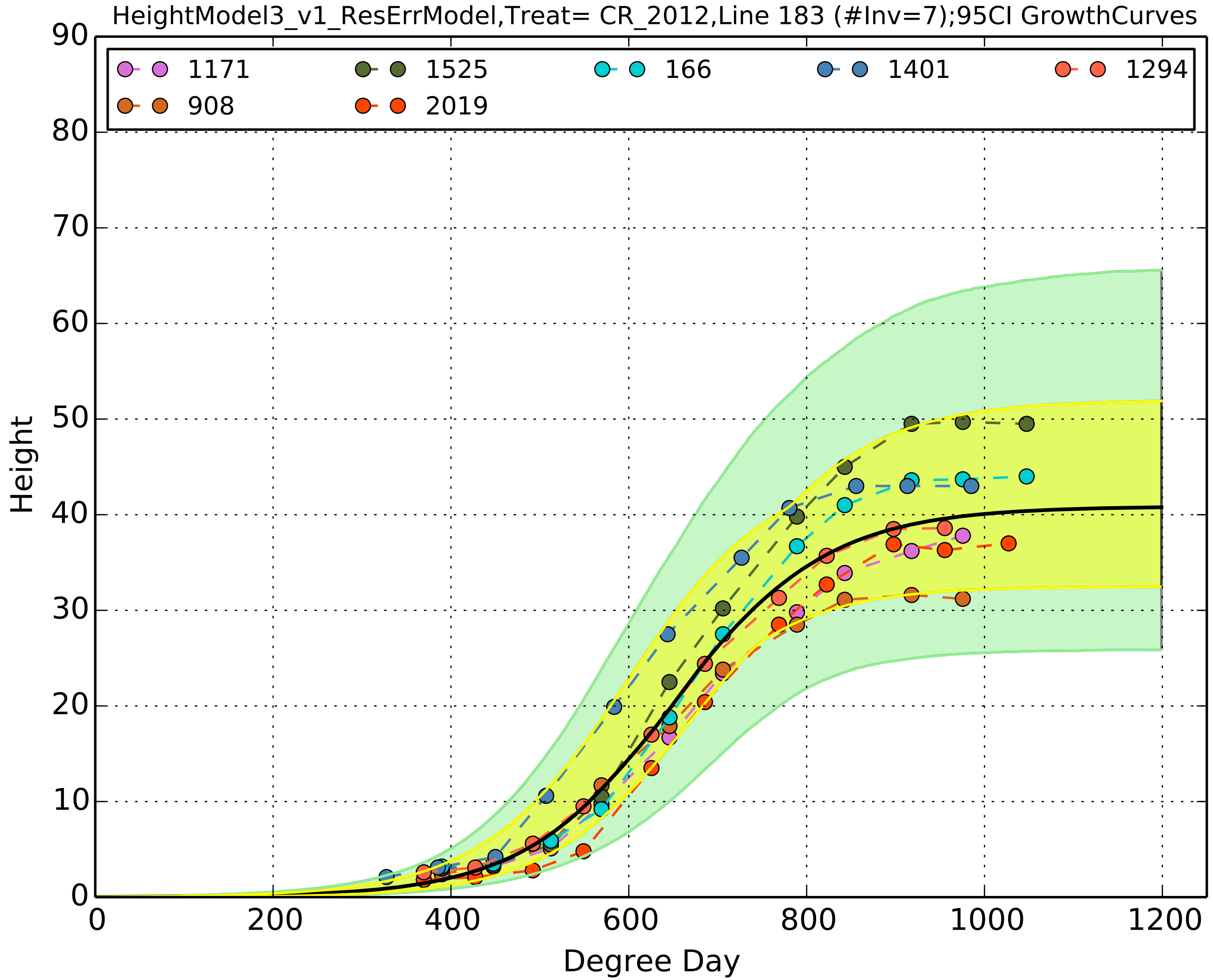

HeightModel3_v1_ResErrModel,Treat= CR_2012,Line 183 (#Inv=7);95CI GrowthCurves
1171
1525
166
1401
1294
908
2019
Height
Degree Day
0
10
20
30
40
50
60
70
80
90
0
200
400
600
800
1000
1200

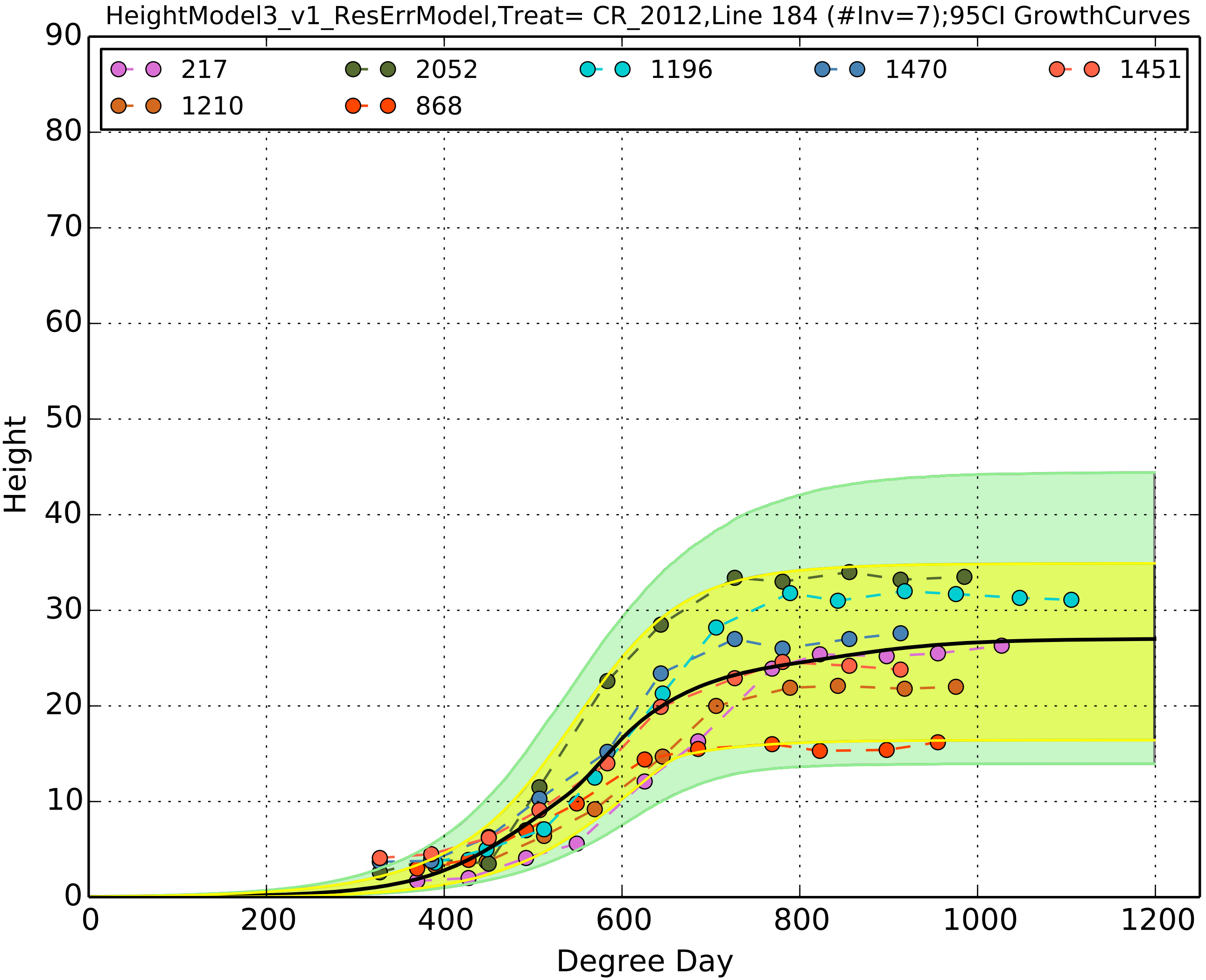

HeightModel3_v1_ResErrModel,Treat= CR_2012,Line 184 (#Inv=7);95CI GrowthCurves
217
2052
1196
1470
1451
1210
868
Height
Degree Day
0
10
20
30
40
50
60
70
80
90
0
200
400
600
800
1000
1200

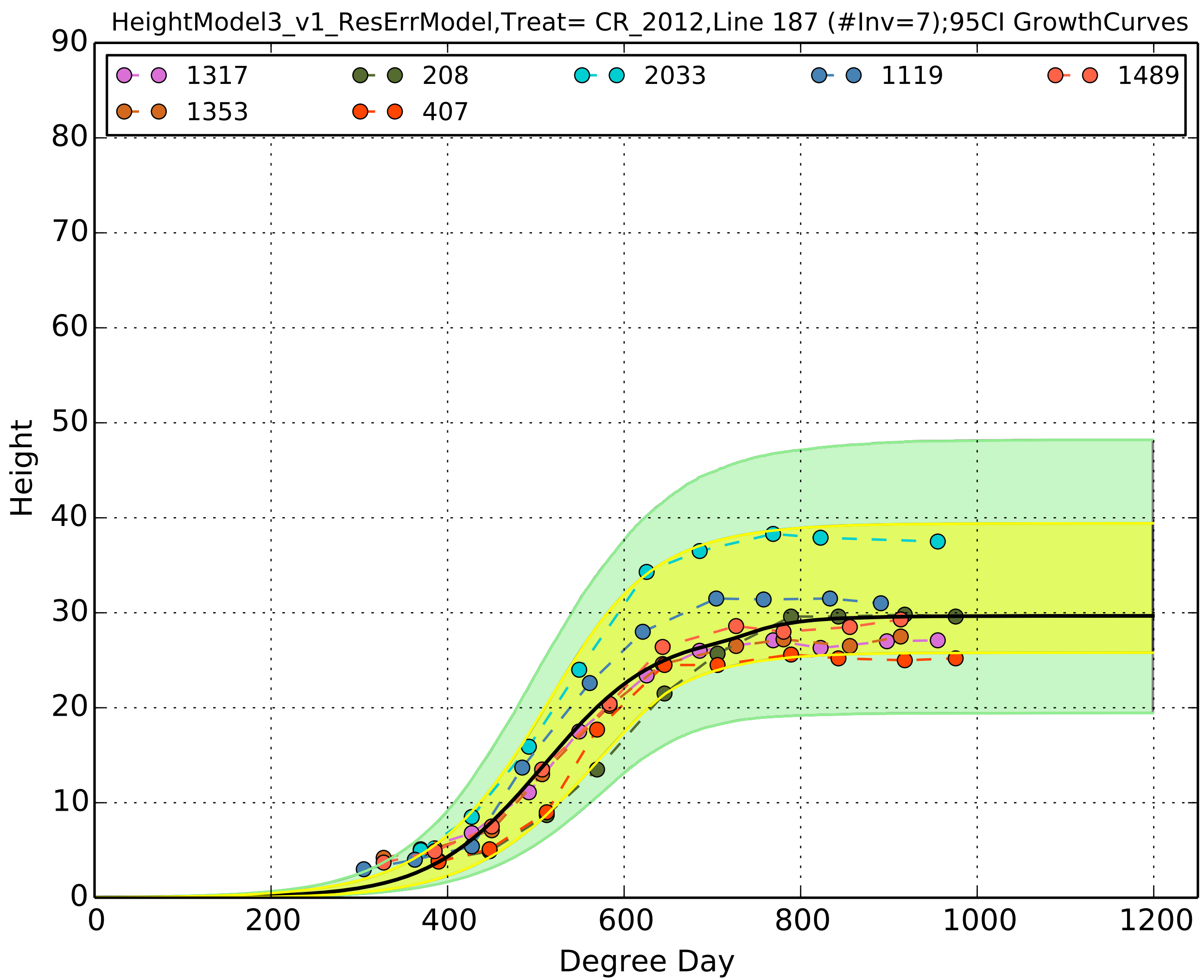
HeightModel3_v1_ResErrModel,Treat= CR_2012,Line 187 (#Inv=7);95CI GrowthCurves
1317
208
2033
1119
1489
1353
407
Height
Degree Day
0
10
20
30
40
50
60
70
80
90
0
200
400
600
800
1000
1200

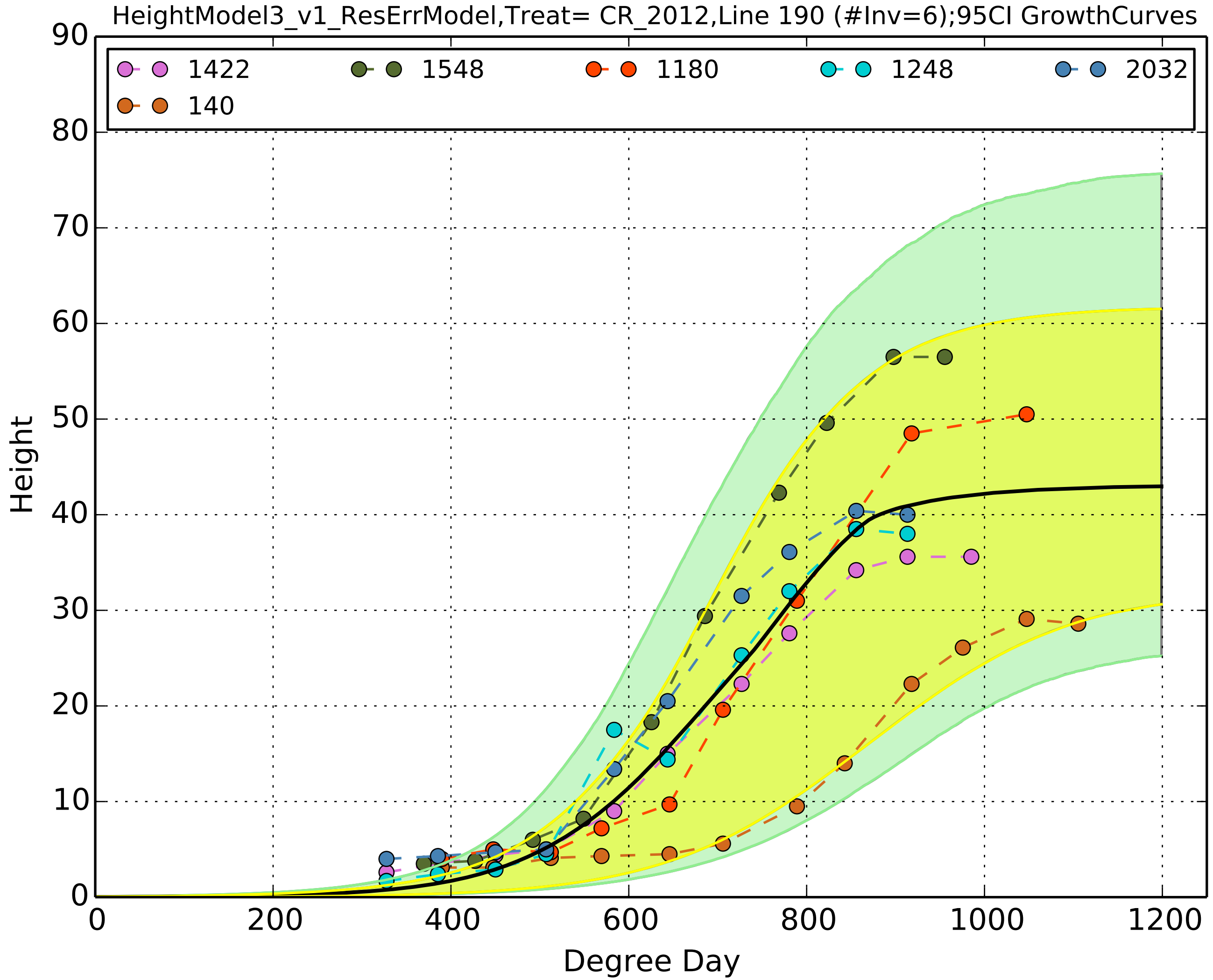
HeightModel3_v1_ResErrModel,Treat= CR_2012,Line 190 (#Inv=6);95CI GrowthCurves
1422
1548
1180
1248
2032
140
Height
Degree Day
0
10
20
30
40
50
60
70
80
90
0
200
400
600
800
1000
1200

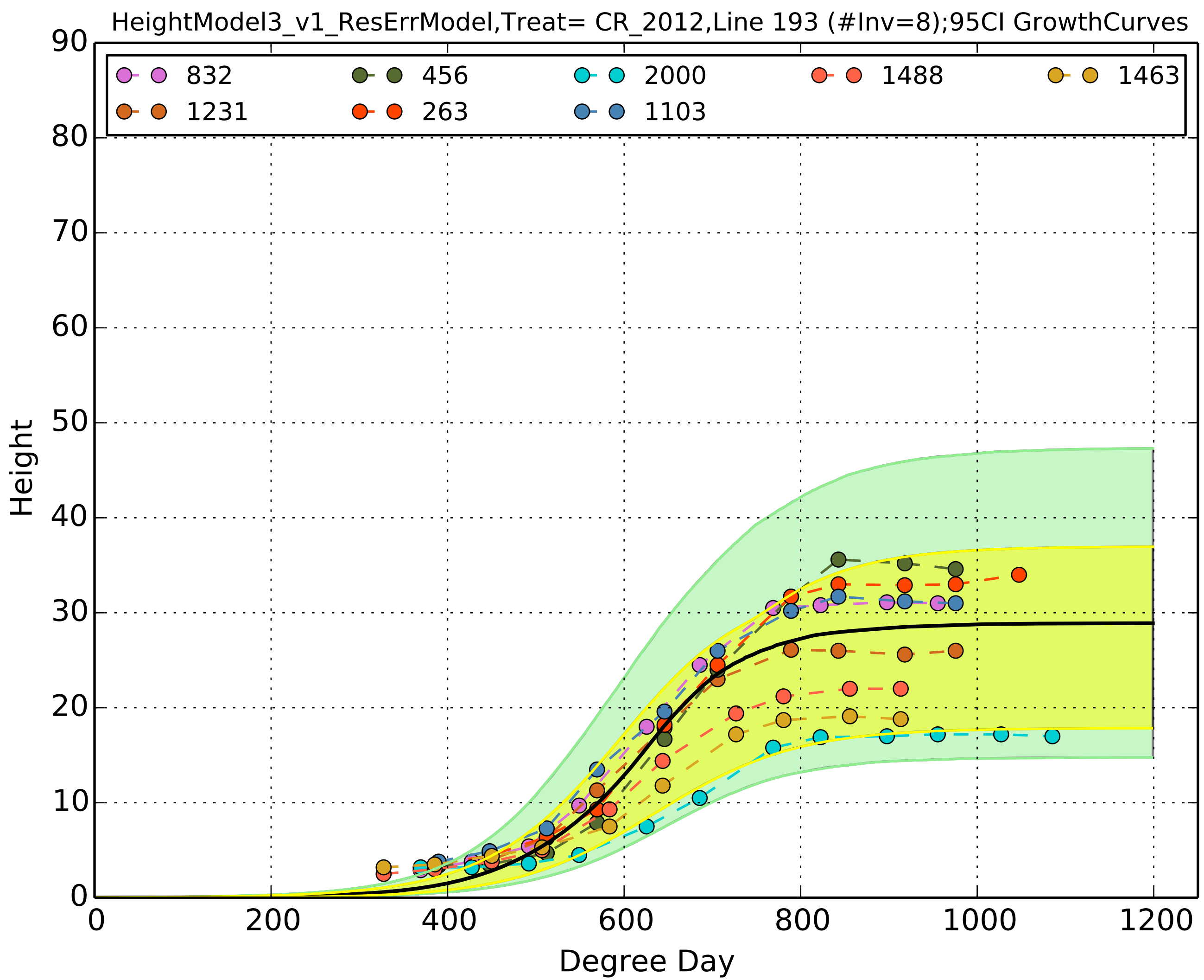

HeightModel3_v1_ResErrModel,Treat= CR_2012,Line 193 (#Inv=8);95CI GrowthCurves
832
456
2000
1488
1463
1231
263
1103
Height
Degree Day
0
10
20
30
40
50
60
70
80
90
0
200
400
600
800
1000
1200

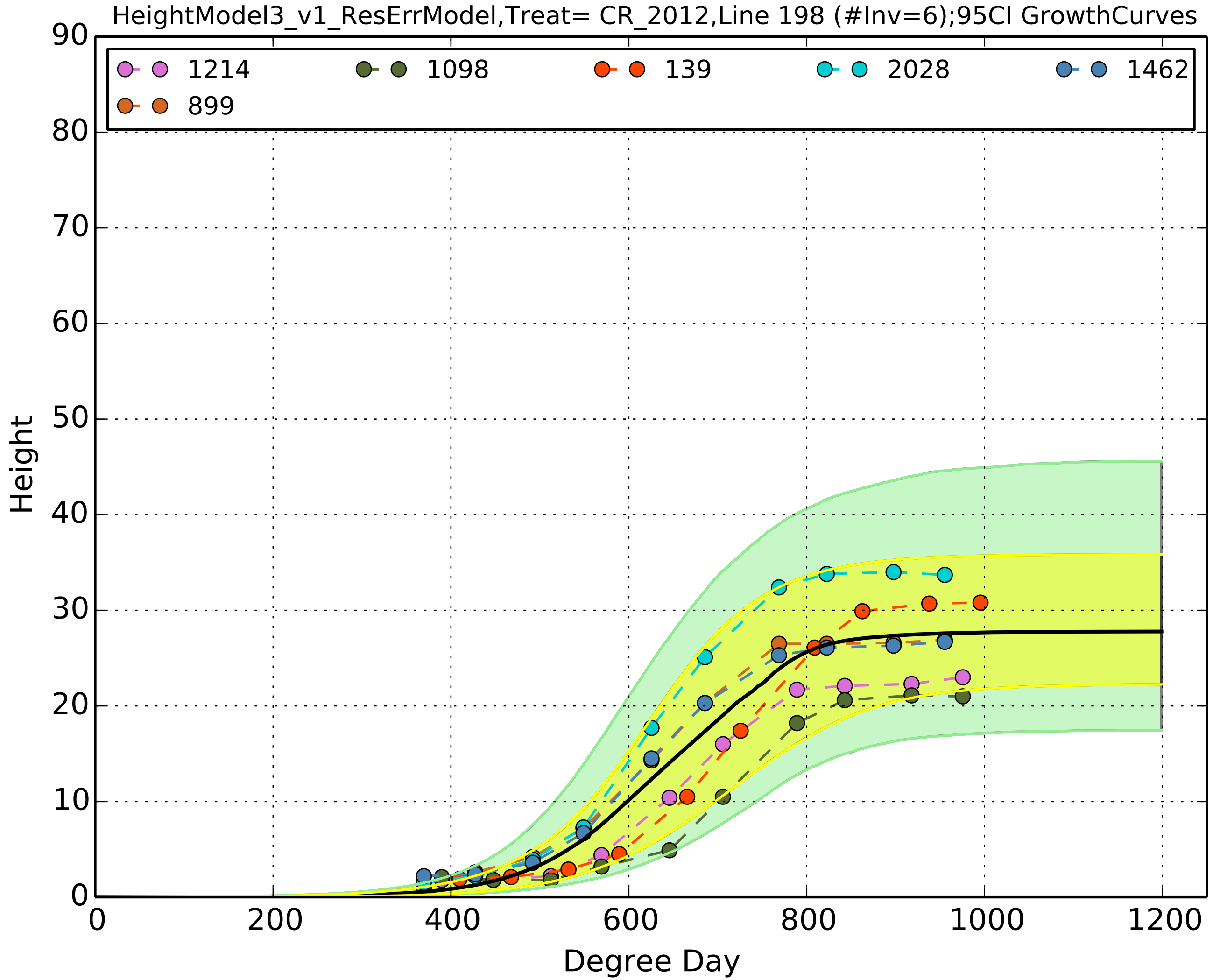

HeightModel3_v1_ResErrModel,Treat= CR_2012,Line 198 (#Inv=6);95CI GrowthCurves
1214
1098
139
2028
1462
899
Height
Degree Day
0
10
20
30
40
50
60
70
80
90
0
200
400
600
800
1000
1200

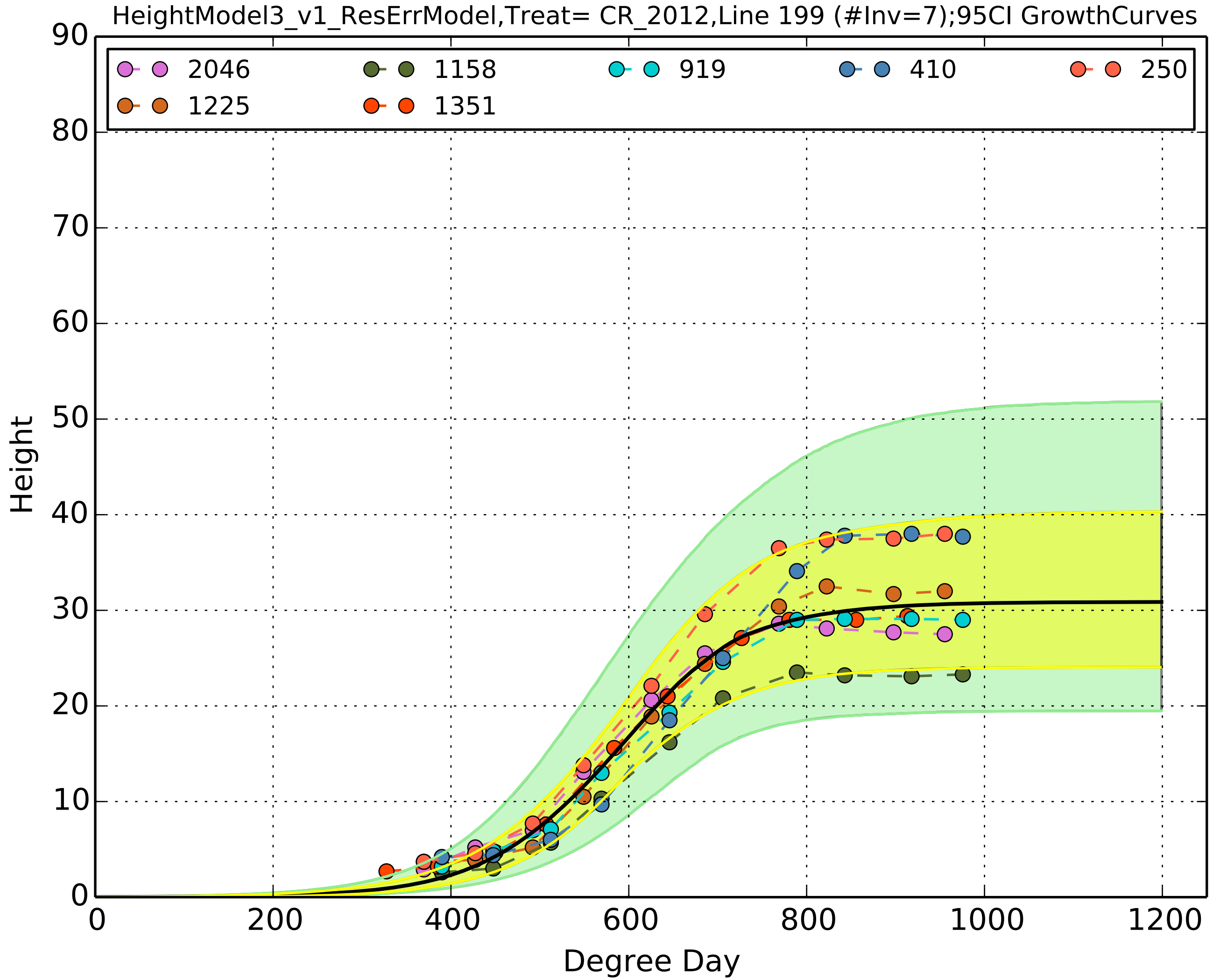

HeightModel3_v1_ResErrModel,Treat= CR_2012,Line 199 (#Inv=7);95CI GrowthCurves
2046
1158
919
410
250
1225
1351
Height
Degree Day
0
10
20
30
40
50
60
70
80
90
0
200
400
600
800
1000
1200

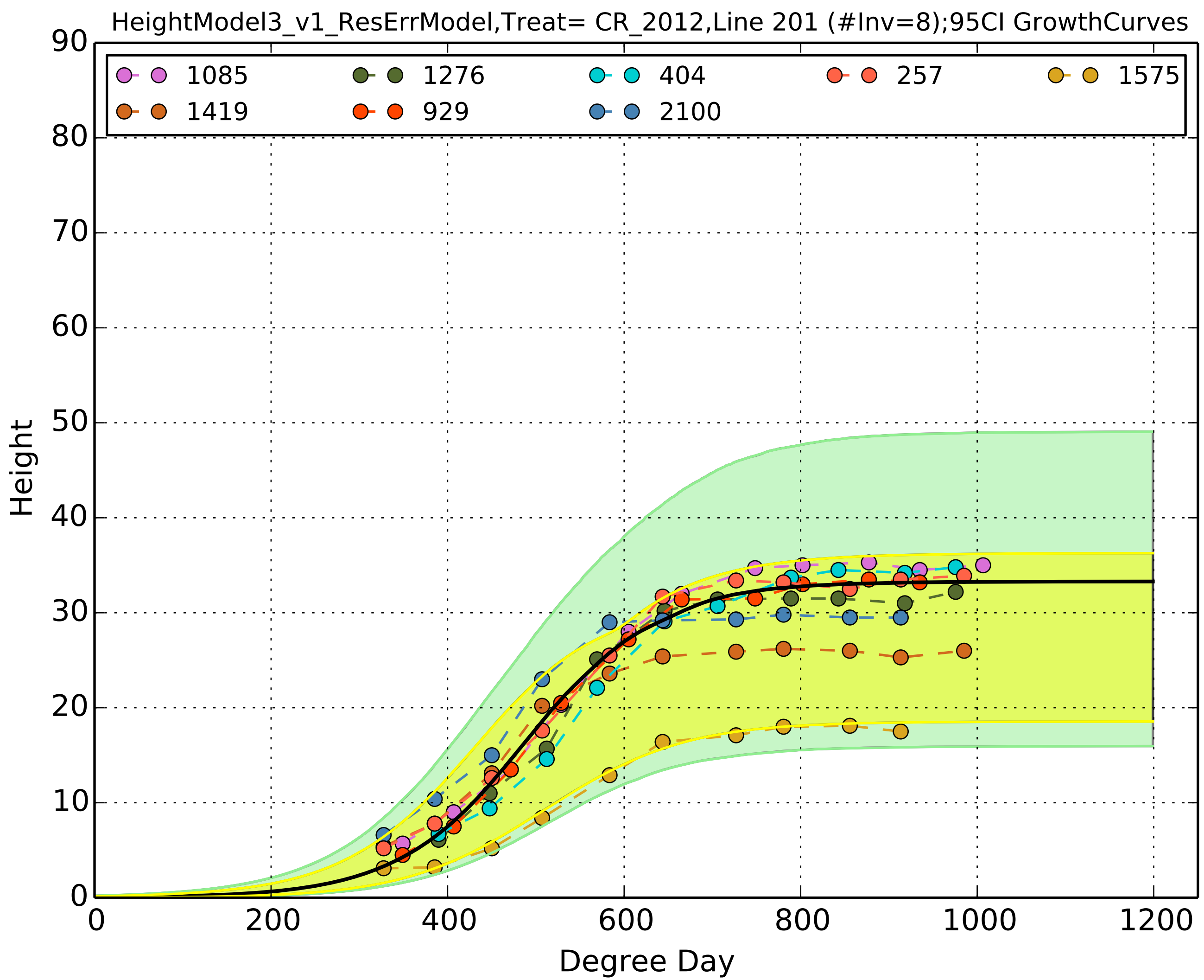

HeightModel3_v1_ResErrModel,Treat= CR_2012,Line 201 (#Inv=8);95CI GrowthCurves
1085
1276
404
257
1575
1419
929
2100
Height
Degree Day
0
10
20
30
40
50
60
70
80
90
0
200
400
600
800
1000
1200

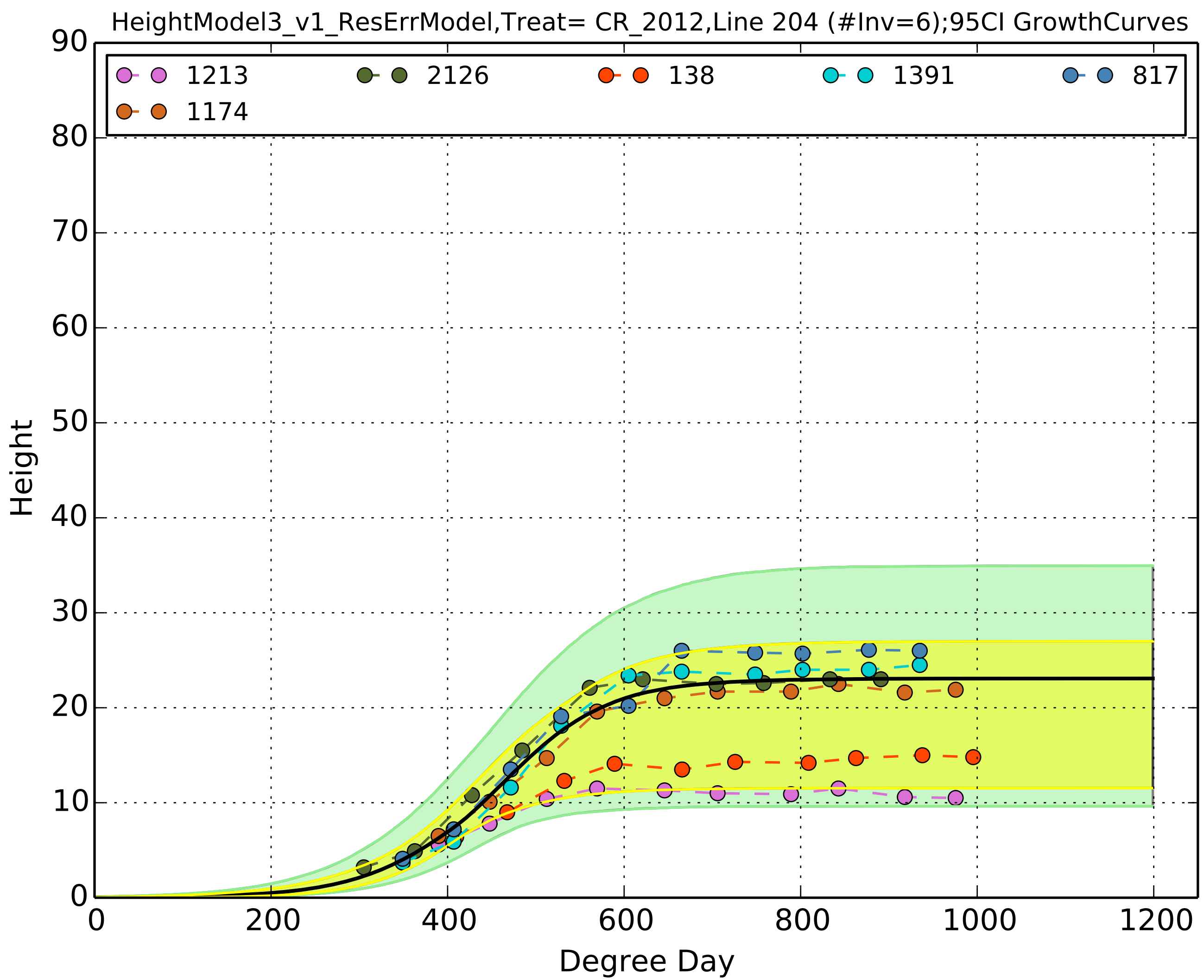

HeightModel3_v1_ResErrModel,Treat= CR_2012,Line 204 (#Inv=6);95CI GrowthCurves
1213
2126
138
1391
817
1174
Height
Degree Day
0
10
20
30
40
50
60
70
80
90
0
200
400
600
800
1000
1200

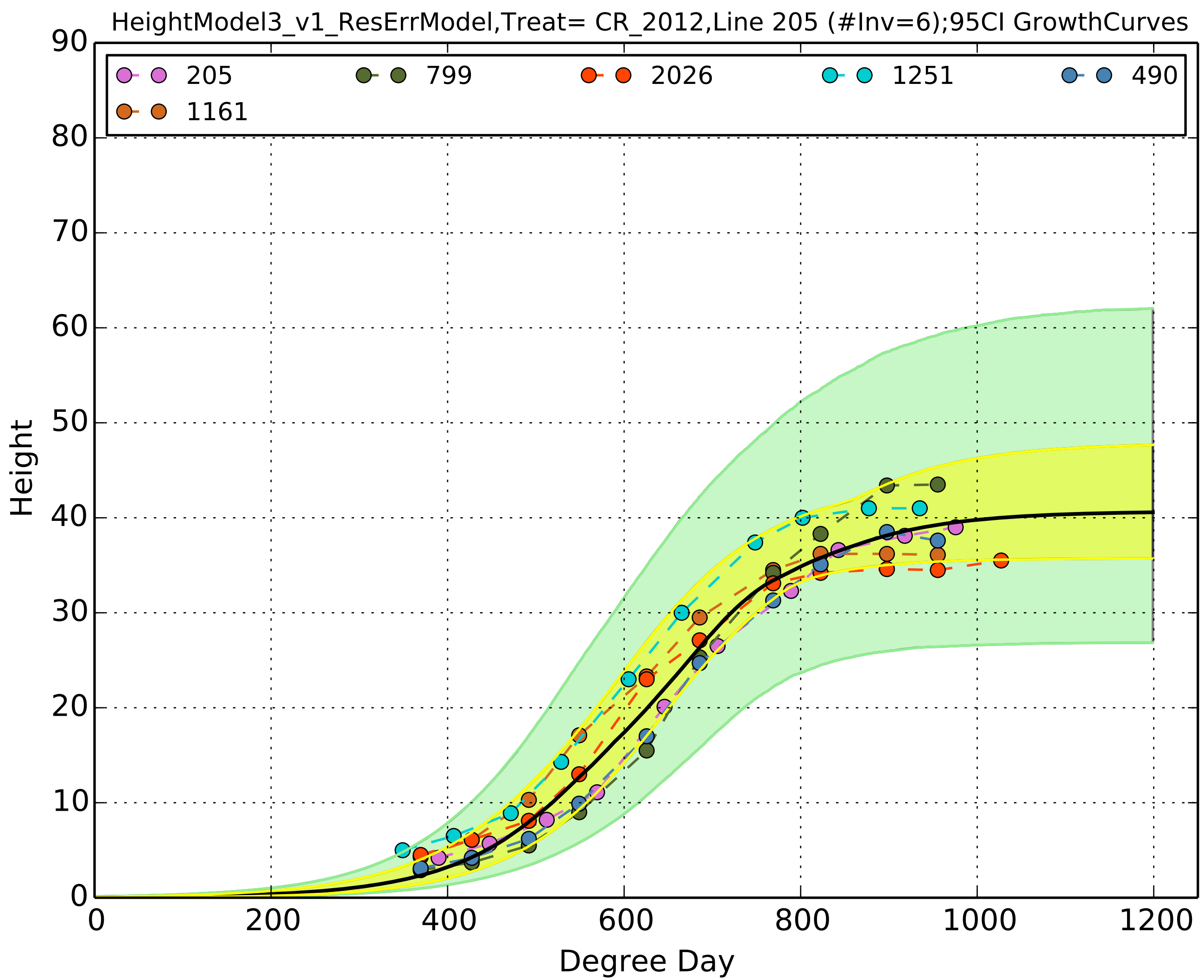
HeightModel3_v1_ResErrModel,Treat= CR_2012,Line 205 (#Inv=6);95CI GrowthCurves
205
799
2026
1251
490
1161
Height
Degree Day
0
10
20
30
40
50
60
70
80
90
0
200
400
600
800
1000
1200

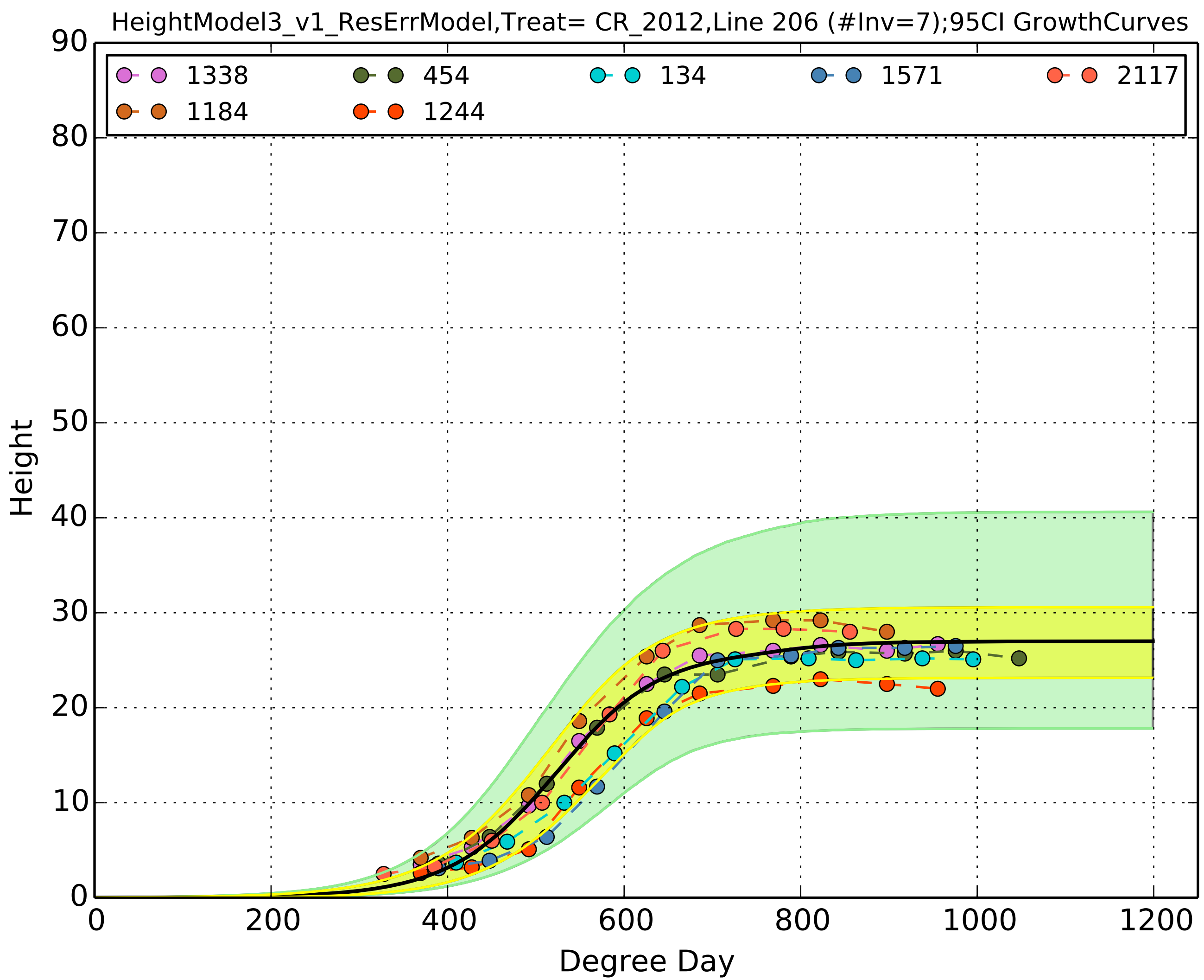

HeightModel3_v1_ResErrModel,Treat= CR_2012,Line 206 (#Inv=7);95CI GrowthCurves
1338
454
134
1571
2117
1184
1244
Height
Degree Day
0
10
20
30
40
50
60
70
80
90
0
200
400
600
800
1000
1200

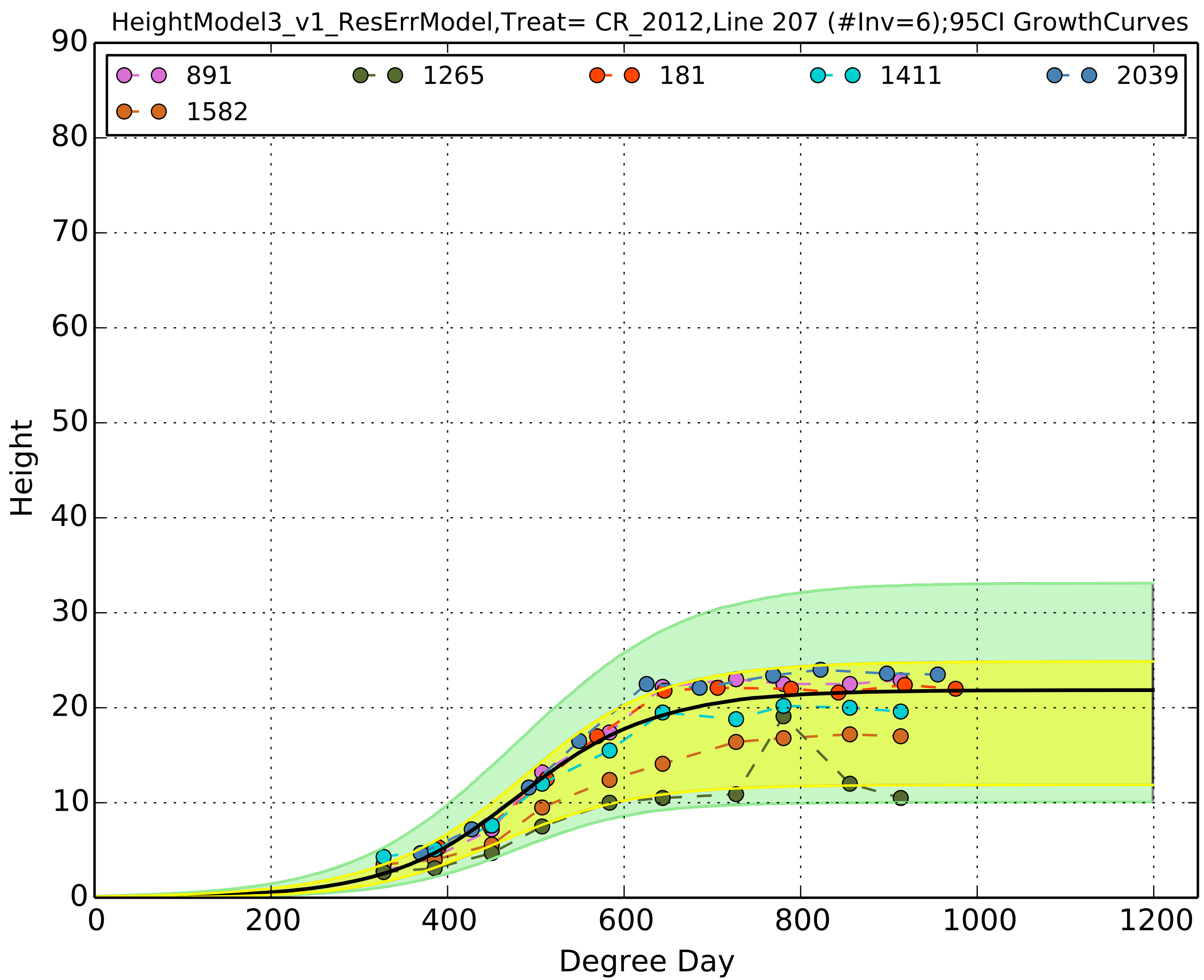

HeightModel3_v1_ResErrModel,Treat= CR_2012,Line 207 (#Inv=6);95CI GrowthCurves
891
1265
181
1411
2039
1582
Height
Degree Day
0
10
20
30
40
50
60
70
80
90
0
200
400
600
800
1000
1200

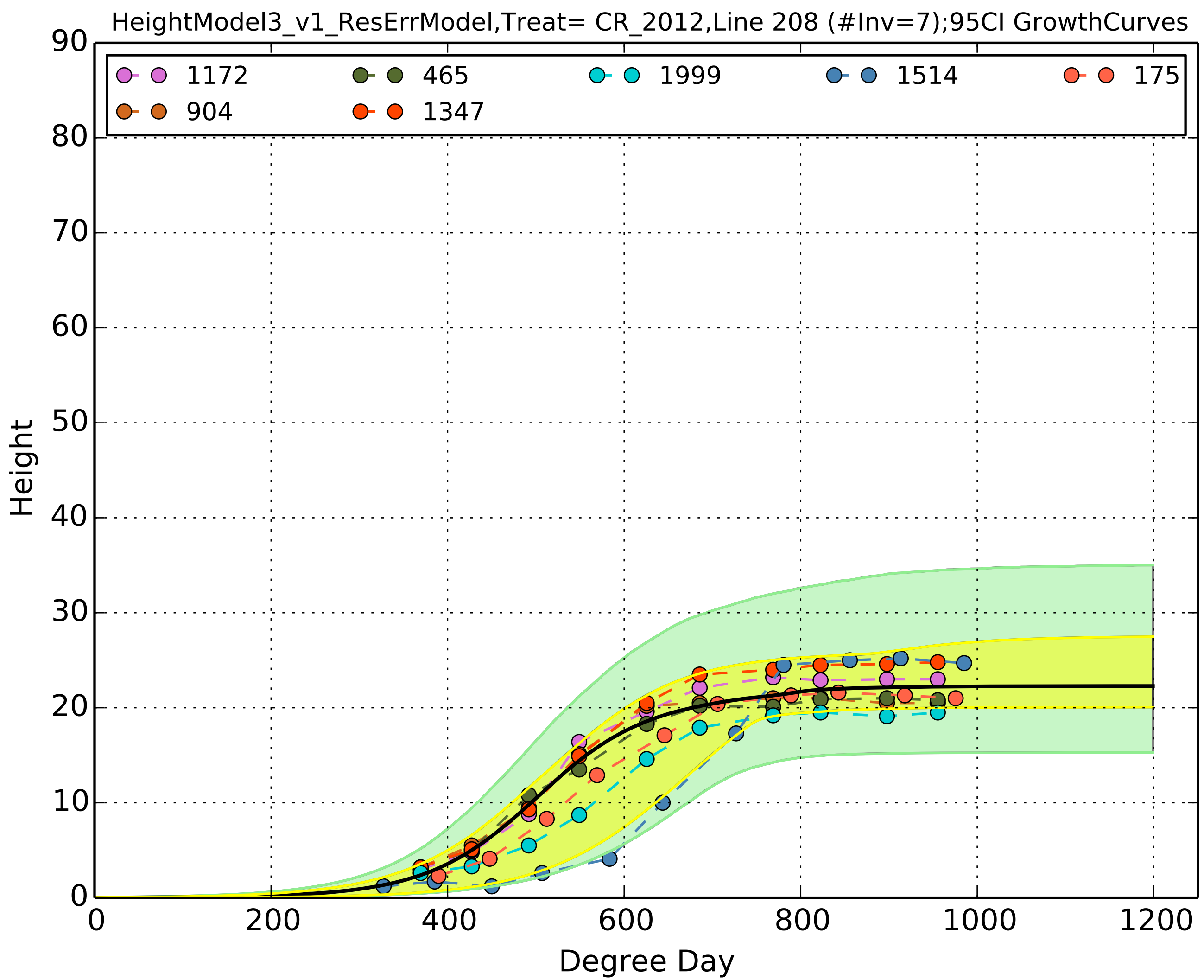
HeightModel3_v1_ResErrModel,Treat= CR_2012,Line 208 (#Inv=7);95CI GrowthCurves
1172
465
1999
1514
175
904
1347
Height
Degree Day
0
10
20
30
40
50
60
70
80
90
0
200
400
600
800
1000
1200

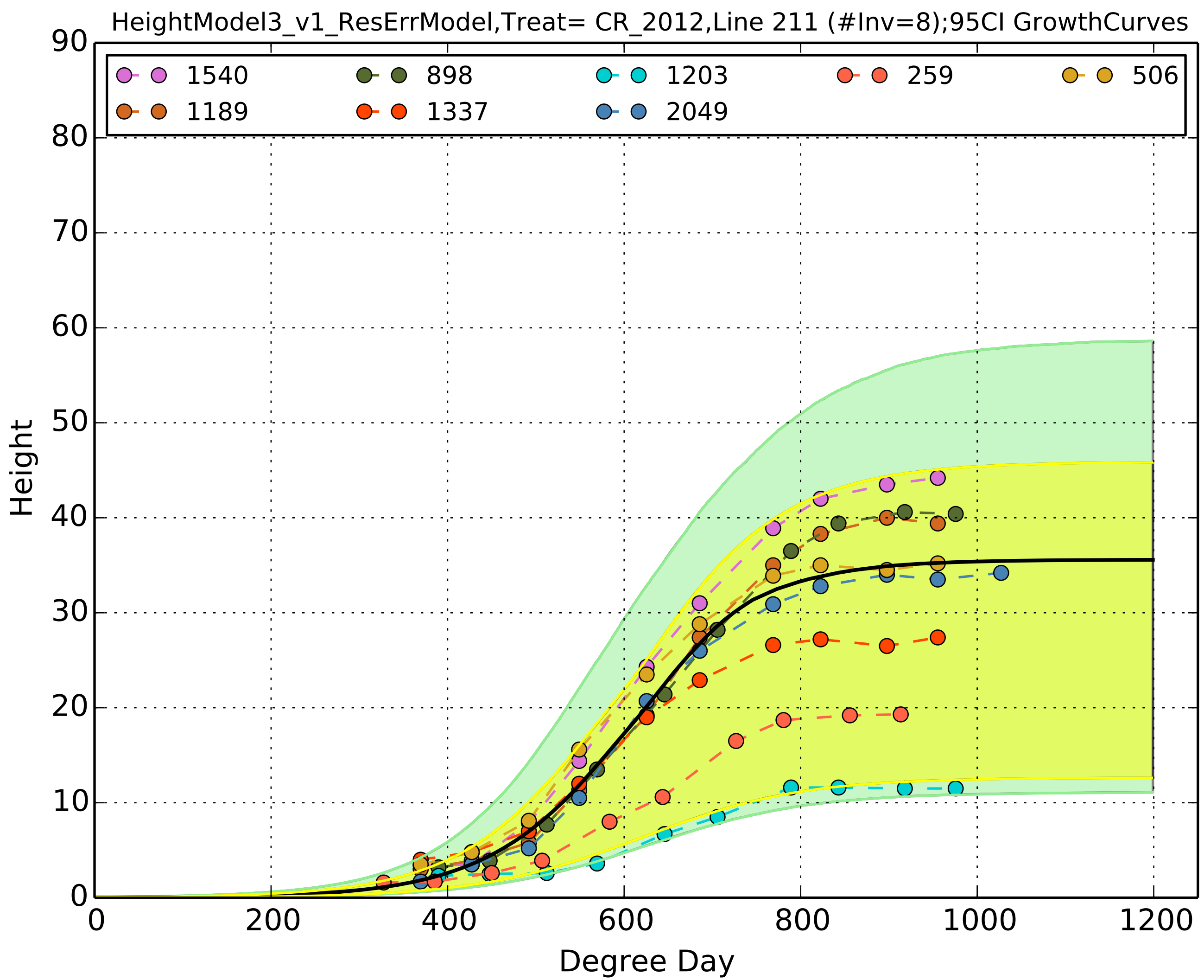
HeightModel3_v1_ResErrModel,Treat= CR_2012,Line 211 (#Inv=8);95CI GrowthCurves
1540
898
1203
259
506
1189
1337
2049
Height
Degree Day
0
10
20
30
40
50
60
70
80
90
0
200
400
600
800
1000
1200

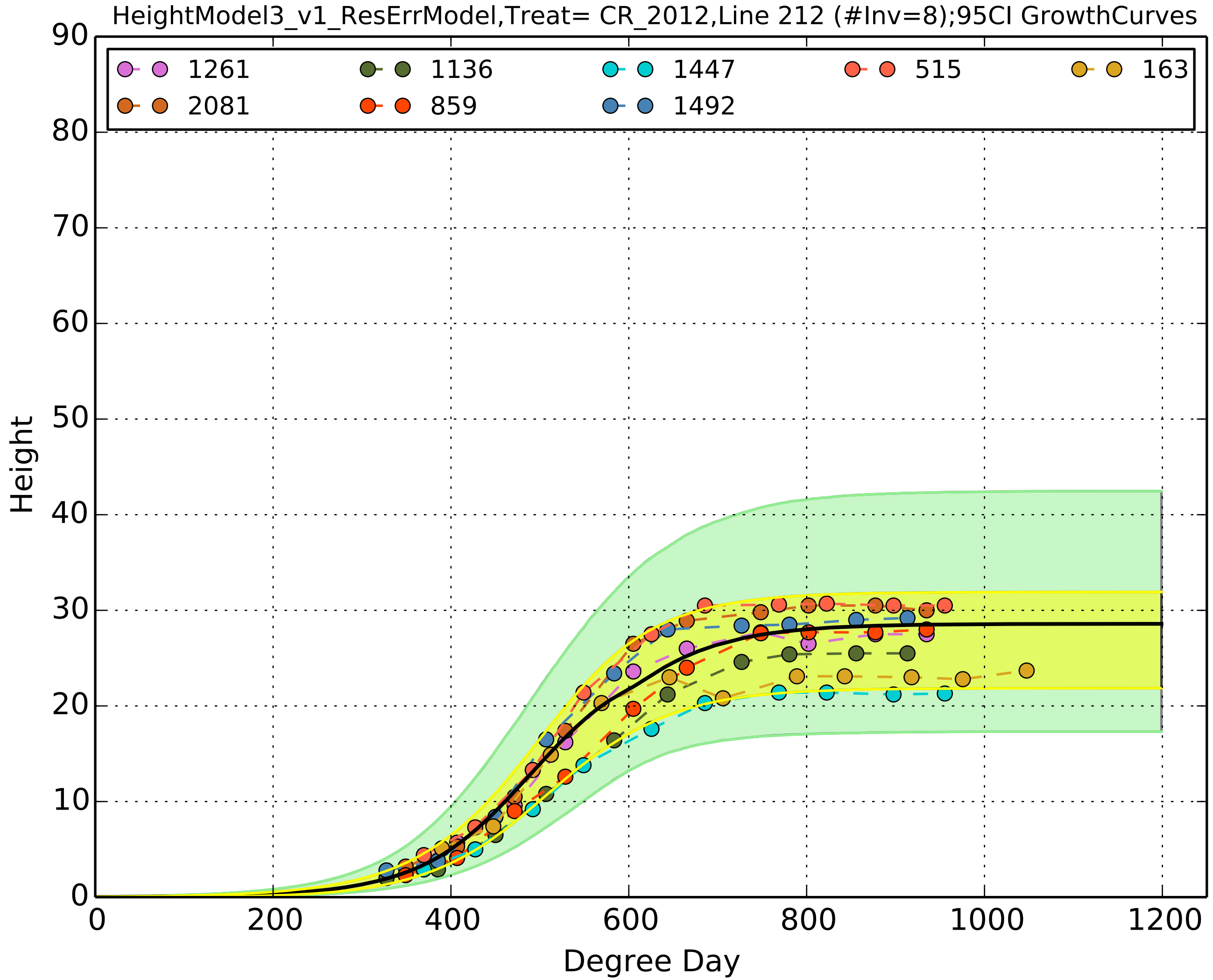

HeightModel3_v1_ResErrModel,Treat= CR_2012,Line 212 (#Inv=8);95CI GrowthCurves
1261
1136
1447
515
163
2081
859
1492
Height
Degree Day
0
10
20
30
40
50
60
70
80
90
0
200
400
600
800
1000
1200

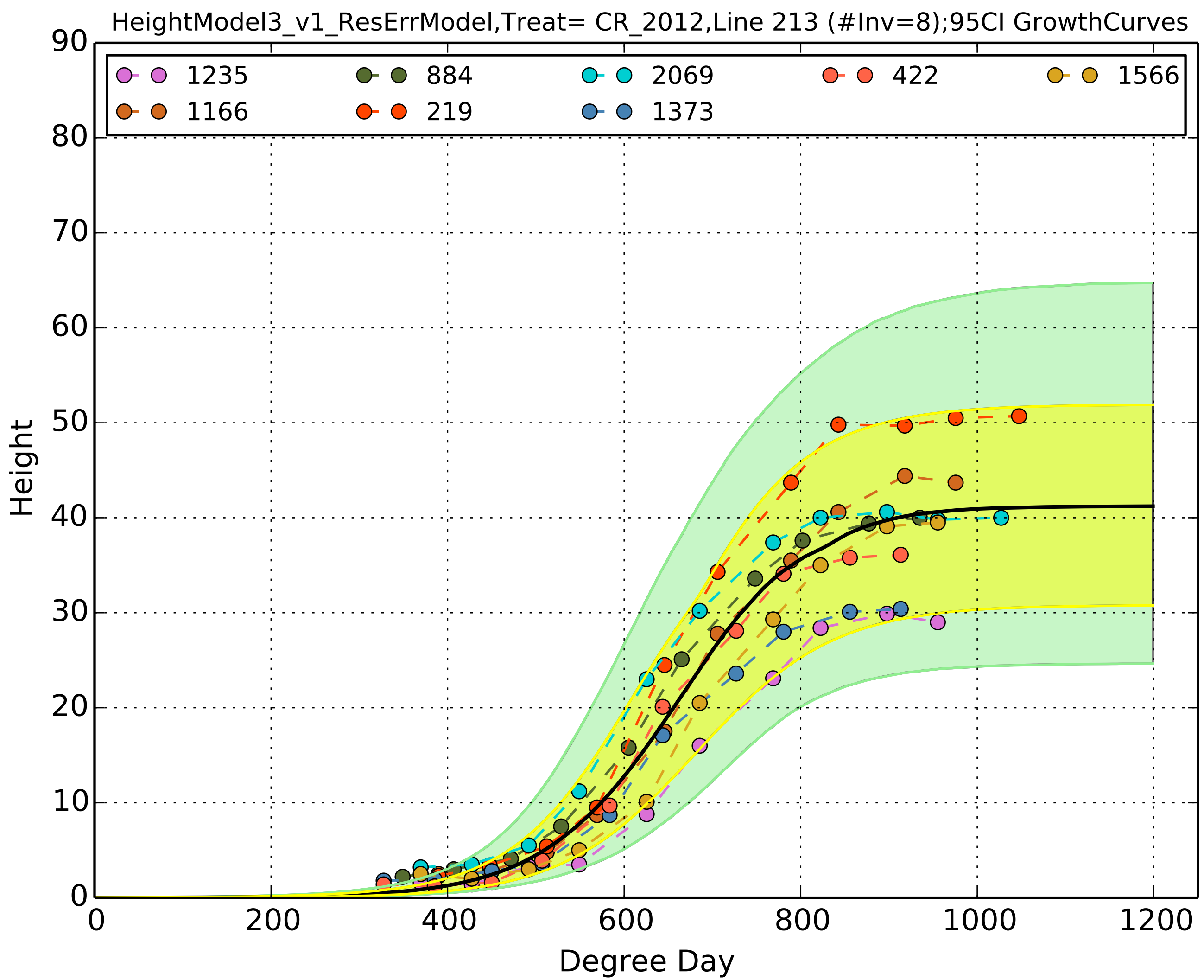

HeightModel3_v1_ResErrModel,Treat= CR_2012,Line 213 (#Inv=8);95CI GrowthCurves
1235
884
2069
422
1566
1166
219
1373
Height
Degree Day
0
10
20
30
40
50
60
70
80
90
0
200
400
600
800
1000
1200

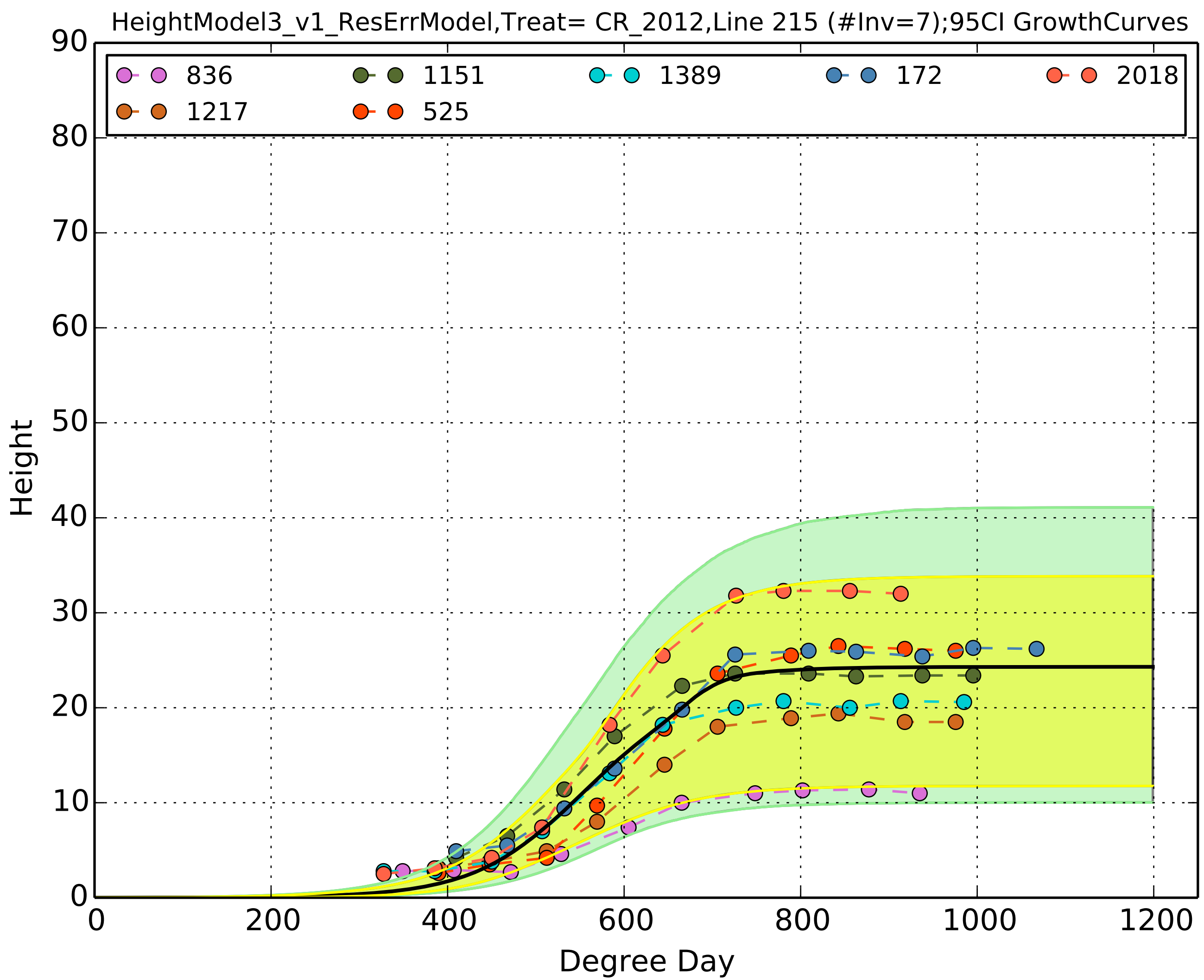
HeightModel3_v1_ResErrModel,Treat= CR_2012,Line 215 (#Inv=7);95CI GrowthCurves
836
1151
1389
172
2018
1217
525
Height
Degree Day
0
10
20
30
40
50
60
70
80
90
0
200
400
600
800
1000
1200

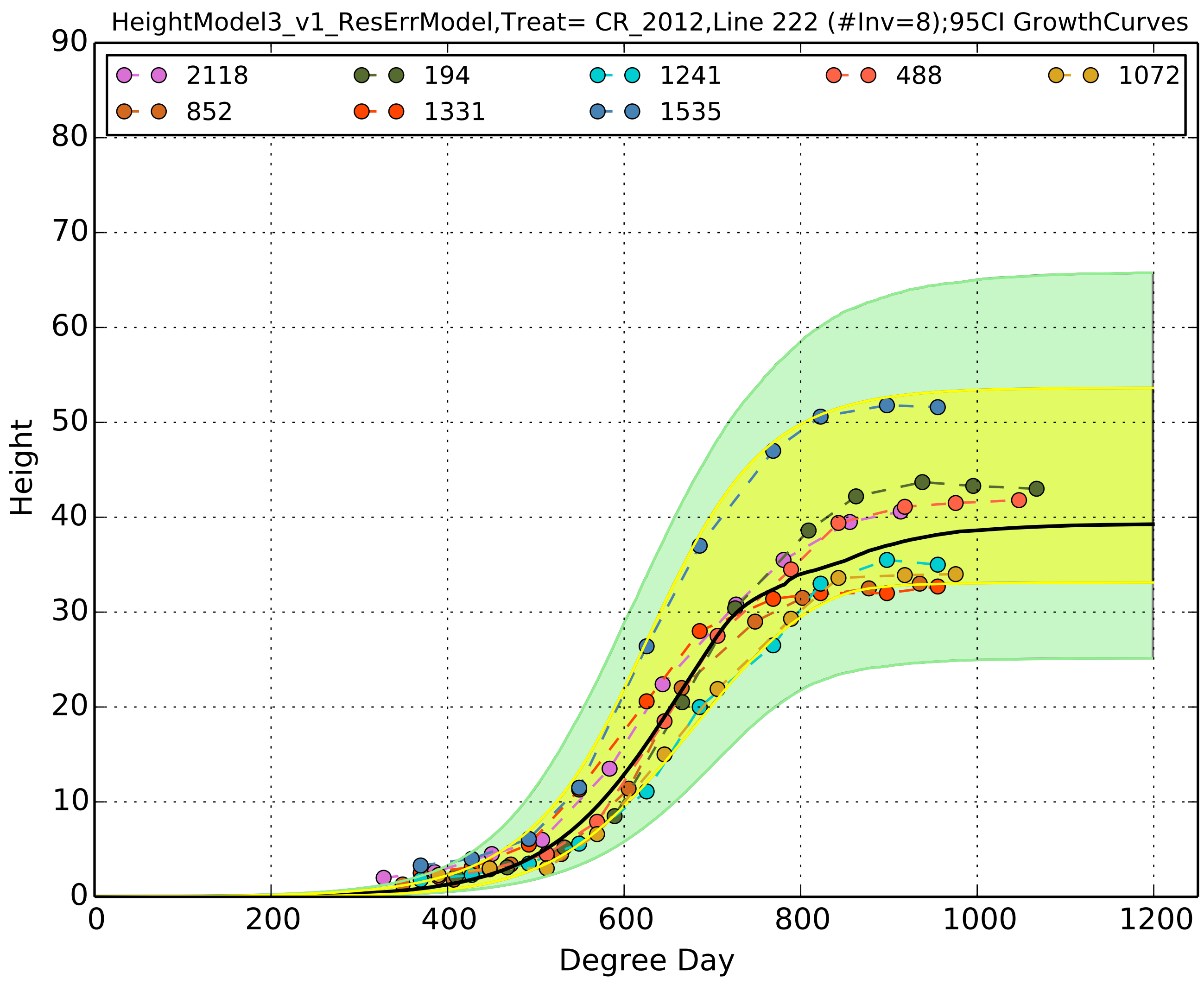

HeightModel3_v1_ResErrModel,Treat= CR_2012,Line 222 (#Inv=8);95CI GrowthCurves
2118
194
1241
488
1072
852
1331
1535
Height
Degree Day
0
10
20
30
40
50
60
70
80
90
0
200
400
600
800
1000
1200

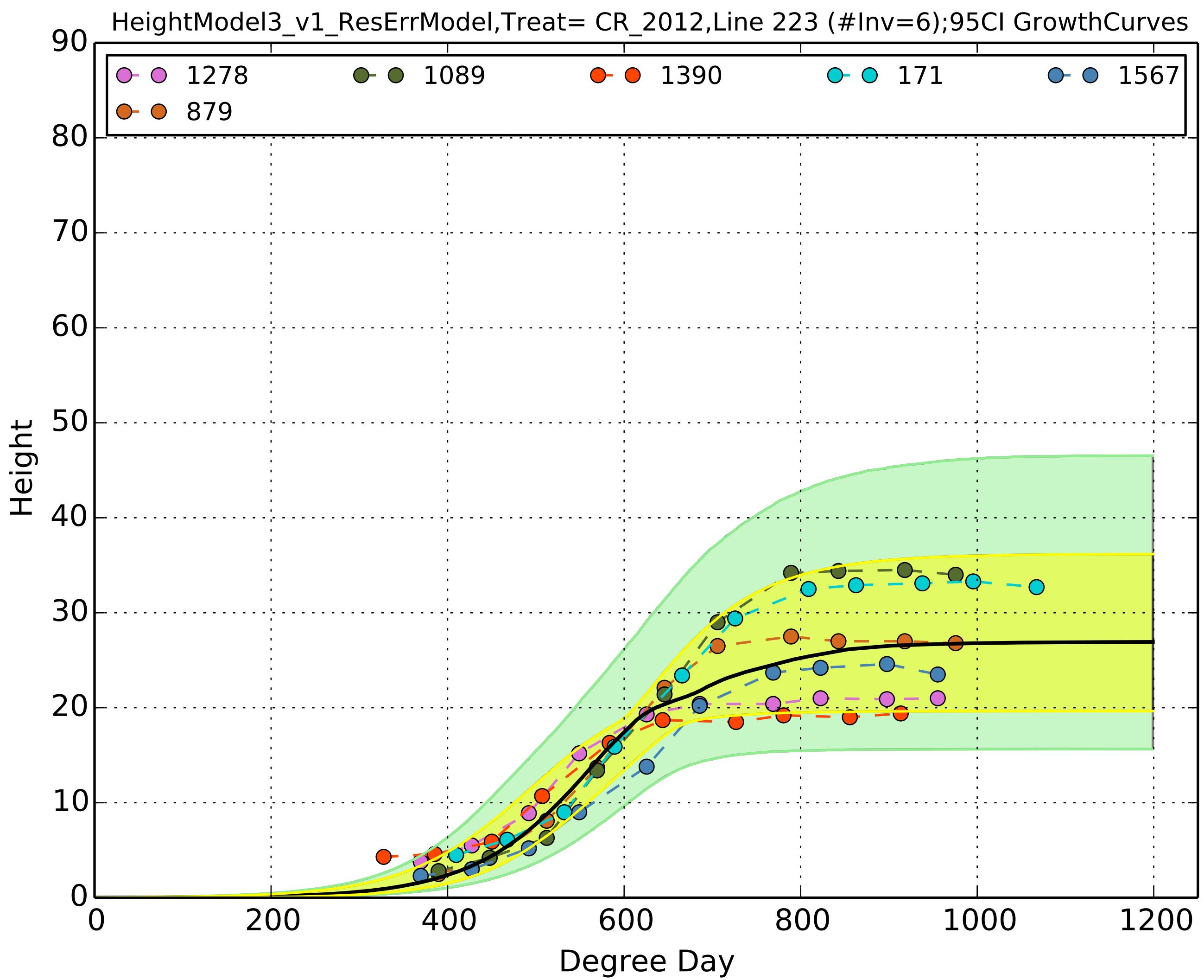
HeightModel3_v1_ResErrModel,Treat= CR_2012,Line 223 (#Inv=6);95CI GrowthCurves
1278
1089
1390
171
1567
879
Height
Degree Day
0
10
20
30
40
50
60
70
80
90
0
200
400
600
800
1000
1200

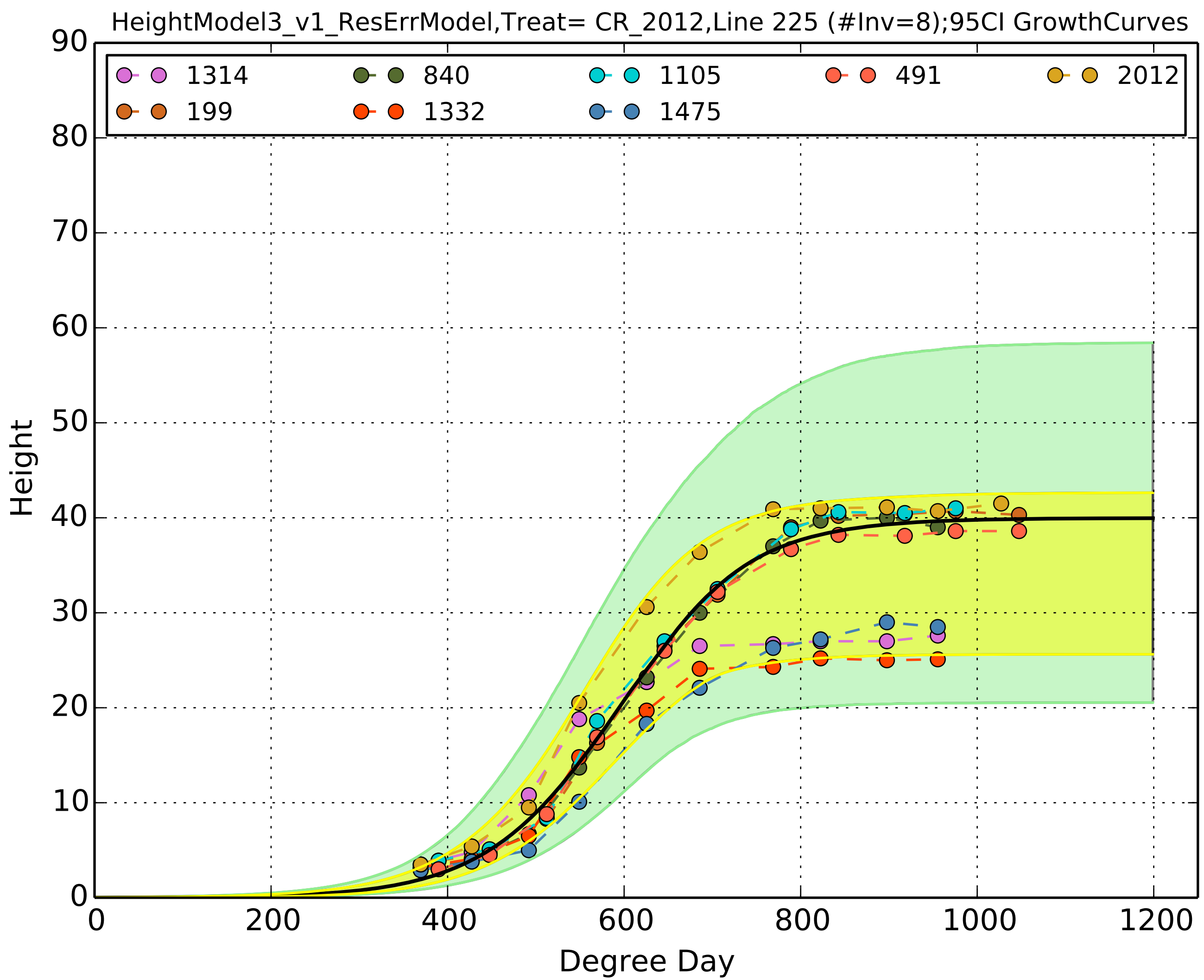
HeightModel3_v1_ResErrModel,Treat= CR_2012,Line 225 (#Inv=8);95CI GrowthCurves
1314
840
1105
491
2012
199
1332
1475
Height
Degree Day
0
10
20
30
40
50
60
70
80
90
0
200
400
600
800
1000
1200

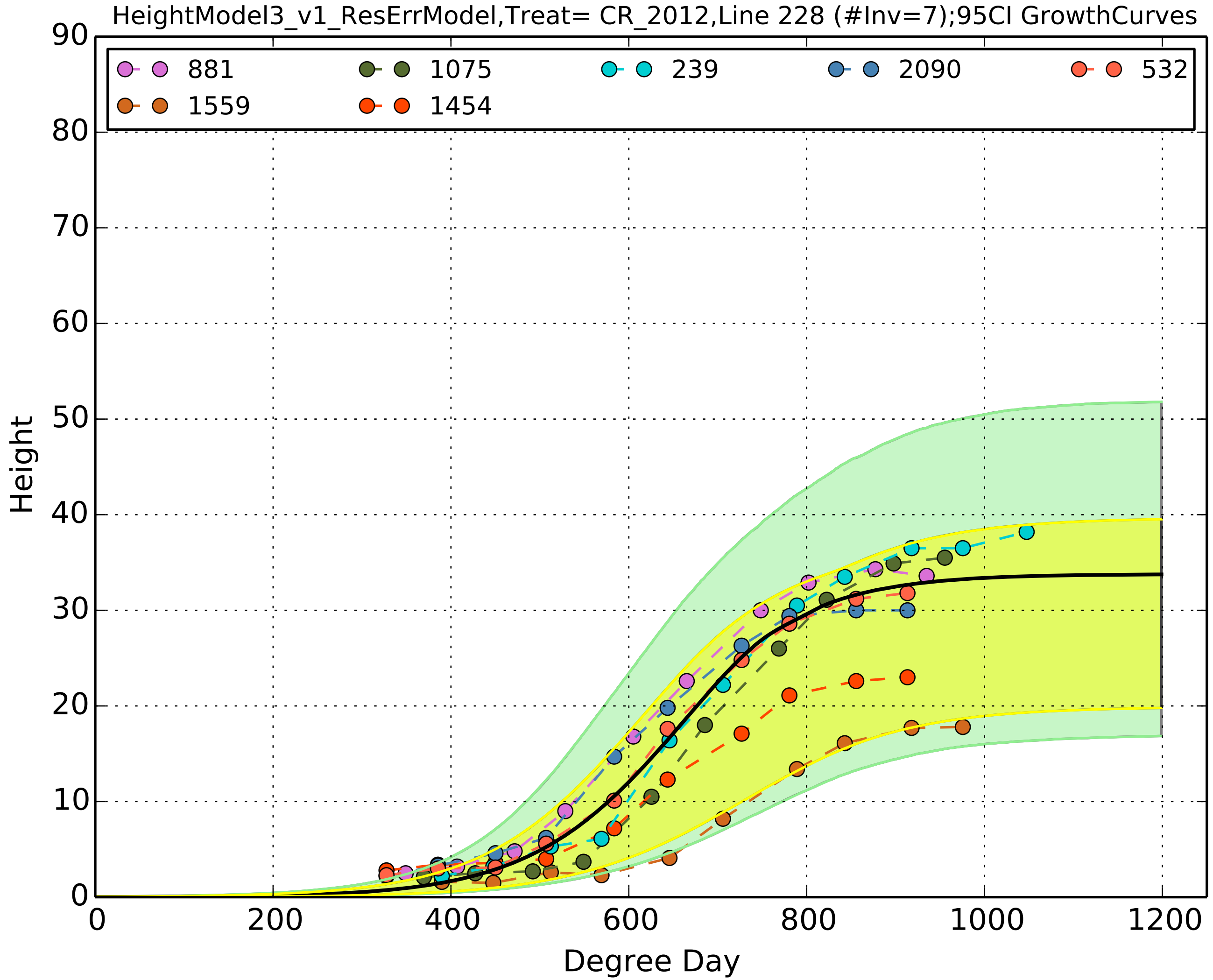

HeightModel3_v1_ResErrModel,Treat= CR_2012,Line 228 (#Inv=7);95CI GrowthCurves
881
1075
239
2090
532
1559
1454
Height
Degree Day
0
10
20
30
40
50
60
70
80
90
0
200
400
600
800
1000
1200

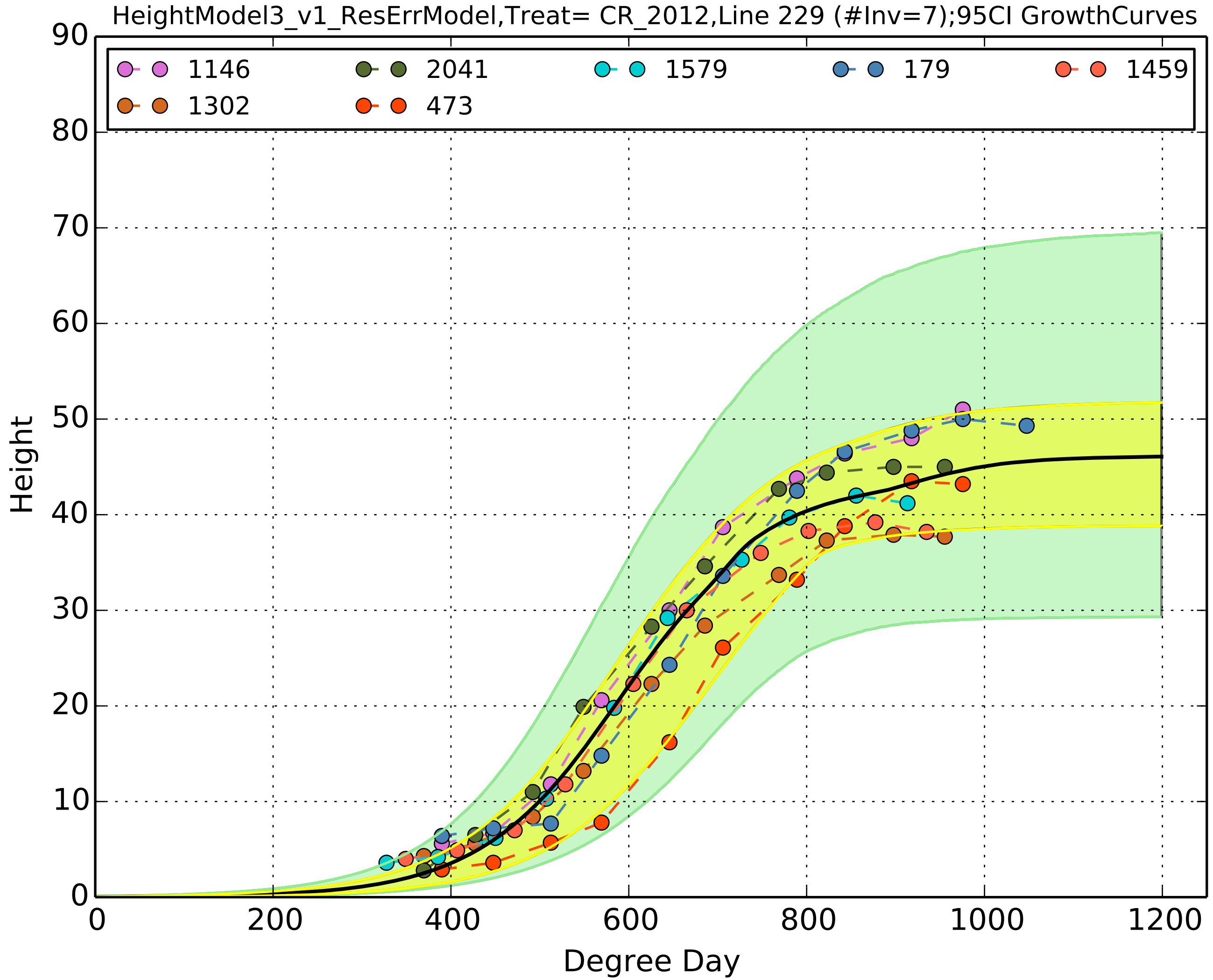
HeightModel3_v1_ResErrModel,Treat= CR_2012,Line 229 (#Inv=7);95CI GrowthCurves
1146
2041
1579
179
1459
1302
473
Height
Degree Day
0
10
20
30
40
50
60
70
80
90
0
200
400
600
800
1000
1200

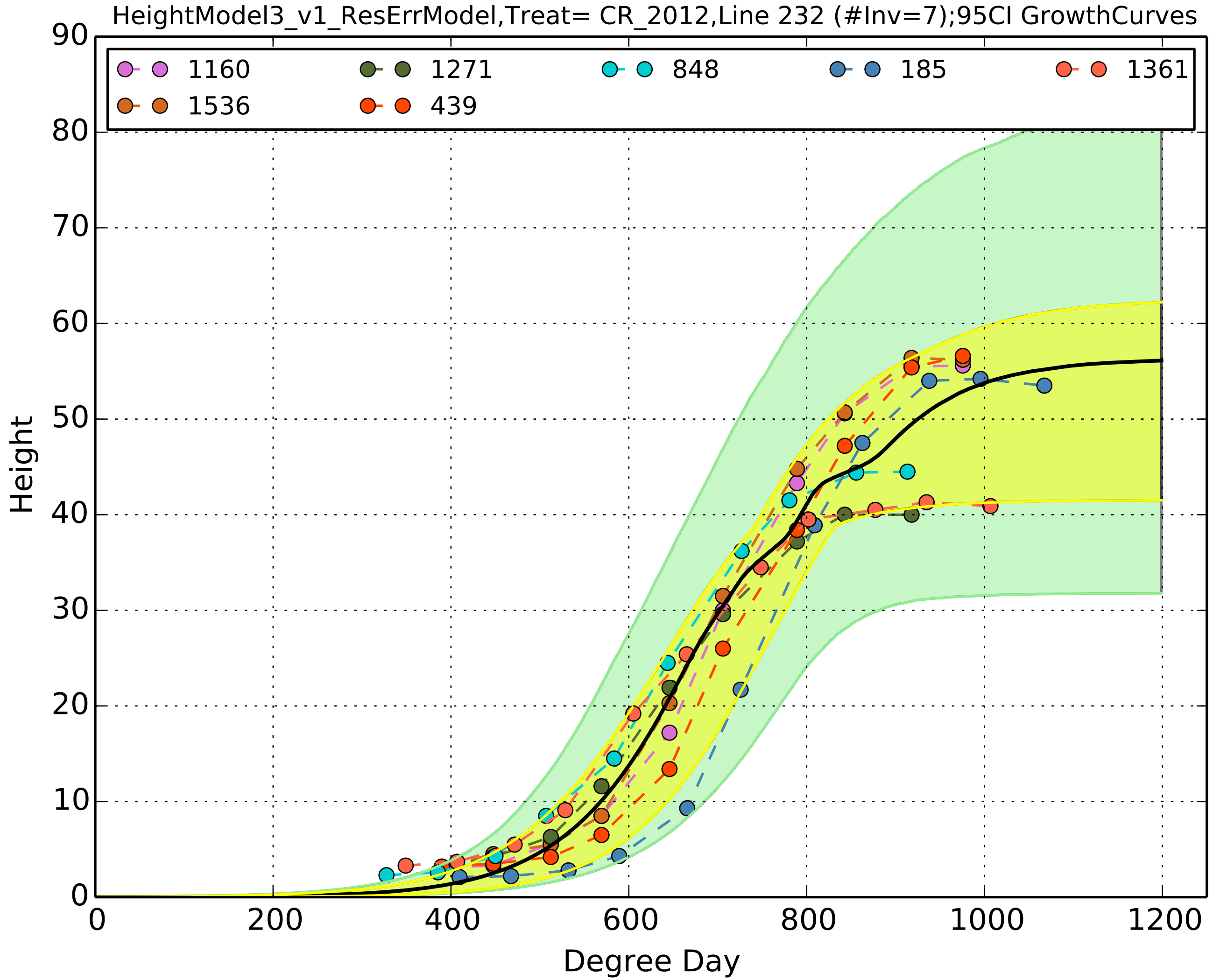

HeightModel3_v1_ResErrModel,Treat= CR_2012,Line 232 (#Inv=7);95CI GrowthCurves
1160
1271
848
185
1361
1536
439
Height
Degree Day
0
10
20
30
40
50
60
70
80
90
0
200
400
600
800
1000
1200

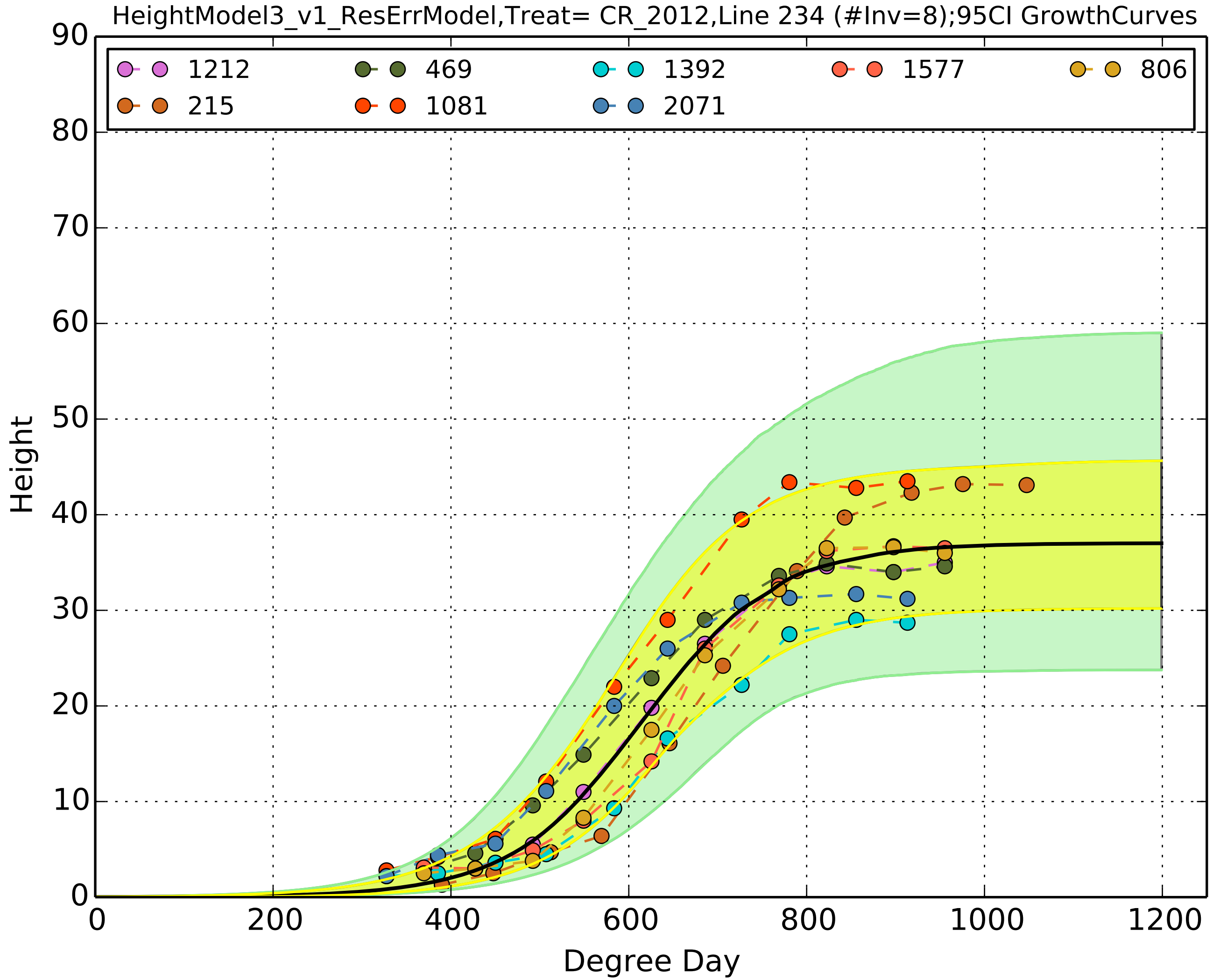

HeightModel3_v1_ResErrModel,Treat= CR_2012,Line 234 (#Inv=8);95CI GrowthCurves
1212
469
1392
1577
806
215
1081
2071
Height
Degree Day
0
10
20
30
40
50
60
70
80
90
0
200
400
600
800
1000
1200

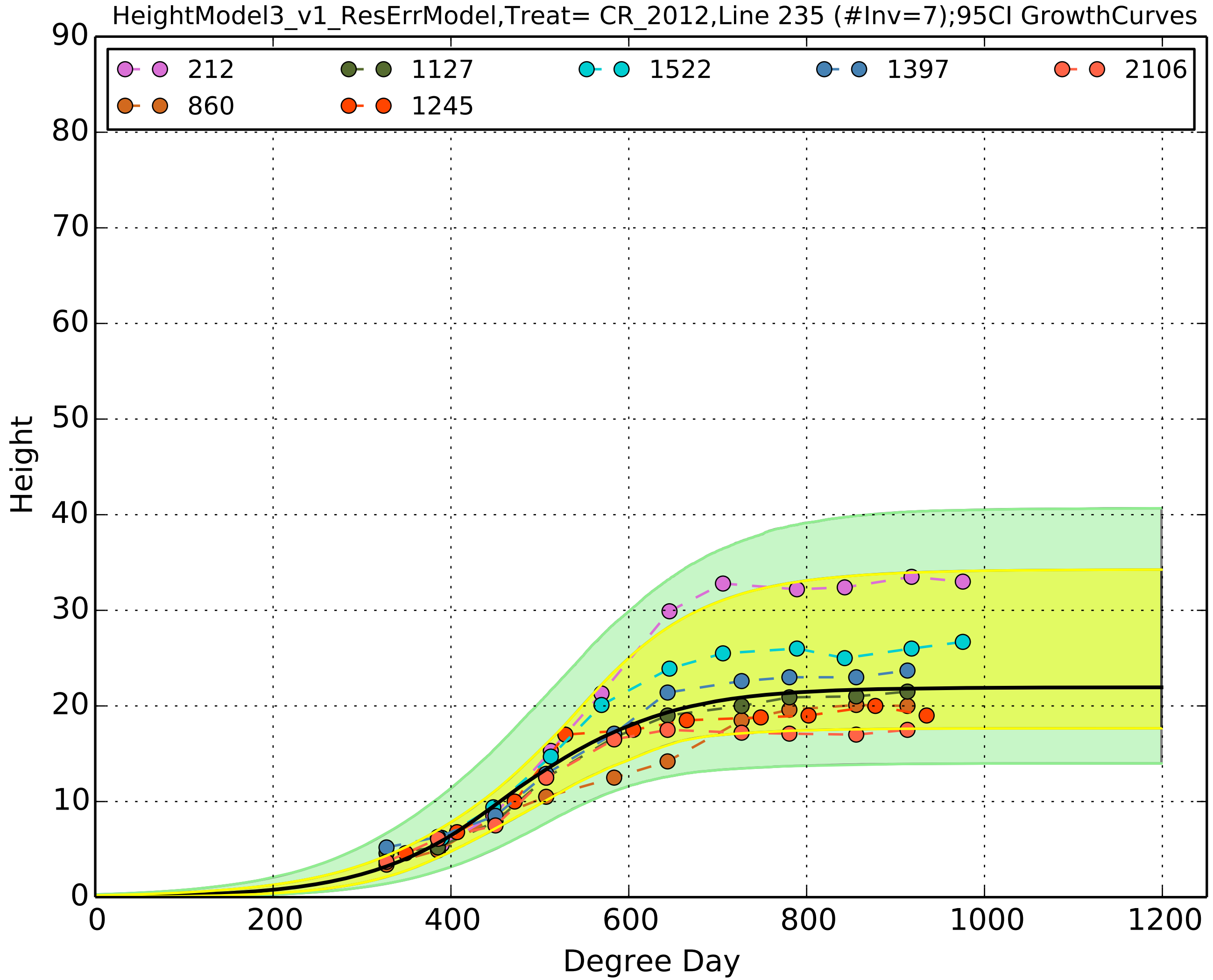

HeightModel3_v1_ResErrModel,Treat= CR_2012,Line 235 (#Inv=7);95CI GrowthCurves
212
1127
1522
1397
2106
860
1245
Height
Degree Day
0
10
20
30
40
50
60
70
80
90
0
200
400
600
800
1000
1200

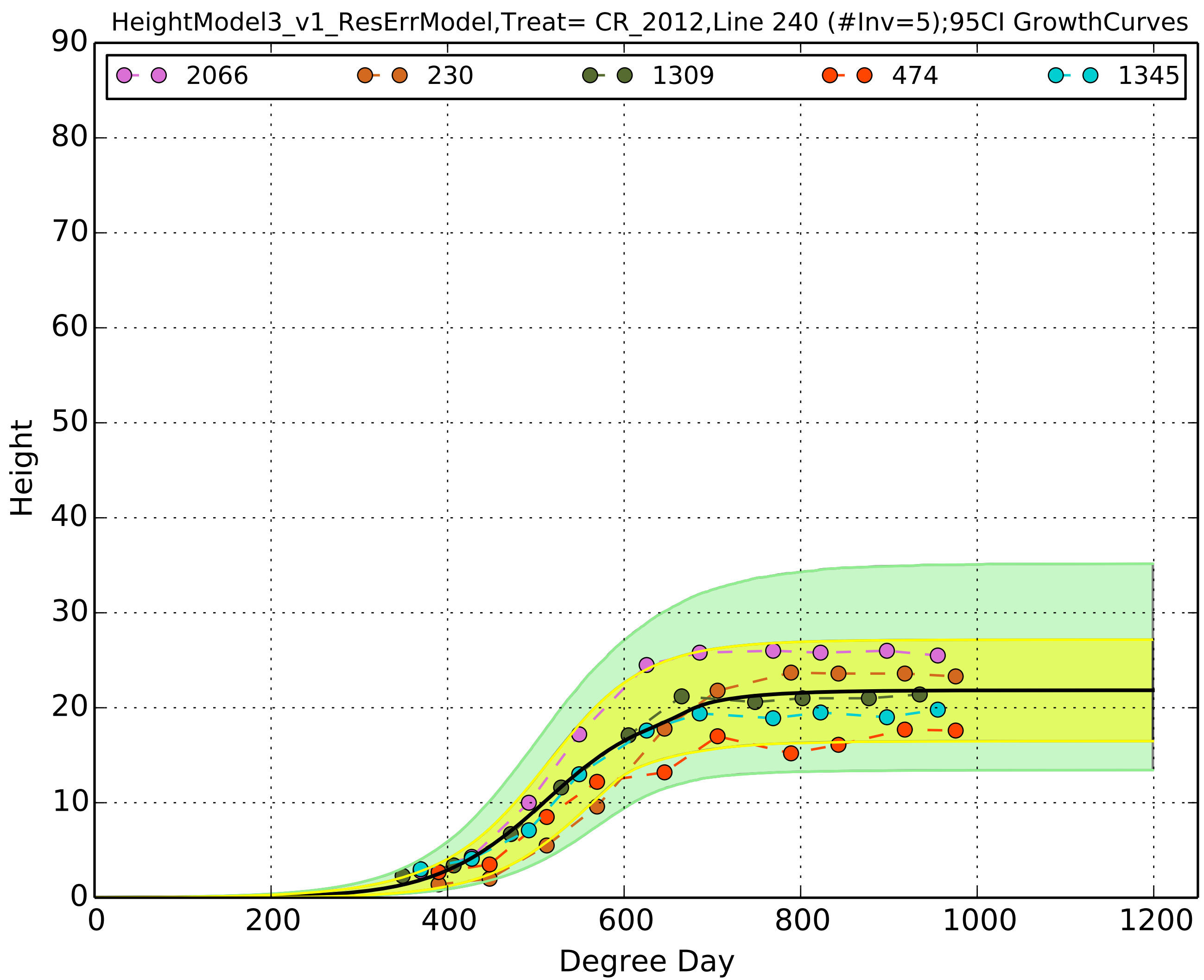

HeightModel3_v1_ResErrModel,Treat= CR_2012,Line 240 (#Inv=5);95CI GrowthCurves
2066
230
1309
474
1345
Height
Degree Day
0
10
20
30
40
50
60
70
80
90
0
200
400
600
800
1000
1200

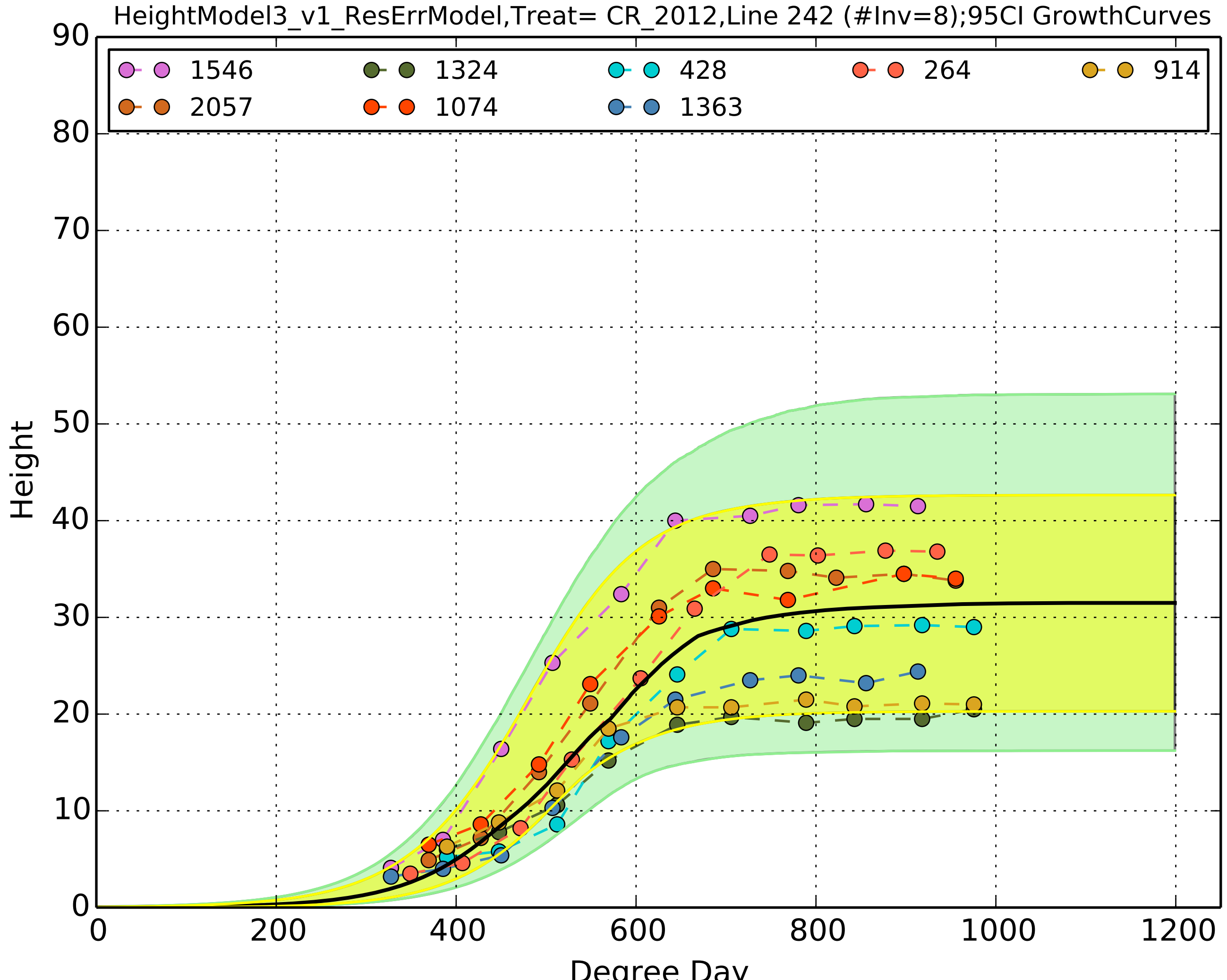

HeightModel3_v1_ResErrModel,Treat= CR_2012,Line 242 (#Inv=8);95CI GrowthCurves
1546
1324
428
264
914
2057
1074
1363
Height
Degree Day
0
10
20
30
40
50
60
70
80
90
0
200
400
600
800
1000
1200

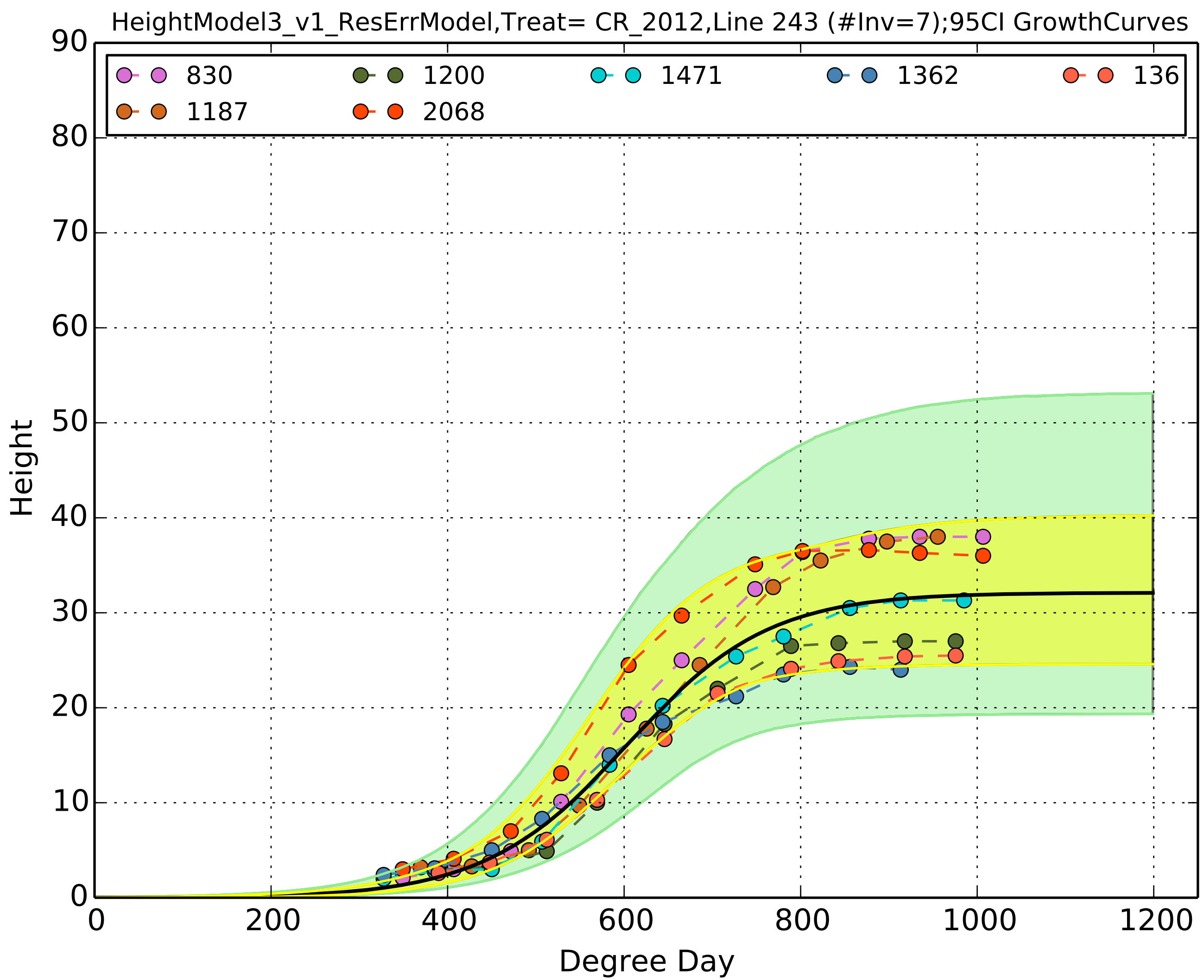

HeightModel3_v1_ResErrModel,Treat= CR_2012,Line 243 (#Inv=7);95CI GrowthCurves
830
1200
1471
1362
136
1187
2068
Height
Degree Day
0
10
20
30
40
50
60
70
80
90
0
200
400
600
800
1000
1200

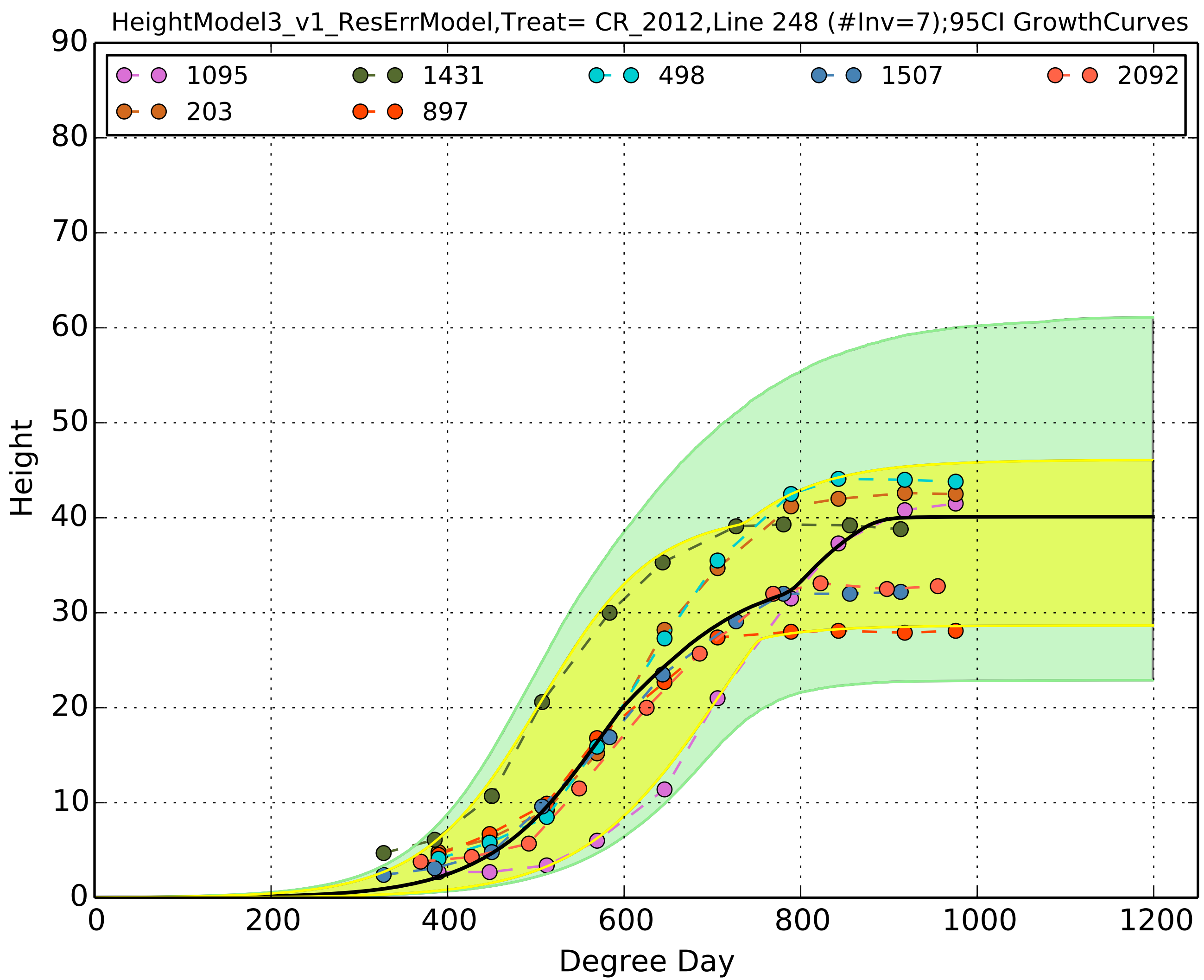
HeightModel3_v1_ResErrModel,Treat= CR_2012,Line 248 (#Inv=7);95CI GrowthCurves
1095
1431
498
1507
2092
203
897
Height
Degree Day
0
10
20
30
40
50
60
70
80
90
0
200
400
600
800
1000
1200

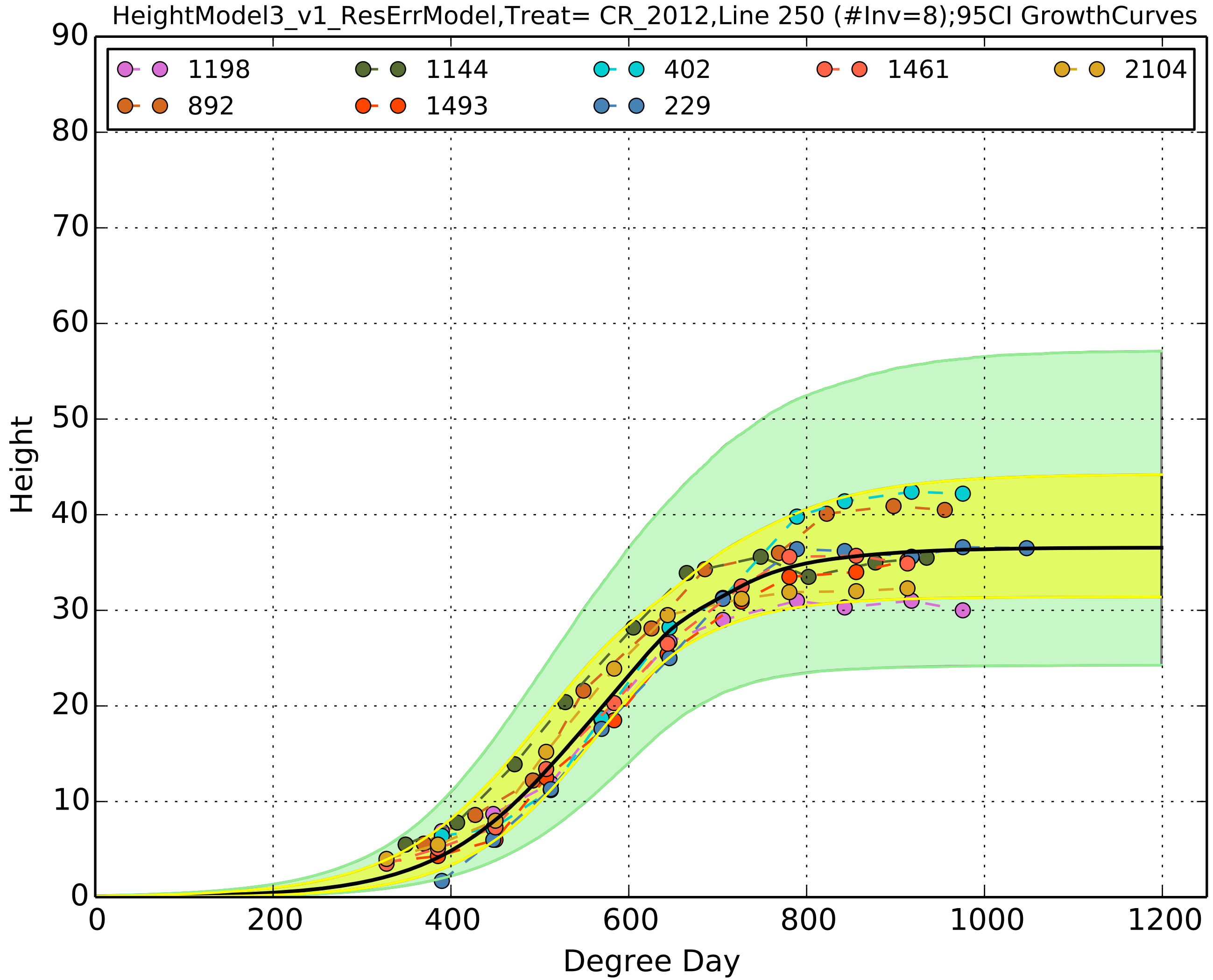
HeightModel3_v1_ResErrModel,Treat= CR_2012,Line 250 (#Inv=8);95CI GrowthCurves
1198
1144
402
1461
2104
892
1493
229
Height
Degree Day
0
10
20
30
40
50
60
70
80
90
0
200
400
600
800
1000
1200

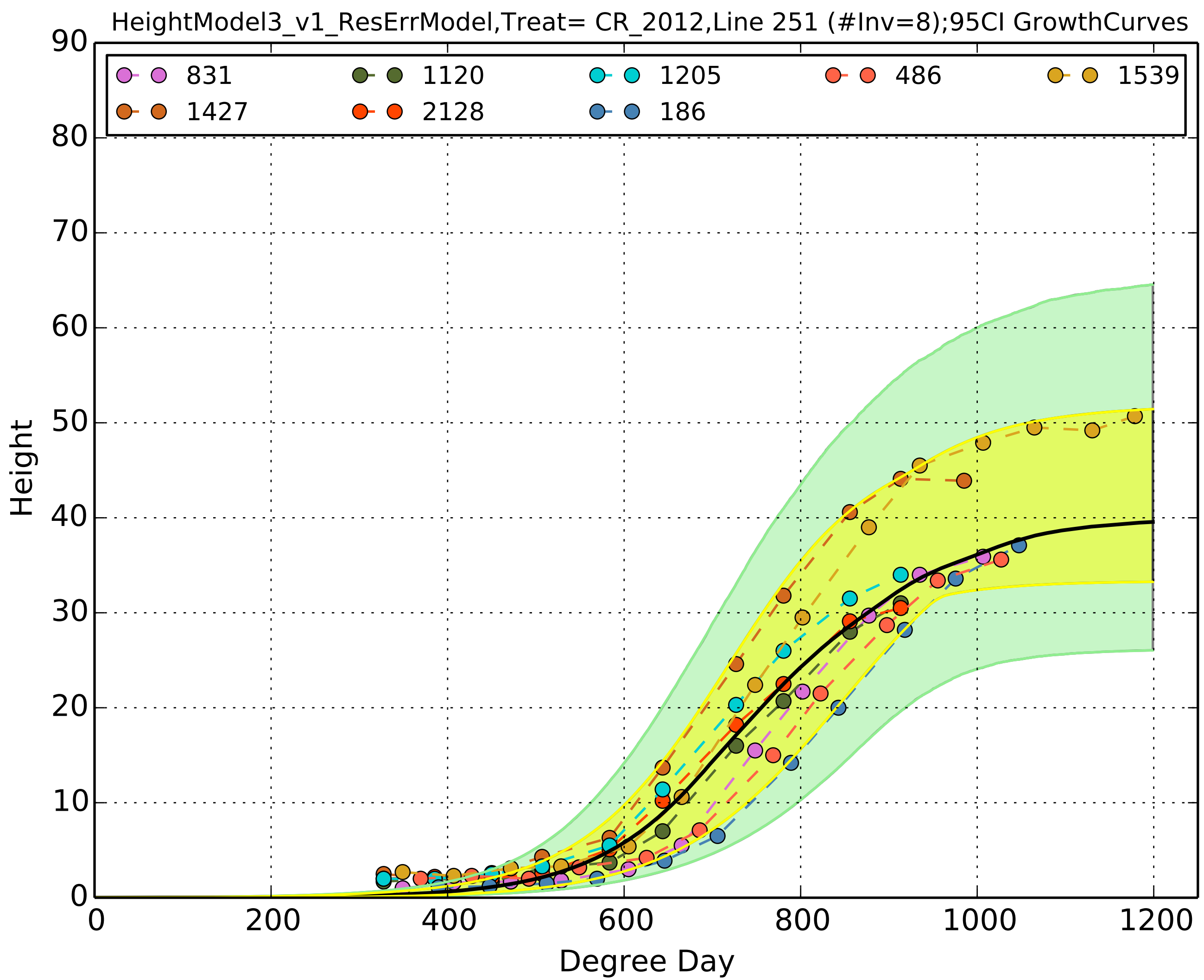

HeightModel3_v1_ResErrModel,Treat= CR_2012,Line 251 (#Inv=8);95CI GrowthCurves
831
1120
1205
486
1539
1427
2128
186
Height
Degree Day
0
10
20
30
40
50
60
70
80
90
0
200
400
600
800
1000
1200

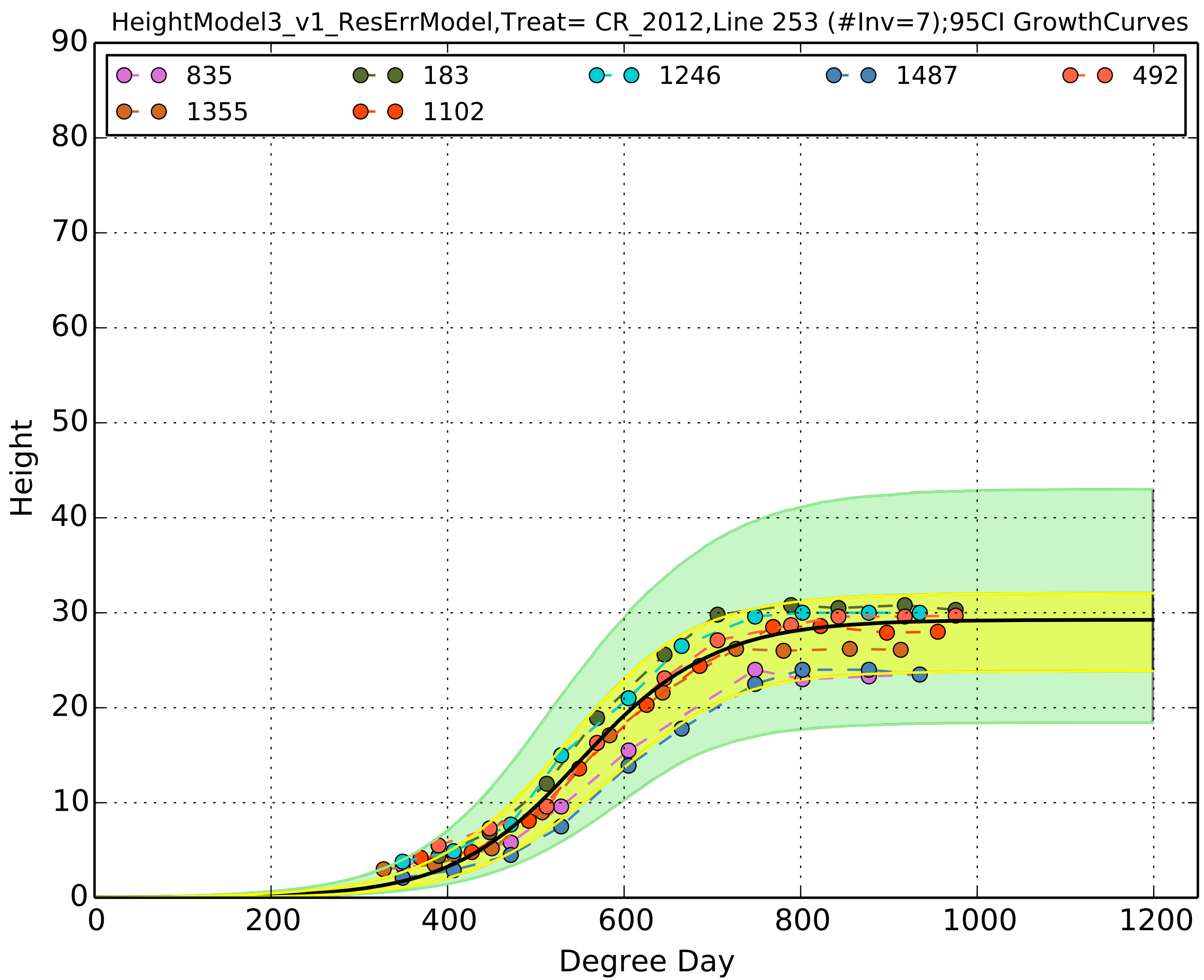

HeightModel3_v1_ResErrModel,Treat= CR_2012,Line 253 (#Inv=7);95CI GrowthCurves
835
183
1246
1487
492
1355
1102
Height
Degree Day
0
10
20
30
40
50
60
70
80
90
0
200
400
600
800
1000
1200

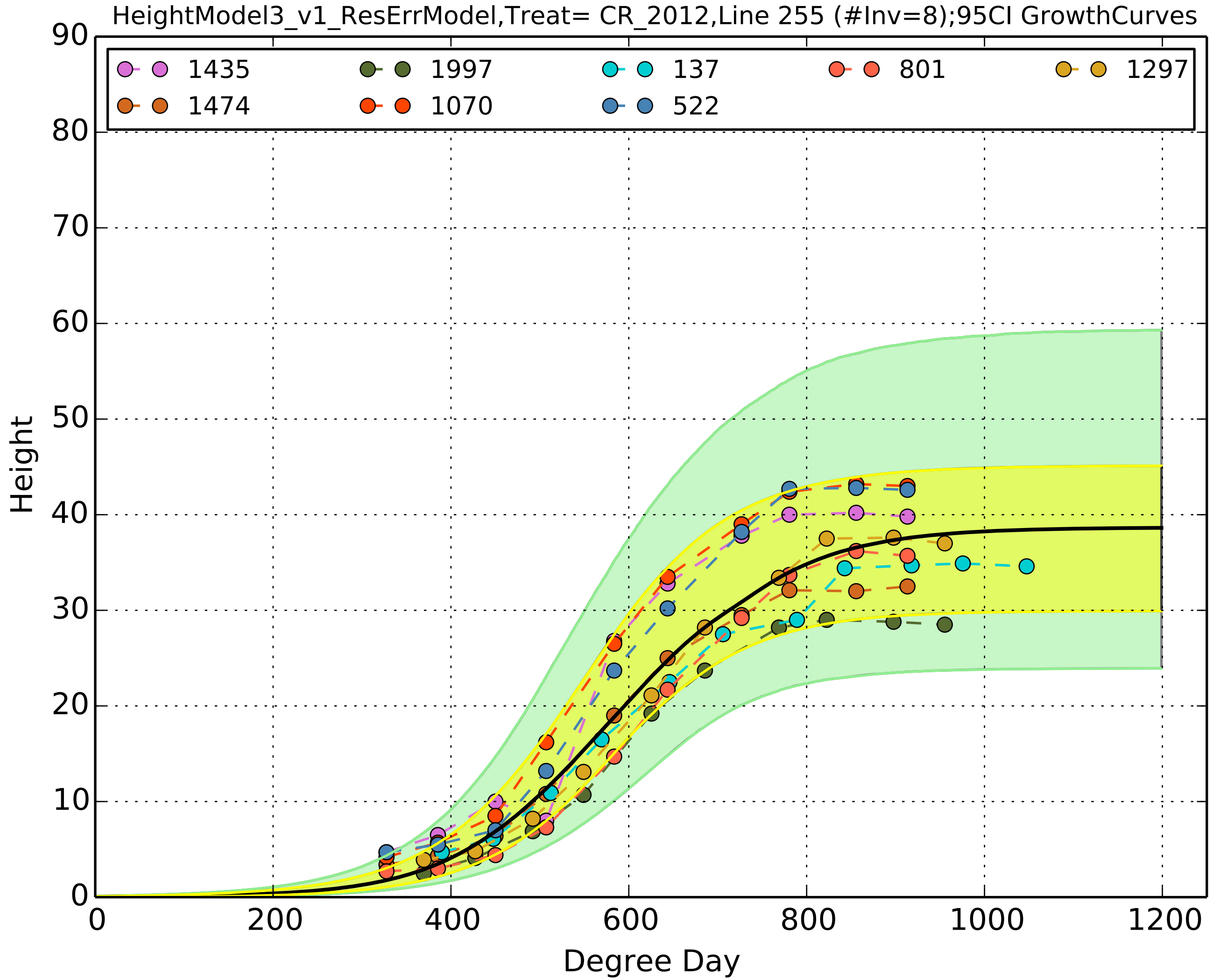
HeightModel3_v1_ResErrModel,Treat= CR_2012,Line 255 (#Inv=8);95CI GrowthCurves
1435
1997
137
801
1297
1474
1070
522
Height
Degree Day
0
10
20
30
40
50
60
70
80
90
0
200
400
600
800
1000
1200

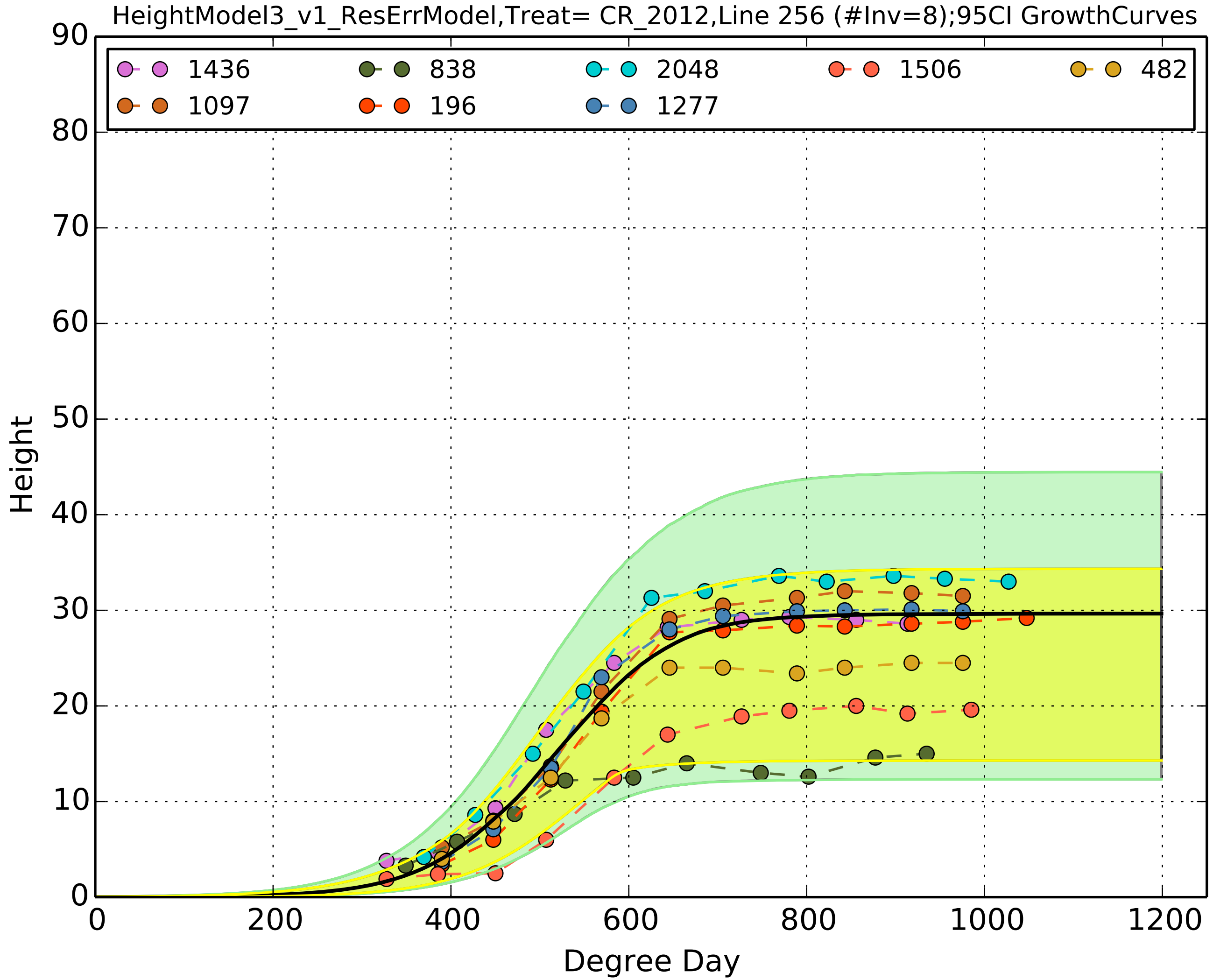

HeightModel3_v1_ResErrModel,Treat= CR_2012,Line 256 (#Inv=8);95CI GrowthCurves
1436
838
2048
1506
482
1097
196
1277
Height
Degree Day
0
10
20
30
40
50
60
70
80
90
0
200
400
600
800
1000
1200

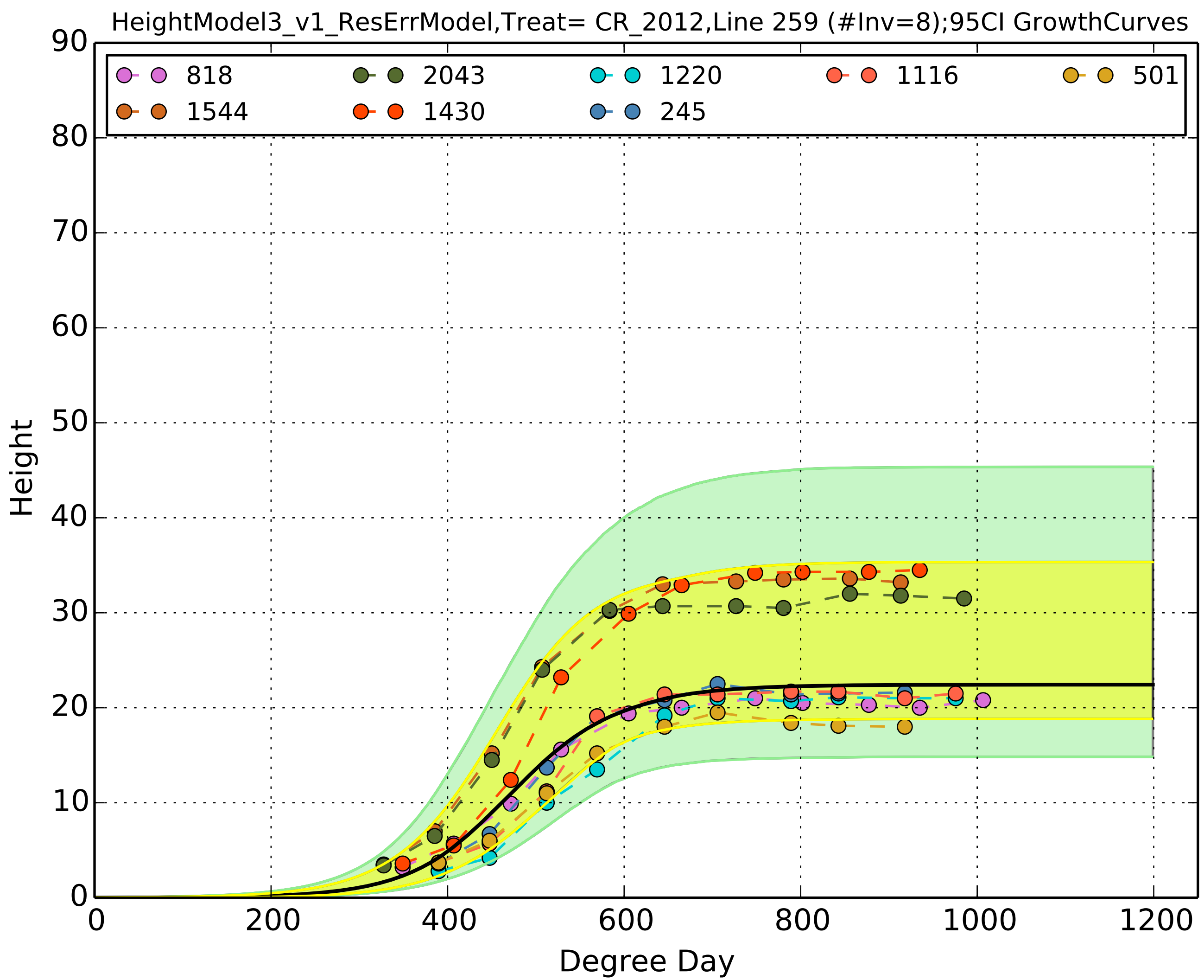
HeightModel3_v1_ResErrModel,Treat= CR_2012,Line 259 (#Inv=8);95CI GrowthCurves
818
2043
1220
1116
501
1544
1430
245
Height
Degree Day
0
10
20
30
40
50
60
70
80
90
0
200
400
600
800
1000
1200

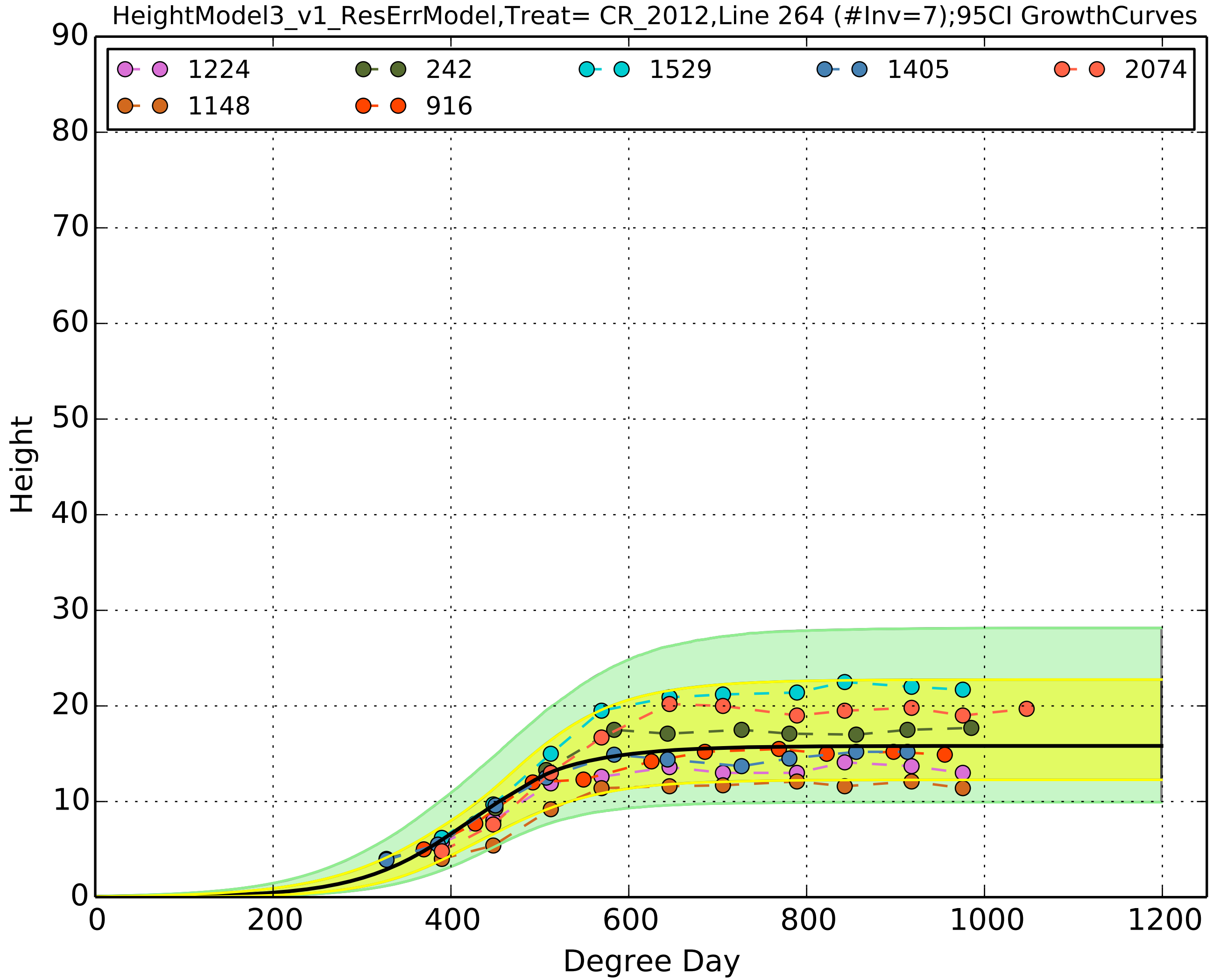
HeightModel3_v1_ResErrModel,Treat= CR_2012,Line 264 (#Inv=7);95CI GrowthCurves
1224
242
1529
1405
2074
1148
916
Height
Degree Day
0
10
20
30
40
50
60
70
80
90
0
200
400
600
800
1000
1200

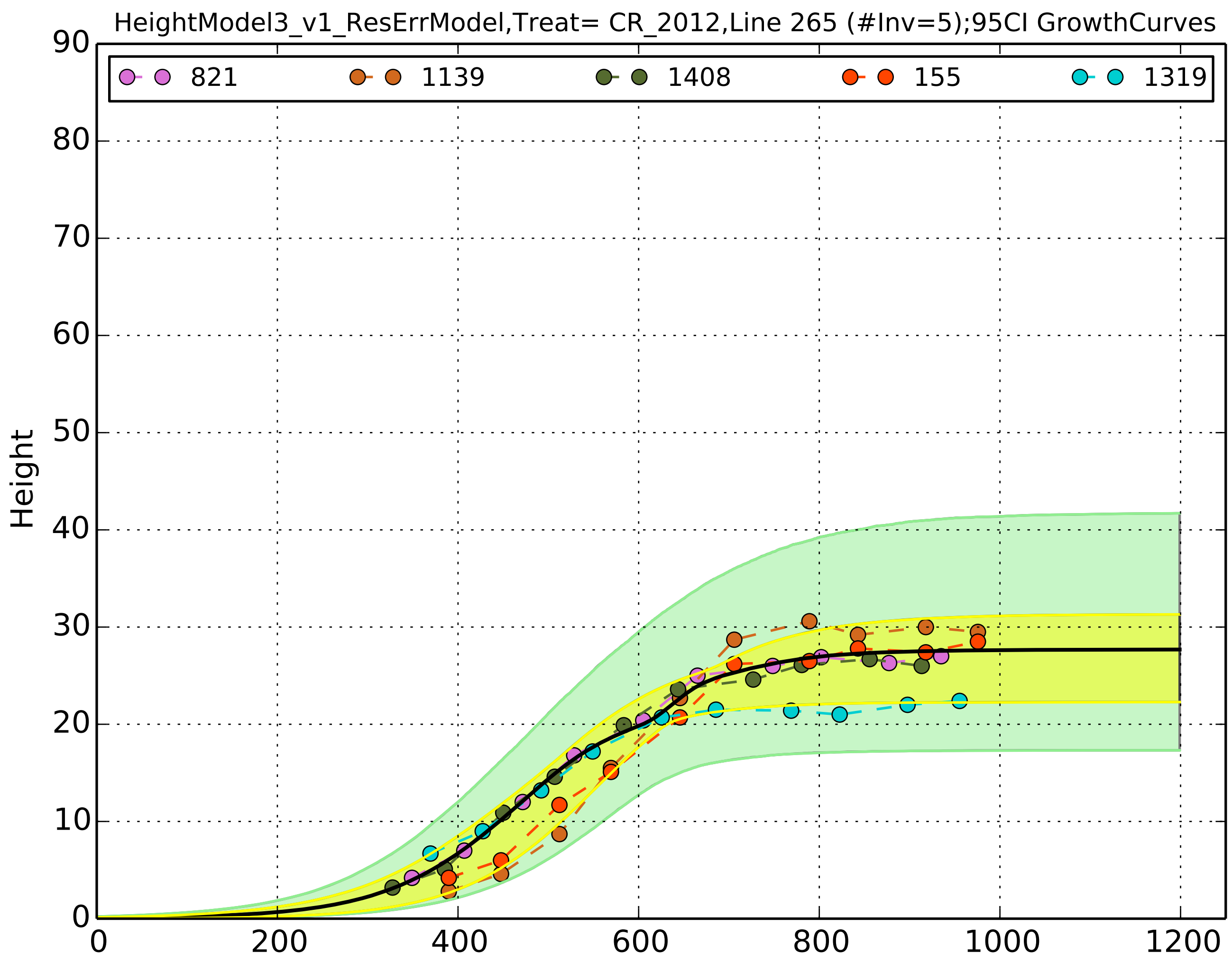

HeightModel3_v1_ResErrModel,Treat= CR_2012,Line 265 (#Inv=5);95CI GrowthCurves
821
1139
1408
155
1319
Height
Degree Day
0
10
20
30
40
50
60
70
80
90
0
200
400
600
800
1000
1200

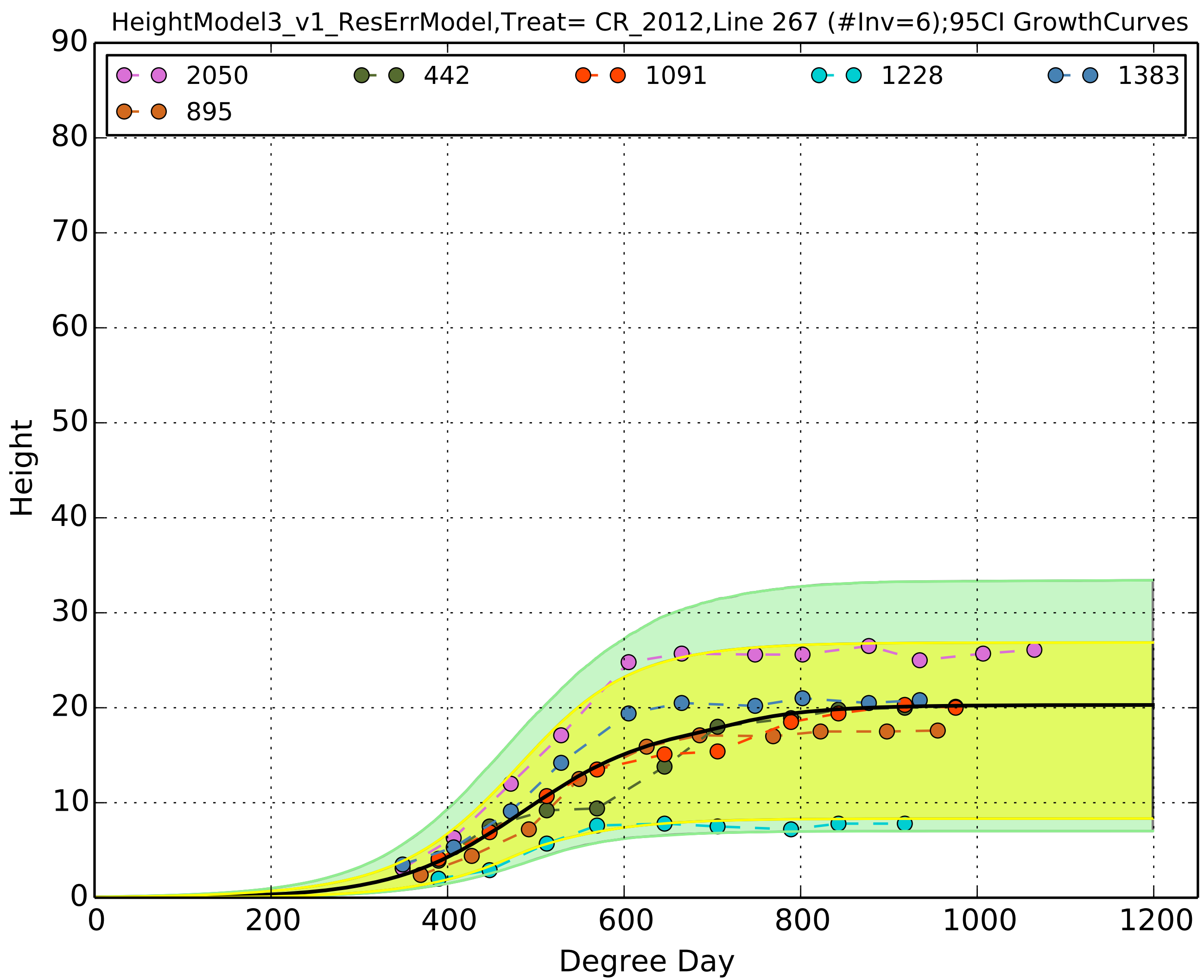

HeightModel3_v1_ResErrModel,Treat= CR_2012,Line 267 (#Inv=6);95CI GrowthCurves
2050
442
1091
1228
1383
895
Height
Degree Day
0
10
20
30
40
50
60
70
80
90
0
200
400
600
800
1000
1200

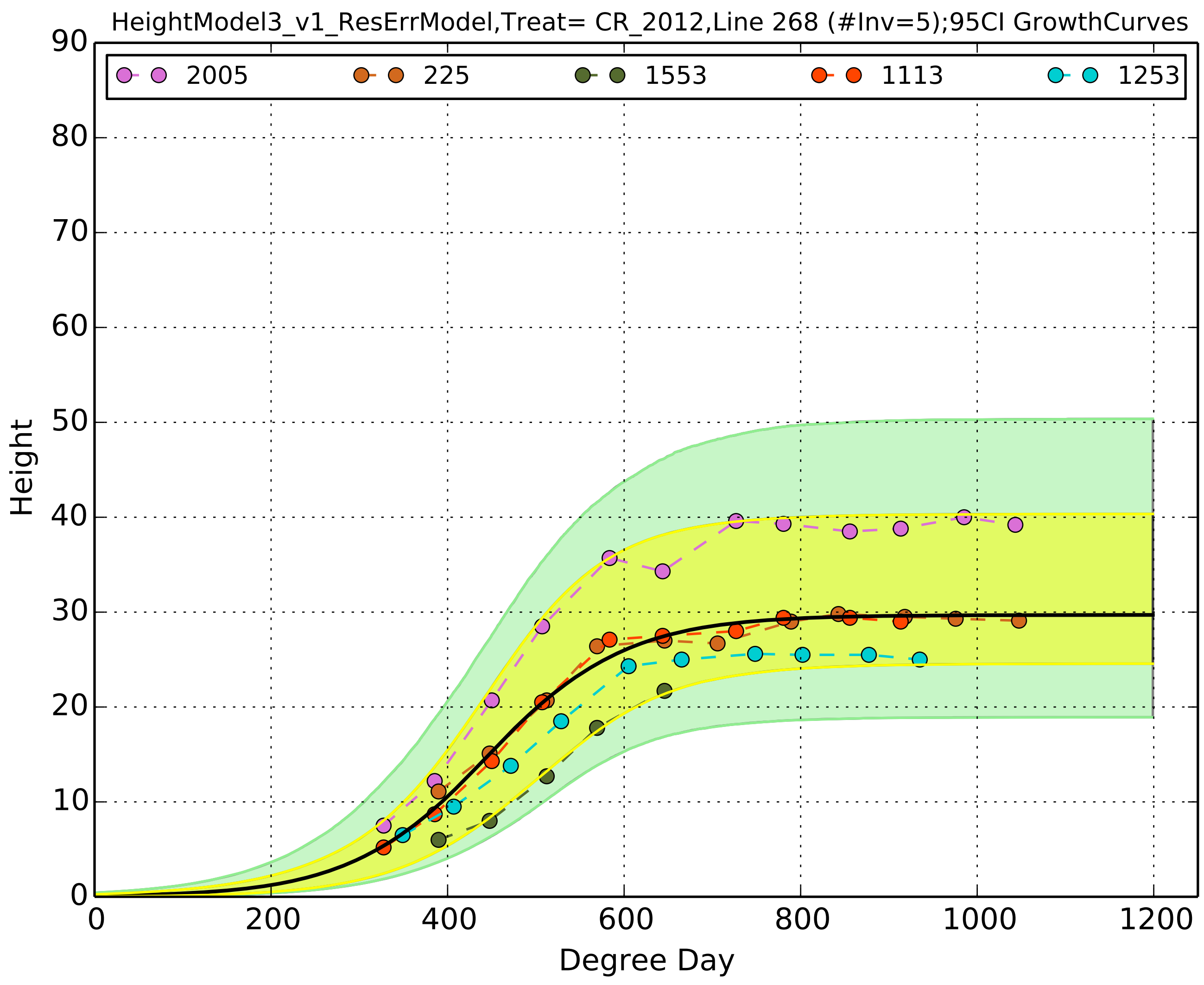

HeightModel3_v1_ResErrModel,Treat= CR_2012,Line 268 (#Inv=5);95CI GrowthCurves
2005
225
1553
1113
1253
Height
Degree Day
0
10
20
30
40
50
60
70
80
90
0
200
400
600
800
1000
1200

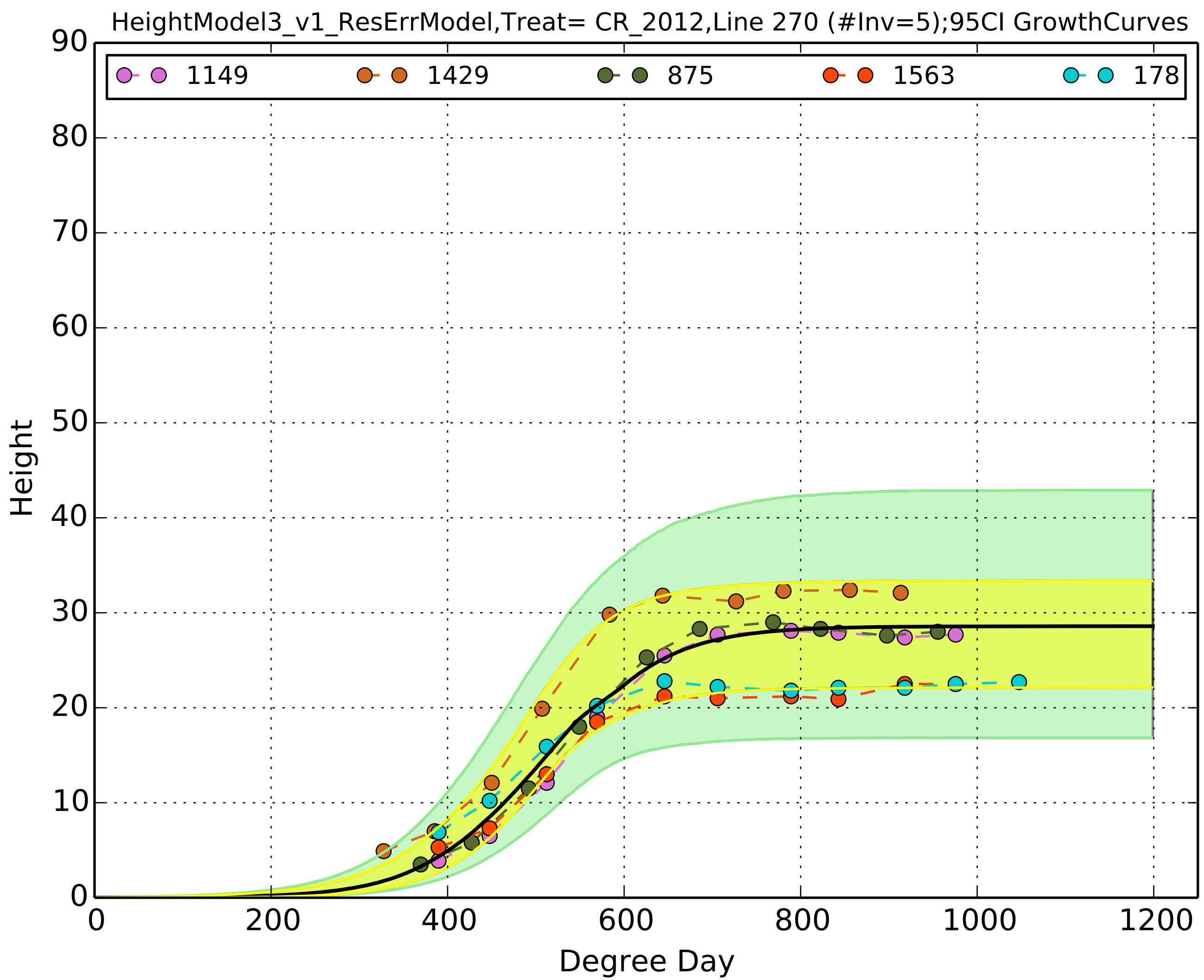

HeightModel3_v1_ResErrModel,Treat= CR_2012,Line 270 (#Inv=5);95CI GrowthCurves
1149
1429
875
1563
178
Height
Degree Day
0
10
20
30
40
50
60
70
80
90
0
200
400
600
800
1000
1200

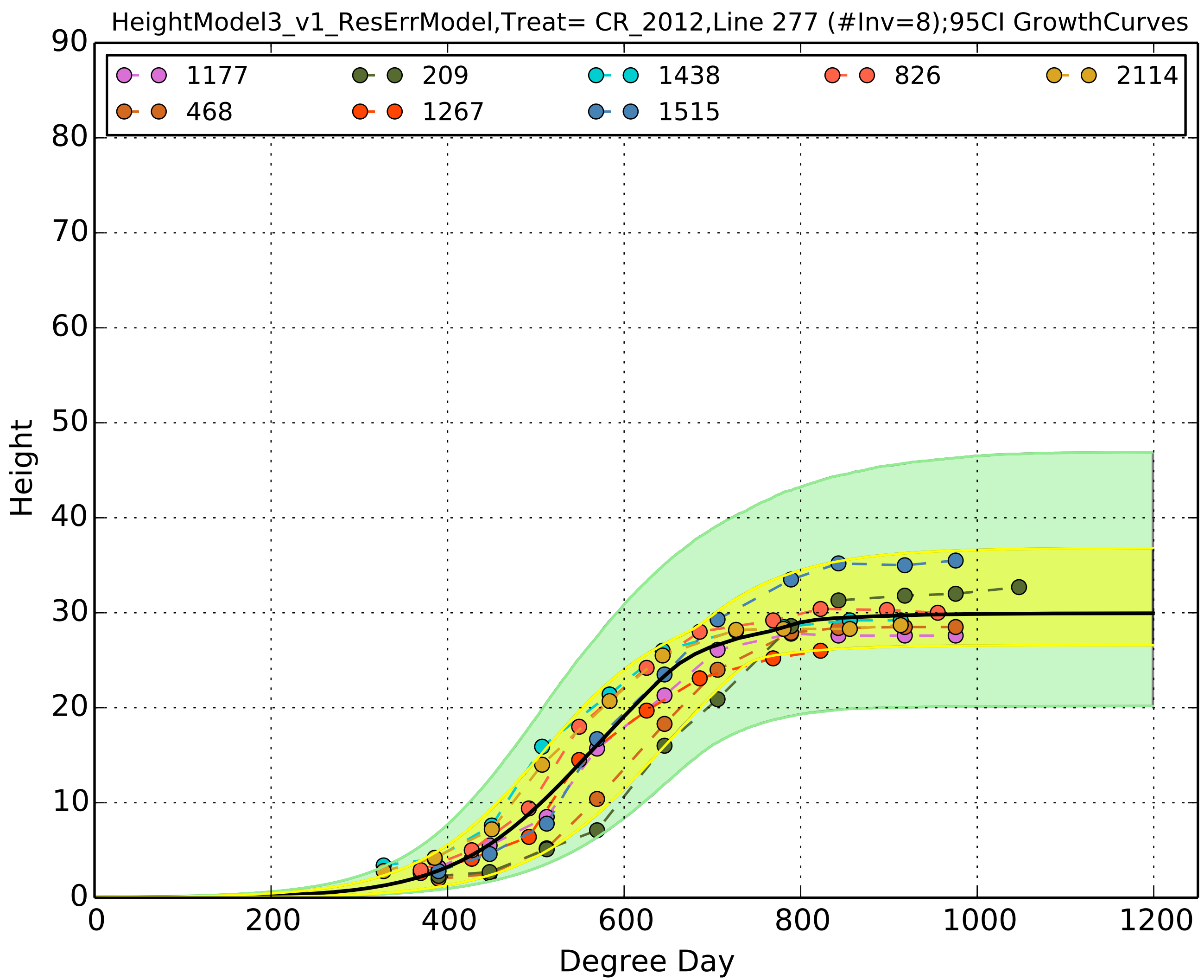
HeightModel3_v1_ResErrModel,Treat= CR_2012,Line 277 (#Inv=8);95CI GrowthCurves
1177
209
1438
826
2114
468
1267
1515
Height
Degree Day
0
10
20
30
40
50
60
70
80
90
0
200
400
600
800
1000
1200

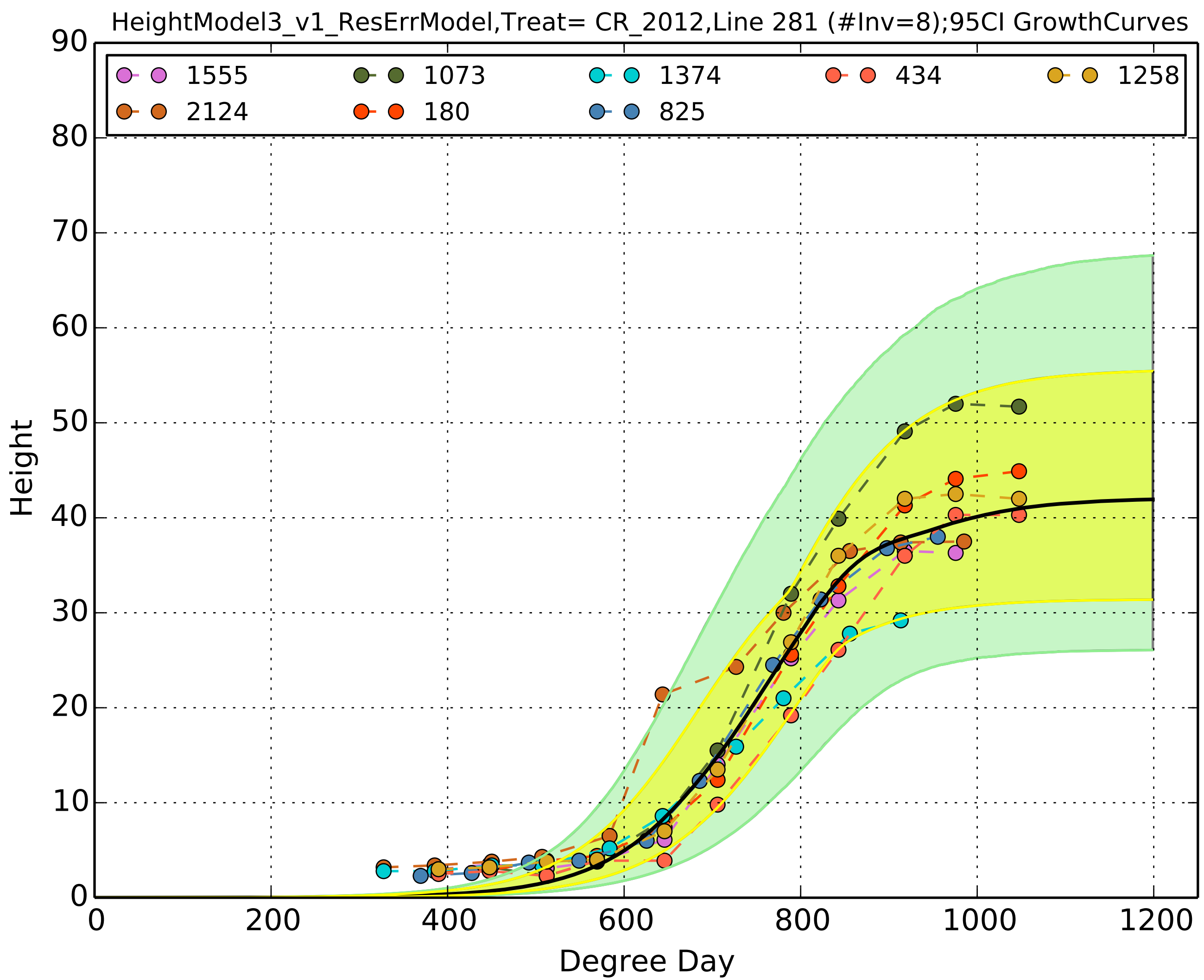
HeightModel3_v1_ResErrModel,Treat= CR_2012,Line 281 (#Inv=8);95CI GrowthCurves
1555
1073
1374
434
1258
2124
180
825
Height
Degree Day
0
10
20
30
40
50
60
70
80
90
0
200
400
600
800
1000
1200

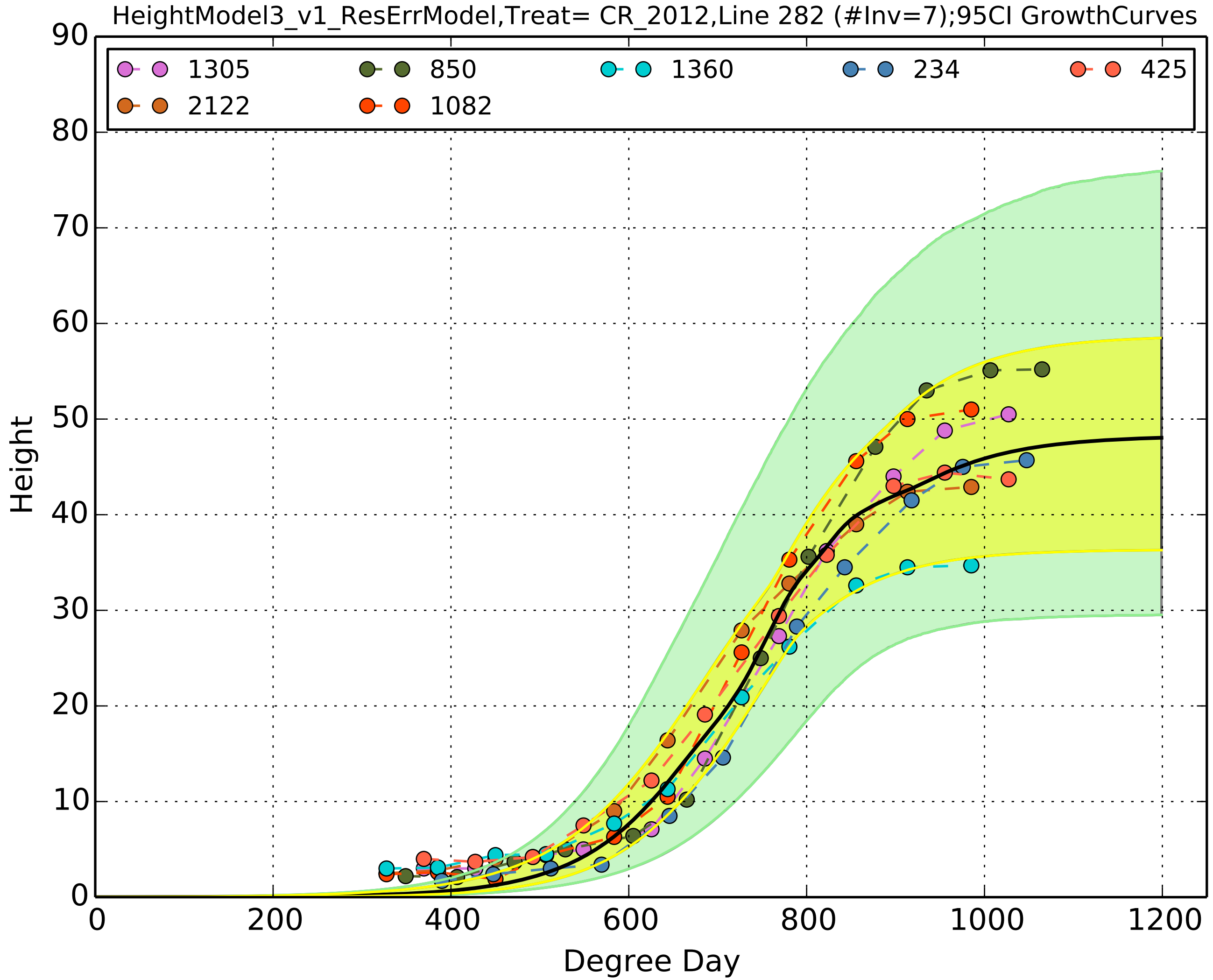

HeightModel3_v1_ResErrModel,Treat= CR_2012,Line 282 (#Inv=7);95CI GrowthCurves
1305
850
1360
234
425
2122
1082
Height
Degree Day
0
10
20
30
40
50
60
70
80
90
0
200
400
600
800
1000
1200

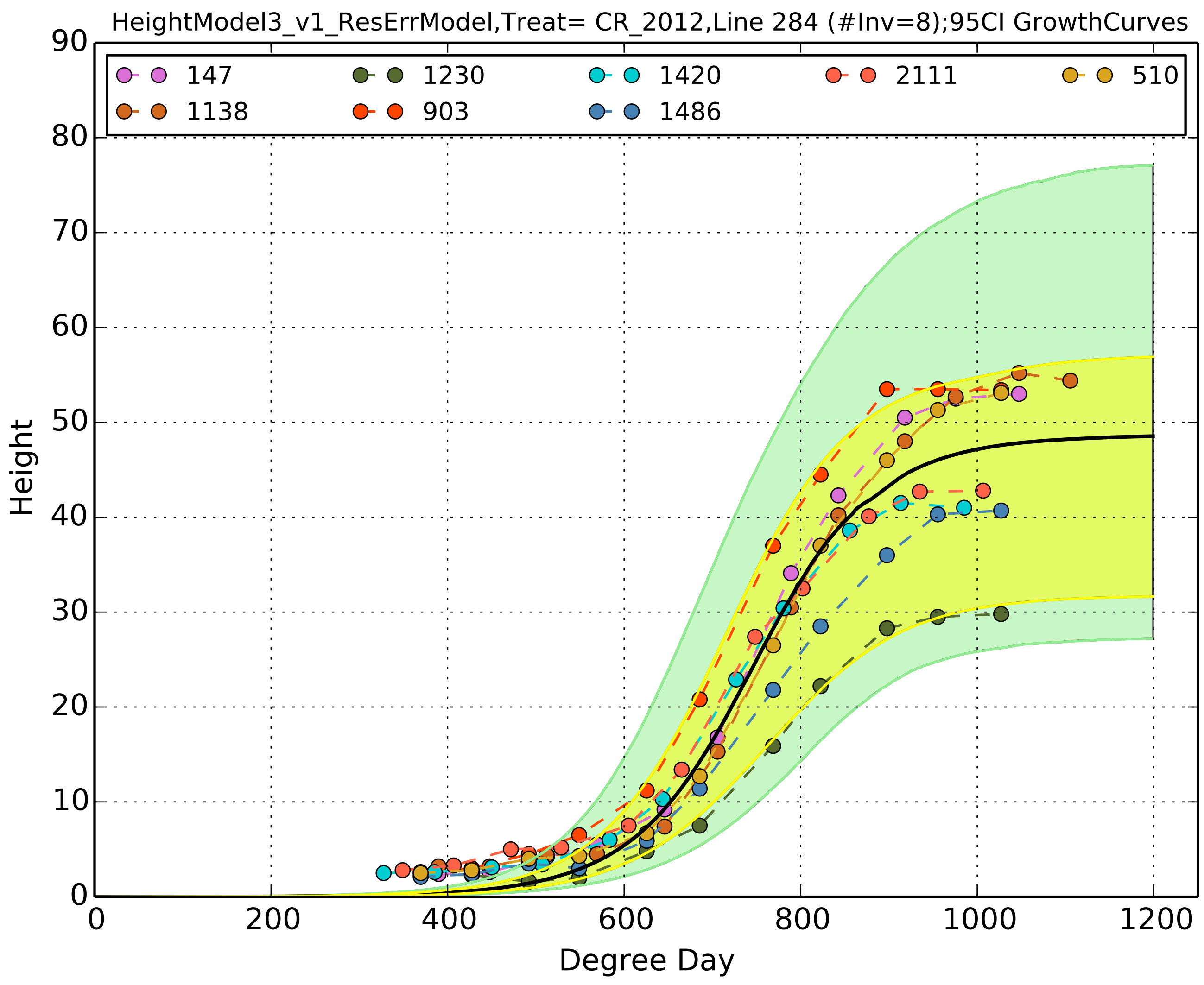

HeightModel3_v1_ResErrModel,Treat= CR_2012,Line 284 (#Inv=8);95CI GrowthCurves
147
1230
1420
2111
510
1138
903
1486
Height
Degree Day
0
10
20
30
40
50
60
70
80
90
0
200
400
600
800
1000
1200

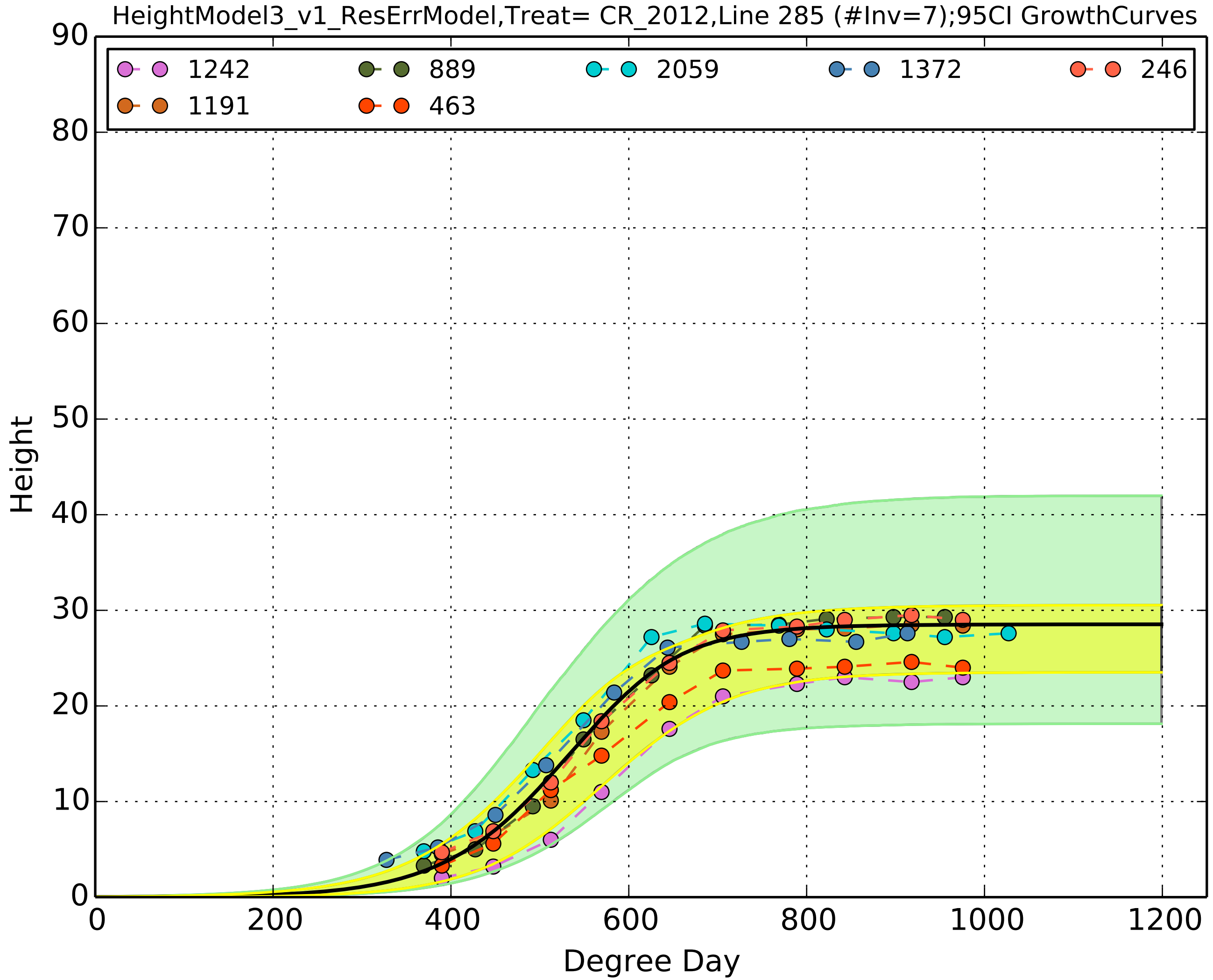

HeightModel3_v1_ResErrModel,Treat= CR_2012,Line 285 (#Inv=7);95CI GrowthCurves
1242
889
2059
1372
246
1191
463
Height
Degree Day
0
10
20
30
40
50
60
70
80
90
0
200
400
600
800
1000
1200

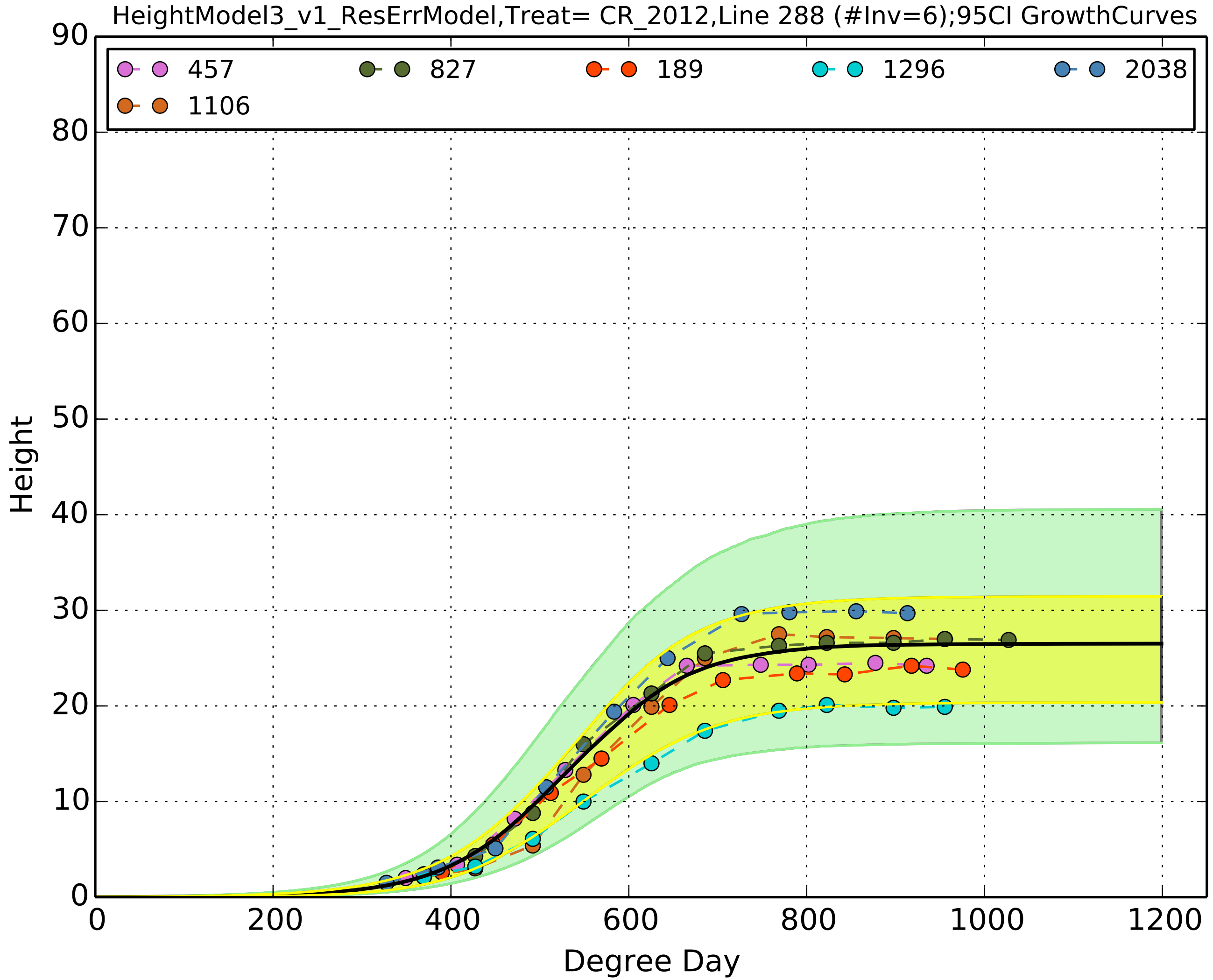
HeightModel3_v1_ResErrModel,Treat= CR_2012,Line 288 (#Inv=6);95CI GrowthCurves
457
827
189
1296
2038
1106
Height
Degree Day
0
10
20
30
40
50
60
70
80
90
0
200
400
600
800
1000
1200

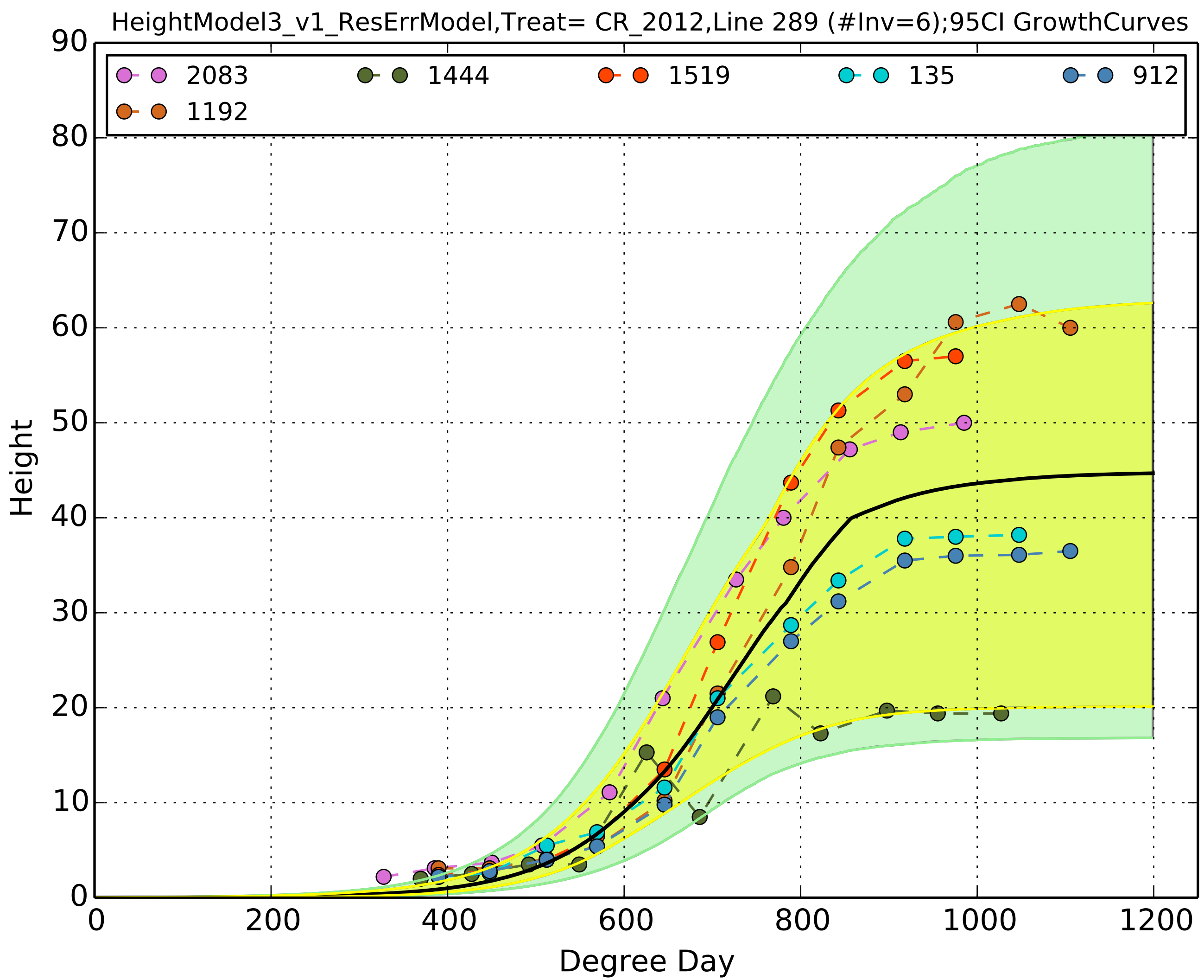

HeightModel3_v1_ResErrModel,Treat= CR_2012,Line 289 (#Inv=6);95CI GrowthCurves
2083
1444
1519
135
912
1192
Height
Degree Day
0
10
20
30
40
50
60
70
80
90
0
200
400
600
800
1000
1200

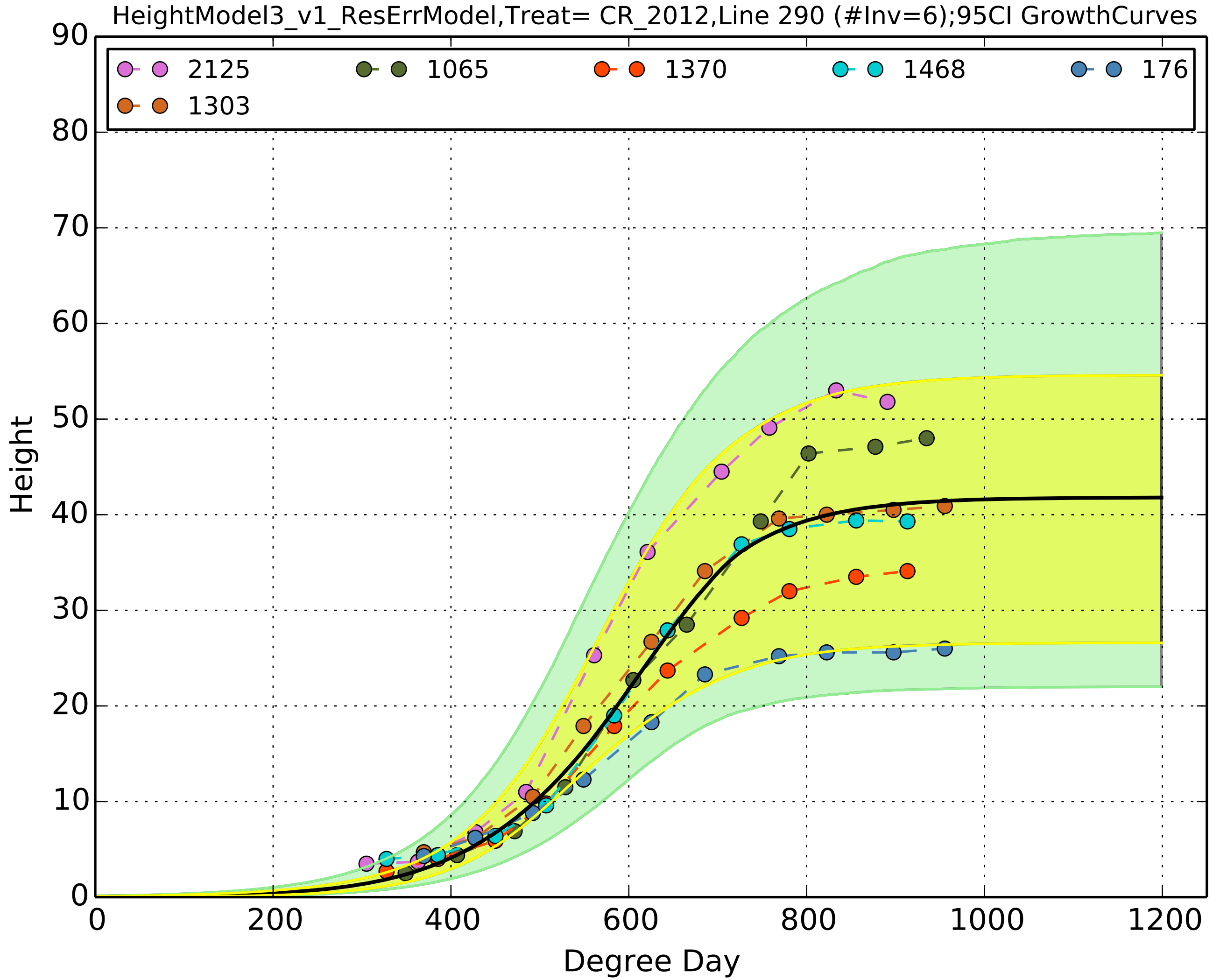

HeightModel3_v1_ResErrModel,Treat= CR_2012,Line 290 (#Inv=6);95CI GrowthCurves
2125
1065
1370
1468
176
1303
Height
Degree Day
0
10
20
30
40
50
60
70
80
90
0
200
400
600
800
1000
1200

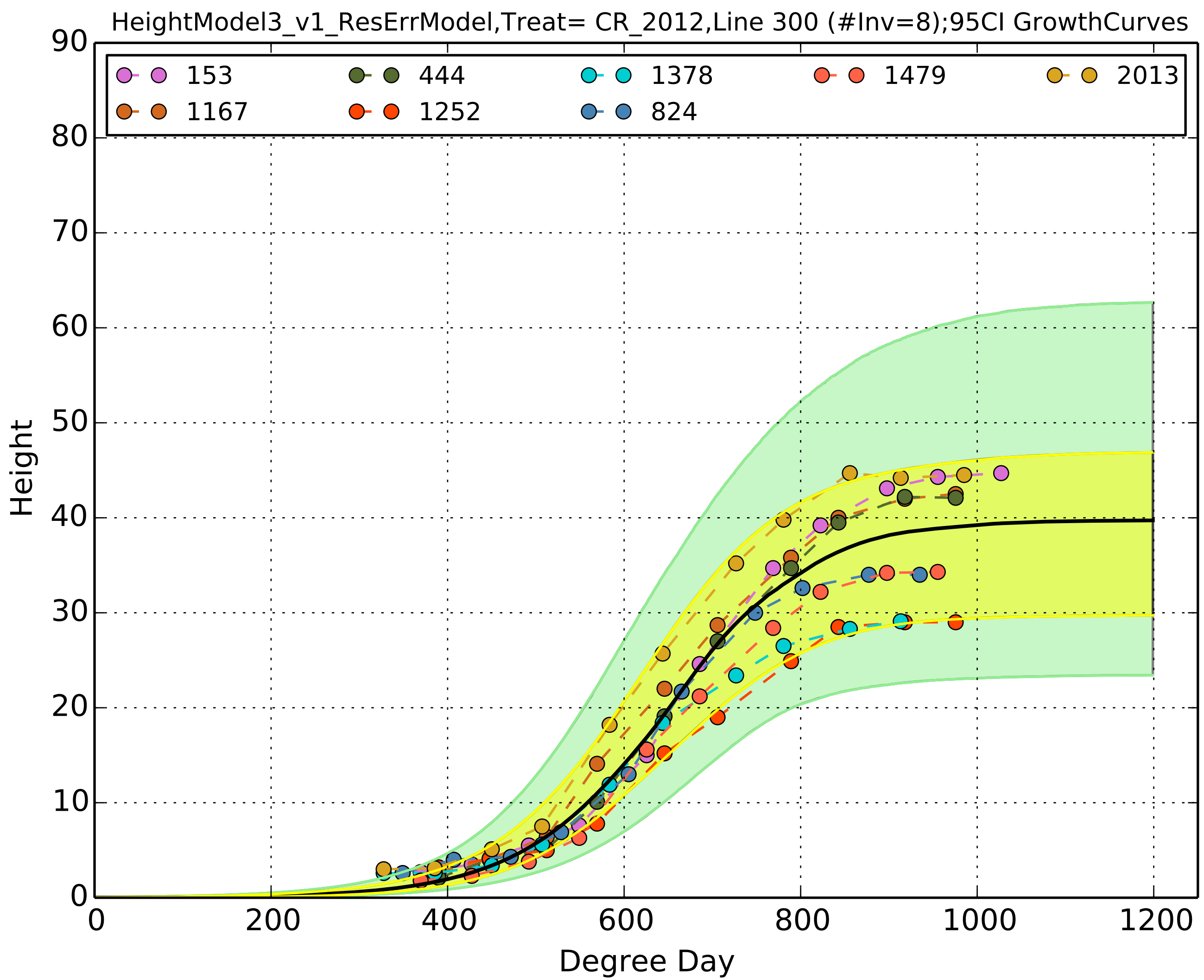

HeightModel3_v1_ResErrModel,Treat= CR_2012,Line 300 (#Inv=8);95CI GrowthCurves
153
444
1378
1479
2013
1167
1252
824
Height
Degree Day
0
10
20
30
40
50
60
70
80
90
0
200
400
600
800
1000
1200

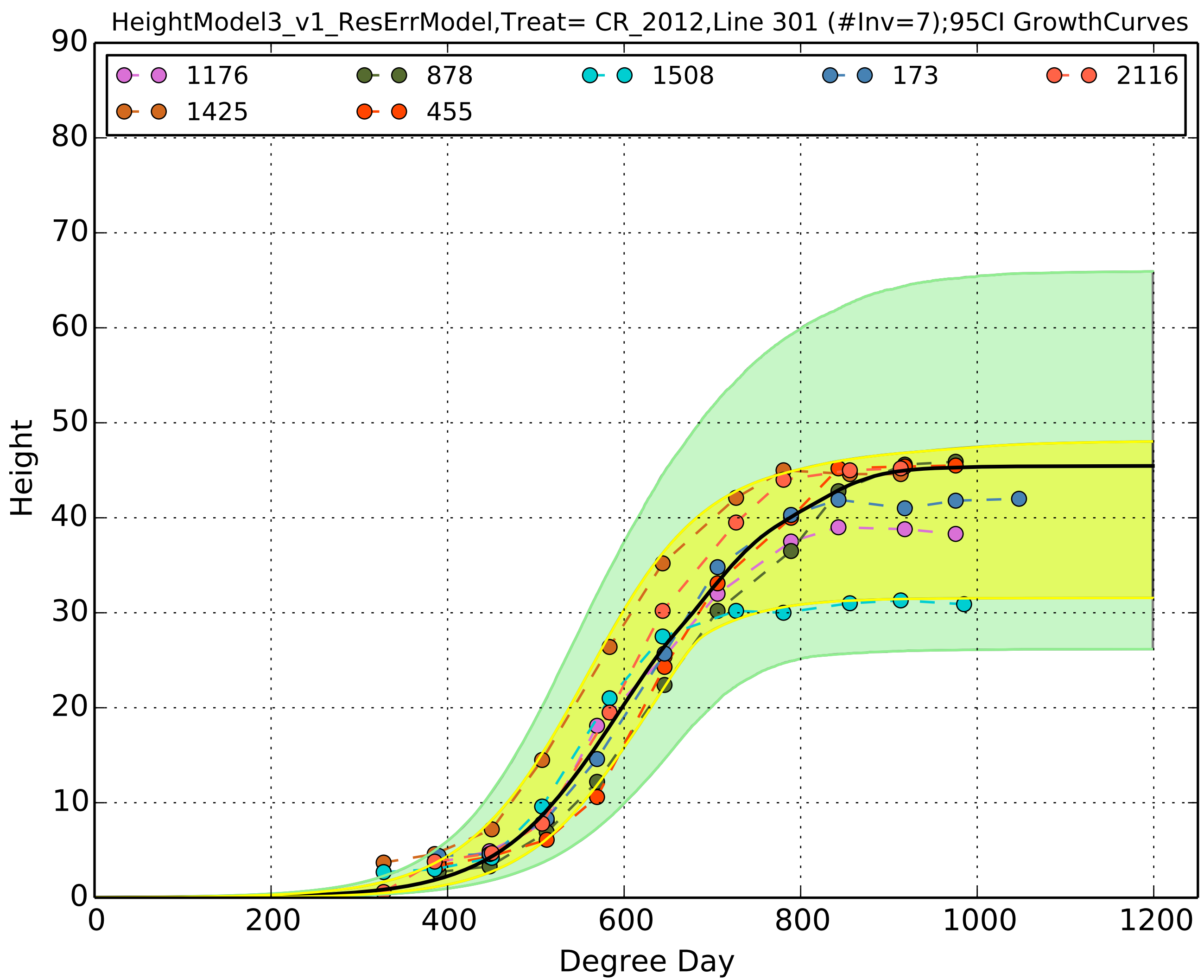
HeightModel3_v1_ResErrModel,Treat= CR_2012,Line 301 (#Inv=7);95CI GrowthCurves
1176
878
1508
173
2116
1425
455
Height
Degree Day
0
10
20
30
40
50
60
70
80
90
0
200
400
600
800
1000
1200

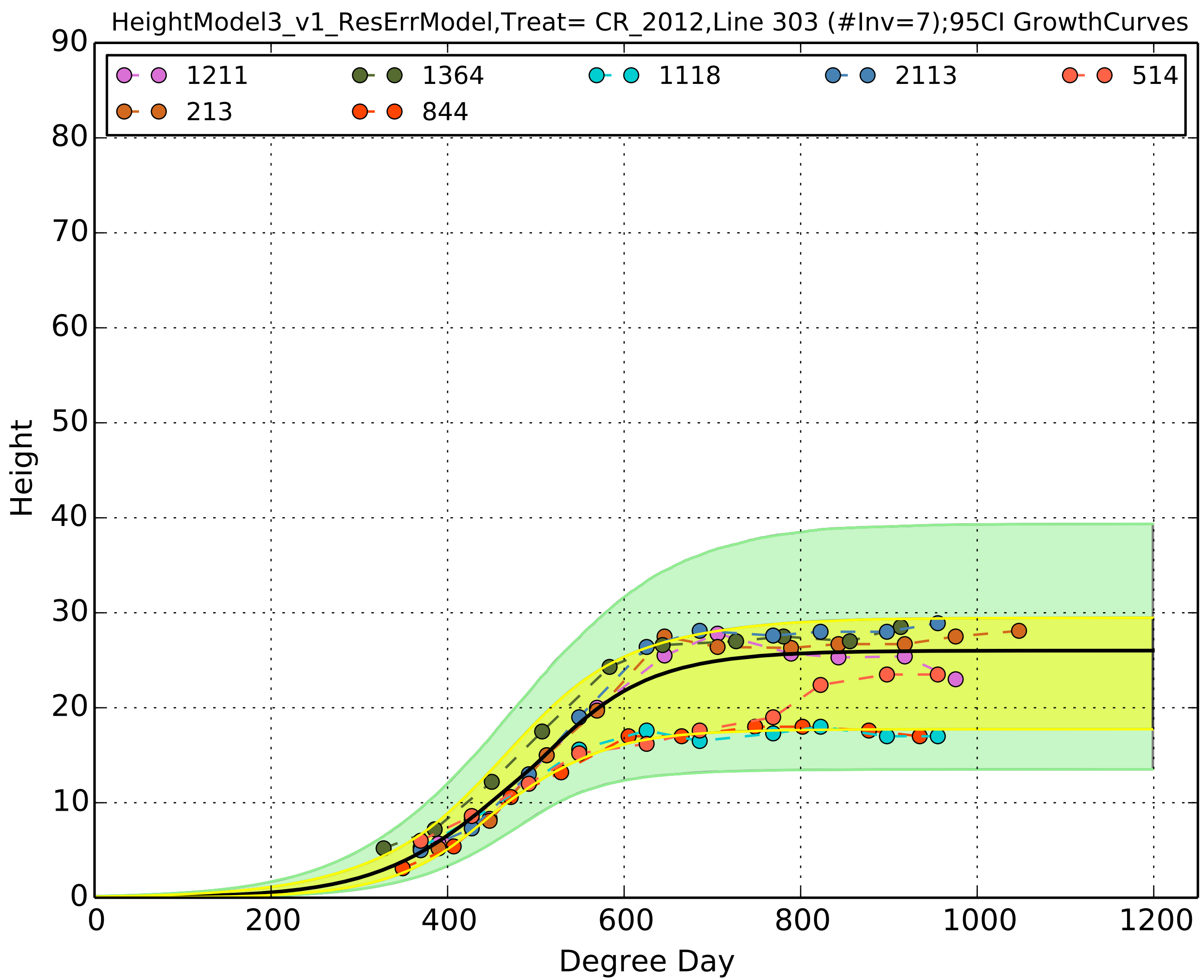

HeightModel3_v1_ResErrModel,Treat= CR_2012,Line 303 (#Inv=7);95CI GrowthCurves
1211
1364
1118
2113
514
213
844
Height
Degree Day
0
10
20
30
40
50
60
70
80
90
0
200
400
600
800
1000
1200

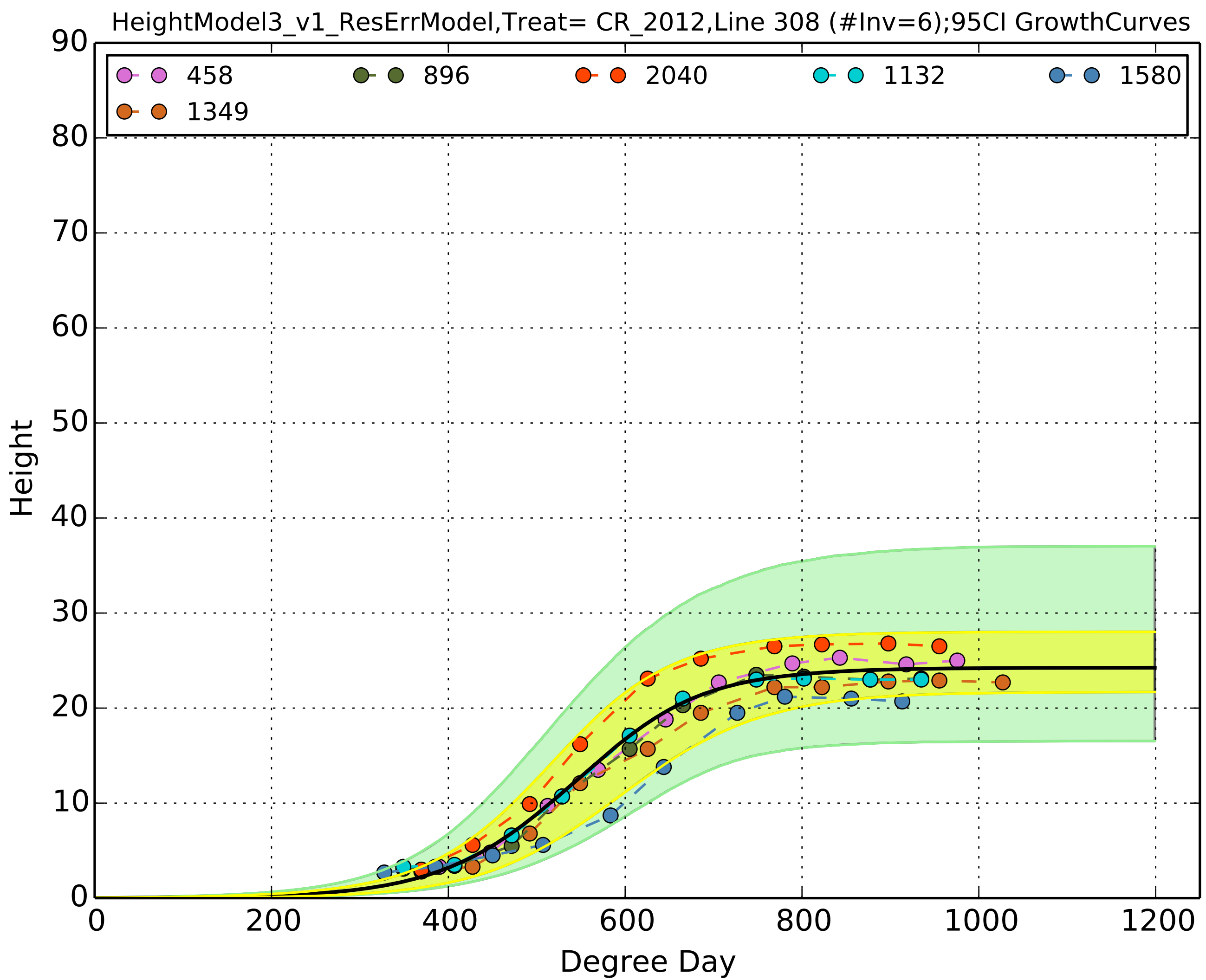

HeightModel3_v1_ResErrModel,Treat= CR_2012,Line 308 (#Inv=6);95CI GrowthCurves
458
896
2040
1132
1580
1349
Height
Degree Day
0
10
20
30
40
50
60
70
80
90
0
200
400
600
800
1000
1200

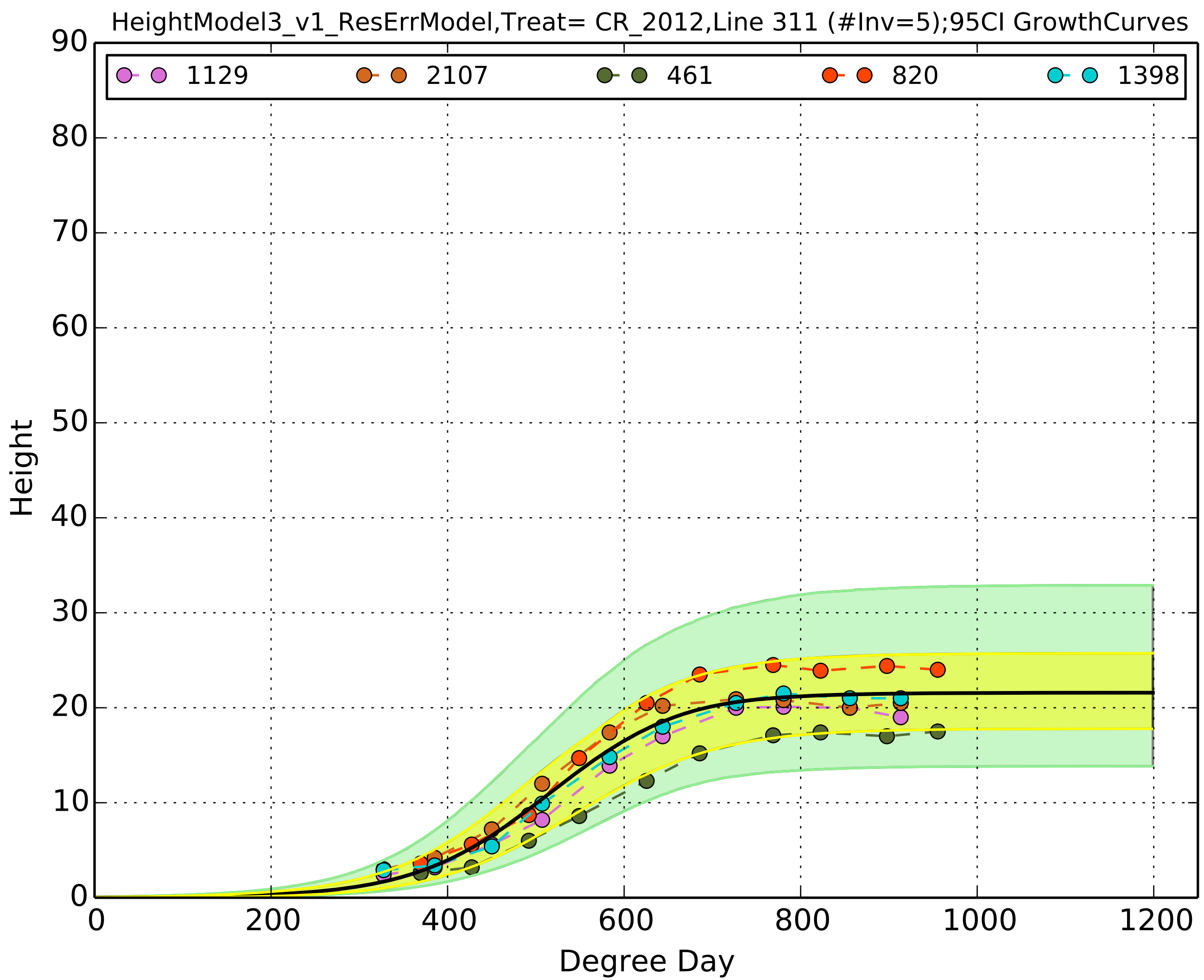
HeightModel3_v1_ResErrModel,Treat= CR_2012,Line 311 (#Inv=5);95CI GrowthCurves
1129
2107
461
820
1398
Height
Degree Day
0
10
20
30
40
50
60
70
80
90
0
200
400
600
800
1000
1200

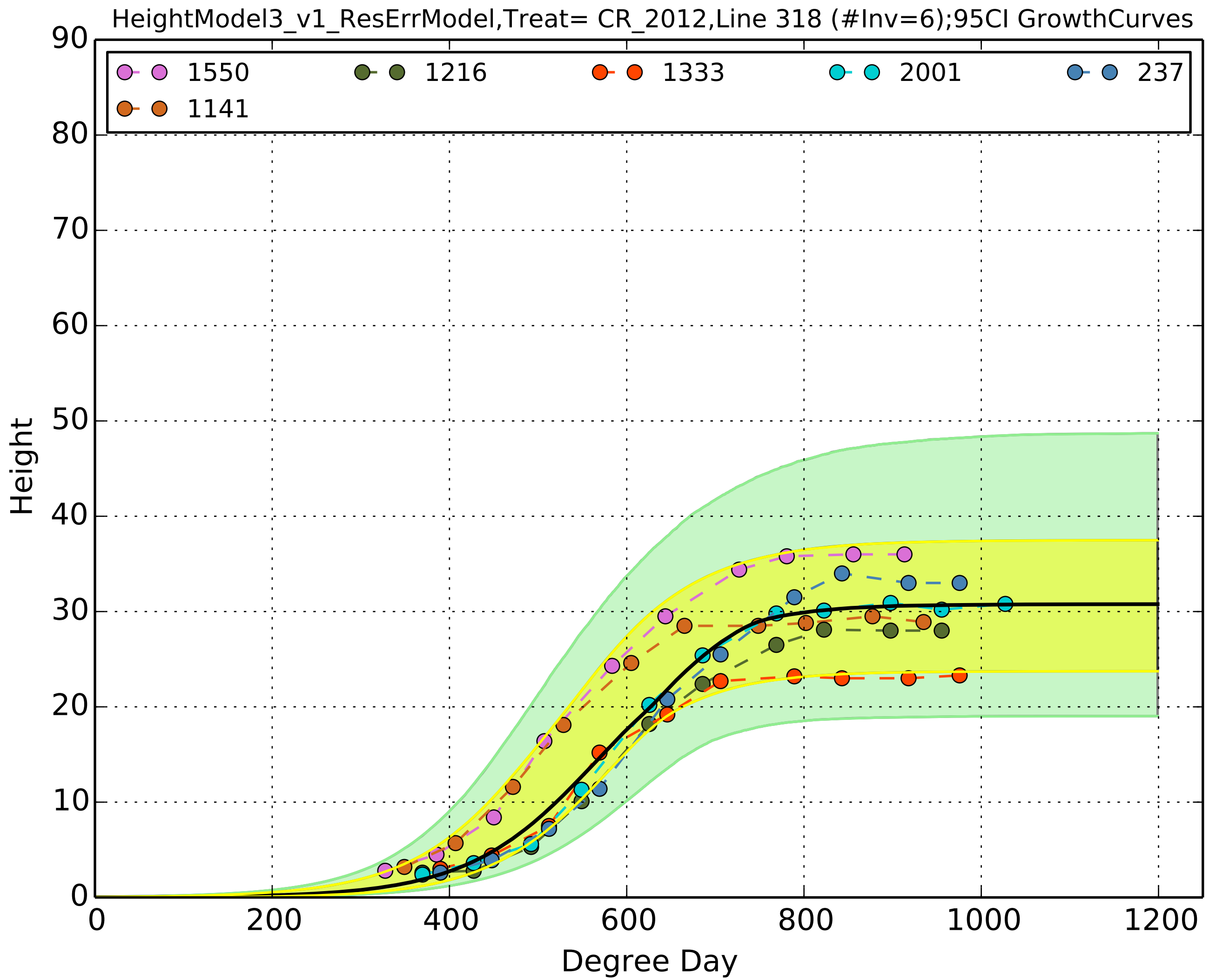

HeightModel3_v1_ResErrModel,Treat= CR_2012,Line 318 (#Inv=6);95CI GrowthCurves
1550
1216
1333
2001
237
1141
Height
Degree Day
0
10
20
30
40
50
60
70
80
90
0
200
400
600
800
1000
1200

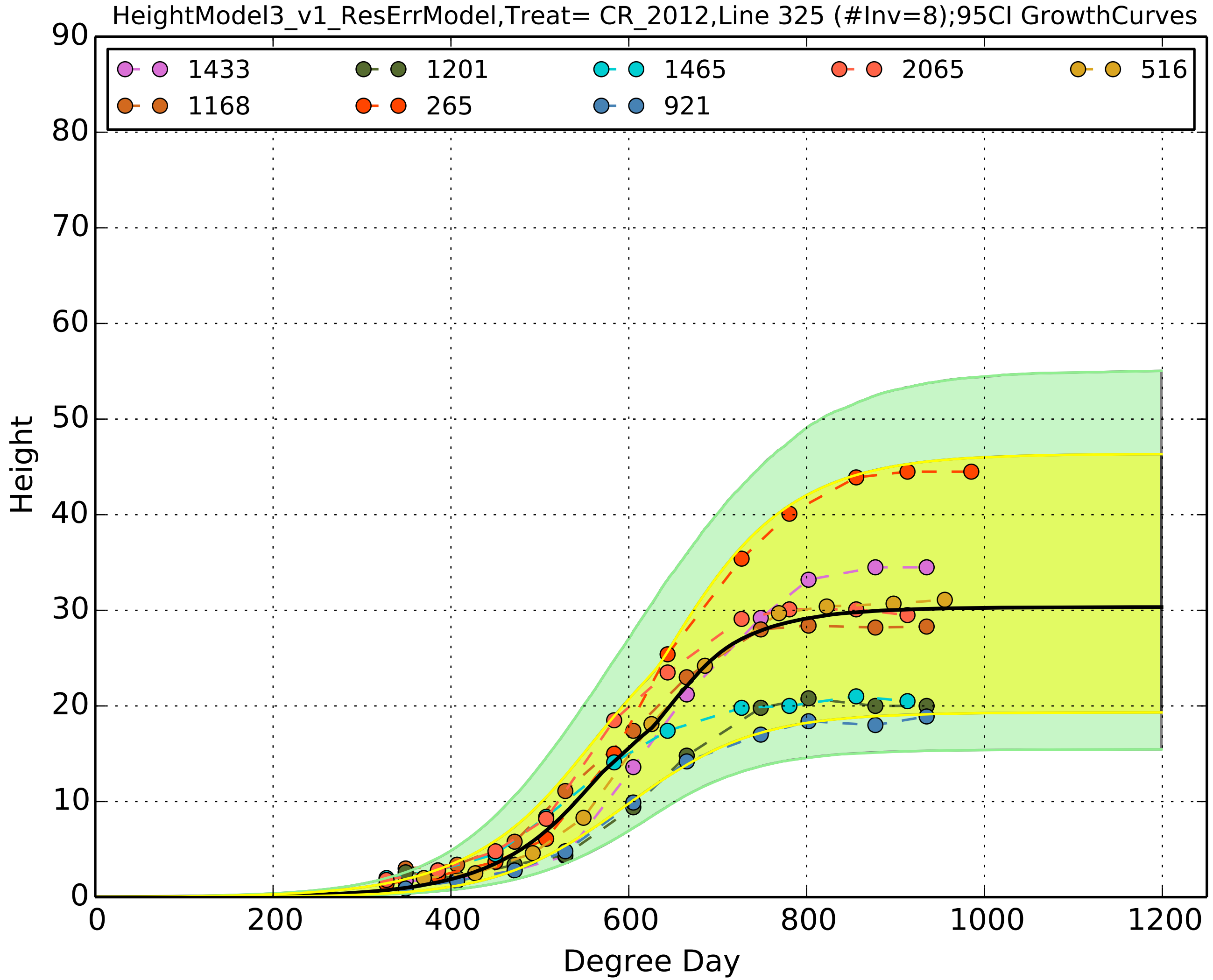

HeightModel3_v1_ResErrModel,Treat= CR_2012,Line 325 (#Inv=8);95CI GrowthCurves
1433
1201
1465
2065
516
1168
265
921
Height
Degree Day
0
10
20
30
40
50
60
70
80
90
0
200
400
600
800
1000
1200

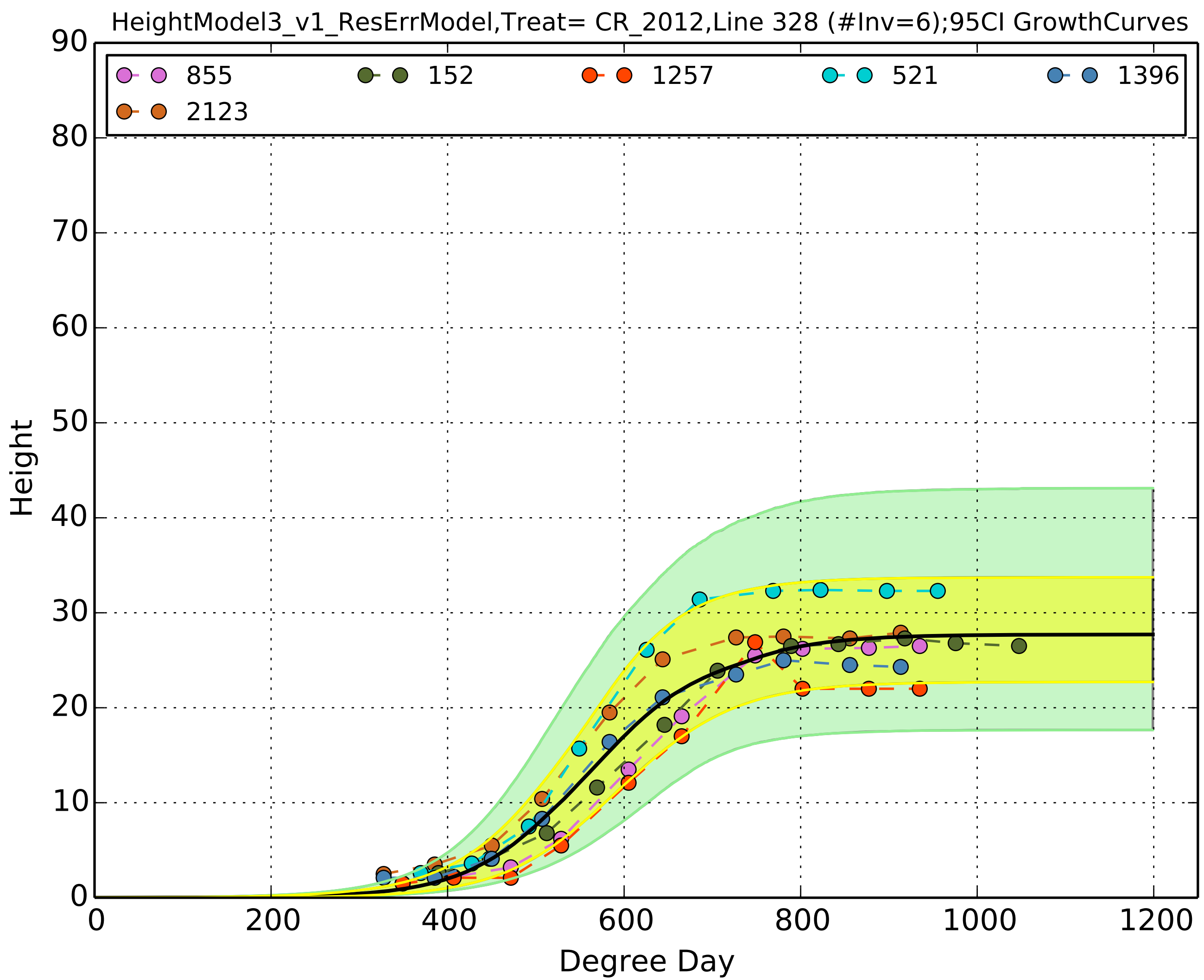

HeightModel3_v1_ResErrModel,Treat= CR_2012,Line 328 (#Inv=6);95CI GrowthCurves
855
152
1257
521
1396
2123
Height
Degree Day
0
10
20
30
40
50
60
70
80
90
0
200
400
600
800
1000
1200

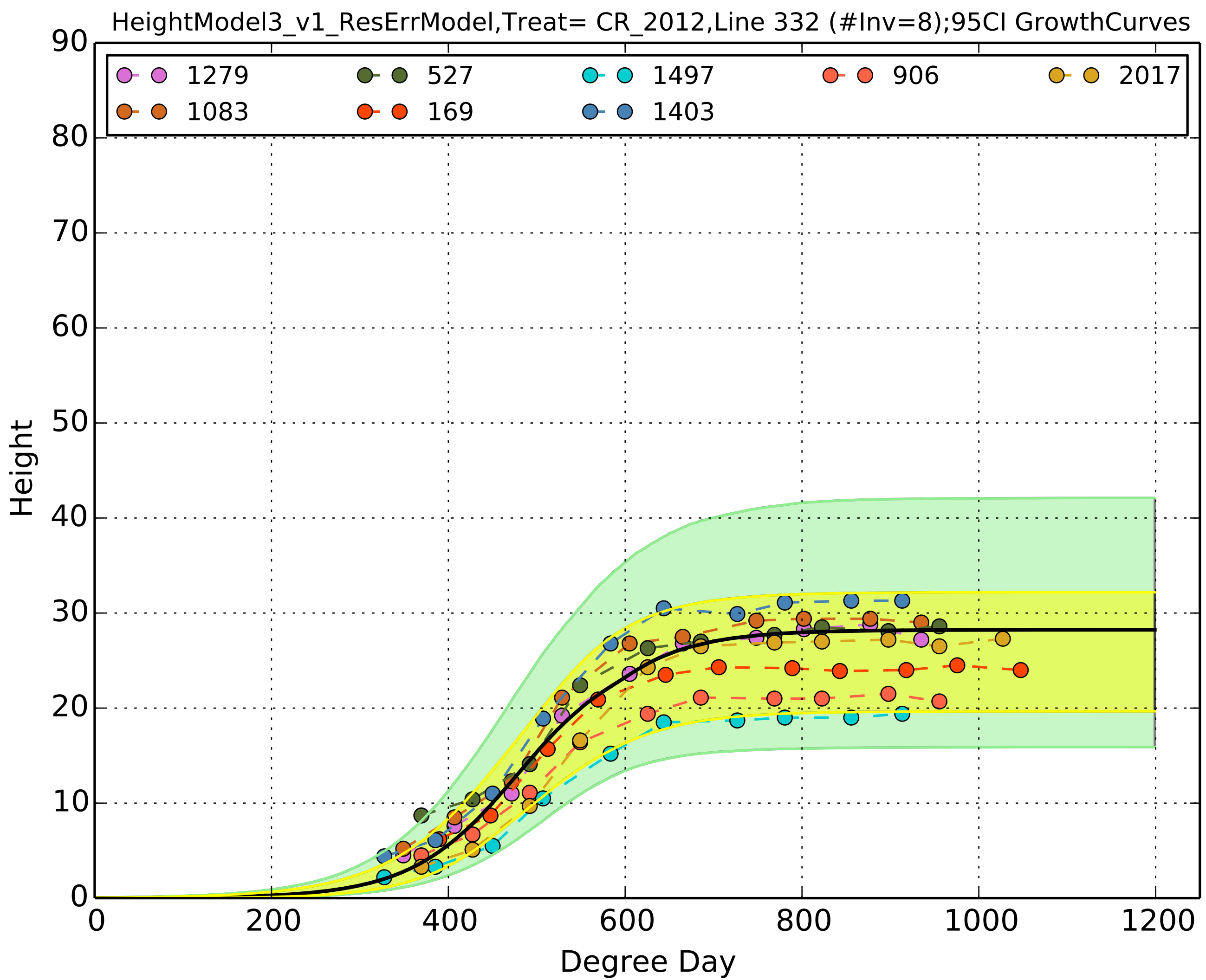
HeightModel3_v1_ResErrModel,Treat= CR_2012,Line 332 (#Inv=8);95CI GrowthCurves
1279
527
1497
906
2017
1083
169
1403
Height
Degree Day
0
10
20
30
40
50
60
70
80
90
0
200
400
600
800
1000
1200

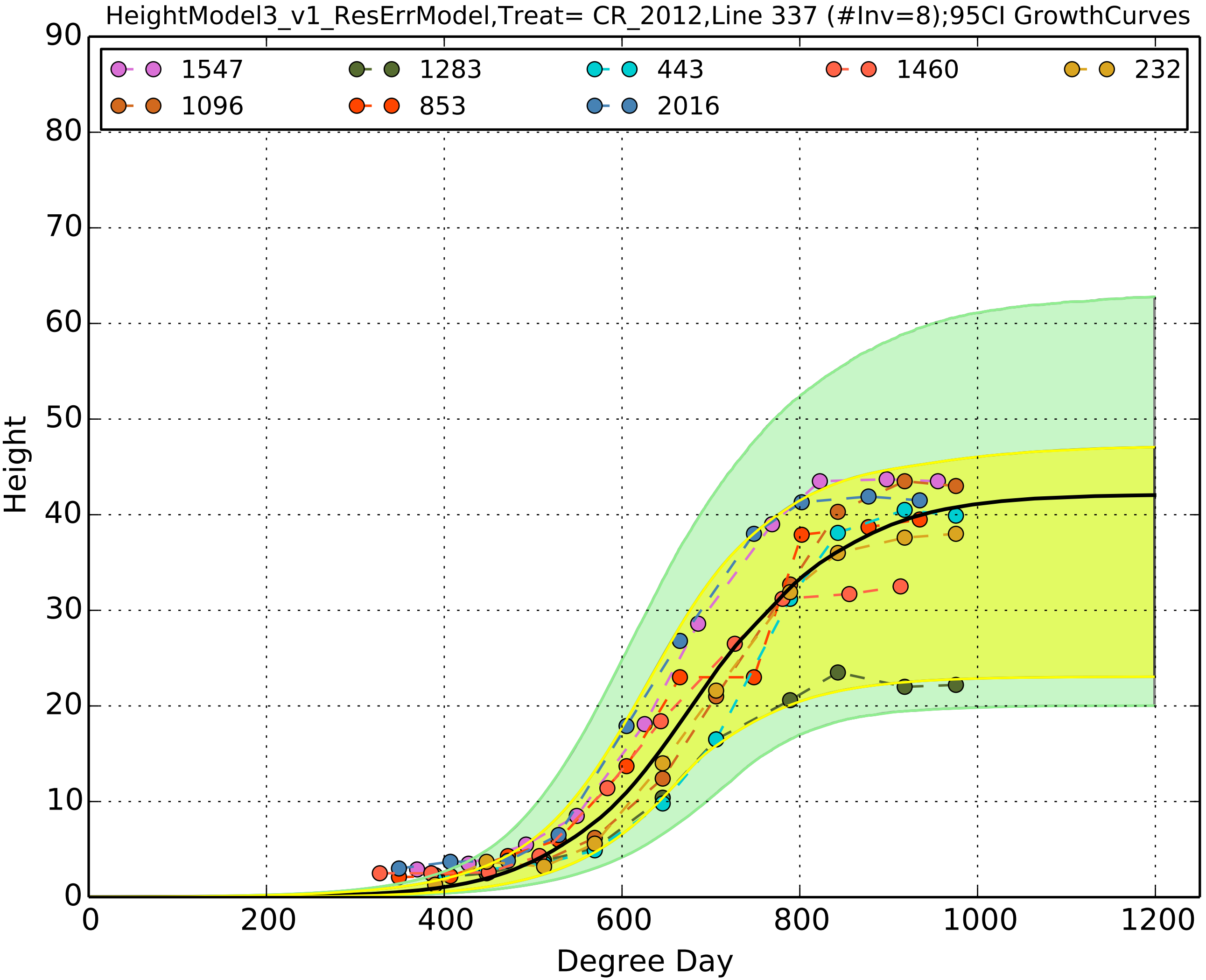
HeightModel3_v1_ResErrModel,Treat= CR_2012,Line 337 (#Inv=8);95CI GrowthCurves
1547
1283
443
1460
232
1096
853
2016
Height
Degree Day
0
10
20
30
40
50
60
70
80
90
0
200
400
600
800
1000
1200

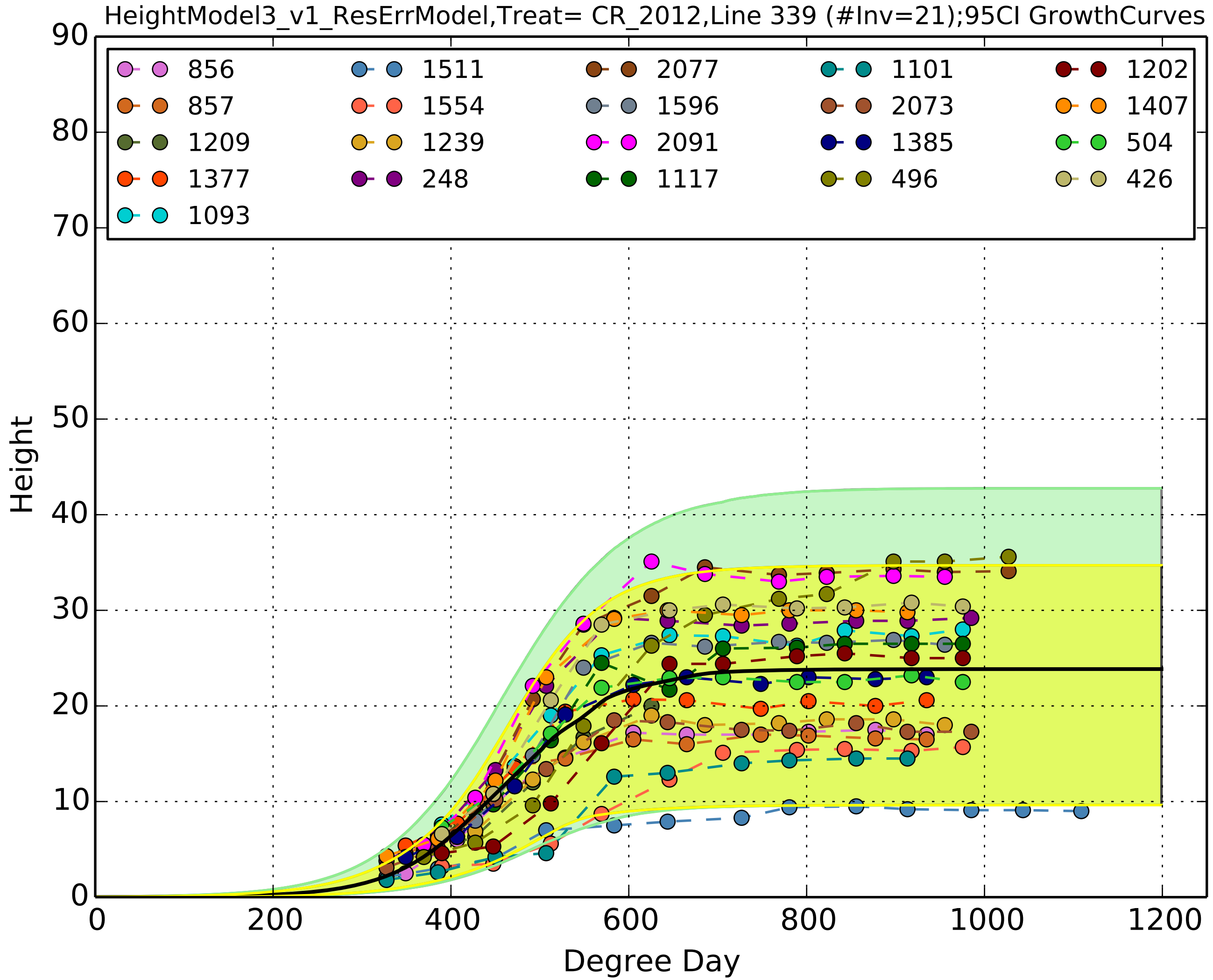
HeightModel3_v1_ResErrModel,Treat= CR_2012,Line 339 (#Inv=21);95CI GrowthCurves
856
857
1209
1377
1093
1511
1554
1239
248
2077
1596
2091
1117
1101
2073
1385
496
1202
1407
504
426
Height
Degree Day
0
10
20
30
40
50
60
70
80
90
0
200
400
600
800
1000
1200

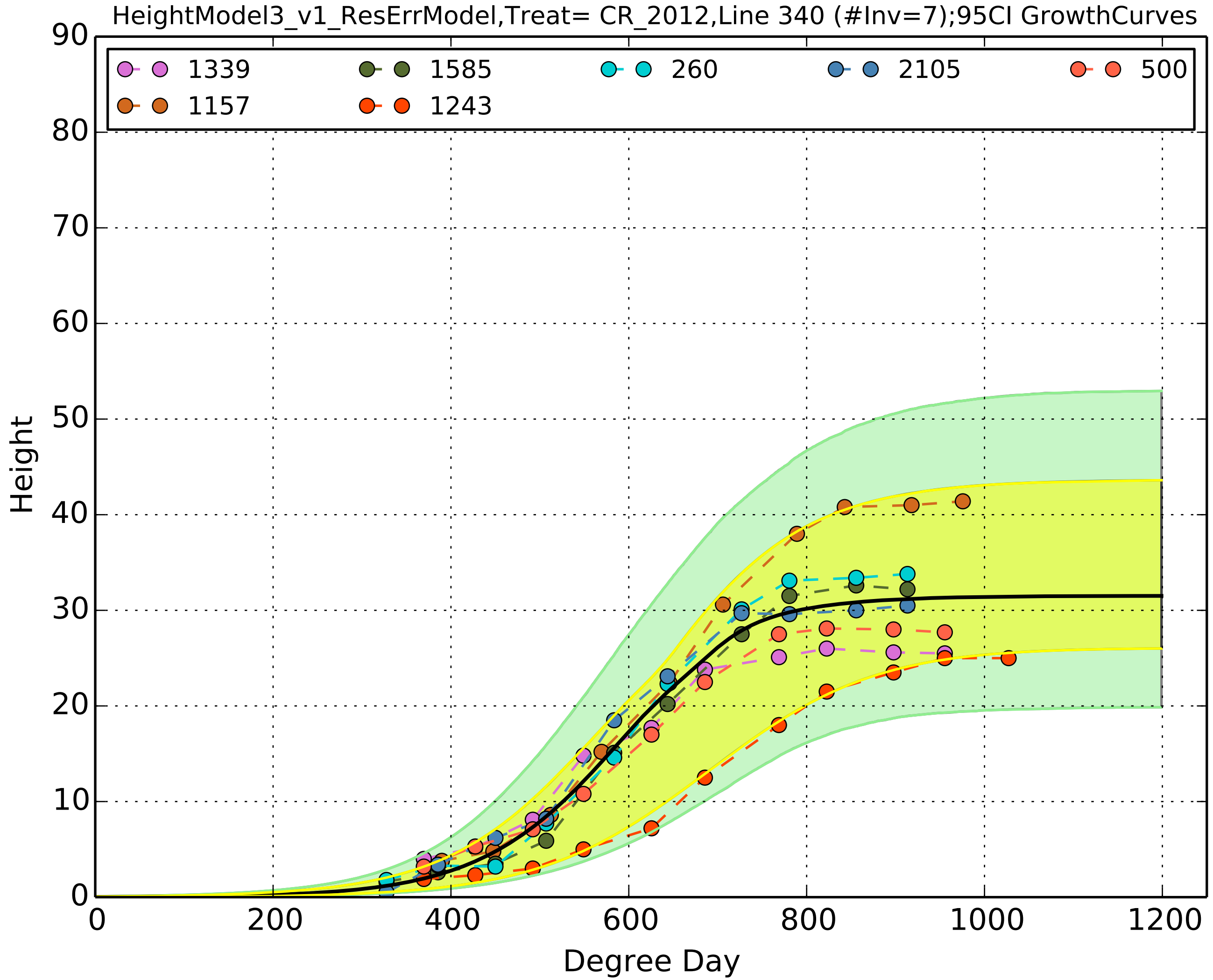
HeightModel3_v1_ResErrModel,Treat= CR_2012,Line 340 (#Inv=7);95CI GrowthCurves
1339
1585
260
2105
500
1157
1243
Height
Degree Day
0
10
20
30
40
50
60
70
80
90
0
200
400
600
800
1000
1200

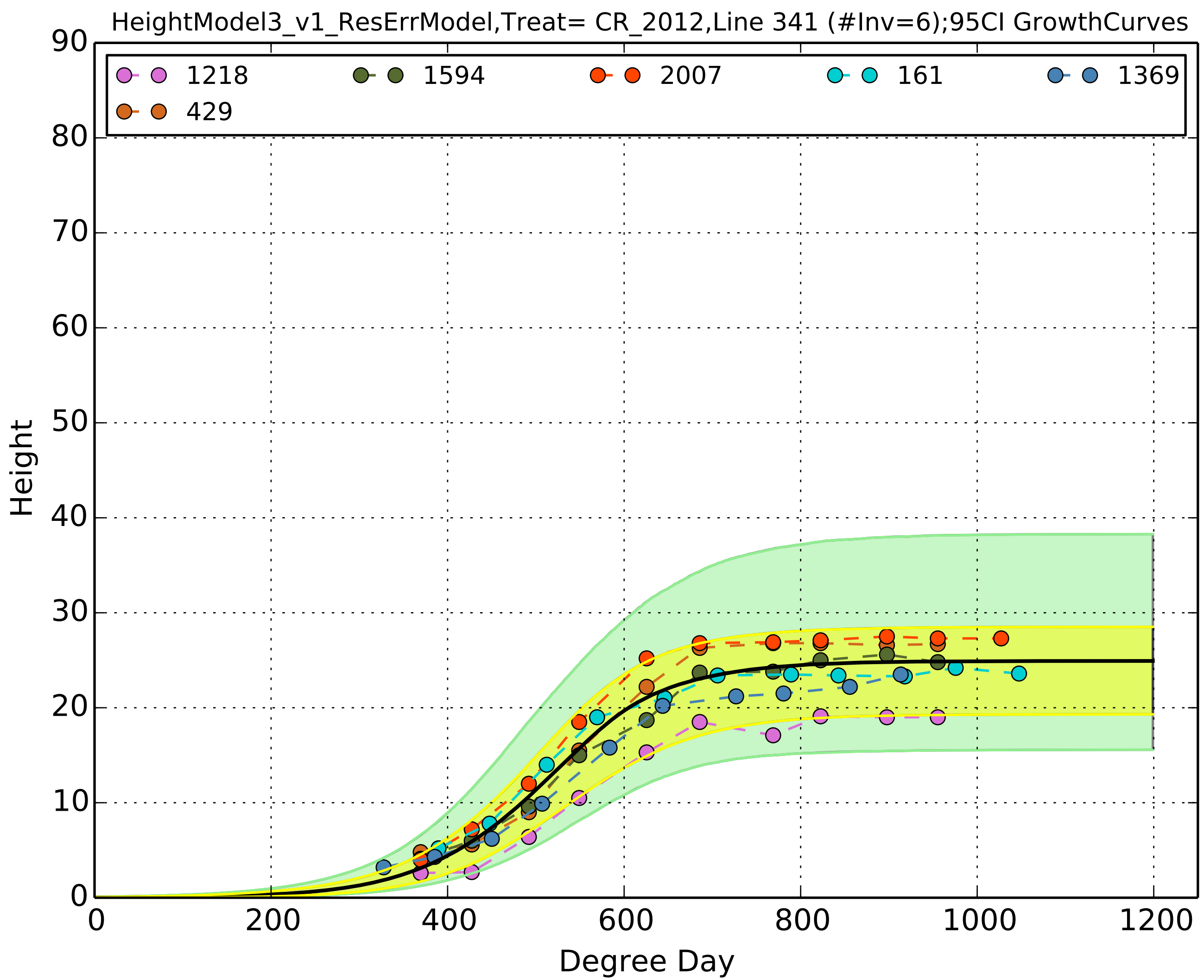

HeightModel3_v1_ResErrModel,Treat= CR_2012,Line 341 (#Inv=6);95CI GrowthCurves
1218
1594
2007
161
1369
429
Height
Degree Day
0
10
20
30
40
50
60
70
80
90
0
200
400
600
800
1000
1200

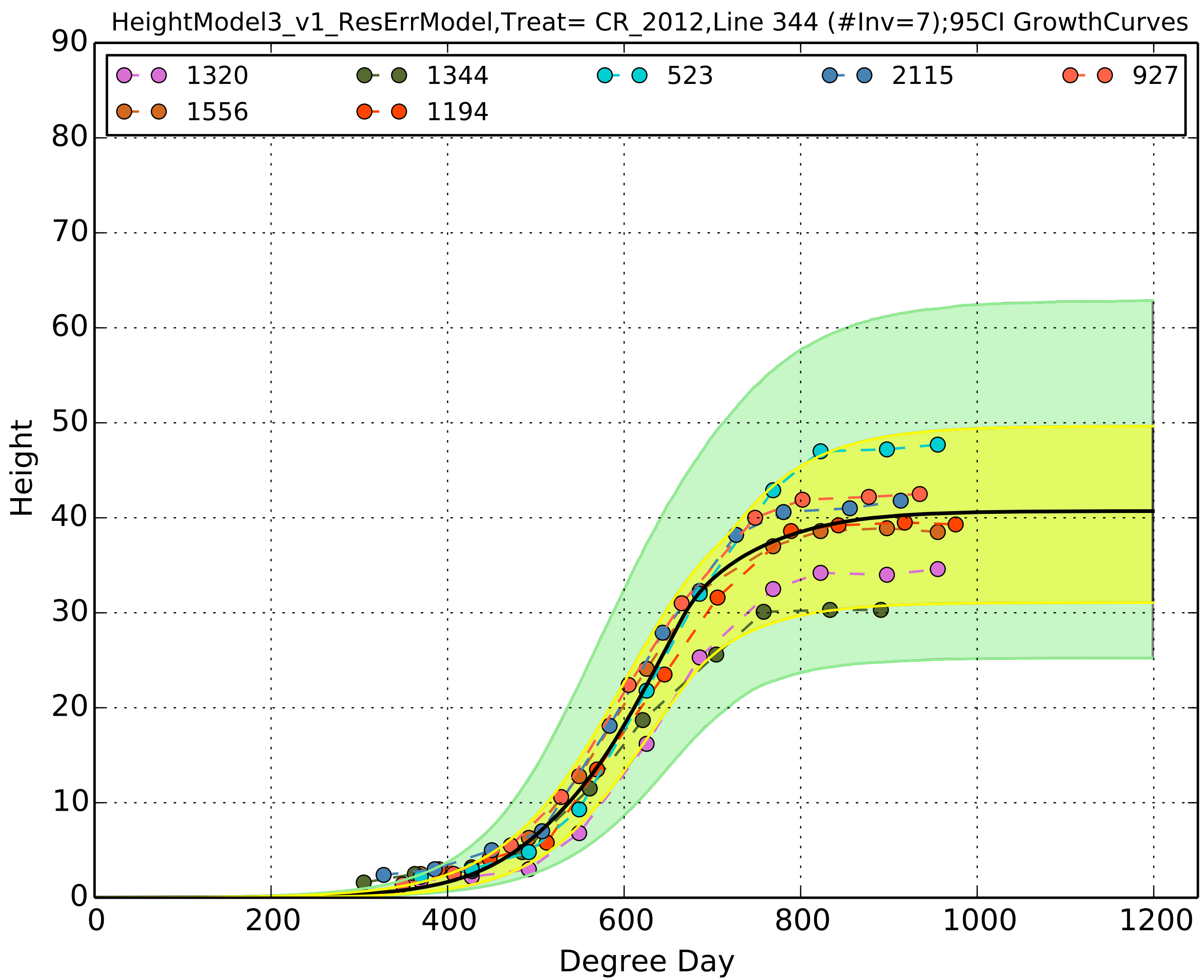

HeightModel3_v1_ResErrModel,Treat= CR_2012,Line 344 (#Inv=7);95CI GrowthCurves
1320
1344
523
2115
927
1556
1194
Height
Degree Day
0
10
20
30
40
50
60
70
80
90
0
200
400
600
800
1000
1200

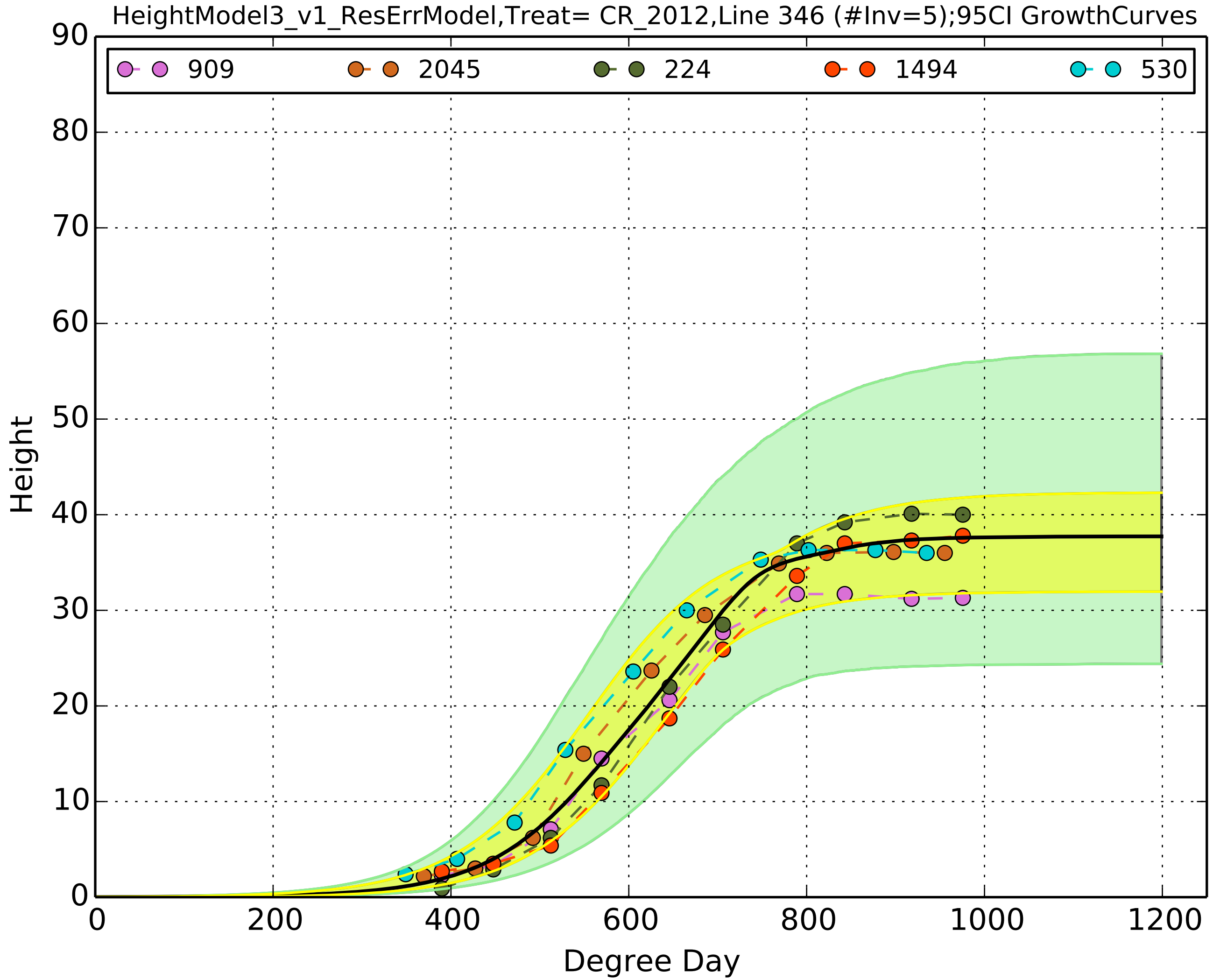

HeightModel3_v1_ResErrModel,Treat= CR_2012,Line 346 (#Inv=5);95CI GrowthCurves
909
2045
224
1494
530
Height
Degree Day
0
10
20
30
40
50
60
70
80
90
0
200
400
600
800
1000
1200

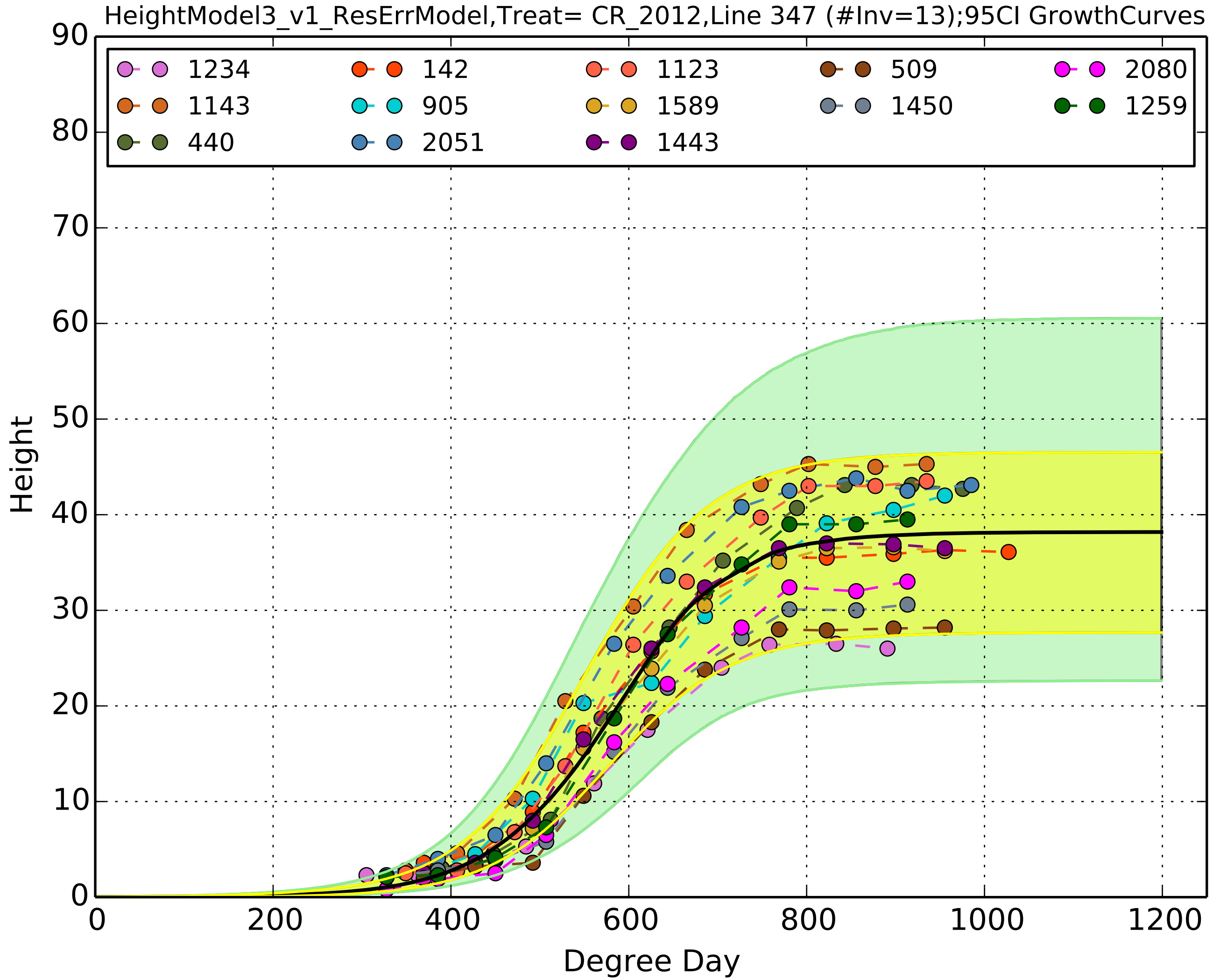
HeightModel3_v1_ResErrModel,Treat= CR_2012,Line 347 (#Inv=13);95CI GrowthCurves
1234
142
1123
509
2080
1143
905
1589
1450
1259
440
2051
1443
Height
Degree Day
0
10
20
30
40
50
60
70
80
90
200
400
600
800
1000
1200

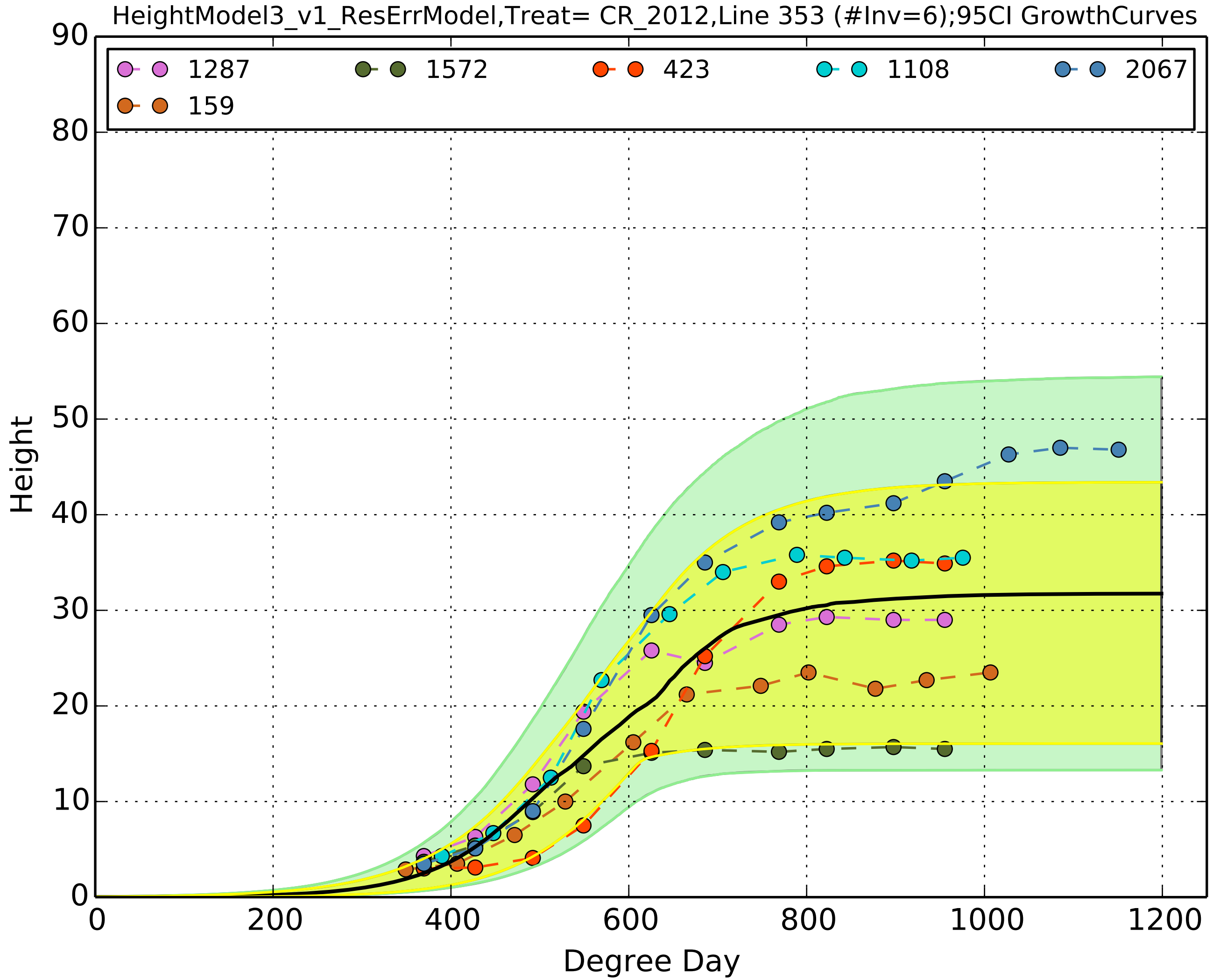
HeightModel3_v1_ResErrModel,Treat= CR_2012,Line 353 (#Inv=6);95CI GrowthCurves
1287
1572
423
1108
2067
159
Height
Degree Day
0
10
20
30
40
50
60
70
80
90
0
200
400
600
800
1000
1200

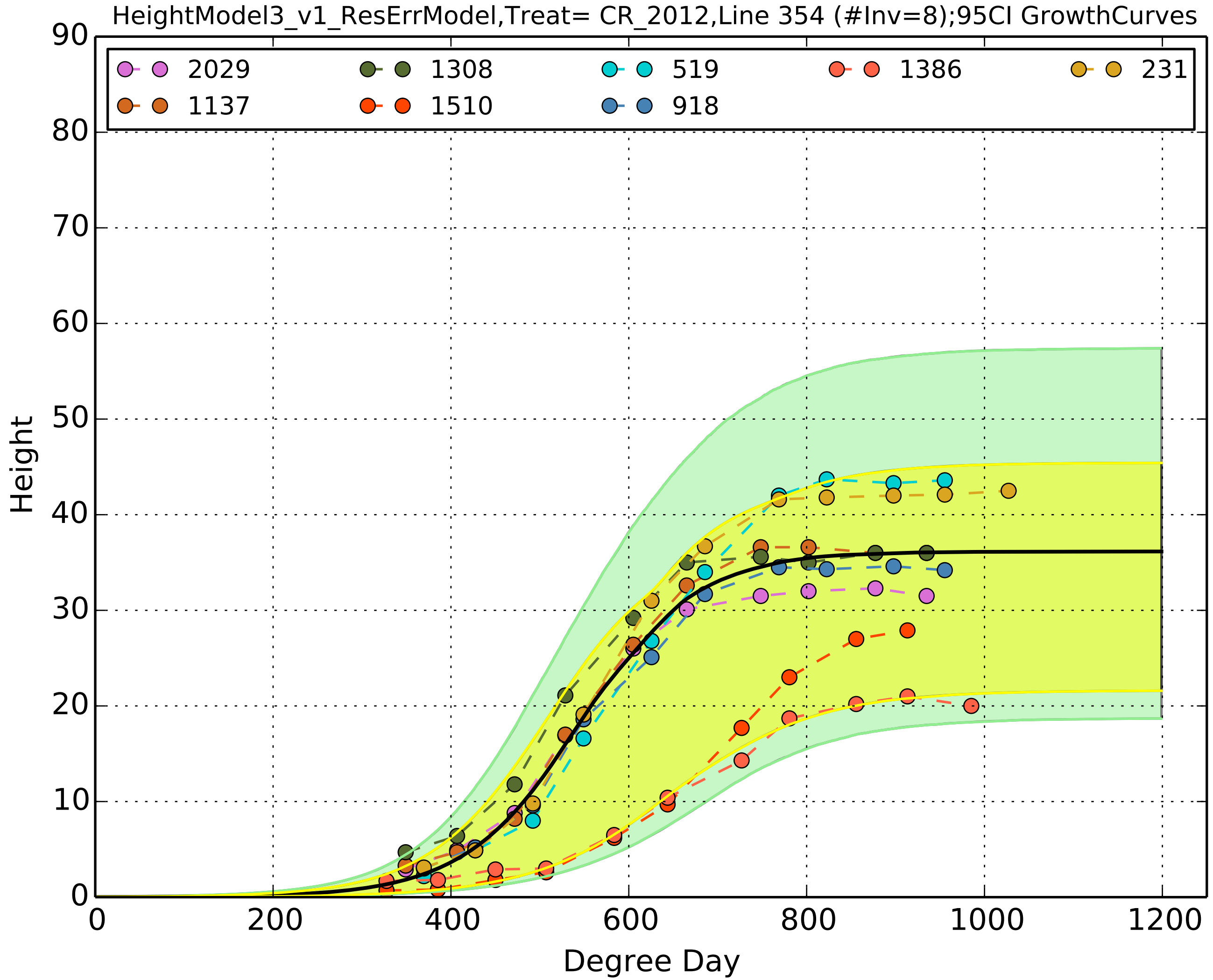

HeightModel3_v1_ResErrModel,Treat= CR_2012,Line 354 (#Inv=8);95CI GrowthCurves
2029
1308
519
1386
231
1137
1510
918
Height
Degree Day
0
10
20
30
40
50
60
70
80
90
0
200
400
600
800
1000
1200

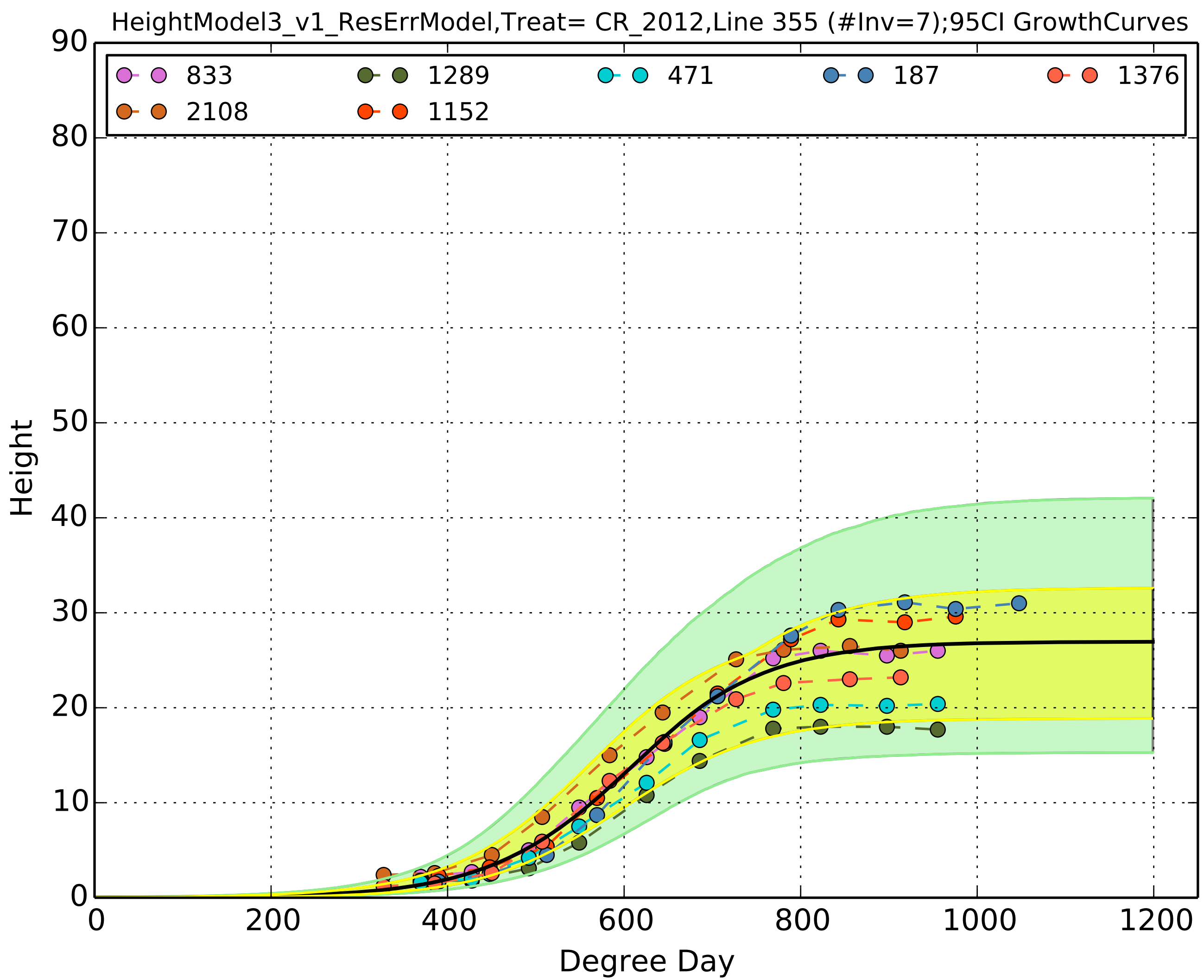

HeightModel3_v1_ResErrModel,Treat= CR_2012,Line 355 (#Inv=7);95CI GrowthCurves
833
1289
471
187
1376
2108
1152
Height
Degree Day
0
10
20
30
40
50
60
70
80
90
0
200
400
600
800
1000
1200

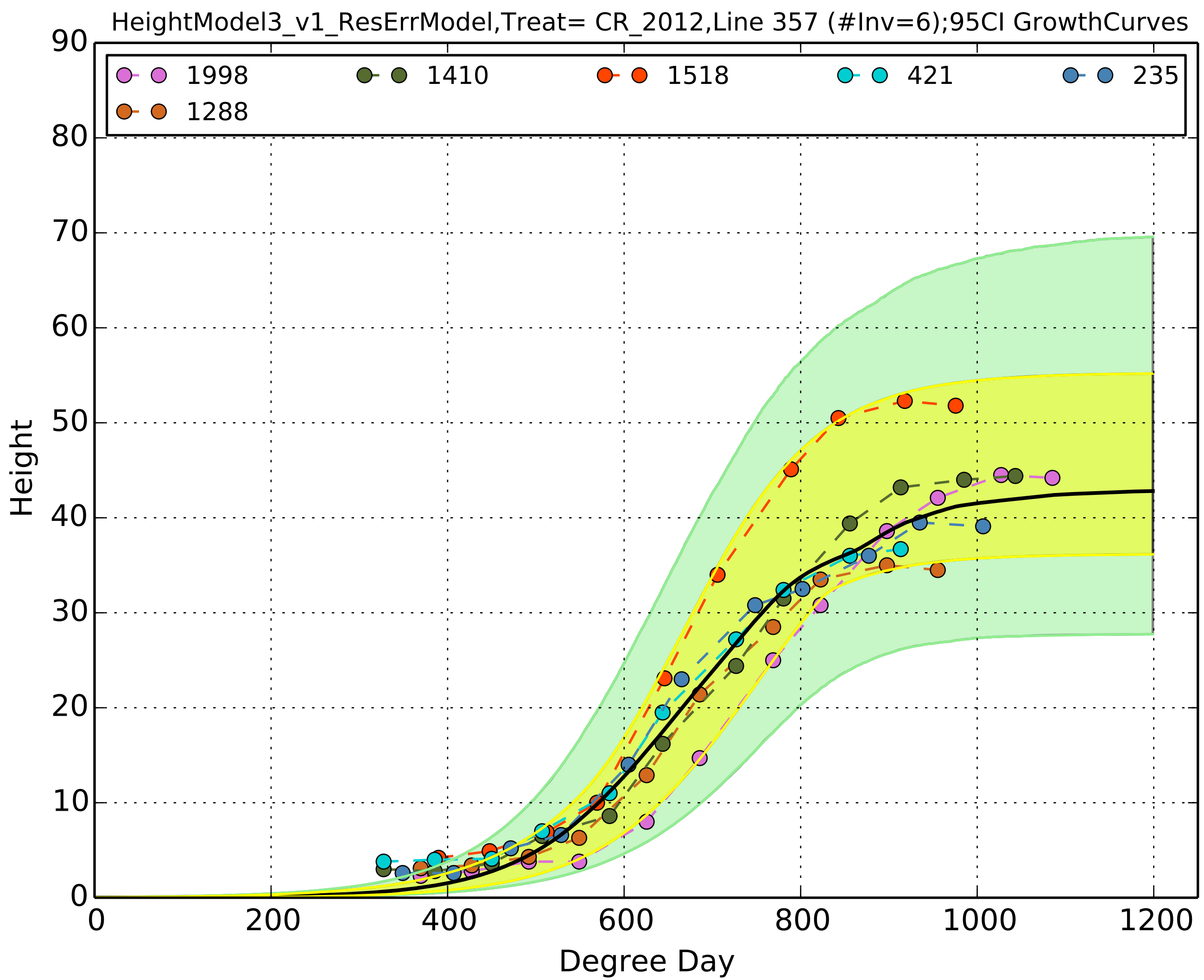

HeightModel3_v1_ResErrModel,Treat= CR_2012,Line 357 (#Inv=6);95CI GrowthCurves
1998
1410
1518
421
235
1288
Height
Degree Day
0
10
20
30
40
50
60
70
80
90
0
200
400
600
800
1000
1200

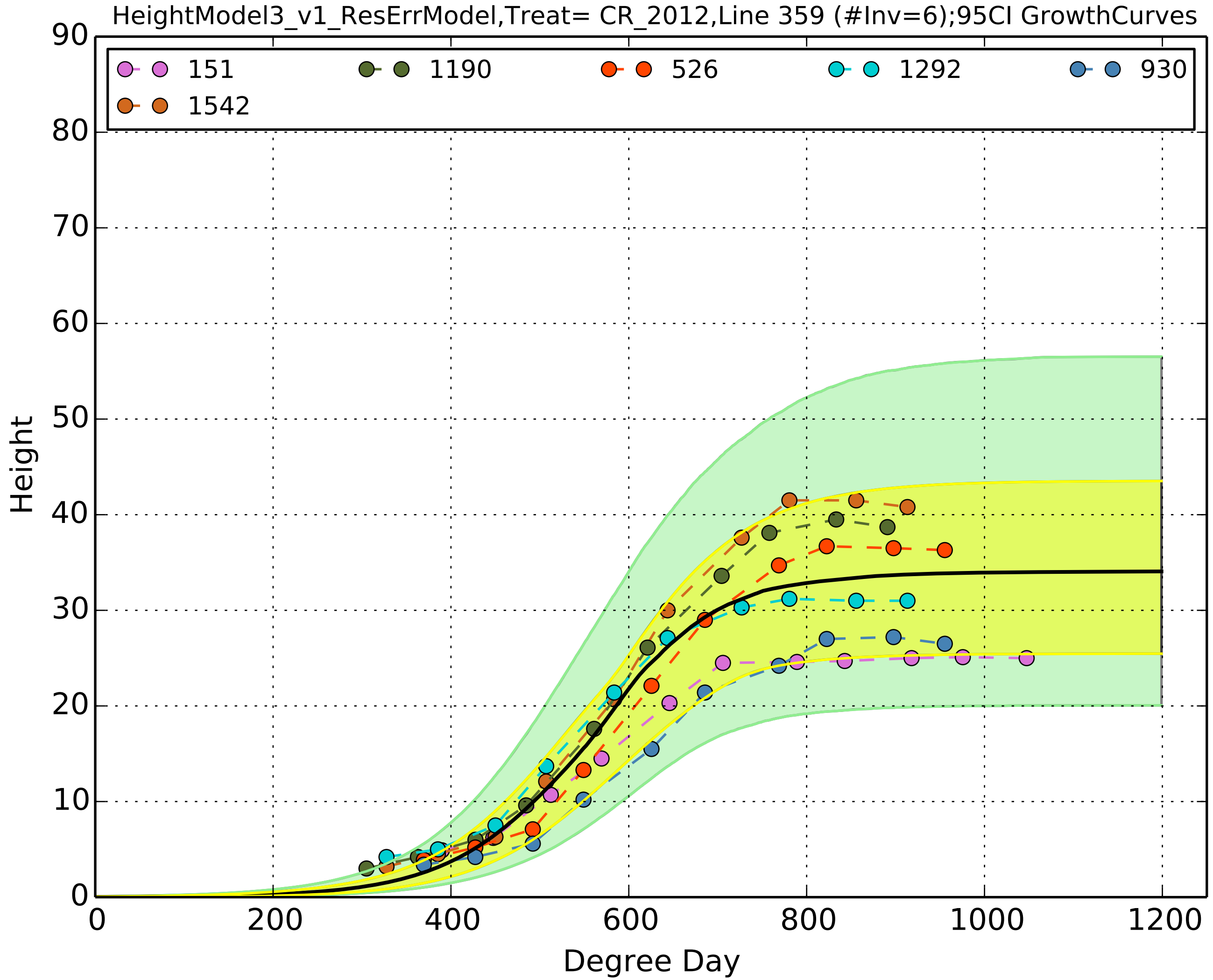

HeightModel3_v1_ResErrModel,Treat= CR_2012,Line 359 (#Inv=6);95CI GrowthCurves
151
1190
526
1292
930
1542
Height
Degree Day
0
10
20
30
40
50
60
70
80
90
0
200
400
600
800
1000
1200

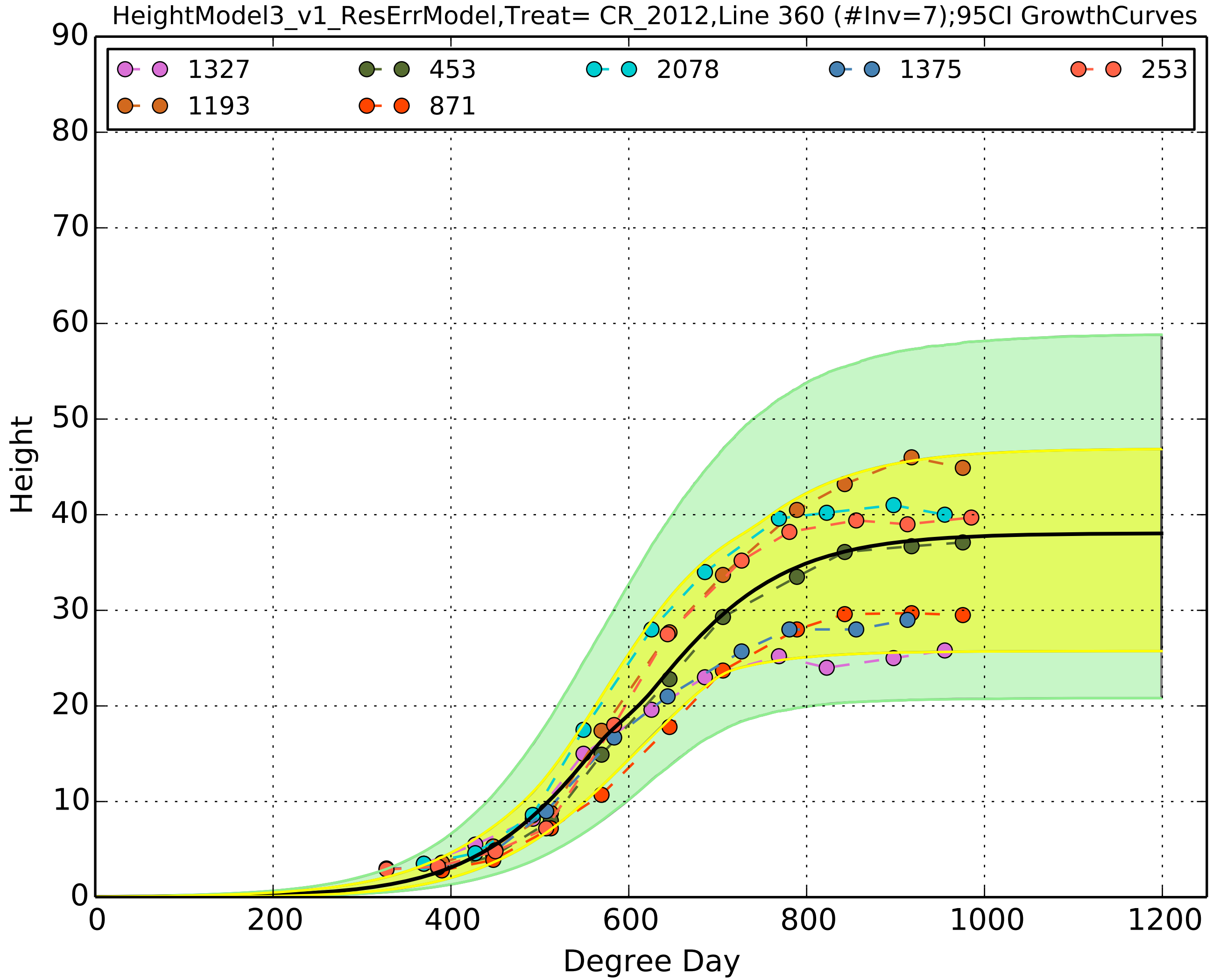

HeightModel3_v1_ResErrModel,Treat= CR_2012,Line 360 (#Inv=7);95CI GrowthCurves
1327
453
2078
1375
253
1193
871
Height
Degree Day
0
10
20
30
40
50
60
70
80
90
0
200
400
600
800
1000
1200

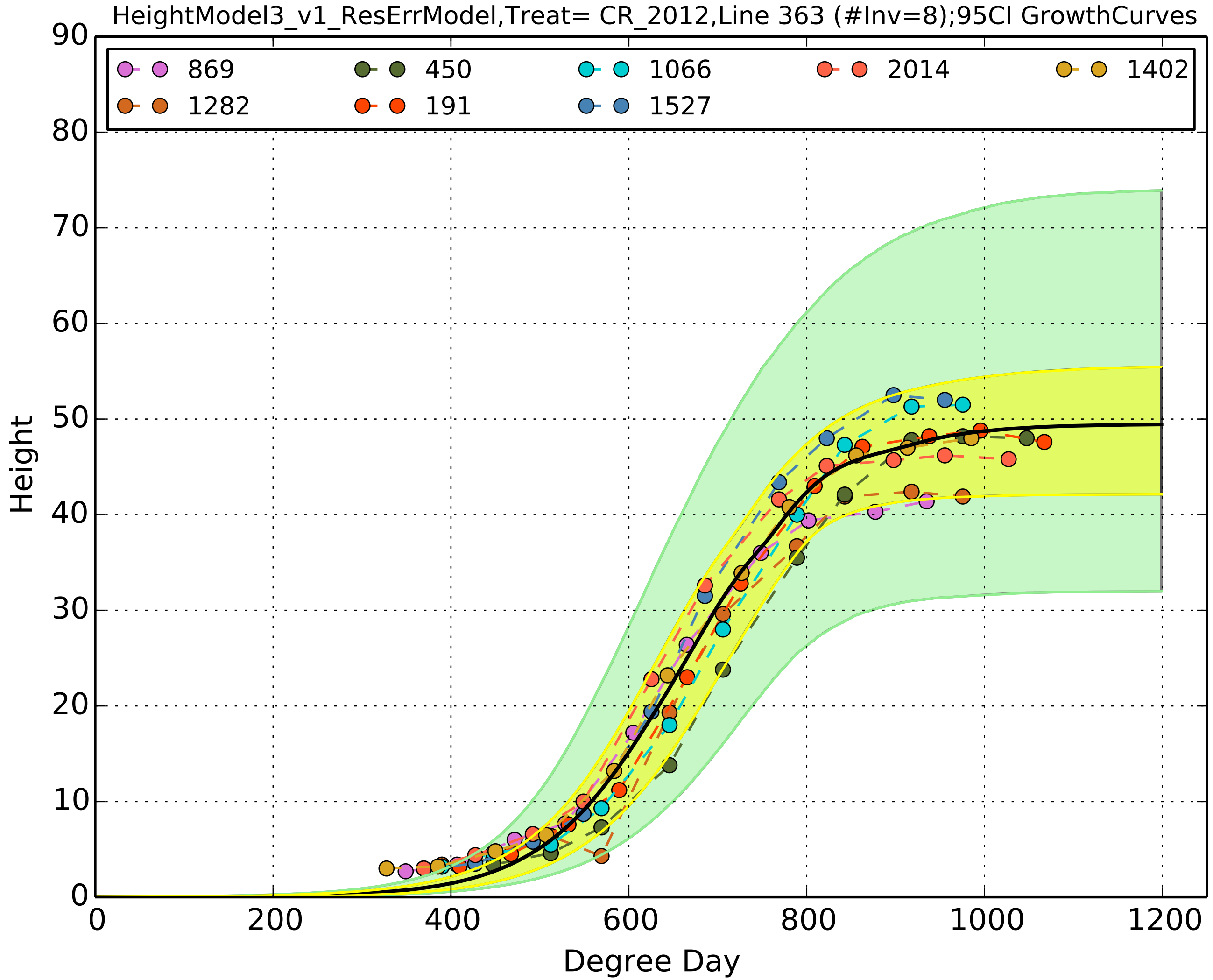

HeightModel3_v1_ResErrModel,Treat= CR_2012,Line 363 (#Inv=8);95CI GrowthCurves
869
450
1066
2014
1402
1282
191
1527
Height
Degree Day
0
10
20
30
40
50
60
70
80
90
0
200
400
600
800
1000
1200

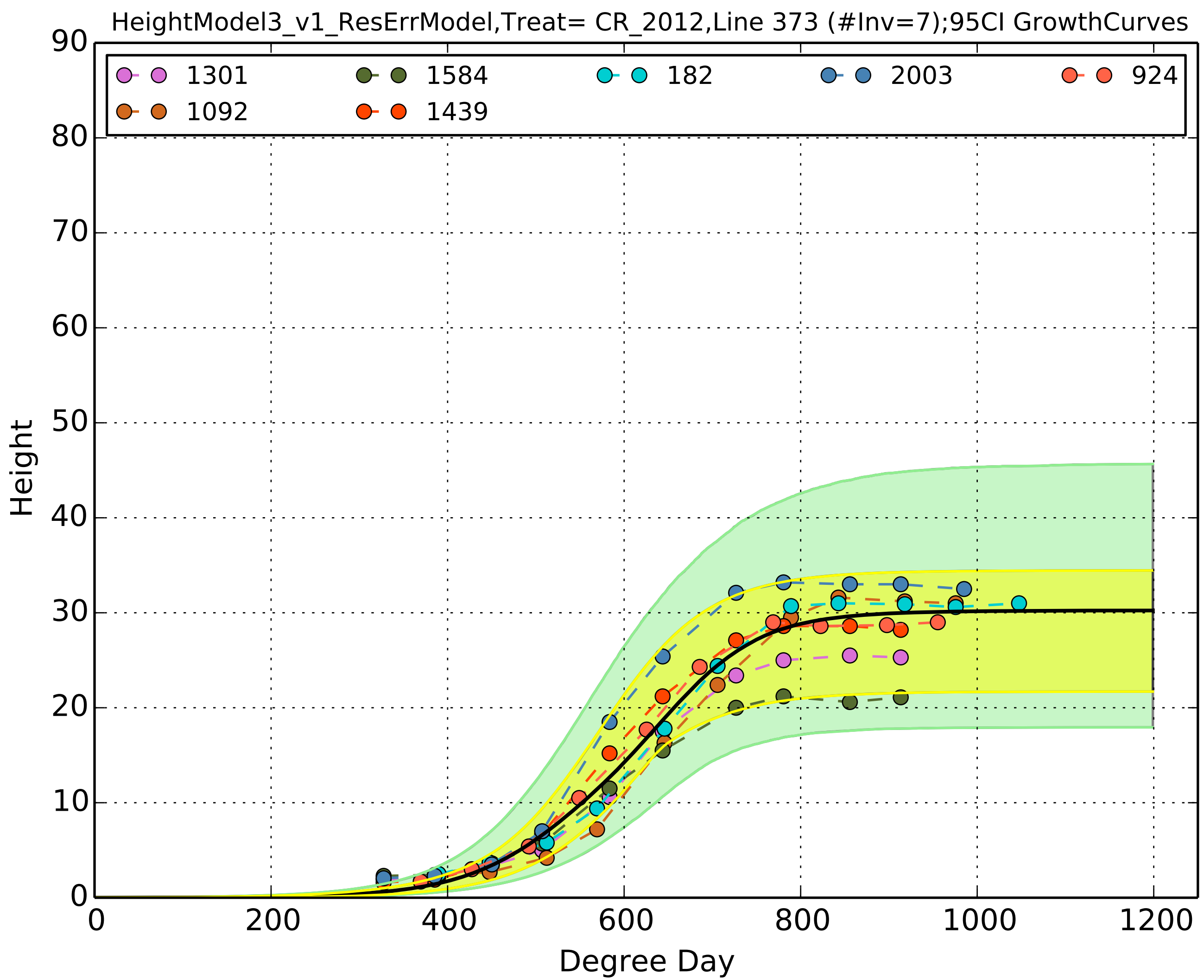
HeightModel3_v1_ResErrModel,Treat= CR_2012,Line 373 (#Inv=7);95CI GrowthCurves
1301
1584
182
2003
924
1092
1439
Height
Degree Day
0
10
20
30
40
50
60
70
80
90
0
200
400
600
800
1000
1200

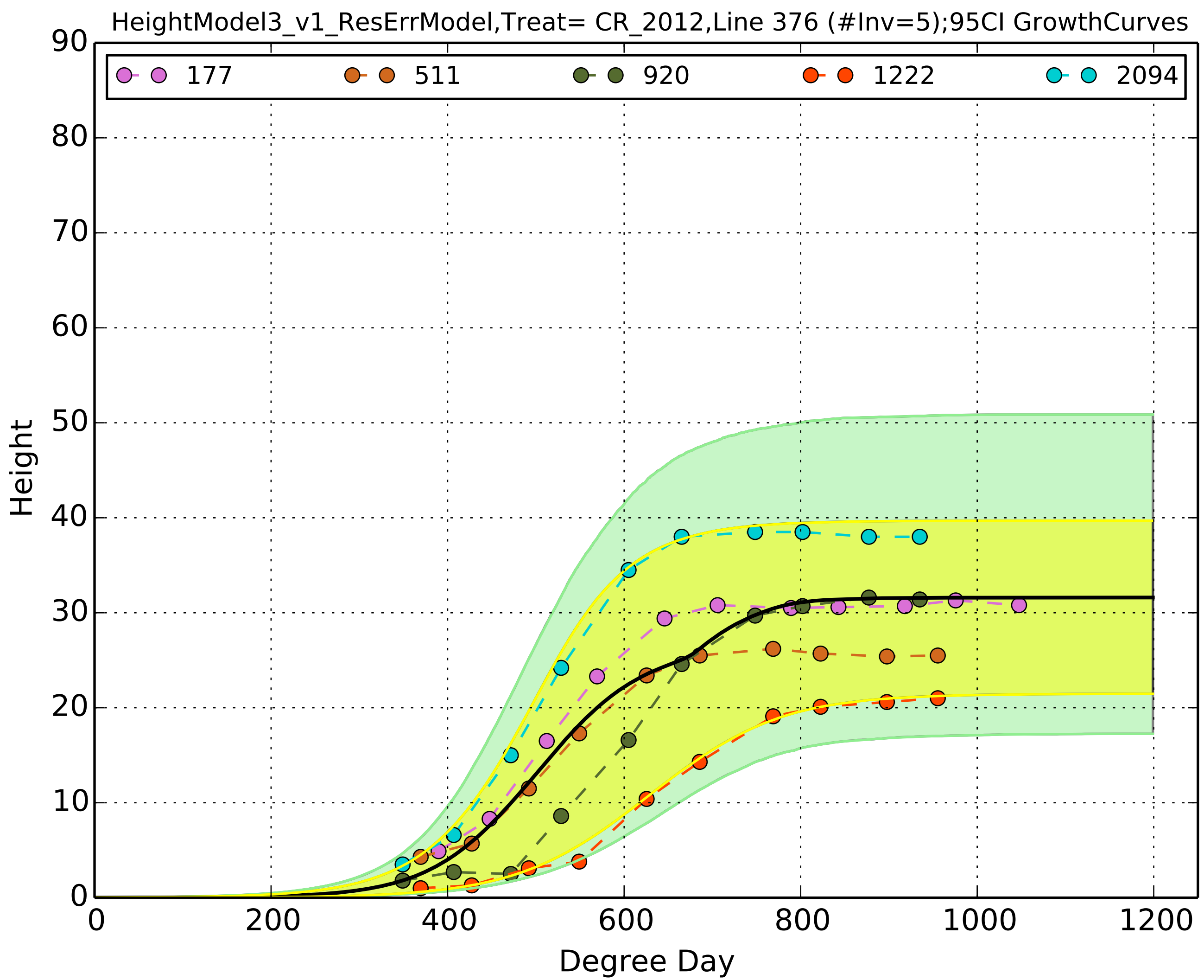
HeightModel3_v1_ResErrModel,Treat= CR_2012,Line 376 (#Inv=5);95CI GrowthCurves
177
511
920
1222
2094
Height
Degree Day
0
10
20
30
40
50
60
70
80
90
0
200
400
600
800
1000
1200

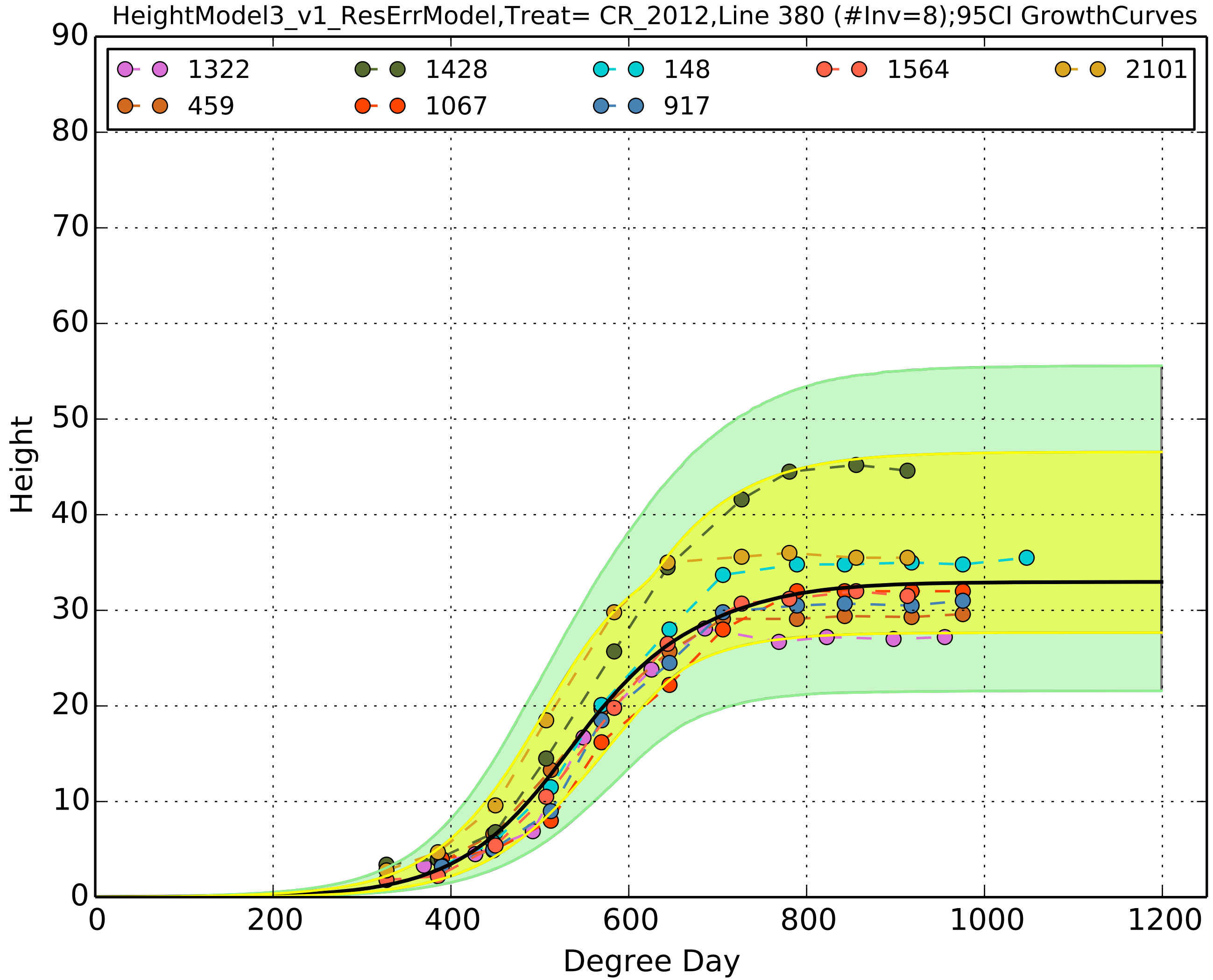
HeightModel3_v1_ResErrModel,Treat= CR_2012,Line 380 (#Inv=8);95CI GrowthCurves
1322
1428
148
1564
2101
459
1067
917
Height
Degree Day
0
10
20
30
40
50
60
70
80
90
0
200
400
600
800
1000
1200

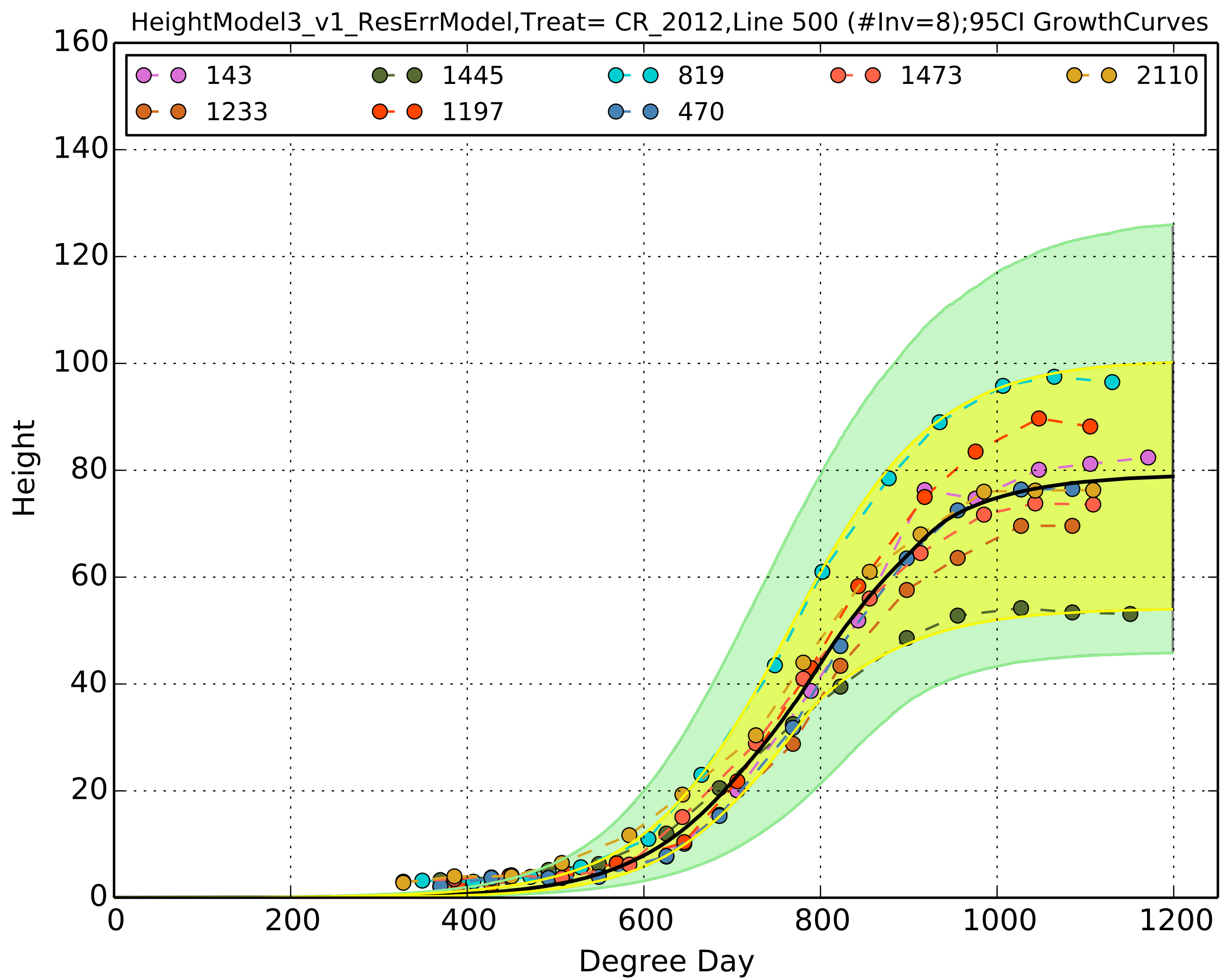

HeightModel3_v1_ResErrModel,Treat= CR_2012,Line 500 (#Inv=8);95CI GrowthCurves
143
1445
819
1473
2110
1233
1197
470
Height
Degree Day
0
20
40
60
80
100
120
140
160
0
200
400
600
800
1000
1200

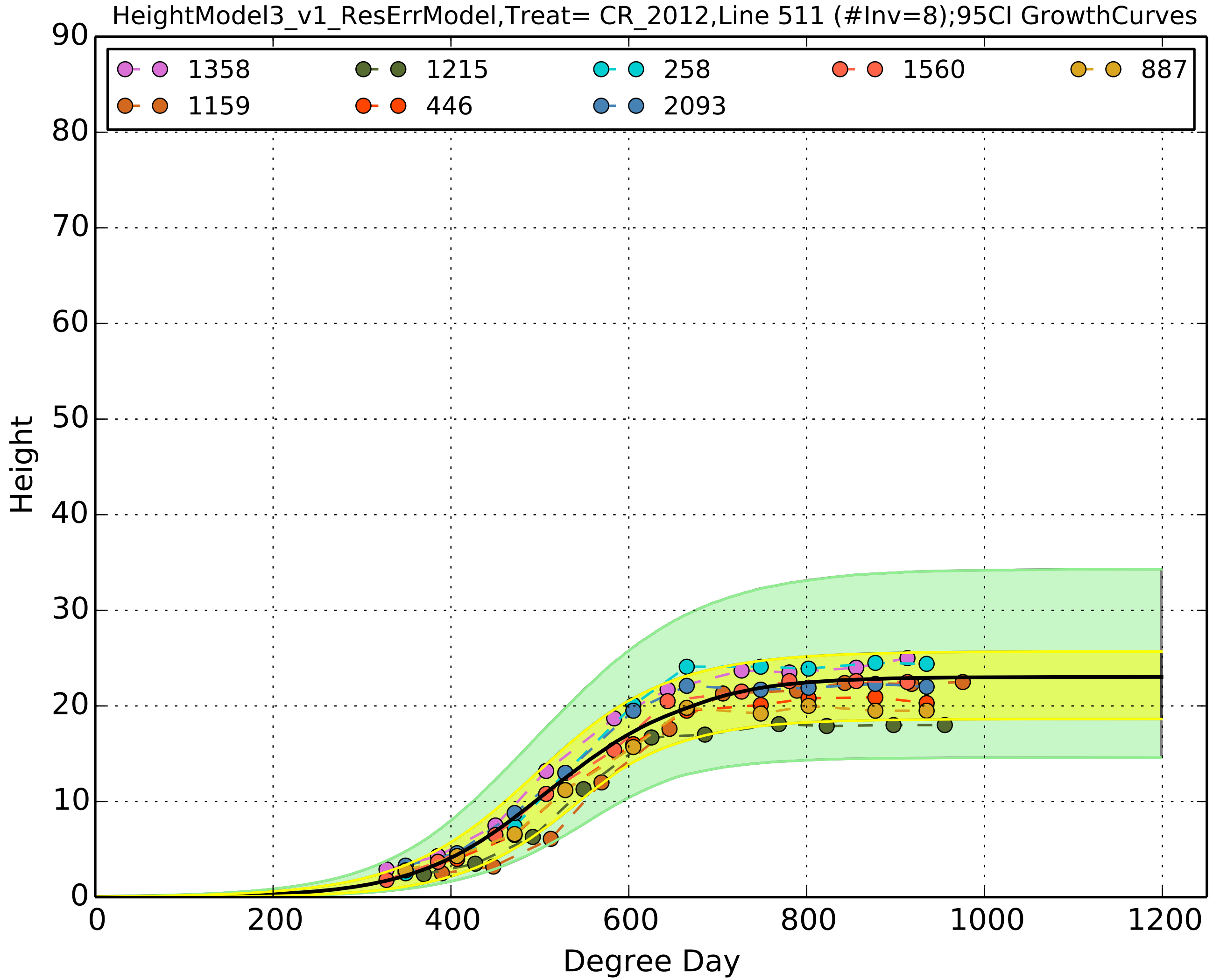

HeightModel3_v1_ResErrModel,Treat= CR_2012,Line 511 (#Inv=8);95CI GrowthCurves
1358
1215
258
1560
887
1159
446
2093
Height
Degree Day
0
10
20
30
40
50
60
70
80
90
0
200
400
600
800
1000
1200
