# Supplementary figures and images for: Integrating transcriptomic network reconstruction and eQTL analyses reveals mechanistic connections between genomic architecture and Brassica rapa development
Source: PLoS Genet. 2019 Sep 12;15(9):e1008367. doi: 10.1371/journal.pgen.1008367 (PMC6759183; doi:10.1371/journal.pgen.1008367)

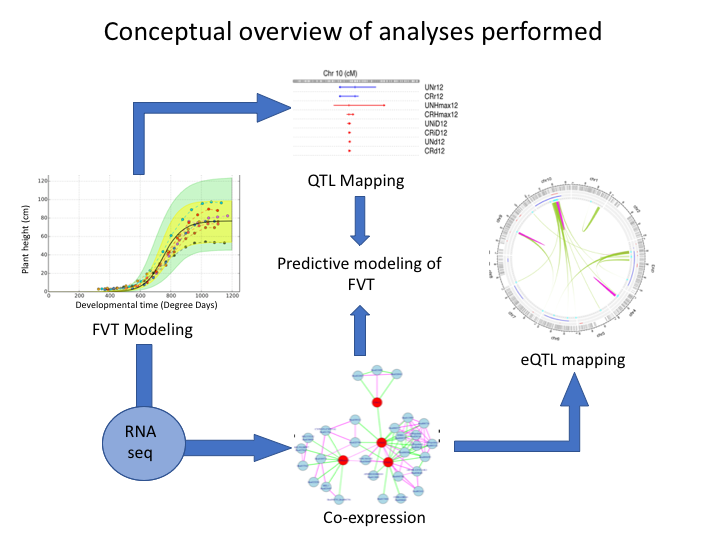

Supplement: S2 Fig — (PNG) [file pgen.1008367.s002.png]

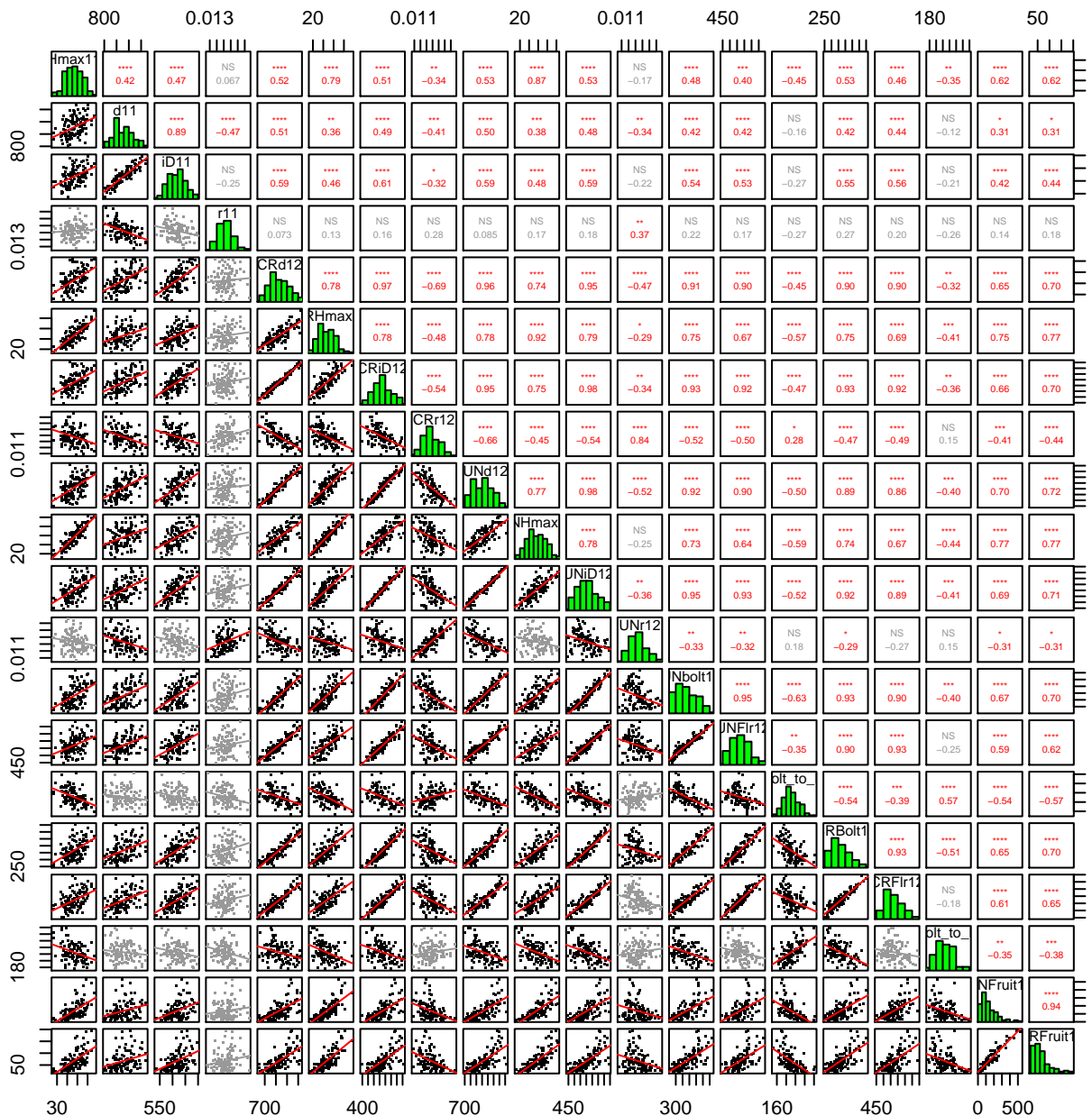

Supplement: S3 Fig — (PDF) [file pgen.1008367.s003.pdf]

MR cutoff = 10

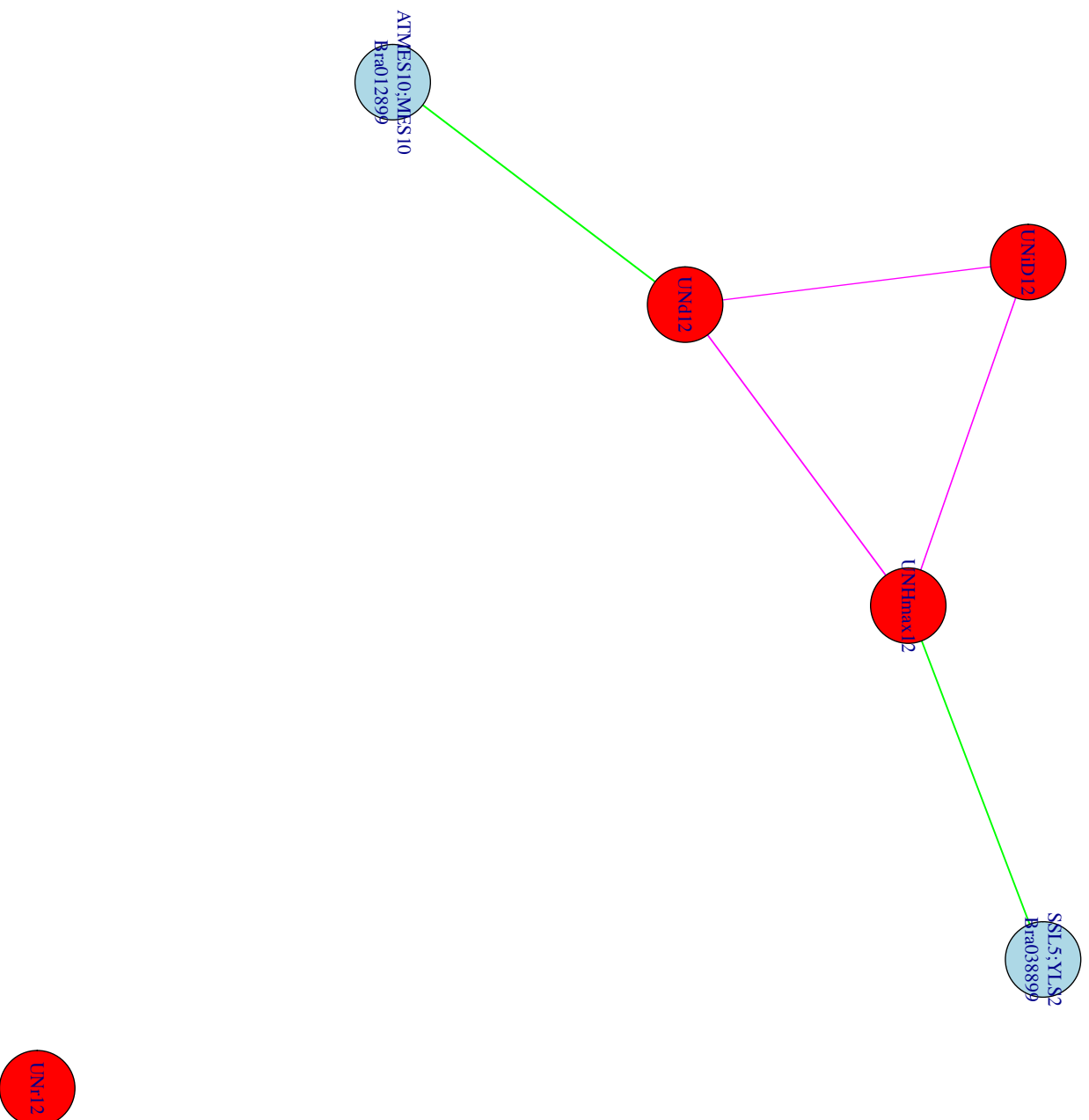

MR cutoff = 20

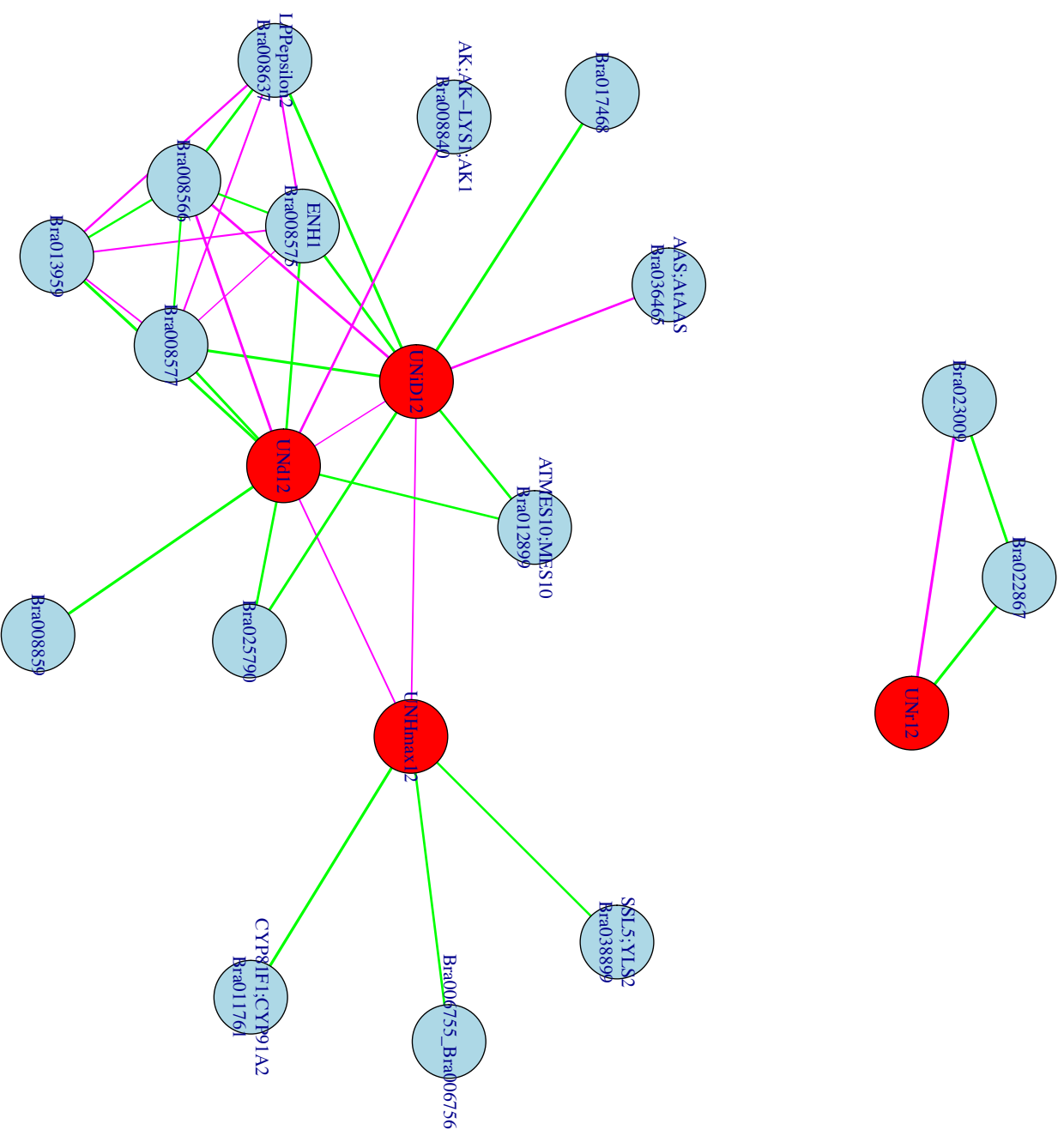

**MR cutoff = 30**

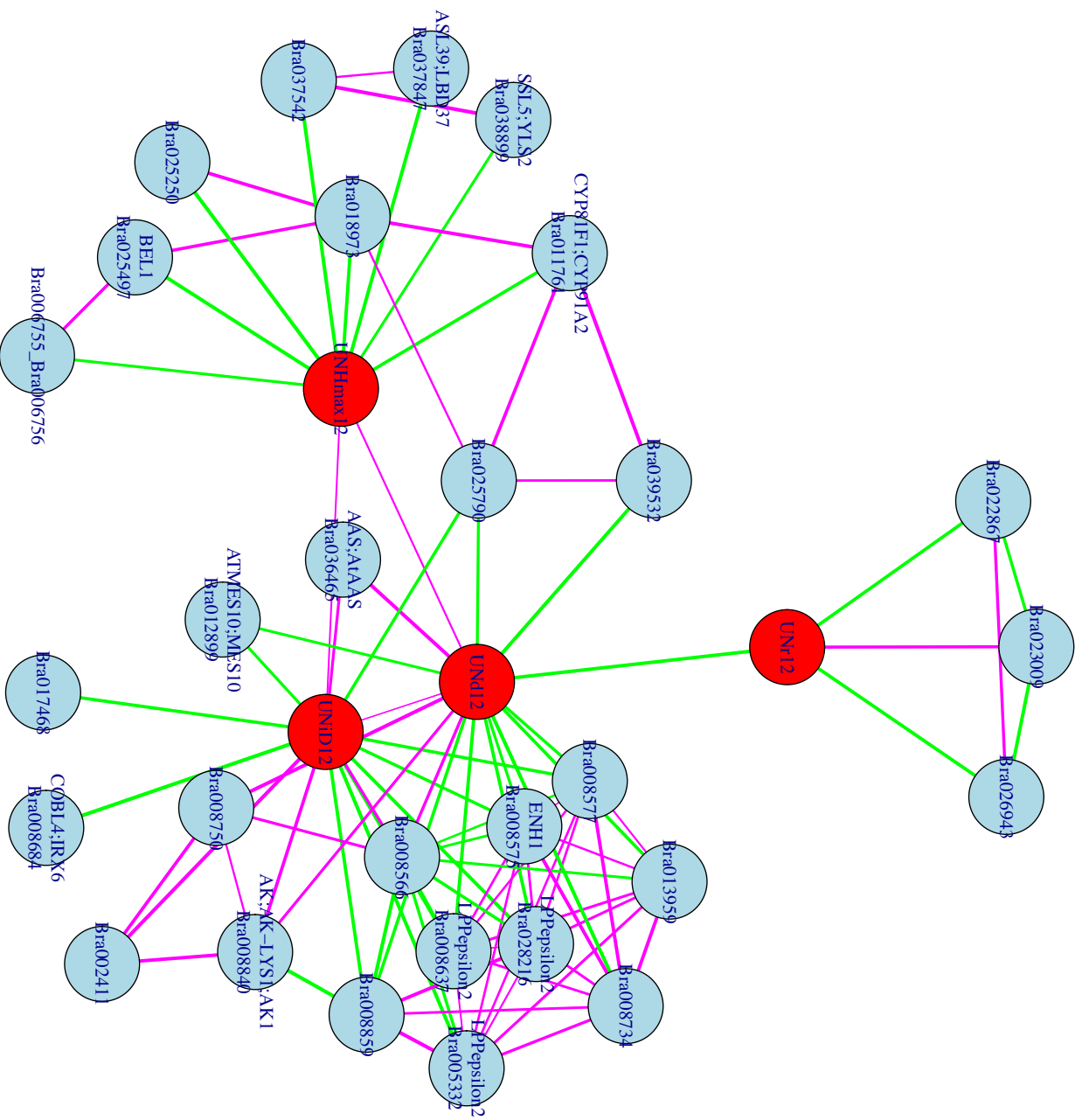

**MR cutoff = 50**

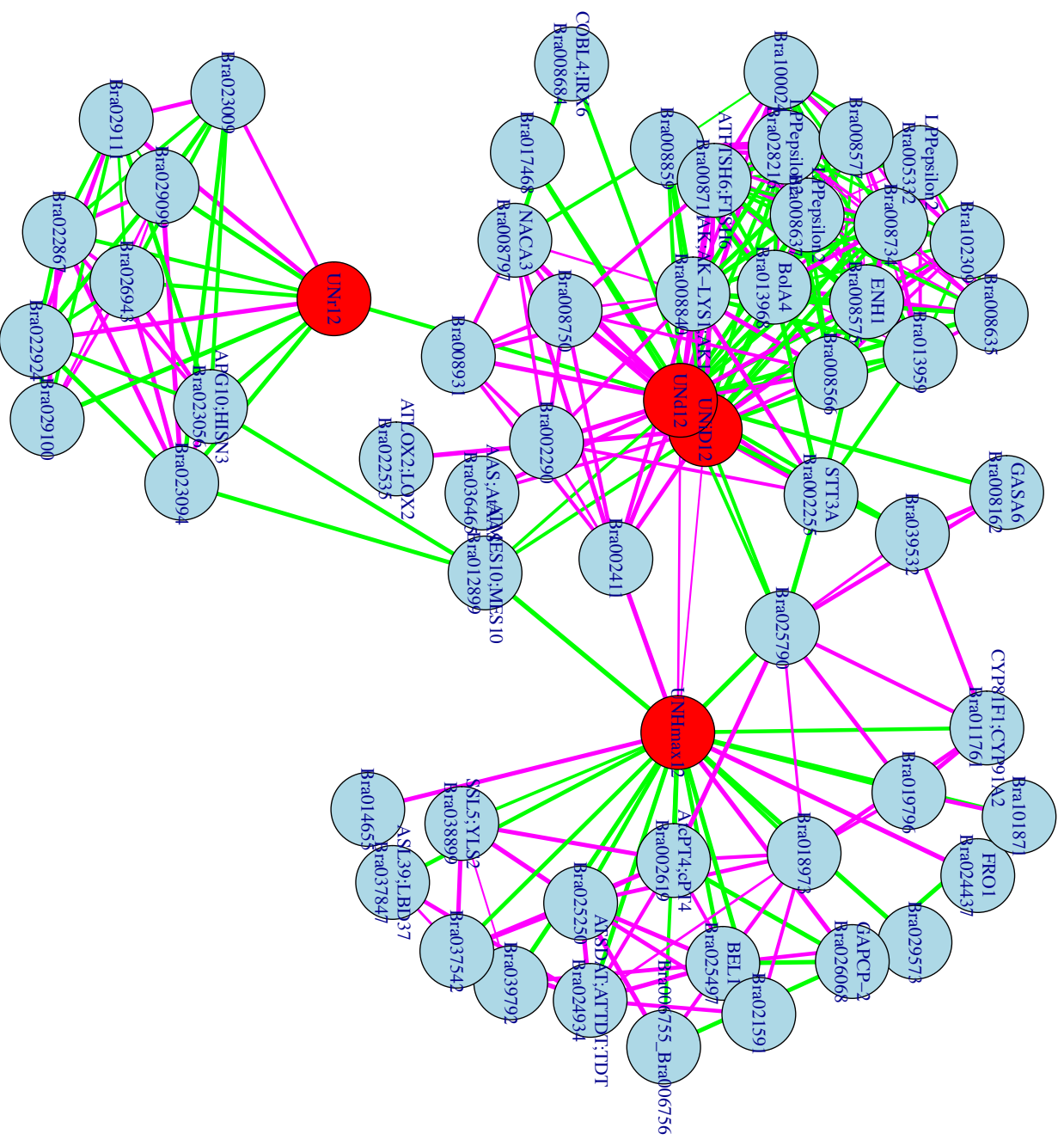

Supplement: S4 Fig — (PDF) [file pgen.1008367.s004.pdf]

MR10

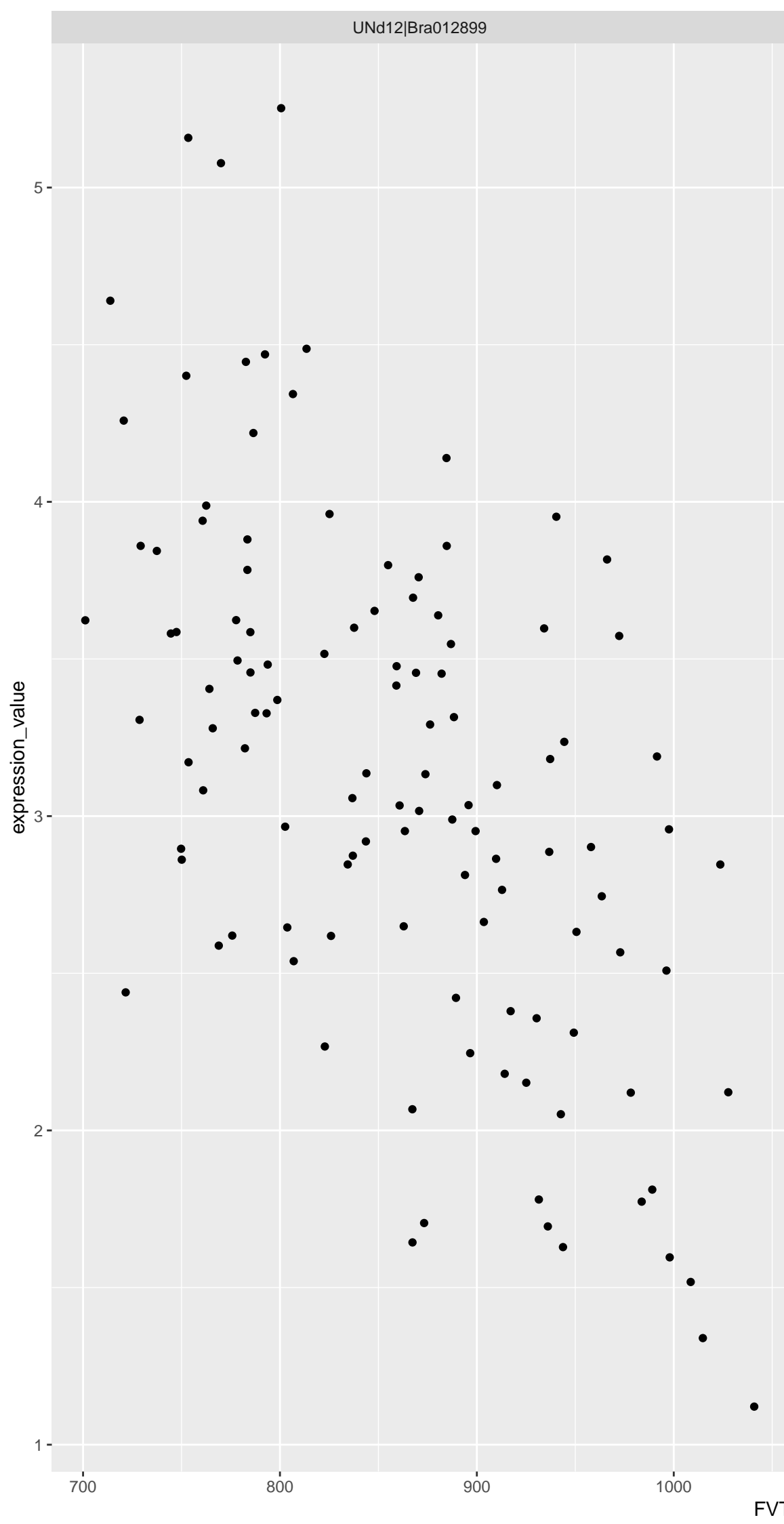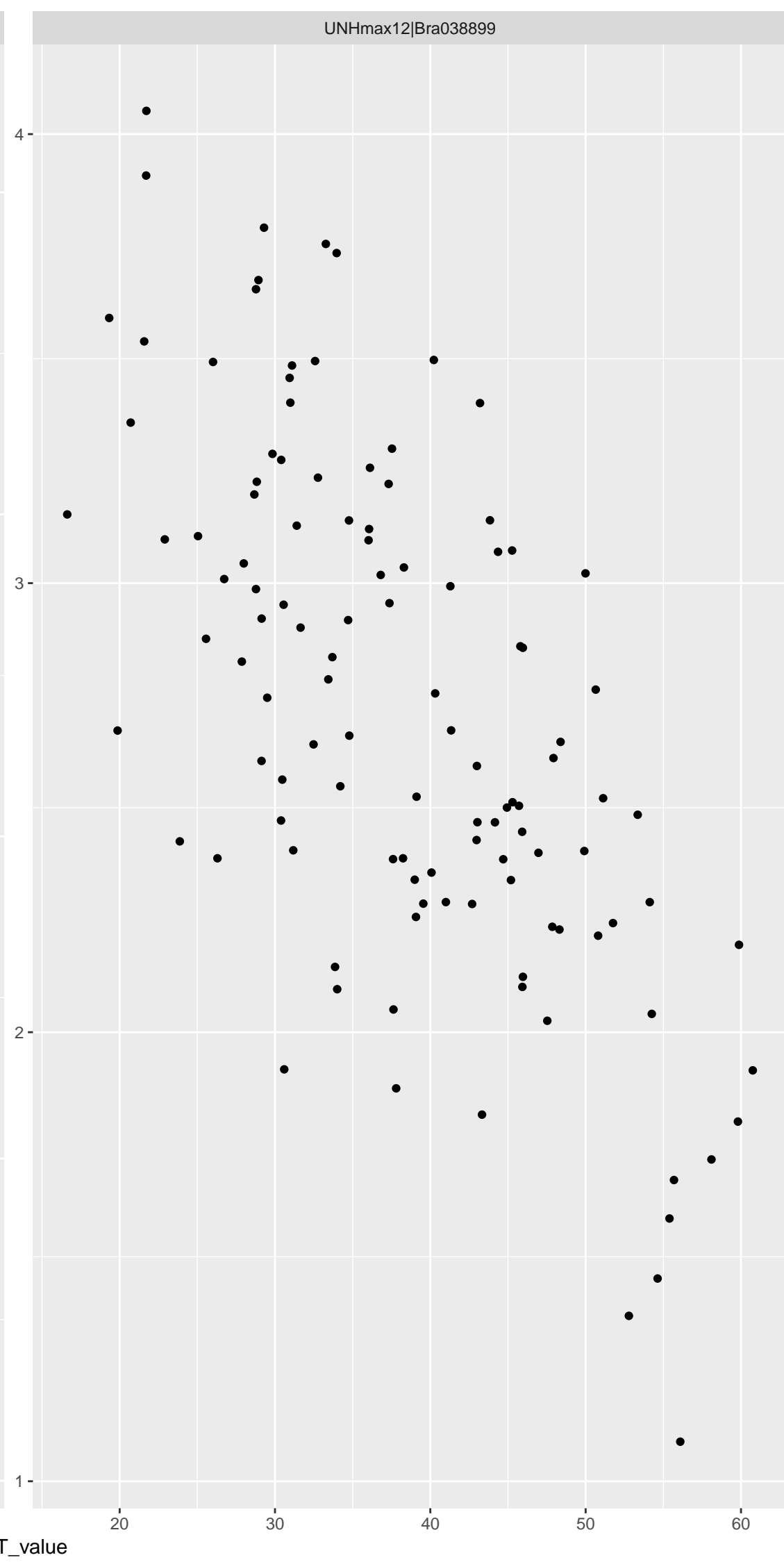

MR20

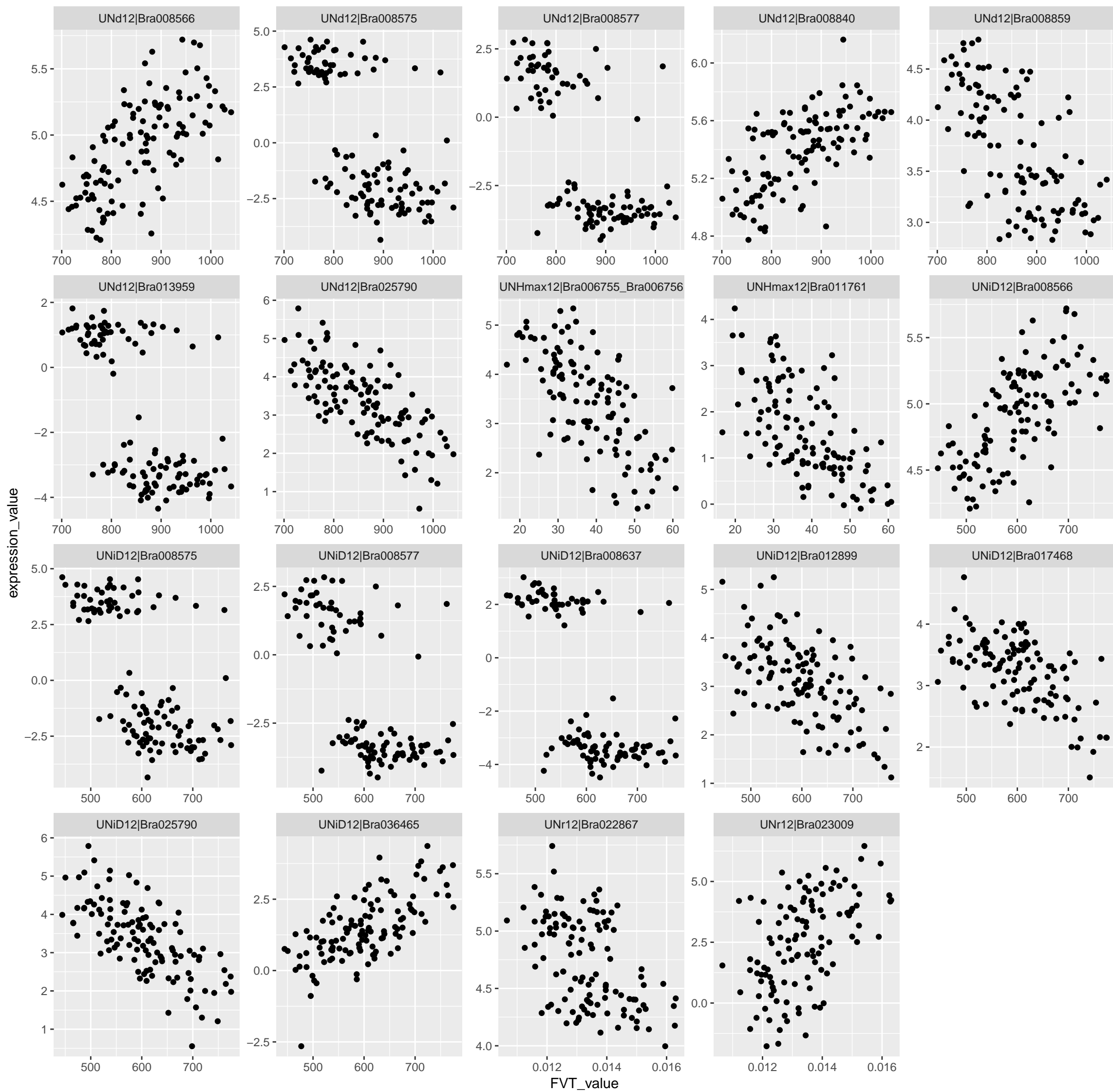

MR30

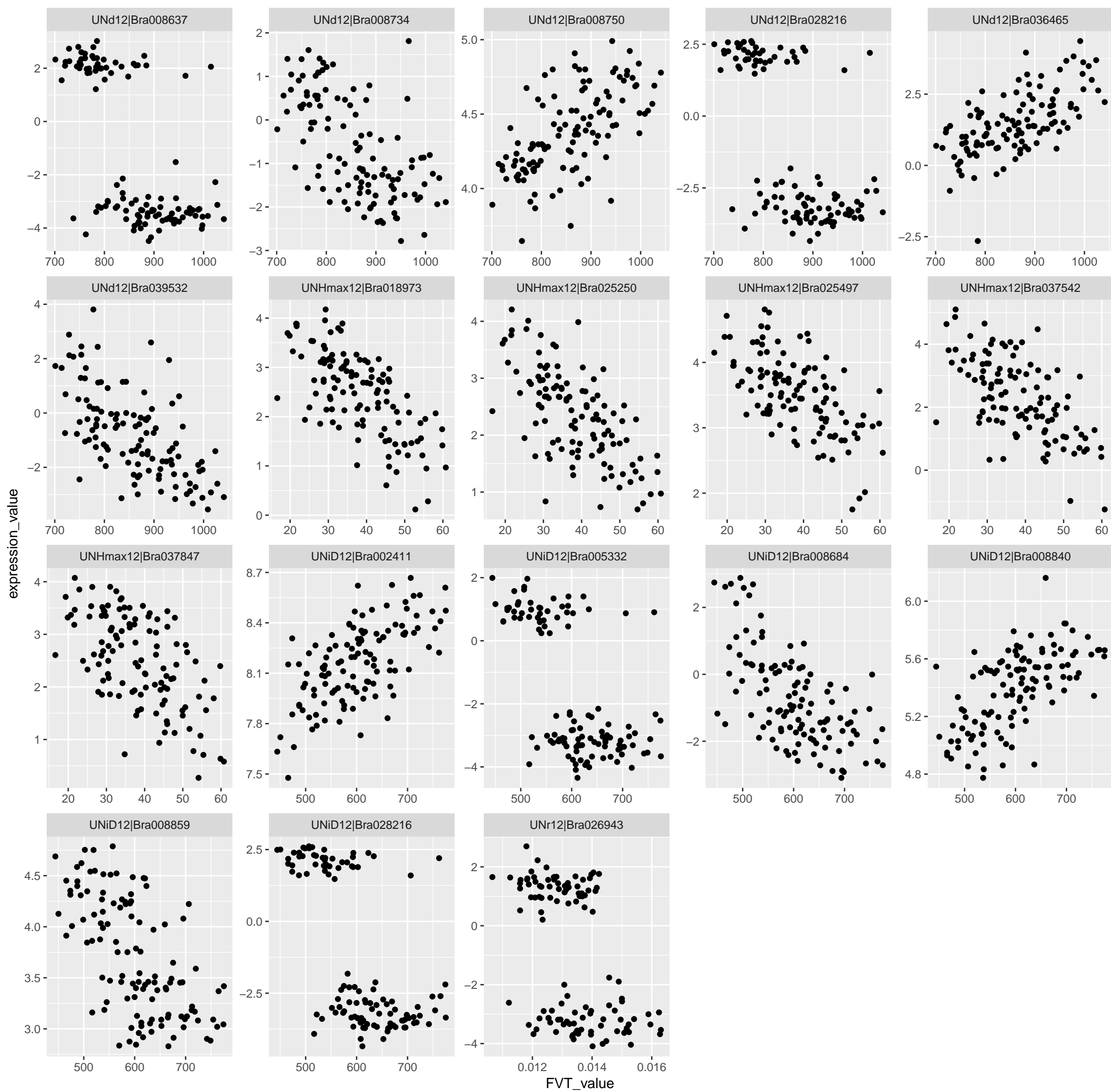

MR50

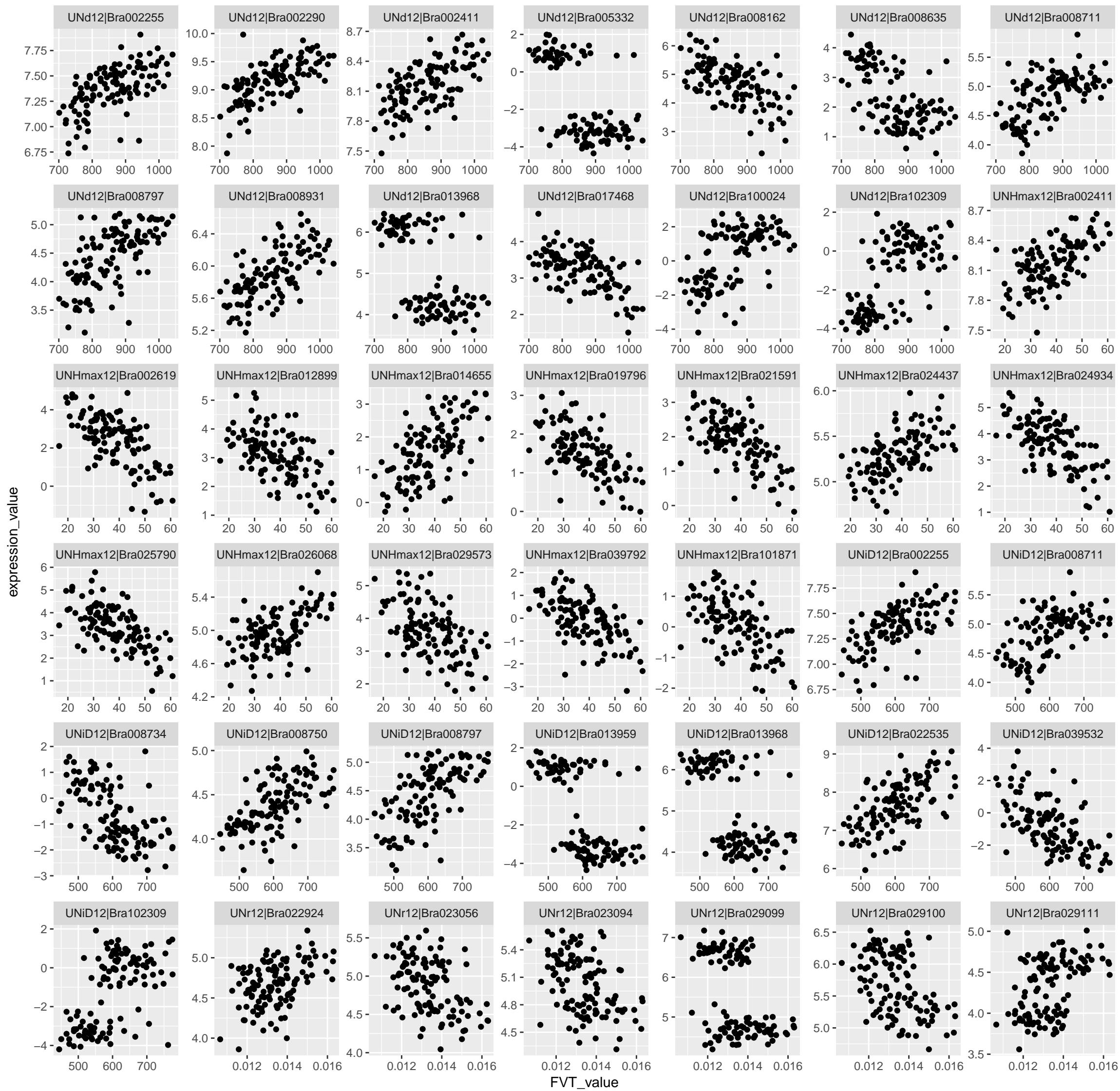

Supplement: S5 Fig — (PDF) [file pgen.1008367.s005.pdf]
