# Supplementary material for: Integrating transcriptomic network reconstruction and eQTL analyses reveals mechanistic connections between genomic architecture and Brassica rapa development
Source: PLoS Genet. 2019 Sep 12;15(9):e1008367. doi: 10.1371/journal.pgen.1008367 (PMC6759183; doi:10.1371/journal.pgen.1008367)
Supplement: S6 Fig — (DOCX) [file pgen.1008367.s006.docx]

S9. Expression trait QTL analysis (eQTL) for WGCNA-identified eigengenes that significantly correlate with UN FVT traits.
